# Catalyst control over pentavalent stereocentres

**DOI:** 10.1038/s41467-023-43750-w

**Published:** 2023-12-04

**Authors:** Anton Budeev, Jianyang Dong, Daniel Häussinger, Christof Sparr

**Affiliations:** Department of Chemistry, University of Basel, Basel, Switzerland

**Keywords:** Stereochemistry, Synthetic chemistry methodology

## Abstract

A monumental diversity of catalytic methods imparts the ability to select one of two configurations of tetravalent stereocentres. Conversely, catalyst control over pentavalent stereocentres, where a fifth moiety bound to the central atom encodes an expanded stereochemical space, remained a challenge to be accomplished. Herein, we report the feasibility of the catalytic tractability of pentavalent stereocentres. A bifunctional iminophosphorane thiourea catalyst enables enantio- and diastereocontrol over pentavalent phosphoranes to differentiate configurationally stable enantiomers and ensembles of diastereomers which emerge together from a single stereocentre. The desired dioxophosphorane stereoisomers are obtained with excellent yield and selectivity (up to 99% yield, 96:4 e.r. and 99:1 d.r.), while stereodivergent catalysis reroutes the reaction for selective access to each of the viable stereoisomeric states of pentavalent phosphoranes. Considering the diversity of high-valent main group species, it is expected that catalyst control over pentavalent stereocentres significantly increases the synthetically addressable stereochemical space.

## Abstract (web summary)

Pentavalent stereocentres encode an expanded stereochemical space, but their catalytic tractability remained unprecedented. In this report, the authors report that catalyst control over pentavalent stereocentres is feasible.

## Introduction

**Fig. 1:**
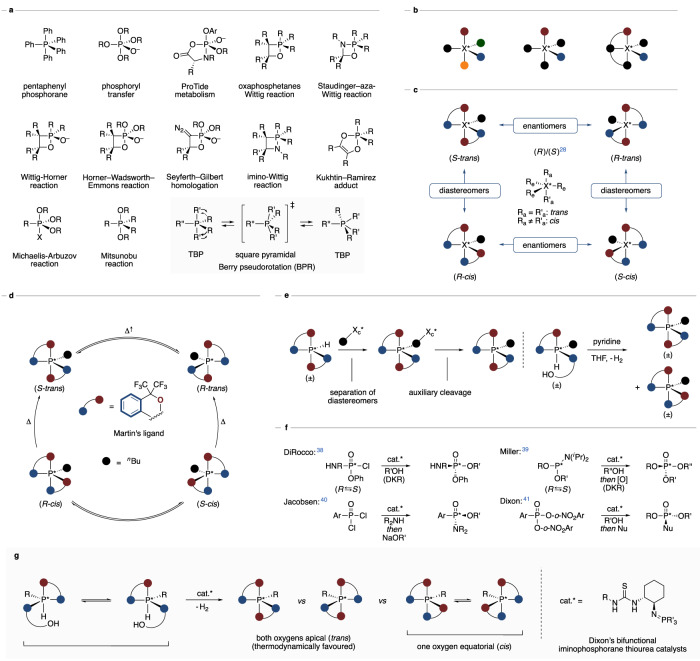
Background and concept. **a** Selected pentavalent phosphoranes. **b** Pentavalent stereocentres. **c** Fourfold stereogenicity in pentavalent stereocentres. **d** Stereoisomerisation of pentavalent dioxophosphoranes^32–37^. **e** Auxiliary (X_c_*) approaches & dehydrogenation of hydridophosphoranes. **f** Recent strategies to catalytically control tetravalent phosphorus stereocentres. **g** Catalyst control over pentavalent phosphorus stereocentres (this work).

Endeavoured by Staudinger^1^, a pentavalent phosphorane (Ph_5_P) was first prepared by Wittig and Rieber to validate their stability in 1949 (Fig. 1a)^2^. The intermediacy of pentavalent phosphoranes for phosphoryl transfer was thereupon proposed by Westheimer^3^, which recently received further significance by the metabolism of ProTides^4^. In current synthetic methodology, a great number of indispensable transformations proceed through pentavalent phosphorane intermediates, such as the oxaphosphetanes of the Wittig reaction^5^, the oxazaphosphetidines of the Staudinger–aza-Wittig reaction^6^, or the phosphoranes of the Wittig–Horner^7^, Horner–Wadsworth–Emmons^8^ and Seyferth–Gilbert reaction^9^. These validated or assumed species furthermore include the azaphosphetanes of the imino-Wittig reaction^10–12^, the Kukhtin–Ramirez adduct^13–15^, the phosphoranes of the Michaelis–Arbuzov^16^ and the Mitsunobu reaction^17^, besides intermediates of other pertinent transformations.^18–20^ Despite the ability of pentavalent phosphoranes with their trigonal bipyramidal (TBP) structure to undergo Berry pseudorotations through square pyramidal transition states^21–24^, a broad variety of configurationally stable phosphoranes was obtained for relatively congested compounds or upon bidentate binding to the phosphorus centre^18,19,25^, for instance with the Martin ligand^26–28^. In contrast to the classical Le Bel–van ’t Hoff stereoisomerism of tetravalent stereocentres that results in twofold stereogenicity^29,30^, pentavalent stereocentres commonly give rise to an increased number of isomers emerging from a single stereocentre (Fig. 1b)^31^. Even with two identical unsymmetric bidentate ligands and a fifth equatorial residue, four isomers emanate from a single phosphorane stereocentre in the form of two diastereomeric pairs of enantiomers (Fig. 1c, TBP: R_a _= apical, R_e _= equatorial). Notably, in their pioneering work, Akiba and co-workers established the configurational stability of pentavalent *trans*-dioxophosphoranes and further isolated the thermodynamically disfavoured anti-apicophilic *cis*-isomer as an ensemble of equilibrating enantiomers (Fig. 1d)^32–37^. Moreover, an auxiliary strategy with diastereomer separation allowed to prepare enantioenriched phosphoranes^32^, while hydridophosphoranes treated with pyridine were observed to provide a mixture of racemic dioxophosphorane diastereomers (Fig. 1e)^33^. In contrast to pentavalent stereocentres, remarkable recent strategies by the DiRocco^38^, Miller^39^, Jacobsen^40^ and Dixon^41^ groups enable to catalytically control tetravalent phosphorous (V) stereocentres by dynamic kinetic resolution (DKR) or desymmetrisation approaches (Fig. 1f).

From this background and with our interest in catalyst control over higher-order stereogenicity^42^, with previous emphasis on atropisomers^43,44^, overcrowded alkenes^45^ and efforts on Co-complexes^46^, we questioned if pentavalent stereocentres that encode more than two stereoisomers per stereogenic unit are catalytically addressable (Fig. 1g). More specifically, we envisioned a catalytic activation of rapidly racemising hydridophosphoranes^37^ to allow enantio- and diastereocontrol to govern the configuration of pentavalent phosphorus stereocentres. To study the tractability of pentavalent stereocentres within their extended stereochemical space, we resorted to chiral thioureas and in particular Dixon’s bifunctional iminophosphoranes^47^, as a multitude of diversified catalysts are accessible due to their high modularity and in situ accessibility.

Using iminophosphorane thioureas, we report herein that catalyst control over pentavalent phosphorus stereocentres is feasible by means of DKR with enantio- and diastereoselectivity, while rerouting allows to stereodivergently address all stable stereoisomeric forms of the desired dioxophosphoranes.

## Results

**Fig. 2:**
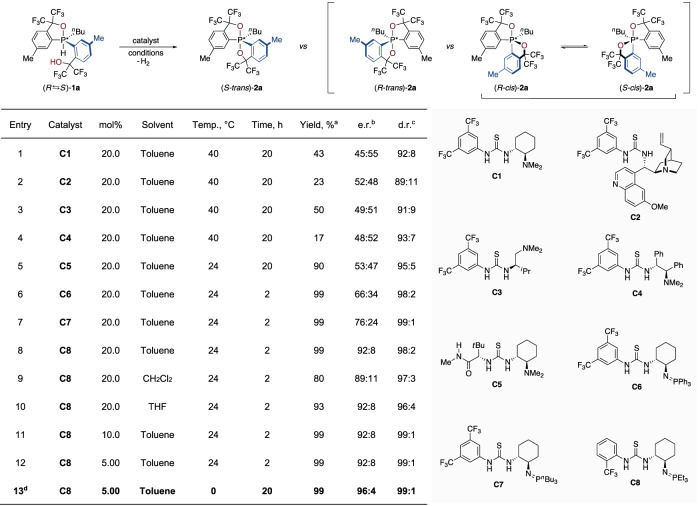
Optimisation of the reaction conditions. Conditions: **1a** (30.1 mg, 50.0 μmol), with indicated catalysts in 5.0 mL toluene. ^a^Isolated yield. ^b^Determined as (*S*):(*R*) by HPLC of isolated product on a chiral stationary phase. ^c^Determined as (*trans*):(*cis*) by ^19^F NMR analysis of the crude reaction mixture. ^d^2.5 mL instead of 5.0 mL toluene. Optimised conditions in bold.

In the method development, control over the configuration of pentavalent stereocentres involved overall the evaluation of 23 known and 103 unprecedented chiral catalysts for the stereoselective dehydrogenation of hydridophosphoranes (Fig. 2 & ESI).

The Takemoto bifunctional thiourea catalyst **C1**^48^ (entry 1) initially provided dioxophosphorane **2a** in moderate yield (43%) and good diastereoselectivity (92:8 d.r.) in favour of the *trans* isomer with a slight enantioenrichment (e.r. 45:55). An improvement by variation of the linkage between the thiourea and basic amine moiety (**C2–C4**, entries 2–4) was not observed. The Jacobsen-type catalysts possessing an additional stereocentre (**C5**^49^, entry 5) provided the product in high yield (90%), but the enantioenrichment remained modest (53:47 e.r.). However, changing the tertiary amine to Dixon’s superbasic iminophosphorane catalyst **C6**^47^ (entry 6) resulted in an increased enantioselectivity (66:34 e.r.), excellent yield (99%) and a diastereoselectivity of 98:2. Briefly, further optimisation revealed that stereocontrol correlates with the catalyst’s basicity, with more basic and reactive iminophosphoranes bearing aliphatic groups giving superior results (**C7**, entry 7, 76:24 e.r., ESI). Gratifyingly, the introduction of a key CF_3_ *ortho*-substituted aryl moiety to the thiourea and a change to a small triethyl iminophosphorane culminated in excellent selectivities (catalyst **C8**, 99% yield, 92:8 e.r., 98:2 d.r., entry 8). Variation of the solvent confirmed toluene as optimal, with CH_2_Cl_2_ and THF providing slightly diminished enantio- and diastereoselectivities (entries 9–10, ESI). We were pleased to observe that high stereocontrol was maintained with a reduced catalyst loading of 5.00 mol% (entries 11–12) and that decreasing the reaction temperature to 0 °C led to further improvement of the selectivity (96:4 e.r.) (entry 13).

**Fig. 3:**
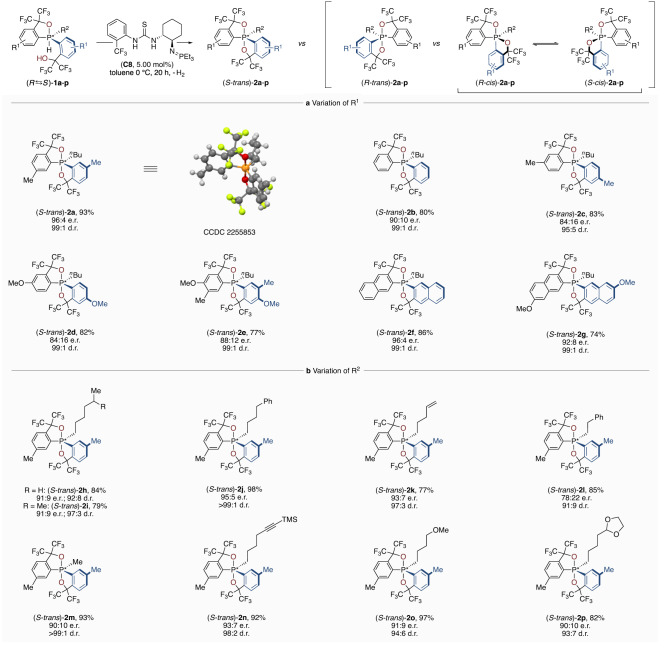
Scope of the method. Conditions: hydridophosphorane substrate **1a**-**p** (100 μmol), catalyst **C8** (5.00 μmol), 5.0 mL toluene, 0 °C, 20 h. Yields are given for isolated products. The d.r. was determined as (*trans*):(*cis*) by ^19^F NMR of the crude reaction mixture and the e.r. as (*S*):(*R*) by HPLC on a chiral stationary phase of the isolated product.

With optimised conditions in hand, we explored the scope of the catalyst control over pentavalent phosphorane stereocentres (Fig. 3). The reaction was successfully scaled to 100 μmol without affecting selectivity, providing the dioxophosphorane **2a** in 93% yield with 96:4 e.r. and 99:1 d.r. X-ray crystallographic analysis of a single crystal revealed the absolute configuration as (*S*-*trans*)-**2a**. We continued by exploring substrates with different aryl moieties at the Martin ligand to study the influence of steric and electronic effects on the outcome of the reaction. Unsubstituted (*S*-*trans*)-**2b** was obtained in excellent yield and diastereoselectivity with an e.r. of 90:10, whereas other positions at the aryl ring for (*S*-*trans*)-**2c**-**e** led to a somewhat compromised level of stereocontrol. A slightly lower enantioselectivity was observed for disubstituted (*S*-*trans*)-**2e** (88:12 e.r., 99:1 d.r.), whereas excellent stereocontrol was reached for the naphthyl congeners (*S*-*trans*)-**2f** (96:4 e.r., 99:1 d.r.) and (*S*-*trans*)-**2g** (92:8 e.r., 99:1 d.r.). We next investigated the impact of the fifth moiety bound to the phosphorus stereocentre (Fig. 3b). High yields and selectivities were obtained for the phosphoranes bearing long primary alkyl chains ((*S*-*trans*)-**2h**-**k**, (*S*-*trans*)-**2n**-**p**). Interestingly, contraction of the linkage of (*S*-*trans*)-**2l** as compared to (*S*-*trans*)-**2j** revealed that shorter chains impact the enantiodifferentiation and yield (85%). In contrast, the reaction was highly efficient (93% yield) and selective with a small methyl substituent ((*S-trans*)-**2m**, 90:10 e.r., >99:1 d.r.). Furthermore, the method is mild and compatible with several functionalities, including alkenes ((*S*-*trans*)-**2k**), alkynes ((*S*-*trans*)-**2n**), as well as ether and acetal groups ((*S*-*trans*)-**2p,o**), providing diverse phosphoranes with high yield and stereoselectivity.

**Fig. 4:**
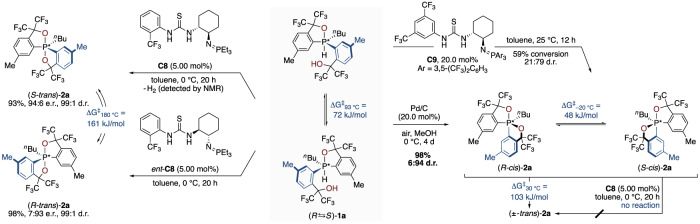
Stereodivergent catalyst control over pentavalent phosphorus stereocentres and stereodynamic behaviour. The d.r. was determined as (*trans*): (*cis*) by ^19^F NMR of the crude reaction mixture and the e.r. as (*S*):(*R*) by HPLC on a chiral stationary phase of the isolated product.

To confirm the feasibility of complete catalyst control over high-valent stereocentres, we proceeded by investigating the possibility for stereodivergent catalysis (Fig. 4). As expected, with the enantiomer of the catalyst *ent*-**C8**, the product with the opposite absolute configuration was readily obtained with inverted enantioselectivity ((*R*-*trans*)-**2a**, 3:97 e.r., 99:1 d.r.). In strong contrast, redirecting the diastereoselectivity proved to be distinctly challenging. After an extensive assessment of catalysts and conditions, we found that catalyst **C9** with electron-poor aryl groups at the iminophosphorane moiety gives rise to reversed diastereoselectivity, albeit with a comparatively low yield and level of diastereocontrol (details in the ESI). However, upon validating the generation of H_2_ by NMR, we observed that palladium catalysis (Pd/C), as an alternative dehydrogenation manifold, enables a remarkable selectivity for the (*cis*)-**2a** diastereomer. Under optimised conditions, palladium on charcoal ultimately furnished the thermodynamically disfavoured diastereomer with excellent yield and diastereoselectivity (98%, 6:94 d.r.). With access to both enantiomers of the *trans*-configured phosphorane as well as the ensemble of equilibrating enantiomers of the anti-apicophilic (*cis*)-**2a** diastereomer, the ability for stereodivergent catalyst control over pentavalent stereocentres was conclusively verified.

The nature of the obtained dioxophosphoranes was next investigated by assessing their stereodynamic behaviour. To determine the rate of enantiomerisation of the thermodynamically more stable *trans*-isomer with both oxygens placed in apical positions, a sample of enantioenriched ((*S*-*trans*)-**2a**) (94:6 e.r., 99:1 d.r.) was heated to 180 °C to measure a Δ*G*^‡^_180 °C_ of 161 kJ mol^–1^. The diastereoisomerisation of (*cis*)-**2a** to (*trans*)-**2a** in heptane at 30 °C proceeds with a barrier of Δ*G*^‡^_30 °C_ = 103 kJ mol^–1^, whereas no reaction was observed when (*cis*)-**2a** was subjected to identical *trans*-selective reaction conditions, substantiating that the *cis*-configured phosphorane is not an intermediate in the stereocontrolled synthesis of (*trans*)-**2a**. The enantiomerisation barrier of (*cis*)-**2a** determined by ^19^F VT-NMR gave a Δ*G*^‡^_–20 °C_ of 48 kJ mol^–1^, which underscores the rapid interconversion of this ensemble of enantiomers in agreement with the data reported by Akiba and co-workers^37^. Moreover, the interconversion rate of the two enantiomers of the starting material **1a** was measured by coalescence ^19^F VT-NMR, giving a Δ*G*^‡^_93 °C_ = 72 kJ mol^–1^ to verify that a DKR takes place during the catalyst-controlled synthesis of pentavalent phosphorane stereocentres.

In conclusion, the feasibility of catalyst control over pentavalent stereocentres was established by employing bifunctional iminophosphorane thiourea catalysis for the stereoselective synthesis of dioxophosphoranes, rendering an extended stereochemical space emerging from a single stereocentre synthetically addressable. Control over each viable stereoisomer was attained by a stereodivergent approach, with the diastereo- and enantiomers selectable by the choice of catalyst. Furthermore, the DKR of the substrates and the stereodynamic nature of the products were validated. It is thus anticipated that the significantly increased stereochemical space of the diverse high-valent main group species is rendered synthetically accessible with catalyst control over pentavalent stereocentres.

## Methods

### Enantio- and *trans*-diastereoselective catalyst control over pentavalent stereocentres

To a mixture of the hydridophosphorane substrate **1a**-**p** (100 μmol, 1.00 eq.) and catalyst **C8** (2.17 mg, 5.00 μmol, 5.00 mol%) in a dried 20 mL crimp cap vial under an Ar atmosphere at 0 °C was added toluene (5.0 mL). The mixture was stirred at 0 °C for 20 h and the solvent removed under reduced pressure at 10 °C to 20 °C. The d.r. was determined by ^19^F NMR of the residue, before it was purified by silica gel flash column chromatography (230–400 mesh) to isolate the desired product. The e.r. of the isolated product was measured by HPLC on a chiral stationary phase.

### Stereodivergent *cis*-selective catalyst control

To a mixture of the hydridophosphorane substrate **1a** (60.2 mg, 100 μmol, 1.00 eq.) and Pd/C (5.00 % wt, 42.6 mg, 20.0 μmol, 20.0 mol%) in a 20 mL crimp cap vial at 0 °C under ambient atmosphere was added MeOH (HPLC-grade, 10 mL). Three needles were inserted through the cap of the vial for gas exchange with the open atmosphere. The mixture was stirred at 0 °C for 4 days, filtered through a short pad of silica gel (230 – 400 mesh) and the silica gel was washed with MeOH (2 ×5.0 mL). The solvent was removed under reduced pressure at 10 °C–20 °C to afford the title compound as a white solid (59.0 mg, 98.3 μmol, 98%, 6:94 d.r.). At room temperature, the product undergoes gradual interconversion to (±-*trans*)-**2a** and was therefore stored at −20 °C.

### Supplementary information

SI

Peer Review File

## Data Availability

All data are available in the main text and Supplementary Information, including supplementary methods, experimental details, NMR spectra and crystallographic data. Supplementary crystallographic data for this paper can be obtained from the Cambridge Crystallographic Data Centre at www.ccdc.cam.ac.uk/structures (CCDC 2255853).
