# Supplementary material for: Catalyst control over pentavalent stereocentres
Source: Nat Commun. 2023 Dec 4;14:8013. doi: 10.1038/s41467-023-43750-w (PMC10696079; doi:10.1038/s41467-023-43750-w)
Supplement: Supplementary file 1 — SI [file 41467_2023_43750_MOESM1_ESM.pdf]

Supplementary Information

Catalyst control over pentavalent stereocentres

Anton Budeev[1], Jianyang Dong[1], Daniel Häussinger[1], Christof Sparr[1]*

[1] Department of Chemistry, University of Basel

* Email: christof.sparr@unibas.ch

Table of contents

Supplementary Methods 2

General information 2

Substrate synthesis 3

Optimisation of the reaction conditions for *trans*-/enantioselective catalyst control over pentavalent stereocentres 25

Initial catalyst, solvent, and temperature evaluation 25

Final optimisation 39

Catalyst synthesis 41

General procedure for catalyst control over pentavalent stereocentres 43

Stereodivergent control over pentavalent stereocentres 55

Synthesis of ((*R*-*trans*)-**2a**) 55

Optimisation of the reaction conditions for diastereodivergent control over pentavalent stereocentres: *cis*-diastereoselective bifunctional iminophosphorane-thiourea catalysed reaction 56

Evaluation of the conditions for *cis*-selective Pd-catalysed reaction 58

Evaluation of the configurational stabilities 62

Reactivity of (*cis*)-2a 68

X-ray crystallographic analysis 70

HPLC traces 73

NMR spectra 90

Supplementary References 202

Supplementary Methods

General information

All chemicals were reagent grade (Acros Organics, Alfa Aesar, Apollo, Fluka, Fluorochem, Riedel-de Häen, Sigma-Aldrich, TCI) and used as supplied. All reactions were carried out in dried glassware under an Ar atmosphere unless stated otherwise. Toluene (99.5%, Extra Dry, over Molecular Sieves, Stabilized, AcroSeal®, Code: 364415000) and CH_2Cl_2 (99.5%, Extra Dry, over Molecular Sieves, Stabilized, AcroSeal®, Code: 348465000) were purchased from Acros Organics. Petroleum ether (40-60 °C) was purchased from Biosolve. Extracts were dried over technical grade Na_2SO_4. Analytical thin layer chromatography (TLC) was performed on pre-coated Merck silica gel 60 F254 plates (0.25 mm) and visualised by UV. Flash column chromatography was carried out on Silicycle SiliaFlash P60 (230 – 400 mesh). Concentration *in vacuo* was performed by rotary evaporation to ~ 10 mbar at 40 °C, drying at ~ 0.5 mbar and at r.t. Optical rotations of enantioenriched diastereomeric mixtures were obtained at r.t. on a Jasco P-2000 polarimeter at 589 nm using a 1.00 mL cell with a length of 100 mm; optical rotations are reported in (°·mL)/(g·dm), concentrations in g/100 mL. IR spectra were measured on an ATR *Varian Scimitar 800* FT-IR spectrometer and are reported in cm^{-1}. The intensities of the bands are reported as: w = weak, m = medium, s = strong. ^{1}H NMR and ^{13}C NMR spectra were recorded on a *Bruker Avance III* 500 MHz or 400 MHz spectrometer at 298 K in $CDCl_3$ supplied by *Cambridge Isotope Laboratories* (DLM-7TB-100S). Chemical shifts (δ) are reported in ppm relative to tetramethylsilane (0.00 ppm). The multiplicities are reported in Hz as: s = singlet, br = broad singlet, d = doublet, t = triplet, q = quartet and m = multiplet. DEPT135 and 2-dimensional experiments (COSY, HMBC, HMQC, NOESY, TOCSY) were used to support assignments but are not included in this document. High-resolution spectrometry (HR-ESI) was performed by Dr. Michael Pfeffer of the University of Basel on a *Bruker maXis 4G* QTOF ESI mass spectrometer. X-ray crystallography was performed by Dr. Alessandro Prescimone on a *Bruker Kappa Apex 2* or a *Stoe StadiVari* diffractometer. Enantiomeric ratios were determined by HPLC on a chiral stationary phase using the indicated analytical columns and eluents with the retention times (R_t) reported in minutes. The phosphorane compounds were named according to previous literature[1] and the IUPAC rules (Nomenclature of Inorganic Chemistry IUPAC Recommendations 2005, sections 9.3.2, 9.3.3 and 9.3.4)[2].

Substrate synthesis

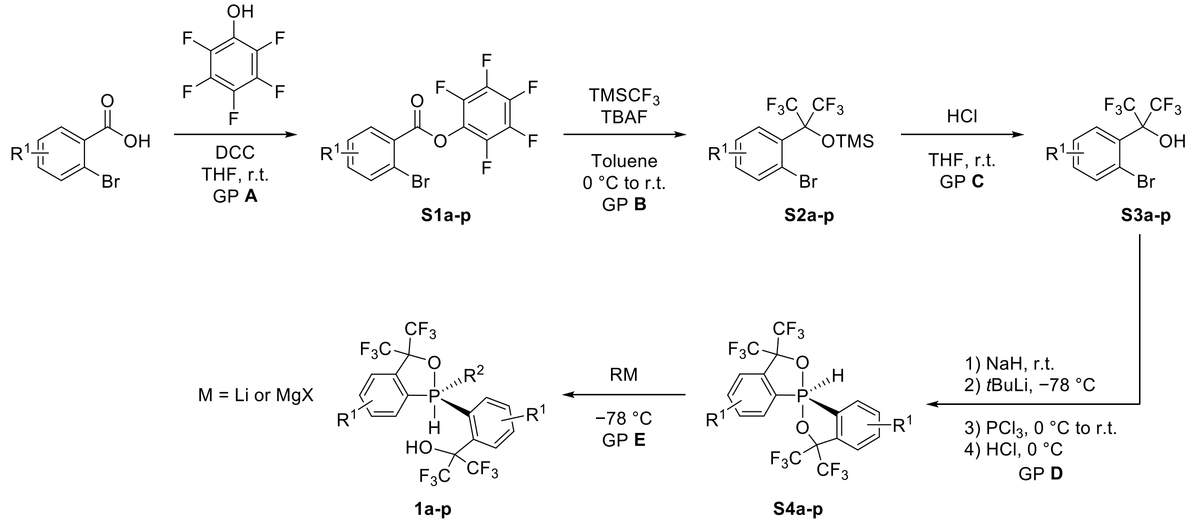

General Procedure A: Synthesis of pentafluorophenyl esters[3]

To a stirred solution of carboxylic acid (1.00 eq.) and pentafluorophenol (1.12 eq.) in THF (0.27 $molL^{-1}$) was added *N,N'*-dicyclohexylcarbodiimide (1.13 eq.) at r.t. The progress of the reaction was monitored by TLC (petroleum ether/EtOAc mixture). The *N,N'*-dicyclohexylurea was removed by filtration and the filtrate was evaporated under reduced pressure. The residue was purified by flash column chromatography on silica gel to afford the desired product.

General Procedure B: Addition of the CF_3 moieties[3]

To a stirred solution of pentafluorophenyl ester (**S1**, 1.00 eq.) in toluene (0.20 $molL^{-1}$) was added trimethyl(trifluoromethyl)silane (6.00 eq.). The reaction mixture was cooled to 0 °C and a solution of tetrabutylammonium fluoride (1.00 $molL^{-1}$ in THF, 0.35 eq.) was added dropwise. The reaction mixture was allowed to reach r.t. and stirred for 18 h. The reaction was monitored by TLC (petroleum ether), and, if necessary, additional trimethyl(trifluoromethyl)silane was added. Et_2O was added and the organic phase was washed with aqueous HCl (1.00 $molL^{-1}$, 30.0 mL). The aqueous phase was extracted with Et_2O (2 x 50 mL). The combined organic phases were washed with H_2O (80 mL), dried over Na_2SO_4 and concentrated under reduced pressure. The residue was purified by flash column chromatography on silica gel to afford the desired product, for some reactions, purification was not required and the next step was carried out directly.

General Procedure C: Synthesis of hexafluoropropan-2-ols[3]

To a stirred solution of trimethyl[2,2,2-trifluoro-1-organyl-1-(trifluoromethyl)ethoxy]silane (**S2**, 1.00 eq.) in THF (0.20 $molL^{-1}$) was added 6.00 $molL^{-1}$ HCl (half volume of THF). The reaction mixture was stirred overnight at r.t. The mixture was diluted with H_2O (50 mL) and the product was extracted with Et_2O (2 x 40 mL). The organic phases were combined, dried over Na_2SO_4 and concentrated under reduced pressure. The residue was purified by flash column chromatography on silica gel to afford the desired product.

General Procedure D: Attachment of the ligands[4]

To a stirred solution of hexafluoropropan-2-ol (**S3**, 1.00 eq.) in diethyl ether (0.30 $molL^{-1}$) was added NaH (60% in mineral oil, 1.10 eq.) under an Ar atmosphere at r.t. The mixture was stirred for 30 min and cooled to −78 °C. A solution of ^{t}BuLi (1.70 $molL^{-1}$ in pentane, 2.20 eq.) was added dropwise and the reaction was warmed to 0 °C. PCl_3 (0.50 eq.) was added dropwise and the reaction mixture was allowed to warm to r.t. and stirred for 16 h. The reaction was quenched with HCl (6.00 $molL^{-1}$, 10.0 mL) and extracted with Et_2O (3 x 40 mL). The organic phase was washed with brine (60 mL), dried over Na_2SO_4 and concentrated under reduced pressure. The residue was purified by flash column chromatography on silica gel to afford the desired product.

General Procedure E: Synthesis of hydridophosphorane substrates with commercially available organometallic reagents[1]

Organometallic reagent (7.00 eq.) was added to a solution of precursor **S4** (1.00 eq.) in dry Et_2O (0.10 $molL^{-1}$) under an Ar atmosphere at −78 °C. The cooling bath was removed and the solution was stirred at r.t. for 2 h. The solution was quenched with HCl (1.00 $molL^{-1}$, 7.00 mL) at 0 °C and extracted with Et_2O (3 x 10 mL). The organic phase was washed with brine (25 mL), dried over Na_2SO_4 and concentrated under reduced pressure. The residue was purified by flash column chromatography on silica gel (solvent precooled in a freezer (−20 °C) was used) to afford the desired product. The hydridophosphoranes thus obtained are unstable, and dehydrogenation by-products are generated during column chromatography and NMR analysis. Therefore, the product was used in the next step directly after the purification. For long-term storage, the product should be kept in a freezer (−20 °C) under an Ar atmosphere.

General Procedure F: Synthesis of hydridophosphorane substrates with Grignard reagents prepared in situ

Grignard reagents were prepared according to a modified literature procedure.[5] To a 10 mL crimp cap vial containing magnesium (7.10 eq.), a solution of bromide (7.00 eq.) in dry THF (1.08 $molL^{-1}$) was added under an Ar atmosphere, followed by 3 drops of dibromoethane. The reaction mixture was stirred for 10 min at r.t., followed by 1.5 h at 55 °C. The mixture was cooled to –78 °C and precursor **S4** (1.00 eq.) was added as a solution in dry THF (0.15 $molL^{-1}$) under an Ar atmosphere. The cooling bath was removed and the solution was stirred at r.t. for 2 h. The reaction mixture was quenched with HCl (1.00 $molL^{-1}$, 5.00 mL) at 0 °C and extracted with Et_2O (3 x 10 mL). The organic phase was washed with brine (5.0 mL), dried over Na_2SO_4 and concentrated under reduced pressure. The residue was purified by flash column chromatography on silica gel (solvent precooled in a freezer (−20 °C) was used) to afford the desired product. The hydridophosphoranes thus obtained are unstable, and dehydrogenation by-products are generated during column chromatography and NMR analysis. Therefore, the product was used in the next step directly after the purification. For long-term storage, the product should be kept in a freezer (−20 °C) under an Ar atmosphere.

Compound **1b** was prepared according to a literature reported procedure.[1]

2,3,4,5,6-Pentafluorophenyl 2-bromo-4-methylbenzoate (S1a)

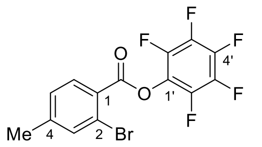

Following the general procedure **A**, the reaction of 2-bromo-4-methylbenzoic acid (1.72 g, 8.00 mmol, 1.00 eq.), pentafluorophenol (1.65 g, 8.96 mmol, 1.12 eq.) and *N,N'*-dicyclohexylcarbodiimide (1.87 g, 9.04 mmol, 1.13 eq.) in THF (30.0 mL) provided the title compound after column chromatography on silica gel (petroleum ether) as white crystals (2.68 g, 7.00 mmol, 88%, m.p. = 63 – 64 °C); R_f 0.30 (petroleum ether); IR $\tilde{\nu}_{max}$ (neat): 2972w, 1768m, 1600w, 1518s, 1232m, 1067m, 1011s, 827w, 754w, 678w; ^{1}H NMR (500 MHz, $CDCl_3$) δ 8.04 (d, $J_{H\text{-}H}$ = 8.2 Hz, 1H, C6*H*), 7.61 (s, 1H, C3*H*), 7.27 (d, $^3J_{H\text{-}H}$ = 7.8 Hz, 1H, C5*H*), 2.43 (s, 3H, C4C*H*$_3$); ^{13}C{^{1}H} NMR (126 MHz, $CDCl_3$) δ 161.3 (*C*O$_2$R), 146.0 (*C*4), 142.3 (C_6F_5-*C*), 140.6 (C_6F_5-*C*), 140.4 (C_6F_5-*C*), 138.9 (C_6F_5-*C*), 138.6 (C_6F_5-*C*), 136.9 (C_6F_5-*C*), 135.8 (*C*3), 132.7 (*C*6), 128.3 (*C*5), 125.1 (*C*1), 123.5 (*C*2), 21.3 (C4*C*H$_3$); ^{19}F{^{1}H} NMR (376 MHz, $CDCl_3$) δ −149.4 – −154.1 (m, 2F, C2'*F and* C6'*F*), −157.7 (t, ^3J = 21.7 Hz, 1F, C4'*F*), −160.1 – −164.0 (m, 2F, C3'*F and* C5'*F*); HRMS (ESI$^-$): *m/z* calcd. 212.9556 for ([M−C_6F_5]$^-$), found 212.9557.

((2-(2-Bromo-4-methylphenyl)-1,1,1,3,3,3-hexafluoropropan-2-yl)oxy)trimethylsilane (S2a)

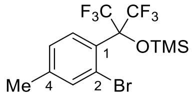

Following the general procedure **B**, the reaction of pentafluorophenyl ester **S1a** (1.34 g, 3.53 mmol, 1.00 eq.), trimethyl(trifluoromethyl)silane (3.10 mL, 21.2 mmol, 6.00 eq.) and tetrabutylammonium fluoride (1.00 molL^{-1} in THF, 1.24 mL, 1.24 mmol, 0.35 eq.) in toluene (18.0 mL) provided the title compound after column chromatography on silica gel (petroleum ether) as colourless oil (1.00 g, 2.44 mmol, 69%); R_f 0.80 (petroleum ether); IR $\tilde{\nu}_{max}$ (neat): 2968m, 2361m, 1709w, 1522w, 1381w, 1196s, 1147m, 1052m, 945m, 762w, 731w, 696w; ^{1}H NMR (500 MHz, $CDCl_3$) δ 7.58 – 7.54 (m, 1H, C3*H*), 7.52 (d, $^3J_{H\text{-}H}$ = 8.4 Hz, 1H, C6*H*), 7.12 (dd, $^3J_{H\text{-}H}$ = 8.3 Hz, $^4J_{H\text{-}H}$ = 2.0 Hz, 1H, C5*H*), 2.30 (s, 3H, C4C*H*$_3$), 0.23 (s, 9H, Si(C*H*$_3$)$_3$); ^{13}C{^{1}H} NMR (126 MHz, $CDCl_3$) δ 141.6 (*C*4), 137.9 (*C*3), 129.8 (p, J = 3.1 Hz, *C*1), 128.0 (*C*6), 127.3 (*C*5), 123.0 (q, $^1J_{C\text{-}F}$ = 293.0 Hz, 2C, C(*C*F$_3$)$_2$), 122.3 (*C*2), 81.9 (hept, $^2J_{C\text{-}F}$ = 30.0 Hz, *C*(CF$_3$)$_2$), 20.4 (Ar*C*H$_3$), 1.6 (Si(*C*H$_3$)$_3$); ^{19}F{^{1}H} NMR (470 MHz, $CDCl_3$) δ −70.6 (6F, C(C*F*$_3$)$_2$). HRMS (ESI$^-$): *m/z* calcd. 334.9512 for ([M−TMS]$^-$), found 334.9517.

2-(2-Bromo-4-methylphenyl)-1,1,1,3,3,3-hexafluoropropan-2-ol (S3a)

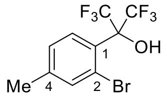

Following the general procedure **C**, the reaction of crude **S2a** (3.53 mmol, 1.00 eq.) in THF (18.0 mL) and hydrochloric acid (12.0 molL^{-1}, 18.0 mL) provided the title compound after column chromatography on silica gel (petroleum ether/EtOAc 5:1) as yellow oil (0.92 g, 2.73 mmol, 77%); R_f 0.30 (petroleum ether/EtOAc 5:1); IR $\tilde{\nu}_{max}$ (neat): 3700m, 2977s, 2939s, 2361m, 1345w, 1054s, 1033m, 1013m, 785m, 668w, 632w; ^{1}H NMR (400 MHz, $CDCl_3$) δ 7.58 (d, $^3J_{H\text{-}H}$ = 8.4 Hz, 1H, C6*H*), 7.53 (s, 1H, C3*H*), 7.22 (d, $^3J_{H\text{-}H}$ = 8.4 Hz, 1H, C5*H*), 5.37 (br. s, 1H, O*H*), 2.36 (s, 3H, C4C*H*$_3$); ^{13}C{^{1}H} NMR (151 MHz, $CDCl_3$) δ 142.4 (*C*4), 137.1 (*C*3), 130.2 (*C*1), 128.8 (*C*6),

124.4 ($C5$), 122.7 (q, $^{1}J_{C\text{-}F}$ = 289.8 Hz, 2C, C(CF_3)$_2$), 120.1 ($C2$), 80.1 (hept, $^{2}J_{C\text{-}F}$ = 30.0 Hz, C(CF_3)$_2$), 20.5 (C4CH_3); ^{19}F{^{1}H} NMR (376 MHz, $CDCl_3$) δ −73.8 (6F, C(CF_3)$_2$); HRMS (ESI$^-$): *m/z* calcd. 334.9512 for ([M−H]$^-$), found 334.9518.

[*TBPY*-5-11']-6,6'-Dimethyl-1-hydro-3,3,3',3'-tetrakis(trifluoromethyl)-3*H*,3'*H*-1λ^5,1'-spirobi[benzo[*c*][2,1]oxaphosphole] (S4a)

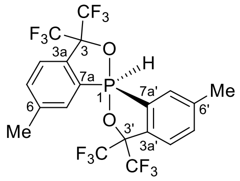

Following the general procedure **D**, the reaction of alcohol **S3a** (0.61 g, 1.81 mmol, 1.00 equiv.), NaH (47.8 mg, 1.99 mmol, 1.10 eq.), ^{t}BuLi (1.70 molL^{-1} in pentane, 2.40 mL, 3.98 mmol, 2.20 eq.) and PCl_3 (79.0 μL, 0.90 mmol, 0.50 eq.) in Et_2O (18.0 mL) provided the title compound after column chromatography on silica gel (petroleum ether) as white crystals (154 mg, 0.28 mmol, 31%, m.p. = 145 – 146 °C); R_f 0.60 (petroleum ether); IR $\tilde{\nu}_{max}$ (neat): 2925m, 2361m, 1277m, 1196s, 1152m, 1078m, 972s, 895m, 825m, 757m, 703m, 640w; ^{1}H NMR (400 MHz, $CDCl_3$) δ 8.32 (d, $^{2}J_{H\text{-}P}$ = 728.5 Hz, 1H, P*H*), 8.06 (d, $J_{H\text{-}H}$ = 12.5 Hz, 2H, C4*H* and C4'*H*), 7.66 (s, 2H, C7*H* and C7'*H*), 7.54 (d, $^{3}J_{H\text{-}H}$ = 7.9 Hz, 2H, C5*H* and C5'*H*), 2.48 (s, 6H, C6CH_3 and C6'CH_3); ^{13}C{^{1}H} NMR (126 MHz, $CDCl_3$) δ 142.4 (d, $^{3}J_{C\text{-}P}$ = 14.4 Hz, *C*6 and *C*6'), 137.1 (d, $^{2}J_{C\text{-}P}$ = 10.4 Hz, *C*7 and *C*7'), 135.3 (d, $^{4}J_{C\text{-}P}$ = 3.5 Hz, *C*5 and *C*5'), 134.8 (d, $^{3}J_{C\text{-}P}$ = 21.5 Hz, *C*4 and *C*4'), 126.7 (d, $^{1}J_{C\text{-}P}$ = 156.7 Hz, *C*7a and *C*7a'), 125.0 (d, $^{2}J_{C\text{-}P}$ = 16.9 Hz, *C*3a and *C*3a'), 122.7 (q, $^{1}J_{C\text{-}F}$ = 287.2 Hz, 2C, C3CF_3 and C3'CF_3), 122.5 (q, $^{1}J_{C\text{-}F}$ = 286.7 Hz, 2C, C3CF_3 and C3'CF_3), 82.0 (hept, $^{2}J_{C\text{-}F}$ = 31.1 Hz, 2C, *C*3 and *C*3'), 21.5 (C6CH_3 and C6'CH_3); ^{19}F{^{1}H} NMR (470 MHz, $CDCl_3$) δ −74.7 (q, $^{4}J_{F\text{-}F}$ = 9.1 Hz, 6F, C3CF_3 and C3'CF_3), −76.0 (qd, $^{4}J_{F\text{-}F}$ = 9.1, 3.2 Hz, 6F, C3CF_3 and C3'CF_3); ^{31}P{^{1}H} NMR (202 MHz, $CDCl_3$) δ −46.6; HRMS (ESI$^-$): *m/z* calcd. 543.0389 for ([M−H]$^-$), found 543.0398.

[*TBPY*-5-15]-1,1,1,3,3,3-Hexafluoro-2-(2-(1-butyl-1-hydro-6-methyl-3,3-bis(trifluoromethyl)-3*H*-1λ^5-benzo[*c*][2,1]oxaphosphol-1-yl)-4-methylphenyl)propan-2-ol (1a)

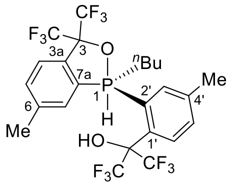

Following the general procedure **E**, the reaction of **S4a** (154 mg, 0.28 mmol, 1.00 eq.) and ^{n}BuLi (1.60 molL^{-1} in hexanes, 1.24 mL, 1.98 mmol, 7.00 eq.) in Et_2O (3.0 mL) provided the title compound after column chromatography on silica gel (petroleum ether) as white crystals (129 mg, 0.21 mmol, 76%, m.p. = 108 – 109 °C (decomposition)); R_f 0.20 (petroleum ether); IR $\tilde{\nu}_{max}$ (neat): 3307m, 2943m, 2830m, 2361m, 1768m, 1518s, 1450w, 1253w, 1190m, 1135w, 1122s, 944m, 890w, 650w; ^{1}H NMR (500 MHz, $CDCl_3$) δ 9.11 (br. s, 1H, O*H*), 7.81 – 7.69 (m, 2H, Ar-C*H*), 7.65 (t, *J* = 5.1 Hz, 1H, Ar-C*H*), 7.59 (d, *J* = 8.0 Hz, 1H, Ar-C*H*), 7.28 – 7.19 (m, 2H, Ar-C*H*), 6.02 (dd, $^{1}J_{H\text{-}P}$ = 272.7, 5.8 Hz, 1H, P*H*), 3.17 – 3.01 (m, 1H, ^{n}Bu-C*H*), 2.60 (s, 3H, C6CH_3 *or* C4'CH_3), 2.57 – 2.45 (m, 1H, ^{n}Bu-C*H*), 2.25 (s, 3H, C6CH_3 *or* C4'CH_3), 1.56 – 1.45 (m, 1H, ^{n}Bu-C*H*), 1.38 (qd, *J* = 7.2,

2.2 Hz, 2H, ^{n}Bu-CH), 1.31 – 1.24 (m, 1H, ^{n}Bu-CH), 0.87 (t, $^{3}J_{H\text{-}H}$ = 7.3 Hz, 3H, ^{n}Bu-CH_3); ^{19}F{^{1}H} NMR (470 MHz, CDCl$_3$) δ −72.6 (s, 3F, CF_3), −75.8 (q, $^{4}J_{F\text{-}F}$ = 9.2 Hz, 3F, CF_3), −76.3 (q, $^{4}J_{F\text{-}F}$ = 8.8 Hz, 3F, CF_3), −76.6 (dq, $^{4}J_{F\text{-}F}$ = 9.9, 4.9 Hz, 3F, CF_3); ^{31}P{^{1}H} NMR (202 MHz, CDCl$_3$) δ −35.0. HRMS (ESI^{+}): *m/z* calcd. 603.1317 for ([M+H]$^{+}$), found 603.1329.

2,3,4,5,6-Pentafluorophenyl 2-bromo-5-methylbenzoate (S1c)

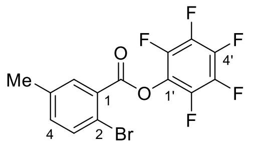

Following the general procedure **A**, the reaction of 2-bromobenzoic acid (1.61 g, 8.00 mmol, 1.00 eq.), pentafluorophenol (1.65 g, 8.96 mmol, 1.12 eq.) and *N,N'*-dicyclohexylcarbodiimide (1.87 g, 9.04 mmol, 1.13 eq.) in THF (30 mL) provided the title compound after column chromatography on silica gel (petroleum ether) as white crystals (2.66 g, 7.25 mmol, 91%). R$_f$ 0.30 (petroleum ether). The spectral data were in accordance with those previously reported in the literature.[3]

((2-(2-Bromo-5-methylphenyl)-1,1,1,3,3,3-hexafluoropropan-2-yl)oxy)trimethylsilane (S2c)

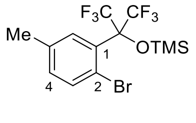

Following the general procedure **B**, the reaction of pentafluorophenyl ester **S1c** (1.72 g, 4.50 mmol, 1.00 eq.), trimethyl(trifluoromethyl)silane (4.00 mL, 27.0 mmol, 6.00 eq.) and tetrabutylammonium fluoride (1.00 molL^{-1} in THF, 1.58 mL, 1.58 mmol, 0.35 eq.) in toluene (23 mL) provided the title compound after column chromatography on silica gel (petroleum ether) as white crystals (1.44 g, 3.53 mmol, 78%). R$_f$ 0.80 (petroleum ether). The spectral data were in accordance with those previously reported in the literature.[3]

2-(2-Bromo-5-methylphenyl)-1,1,1,3,3,3-hexafluoropropan-2-ol (S3c)

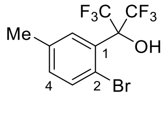

Following the general procedure **C**, the reaction of **S2c** (1.44 g, 3.53 mmol, 1.00 eq.) in THF (18 mL) and hydrochloric acid (12.0 molL^{-1}, 18.0 mL) provided the title compound after column chromatography on silica gel (petroleum ether/EtOAc 5:1) as white crystals (1.04 g, 3.09 mmol, 87%, m.p. = 51 – 52 °C); R$_f$ 0.30 (petroleum ether/EtOAc 5:1); IR $\tilde{\nu}_{max}$ (neat): 3497m, 2928w, 1912w, 1753w, 1478m, 1379w, 1203s, 1118s, 1027m, 945s, 816m, 730m, 695w, 667w; ^{1}H NMR (500 MHz, CDCl$_3$) δ 7.56 (d, $^{3}J_{H\text{-}H}$ = 8.2 Hz, 1H, C3H), 7.50 (s, 1H, C6H), 7.13 (d, $^{3}J_{H\text{-}H}$ = 8.2 Hz, 1H, C4H), 5.43 (br. s, 1H, OH), 2.36 (s, 3H, C5CH_3); ^{13}C{^{1}H} NMR (151 MHz, CDCl$_3$) δ 138.1 (*C*5), 136.4 (*C*3), 132.5 (*C*6), 131.1 (*C*4), 127.2 (*C*1), 122.7 (q, $^{1}J_{C\text{-}F}$ = 291.6 Hz, 2C, C(*C*F$_3$)$_2$), 117.0 (C2), 80.1 (hept, $^{2}J_{C\text{-}F}$ = 29.8 Hz, *C*(CF$_3$)$_2$), 21.2 (C5*C*H$_3$); ^{19}F{^{1}H} NMR (470 MHz, CDCl$_3$) δ −73.5 (6F, C(CF_3)$_2$); HRMS (ESI^{-}): *m/z* calcd. 334.9512 for ([M−H]$^{-}$), found 334.9518.

[*TBPY*-5-11']-5,5'-Dimethyl-1-hydro-3,3,3',3'-tetrakis(trifluoromethyl)-3*H*,3'*H*-1λ^5,1'-spirobi[benzo[*c*][2,1]oxaphosphole] (S4c)

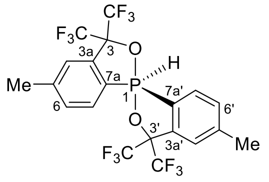

Following the general procedure **D**, the reaction of alcohol **S3c** (725 mg, 2.15 mmol, 1.00 eq.), NaH (56.8 mg, 1.99 mmol, 1.10 eq.), ^{t}BuLi (1.70 molL^{-1} in pentane, 2.80 mL, 4.73 mmol, 2.20 eq.) and PCl_3 (94.0 μL, 0.90 mmol, 0.50 eq.) in Et_2O (22 mL) provided the title compound after column chromatography on silica gel (petroleum ether) as white crystals (228 mg, 0.42 mmol, 19%, m.p. = 143 – 144 °C); R$_f$ 0.60 (petroleum ether); IR $\tilde{\nu}_{max}$ (neat): 2930w, 2362m, 1607m, 1455w, 1278m, 1208s, 1175s, 1109m, 1082m, 969m, 907m, 828w, 735m, 652w; ^{1}H NMR (500 MHz, $CDCl_3$) δ 8.29 (d, $^1J_{H-P}$ = 725.1 Hz, 1H, P*H*), 8.13 (dd, *J* = 11.6, 8.1 Hz, 2H, C7*H* and C7'*H*), 7.56 (s, 2H, C4*H* and C4'*H*), 7.52 – 7.47 (m, 2H, C6*H* and C6'*H*), 2.51 (s, 6H, C5CH_3 and C5'CH_3); ^{13}C{^{1}H} NMR (151 MHz, $CDCl_3$) δ 145.7 (2C, *C*5 and *C*5'), 137.7 (d, $^2J_{C-P}$ = 21.4 Hz, 2C, *C*3a and *C*3a'), 136.4 (d, $^2J_{C-P}$ = 11.0 Hz, 2C, *C*7 and *C*7'), 133.0 (d, $^3J_{C-P}$ = 14.5 Hz, 2C, *C*4 and *C*4'), 125.8 (d, $^3J_{C-P}$ = 16.4 Hz, 2C, *C*6 and *C*6'), 123.5 (d, $^1J_{C-P}$ = 160.8 Hz, 2C, *C*7a and *C*7a'), 122.8 (q, $^1J_{C-F}$ = 287.8 Hz, 2C, C3*C*F_3 and C3'*C*F_3), 122.6 (q, $^1J_{C-F}$ = 288.3 Hz, 2C, C3*C*F_3 and C3'*C*F_3), 81.9 (hept, $^2J_{C-F}$ = 31.1 Hz, 2C, *C*3 and *C*3'), 21.8 (2C, C5*C*H_3 and C5'*C*H_3); ^{19}F{^{1}H} NMR (376 MHz, $CDCl_3$) δ −74.5 (q, $^4J_{F-F}$ = 9.0 Hz, 6F, C3CF_3 and C3'CF_3), −75.8 (qd, $^4J_{F-F}$ = 9.1, 3.1 Hz, 6F, C3CF_3 and C3'CF_3); ^{31}P{^{1}H} NMR (202 MHz, $CDCl_3$) δ −47.0; HRMS (ESI$^-$): *m/z* calcd. 543.0389 for ([M−H]$^-$), found 543.0399.

[*TBPY*-5-15]-1,1,1,3,3,3-Hexafluoro-2-(2-(1-butyl-1-hydro-5-methyl-3,3-bis(trifluoromethyl)-3*H*-1λ^5-benzo[*c*][2,1]oxaphosphol-1-yl)-5-methylphenyl)propan-2-ol (1c)

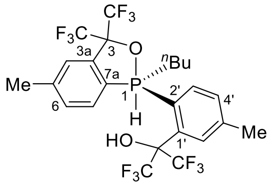

Following the general procedure **E**, the reaction of **S4c** (228 mg, 0.42 mmol, 1.00 eq.) and ^{n}BuLi (1.60 molL^{-1} in hexanes, 1.83 mL, 2.93 mmol, 7.00 eq.) in Et_2O (4.0 mL) provided the title compound after column chromatography on silica gel (petroleum ether) as white crystals (176 mg, 0.29 mmol, 91%, m.p. = 101 – 102 °C (decomposition)); R$_f$ 0.20 (petroleum ether); IR $\tilde{\nu}_{max}$ (neat): 3325s, 2943s, 2831m, 2361m, 1452m, 1418m, 1114w, 1021s, 648m; ^{1}H NMR (500 MHz, $CDCl_3$) δ 9.17 (br. s, 1H, O*H*), 7.88 (dd, *J* = 14.8, 7.7 Hz, 1H, Ar-C*H*), 7.63 (d, *J* = 7.1 Hz, 1H, Ar-C*H*), 7.59 – 7.51 (m, 2H, Ar-C*H*), 7.37 (dd, *J* = 22.7, 8.1 Hz, 1H, Ar-C*H*), 7.14 (d, *J* = 8.0 Hz, 1H, Ar-C*H*), 6.07 (dd, $^1J_{H-P}$ = 272.2 Hz, *J* = 6.8 Hz, 1H, P*H*), 3.19 – 2.95 (m, 1H, ^{n}*Bu*), 2.65 – 2.56 (m, 1H, ^{n}*Bu*), 2.56 (s, 3H, C5CH_3 or C5'CH_3), 2.38 (s, 3H, C5CH_3 or C5'CH_3), 1.57 – 1.47 (m, 1H, ^{n}Bu-C*H*), 1.40 (hd, *J* = 7.2, 1.4 Hz, 2H, ^{n}Bu-C*H*), 1.36 – 1.24 (m, 1H, ^{n}Bu-C*H*), 0.88 (t, $^3J_{H-H}$ = 7.3 Hz, 3H, ^{n}Bu-CH_3); ^{19}F{^{1}H} NMR (376 MHz, $CDCl_3$) δ −72.3 (s, 3F, CF_3), −75.7 (s, 3F, CF_3), −76.2 (s, 3F, CF_3), −76.4 (s, 3F, CF_3); Due to long NMR measurements, dehydrogenation side-products were formed. ^{31}P{^{1}H} NMR (202 MHz, $CDCl_3$) δ −4.7

(dehydrogenation side-product, (*cis*)-**2c**), −20.0 (dehydrogenation side-product, (*trans*)-**2c**), −36.1 (**1c**); HRMS (ESI^+): *m/z* calcd. 603.1317 for ($[M+H]^+$), found 603.1310.

2,3,4,5,6-Pentafluorophenyl 2-bromo-5-methoxybenzoate (S1d)

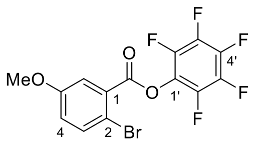

Following the general procedure **A**, the reaction of 2-bromo-5-methoxybenzoic acid (1.85 g, 8.00 mmol, 1.00 eq.), pentafluorophenol (1.65 g, 8.96 mmol, 1.12 eq.) and *N,N'*-dicyclohexylcarbodiimide (1.87 g, 9.04 mmol, 1.13 eq.) in THF (30 mL) provided the title compound after column chromatography on silica gel (pentane/CH_2Cl_2 4:1) as white solid (3.04 g, 7.65 mmol, 96%, m.p. = 58 – 59 °C); R_f 0.70 (pentane/CH_2Cl_2 4:1); IR $\tilde{\nu}_{max}$ (neat): 3013w, 2361w, 1773s, 1596w, 1518s, 1476m, 1407w, 1282m, 1205s, 1075m, 1010s, 898w, 821w; ^{1}H NMR (500 MHz, $CDCl_3$) δ 7.65 (d, $^3J_{H\text{-}H}$ = 8.8 Hz, 1H, C3*H*), 7.59 (d, $^4J_{H\text{-}H}$ = 3.1 Hz, 1H, C6*H*), 7.03 (dd, $^3J_{H\text{-}H}$ = 8.9, $^4J_{H\text{-}H}$ = 3.1 Hz, 1H, C4*H*), 3.87 (s, 3H, OCH_3); ^{13}C{^{1}H} NMR (126 MHz, $CDCl_3$) δ 161.5 (CO_2R), 158.9 (*C*5), 142.4 (C6F5-*C*), 140.9 (C6F5-*C*), 140.4 (C6F5-*C*), 139.1 (C6F5-*C*), 137.1 (C6F5-*C*), 136.0 (*C*3), 129.1 (*C*1), 125.1 (C6F5-*C*), 120.9 (*C*4), 117.7 (*C*6), 113.6 (*C*2), 56.0 (OCH_3); ^{19}F{^{1}H} NMR (376 MHz, $CDCl_3$) δ −151.8 – −152.0 (m, 2F, C2'*F* and C6'*F*), −157.4 (t, *J* = 21.7 Hz, 1F, C4'*F*), −162.0 – −162.2 (m, 2F, C3'*F* and C5'*F*); HRMS (ESI^+): *m/z* calcd. 396.9493 for ($[M+H]^+$), found 396.9493.

((2-(2-Bromo-5-methoxyphenyl)-1,1,1,3,3,3-hexafluoropropan-2-yl)oxy)trimethylsilane (S2d)

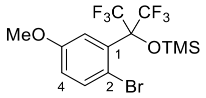

Following the general procedure **B**, the reaction of pentafluorophenyl ester (2.94 g, 7.40 mmol, 1.00 eq.), trimethyl(trifluoromethyl)silane (6.56 mL, 44.4 mmol, 6.00 eq.) and tetrabutylammonium fluoride (1.00 $molL^{-1}$ in THF, 2.59 mL, 2.59 mmol, 0.35 eq.) in toluene (37 mL) provided the title compound after column chromatography on silica gel (pentane) as a colourless liquid (1.92 g, 4.51 mmol, 61%); R_f 0.60 (pentane); IR $\tilde{\nu}_{max}$ (neat): 2985w, 2908w, 2842w, 2361w, 1772w, 1602w, 1469m, 1404w, 1217s, 1193s, 1026w, 977m, 879m, 670w, 632m; ^{1}H NMR (500 MHz, $CDCl_3$) δ 7.61 (d, $^3J_{H\text{-}H}$ = 8.8 Hz, 1H, C3*H*), 7.22 (d, $^4J_{H\text{-}H}$ = 2.9 Hz, 1H, C6*H*), 6.81 (dd, $^3J_{H\text{-}H}$ = 8.8 Hz, $^4J_{H\text{-}H}$ = 2.9 Hz, 1H, C4*H*), 3.80 (s, 3H, OCH_3), 0.24 (s, 9H, Si(CH_3)$_3$); ^{13}C{^{1}H} NMR (126 MHz, $CDCl_3$) δ 158.3 (*C*5), 137.9 (*C*3), 131.1 (*C*1), 122.9 (q, $^1J_{C\text{-}F}$ = 291.8 Hz, 2C, C(*C*F_3)$_2$), 117.0 (*C*6), 116.0 (*C*4), 112.6 (*C*2), 81.7 (hept, $^2J_{C\text{-}F}$ = 30.0 Hz, *C*(CF_3)$_2$), 55.6 (OCH_3), 1.7 (Si(CH_3)$_3$); ^{19}F{^{1}H} NMR (376 MHz, $CDCl_3$) δ −70.4 (6F, C(C*F*$_3$)$_2$); HRMS (ESI^-): *m/z* calcd. 350.9461 for ($[M–TMS]^-$), found 350.9467.

2-(2-Bromo-5-methoxyphenyl)-1,1,1,3,3,3-hexafluoropropan-2-ol (S3d)

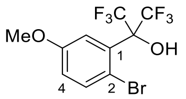

Following the general procedure **C**, the reaction of trimethylsilane (1.82 g, 4.28 mmol, 1.00 eq.) in THF (21 mL) and hydrochloric acid (12.0 $molL^{-1}$, 10.5 mL) provided the title compound after column chromatography on silica gel (pentane/CH_2Cl_2 4:1) as white solid (1.36 g, 3.85 mmol, 90%, m.p. = 53 – 54 °C); R_f 0.40 (pentane/CH_2Cl_2

4:1); IR $\tilde{\nu}_{max}$ (neat): 3484m, 3014w, 2361m, 1601m, 1479m, 1374w, 1226s, 1128m, 965m, 878w, 815w, 673w, 632m; ^{1}H NMR (500 MHz, $CDCl_3$) δ 7.58 (d, $^{3}J_{H\text{-}H}$ = 8.9 Hz, 1H, C3*H*), 7.25 (m, 1H, C6*H* overlapping with $CDCl_3$), 6.88 (dd, $^{3}J_{H\text{-}H}$ = 8.9, $^{4}J_{H\text{-}H}$ = 3.0 Hz, 1H, C4*H*), 5.45 (s, 1H, O*H*), 3.82 (s, 3H, OCH_3); ^{13}C{^{1}H} NMR (126 MHz, $CDCl_3$) δ 158.9 (*C*5), 137.2 (*C*3), 128.2 (*C*1), 122.6 (q, $^{1}J_{C\text{-}F}$ = 288.5 Hz, 2C, C(*C*F_3)$_2$), 117.2 (hept, $^{4}J_{C\text{-}F}$ = 3.0 Hz, *C*6), 117.0 (*C*4), 110.3 (*C*2), 80.1 (hept, $^{2}J_{C\text{-}F}$ = 28.5 Hz, *C*(CF_3)$_2$), 55.8 (O*C*H_3); ^{19}F{^{1}H} NMR (376 MHz, $CDCl_3$) δ −73.7 (6F, C(C*F*$_3$)$_2$); HRMS (ESI$^+$): *m/z* calcd. 352.9606 for ([M+H]$^+$), found 352.9606.

[*TBPY*-5-11']-5,5'-Dimethoxy-1-hydro-3,3,3',3'-tetrakis(trifluoromethyl)-3*H*,3'*H*-1λ^5,1'-spirobi[benzo[*c*][2,1]oxaphosphole] (S4d)

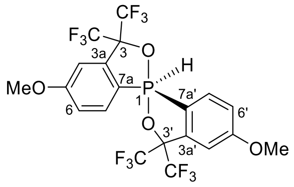

Following the general procedure **D**, the reaction of alcohol (1.20 g, 3.40 mmol, 1.00 eq.), NaH (150 mg, 3.74 mmol, 1.10 eq.), ^{t}BuLi (1.70 molL^{-1} in pentane, 4.40 mL, 7.48 mmol, 2.20 eq.) and PCl_3 (148 µL, 1.70 mmol, 0.50 eq.) in Et_2O (17 mL) provided the title compound after column chromatography on silica gel (pentane) as white solid (153 mg, 0.27 mmol, 8%, m.p. = 153 – 154 °C); R$_f$ 0.30 (pentane); IR $\tilde{\nu}_{max}$ (neat): 3464w, 3218w, 3021w, 2925w, 2361m, 1602m, 1245m, 1192m, 1079s, 991m, 906s, 822m, 730s; ^{1}H NMR (500 MHz, $CDCl_3$) δ 8.25 (d, $^{1}J_{H\text{-}P}$ = 724.3 Hz, 1H, P*H*), 8.14 (dd, $^{3}J_{H\text{-}P}$ = 11.1 Hz, $^{3}J_{H\text{-}H}$ = 8.7 Hz, 2H, C7*H* and C7'*H*), 7.24 – 7.19 (m, 2H, C4*H* and C4'*H*), 7.19 (ddd, $^{3}J_{H\text{-}H}$ = 8.7 Hz, *J* = 4.3, 2.4 Hz, 2H, C6*H* and C6'*H*), 3.91 (s, 6H, C5OCH_3 and C5'OCH_3); ^{13}C{^{1}H} NMR (126 MHz, $CDCl_3$) δ 164.7 (d, $^{4}J_{C\text{-}P}$ = 3.5 Hz, 2C, *C*5 and *C*5'), 139.7 (d, $^{2}J_{C\text{-}P}$ = 23.6 Hz, 2C, *C*3a and *C*3a'), 137.9 (d, $^{2}J_{C\text{-}P}$ = 12.1 Hz, 2C, *C*7 and *C*7'), 123.9 (q, $^{1}J_{C\text{-}F}$ = 287.6 Hz, 2C, C3(*C*F_3)$_2$ and C3'(*C*F_3)$_2$), 121.9 (q, $^{1}J_{C\text{-}F}$ = 285.8 Hz, 2C, C3(*C*F_3)$_2$ and C3'(*C*F_3)$_2$), 117.9 (d, $^{3}J_{C\text{-}P}$ = 15.7 Hz, 2C, *C*6 and *C*6'), 117.2 (d, $^{1}J_{C\text{-}P}$ = 166.8 Hz, 2C, *C*7a and *C*7a'), 110.8 (d, $^{3}J_{C\text{-}P}$ = 17.5 Hz, 2C, *C*4 and *C*4'), 82.0 – 80.4 (m, 2C, *C*3 and *C*3'), 56.0 (2C, C5O*C*H_3 and C5'O*C*H_3); ^{19}F{^{1}H} NMR (376 MHz, $CDCl_3$) δ −74.6 (q, *J* = 9.1 Hz, 6F, C3C*F*$_3$ and C3'C*F*$_3$), −75.9 (qd, *J* = 9.1, 3.0 Hz, 6F, C3C*F*$_3$ and C3'C*F*$_3$); ^{31}P{^{1}H} NMR (202 MHz, $CDCl_3$) δ −48.3; HRMS (ESI$^+$): *m/z* calcd. 577.0433 for ([M+H]$^+$), found 577.0422.

[*TBPY*-5-15]-1,1,1,3,3,3-Hexafluoro-2-(2-(1-butyl-1-hydro-5-methoxy-3,3-bis(trifluoromethyl)-3*H*-1λ^5-benzo[*c*][2,1]oxaphosphol-1-yl)-5-methoxyphenyl)propan-2-ol (1d)

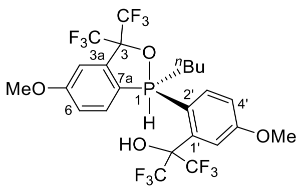

Following the general procedure **E**, the reaction of **S4d** (120 mg, 0.21 mmol, 1.00 eq.) and ^{n}BuLi (1.60 molL^{-1} in hexanes, 0.91mL, 1.46 mmol, 7.00 eq.) in Et_2O (2.1 mL) provided the title compound after column chromatography on silica gel (pentane/CH_2Cl_2 4:1) as colourless oil (98.0 mg, 0.15 mmol, 74%); R$_f$ 0.45 (pentane/CH_2Cl_2 4:1); IR $\tilde{\nu}_{max}$ (neat): 3054m, 2924w, 2361s, 2212w, 1603m, 1212m, 1148m, 965m, 913w, 773s, 608w; ^{1}H NMR (400 MHz, $CDCl_3$) δ 9.26 (br. s, 1H, O*H*), 7.85 (dd, *J* = 14.4, 9.0 Hz, 1H, Ar-C*H*), 7.47 – 7.31

(m, 2H, Ar-CH), 7.25 – 7.14 (m, 2H, Ar-CH), 6.83 (d, J = 8.7 Hz, 1H, Ar-CH), 6.02 (dd, $^1J_{H\text{-}P}$ = 272.5 Hz, J = 6.0 Hz, 1H, PH), 3.92 (s, 3H, C5OCH_3 or C5'OCH_3), 3.80 (s, 3H, C5OCH_3 or C5'OCH_3), 3.07 – 2.92 (m, 1H, Alk-CH), 2.60 – 2.46 (m, 1H, Alk-CH), 1.51 – 1.43 (m, 1H, Alk-CH), 1.32 – 1.19 (m, 3H, Alk-CH), 0.86 (t, $^3J_{H\text{-}H}$ = 7.2 Hz, 3H, P(CH$_2$)$_3$CH_3); ^{19}F{^{1}H} NMR (376 MHz, CDCl$_3$) δ −72.4 (3F, CF3), −75.8 (q, $^4J_{F\text{-}F}$ = 9.1 Hz, 3F, CF3), −76.2 (q, $^4J_{F\text{-}F}$ = 8.2 Hz, 3F, CF3), −76.5 (m, 3F, CF3); ^{31}P{^{1}H} NMR (162 MHz, CDCl$_3$) δ −37.4; HRMS (ESI$^+$): *m/z* calcd. 635.1215 for ([M+H]$^+$), found 635.1222.

2,3,4,5,6-Pentafluorophenyl 2-bromo-5-methoxy-4-methylbenzoate (S1e)

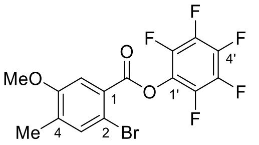

Following the general procedure **A**, the reaction of 2-bromobenzoic acid (1.96 g, 8.00 mmol, 1.00 eq.), pentafluorophenol (1.65 g, 8.96 mmol, 1.12 eq.) and *N,N'*-dicyclohexylcarbodiimide (1.87 g, 9.04 mmol, 1.13 eq.) in THF (30 mL) provided the title compound after column chromatography on silica gel (petroleum ether) as white crystals (3.19 g, 7.76 mmol, 97%, m.p. = 106 – 107 °C); R$_f$ 0.30 (petroleum ether); IR $\tilde{\nu}_{max}$ (neat): 3699m, 2970s, 2866m, 2361s, 2340m, 1520m, 1214w, 1054s, 1012s, 745w, 684w; ^{1}H NMR (500 MHz, CDCl$_3$) δ 7.54 (s, 2H, C3H and C6H), 3.93 (s, 3H, OCH_3), 2.30 (s, 3H, C4CH_3); ^{13}C{^{1}H} NMR (126 MHz, CDCl$_3$) δ 161.3 (CO$_2$R), 156.8 (C5), 142.3 (C$_6$F$_5$-C), 140.6 (C$_6$F$_5$-C), 140.3 (C$_6$F$_5$-C), 138.9 (C$_6$F$_5$-C), 138.6 (C$_6$F$_5$-C), 136.9 (C$_6$F$_5$-C), 136.6 (C3), 135.3 (C4), 125.6 (C1), 114.1 (C6), 113.3 (C2), 55.7 (C5OCH$_3$), 16.2 (C4CH$_3$); ^{19}F{^{1}H} NMR (376 MHz, CDCl$_3$) δ −151.9 (dq, J = 15.6, 5.3 Hz, 2F, C2'F and C6'F), −157.7 (q, J = 21.6 Hz, 1F, C4'F), −162.0 – −162.4 (m, 2F, C3'F and C5'F); HRMS (ESI$^-$): *m/z* calcd. 242.9657 for ([M−C$_6$F$_5$]$^-$), found 242.9664.

((2-(2-Bromo-5-methoxy-4-methylphenyl)-1,1,1,3,3,3-hexafluoropropan-2-yl)oxy)trimethylsilane (S2e)

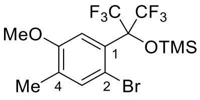

Following the general procedure **B**, the reaction of pentafluorophenyl ester **S1e** (3.18 g, 7.70 mmol, 1.00 eq.), trimethyl(trifluoromethyl)silane (6.90 mL, 46.4 mmol, 6.00 eq.) and tetrabutylammonium fluoride (1.00 molL^{-1} in THF, 2.71 mL, 2.71 mmol, 0.35 eq.) in toluene (39 mL) provided the title compound after column chromatography on silica gel (petroleum ether) as colourless oil (2.64 g, 6.01 mmol, 78%); R$_f$ 0.80 (petroleum ether); IR $\tilde{\nu}_{max}$ (neat): 2967w, 2359m, 2253w, 1465w, 1251m, 1197m, 1052w, 903s, 723s, 649m; ^{1}H NMR (500 MHz, CDCl$_3$) δ 7.46 (s, 1H, C3H), 7.06 (s, 1H, C6H), 3.81 (s, 3H, OCH_3), 2.18 (s, 3H, C4CH_3), 0.24 (s, 9H, Si(CH_3)$_3$); ^{13}C{^{1}H} NMR (151 MHz, CDCl$_3$) δ 156.4 (C5), 138.5 (C3), 130.5 (C6), 127.9 (C4), 122.9 (q, $^1J_{C\text{-}F}$ = 292.5 Hz, 2C, C(CF$_3$)$_2$), 112.4 (C2), 111.5 (C1), 81.7 (hept, $^2J_{C\text{-}F}$ = 29.9 Hz, C(CF$_3$)$_2$), 55.4 (C5OCH$_3$), 15.5 (C4CH$_3$), 1.6 (Si(CH$_3$)$_3$); ^{19}F{^{1}H} NMR (470 MHz, CDCl$_3$) δ −70.5(6F, C(CF_3)$_2$); HRMS (ESI$^-$): *m/z* calcd. 364.9617 for ([M−TMS]$^-$), found 364.9625.

2-(2-Bromo-5-methoxy-4-methylphenyl)-1,1,1,3,3,3-hexafluoropropan-2-ol (S3e)

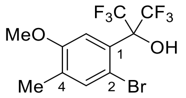

Following the general procedure **C**, the reaction of **S2e** (3.08 g, 7.00 mmol, 1.00 eq.) in THF (35 mL) and hydrochloric acid (12.0 molL^{-1}, 35.0 mL) provided the title compound after column chromatography on silica gel (petroleum ether/EtOAc 5:1) as white crystals (1.93 g, 5.26 mmol, 75%, m.p. = 40 – 41 °C); R$_f$ 0.30 (petroleum ether/EtOAc 5:1); IR $\tilde{\nu}_{max}$ (neat): 3483m, 2960m, 2848w, 2359m, 1607w, 1508m, 1465m, 1354m, 1208s, 1123s, 1046m, 979m, 862m, 782m, 702w.635w; ^{1}H NMR (500 MHz, CDCl$_3$) δ 7.41 (s, 1H, C3*H*), 7.09 (s, 1H, C6*H*), 5.52 (br. s, 1H, O*H*), 3.82 (s, 3H, C5OC*H*$_3$), 2.21 (s, 3H, C4C*H*$_3$); ^{13}C{^{1}H} NMR (151 MHz, CDCl$_3$) δ 157.0 (*C*5), 137.6 (*C*3), 131.5 (*C*4), 125.0 (*C*1), 122.7 (q, $^{1}J_{C\text{-}F}$ = 289.3 Hz, 2C, C(*C*F$_3$)$_2$), 111.6 (*C*6), 110.1 (*C*2), 80.0 (hept, $^{2}J_{C\text{-}F}$ = 29.8 Hz, *C*(CF$_3$)$_2$), 55.5 (O*C*H$_3$), 15.7 (C4*C*H$_3$); ^{19}F{^{1}H} NMR (470 MHz, CDCl$_3$) δ −73.7 (6F, C(C*F*$_3$)$_2$); HRMS (ESI$^-$): *m/z* calcd. 364.9617 for ([M−H]$^-$), found 364.9625.

[*TBPY*-5-11']-5,5'-Dimethoxy-6,6'-dimethyl-1-hydro-3,3,3',3'-tetrakis(trifluoromethyl)-3*H*,3'*H*-1λ^5,1'-spirobi[ben-zo[*c*][2,1]oxaphosphole] (S4e)

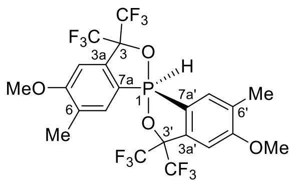

Following the general procedure **D**, the reaction of alcohol **S3e** (1.75 g, 4.76 mmol, 1.00 eq.), NaH (126 mg, 5.24 mmol, 1.10 eq.), ^{t}BuLi (1.70 molL^{-1} in pentane, 6.20 mL, 10.5 mmol, 2.20 eq.) and PCl$_3$ (208 µL, 2.38 mmol, 0.50 eq.) in Et$_2$O (24 mL) provided the title compound after column chromatography on silica gel (petroleum ether) as white crystals (189 mg, 0.31 mmol, 13%, m.p. = 199 – 200 °C); R$_f$ 0.60 (petroleum ether); IR $\tilde{\nu}_{max}$ (neat): 2926m, 2858w, 2359w, 1600m, 1461m, 1277m, 1254s, 1191s, 1116m, 1068s, 968m, 902m, 840m, 751m, 647w; ^{1}H NMR (500 MHz, CDCl$_3$) δ 8.25 (d, $^{1}J_{H\text{-}P}$ = 722.6 Hz, 1H, P*H*), 7.96 (d, $J_{H\text{-}H}$ = 11.1 Hz, 2H, C7*H* and C7'*H*), 7.10 (s, 2H, C4*H* and C4'*H*), 3.94 (s, 6H, C5OC*H*$_3$ and C5'OC*H*$_3$), 2.28 (s, 6H, C6C*H*$_3$ and C6'C*H*$_3$); ^{13}C{^{1}H} NMR (126 MHz, CDCl$_3$) δ 162.6 (d, $^{4}J_{C\text{-}P}$ = 3.6 Hz, 2C, *C*5 and *C*5'), 137.3 (d, $^{2}J_{C\text{-}P}$ = 12.2 Hz, 2C, *C*7 and *C*7'), 137.3 (d, $^{2}J_{C\text{-}P}$ = 22.7 Hz, 2C, *C*3a and *C*3a'), 131.2 (d, $^{3}J_{C\text{-}P}$ = 15.1 Hz, 2C, *C*6 and *C*6'), 122.7 (q, $^{1}J_{C\text{-}F}$ = 287.4 Hz, 2C, C3*C*F$_3$ and C3'*C*F$_3$), 122.5 (q, $^{1}J_{C\text{-}F}$ = 286.9 Hz, 2C, C3*C*F$_3$ and C3'*C*F$_3$), 116.6 (d, $^{1}J_{C\text{-}P}$ = 165.0 Hz, 2C, *C*7a and *C*7a'), 105.8 (d, $^{3}J_{C\text{-}P}$ = 17.9 Hz, 2C, *C*4 and *C*4'), 81.8 (hept, $^{2}J_{C\text{-}F}$ = 30.8 Hz, 2C, *C*3 and *C*3'), 55.7 (2C, O*C*H$_3$), 16.6 (2C, C6*C*H$_3$); ^{19}F{^{1}H} NMR (470 MHz, CDCl$_3$) δ −74.6 (q, $^{4}J_{F\text{-}F}$ = 9.1 Hz, 6F, C3C*F*$_3$ and C3'C*F*$_3$), −76.0 (qd, $^{4}J_{F\text{-}F}$ = 9.1, 2.9 Hz, 6F, C3C*F*$_3$ and C3'C*F*$_3$); ^{31}P{^{1}H} NMR (202 MHz, CDCl$_3$) δ −47.1; HRMS (ESI$^-$): *m/z* calcd. 603.0600 for ([M−H]$^-$), found 603.0612.

[*TBPY*-5-15]-1,1,1,3,3,3-Hexafluoro-2-(2-(1-butyl-1-hydro-5-methoxy-6-methyl-3,3-bis(trifluoromethyl)-3*H*-1λ^5-benzo[*c*][2,1]oxaphosphol-1-yl)-5-methoxy-4-methylphenyl)propan-2-ol (1e)

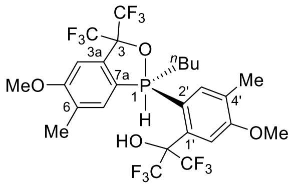

Following the general procedure **E**, the reaction of **S4e** (185 mg, 0.31 mmol, 1.00 eq.) and ^{n}BuLi (1.60 molL^{-1} in hexanes, 1.34 mL, 2.15 mmol, 7.00 eq.) in Et_2O (3.0 mL) provided the title compound after column chromatography on silica gel (petroleum ether/EtOAc 20:1) as white crystals (156 mg, 0.24 mmol, 77%, m.p. = 121 – 122 °C (decomposition)); R_f 0.20 (petroleum ether/EtOAc 20:1); IR $\tilde{\nu}_{max}$ (neat):2966m, 2866m, 2362m, 1769w, 1604w, 1516w, 1465m, 1254s, 2291s, 2252s, 1067s, 964m, 911m, 714m, 679w; ^{1}H NMR (500 MHz, $CDCl_3$) δ 9.41 (br. s, 1H, O*H*), 7.65 (d, $J_{H\text{-}H}$ = 14.5 Hz, 1H, Ar-C*H*), 7.23 – 7.09 (m, 3H, Ar-C*H*), 5.95 (dd, $^1J_{H\text{-}P}$ = 271.9 Hz, *J* = 7.0 Hz, 1H, P*H*), 3.93 (s, 3H, C5CH_3 or C5'CH_{33}), 3.84 (s, 3H, C5CH_3 or C5'CH_3), 2.98 (q, *J* = 15.0 Hz, 1H, ^{n}Bu-C*H*), 2.62 – 2.44 (m, 1H, ^{n}Bu-C*H*), 2.40 (s, 3H, C6CH_3 or C4'CH_3), 2.10 (s, 3H, C6CH_3 or C4'CH_3), 1.52 – 1.45 (m, 1H, ^{n}Bu-C*H*), 1.36 (p, *J* = 7.4 Hz, 2H, ^{n}Bu-C*H*), 1.30 – 1.25 (m, 1H, ^{n}Bu-C*H*), 0.86 (t, $^3J_{H\text{-}H}$ = 7.2 Hz, 3H, ^{n}Bu-CH_3); ^{19}F{^{1}H} NMR (470 MHz, $CDCl_3$) δ −72.5 (s, 3F, CF_3), −75.7 (q, $^4J_{F\text{-}F}$ = 9.1 Hz, 3F, CF_3), −76.1 (q, $^4J_{F\text{-}F}$ = 8.7 Hz, 3F, CF_3), −76.4 (dq, $^4J_{F\text{-}F}$ = 10.8, 6.7 Hz, 3F, CF_3); ^{31}P{^{1}H} NMR (202 MHz, $CDCl_3$) δ −37.4; HRMS (ESI$^+$): *m/z* calcd. 663.1528 for ([M+H]$^+$), found 663.1535.

3-Bromo-2-naphthoic acid (S5f)

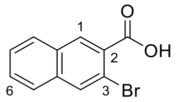

The title compound was prepared according to the modified literature procedure.[6] In a 1.0 L round-bottom flask iodobenzene diacetate (9.86 g, 30.0 mmol, 2.00 eq.) and I_2 (7.61 g, 30.0 mmol, 2.00 eq.) were dissolved in DMF (150 mL) under atmospheric air. The reaction was stirred at r.t. for 1 h and 2-naphthoic acid (2.64 g, 15.0 mmol, 1.00 eq.), $Pd(OAc)_2$ (168 mg, 0.75 mmol, 5.00 mol%) and tetramethylammonium bromide (3.47 g, 22.5 mmol, 1.50 eq.) were added under atmospheric air. A reflux condenser with deflated balloon was attached to the flask, and the reaction mixture was stirred at 100 °C for 24 h. The reaction mixture was cooled to room temperature and 10% aq. Na_2CO_3 (160 mL) was added. The mixture was washed with Et_2O (2 x 200 mL), and the aqueous layer was acidified with HCl (12.0 molL^{-1}, 38 mL). The aqueous layer was extracted with EtOAc (4 x 200 mL), and the combined organic phases were washed with sat. aq. $Na_2S_2O_3$ (3 x 100 mL), dried over Na_2SO_4 and concentrated under vacuum. The residue was purified by column chromatography on silica gel (hexane/Et_2O 8:1) to afford 3-bromo-2-naphthoic acid as a white solid (1.33 g, 5.30 mmol, 35%). The spectral data were in accordance with those previously reported in the literature.[6]

2,3,4,5,6-Pentafluorophenyl 3-bromo-2-naphthoate (S1f)

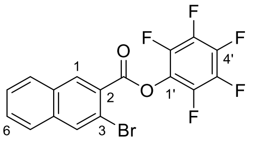

Following the general procedure **A**, the reaction of acid **S5f** (1.38 g, 5.50 mmol, 1.00 eq.), pentafluorophenol (1.13 g, 6.16 mmol, 1.12 eq.) and *N,N'*-dicyclohexylcarbodiimide (1.28 g, 6.21 mmol, 1.13 eq.) in THF (28 mL) provided the title compound after column chromatography on silica gel (petroleum ether) as white crystals (2.00 g, 4.80 mmol, 87%, m.p. = 92 – 93 °C); R_f 0.30 (petroleum ether); IR $\tilde{\nu}_{max}$ (neat): 3708w, 2970m, 2361m, 1768s, 1625w, 1517s, 1449m, 1253m, 1190m, 1053s, 994s, 891m, 759m, 651w; ^{1}H NMR (500 MHz, $CDCl_3$) δ 8.68 (s, 1H, C4*H*), 8.22 (s, 1H, C1*H*), 7.95 (d, $^3J_{H-H}$ = 7.7 Hz, 1H, C6*H*), 7.81 (d, $^3J_{H-H}$ = 7.8 Hz, 1H, C9*H*), 7.71 – 7.65 (m, 1H, C8*H*), 7.65 – 7.58 (m, 1H, C7*H*); ^{13}C{^{1}H} NMR (126 MHz, $CDCl_3$) δ 161.4 (*C*O_2R), 142.4 (C_6F_5-*C*), 140.7 (C_6F_5-*C*), 140.4 (C_6F_5-*C*), 139.0 (C_6F_5-*C*), 138.5 (C_6F_5-*C*), 136.9 (C_6F_5-*C*), 135.9 (*C*5), 134.4 (*C*1), 133.8 (*C*4), 130.9 (*C*7), 130.1 (*C*10), 129.1 (*C*8), 127.6 (*C*9), 126.8 (*C*6), 125.1 (*C*2), 117.1 (*C*3); ^{19}F{^{1}H} NMR (376 MHz, $CDCl_3$) δ −151.7 – −152.0 (m, 2F, C2'*F* and C6'*F*), −157.4 (t, *J* = 21.8 Hz, 1F, C4'*F*), −161.8 – −162.1 (m, 2F, C3'*F* and C5'*F*); HRMS (ESI$^+$): *m/z* calcd. 438.9364 for ([M+Na]$^+$), found 438.9362.

((2-(3-Bromonaphthalen-2-yl)-1,1,1,3,3,3-hexafluoropropan-2-yl)oxy)trimethylsilane (S2f)

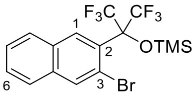

Following the general procedure **B**, the reaction of pentafluorophenyl ester **S1f** (1.90 g, 4.56 mmol, 1.00 eq.), trimethyl(trifluoromethyl)silane (4.10 mL, 27.4 mmol, 6.00 eq.) and tetrabutylammonium fluoride (1.00 molL^{-1} in THF) (1.60 mL, 1.60 mmol, 0.35 eq.) in toluene (23 mL) provided the title compound after column chromatography on silica gel (petroleum ether) as yellow oil (1.48 g, 3.32 mmol, 73%); R_f 0.80 (petroleum ether); IR $\tilde{\nu}_{max}$ (neat): 2961w, 2225w, 1583w, 1490w, 1430w, 1280m, 1254m, 1213s, 1185s, 1002m, 963m, 847s, 746m, 727m, 635w; ^{1}H NMR (500 MHz, $CDCl_3$) δ 8.27 (s, 1H, C4*H*), 8.18 (s, 1H, C1*H*), 7.85 (d, $^3J_{H-H}$ = 8.1 Hz, 1H, C9*H*), 7.75 (d, $^3J_{H-H}$ = 9.4 Hz, 1H, C6*H*), 7.63 – 7.52 (m, 2H, C7*H and* C8*H*), 0.25 (s, 9H, Si(CH_3)$_3$); ^{13}C{^{1}H} NMR (151 MHz, $CDCl_3$) δ 135.9 (*C*4), 134.0 (*C*2), 131.0 (*C*10), 130.7 (*C*1), 128.9 (*C*9), 128.8 (*C*6), 127.3 (*C*7), 127.1 (*C*5), 126.4 (*C*8), 123.0 (q, $^1J_{C-F}$ = 292.8 Hz, 2C, C(*C*F_3)$_2$), 117.9 (*C*3), 82.2 (hept, $^2J_{C-F}$ = 30.0 Hz, *C*(CF_3)$_2$), 1.6 (Si(*C*H_3)$_3$); ^{19}F{^{1}H} NMR (470 MHz, $CDCl_3$) δ −70.0 (6F, C(C*F*$_3$)$_2$); HRMS (ESI$^-$): *m/z* calcd 370.9512. for ([M−TMS]$^-$), found 370.9517.

2-(3-Bromonaphthalen-2-yl)-1,1,1,3,3,3-hexafluoropropan-2-ol (S3f)

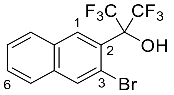

Following the general procedure **C**, the reaction of **S2f** (1.34 g, 3.00 mmol, 1.00 eq.) in THF (15 mL) and hydrochloric acid (12.0 molL^{-1}, 15.0 mL) provided the title compound after column chromatography on silica gel (petroleum ether/EtOAc 5:1) as white crystals (0.94 g, 2.52 mmol, 84%, m.p. = 83 – 84 °C); R_f 0.30 (petroleum ether/EtOAc 5:1); IR $\tilde{\nu}_{max}$ (neat): 3449m, 2971m, 2866w, 2360m, 1585m, 1491w, 1372w, 1261m, 1227s, 1200s,

1114m, 953m, 883m, 847m, 724m, 667w, 639w; ^{1}H NMR (500 MHz, $CDCl_3$) δ 8.23 (s, 2H, C1*H* and C4*H*), 7.89 (d, $^{3}J_{H\text{-}H}$ = 8.2 Hz, 1H, C9*H*), 7.78 (d, $^{3}J_{H\text{-}H}$ = 7.8 Hz, 1H, C6*H*), 7.66 – 7.55 (m, 2H, C7*H* and C8*H*), 5.48 (br. s, 1H, O*H*); ^{13}C{^{1}H} NMR (151 MHz, $CDCl_3$) δ 135.5 (*C*4), 134.1 (*C*2), 131.3 (*C*1), 129.2 (*C*9), 129.0 (*C*6), 127.9 (*C*8), 127.7 (*C*10), 126.5 (*C*7), 123.8 (*C*5), 122.8 (q, $^{1}J_{C\text{-}F}$ = 288.7 Hz, 2C, C(*C*F_3)$_2$), 115.7 (C3), 80.4 (hept, $^{2}J_{C\text{-}F}$ = 30.0 Hz, *C*(CF_3)$_2$”); ^{19}F{^{1}H} NMR (470 MHz, $CDCl_3$) δ −73.3 (6F, C(C*F*$_3$)$_2$); HRMS (ESI$^-$): *m/z* calcd. 370.9512 for ([M−H]$^-$), found 370.9517.

[*TBPY*-5-11']-1-Hydro-3,3,3',3'-tetrakis(trifluoromethyl)-3*H*,3'*H*-1λ^5,1'-spirobi[naphtho[2,3-*c*][2,1]oxaphosphole] (S4f)

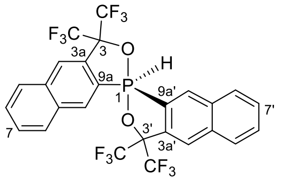

Following the general procedure **D**, the reaction of alcohol **S3f** (942 mg, 2.52 mmol, 1.00 eq.), NaH (66.7 mg, 2.78 mmol, 1.10 eq.), ^{t}BuLi (1.70 molL^{-1} in pentane, 3.30 mL, 5.55 mmol, 2.20 eq.) and PCl_3 (110 μL, 0.90 mmol, 0.50 eq.) in Et_2O (22 mL) provided the title compound after column chromatography on silica gel (petroleum ether) as white crystals (452 mg, 0.73 mmol, 58%, m.p. = 203 – 204 °C); R_f 0.60 (petroleum ether); IR $\tilde{\nu}_{max}$ (neat): 2362w, 1598w, 1305m, 1275m, 1205s, 1174s, 1109m, 970m, 895s, 826m, 751m, 697m, 611m; ^{1}H NMR (500 MHz, $CDCl_3$) δ 8.95 (d, $^{3}J_{H\text{-}P}$ = 14.4 Hz, 2H, C9*H* and C9’*H*), 8.58 (d, $^{1}J_{H\text{-}P}$ = 731.5 Hz, 1H, P*H*), 8.29 (s, 2H, C4*H* and C4’*H*), 8.08 (d, $^{3}J_{H\text{-}H}$ = 7.5 Hz, 2H, C8*H* and C8’*H*), 8.04 (d, $^{3}J_{H\text{-}H}$ = 8.3 Hz, 2H, C5*H* and C5’*H*), 7.75 (t, $^{3}J_{H\text{-}H}$ = 6.8 Hz, 2H, C7*H* and C7’*H*), 7.69 (t, $^{3}J_{H\text{-}H}$ = 7.5 Hz, 2H, C6*H* and C6’*H*); ^{13}C{^{1}H} NMR (126 MHz, $CDCl_3$) δ 140.0 (d, $^{2}J_{C\text{-}P}$ = 10.3 Hz, 2C, *C*9 and *C*9’), 135.6 (d, $^{4}J_{C\text{-}P}$ = 3.0 Hz, 2C, *C*4a and *C*4a’), 134.4 (d, $^{3}J_{C\text{-}P}$ = 16.2 Hz, 2C, *C*8a and *C*8a’), 131.2 (d, $^{2}J_{C\text{-}P}$ = 22.7 Hz, 2C, *C*3a and *C*3a’), 129.8 (2C, *C*8 and *C*8’), 129.8 (2C, *C*7 and *C*7’), 128.4 (d, $^{5}J_{C\text{-}P}$ = 1.4 Hz, 2C, *C*5 and *C*5’), 128.1 (d, *J* = 1.5 Hz, 2C, *C*6 and *C*6’), 125.2 (d, $^{3}J_{C\text{-}P}$ = 15.6 Hz, 2C, *C*4 *and C*4’), 122.9 (q, $^{1}J_{C\text{-}F}$ = 287.2 Hz, 2C, C3*C*F_3 and C3’*C*F_3), 122.8 (q, $^{1}J_{C\text{-}F}$ = 286.6 Hz, 2C, C3*C*F_3 and C3’*C*F_3), 122.1 (d, $^{1}J_{C\text{-}P}$ = 161.1 Hz, 2C, *C*9a and *C*9a’), 81.7 (hept, $^{2}J_{C\text{-}F}$ = 30.2 Hz, 2C, *C*3 and *C*3’); ^{19}F{^{1}H} NMR (470 MHz, $CDCl_3$) δ −74.4 (q, $^{4}J_{F\text{-}F}$ = 9.1 Hz, 6F, C3C*F*$_3$ and C3’C*F*$_3$), −75.9 (qd, $^{4}J_{F\text{-}F}$ = 9.1, 3.1 Hz, 6F, C3C*F*$_3$ and C3’C*F*$_3$); ^{31}P NMR (202 MHz, $CDCl_3$) δ −49.4; HRMS (ESI$^-$): *m/z* calcd. 615.0389 for ([M−H]$^-$), found 615.0401.

[*TBPY*-5-15]-1,1,1,3,3,3-Hexafluoro-2-(3-(1-butyl-1-hydro-3,3-bis(trifluoromethyl)-3*H*-1λ^5-naphtho[2,3-*c*][2,1]oxaphosphol-1-yl)-naphthalen-2-yl)propan-2-ol (1f)

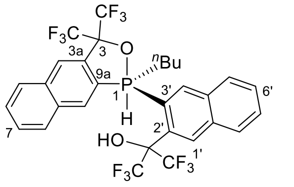

Following the general procedure **E**, the reaction of **S4f** (189 mg, 0.31 mmol, 1.00 eq.) and ^{n}BuLi (1.60 molL^{-1} in hexanes, 1.34 mL, 2.15 mmol, 7.00 eq.) in Et_2O (3.0 mL) provided the title compound after column chromatography on silica gel (petroleum ether) as white crystals (152 mg, 0.23 mmol, 74%, m.p. = 163 – 164 °C

(decomposition)); R_f 0.20 (petroleum ether); IR $\tilde{\nu}_{max}$ (neat):3320s, 2943m, 2831m, 2361m, 1452m, 1416m, 1115w, 1021s, 668m; ^{1}H NMR (500 MHz, $CDCl_3$) δ 9.34 (br. s, 1H, O*H*), 8.59 (d, *J* = 17.6 Hz, 1H, Ar-C*H*), 8.29 (d, *J* = 7.0 Hz, 1H, Ar-C*H*), 8.12 (s, 2H, Ar-C*H*), 8.03 (d, *J* = 25.3 Hz, 1H, Ar-C*H*), 7.97 – 7.92 (m, 1H, Ar-C*H*), 7.80 (d, *J* = 8.2 Hz, 1H, Ar-C*H*), 7.71 – 7.64 (m, 2H, Ar-C*H*), 7.56 (d, *J* = 8.2 Hz, 1H, Ar-C*H*), 7.47 (t, *J* = 7.5 Hz, 1H, Ar-C*H*), 7.40 (t, *J* = 7.5 Hz, 1H, Ar-C*H*), 6.40 (dd, $^1J_{H\text{-}P}$ = 277.8, 5.8 Hz, 1H, P*H*), 3.23 – 3.10 (m, 1H, ^{n}Bu-C*H*), 2.58 (tdd, *J* = 18.1, 11.0, 5.1 Hz, 1H, ^{n}Bu-C*H*), 1.61 – 1.48 (m, 1H, ^{n}Bu-C*H*), 1.40 – 1.21 (m, 3H, ^{n}Bu-C*H*), 0.79 (t, $^3J_{H\text{-}H}$ = 7.1 Hz, 3H, ^{n}Bu-CH_3); ^{19}F{^{1}H} NMR (470 MHz, $CDCl_3$) δ −72.0 (s, 3F, CF_3), −75.8 (q, $^4J_{F\text{-}F}$ = 9.1 Hz, 3F, CF_3), −76.3 (q, $^4J_{F\text{-}F}$ = 8.9 Hz, 3F, CF_3), −76.6 (dq, $^4J_{F\text{-}F}$ = 10.4, 5.2 Hz, 3F, CF_3); ^{31}P{^{1}H} NMR (202 MHz, $CDCl_3$) δ −32.9; HRMS (ESI$^-$): *m/z* calcd. 673.1171 for ([M−H]$^-$), found 673.1166.

3-Bromo-6-methoxy-2-naphthoic acid (S5g)

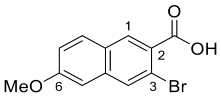

The title compound was prepared according to the modified literature procedure.[6] In a 1.0 L round-bottom flask iodobenzene diacetate (16.43 g, 50.0 mmol, 2.00 eq.) and I_2 (12.69 g, 50.0 mmol, 2.00 eq.) were dissolved in DMF (250 mL) under atmospheric air. The reaction was stirred at r.t. for 1 h and 6-methoxy-2-naphthoic acid (5.06 g, 25.0 mmol, 1.00 eq.), $Pd(OAc)_2$ (281 mg, 1.25 mmol, 5.00 mol%) and tetramethylammonium bromide (5.78 g, 37.5 mmol, 1.50 eq.) were added under atmospheric air. A reflux condenser with deflated balloon was attached to the flask, and the reaction mixture was stirred at 100 °C for 24 h. The reaction mixture was cooled to room temperature and 10% aq. Na_2CO_3 (265 mL) was added. The mixture was washed with Et_2O (2 x 200 mL), and the aqueous layer was acidified with HCl (12.0 molL^{-1}, 63 mL). The aqueous layer was extracted with EtOAc (4 x 200 mL), and the combined organic phases were washed with sat. aq. $Na_2S_2O_3$ (3 x 100 mL), dried over Na_2SO_4 and concentrated under vacuum. The crude material thus obtained was used in the next step without further purification.

2,3,4,5,6-Pentafluorophenyl 3-bromo-6-methoxy-2-naphthoate (S1g)

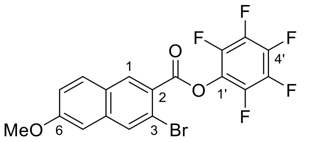

Following the general procedure **A**, the reaction of acid **S5g** (2.63 g, 9.40 mmol, 1.00 eq.), pentafluorophenol (1.93 g, 10.5 mmol, 1.12 eq.) and *N,N'*-dicyclohexylcarbodiimide (2.18 g, 10.6 mmol, 1.13 eq.) in THF (47 mL) provided the title compound after column chromatography on silica gel (petroleum ether) as yellow crystals (680 mg, 1.52 mmol, 16%, m.p. = 96 – 97 °C); R_f 0.30 (petroleum ether); IR $\tilde{\nu}_{max}$ (neat): 2934m, 2859m, 2361m, 1764s, 1622s, 1518s, 1462m, 1390m, 1249m, 1185s, 1051m, 993s, 909m, 816m, 733m, 651w; ^{1}H NMR (500 MHz, $CDCl_3$) δ 8.67 (s, 1H, C4*H*), 8.14 (s, 1H, C1*H*), 7.86 (d, $^3J_{H\text{-}H}$ = 9.0 Hz, 1H, C8*H*), 7.29 – 7.25 (m, 1H, C9*H*), 7.09 (d, $J_{H\text{-}H}$ = 2.5 Hz, 1H, C5*H*), 3.98 (s, 3H, OCH_3); ^{13}C{^{1}H} NMR (126 MHz, $CDCl_3$) δ 161.3 (*C*O_2R), 161.0 (*C*7), 142.3 (C_6F_5-*C*), 140.6 (C_6F_5-*C*), 140.4 (C_6F_5-*C*), 138.9 (C_6F_5-*C*), 138.6 (C_6F_5-*C*), 138.0 (*C*2), 136.9 (C_6F_5-*C*), 134.5 (*C*1), 132.6 (*C*9), 130.8 (*C*4), 126.4 (*C*5), 122.1 (*C*10), 120.8 (*C*8), 118.4 (*C*3), 104.6 (*C*6), 55.5 (O*C*H_3); ^{19}F{^{1}H} NMR (376 MHz, $CDCl_3$) δ −151.8 – −152.1 (m, 2F, C2'*F* and C6'*F*), −157.7 (t, *J* = 21.8 Hz,

1F, C4’*F*), −162.0 – −162.3 (m, 2F, C3’*F* and C5’*F*); HRMS (ESI$^-$): *m/z* calcd. 182.9875 for ([C_6F_5O]$^-$), found 182.9878.

((2-(3-Bromo-6-methoxynaphthalen-2-yl)-1,1,1,3,3,3-hexafluoropropan-2-yl)oxy)trimethylsilane (S2g)

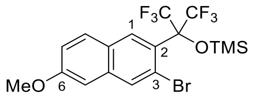

Following the general procedure **B**, the reaction of pentafluorophenyl ester **S1g** (0.68 g, 1.52 mmol, 1.00 eq.), trimethyl(trifluoromethyl)silane (1.35 mL, 9.12 mmol, 6.00 eq.) and tetrabutylammonium fluoride (1.00 molL^{-1} in THF, 0.50 mL, 0.53 mmol, 0.35 eq.) in toluene (8.0 mL) provided the title compound after column chromatography on silica gel (petroleum ether) as yellow crystals (482 mg, 1.01 mmol, 67%, m.p. = 71 – 72 °C); R_f 0.80 (petroleum ether); IR $\tilde{\nu}_{max}$ (neat): 2961m, 2845w, 2360m, 1630m, 1492m, 1396m, 1249m, 1214s, 1189s, 1004m, 848s, 727m, 636w; ^{1}H NMR (500 MHz, $CDCl_3$) δ 8.15 (s, 1H, C4*H*), 8.06 (s, 1H, C1*H*), 7.73 (d, $^3J_{H-H}$ = 9.0 Hz, 1H, C8*H*), 7.19 (dd, J_{H-H} = 9.0, 2.5 Hz, 1H, C9*H*), 7.00 (d, J_{H-H} = 2.6 Hz, 1H, C5*H*), 3.93 (s, 3H, OCH_3), 0.24 (s, 9H, Si(CH_3)$_3$); ^{13}C{^{1}H} NMR (151 MHz, $CDCl_3$) δ 159.8 (*C*7), 135.5 (*C*2), 134.6 (*C*4), 130.5 (*C*9), 130.2 (*C*1), 126.6 (*C*5), 124.5 (*C*10), 123.0 (q, $^1J_{C-F}$ = 292.5 Hz, 2C, C(CF_3)), 120.5 (*C*8), 118.7 (*C*3), 103.9 (*C*6), 82.1 (hept, $^1J_{C-F}$ = 29.9 Hz, *C*(CF_3)$_2$), 55.6 (O*C*H_3), 1.6 (Si(*C*H_3)$_3$); ^{19}F{^{1}H} NMR (470 MHz, $CDCl_3$) δ −70.1 (6F, C(CF_3)$_2$); HRMS (ESI$^-$): *m/z* calcd. 400.9617 for ([M−TMS]$^-$), found 400.9622.

2-(3-Bromo-6-methoxynaphthalen-2-yl)-1,1,1,3,3,3-hexafluoropropan-2-ol (S3g)

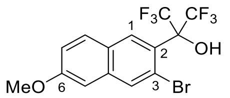

Following the general procedure **C**, the reaction of **S2g** (310 mg, 0.65 mmol, 1.00 eq.) in THF (3.5 mL) and hydrochloric acid (12.0 molL^{-1}, 3.50 mL) provided the title compound after column chromatography on silica gel (petroleum ether/EtOAc 5:1) as white crystals (173 mg, 0.43 mmol, 43%, m.p. = 101 – 102 °C); R_f 0.30 (petroleum ether/EtOAc 5:1); IR $\tilde{\nu}_{max}$ (neat): 3700m, 3485m, 2970s, 2866m, 2361m, 1629m, 1492m, 1396m, 1210s, 1119m, 1054s, 1032s, 896m, 726m, 642w; ^{1}H NMR (500 MHz, $CDCl_3$) δ 8.11 (d, $^3J_{H-H}$ = 10.2 Hz, 2H, C1*H* and C4*H*), 7.76 (d, $^3J_{H-H}$ = 9.0 Hz, 1H, C8*H*), 7.25 – 7.21 (m, 1H, C9*H*), 7.02 (s, 1H, C6*H*), 5.45 (br. s, 1H, O*H*), 3.93 (s, 3H, C6OCH_3); ^{13}C{^{1}H} NMR (151 MHz, $CDCl_3$) δ 160.1 (*C*7), 135.7 (*C*2), 134.0 (*C*4), 130.7 (*C*1), 130.5 (*C*9), 126.8 (*C*5), 122.8 (q, $^1J_{C-F}$ = 289.6 Hz, 2C, C(CF_3)$_2$), 121.1 (*C*8 and *C*10), 116.5 (*C*3), 104.0 (*C*6), 80.3 (hept, $^2J_{C-F}$ = 30.1 Hz, *C*(CF_3)$_2$), 55.6 (C6O*C*H_3); ^{19}F{^{1}H} NMR (470 MHz, $CDCl_3$) δ −73.4 (6F, C(CF_3)$_2$); HRMS (ESI$^-$): *m/z* calcd. 400.9617 for ([M−H]$^-$), found 400.9623.

[*TBPY*-5-11']-7,7'-Dimethoxy-1-hydro-3,3,3',3'-tetrakis(trifluoromethyl)-3*H*,3'*H*-1λ^5,1'-spirobi[naphtho[2,3-*c*][2,1]oxa-phosphole] (S4g)

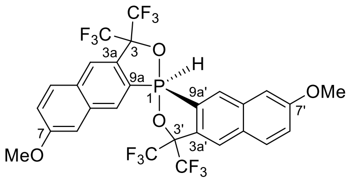

Following the general procedure **D**, the reaction of alcohol **S3g** (173 mg, 0.43 mmol, 1.00 eq.), NaH (11.3 mg, 0.47 mmol, 1.10 eq.), ^{t}BuLi (1.70 molL^{-1} in pentane, 0.60 mL, 0.94 mmol, 2.20 eq.) and PCl_3 (19.0 µL, 18.7 µmol, 0.50 eq.) in Et_2O (4.0 mL) provided the title compound after column chromatography on silica gel (petroleum ether) as white crystals (28.0 mg, 41.4 µmol, 19%, m.p. = 230 – 231 °C); R$_f$ 0.60 (petroleum ether); IR $\tilde{\nu}_{max}$ (neat): 3705m, 2969s, 2361m, 1248m, 1192m, 1054s, 1033s, 1013m, 848m, 731m, 652w; ^{1}H NMR (500 MHz, $CDCl_3$) δ 8.70 (d, $^3J_{H-P}$ = 14.4 Hz, 2H, C9*H* and C9'*H*), 8.42 (d, $^1J_{H-P}$ = 730.5 Hz, 1H, P*H*), 8.09 (s, 2H, C4*H* and C4'*H*), 7.82 (d, $^3J_{H-H}$ = 9.0 Hz, 2H, C5*H* and C5'*H*), 7.30 (dd, $^3J_{H-H}$ = 9.0 Hz, $^4J_{H-H}$ = 2.5 Hz, 2H, C6*H* and C6'*H*), 7.19 (s, 2H, C8*H* and C8'*H*), 3.91 (s, 6H, 2OC*H*$_3$); ^{13}C{^{1}H} NMR (151 MHz, $CDCl_3$) δ 159.3 (d, $^5J_{C-P}$ = 1.9 Hz, 2C, *C*7 and *C*7'), 138.2 (d, $^2J_{C-P}$ = 10.7 Hz, 2C, *C*9 and *C*9'), 136.2 (d, $^3J_{C-P}$ = 16.6 Hz, 2C, *C*8a and *C*8a'), 131.4 (d, $^4J_{C-P}$ = 3.0 Hz, 2C, *C*4a and *C*4a'), 129.9 (d, $^5J_{C-P}$ = 1.6 Hz, 2C, *C*5 and *C*5'), 129.3 (d, $^2J_{C-P}$ = 22.7 Hz, 2C, *C*3a and *C*3a'), 125.0 (d, $^3J_{C-P}$ = 16.1 Hz, 2C, *C*4 and *C*4'), 123.3 (2C, *C*6 and *C*6'), 122.9 (d, $^1J_{C-P}$ = 160.2 Hz, 2C, *C*9a and *C*9a'), 107.0 (2C, *C*8 and *C*8'), 55.7 (2C, O*C*H_3); *C*3, *C*3', C3*C*F_3 and C3'*C*F_3 signals were not observed due to low concentration of the sample; ^{19}F{^{1}H} NMR (376 MHz, $CDCl_3$) δ −74.4 (q, $^4J_{F-F}$ = 9.1 Hz, 6F, C3C*F*$_3$ and C3'C*F*$_3$), −76.0 (qd, J_{F-F} = 9.0, 3.1 Hz, 6F, C3C*F*$_3$ and C3'C*F*$_3$); ^{31}P{^{1}H} NMR (202 MHz, $CDCl_3$) δ −49.1; HRMS (ESI$^-$): *m/z* calcd. 675.0600 for ([M−H]$^-$), found 675.0608.

[*TBPY*-5-15]-1,1,1,3,3,3-Hexafluoro-2-(3-(1-butyl-1-hydro-7-methoxy-3,3-bis(trifluoromethyl)-3*H*-1λ^5-naphtho[2,3-*c*][2,1]oxaphosphol-1-yl)-6-methoxynaphthalen-2-yl)propan-2-ol (1g)

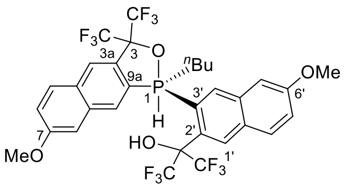

Following the general procedure **E**, the reaction of **S4g** (28.0 mg, 41.4 µmol, 1.00 eq.) and ^{n}BuLi (1.60 molL^{-1} in hexanes, 0.18 mL, 0.29 mmol, 7.00 eq.) in Et_2O (1.0 mL) provided the title compound after column chromatography on silica gel (petroleum ether/EtOAc 20:1) as white crystals (22.0 mg, 30.0 µmol, 73%, m.p. = 182 – 183 °C (decomposition)); R$_f$ 0.20 (petroleum ether/EtOAc 20:1); IR $\tilde{\nu}_{max}$ (neat): 37570m, 2962s, 2934m, 2183m, 2138m, 1629m, 1503m, 1467m, 1272m, 1194s, 1029m, 966m, 899m, 653w; ^{1}H NMR (400 MHz, $CDCl_3$) δ 9.37 (br. s, 1H, O*H*), 8.54 (d, *J* = 17.6 Hz, 1H, Ar-C*H*), 8.27 (s, 1H, Ar-C*H*), 8.12 (s, 1H, Ar-C*H*), 8.02 – 7.88 (m, 2H, Ar-C*H H*), 7.77 (d, *J* = 9.0 Hz, 1H, Ar-C*H*), 7.48 (d, *J* = 2.5 Hz, 1H, Ar-C*H*), 7.41 (dd, *J* = 9.0, 2.5 Hz, 1H, Ar-C*H*), 7.20 (dd, *J* = 8.9, 2.5 Hz, 1H, Ar-C*H*), 6.90 (d, *J* = 2.5 Hz, 1H, Ar-C*H*), 6.41 (dd, $^1J_{H-P}$ = 276.2 Hz, *J* = 5.8 Hz, 1H, P*H*), 4.05 (s, 3H, C7C*H*$_3$ or C6'C*H*$_3$), 3.83 (s, 3H, C7C*H*$_3$ or C6'C*H*$_3$), 3.22 (q, *J* = 14.5 Hz, 1H, ^{n}Bu-C*H*), 2.64 (ddt, *J* = 21.9, 14.5, 6.3 Hz, 1H, ^{n}Bu-C*H*), 1.67 (d, *J* = 7.3 Hz, 1H, ^{n}Bu-C*H*), 1.46 – 1.36 (m, 3H,

^{n}Bu-C*H*), 0.88 (t, $^3J_{H\text{-}H}$ = 7.2 Hz, 3H, ^{n}Bu-CH_3); ^{19}F{^{1}H} NMR (376 MHz, $CDCl_3$) δ −72.2 (s, 3F, CF_3), −75.9 (q, $^4J_{F\text{-}F}$ = 9.1 Hz, 3F, CF_3), −76.4 (d, $^4J_{F\text{-}F}$ = 9.1 Hz, 3F, CF_3), −76.7 (dt, $^4J_{F\text{-}F}$ = 9.3, 5.0 Hz, 3F, CF_3); Due to long NMR measurements, dehydrogenation side-product was formed. ^{31}P{^{1}H} NMR (162 MHz, $CDCl_3$) δ −5.8 (dehydrogenation side-product, (*cis*)-**2g**), −33.1 (**1g**); HRMS (ESI$^-$): *m/z* calcd. 733.1383 for ([M−H]$^-$), found 733.1395.

[*TBPY*-5-15]-1,1,1,3,3,3-Hexafluoro-2-(4-methyl-2-(1-hexyl-1-hydro-6-methyl-3,3-bis(trifluoromethyl)-3*H*-1λ^5-benzo[*c*][2,1]oxaphosphol-1-yl)phenyl)propan-2-ol (1h)

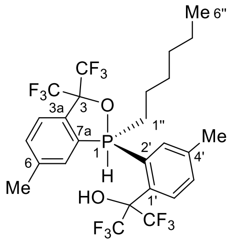

Following the general procedure **E**, the reaction of **S4a** (150 mg, 0.28 mmol, 1.00 eq.) and hexylmagnesium bromide (1.00 molL^{-1} in THF, 1.93 mL, 1.93 mmol, 7.00 eq.) in Et_2O (3.0 mL) provided the title compound after column chromatography on silica gel (petroleum ether) as white crystals (158 mg, 0.26 mmol, 95%, m.p. = 85 – 86 °C (decomposition)); R_f 0.20 (petroleum ether); IR $\tilde{\nu}_{max}$ (neat): 3310s, 2943m, 2831m, 2361m, 2043w, 1452m, 1417m, 1115w, 1022s; ^{1}H NMR (500 MHz, $CDCl_3$) δ 9.10 (br. s, 1H, O*H*), 7.79 – 7.69 (m, 2H, Ar-C*H*), 7.68 – 7.63 (m, 1H, Ar-C*H*), 7.59 (d, *J* = 8.4 Hz, 1H, Ar-C*H*), 7.22 (d, *J* = 8.8 Hz, 2H, Ar-C*H*), 6.01 (d, $^1J_{H\text{-}H}$ = 269.6 Hz, 1H, P*H*), 3.16 – 2.99 (m, 1H, C1''*H*), 2.60 (s, 3H, C6CH_3 or C4'CH_3), 2.56 – 2.48 (m, 1H, C1''H), 2.25 (s, 3H, C6CH_3 or C4'CH_3), 1.57 (s, 2H, C2''*H*), 1.35 (q, *J* = 6.9 Hz, 2H, C3''*H*), 1.26 – 1.22 (m, 4H, C5''*H* and , C4''*H*), 0.84 (t, $^3J_{H\text{-}H}$ = 6.5 Hz, 3*H*, C6''H); ^{19}F{^{1}H} NMR (470 MHz, $CDCl_3$) δ −72.6 (s, 3F, CF_3), −75.8 (q, $^4J_{F\text{-}F}$ = 9.0 Hz, 3F, CF_3), −76.1 – −76.4 (m, 3F, CF_3), −76.6 (dt, $^4J_{F\text{-}F}$ = 9.8, 4.9 Hz, 3F, CF_3); Due to long NMR measurements, dehydrogenation side-products were formed. ^{31}P{^{1}H} NMR (202 MHz, $CDCl_3$) δ −4.3 (dehydrogenation side-product, (*cis*)-**2h**), −19.8 (dehydrogenation side-product, (*trans*)-**2h**), −34.9 (**1h**); HRMS (ESI$^-$): *m/z* calcd. 629.1484 for ([M−H]$^-$), found 629.1495.

[*TBPY*-5-15]-1,1,1,3,3,3-Hexafluoro-2-(4-methyl-2-(1-hydro-6-methyl-1-(5-methylhexyl)-3,3-bis(trifluoro-methyl)-3*H*-1λ^5-benzo[*c*][2,1]oxaphosphol-1-yl)phenyl)propan-2-ol (1i)

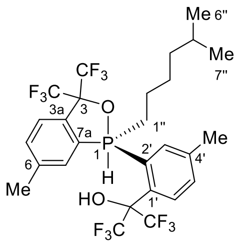

Following the general procedure **F**, the reaction with **S4a** (167 mg, 0.31 mmol, 1.00 eq.), 1-bromo-5-methylhexane (385 mg, 2.15 mmol, 7.00 eq.) and magnesium (53 mg, 2.18 mmol, 7.10 eq.) provided the title compound after column chromatography on silica gel (pentane) as colourless oil (159 mg, 0.25 mmol, 80%). R_f.0.30 (pentane); IR $\tilde{\nu}_{max}$ (neat): 3091m, 2932w, 2125w, 1273s, 1195s, 1077m, 963s, 935w, 824w, 703m, 632m; ^{1}H NMR (400 MHz, $CDCl_3$) δ 9.11 (br. s, 1H, O*H*), 7.90 – 7.49 (m, 4H, Ar-C*H*), 7.29 – 7.13 (m, 2H, Ar-C*H* overlapping

with $CDCl_3$), 6.01 (d, $^{1}J_{H\text{-}P}$ = 272.8 Hz, 1H, P*H*), 3.08 – 3.04 (m, 1H, Alk-C*H*), 2.60 (s, 3H, C6CH_3 or C4'CH_3), 2.56 – 2.46 (m, 1H, Alk-C*H*), 2.25 (s, 3H, C6CH_3 or C4'CH_3), 1.53 – 1.39 (m, 2H, C5''*H* overlapping with Alk-C*H*), 1.39 – 1.20 (m, 3H, Alk-C*H*), 1.11 (q, *J* = 7.3 Hz, 2H, Alk-C*H*), 0.82 (d, $^{3}J_{H\text{-}H}$ = 6.7 Hz, 6H, C6"H_3 and C7"H_3); $^{19}F\{^{1}H\}$ NMR (376 MHz, $CDCl_3$) δ −72.6 (3F, CF_3), −75.9 (d, $^{4}J_{F\text{-}F}$ = 9.3 Hz, 3F, CF_3), −76.3 (d, $^{4}J_{F\text{-}F}$ = 10.6 Hz, 3F, CF_3), −76.6 (3F, CF_3); Due to long NMR measurements, dehydrogenation side-product was formed. $^{31}P\{^{1}H\}$ NMR (162 MHz, $CDCl_3$) δ −4.3 (dehydrogenation side-product, (*cis*)-**2i**), −34.9 (**1i**); HRMS (ESI$^+$): *m/z* calcd. 645.1786 for ([M+H]$^+$), found 645.1788.

[*TBPY*-5-15]-1,1,1,3,3,3-Hexafluoro-2-(4-methyl-2-(1-hydro-6-methyl-1-(4-phenylbutyl)-3,3-bis(trifluoromethyl)-3*H*-1λ^5-benzo[*c*][2,1]oxaphosphol-1-yl)phenyl)propan-2-ol (1j)

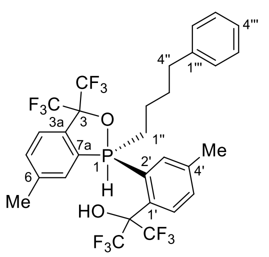

Following the general procedure **F**, the reaction with **S4a** (167 mg, 0.31 mmol, 1.00 eq.), 1-bromo-4-phenylbutane (458 mg, 2.15 mmol, 7.00 eq.) and magnesium (53 mg, 2.18 mmol, 7.10 eq.) provided the title compound after column chromatography on silica gel (pentane/CH_2Cl_2 4:1) as white solid (70 mg, 0.10 mmol, 34%, m.p. = 123 – 124 °C (decomposition)); R_f 0.70 (pentane/CH_2Cl_2 4:1); IR $\tilde{\nu}_{max}$ (neat): 3029m, 2930w, 2361w, 2130w, 1272s, 1193s, 1148s, 1077m, 963s, 873w, 825w, 701s; ^{1}H NMR (400 MHz, $CDCl_3$) δ 9.08 (s, 1H, O*H*), 7.75 – 7.56 (m, 4H, Ar-C*H*), 7.26 – 7.12 (m, 5H, Ar-C*H*), 7.09 (dd, *J* = 7.0, 1.7 Hz, 2H, Ar-C*H*), 5.98 (dd, *J* = 272.8, *J* = 5.6 Hz, 1H, P*H*), 3.21 – 3.05 (m, 1H, Alk-C*H*), 2.59 (s, 3H, C6CH_3 or C4'CH_3), 2.58 – 2.45 (m, 3H, Alk-C*H*), 2.25 (s, 3H, C6CH_3 or C4'CH_3), 1.69 (pd, *J* = 7.6, 2.1 Hz, 2H, Alk-C*H*), 1.62 – 1.57 (m, 1H, Alk-C*H*), 1.34 (tq, *J* = 12.1, 6.6 Hz, 1H, Alk-C*H*); $^{19}F\{^{1}H\}$ NMR (376 MHz, $CDCl_3$) δ −72.6 (3F, C*F*3), −75.8 (q, $^{4}J_{F\text{-}F}$ = 9.2 Hz, 3F, C*F*3), −76.3 (d, $^{4}J_{F\text{-}F}$ = 9.8 Hz, 3F, C*F*3), −76.6 (tq, $^{4}J_{F\text{-}F}$ = 9.6, *J* = 4.6 Hz, 3F, C*F*3); $^{31}P\{^{1}H\}$ NMR (162 MHz, $CDCl_3$) δ −35.5; HRMS (ESI$^+$): *m/z* calcd. 679.1630 for ([M+H]$^+$), found 679.1636.

[*TBPY*-5-15]-1,1,1,3,3,3-Hexafluoro-2-(4-methyl-2-(1-hydro-6-methyl-1-(pent-4-en-1-yl)-3,3-bis(trifluoromethyl)-3*H*-1λ^5-benzo[*c*][2,1]oxaphosphol-1-yl)phenyl)propan-2-ol (1k)

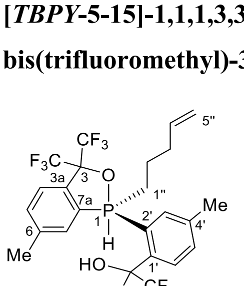

Following the general procedure **E**, the reaction of **S4a** (150 mg, 0.28 mmol, 1.00 eq.) and 4-penten-1-yl-magnesium bromide (1.00 molL^{-1} in THF, 1.93 mL, 1.93 mmol, 7.00 eq.) in Et_2O (3.0 mL) provided the title compound after column chromatography on silica gel (petroleum ether) as white solid (136 mg, 0.23 mmol, 82%, m.p. = 62 – 63 °C (decomposition)); R_f 0.20 (petroleum ether); IR $\tilde{\nu}_{max}$ (neat): 3320s, 2945m, 2832m, 2361m, 2340m, 1650w, 1453m, 1417m, 1193w, 1116w, 1019s, 668m; ^{1}H NMR (500 MHz, $CDCl_3$) δ 9.04 (br. s, 1H, O*H*), 7.81 – 7.69 (m, 2H, Ar-C*H*), 7.65 (s, 1H, Ar-C*H*), 7.59 (d, *J* = 8.1 Hz, 1H, Ar-C*H*), 7.23 (d, *J* = 9.2 Hz, 2H,

Ar-CH), 6.02 (d, $^1J_{H\text{-}P}$ = 266.4 Hz, 1H, PH), 5.74 – 5.64 (m, 1H, C4''H), 5.03 – 4.90 (m, 2H, C5''H), 3.10 (q, J = 14.5 Hz, 1H, Alk-CH), 2.60 (s, 3H, ArCH_3), 2.56 – 2.45 (m, 1H, Alk-CH), 2.25 (s, 3H, ArCH_3), 2.11 (q, J = 7.3 Hz, 2H, Alk-CH), 1.68 – 1.60 (m, 1H, Alk-CH), 1.40 (dd, J = 13.0, 6.2 Hz, 1H, Alk-CH); ^{19}F{^{1}H} NMR (470 MHz, $CDCl_3$) δ −72.6 (3F, CF_3), −75.8 (q, $^4J_{F\text{-}F}$ = 9.3 Hz, 3F, CF_3), −76.3 (d, J = 9.8 Hz, 3F, CF_3), −76.6 (dq, J = 9.9, 4.9 Hz, 3F, CF_3); Due to long NMR measurements, dehydrogenation side-products were formed. ^{31}P{^{1}H} NMR (202 MHz, $CDCl_3$) δ −4.4 (dehydrogenation side-product (*cis*)-**2k**), −20.1 (dehydrogenation side-product (*trans*)-**2k**), −35.8 (**1k**). HRMS (ESI$^-$): *m/z* calcd. 613.1171 for ([M−H]$^-$), found 613.1182.

[*TBPY*-5-15]-1,1,1,3,3,3-Hexafluoro-2-(4-methyl-2-(1-hydro-6-methyl-1-phenethyl-3,3-bis(trifluoromethyl)-3*H*-1λ⁵-benzo[*c*][2,1]oxaphosphol-1-yl)phenyl)propan-2-ol (1l)

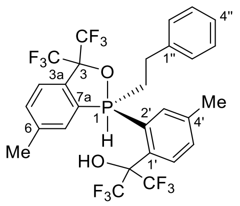

Following the general procedure **E**, the reaction of **S4a** (150 mg, 0.28 mmol, 1.00 eq.) and phenethylmagnesium chloride solution (1.00 molL^{-1} in THF, 1.93 mL, 1.93 mmol, 7.00 eq.) in Et_2O (3.0 mL) provided the title compound after column chromatography on silica gel (petroleum ether) as white solid (126 mg, 0.21 mmol, 76%, m.p. = 145 − 146 °C (decomposition)); R_f 0.20 (petroleum ether); IR $\tilde{\nu}_{max}$ (neat): 3308s, 2943m, 2831m, 2361m, 1452m, 1417m, 1115w, 1021s, 650m. ^{1}H NMR (500 MHz, $CDCl_3$) δ 9.04 (br. s, 1H, OH), 7.76 (t, J = 8.3 Hz, 1H, Ar-CH), 7.68 (d, J = 15.7 Hz, 2H, Ar-CH), 7.62 (d, J = 7.9 Hz, 1H, Ar-CH), 7.33 – 7.26 (m, 4H, Ar-CH), 7.24 – 7.20 (m, 1H, Ar-CH), 7.17 – 7.12 (m, 2H, Ar-CH), 6.17 (d, $^1J_{H\text{-}P}$ = 271.1 Hz, 1H, PH), 3.47 (q, J = 13.9 Hz, 1H, Alk-CH), 2.89 (q, J = 14.2 Hz, 2H, Alk-CH), 2.69 – 2.62 (m, 1H, Alk-CH), 2.60 (s, 3H, ArCH_3), 2.29 (s, 3H, ArCH_3); ^{19}F NMR (470 MHz, $CDCl_3$) δ −72.6 (3F, CF_3), −75.7 (d, J = 9.3 Hz, 3F, CF_3), −76.0 – −76.3 (m, 3F, CF_3), −76.6 (dq, J = 10.0, 4.9 Hz, 3F, CF_3); Due to long NMR measurements, dehydrogenation side-products were formed. ^{31}P NMR (202 MHz, $CDCl_3$) δ −6.0 (dehydrogenation side-product (*cis*)-**2l**), −22.0 (dehydrogenation side-product (*trans*)-**2l**), −38.0 (**1l**); HRMS (ESI$^+$): *m/z* calcd. 651.1317 for ([M+H]$^+$), found 651.1327.

[*TBPY*-5-15]-1,1,1,3,3,3-Hexafluoro-2-(2-(1,6-dimethyl-1-hydro-3,3-bis(trifluoromethyl)-3*H*-1λ⁵-benzo[*c*][2,1]oxaphosphol-1-yl)-4-methylphenyl)propan-2-ol (1m)

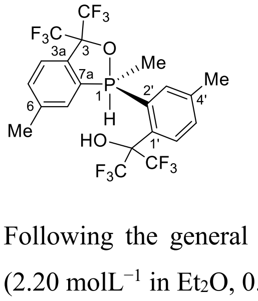

Following the general procedure **E**, the reaction of **S4a** (150 mg, 0.28 mmol, 1.00 eq.) and methyllithium (2.20 molL^{-1} in Et_2O, 0.88 mL, 1.93 mmol, 7.00 eq.) in Et_2O (3.0 mL) provided the title compound after column chromatography on silica gel (pentane/CH_2Cl_2 4:1) as white crystals (120 mg, 0.21 mmol, 78%, m.p. = 101 – 102 °C (decomposition)); R_f 0.70 (pentane/CH_2Cl_2 4:1); IR $\tilde{\nu}_{max}$ (neat): 3092m, 2793w, 2361m, 2136w, 1493w, 1259s, 1192s, 1147s, 1078m, 963s, 908w, 825m, 774m, 700m; ^{1}H NMR (400 MHz, $CDCl_3$) δ 8.94 (br. s,

1H, O*H*), 7.88 – 7.50 (m, 4H, Ar-C*H*), 7.27 – 7.18 (m, 2H, Ar-C*H* overlapping with $CDCl_3$), 6.28 (dq, $^1J_{H-P}$ = 275.6 Hz, $^3J_{H-H}$ = 4.1 Hz, 1H, P*H*), 2.60 (s, 3H, C6CH_3 or C4'CH_3), 2.39 (dd, $^2J_{H-P}$ = 14.9 Hz, $^3J_{H-H}$ = 4.0 Hz, 3H, PCH_3), 2.26 (s, 3H, C6CH_3 or C4'CH_3); $^{19}F\{^1H\}$ NMR (376 MHz, $CDCl_3$) δ −72.6 (3F, CF_3), −76.0 (m, 3F, CF_3), −76.5 (3F, CF_3), −76.6 – −76.9 (m, 3F, CF_3); Due to long NMR measurements, dehydrogenation side-product was formed. $^{31}P\{^1H\}$ NMR (162 MHz, $CDCl_3$) δ −23.7 (dehydrogenation side-product, (*trans*)-**2m**), −52.7 (**1m**); HRMS (ESI$^+$): *m/z* calcd. 561.0847 for ($[M+H]^+$), found 561.0852.

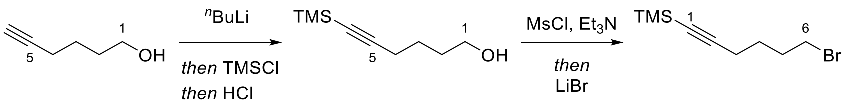

6-(Trimethylsilyl)hex-5-yn-1-ol

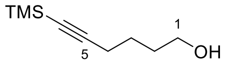

The title compound was obtained according to the modified literature procedure.[7] To a solution of 5-hexyn-1-ol (2.02 g, 20.0 mmol, 1.00 eq.) in THF (50 mL), ^{n}BuLi (1.60 molL^{-1} in hexanes, 27.5 mL, 44.0 mmol, 2.20 eq.) was slowly added under an Ar atmosphere at −78 °C. The reaction mixture was stirred for 1 h at -78 °C. TMSCl (7.83 mL, 60.0 mmol, 3.00 eq.) was added and the reaction was allowed to warm slowly to r.t. for 16 h. The reaction was cooled to 0 °C and quenched with HCl (1.00 molL^{-1}, 50 mL) and stirred at r.t. (until bis-silylated product was no longer present according to TLC). The reaction mixture was then extracted with Et_2O (3 x 40 mL) and the combined organic layers were washed with brine (40 mL), sat. aq. $NaHCO_3$ (40 mL), and once more with brine (40 mL) before drying with Na_2SO_4 and concentration under the reduced pressure. The residue was purified by flash column chromatography (hexane/EtOAc 9:1 → 7:3) to afford the title compound as a colourless liquid (3.23 g, 19.0 mmol, 95%); R_f.0.25 (hexane/EtOAc 4:1); IR $\tilde{\nu}_{max}$ (neat): 3330m, 2955m, 2361m, 2175m, 1429w, 1250s, 1047m, 841s, 634s; ^{1}H NMR (500 MHz, $CDCl_3$) δ 3.67 (t, $^3J_{H-H}$ = 6.3 Hz, 2H, C1*H*), 2.26 (t, $^3J_{H-H}$ = 6.8 Hz, 2H, C4*H*), 1.72 – 1.64 (m, 2H, C2*H*), 1.63 – 1.55 (m, 2H, C3*H*), 1.47 (br. s, 1H, O*H*), 0.13 (s, 9H, Si(CH_3)$_3$); $^{13}C\{^1H\}$ NMR (126 MHz, $CDCl_3$) δ 107.3 (*C*5), 84.90 (*C*6), 62.5 (*C*1), 31.9 (*C*2), 25.0 (*C*3), 19.7 (*C*4), 0.3 (3C, Si(*C*H_3)$_3$); HRMS (ESI$^+$): *m/z* calcd. 171.1200 for ($[M+H]^+$), found 171.1202.

(6-Bromohex-1-yn-1-yl)trimethylsilane

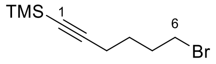

The title compound was obtained according to the modified literature procedure.[8] To a solution of 6-(trimethylsilyl)hex-5-yn-1-ol (3.10 g, 18.2 mmol, 1.00 eq.) and triethylamine (3.33 mL, 23.7 mmol, 1.30 eq.) in THF (36 mL), methanesulfonyl chloride (1.83 mL, 23.7 mmol, 1.30 eq.) was added dropwise at 0 °C. After stirring for 3 h at r.t., water (30 mL) was added, the aqueous phase extracted with CH_2Cl_2 (4 x 25 mL), the combined organic layers dried with Na_2SO_4 and concentrated under reduced pressure. The residue was dissolved in acetone (19 mL), LiBr (4.74 g, 54.6 mmol, 3.00 eq.) was added and the heterogeneous mixture stirred for 15 h at 56 °C. The reaction mixture was cooled to r.t. and diluted with water (30 mL). The aqueous phase was extracted with Et_2O (3 x 25 mL), combined organic layers dried over Na_2SO_4 and concentrated under the reduced pressure. Flash column

chromatography on silica gel (pentane/Et_2O 100:0 → 99:1) afforded the title compound as a colourless liquid (3.82 g, 16.4 mmol, 90%); R_f 0.45 (pentane); IR $\tilde{\nu}_{max}$ (neat): 2958m, 2361w, 2175m, 1433w, 1249s, 1029w, 926w, 841s, 639s; ^{1}H NMR (500 MHz, $CDCl_3$) δ 3.44 (t, $^3J_{H\text{-}H}$ = 6.7 Hz, 2H, C6*H*), 2.27 (t, $^3J_{H\text{-}H}$ = 7.0 Hz, 2H, C3*H*), 2.02 – 1.93 (m, 2H, C5*H*), 1.72 – 1.63 (m, 2H, C4*H*), 0.14 (s, 9H, Si(CH_3)$_3$); ^{13}C{^{1}H} NMR (126 MHz, $CDCl_3$) δ 106.5 (*C*2), 85.4 (*C*1), 33.4 (*C*6), 31.8 (*C*5), 27.1 (*C*4), 19.2 (*C*3), 0.3 (3C, Si(CH_3)$_3$); HRMS (ESI$^+$): *m/z* calcd. 338.9328 for ([M+Ag]$^+$), found 338.9323.

[*TBPY*-5-15]-1,1,1,3,3,3-Hexafluoro-2-(4-methyl-2-(1-hydro-6-methyl-3,3-bis(trifluoromethyl)-1-(6-(trimethylsilyl)hex-5-yn-1-yl)-3*H*-1λ^5-benzo[*c*][2,1]oxaphosphol-1-yl)phenyl)propan-2-ol (1n)

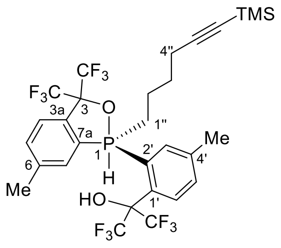

Following the general procedure **F**, the reaction with **S4a** (167 mg, 0.31 mmol, 1.00 eq.), (6-bromo-hex-1-yn-1-yl)trimethylsilane (501 mg, 2.15 mmol, 7.00 eq.) and magnesium (53 mg, 2.18 mmol, 7.10 eq.) provided the title compound after column chromatography on silica gel (pentane/CH_2Cl_2 4:1) as white solid (193 mg, 0.28 mmol, 90% , m.p. = 144 – 145 °C (decomposition)); R_f 0.70 (pentane/CH_2Cl_2 4:1); IR $\tilde{\nu}_{max}$ (neat): 3081m, 2797w, 2361w, 1411w, 1256s, 1191s, 1146s, 1077m, 963s, 934m, 841s, 733s; ^{1}H NMR (400 MHz, $CDCl_3$) δ 9.04 (br. s, 1H, O*H*), 7.88 – 7.53 (m, 4H, Ar-C*H*), 7.29 – 7.20 (m, 2H, Ar-C*H*), 6.03 (d, $^1J_{H\text{-}P}$ = 272.9 Hz, 1H, P*H*), 3.07 (q, *J* = 13.9 Hz, 1H, Alk-C*H*), 2.60 (s, 3H, C6CH_3 or C4'CH_3), 2.57 – 2.46 (m, 1H, Alk-C*H*), 2.26 (s, 3H, C6CH_3 or C4'CH_3), 2.20 (t, $^3J_{H\text{-}H}$ = 6.7 Hz, 2H, C4''*H*), 1.70 – 1.51 (m, 3H, Alk-C*H*), 1.49 – 1.34 (m, 1H, Alk-C*H*), 0.09 (s, 9H, Si(CH_3)$_3$); ^{19}F{^{1}H} NMR (376 MHz, $CDCl_3$) δ −72.6 (3F, CF_3), −75.9 (q, $^4J_{F\text{-}F}$ = 9.1 Hz, 3F, CF_3), −76.3 (d, $^4J_{F\text{-}F}$ = 9.0 Hz, 3F, CF_3), −76.6 – −76.7 (m, 3F, CF_3); Due to long NMR measurements, dehydrogenation side-product was formed. ^{31}P{^{1}H} NMR (162 MHz, $CDCl_3$) δ −4.8 (dehydrogenation side-product, (*cis*)-**2n**), −36.0 (**1n**); HRMS (ESI$^+$): *m/z* calcd. 699.1712 for ([M+H]$^+$), found 699.1712.

[*TBPY*-5-15]-1,1,1,3,3,3-Hexafluoro-2-(2-(1-hydro-1-(4-methoxybutyl)-6-methyl-3,3-bis(trifluoromethyl)-3*H*-1λ^5-benzo[*c*][2,1]oxaphosphol-1-yl)-4-methylphenyl)propan-2-ol (1o)

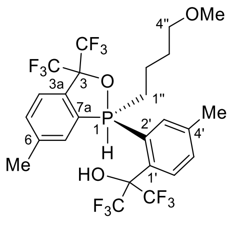

Following the general procedure **F**, the reaction with **S4a** (167 mg, 0.31 mmol, 1.00 eq.), 1-bromo-4-methoxybutane (359 mg, 2.15 mmol, 7.00 eq.) and magnesium (53 mg, 2.18 mmol, 7.10 eq.) provided the title compound after column chromatography on silica gel (pentane/CH_2Cl_2 2:1) as colourless oil (147 mg, 0.23 mmol, 76%); R_f 0.40 (pentane/CH_2Cl_2 2:1); IR $\tilde{\nu}_{max}$ (neat): 3088m, 2362m, 2130w, 1272s, 1193s, 1148s, 963s, 825m, 703m, 632s; ^{1}H NMR (400 MHz, $CDCl_3$) δ 9.09 (s, 1H, O*H*), 7.93 – 7.53 (m, 4H, Ar-C*H*), 7.32 – 7.09 (m, 2H,

Ar-C*H* overlapping with $CDCl_3$), 6.03 (d, $^{1}J_{H\text{-}P}$ = 273.0 Hz, 1H, P*H*), 3.40 – 3.30 (m, 2H, C4''H_2), 3.28 (s, 3H, OCH_3), 3.20 – 3.03 (m, 1H, Alk-C*H*), 2.59 (s, 3H, C6CH_3 or C4'CH_3), 2.54 – 2.45 (m, 1H, Alk-C*H*), 2.26 (s, 3H, C6CH_3 or C4'CH_3), 1.60 – 1.53 (m, 3H, Alk-C*H*), 1.46 – 1.34 (m, 1H, Alk-C*H*); $^{19}F\{^{1}H\}$ NMR (376 MHz, $CDCl_3$) δ −72.6 (3F, CF_3), −75.8 – −76.0 (m, 3F, CF_3), −76.3 (3F, CF_3), −76.6 (3F, CF_3); Due to long NMR measurements, dehydrogenation side-product was formed. $^{31}P\{^{1}H\}$ NMR (162 MHz, $CDCl_3$) δ −4.6 (dehydrogenation side-product, (*cis*)-**2o**), −35.4 (**1o**); HRMS (ESI$^+$): *m/z* calcd. 633.1422 for ([M+H]$^+$), found 633.1433.

[*TBPY*-5-15]-1,1,1,3,3,3-Hexafluoro-2-(2-(1-(4-(1,3-dioxolan-2-yl)butyl)-6-methyl-3,3-bis(trifluoromethyl)-3*H*-1λ^5-benzo[*c*][2,1]oxaphosphol-1-yl)-4-methylphenyl)propan-2-ol (1p)

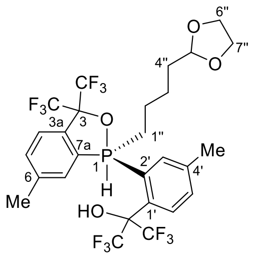

Following the general procedure **F**, the reaction with **S4a** (167 mg, 0.31 mmol, 1.00 eq.), 2-(4-bromo-butyl)-1,3-dioxolane (449 mg, 2.15 mmol, 7.00 eq.) and magnesium (53 mg, 2.18 mmol, 7.10 eq.) provided the title compound after column chromatography on silica gel (pentane/CH_2Cl_2 2:1) as colourless oil (145 mg, 0.22 mmol, 70%); R_f 0.30 (pentane/CH_2Cl_2 2:1); IR $\tilde{\nu}_{max}$ (neat): 3066m, 2362m, 2127w, 1410w, 1272s, 1193s, 1147s, 1077m, 963s, 825m, 774m, 703m, 648w; ^{1}H NMR (400 MHz, $CDCl_3$) δ 9.04 (br. s, 1H, O*H*), 7.84 – 7.55 (m, 4H, Ar-C*H*), 7.37 – 7.15 (m, 2H, Ar-C*H*), 6.01 (d, *J* = 272.5 Hz, 1H, P*H*), 4.80 (t, *J* = 4.5 Hz, 1H, C5''*H*), 3.95 – 3.87 (m, 2H, C6''*H* and C7''*H*), 3.86 – 3.78 (m, 2H, C6''*H* and C7''*H*), 3.20 – 2.99 (m, 1H, Alk-C*H*), 2.69 – 2.46 (m, 4H, C6CH_3 or C4'CH_3 overlapping with Alk-C*H*), 2.25 (s, 3H, C6CH_3 or C4'CH_3), 1.64 – 1.55 (m, 3H, Alk-C*H*), 1.54 – 1.46 (m, 2H, Alk-C*H*), 1.41 – 1.28 (m, 1H, Alk-C*H*); $^{19}F\{^{1}H\}$ NMR (376 MHz, $CDCl_3$) δ −72.6 (3F, CF_3), −75.7 – −76.0 (m, 3F, CF_3), −76.2 – −76.4 (m, 3F, CF_3), −76.5 – −76.8 (m, 3F, CF_3); Due to long NMR measurements, dehydrogenation side-product was formed. $^{31}P\{^{1}H\}$ NMR (162 MHz, $CDCl_3$) δ −4.8 (dehydrogenation side-product, (*cis*)-**2p**), −35.7 (**1p**); HRMS (ESI$^+$): *m/z* calcd. 675.1528 for ([M+H]$^+$), found 675.1535.

Optimisation of the reaction conditions for *trans*-/enantioselective catalyst control over pentavalent stereocentres

Initial catalyst, solvent, and temperature evaluation

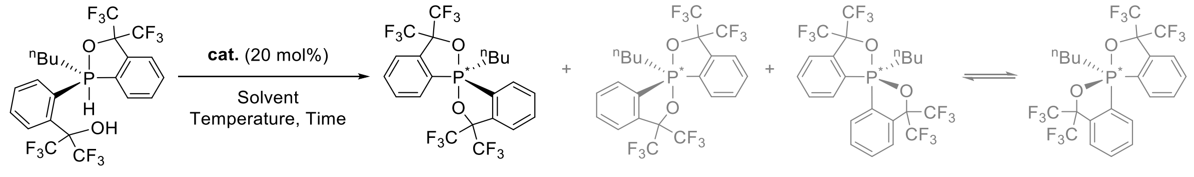

General procedure for the initial evaluation of the catalyst and reaction conditions

In a 1.5 mL crimp cap vial, catalyst (0.40 µmol, 20.0 mol%) and compound **1b** (1.15 mg, 2.00 µmol, 1.00 eq.) were dissolved in the specified solvent (0.30 mL) under an Ar atmosphere. The reaction mixture was stirred at the indicated temperature for the time indicated. The solvent was removed under a stream of nitrogen. The residue was analysed by ^{19}F NMR and HPLC on a chiral stationary phase to determine the conversion, d.r., and e.r.

Supplementary Table 1. Catalyst, solvent, and temperature evaluation for *trans*-/enantioselective dehydrogenation of **1b** on 2.00 µmol scale

Entry	Cat.	Solvent	Temp., C	Time, h	Conv., %	e.r.	d.r.
1	**-**	Toluene	40	20	19	-	59:41
2	**C1**	Toluene	40	20	100	52:48	79:21
3	**C4**	Toluene	40	20	36	n.d.	80:20
4	**SC1**	Toluene	40	20	46	49:51	84:16
5	**SC2**	Toluene	40	20	26	51:49	72:28
6	**SC3**	Toluene	40	20	48	51:49	77:23
7	**SC4**	Toluene	40	20	62	52:48	88:12
8	**SC5**	Toluene	40	20	31	51:49	70:30
9	**C5**	CH2Cl2	40	20	100	53:47	98:2
10	**C5**	Toluene	r.t.	20	100	57:43	96:4
11	**SC6**	Toluene	r.t.	20	100	53:47	>99:1
12	**SC7**	Toluene	r.t.	20	100	51:49	96:4
13	**SC8**	Toluene	r.t.	20	100	56:44	97:3

14	**SC9**	Toluene	r.t.	20	100	55:45	98:2
15	**SC10**	Toluene	r.t.	20	100	50:50	97:3
16	**SC11**	Toluene	r.t.	20	100	48:52	>99:1
17	**SC12**	Toluene	r.t.	20	100	47:53	97:3
18	**SC13**	Toluene	r.t.	20	100	47:53	99:1
19	**SC14**	Toluene	r.t.	20	100	49:51	>99:1
20	**SC15**	Toluene	r.t.	20	100	49:51	>99:1
21	**SC16**	Toluene	r.t.	12	100	76:24	>99:1
22	**SC17**	Toluene	r.t.	12	100	76:24	99:1
23	**SC18**	Toluene	r.t.	12	100	77:23	99:1
24	**SC19**	Toluene	r.t.	12	40	50:50	76:24
25	**SC20**	Toluene	r.t.	12	<5	n.d.	21:79
26	**SC21**	Toluene	r.t.	12	21	50:50	65:35
27	**SC22**	Toluene	r.t.	12	54	58:42	98:2
28	**SC23**	Toluene	r.t.	12	100	68:32	99:1
29	**SC24**	Toluene	r.t.	12	100	76:24	>99:1
30	**SC25**	Toluene	r.t.	12	6	52:48	89:11
31	**SC26**	Toluene	r.t.	12	100	78:22	>99:1
32	**SC27**	Toluene	r.t.	12	43	53:47	68:32
33	**SC28**	Toluene	r.t.	12	100	76:24	>99:1
34	**SC29**	Toluene	r.t.	12	100	50:50	98:2
35	**SC30**	Toluene	r.t.	12	100	78:22	>99:1
36	**SC31**	Toluene	r.t.	12	100	77:23	>99:1

37	**SC32**	Toluene	r.t.	12	86	63:37	84:16
38	**SC33**	Toluene	r.t.	12	77	50:50	63:37
39	**SC34**	Toluene	r.t.	12	98	71:29	92:8
40	**SC35**	Toluene	r.t.	12	100	68:32	87:13
41	**SC36**	Toluene	r.t.	12	75	65:35	86:14
42	**SC37**	Toluene	r.t.	12	56	64:36	85:15
43	**C7**	Toluene	r.t.	14	100	78:22	99:1
44	**C7**	DCE	r.t.	14	100	70:30	>99:1
45	**C7**	CCl4	r.t.	14	100	75:25	96:4
46	**C7**	Benzene	r.t.	14	100	77:23	>99:1
47	**C7**	CH2Cl2	r.t.	14	100	71:29	>99:1
48	**C7**	*o-Xylene*	r.t.	14	100	77:23	>99:1
49	**C7**	*m-Xylene*	r.t.	14	100	78:22	>99:1
50	**C7**	*p-Xylene*	r.t.	14	100	78:22	99:1
51	**C7**	Mesitylene	r.t.	14	100	79:21	>99:1
52	**C7**	PhCF3	r.t.	14	100	73:27	99:1
53	**SC38**	Toluene	r.t.	12	100	76:24	>99:1
54	**SC39**	Toluene	r.t.	12	100	76:24	>99:1
55	**SC40**	Toluene	r.t.	12	22	50:50	84:16
56	**SC41**	Toluene	r.t.	12	94	64:36	94:6
57	**SC42**	Toluene	r.t.	12	<5	50:50	n.d.
58	**SC43**	Toluene	r.t.	12	100	75:25	>99:1
59	**SC44**	Toluene	r.t.	12	100	62:38	>99:1

60	**SC45**	Toluene	r.t.	12	100	59:41	96:4
61	**SC46**	Toluene	r.t.	12	100	76:24	99:1
62	**SC47**	Toluene	r.t.	12	100	75:25	97:3
63	**SC48**	Toluene	r.t.	12	100	79:21	>99:1
64	**SC49**	Toluene	r.t.	12	54	61:39	98:2
65	**SC50**	Toluene	r.t.	12	100	78:22	99:1
66	**SC51**	Toluene	r.t.	12	100	78:22	>99:1
67	**SC52**	Toluene	r.t.	12	<5	53:47	n.d.
68	**SC53**	Toluene	r.t.	12	72	58:42	97:3
69	**SC54**	Toluene	r.t.	12	<5	n.d.	n.d.
70	**SC55**	Toluene	r.t.	12	12	53:47	56:44
71	**SC56**	Toluene	r.t.	12	0	n.d.	n.d.
72	**SC57**	Toluene	r.t.	12	100	52:48	97:3
73	**SC58**	Toluene	r.t.	12	7	50:50	10:90
74	**SC59**	Toluene	r.t.	12	<5	50:50	n.d.
75	**SC60**	Toluene	r.t.	12	6	50:50	92:8
76	**SC61**	Toluene	r.t.	12	14	47:53	81:9
77	**SC62**	Toluene	r.t.	14	99	52:48	82:18
78	**SC63**	Toluene	r.t.	14	100	58:42	83:17
79	**SC64**	Toluene	r.t.	14	100	72:28	99:1
80	**SC65**	Toluene	r.t.	14	98	50:50	72:28
81	**SC66**	Toluene	r.t.	14	<5	54:46	91:9
82	**SC67**	Toluene	r.t.	14	100	65:35	99:1

83	**SC68**	Toluene	r.t.	2	99	71:29	99:1
84	**SC69**	Toluene	r.t.	2	100	71:29	99:1
85	**SC70**	Toluene	r.t.	2	100	72:28	>99:1
86	**SC71**	Toluene	r.t.	2	98	68:32	95:5
87	**SC72**	Toluene	r.t.	2	71	73:27	99:1
88	**SC73**	Toluene	r.t.	2	100	80:20	>99:1
89	**SC74**	Toluene	r.t.	2	100	79:21	>99:1
90	**SC75**	Toluene	r.t.	2	100	73:27	>99:1
91	**SC76**	Toluene	r.t.	2	100	71:29	99:1
92	**SC77**	Toluene	r.t.	14	43	50:50	68:32
93	**SC78**	Toluene	r.t.	2	100	73:27	98:2
94	**SC79**	Toluene	r.t.	2	54	64:36	54:46
95	**SC80**	Toluene	r.t.	2	54	61:39	71:29
96	**SC81**	Toluene	r.t.	2	12	53:47	64:36
97	**SC82**	Toluene	r.t.	2	20	52:48	76:24
98	**SC83**	Toluene	r.t.	2	100	44:56	80:20
99	**SC84**	Toluene	r.t.	2	7	50:50	50:50
100	**SC85**	Toluene	r.t.	14	100	76:24	99:1
101	**SC86**	Toluene	r.t.	14	100	77:23	99:1
102	**SC87**	Toluene	r.t.	14	100	75:25	96:4
103	**SC88**	Toluene	r.t.	14	100	69:31	99:1
104	**SC89**	Toluene	r.t.	14	100	66:34	99:1
105	**SC90**	Toluene	r.t.	14	100	67:33	99:1

106	**SC91**	Toluene	r.t.	14	100	63:37	>99:1
107	**SC92**	Toluene	r.t.	14	100	58:42	99:1
108	**SC93**	Toluene	r.t.	14	100	72:28	>99:1
109	**SC94**	Toluene	r.t.	14	100	56:44	93:7
110	**SC95**	Toluene	r.t.	14	100	61:39	>99:1
111	**SC96**	Toluene	r.t.	14	100	66:34	>99:1
112	**SC97**	Toluene	r.t.	14	98	72:28	97:3
113	**SC98**	Toluene	r.t.	14	100	81:19	>99:1
114	**SC99**	Toluene	r.t.	14	100	81:19	99:1
115	**SC100**	Toluene	r.t.	14	100	82:18	>99:1
116	**SC101**	Toluene	r.t.	14	98	87:13	98:2
117	**SC101**	Toluene	0	35	96	91:9	97:3
118	**SC101**	Toluene	-10	30	89	87:13	93:7
119	**SC102**	Toluene	r.t.	14	97	63:37	91:9
120	**SC103**	Toluene	r.t.	14	100	71:29	98:2
121	**SC104**	Toluene	r.t.	14	100	71:29	96:4
122	**SC105**	Toluene	r.t.	14	99	85:15	98:2
123	**SC106**	Toluene	r.t.	14	99	82:18	98:2
124	**SC107**	Toluene	r.t.	14	98	57:43	89:11
125	**SC108**	Toluene	r.t.	14	100	63:37	96:4
126	**SC109**	Toluene	r.t.	14	38	66:34	80:20
127	**C8**	Toluene	r.t.	16	100	87:13	97:3
128	**SC110**	Toluene	r.t.	14	100	83:17	98:2

129	**SC111**	Toluene	r.t.	14	100	76:24	97:3
130	**SC112**	Toluene	r.t.	14	100	87:13	98:2
131	**SC113**	Toluene	r.t.	14	98	82:18	88:12
132	**SC114**	Toluene	r.t.	14	100	82:18	99:1
133	**SC115**	Toluene	r.t.	14	26	73:27	92:8
134	**SC116**	Toluene	r.t.	16	100	82:18	97:3
135	**C8**	Toluene	0	16	93	92:8	96:4

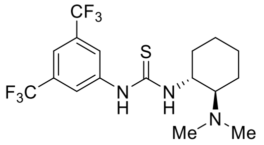
C1

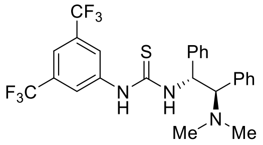
C4

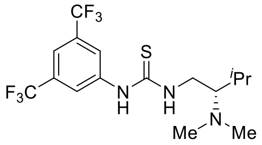
SC1

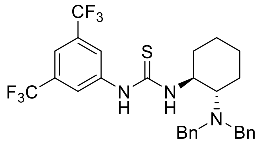
SC2

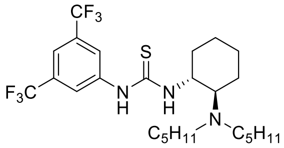
SC3

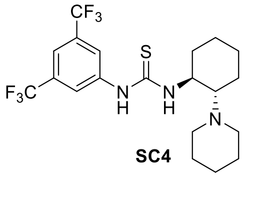
SC4

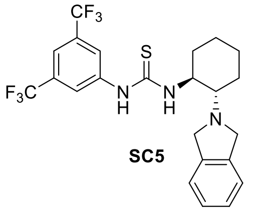
SC5

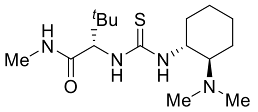
C5

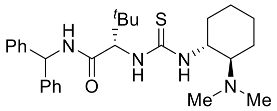
SC6

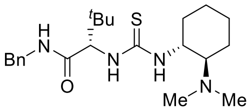
SC7

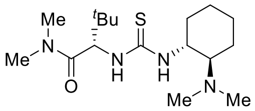
SC8

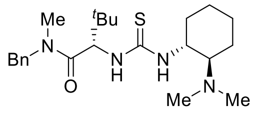
SC9

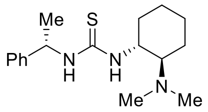
SC10

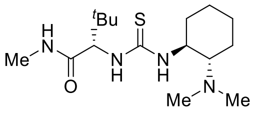
SC11

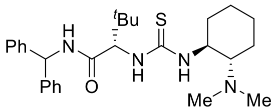
SC12

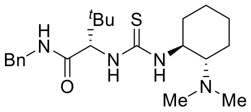
SC13

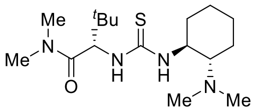
SC14

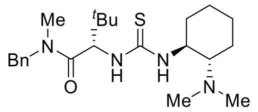
SC15

Supplementary Table 1 (cont.). Catalyst, solvent, and temperature evaluation for *trans*-/enantioselective dehydrogenation of **1b** on 2.00 μmol scale

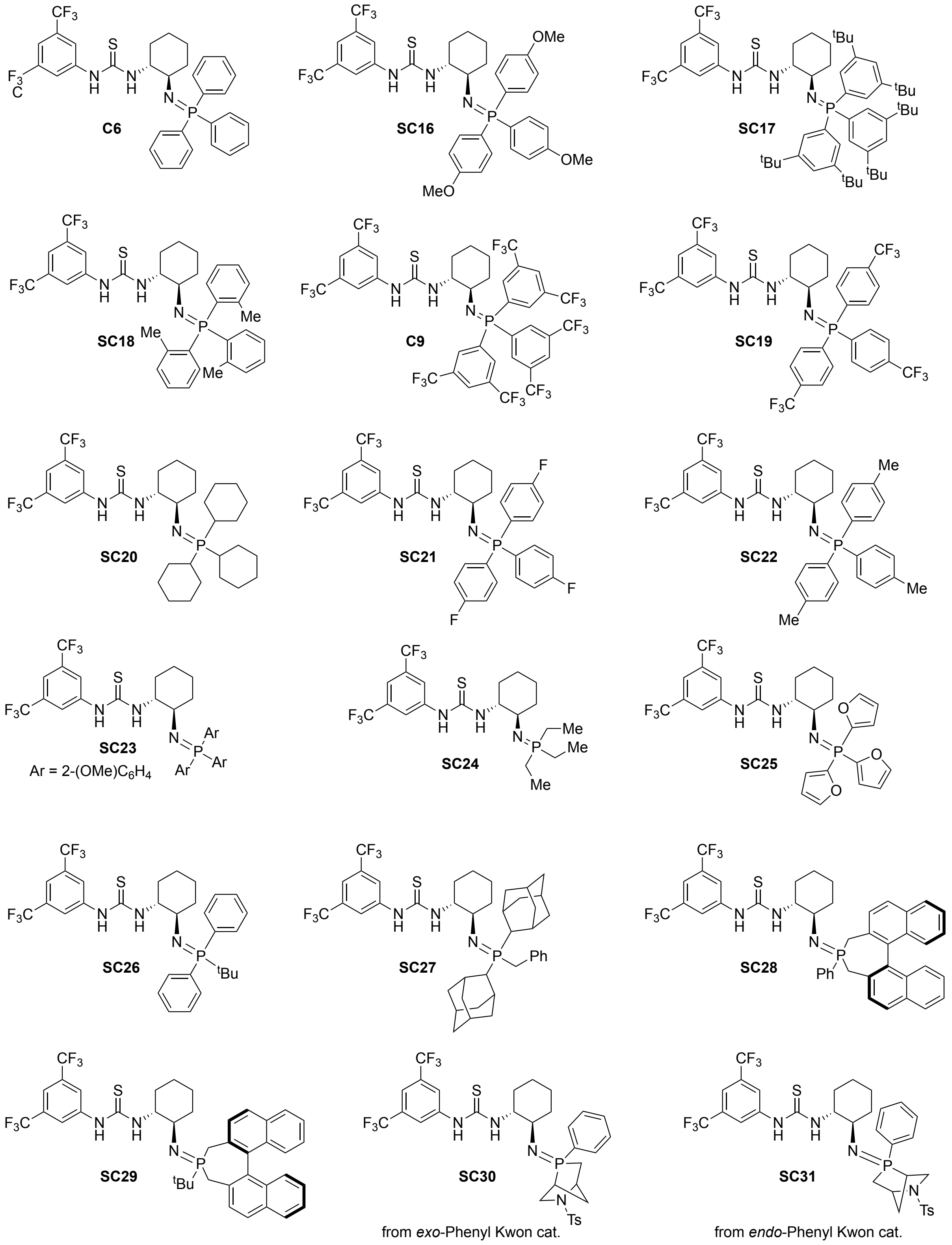

Supplementary Table 1 (cont.). Catalyst, solvent, and temperature evaluation for *trans*-/enantioselective dehydrogenation of **1b** on 2.00 μmol scale

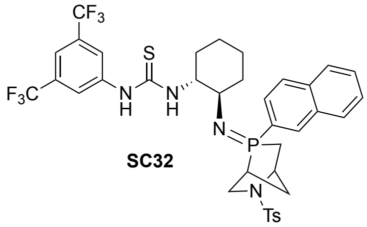

SC32

from *exo*-2-Naphthyl Kwon cat.

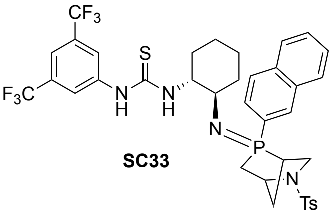

SC33

from *endo*-2-Naphthyl Kwon cat.

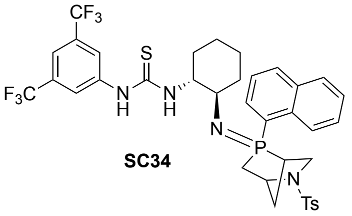

SC34

from *endo*-1-Naphthyl Kwon cat.

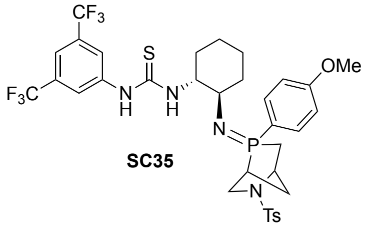

SC35

from *exo*-4-Methoxyphenyl Kwon cat.

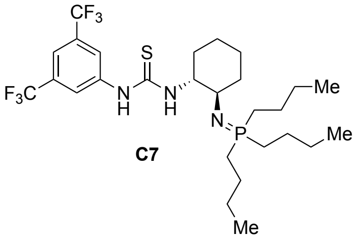

C7

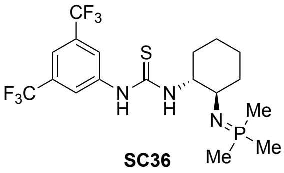

SC36

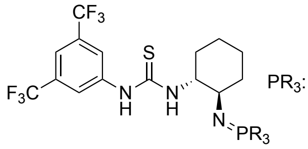

2.00 eq. of azide precursor were used

PR3:

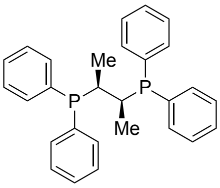

SC37
(*S*,*S*)-Chiraphos

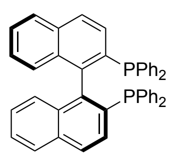

SC38
(*S*)-BINAP

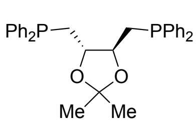

SC39
(*S*,*S*)-DIOP

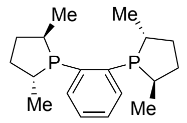

SC40
(*R*,*R*)-DuPhos

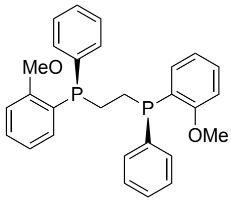

SC41
(*R*,*R*)-DIPAMP

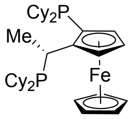

SC42

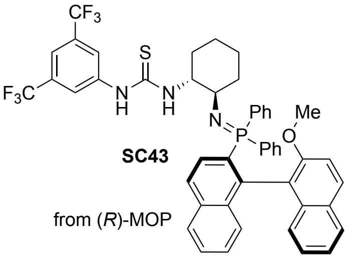

SC43

from (*R*)-MOP

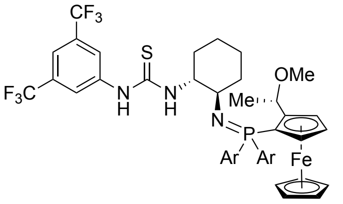

SC44 Ar = 4-MeO-3,5-$(CH_3)_2C_6H_2$
SC45 Ar = 2-Furyl

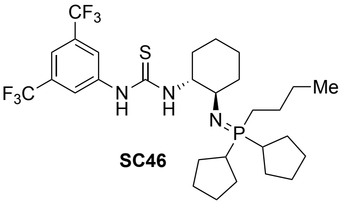

SC46

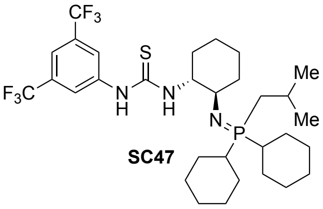

SC47

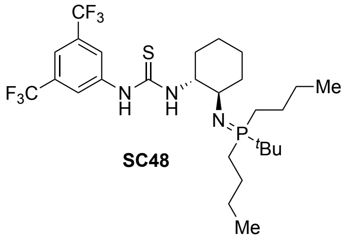

SC48

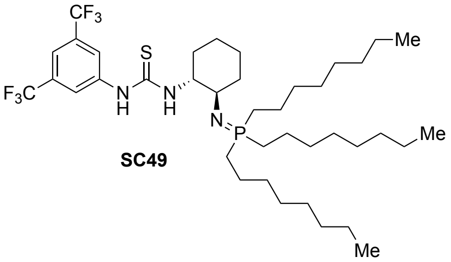

SC49

Supplementary Table 1 (cont.). Catalyst, solvent, and temperature evaluation for *trans*-/enantioselective dehydrogenation of **1b** on 2.00 μmol scale

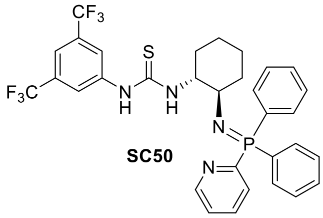

SC50

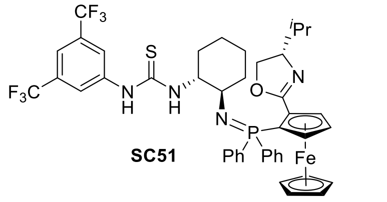

SC51

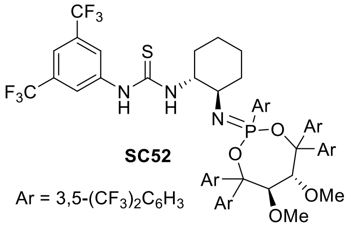

SC52

Ar = 3,5-$(CF_3)_2C_6H_3$

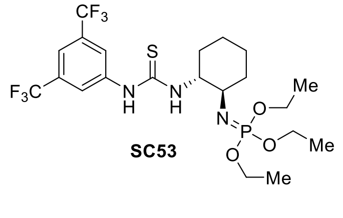

SC53

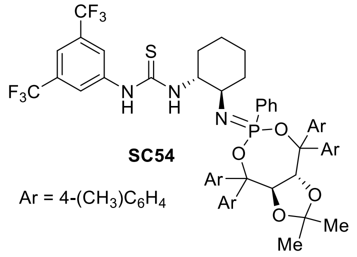

SC54

Ar = 4-$(CH_3)C_6H_4$

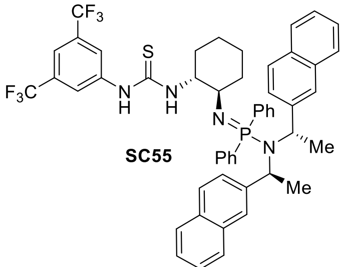

SC55

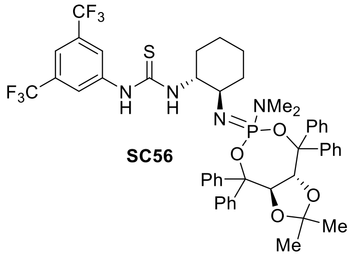

SC56

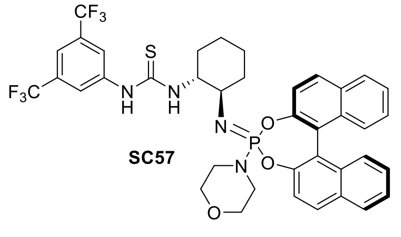

SC57

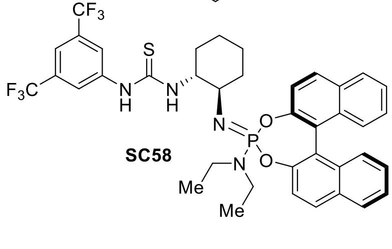

SC58

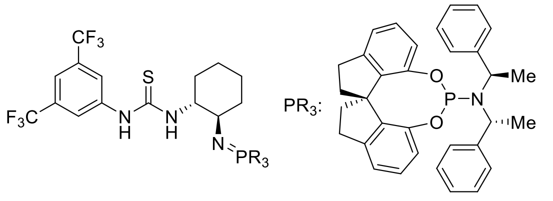

SC59
(*R*)-SIPHOS-PE

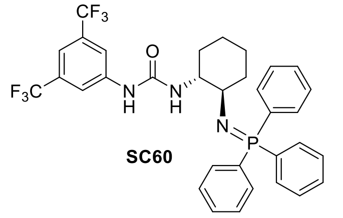

SC60

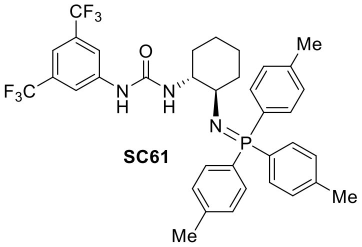

SC61

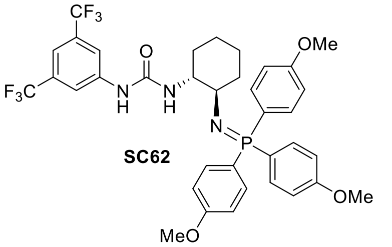

SC62

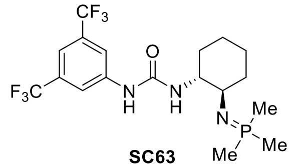

SC63

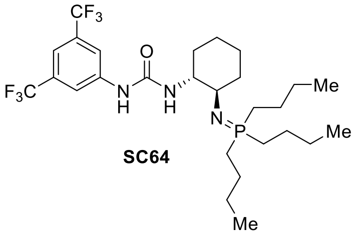

SC64

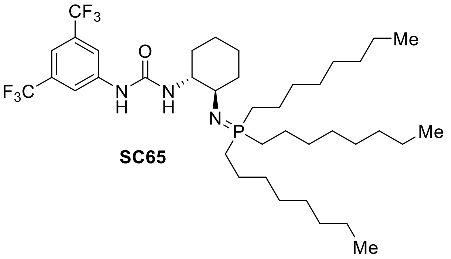

SC65

Supplementary Table 1 (cont.). Catalyst, solvent, and temperature evaluation for *trans*-/enantioselective dehydrogenation of **1b** on 2.00 μmol scale

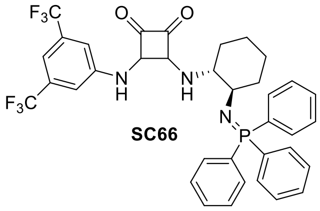

SC66

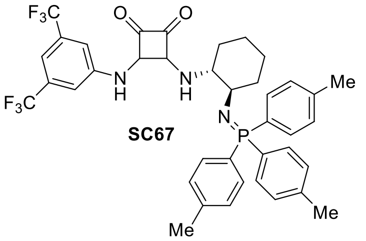

SC67

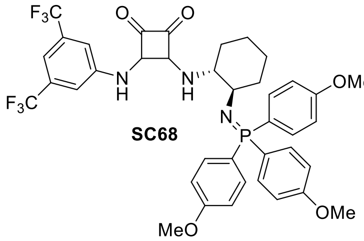

SC68

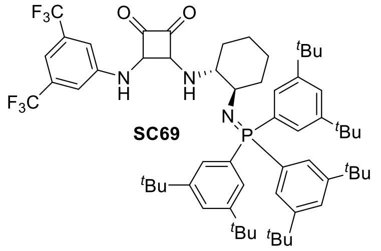

SC69

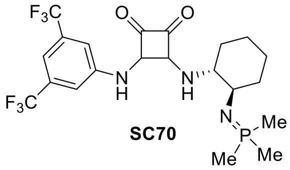

SC70

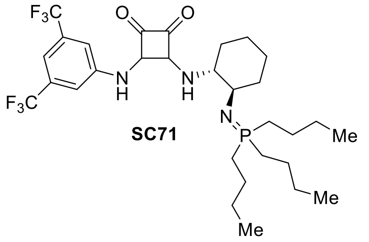

SC71

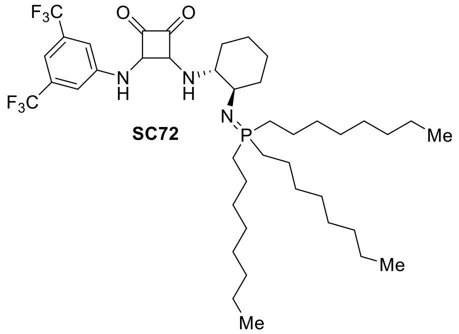

SC72

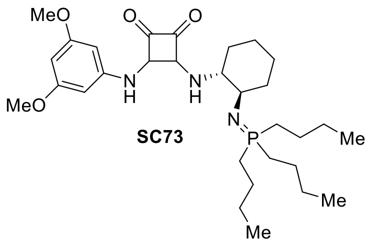

SC73

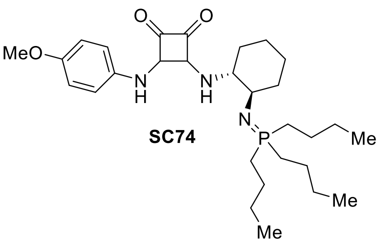

SC74

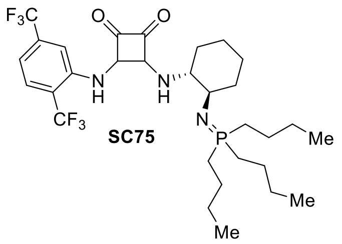

SC75

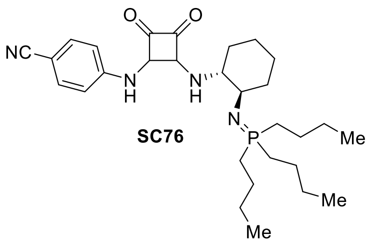

SC76

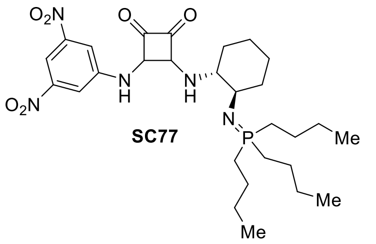

SC77

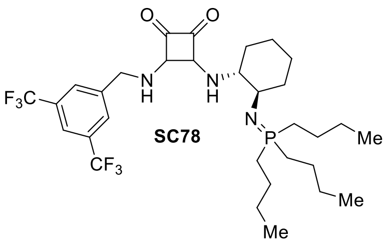

SC78

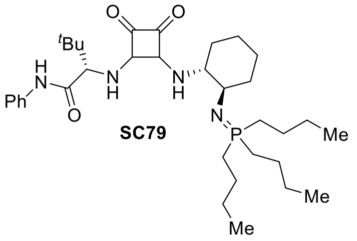

SC79

Supplementary Table 1 (cont.). Catalyst, solvent, and temperature evaluation for *trans*-/enantioselective dehydrogenation of **1b** on 2.00 μmol scale

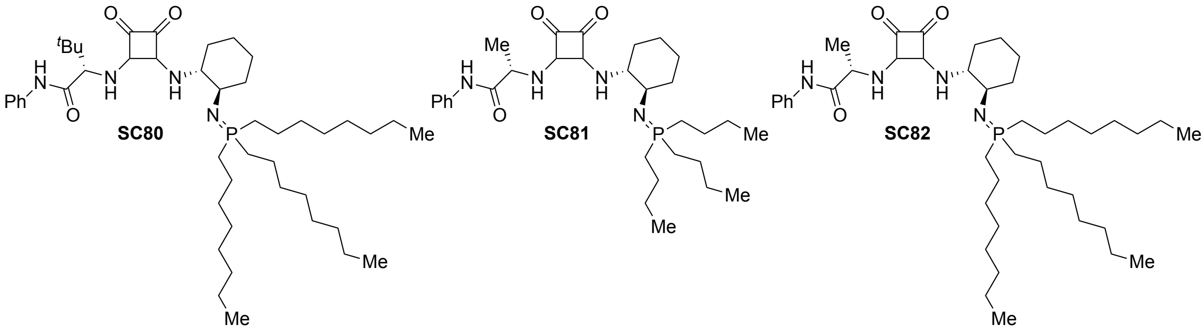

SC80 **SC81** **SC82**

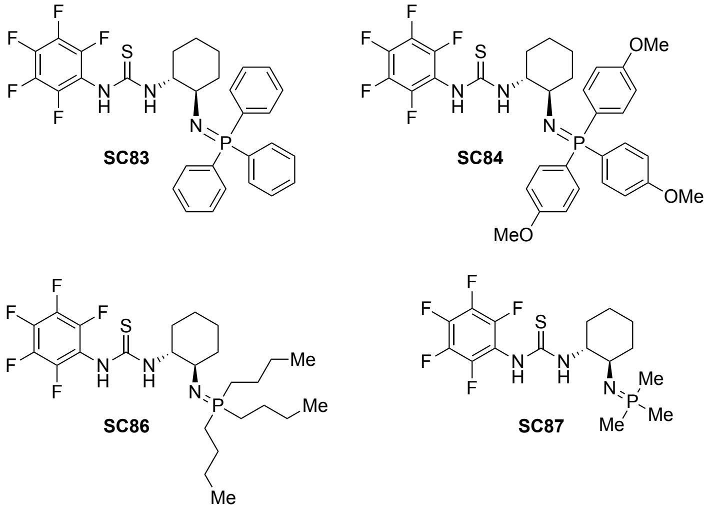

SC83 **SC84** **SC85** **SC86** **SC87**

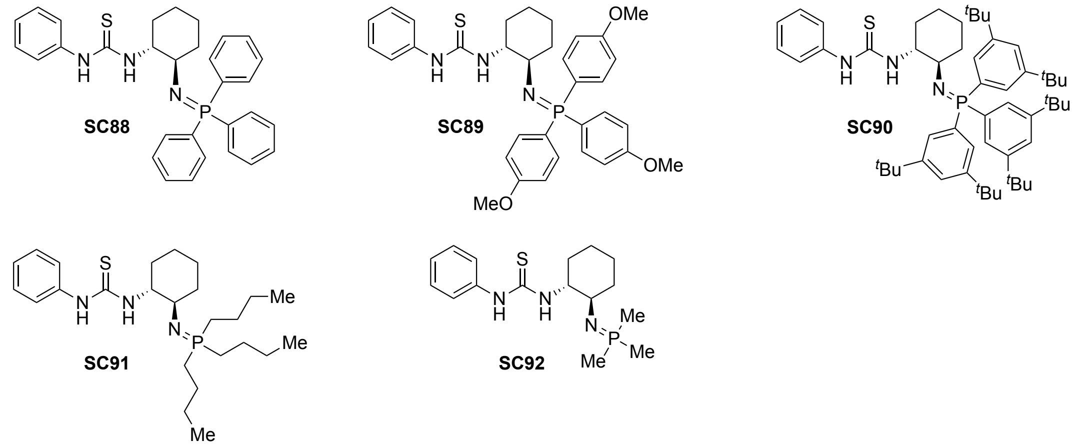

SC88 **SC89** **SC90** **SC91** **SC92**

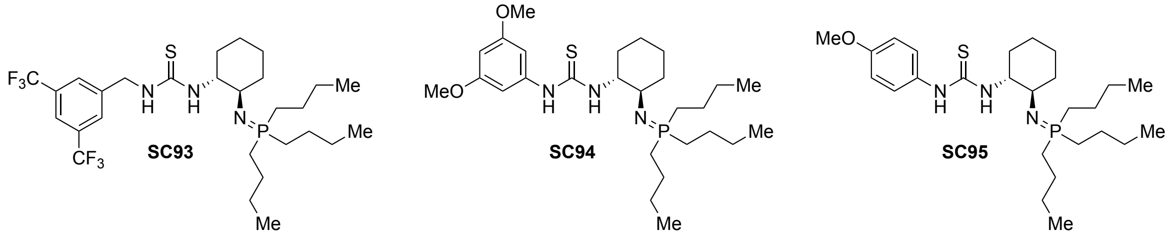

SC93 **SC94** **SC95**

Supplementary Table 1 (cont.). Catalyst, solvent, and temperature evaluation for *trans*-/enantioselective dehydrogenation of **1b** on 2.00 μmol scale

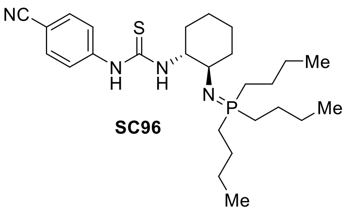

SC96

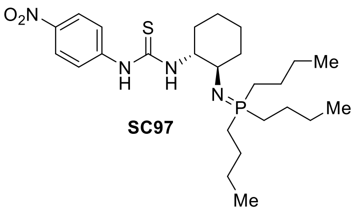

SC97

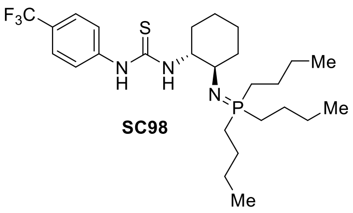

SC98

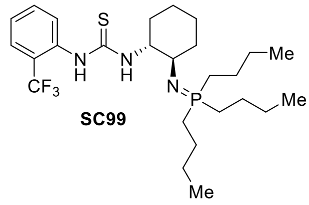

SC99

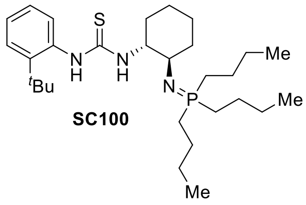

SC100

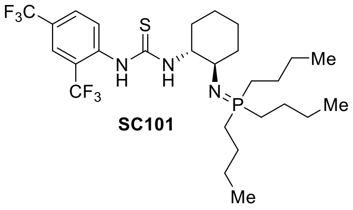

SC101

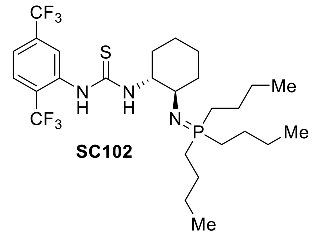

SC102

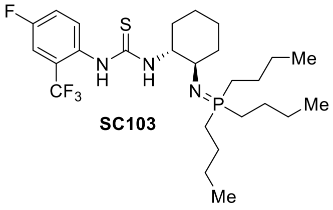

SC103

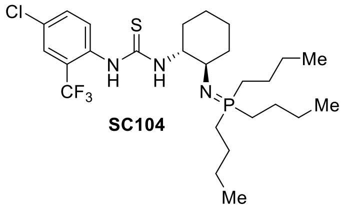

SC104

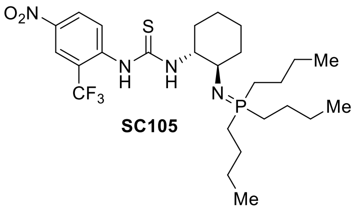

SC105

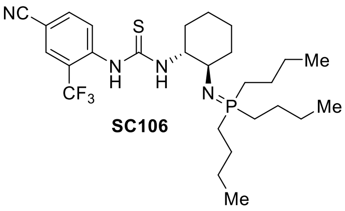

SC106

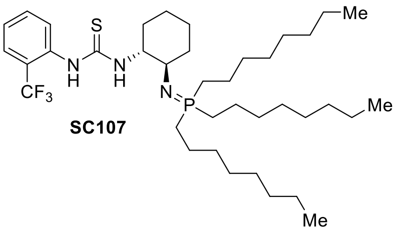

SC107

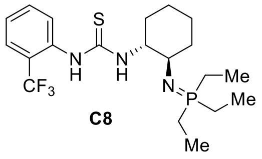

C8

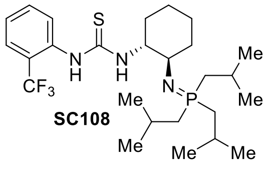

SC108

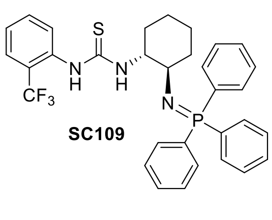

SC109

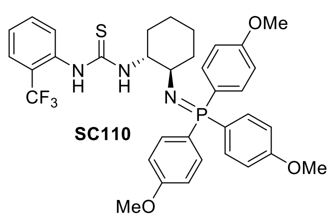

SC110

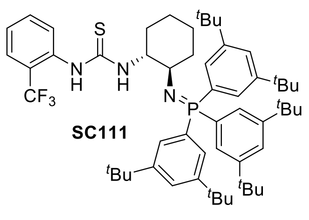

SC111

Supplementary Table 1 (cont.). Catalyst, solvent, and temperature evaluation for *trans*-/enantioselective dehydrogenation of **1b** on 2.00 μmol scale

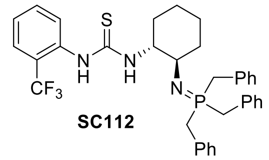

SC112

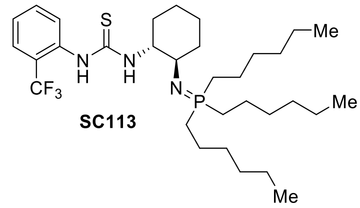

SC113

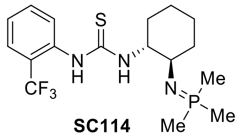

SC114

Optimisation of the reaction conditions for *trans*-/enantioselective catalyst control over pentavalent stereocentres

Final optimisation

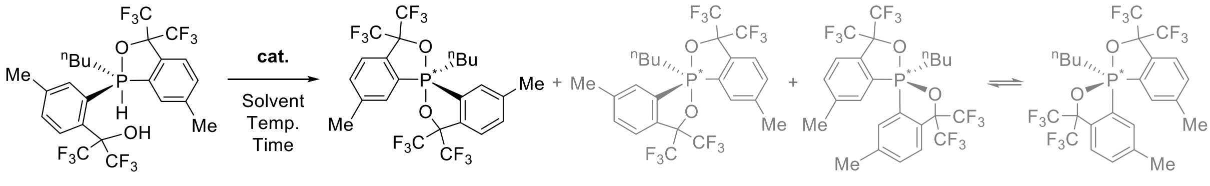

General procedure for the method optimisation

In a dry 20 mL crimp cap vial, catalyst and compound **1a** (30.1 mg, 50.0 µmol, 1.00 eq.) were dissolved in the specified solvent (5.0 mL) under an Ar atmosphere. The reaction mixture was stirred at the indicated temperature for the time indicated. The solvent was removed under a stream of nitrogen. The residue was purified by flash column chromatography (pentane/CH_2Cl_2 4:1).

Supplementary Table 2. Optimisation of the reaction conditions with **1a** on 50.0 µmol scale

Entry	Catalyst	Loading/ mol%	Solvent	Temp/°C	Time/h	Yield/%[a]	e.r.[b]	d.r.[c]
1	**C1**	20.0	Toluene	40	20	43	45:55	92:8
2	**C2**	20.0	Toluene	40	20	23	52:48	89:11
3	**C3**	20.0	Toluene	40	20	50	49:51	91:9
4	**C4**	20.0	Toluene	40	20	17	48:52	93:7
5	**C5**	20.0	Toluene	24	20	90	53:47	95:5
6	**C6**	20.0	Toluene	24	2	99	66:34	98:2
7	**C7**	20.0	Toluene	24	2	99	76:24	99:1
8	**C8**	20.0	Toluene	24	2	99	92:8	98:2
9	**C8**	20.0	CH_2Cl_2	24	2	80	89:11	97:3
10	**C8**	20.0	THF	24	2	93	92:8	96:4
11	**C8**	10.0	Toluene	24	2	99	92:8	99:1
12	**C8**	5.00	Toluene	24	2	99	92:8	99:1
**13[d]**	**C8**	**5.00**	**Toluene**	**0**	**20**	**99**	**96:4**	**99:1**

[a] Isolated yield. [b] Determined by HPLC analysis on a chiral stationary phase. [c] Determined by ^{19}F NMR analysis of the crude reaction mixture. [d] 2.5 mL toluene.

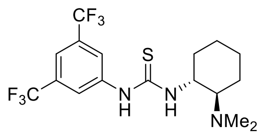

C1

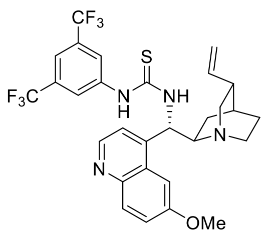

C2

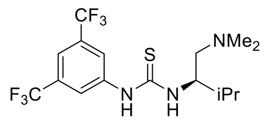

C3

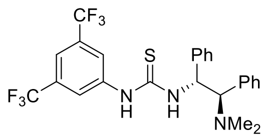

C4

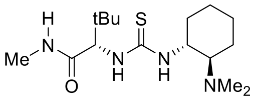

C5

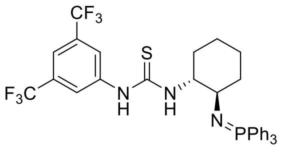

C6

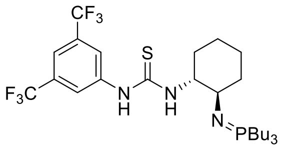

C7

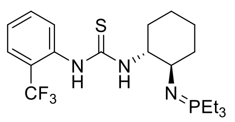

C8

Catalyst synthesis

Catalysts used in this work were purchased from commercial sources or prepared according to the literature reported procedures.[9-11] *tert*-Butyl ((1*R*,2*R*)-2-azidocyclohexyl)carbamate and *tert*-Butyl ((1*S*,2*S*)-2-azidocyclohexyl)carbamate were prepared using literature reported procedures.[10,12,13]

Representative procedure for the synthesis of bifunctional iminophosphorane catalysts

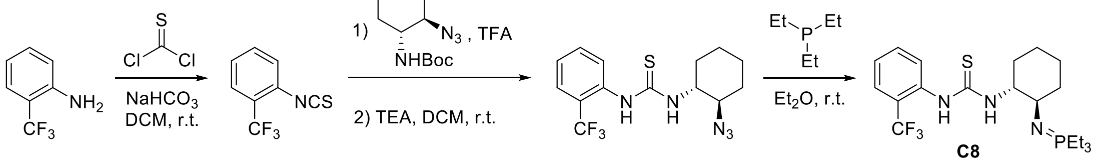

1-Isothiocyanato-2-(trifluoromethyl)benzene (S6)[9]

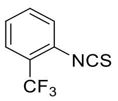

In a 100 mL round-bottom flask, a saturated solution of sodium hydrogen carbonate (20.0 mL) was added to a solution of 2-(trifluoromethyl)aniline (1.29 g, 8.00 mmol, 1.00 eq.) in CH_2Cl_2 (20 mL) at 0 °C. The mixture was stirred for 10 min, stirring was stopped, and thiophosgene (0.92 mL, 12.0 mmol, 1.50 eq.) was added to the CH_2Cl_2 layer in one portion via syringe. The resulting mixture was stirred (~500 rpm) for 1 h at r.t. The reaction mixture was transferred to a separatory funnel and the organic layer removed. The aqueous layer was extracted with CH_2Cl_2 (2 x 25 mL) and the combined organic layers dried over Na_2SO_4. The solvent was removed under reduced pressure, the residue was purified by flash column chromatography (pentane:CH_2Cl_2 100:0 → 95:5) to afford 1-isothiocyanato-2-(trifluoromethyl)benzene as a pale yellow liquid (398 mg, 1.96 mmol, 25%); R_f 0.65 (pentane); The spectral data were in accordance with those previously reported in the literature.[14]

1-((1*R*,2*R*)-2-Azidocyclohexyl)-3-(2-(trifluoromethyl)phenyl)thiourea (S7)

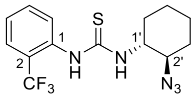

The title compound was obtained according to the modified literature procedure.[10] An ice-cooled 10 mL crimp cap vial containing *tert*-butyl ((1*R*,2*R*)-2-azidocyclohexyl)carbamate (253 mg, 1.05 mmol, 1.00 eq.) was placed behind a blast shield. TFA (1.00 mL) was added carefully dropwise and the resulting solution was stirred at r.t. for 2 h. TFA was evaporated under a stream of N_2, the residue dissolved in Et_2O (5.0 mL) and aq. 2 $molL^{-1}$ NaOH solution added until pH 14. The aqueous phase was extracted with Et_2O (3 x 10 mL) and the combined organic layers were dried over Na_2SO_4 and concentrated **(CAUTION: solvents were evaporated under a stream of N_2 behind a blast-shield)**. The residue was dissolved in CH_2Cl_2 (1.0 mL) and **S6** (214 mg, 1.05 mmol, 1.00 eq.) was added. The resulting solution was stirred at r.t. for 20 h. The solution was loaded onto a silica gel column and chromatographed (CH_2Cl_2) to afford **S7** as a white solid (293 mg, 0.85 mmol, 81%, m.p. = 114 – 115 °C (decomposition)); R_f 0.40 (CH_2Cl_2); $[\alpha]_D^{25}$ – 84.8 (*c* 0.1, $CHCl_3$); IR $\tilde{\nu}_{max}$ (neat): 3230m, 3049w, 2938m, 2861w, 2360w, 2095s, 1532s, 1456m, 1317s, 1261m, 1165s, 1125s, 1058m, 911w, 868w, 719w, 648s; ^1H NMR (500 MHz, $CDCl_3$) δ 7.74 (d, $^3J_{H-H}$ = 7.9 Hz, 1H, C3*H* overlapping with br. s, 1H, C1N*H*), 7.63 (t, $^3J_{H-H}$ = 7.5, 1H,

C5*H*)), 7.60 – 7.56 (m, 1H, C6*H*)), 7.43 (t, $^3J_{H-H}$ = 7.7 Hz, 1H, C4*H*), 5.82 (br. s, 1H, C1'N*H*), 4.17 (br. s, 1H, C1'*H*), 3.27 – 3.07 (m, 1H, Cy-*H*), 2.43 – 2.20 (m, 1H, Cy-C*H*), 2.09 (dd, *J* = 12.5, 4.0 Hz, 1H, Cy-C*H*), 1.81 (dtd, *J* = 13.4, 3.6, 1.9 Hz, 1H, Cy-C*H*), 1.74 – 1.63 (m, 1H, Cy-C*H*), 1.51 (tdd, *J* = 12.9, 11.1, 3.8 Hz, 1H, Cy-C*H*), 1.37 (qt, *J* = 13.4, 3.5 Hz, 1H, Cy-C*H*), 1.31 – 1.19 (m, 1H, Cy-C*H*), 1.14 (d, *J* = 12.4 Hz, 1H, Cy-C*H*); $^{13}C\{^1H\}$ NMR (126 MHz, $CDCl_3$) δ 181.9 (*C*=S), 134.7 (*C*1), 133.3 (*C*5), 129.5 (*C*6), 127.8 (*C*4), 127.5 (*C*3), 126.6 (*C*2) 123.3 (q, $^1J_{C-F}$ = 273.1 Hz, CF_3), 64.1 (Cy-*C*), 58.5 (Cy-*C*), 31.7 (Cy-*C*), 30.7 (Cy-*C*), 24.1 (Cy-*C*), 24.1 (Cy-*C*); $^{19}F\{^1H\}$ NMR (376 MHz, $CDCl_3$) δ −61.6; HRMS (ESI$^+$): *m/z* calcd. 344.1151 for ($[M+H]^+$), found 344.1158.

1-((1*R*,2*R*)-2-((Triethyl-λ^5-phosphaneylidene)amino)cyclohexyl)-3-(2-(trifluoromethyl)-phenyl)thiourea (C8)

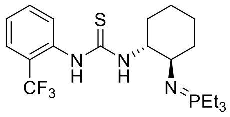

The title compound was obtained according to the modified literature procedure.[11] In a 10 mL crimp cap vial, triethylphosphine (90.0 μL, 0.61 mmol, 1.00 eq.) was added under an Ar atmosphere to a solution of azide **S7** (209 mg, 0.61 mmol, 1.00 eq.) in dry THF (4.8 mL) at r.t. The mixture was stirred at r.t. for 24 h and the solvent was removed under a stream of N_2. Pentane (2.0 mL) was added and the mixture was stirred vigorously (~1000 rpm) for 2 h. The solvent was decanted from the resultant suspension, the solid was washed with cold pentane (2 x 1.0 mL). The solvent was removed under a stream of N_2, the solid was dried in a high vacuum (~ 0.5 mbar) for 4 h at r.t, flushed with Ar and stored in a sealed crimp cap vial at −20 °C. The product thus obtained (259 mg, 0.60 mmol, 98%, white solid, m.p = 105 – 106 °C (decomposition)) was used in the catalytic reactions without further purification; $[\alpha]_D^{25}$ – 67.9 (*c* 0.1, $CHCl_3$); IR $\tilde{\nu}_{max}$ (neat): 3664w, 3422w, 2934m, 2887m, 2860m, 2361m, 2341m, 1691w, 1599m, 1506s, 1454s, 1317s, 1250m, 1162s, 1118s, 1051s, 912w, 848w, 753s, 647m; ^1H NMR (500 MHz, $CDCl_3$) δ 8.04 (br. s, 1H), 7.47 (d, $^3J_{H-H}$ = 7.4 Hz, 1H), 7.37 (t, $^3J_{H-H}$ = 7.8 Hz, 1H), 6.89 (t, $^3J_{H-H}$ = 7.6 Hz, 1H), 3.89 (br. s, 1H), 3.77 – 3.70 (m, 1H), 2.67 – 2.54 (m, 1H), 2.16 – 2.05 (m, 1H), 1.92 – 1.64 (m, 9H), 1.56 – 1.40 (m, 1H), 1.40 – 0.93 (m, 13H); $^{13}C\{^1H\}$ NMR (126 MHz, $CDCl_3$) δ 181.9, 149.8, 131.2, 126.2, 125.5, 125.0 (q, $^1J_{C-F}$ = 272.6 Hz, CF_3), 122.8, 120.2, 68.1, 60.9 (d, *J* = 5.1 Hz), 57.9, 36.1, 32.9, 25.3 (d, $^1J_{C-P}$ = 101.8 Hz, 3C, P(CH_2CH_3)$_3$), 15.3 (d, J_{C-P} = 60.7 Hz), 5.3; $^{19}F\{^1H\}$ NMR (376 MHz, $CDCl_3$) δ −60.2; $^{31}P\{^1H\}$ NMR (162 MHz, $CDCl_3$) δ 54.7; HRMS (ESI$^+$): *m/z* calcd. 434.2001 for ($[M+H]^+$), found 434.2008.

General procedure for catalyst control over pentavalent stereocentres

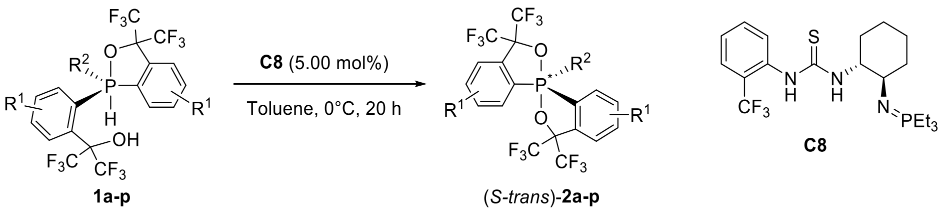

General Procedure G: Enantio- and *trans*-diastereoselective catalyst control over pentavalent stereocentres

To a mixture of the hydridophosphorane substrate **1a-p** (100 µmol, 1.00 eq.) and catalyst **C8** (2.17 mg, 5.00 µmol, 5.00 mol%) in a dried 20 mL crimp cap vial under an Ar atmosphere at 0 °C was added toluene (5.0 mL). The mixture was stirred at 0 °C for 20 h and the solvent removed under reduced pressure at 10 – 20 °C. The d.r. was determined by ^{19}F NMR of the residue, before it was purified by silica gel flash column chromatography (230 – 400 mesh) to isolate the desired product. The e.r. of the isolated product was measured by HPLC on a chiral stationary phase.

Synthesis of the racemic reference material

In a 1.5 mL crimp cap vial, triethylamine (5.00 µL, 35.6 µmol, 18.0 eq.) was added to a solution of **1** (2.00 µmol, 1.00 eq.) in THF (0.40 mL) under an ambient atmosphere. The mixture was stirred at 70 °C for 16 h. The volatiles were removed under a stream of nitrogen, the residue was dried under reduced pressure (~ 0.5 mbar), and redissolved in heptane (1.3 mL). The resulting solution was analysed by HPLC on a chiral stationary phase.

[*TBPY*-5-11'-*A*]-1-Butyl-6,6'-dimethyl-3,3,3',3'-tetrakis(trifluoromethyl)-3*H*,3'*H*-1λ^5,1'-spirobi[benzo[*c*][2,1]oxaphosphole] ((*S-trans*)-2a)

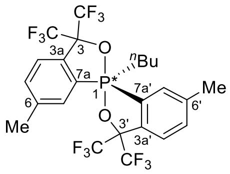

Prepared according to the general procedure **G** using P-H (apical) phosphorane **1a** (60.2 mg, 100 µmol, 1.00 eq.). The crude material was purified by column chromatography on silica gel (petroleum ether) to provide the title compound as white solid (56.0 mg, 93.0 µmol, 93%, 96:4 e.r., 99:1 d.r., m.p. = 91 – 92 °C). R_f 0.60 (petroleum ether); $[\alpha]_D^{25}$ – 69.0 (*c* 0.1, $CHCl_3$); IR $\tilde{\nu}_{max}$ (neat): 3708w, 2970m, 2867w, 2361m, 1422w, 1264m, 1204m, 1147w, 1054m, 1013m, 9744m, 896w, 828w, 732s; ^{1}H NMR (500 MHz, $CDCl_3$) δ 8.19 (d, $^3J_{H-P}$ = 11.5 Hz, 2H, C7*H* and C7'*H*), 7.63 – 7.56 (m, 2H, C4*H* and C4'*H*), 7.48 (d, $^3J_{H-H}$ = 7.9 Hz, 2H, C5*H* and C5'*H*), 2.48 (s, 6H, C6CH_3 and C6'CH_3), 2.34 (dtd, *J* = 17.1, 12.5, 5.1 Hz, 1H, ^{n}Bu-C*H*), 2.19 (qd, *J* = 12.2, 3.8 Hz, 1H, ^{n}Bu-C*H*), 1.83 – 1.72 (m, 1H, ^{n}Bu-C*H*), 1.30 – 1.19 (m, 3H, ^{n}Bu-C*H*), 0.81 (t, $^3J_{H-H}$ = 7.2 Hz, 3H, ^{n}Bu-CH_3); ^{13}C{^{1}H} NMR (126 MHz, $CDCl_3$) δ 141.7 (d, $^3J_{C-P}$ = 13.9 Hz, 2C, *C*6 and *C*6'), 137.7 (d, $^2J_{C-P}$ = 9.8 Hz, 2C, *C*7 and *C*7'), 134.6 (d, $^4J_{C-P}$ = 3.4 Hz, 2C, *C*5 and *C*5'), 133.7 (d, $^2J_{C-P}$ = 19.1 Hz, 2C, *C*3a and *C*3a'), 130.5 (d, $^1J_{C-P}$ = 158.0 Hz, 2C, *C*7a and *C*7a'), 124.6 (d, $^3J_{C-P}$ = 16.0 Hz, 2C, *C*4 and *C*4'), 122.9 (q, $^1J_{C-F}$ = 286.7 Hz, 2C, C3*C*F_3 and C3'*C*F_3), 122.7 (q,

$^{1}J_{C-F}$ = 289.06 Hz, 2C, C3*C*F$_3$ and C3'*C*F$_3$), 81.3 (hept, $^{2}J_{C-F}$ = 31.0 Hz, 2C, *C*3 and *C*3'), 39.3 (d, $^{1}J_{C-P}$ = 117.2 Hz, P*C*H$_2$(CH$_2$)$_2$CH$_3$), 25.5 (d, J_{C-P} = 6.2 Hz, ^{n}Bu-*C*), 23.8 (d, J_{C-P} = 23.8 Hz, ^{n}Bu-*C*), 21.5 (d, $^{4}J_{C-P}$ = 1.6 Hz, C6*C*H$_3$ and C6'*C*H$_3$), 13.3 (d, $^{4}J_{C-P}$ = 1.3 Hz, ^{n}Bu-*C*H$_3$); ^{19}F{^{1}H} NMR (471 MHz, CDCl$_3$) δ −74.7 (q, $^{4}J_{F-F}$ = 9.3 Hz, 6F, C3C*F*$_3$ and C3'C*F*$_3$), −75.1 (q, $^{4}J_{F-F}$ = 9.5 Hz, 6F, C3C*F*$_3$ and C3'C*F*$_3$); ^{31}P{^{1}H} NMR (202 MHz, CDCl$_3$) δ −19.7. HRMS (ESI^{+}): *m/z* calcd. 601.1160 for ([M+H]$^{+}$), found 601.1158; The e.r was determined by HPLC using a *Chiralcel IB-N3* analytical column (0.50 mL min^{-1}, 100% heptane, 5 °C): t_{minor} = 7.8 min and t_{major} = 8.4 min. Assignments of HPLC peaks are in accord with their UV spectra.

[*TBPY*-5-11'-*A*]-1-Butyl-3,3,3',3'-tetrakis(trifluoromethyl)-3*H*,3'*H*-1λ^5,1'-spirobi[benzo[*c*][2,1]oxaphosphole] ((*S-trans*)-2b)

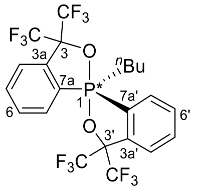

Prepared according to the general procedure **G** using P-H (apical) phosphorane **1b** (60.2 mg, 100 μmol, 1.00 eq.). The crude material was purified by column chromatography on silica gel (pentane) to provide the title compound as white solid (46.0 mg, 80.4 μmol, 80%, 90:10 e.r., 99:1 d.r.); R$_f$ 0.80 (pentane); $[\alpha]_{D}^{24}$ – 29.2 (*c* 0.1, CHCl$_3$); The spectral data were in accordance with those previously reported in the literature.[1] The e.r was determined by HPLC using a *Chiralcel IB-N3* analytical column (0.50 mL min^{-1}, 100% heptane, 5 °C): t_{minor} = 8.3 min and t_{major} = 8.8 min. Assignments of HPLC peaks are in accord with their UV spectra.

[*TBPY*-5-11'-*A*]-1-Butyl-5,5'-dimethyl-3,3,3',3'-tetrakis(trifluoromethyl)-3*H*,3'*H*-1λ^5,1'-spirobi[benzo[*c*][2,1]oxaphosphole] ((*S-trans*)-2c)

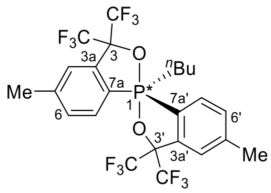

Prepared according to the general procedure **G** using P-H (apical) phosphorane **1c** (60.2 mg, 100 μmol, 1.00 eq.). The crude material was purified by column chromatography on silica gel (petroleum ether) to provide the title compound as white crystals (50.0 mg, 83.0 μmol, 83%, 84:16 e.r., 95:5 d.r., m.p. = 88 – 89 °C). R$_f$ 0.60 (petroleum ether); $[\alpha]_{D}^{25}$ –28.2 (*c* 0.1, CHCl$_3$); IR $\tilde{\nu}_{max}$ (neat): 3323s, 2944m, 2831m, 2361m, 1650w, 1452m, 1417m, 1114w, 1021s, 647m; ^{1}H NMR (500 MHz, CDCl$_3$) δ 8.34 – 8.17 (m, 2H, C7*H* and C7'*H*), 7.50 (s, 2H, C4*H* and C4'*H*), 7.48 – 7.43 (m, 2H, C6*H* and C6'*H*), 2.48 (s, 6H, C5C*H*$_3$ and C5'C*H*$_3$), 2.37 – 2.24 (m, 1H, ^{n}Bu-C*H*), 2.17 (qdd, *J* = 12.4, 4.2, 1.5 Hz, 1H, ^{n}Bu-C*H*), 1.89 – 1.67 (m, 1H, ^{n}Bu-C*H*), 1.32 – 1.13 (m, 3H, ^{n}Bu-C*H*), 0.80 (t, $^{3}J_{H-H}$ = 6.4 Hz, 3H, ^{n}Bu-C*H*$_3$); ^{13}C{^{1}H} NMR (126 MHz, CDCl$_3$) δ 144.7 (d, $^{4}J_{C-P}$ = 3.4 Hz, 2C, *C*5 and *C*5'), 136.9 (d, $^{2}J_{C-P}$ = 10.2 Hz, 2C, *C*7 and *C*7'), 136.7 (d, $^{2}J_{C-P}$ = 19.7 Hz, 2C, *C*3a and *C*3a'), 132.5 (d, $^{3}J_{C-P}$ = 14.3 Hz, 2C, *C*6 and *C*6'), 127.4 (d, $^{1}J_{C-P}$ = 161.9 Hz, 2C, *C7a* and *C*7a'), 125.4 (d, $^{3}J_{C-P}$ = 15.6 Hz, 2C, *C*4 and *C*4'), 122.9 (q, $^{1}J_{C-F}$ = 285.6 Hz, 2C, C3*C*F$_3$ and C3'*C*F$_3$), 122.7 (q, $^{1}J_{C-F}$ = 289.1Hz, 2C, C3*C*F$_3$ and C3'*C*F$_3$), 81.3 (hept, $^{2}J_{C-F}$ = 30.2 Hz, 2C, *C*3 and *C*3'), 39.3 (d, $^{1}J_{C-P}$ = 117.4 Hz, ^{n}Bu-*C*H$_2$(CH$_2$)$_2$CH$_3$), 25.5 (d, J_{C-P} = 6.2 Hz, ^{n}Bu-*C*), 23.8

(d, $J_{C\text{-}P}$ = 23.7 Hz, ^{n}Bu-*C*), 21.7 (d, $^5J_{C\text{-}P}$ = 1.7 Hz, 2C, C5*C*H3 and C5'*C*H3), 13.4 (d, $^4J_{C\text{-}P}$ = 1.2 Hz, ^{n}Bu-*C*H3); ^{19}F{^{1}H} NMR (471 MHz, CDCl3) δ −74.7 (dd, $J_{F\text{-}F}$ = 10.9, 7.5 Hz, 6F, C3C*F*3 and C3'C*F*3), −74.9 (q, $^4J_{F\text{-}F}$ = 9.4 Hz, 6F, C3C*F*3 and C3'C*F*3); ^{31}P{^{1}H} NMR (202 MHz, CDCl3) δ −20.0; HRMS (ESI$^+$): *m/z* calcd. 601.1160 for ([M+H]$^+$), found 601.1151; The e.r was determined by HPLC using a *Chiralcel IB-N3* analytical column (0.50 mL min^{-1}, 100% heptane, 5 °C): t_{major} = 7.8 min and t_{minor} = 8.0 min. Assignments of HPLC peaks are in accord with their UV spectra.

[*TBPY*-5-11'-*A*]-1-Butyl-5,5'-dimethoxy-3,3,3',3'-tetrakis(trifluoromethyl)-3*H*,3'*H*-1λ^5,1'-spirobi[benzo[*c*][2,1]oxaphosphole] ((*S*-*trans*)-2d)

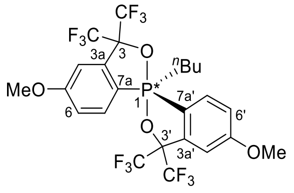

Prepared according to the general procedure **G** using P-H (apical) phosphorane **1d** (63.4 mg, 100 µmol, 1.00 eq.). The crude material was purified by column chromatography on silica gel (petroleum ether/EtOAc 20:1) to provide the title compound as white crystals (52.0 mg, 82.0 µmol, 82%, 84:16 e.r., 99:1 d.r., m.p. = 92 – 93 °C). R_f 0.60 (petroleum ether/EtOAc 20:1); $[\alpha]_D^{25}$ – 15.5 (*c* 0.1, CHCl3); IR $\tilde{\nu}_{max}$ (neat): 3306s, 2943m, 2831m, 2360m, 1451m, 1417m, 1114w, 1021s, 678m, 646m; ^{1}H NMR (500 MHz, CDCl3) δ 8.27 (t, *J* = 9.7 Hz, 2H, C7*H* and C7'*H*), 7.17 (dd, *J* = 7.5, 4.0 Hz, 4H, C4*H,* C4'*H,* C6*H* and C6'*H*), 3.90 (s, 6H, C5OC*H*3 and C5'OC*H*3), 2.29 (dtd, *J* = 17.0, 12.4, 5.1 Hz, 1H, ^{n}Bu-C*H*), 2.17 (qd, *J* = 12.1, 3.8 Hz, 1H, ^{n}Bu-C*H*), 1.83 – 1.69 (m, 1H, ^{n}Bu-C*H*), 1.25 (dq, *J* = 25.9, 6.2 Hz, 3H, ^{n}Bu-C*H*), 0.81 (t, $^3J_{H\text{-}H}$ = 7.2 Hz, 3H, ^{n}Bu-C*H*3); ^{13}C{^{1}H} NMR (126 MHz, CDCl3) δ 164.0 (d, $^4J_{C\text{-}P}$ = 3.4 Hz, 2C, *C*5 and *C*5'), 138.6 (d, $^2J_{C\text{-}P}$ = 20.9 Hz, 2C, *C*3a and *C*3a'), 138.4 (d, $^2J_{C\text{-}P}$ = 11.4 Hz, 2C, *C*7 and *C*7'), 122.8 (q, $^1J_{C\text{-}F}$ = 286.5 Hz, 2C, C3*C*F3 and C3'*C*F3), 122.7 (q, $^1J_{C\text{-}F}$ = 287.6 Hz, 2C, C3*C*F3 and C3'*C*F3), 121.1 (d, $^1J_{C\text{-}P}$ = 167.7 Hz, 2C, *C*7a and *C*7a'), 117.4 (d, $^3J_{C\text{-}P}$ = 15.1 Hz, 2C, *C*6 and *C*6'), 110.2 (d, $^3J_{C\text{-}P}$ = 16.4 Hz, 2C, *C*4 and *C*4'), 81.2 (m, 2C, *C*3 and *C*3'), 55.8 (2C, C5O*C*H3 and C5'O*C*H3), 39.4 (d, $^1J_{C\text{-}P}$ = 118.6 Hz, ^{n}Bu-*C*H2(CH2)2CH3), 25.6 (d, $J_{C\text{-}P}$ = 6.1 Hz, ^{n}Bu-*C*), 23.8 (d, $J_{C\text{-}P}$ = 23.7 Hz, ^{n}Bu-*C*), 13.4 (d, $J_{C\text{-}P}$ = 1.2 Hz, ^{n}Bu-*C*); ^{19}F{^{1}H} NMR (471 MHz, CDCl3) δ −74.7 (q, $^4J_{F\text{-}F}$ = 9.4 Hz, 6F, C3C*F*3 and C3'C*F*3), −75.0 (q, $^4J_{F\text{-}F}$ = 9.4 Hz, 6F, C3C*F*3 and C3'C*F*3); ^{31}P{^{1}H} NMR (202 MHz, CDCl3) δ −21.1; HRMS (ESI$^+$): *m/z* calcd. 633.1059 for ([M+H]$^+$), found 633.1063; The e.r. was determined by HPLC using a *Chiralcel IB-N3* analytical column (0.25 mL min^{-1}, 100% heptane, 5 °C): t_{major} = 21.5 min and t_{minor} = 22.3 min. Assignments of HPLC peaks are in accord with their UV spectra.

[*TBPY*-5-11'-*A*]-1-Butyl-5,5'-dimethoxy-6,6'-dimethyl-3,3,3',3'-tetrakis(trifluorome-thyl)-3*H*,3'*H*-1λ⁵,1'-spirobi[benzo[*c*][2,1]oxaphosphole] ((*S-trans*)-2e)

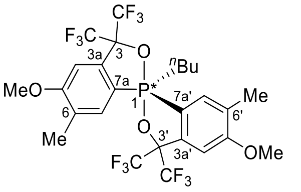

Prepared according to the general procedure **G** using P-H (apical) phosphorane **1e** (66.2 mg, 100 µmol, 1.00 eq.). The crude material was purified by column chromatography on silica gel (petroleum ether/EtOAc 20:1) to provide the title compound as white crystals (51.0 mg, 77.0 µmol, 77%, 88:12 e.r., 99:1 d.r., m.p. = 117 – 118 °C). R_f 0.60 (petroleum ether/EtOAc 20:1); $[\alpha]_D^{25}$ – 15.6 (*c* 0.1, $CHCl_3$); IR $\tilde{\nu}_{max}$ (neat): 3304s, 2943m, 2831m, 2361m, 1452m, 1417m, 1115w, 1022s, 648m; ^{1}H NMR (500 MHz, $CDCl_3$) δ 8.08 (d, $^3J_{H\text{-}P}$ = 10.3 Hz, 2H, C7*H* and C7’*H*), 7.04 (s, 2H, C4*H* and C4’*H*), 3.91 (s, 6H, C5OCH_3 and C5’OCH_3), 2.30 (s, 6H, C6CH_3 and C6’CH_3), 2.28 – 2.21 (m, 1H, ^{n}Bu-C*H*), 2.16 (qd, *J* = 12.3, 3.9 Hz, 1H, ^{n}Bu-C*H*), 1.81 – 1.69 (m, 1H, ^{n}Bu-C*H*), 1.28 – 1.18 (m, 3H, ^{n}Bu-C*H*), 0.81 (t, $^3J_{H\text{-}H}$ = 7.1 Hz, 3H, ^{n}Bu-CH_3); ^{13}C{^{1}H} NMR (126 MHz, $CDCl_3$) δ 162.1 (d, $^4J_{C\text{-}P}$ = 3.6 Hz, 2C, *C*5 and *C*5’), 138.2 (d, $^2J_{C\text{-}P}$ = 11.2 Hz, 2C, *C*7 and *C*7’), 136.3 (d, $^2J_{C\text{-}P}$ = 20.4 Hz, 2C, *C*3a and *C*3a’), 130.6 (d, $^3J_{C\text{-}P}$ = 14.6 Hz, 2C, *C*6 and *C*6’), 123.0 (q, $^1J_{C\text{-}F}$ = 287.1 Hz, 2C, C3*C*F_3 and C3’*C*F_3), 122.8 (q, $^1J_{C\text{-}F}$ = 291.2 Hz, 2C, C3*C*F_3 and C3’*C*F_3), 120.7 (d, $^1J_{C\text{-}P}$ = 165.7 Hz, 2C, *C*7a and *C*7a’), 105.5 (d, $^3J_{C\text{-}P}$ = 17.1 Hz, 2C, *C*4 and *C*4’), 81.3 (hept, $^2J_{C\text{-}F}$ = 30.3 Hz, 2C, *C*3 and *C*3’), 55.7 (2C, C5O*C*H_3 and C5’O*C*H_3), 39.4 (d, $^1J_{C\text{-}P}$ = 118.8 Hz, ^{n}Bu-*C*H_2(CH_2)$_2$$CH_3$), 25.6 (d, $J_{C\text{-}P}$ = 6.0 Hz, ^{n}Bu-*C*H), 23.8 (d, $J_{C\text{-}P}$ = 23.9 Hz, ^{n}Bu-*C*H), 16.8 (2C, C6*C*H_3 and C6’*C*H_3), 13.4 (d, $^4J_{C\text{-}P}$ = 1.3 Hz, ^{n}Bu-*C*H_3); ^{19}F{^{1}H} NMR (471 MHz, $CDCl_3$) δ −74.7 (q, $^4J_{F\text{-}F}$ = 9.4 Hz, 6F, C3C*F*$_3$ and C3’C*F*$_3$), −75.0 (q, $^4J_{F\text{-}F}$ = 9.4 Hz, 6F, C3C*F*$_3$ and C3’C*F*$_3$); ^{31}P{^{1}H} NMR (202 MHz, $CDCl_3$) δ −20.1; HRMS (ESI$^+$): *m/z* calcd. 661.1372 for ([M+H]$^+$), found 661.1365; The e.r was determined by HPLC using a *Chiralcel IB-N3* analytical column (0.20 mL min^{-1}, 100% heptane, 5 °C): t_{minor} = 20.5 min and t_{major} = 21.0 min. Assignments of HPLC peaks are in accord with their UV spectra.

[*TBPY*-5-11'-*A*]-1-Butyl-3,3,3',3'-tetrakis(trifluoromethyl)-3*H*,3'*H*-1λ⁵,1'-spirobi[naphtho[2,3-*c*][2,1]oxaphosphole] ((*S-trans*)-2f)

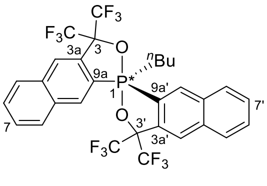

Prepared according to the general procedure **G** using P-H (apical) phosphorane **1f** (67.4 mg, 100 µmol, 1.00 eq.). The crude material was purified by column chromatography on silica gel (petroleum ether) to provide the title compound as white crystals (58.0 mg, 86.0 µmol, 86%, 96:4 e.r., 99:1 d.r., m.p. = 121 – 122 °C). R_f 0.60 (petroleum ether); $[\alpha]_D^{24}$ – 206.3 (*c* 0.1, $CHCl_3$); IR $\tilde{\nu}_{max}$ (neat): 3709m, 2970s, 2867m, 2361s, 2340m, 1275m, 1210m, 1152w, 1053s, 1013m, 969m, 894w, 752m, 671w; ^{1}H NMR (500 MHz, $CDCl_3$) δ 9.07 (d, $^3J_{H\text{-}P}$ = 13.4 Hz, 2H, C9*H and* C9’*H*), 8.22 (s, 2H, C4*H* and C4’*H*), 8.10 (d, $^3J_{H\text{-}H}$ = 7.9 Hz, 2H, C8*H* and C8’*H*), 8.00 (d, $^3J_{H\text{-}H}$ = 7.9 Hz, 2H, C5*H* and C5’*H*), 7.74 – 7.62 (m, 4H, C6*H*, C6’*H*, C7*H* and C7’*H*), 2.49 (dtd, *J* = 16.9, 12.3, 5.0 Hz,

1H, ^{n}Bu-C*H*), 2.35 (qd, *J* = 12.5, 3.9 Hz, 1H, ^{n}Bu-C*H*), 1.97 – 1.83 (m, 1H, ^{n}Bu-C*H*), 1.32 – 1.22 (m, 3H, ^{n}Bu-C*H*), 0.82 (t, $^3J_{H-H}$ = 6.7 Hz, 3H, ^{n}Bu-CH_3); ^{13}C{^{1}H} NMR (126 MHz, $CDCl_3$) δ 140.3 (d, $^2J_{C-P}$ = 9.8 Hz, 2C, *C*9 and *C*9'), 135.5 (d, $^4J_{C-P}$ = 2.8 Hz, 2C, *C*4a and *C*4a'), 134.6 (d, $^3J_{C-P}$ = 15.8 Hz, 2C, *C*8a and *C*8a'), 131.1 (d, $^2J_{C-P}$ = 20.2 Hz, 2C, *C*3a and *C*3a'), 129.9 (2C, *C*8 and *C*8'), 129.5 (2C, *C*6 and *C*6'), 128.4 (d, $^5J_{C-P}$ = 1.4 Hz, 2C, *C*5 and *C*5'), 127.9 (d, $^5J_{C-P}$ = 1.5 Hz, 2C, *C*7 and *C*7'), 126.1 (d, $^1J_{C-P}$ = 161.7 Hz, 2C, *C*9a and *C*9a'), 124.9 (d, $^3J_{C-P}$ = 14.9 Hz, 2C, *C*4 and *C*4'), 123.1 (q, $^1J_{C-F}$ = 286.8 Hz, 2C, C3*C*F_3 and C3'*C*F_3),123.0 (q, $^1J_{C-F}$ = 287.6 Hz, 2C, C3*C*F_3 and C3'*C*F_3), 81.3 (m, 2C, *C*3 and *C*3'), 39.5 (d, $^1J_{C-P}$ = 118.2 Hz, ^{n}Bu-*C*H_2(CH_2)$_2$$CH_3$), 25.6 (d, J_{C-P} = 6.2 Hz, ^{n}Bu-*C*H), 23.8 (d, J_{C-P} = 24.2 Hz, ^{n}Bu-*C*H), 13.4 (d, $^4J_{C-P}$ = 1.3 Hz, ^{n}Bu-*C*H_3); ^{19}F{^{1}H} NMR (471 MHz, $CDCl_3$) δ −74.5 (q, $^4J_{F-F}$ = 9.3 Hz, 6F, C3C*F*$_3$ and C3'C*F*$_3$), −75.0 (q, $^4J_{F-F}$ = 9.4 Hz, 6F, C3C*F*$_3$ and C3'C*F*$_3$); ^{31}P{^{1}H} NMR (202 MHz, $CDCl_3$) δ −22.4; HRMS (ESI$^+$): *m/z* calcd. 673.1160 for ([M+H]$^+$), found 673.1154; The e.r. was determined by HPLC using a *Chiralcel IB-N3* analytical column (0.50 mL min^{-1}, 100% heptane, 5 °C): t_{minor} = 9.7 min and t_{major} = 10.2 min. Assignments of HPLC peaks are in accord with their UV spectra.

[*TBPY*-5-11'-*A*]-1-Butyl-7,7'-dimethoxy-3,3,3',3'-tetrakis(trifluoromethyl)-3*H*,3'*H*-1λ^5,1'-spiro-bi[naphtho[2,3-*c*][2,1]oxaphosphole] ((*S*-*trans*)-2g)

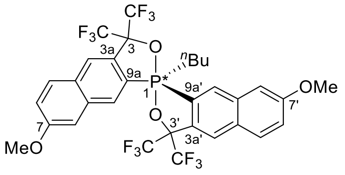

Prepared according to the general procedure **G** using P-H (apical) phosphorane **1g** (73.5 mg, 100 μmol, 1.00 eq.). The crude material was purified by column chromatography on silica gel (petroleum ether/EtOAc 20:1) to provide the title compound as white crystals (54.0 mg, 74.0 μmol, 74%, 92:8 e.r., 99:1 d.r., m.p. = 145 – 146 °C). R_f 0.60 (petroleum ether/EtOAc 20:1); $[\alpha]_D^{24}$ – 309.8 (*c* 0.1, $CHCl_3$); IR $\tilde{\nu}_{max}$ (neat):3709s, 2958s, 2923s, 2858m, 2361m, 1464m, 1378m, 1206m, 1054m, 1013m, 825w, 722m; ^{1}H NMR (500 MHz, $CDCl_3$) δ 8.91 (d, $^3J_{H-P}$ = 13.4 Hz, 2H, C9*H* and C9'*H*), 8.13 – 8.09 (m, 2H, C4*H* and C4'*H*), 7.87 (d, *J* = 9.0 Hz, 2H, C5*H* and C5'*H*), 7.35 (dd, *J* = 9.0, 2.5 Hz, 2H, C6*H* and C6'*H*), 7.31 (d, *J* = 2.5 Hz, 2H, C8*H* and C8'*H*), 3.99 (s, 6H, C7OC*H*$_3$ and C7'OC*H*$_3$), 2.45 (dtd, *J* = 15.8, 12.4, 4.8 Hz, 1H, ^{n}Bu-C*H*), 2.30 (qd, *J* = 12.5, 4.0 Hz, 1H, ^{n}Bu-C*H*), 1.94 – 1.80 (m, 1H, ^{n}Bu-C*H*), 1.33 – 1.27 (m, 3H, ^{n}Bu-C*H*), 0.80 (t, $^3J_{H-H}$ = 7.2 Hz, 3H, ^{n}Bu-C*H*$_3$); ^{13}C{^{1}H} NMR (151 MHz, $CDCl_3$) δ 159.1 (d, *J* = 1.8 Hz, *C*7 and *C*7'), 138.5 (d, *J* = 9.9 Hz, *C*9 and *C*9'), 136.1 (d, *J* = 16.2 Hz, *C*8a and *C*8a'), 131.1 (d, *J* = 2.9 Hz, *C*4a and *C*4a'), 129.7 (d, *J* = 1.5 Hz, *C*5 and *C*5'), 128.9 (d, *J* = 20.2 Hz, *C*3a and *C*3a'), 126.7 (d, $^1J_{C-P}$ = 161.1 Hz, *C*9a and *C*9a'), 124.5 (d, *J* = 15.4 Hz, *C*4 and *C*4'), 123.2 (q, $^1J_{C-F}$ = 287.0 Hz, 2C, C3*C*F_3 and C3'*C*F_3), 123.0 (q, $^1J_{C-F}$ = 287.6 Hz, 2C, C3*C*F_3 and C3'*C*F_3), 122.8 (2C, *C*6 and *C*6'), 107.0 (2C, *C*8 and *C*8'), 81.1 (hept, $^2J_{C-F}$ = 31.1 Hz, 2C, *C*3 and *C*3'), 55.7 (2C, C7O*C*H_3 and C7'O*C*H_3), 39.5 (d, $^1J_{C-P}$ = 118.1 Hz, ^{n}Bu-*C*H_2(CH_2)$_2$$CH_3$), 25.6 (d, *J* = 6.2 Hz, ^{n}Bu-*C*H), 23.8 (d, *J* = 24.2 Hz, ^{n}Bu-*C*H), 13.4 (d, *J* = 1.3 Hz, ^{n}Bu-*C*H_3); ^{19}F{^{1}H} NMR (376 MHz, $CDCl_3$) δ −74.5 (q, $^4J_{F-F}$ = 9.4 Hz, 6F, C3C*F*$_3$ and C3'C*F*$_3$), −75.1 (q, $^4J_{F-F}$ = 9.5 Hz, 6F, C3C*F*$_3$ and C3'C*F*$_3$); ^{31}P{^{1}H} NMR (162 MHz, $CDCl_3$) δ −22.2. HRMS (ESI$^+$): *m/z* calcd. 733.1372 for ([M+H]$^+$), found 733.1358; The e.r. was determined by HPLC using a *Chiralcel IB-N3* analytical column (0.20 mL min^{-1}, 100% heptane, 5 °C): t_{minor} = 48.1 min and t_{major} = 51.8 min. Assignments of HPLC peaks are in accord with their UV spectra.

[*TBPY*-5-11'-*A*]-1-Hexyl-6,6'-dimethyl-3,3,3',3'-tetrakis(trifluoromethyl)-3*H*,3'*H*-1λ⁵,1'-spirobi[benzo[*c*][2,1]oxaphosphole] ((*S-trans*)-2h)

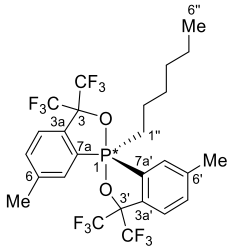

Prepared according to the general procedure **G** using P-H (apical) phosphorane **1h** (63.0 mg, 100 μmol, 1.00 eq.). The crude material was purified by column chromatography on silica gel (pentane) to provide the title compound as colourless oil (53.0 mg, 84.3 μmol, 84%, 91:9 e.r., 92:8 d.r.); R_f 0.80 (pentane); $[\alpha]_D^{23}$ – 67.3 (*c* 0.1, $CHCl_3$); IR $\tilde{\nu}_{max}$ (neat): 2930m, 2361w, 1276m, 1200s, 1144s, 1079m, 971s, 882w, 827w, 653m; ^{1}H NMR (500 MHz, $CDCl_3$) δ 8.20 (d, $^3J_{P-H}$ = 11.6 Hz, 2H, C7*H* and C7'*H*), 7.62-7.56 (m, 2H, C4*H* and C4'*H*), 7.48 (dtd, *J* = 7.9, 1.9, 0.9 Hz, 2H, C5*H* and C5'*H*), 2.48 (s, 6H, C6C*H₃* and C6'C*H₃*), 2.40 – 2.27 (m, 1H, C1''*H*), 2.19 (m, 1H, C1''*H*), 1.89 – 1.71 (m, 1H, *ⁿ*Hex-C*H*), 1.35 – 1.12 (m, 7H, *ⁿ*Hex-C*H₂*), 0.82 (t, *J* = 7.0 Hz, 3H, C6''*H₃*); $^{13}C\{^1H\}$ NMR (126 MHz, $CDCl_3$) δ 141.6 (d, $^3J_{C-P}$ = 13.9 Hz, *C*6 and *C*6'), 137.5 (d, $^2J_{C-P}$ = 9.7 Hz. *C*7 and *C*7'), 134.4 (d, $^4J_{C-P}$ = 3.4 Hz, *C*5 and *C*5'), 133.5 (d, $^2J_{C-P}$ = 19.1 Hz, *C*3a and *C*3a'), 130.4 (d, $^1J_{C-P}$ = 157.8 Hz, *C*7a and *C*7a'), 124.5 (d, $^3J_{C-P}$ = 16.0 Hz, *C*4 and *C*4'), 122.8 (q, $^1J_{C-F}$ = 286.8 Hz, C3*C*F₃ and C3'*C*F₃), 122,6 (q, $^1J_{C-F}$ = 286.6 Hz, C3*C*F₃ and C3'*C*F₃), 81.2 (hept, $^2J_{C-F}$ = 30.1 Hz, *C*3 and *C*3'), 39.5 (d, $^1J_{C-P}$ = 116.7 Hz, *C*1''), 30.9 (d, J_{C-P} = 1.3 Hz, *ⁿ*Hex-*C*H₂), 30.3 (d, J_{C-P} = 22.9 Hz, *ⁿ*Hex-*C*H₂), 23.2 (d, J_{C-P} = 6.2 Hz, *ⁿ*Hex-*C*H₂), 22.3 (*ⁿ*Hex-*C*H₂), 21.4 (d, $^3J_{C-P}$ = 1.5 Hz, C6*C*H₃ and C6'*C*H₃), 13.9 (*C*6''); $^{19}F\{^1H\}$ NMR (471 MHz, $CDCl_3$) δ −74.8 (q, $^4J_{F-F}$ = 9.4 Hz, 6F, 2C*F₃*), −75.1 (q, $^4J_{F-F}$ = 9.4 Hz, 6F, 2C*F₃*); this compound was isolated as a mixture of diastereomers; ^{31}P NMR (202 MHz, $CDCl_3$) δ −4.3 (minor diastereomer, (*cis*)-**2h**), −19.8 (major diastereomer, (*S-trans*)-**2h**); HRMS (ESI$^+$): *m/z* calcd. 629.1473 for ([M+H]$^+$), found 629.1477; The e.r. was determined by HPLC using a *Chiralcel IB-N3* analytical column (0.50 mL min^{-1}, 100% heptane, 5 °C): t_{minor} = 7.7 min and t_{major} = 8.3 min. Assignments of HPLC peaks are in accord with their UV spectra.

[*TBPY*-5-11'-*A*]-6,6'-Dimethyl-1-(5-methylhexyl)-3,3,3',3'-tetrakis(trifluoromethyl)-3*H*,3'*H*-1λ⁵,1'-spirobi[benzo[*c*][2,1]oxaphosphole] ((*S-trans*)-2i)

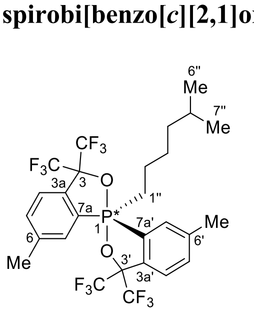

Prepared according to the general procedure **G** using P-H (apical) phosphorane **1i** (64.4 mg, 100 μmol, 1.00 eq.). The crude material was purified by column chromatography on silica gel (pentane) to provide the title compound as white solid (51.0 mg, 79.4 μmol, 79%, 91:9 e.r., 97:3 d.r., m.p. = 88 – 89 °C); R_f 0.80 (pentane); $[\alpha]_D^{23}$ – 62.4 (*c* 0.1, $CHCl_3$); IR $\tilde{\nu}_{max}$ (neat): 2927w, 2361m, 1468w, 1275m, 1199s, 1144s, 1079m, 971s, 909w, 826w, 728w, 653m; ^{1}H NMR (500 MHz, $CDCl_3$) δ 8.19 (d, $^3J_{P-H}$ = 11.5 Hz, 2H, C7*H*, and C7'*H*), 7.62 – 7.56 (m, 2H, C4*H*,

and C4'*H*), 7.48 (dtd, *J* = 7.9, 1.8, 0.8 Hz, 2H, C5*H* and C5'*H*), 2.48 (s, 6H, C6CH_3 and C6'CH_3), 2.39 – 2.28 (m, 1H, C1''*H*), 2.24 – 2.13 (m, 1H, C1''*H*), 1.87 – 1.70 (m, 1H, Alkyl-C*H*), 1.45 (hept, *J* = 13.3 Hz, 1H, C5''*H*), 1.30 – 1.19 (m, 3H, Alkyl-CH_2), 1.13 – 1.03 (m, 2H, Alkyl-CH_2), 0.80 (dd, *J* = 6.6, 0.8 Hz, 6H, C6''CH_3 and C7''CH_3); ^{13}C{^{1}H} NMR (126 MHz, $CDCl_3$) δ 141.7 (d, $^{3}J_{C\text{-}P}$ = 13.9 Hz, 2C, *C*6 and *C*6'), 137.7 (d, $^{2}J_{C\text{-}P}$ = 9.7 Hz, 2C, *C*7 and *C*7'), 134.6 (d, $^{4}J_{C\text{-}P}$ = 3.4 Hz, 2C, *C*5 and *C*5'), 133.7 (d, $^{2}J_{C\text{-}P}$ = 19.1 Hz, 2C, *C*3a and *C*3a'), 130.6 (d, $^{1}J_{C\text{-}P}$ = 157.8 Hz, 2C, *C*7a and *C*7a'), 124.6 (d, $^{3}J_{C\text{-}P}$ = 16.1 Hz, 2C, *C*4 and *C*4'), 123.0 (q, $^{1}J_{C\text{-}F}$ = 286.6 Hz, 2C, C3CF_3 and C3'CF_3) 122.7 (q, $^{1}J_{C\text{-}F}$ = 290.0 Hz, 2C, C3CF_3 and C3'CF_3), 81.3 (hept, $^{2}J_{C\text{-}F}$ = 29.8 Hz, 2C, *C*3 and *C*3'), 39.7 (d, $^{1}J_{C\text{-}P}$ = 116.7 Hz, *C*1''), 38.2 (d, *J* = 1.3 Hz, Alkyl-CH_2), 28.5 (d, *J* = 22.7 Hz, Alkyl-CH_2), 27.9 (*C*5''), 23.7 (d, $J_{C\text{-}P}$ = 6.1 Hz, Alkyl-CH_2), 22.6 (d, $^{6}J_{C\text{-}P}$ = 3.9 Hz, 2C, *C*6''and *C*7''), 21.6 (d, $^{4}J_{C\text{-}P}$ = 1.6 Hz, 2C, C6CH_3 and C6'CH_3); ^{19}F{^{1}H} NMR (471 MHz, $CDCl_3$) δ −74.8 (q, $^{4}J_{F\text{-}F}$ = 9.4 Hz, 6F, C3CF_3 and C3'CF_3), −75.1 (q, $^{4}J_{F\text{-}F}$ = 9.5 Hz, 6F, C3CF_3 and C3'CF_3); this compound was isolated as a mixture of diastereomers; ^{31}P{^{1}H} NMR (202 MHz, $CDCl_3$) δ −4.3 (minor diastereomer, (*cis*)-**2i**), −19.9 (major diastereomer, (*S-trans*)-**2i**); HRMS (ESI^+): *m/z* calcd. 643.1630 for ($[M+H]^+$), found 643.1625; The e.r. was determined by HPLC using a *Chiralcel IB-N3* analytical column (0.50 mL min^{-1}, 100% heptane, 5 °C): t_{minor} = 7.5 min and t_{major} = 8.0 min. Assignments of HPLC peaks are in accord with their UV spectra.

[*TBPY*-5-11'-*A*]-6,6'-Dimethyl-1-(4-phenylbutyl)-3,3,3',3'-tetrakis(trifluoromethyl)-3*H*,3'*H*-1λ^5,1'-spirobi[benzo[*c*][2,1]oxaphosphole] ((*S-trans*)-2j)

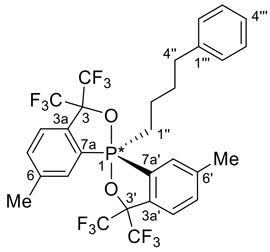

Prepared according to the general procedure **G** using P-H (apical) phosphorane **1j** (67.8 mg, 100 µmol, 1.00 eq.). The crude material was purified by column chromatography on silica gel (pentane) to provide the title compound as colourless oil (66.0 mg, 97.6 µmol, 98%, 95:5 e.r., >99:1 d.r.); R_f 0.50 (pentane); $[\alpha]_D^{25}$ – 61.7 (*c* 0.1, $CHCl_3$); IR $\tilde{\nu}_{max}$ (neat): 3030w, 2362w, 1604w, 1490w, 1276s, 1220s, 1146s, 1079m, 971s, 883w, 826w, 728m; ^{1}H NMR (500 MHz, $CDCl_3$) δ 8.22 – 8.13 (m, 2H, C7*H*, and C7'*H*), 7.61 – 7.57 (m, 2H, C4*H*, and C4'*H*), 7.52 – 7.41 (m, 2H, C5*H* and C5'*H*), 7.27 – 7.17 (m, 2H, C3'''*H* and C5'''*H*), 7.18 – 7.12 (m, 1H, C4'''*H*), 7.12 – 7.04 (m, 2H, C2'''*H* and C6'''*H*), 2.56 – 2.44 (m, 8H, C6CH_3 and C6'CH_3 overlapping with alkyl-CH_2), 2.44 – 2.34 (m, 1H, C1''*H*), 2.31 – 2.18 (m, 1H, C1''*H*), 1.90 – 1.82 (m, 1H, Alkyl-C*H*), 1.63 – 1.49 (m, 2H, Alkyl-CH_2), 1.41 – 1.31 (m, 1H, Alkyl-C*H*); ^{13}C{^{1}H} NMR (126 MHz, $CDCl_3$) δ 142.4 (*C*1'''), 141.8 (d, $^{3}J_{C\text{-}P}$ = 14.1 Hz, 2C, *C*6 and *C*6'), 137.7 (d, $^{2}J_{C\text{-}P}$ = 9.6 Hz, 2C, *C*7 and *C*7'), 134.7 (2C, *C*5 and *C*5'), 133.7 (d, $^{2}J_{C\text{-}P}$ = 19.3 Hz, 2C, *C*3a and *C*3a'), 130.5 (d, $^{1}J_{C\text{-}P}$ = 158.1 Hz, 2C, *C*7a and *C*7a'), 128.4 (4C, *C*2''', *C*3''', *C*5''', *C*6'''), 125.8 (*C*4'''), 124.6 (d, $^{3}J_{C\text{-}P}$ = 15.9 Hz, 2C, *C*4 and *C*4'), 122.9 (q, $^{1}J_{C\text{-}F}$ = 286.1 Hz, 2C, C3CF_3 and C3'CF_3), 122.7 (q, $^{1}J_{C\text{-}F}$ = 287.8 Hz, 2C, C3CF_3 and C3'CF_3), 81.3 (hept, $^{2}J_{C\text{-}F}$ = 30.6 Hz, 2C, *C*3 and *C*3'), 39.5 (d, $^{1}J_{C\text{-}P}$ = 117.6 Hz, *C*1''), 35.3 (Alkyl-CH_2), 32.7 (d, $J_{C\text{-}P}$ = 22.4 Hz, Alkyl-CH_2), 23.5 (d, $J_{C\text{-}P}$ = 6.2 Hz, Alkyl-CH_2), 21.6 (2C, C6CH_3 and C6'CH_3); ^{19}F{^{1}H} NMR (376 MHz, $CDCl_3$) δ −74.7 (q, $^{4}J_{F\text{-}F}$ = 9.2 Hz, 6F, C3CF_3 and C3'CF_3), −75.0 (q, $^{4}J_{F\text{-}F}$ = 9.4 Hz, 6F, C3CF_3 and C3'CF_3); ^{31}P{^{1}H} NMR (202 MHz, $CDCl_3$) δ −20.1; HRMS (ESI^+): *m/z* calcd. 677.1473

for ([M+H]$^+$), found 677.1459; The e.r. was determined by HPLC using a *Chiralcel IB-N3* analytical column (0.50 mL min^{-1}, 100% heptane, 5 °C): t_{minor} = 11.8 min and t_{major} = 12.8 min. Assignments of HPLC peaks are in accord with their UV spectra.

[*TBPY*-5-11'-*A*]-6,6'-Dimethyl-1-(pent-4-en-1-yl)-3,3,3',3'-tetrakis(trifluoromethyl)-3*H*,3'*H*-1λ^5,1'-spirobi[benzo[*c*][2,1]oxaphosphole] ((*S*-*trans*)-2k)

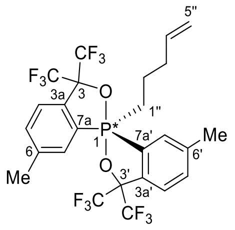

Prepared according to the general procedure **G** using P-H (apical) phosphorane **1k** (61.4 mg, 100 µmol, 1.00 eq.). The crude material was purified by column chromatography on silica gel (pentane) to provide the title compound as colourless oil (47.0 mg, 76.7 µmol, 77%, 93:7 e.r., 97:3 d.r.); R_f 0.80 (pentane); $[\alpha]_D^{24}$ – 64.6 (*c* 0.1, $CHCl_3$); IR $\tilde{\nu}_{max}$ (neat): 3085w, 2929w, 2360w, 1276m, 1200s, 1143s, 1079m, 971m, 827w, 654m; ^{1}H NMR (500 MHz, $CDCl_3$) δ 8.19 (d, $^3J_{P\text{-}H}$ = 11.6 Hz, 2H, C7*H*, and C7'*H*), 7.59 (m, 2H, C4*H*, and C4'*H*), 7.49 (dt, *J* = 8.3, 2.1 Hz, 2H, C5*H* and C5'*H*), 5.65 (ddt, *J* = 17.1, 10.4, 6.8 Hz, 1H, C4''*H*), 4.97 – 4.88 (m, 2H, C5''*H* and C5''*H* overlapping), 2.48 (s, 6H, C6CH_3 and C6'CH_3), 2.34 (dtd, *J* = 16.2, 12.8, 4.7 Hz, 1H, C1''*H*), 2.19 (qd, *J* = 12.1, 4.3 Hz, 1H, C1''*H*), 2.07 – 1.97 (m, 2H, C3''*H*), 1.98 – 1.83 (m, 1H, C2''*H*), 1.36 (qtd, *J* = 12.8, 6.7, 4.1 Hz, 1H, C2''*H*); ^{13}C{^{1}H} NMR (126 MHz, $CDCl_3$) δ 141.6 (d, $^3J_{C\text{-}P}$ = 13.9 Hz, 2C, *C*6 and *C*6'), 137.6 (2C, *C*7 and *C*7'), 137.5 (*C*4''), 134.5 (d, $^4J_{C\text{-}P}$ = 3.4 Hz, 2C, *C*5 and *C*5'), 133.5 (d, $^2J_{C\text{-}P}$ = 19.2 Hz, *C*3a and *C*3a'), 130.3 (d, $^1J_{C\text{-}P}$ = 157.9 Hz, 2C, *C*7a and *C*7a'), 124.5 (d, $^3J_{C\text{-}P}$ = 16.0 Hz, 2C, *C*4 and *C*4'), 122.8 (q, $^1J_{C\text{-}F}$ = 286.5 Hz, 2C, C3*C*F_3 and C3'*C*F_3), 122.6 (q, $^1J_{C\text{-}F}$ = 289.7 Hz, 2C, C3*C*F_3 and C3'*C*F_3), 115.5 (*C*5''), 81.2 (hept, $^2J_{C\text{-}F}$ = 31.3 Hz, 2C, *C*3 and *C*3'), 39.0 (d, $^1J_{C\text{-}P}$ = 117.8 Hz, *C*1''), 34.7 (d, $^3J_{C\text{-}P}$ = 23.8 Hz, *C*3''), 22.8 (d, $^2J_{C\text{-}P}$ = 5.4 Hz, *C*2''), 21.4 (d, $^4J_{C\text{-}P}$ = 1.4 Hz, 2C, C6*C*H_3 and C6'*C*H_3); ^{19}F{^{1}H} NMR (471 MHz, $CDCl_3$) δ −74.8 (q, $^4J_{F\text{-}F}$ = 9.4 Hz, 6F, C3C*F*$_3$ and C3'C*F*$_3$), −75.1 (q, $^4J_{F\text{-}F}$ = 9.4 Hz, 6F, C3C*F*$_3$ and C3'C*F*$_3$); this compound was isolated as a mixture of diastereomers; ^{31}P{^{1}H} NMR (202 MHz, $CDCl_3$) δ −4.4 (minor diastereomer, (*cis*)-**2k**), −20.1 (major diastereomer, (*S*-*trans*)-**2k**); HRMS (ESI$^+$): *m/z* calcd. 613.1160 for ([M+H]$^+$), found 613.1155; The e.r. was determined by HPLC using a *Chiralcel IB-N3* analytical column (0.50 mL min^{-1}, 100% heptane, 5 °C): t_{minor} = 7.9 min and t_{major} = 8.7 min. Assignments of HPLC peaks are in accord with their UV spectra.

[*TBPY*-5-11'-*A*]-6,6'-Dimethyl-1-phenethyl-3,3,3',3'-tetrakis(trifluoromethyl)-3*H*,3'*H*-1λ^5,1'-spirobi[benzo[*c*][2,1]oxaphosphole] ((*S*-*trans*)-2l)

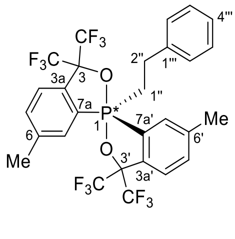

Prepared according to the general procedure **G** using P-H (apical) phosphorane **1l** (65.0 mg, 100 µmol, 1.00 eq.). The crude material was purified by column chromatography on silica gel (pentane) to provide the title compound

as colourless oil (55.0 mg, 84.8 µmol, 85%, 78:22 e.r., 91:9 d.r.); R*f* 0.60 (pentane); $[\alpha]_D^{23}$ – 43.9 (*c* 0.1, $CHCl_3$); IR $\tilde{\nu}_{max}$ (neat): 2927w, 2361w, 1604w, 1274m, 1197s, 1146s, 1078m, 962s, 882w, 827m, 758m, 701m, 653m; ^{1}H NMR (500 MHz, $CDCl_3$) δ 8.21 (d, $^3J_{H\text{-}P}$ = 11.6 Hz, 2H, C7*H* and C7'*H*), 7.66 – 7.60 (m, 2H, C4*H*, and C4'*H*), 7.51 (dt, *J* = 8.0, 2.0 Hz, 2H, C5*H* and C5'*H*), 7.24 – 7.19 (m, 2H, C3'''*H* and C5'''*H*), 7.18 – 7.10 (m, 1H, C4'''*H*), 7.06 (d, *J* = 8.0 Hz, 2H, C2'''*H* and C6'''*H*), 3.19 – 3.10 (m, 1H, C2''*H*), 2.69 – 2.56 (m, 1H, C1''*H*), 2.56 – 2.38 (m, 8H, C2''*H*, C1''*H*, C6CH_3 and C6'CH_3 overlapping); ^{13}C{^{1}H} NMR (126 MHz, $CDCl_3$) δ 141.9 (d, $^3J_{C\text{-}P}$ = 14.0 Hz, 2C, *C*6 and *C*6'), 141.6 (d, $^3J_{C\text{-}P}$ = 23.6 Hz, *C*1'''), 137.7 (d, $^2J_{C\text{-}P}$ = 9.8 Hz, 2C, *C*7 and *C*7'), 134.8 (d, $^4J_{C\text{-}P}$ = 3.5 Hz, 2C, *C*5 and *C*5'), 133.7 (d, $^2J_{C\text{-}P}$ = 19.4 Hz, 2C, *C*3a and *C*3a'), 130.1 (d, $^1J_{C\text{-}P}$ = 158.1 Hz, 2C, *C*7a and *C*7a'), 128.6 (2C, *C*3''' and *C*5'''), 128.3 (2C, *C*2''' and *C*6'''), 126.3 (*C*4'''), 124.7 (d, $^3J_{C\text{-}P}$ = 16.0 Hz, 2C, *C*4 and *C*4'), 123.0 (q, $^1J_{C\text{-}F}$ = 286.1 Hz, 2C, C3*C*F_3 and C3'*C*F_3) 122.7 (q, $^1J_{C\text{-}F}$ = 290.1 Hz, 2C, C3*C*F_3 and C3'*C*F_3), 81.4 (hept, $^2J_{C\text{-}F}$ = 30.9 Hz, 2C, *C*3 and *C*3'), 42.0 (d, $^1J_{C\text{-}P}$ = 114.7 Hz, *C*1''), 30.2 (d, $^2J_{C\text{-}P}$ = 5.0 Hz. *C*2''), 21.6 (d, $^4J_{C\text{-}P}$ = 1.5 Hz, 2C, C6*C*H_3 and C6'*C*H_3); ^{19}F{^{1}H} NMR (471 MHz, $CDCl_3$) δ −74.8 (q, $^4J_{F\text{-}F}$ = 9.2 Hz, 6F, C3C*F*$_3$ and C3'C*F*$_3$), −74.9 (q, $^4J_{F\text{-}F}$ = 9.2 Hz, 6F, C3C*F*$_3$ and C3'C*F*$_3$); ^{31}P{^{1}H} NMR (202 MHz, $CDCl_3$) δ −22.0; HRMS (ESI$^+$): *m/z* calcd. 649.1160 for ([M+H]$^+$), found 649.1163; The e.r. was determined by HPLC using a *Chiralcel IB-N3* analytical column (0.50 mL min^{-1}, 100% heptane, 5 °C): t_{minor} = 9.2 min and t_{major} = 9.7 min. Assignments of HPLC peaks are in accord with their UV spectra.

[*TBPY*-5-11'-*A*]-1,6,6'-Trimethyl-3,3,3',3'-tetrakis(trifluoromethyl)-3*H*,3'*H*-1λ^5,1'-spirobi[benzo[*c*][2,1]oxaphosphole] ((*S*-*trans*)-2m)

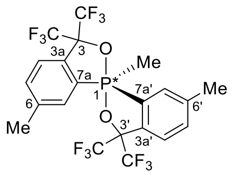

Prepared according to the general procedure **G** using P-H (apical) phosphorane **1m** (56.0 mg, 100 µmol, 1.00 eq.). The crude material was purified by column chromatography on silica gel (pentane) to provide the title compound as white crystals (52.0 mg, 93.1 µmol, 93%, 90:10 e.r., >99:1 d.r., m.p. = 134 – 135 °C); R*f* 0.85 (pentane); $[\alpha]_D^{25}$ – 77.3 (*c* 0.1, $CHCl_3$); IR $\tilde{\nu}_{max}$ (neat): 2927w, 2361m, 1275s, 1197s. 1143s, 1079m, 972s, 827m, 650m; ^{1}H NMR (500 MHz, $CDCl_3$) δ 8.20 (d, $^3J_{P\text{-}H}$ = 11.9 Hz, 2H, C7*H* and C7'*H*), 7.63 – 7.57 (m, 2H, C4*H*, and C4'*H*), 7.49 (dt, *J* = 8.0, 2.0 Hz, 2H, C5*H* and C5'*H*), 2.48 (s, 6H, C6CH_3 and C6'CH_3), 2.10 (d, $^2J_{P\text{-}H}$ = 16.6 Hz, 3H, PCH_3); ^{13}C{^{1}H} NMR (126 MHz, $CDCl_3$) δ 141.9 (d, $^3J_{C\text{-}P}$ = 14.1 Hz, 2C, *C*6 and *C*6'), 137.3 (d, $^2J_{C\text{-}P}$ = 10.1 Hz, 2C, *C*7 and *C*7'), 134.8 (d, $^4J_{C\text{-}P}$ = 3.5 Hz, 2C, *C*5 and *C*5'), 133.5 (d, $^2J_{C\text{-}P}$ = 20.3 Hz, 2C, *C*3a and *C*3a'), 130.8 (d, $^1J_{C\text{-}P}$ = 159.2 Hz, 2C, *C*7a and *C*7a'), 124.6 (d, $^3J_{C\text{-}P}$ = 16.4 Hz, 2C, *C*4 and *C*4'), 122.9 (q, $^1J_{C\text{-}F}$ = 286.8 Hz, 2C, C3*C*F_3 and C3'*C*F_3), 122.7 (q, $^1J_{C\text{-}F}$ = 290.3 Hz, 2C, C3*C*F_3 and C3'*C*F_3), 81.4 (hept, $^2J_{C\text{-}F}$ = 31.0 Hz, 2C, *C*3 and *C*3'), 25.4 (d, $^1J_{C\text{-}P}$ = 124.9 Hz, P*C*H_3), 21.6 (d, $^4J_{C\text{-}P}$ = 1.5 Hz, 2C, C6*C*H_3 and C6'*C*H_3); ^{19}F{^{1}H} NMR (471 MHz, $CDCl_3$) δ −74.7 (q, $^4J_{F\text{-}F}$ = 9.4 Hz, 6F, C3C*F*$_3$ and C3'C*F*$_3$), −75.1 (q, $^4J_{F\text{-}F}$ = 9.4 Hz, 6F, C3C*F*$_3$ and C3'C*F*$_3$); ^{31}P{^{1}H} NMR (202 MHz, $CDCl_3$) δ −23.7; HRMS (ESI$^+$): *m/z* calcd. 559.0691 for ([M+H]$^+$), found 559.0681; The e.r. was determined by HPLC using a *Chiralcel IB-N3* analytical column (0.50 mL min^{-1}, 100% heptane, 5 °C): t_{minor} = 8.0 min and t_{major} = 8.6 min. Assignments of HPLC peaks are in accord with their UV spectra.

[*TBPY*-5-11'-*A*]-6,6'-Dimethyl-3,3,3',3'-tetrakis(trifluoromethyl)-1-(6-(trimethylsi-lyl)hex-5-yn-1-yl)-3*H*,3'*H*-1λ^5,1'-spirobi[benzo[*c*][2,1]oxaphosphole] ((*S-trans*)-2n)

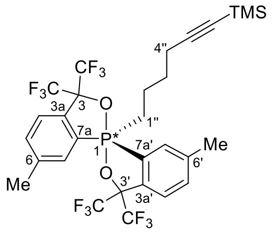

Prepared according to the general procedure **G** using P-H (apical) phosphorane **1n** (69.9 mg, 100 μmol, 1.00 eq.). The crude material was purified by column chromatography on silica gel (pentane) to provide the title compound as colourless oil (64.0 mg, 91.9 μmol, 92%, 93:7 e.r., 98:2 d.r.); R_f 0.40 (pentane); $[\alpha]_D^{24}$ – 65.0 (*c* 0.1, $CHCl_3$); IR $\tilde{\nu}_{max}$ (neat): 2926w, 2361m, 1488w, 1276m, 1201s, 1147m, 1079m, 972s, 846w, 703m, 633m; ^{1}H NMR (500 MHz, $CDCl_3$) δ 8.19 (d, $^3J_{H\text{-}P}$ = 11.5 Hz, 2H, C7*H* and C7'*H*), 7.62 – 7.56 (m, 2H, C4*H*, and C4'*H*), 7.49 (dtd, *J* = 7.9, 1.8, 0.8 Hz, 2H, C5*H* and C5'*H*), 2.48 (d, $^5J_{H\text{-}P}$ = 0.9 Hz, 6H, C6C*H*$_3$ and C6'C*H*$_3$), 2.41 – 2.28 (m, 1H, C1''*H*), 2.20 (qd, *J* = 12.3, 4.2 Hz, 1H, C1''*H*), 2.14 (t, $^3J_{H\text{-}H}$ = 7.0 Hz, 2H, C4''*H*), 1.98 – 1.85 (m, 1H, C2''*H*), 1.47 (ddt, *J* = 12.0, 7.3, 4.0 Hz, 2H, C3''*H*), 1.41 – 1.32 (m, 1H, C2''*H*), 0.08 (s, 9H, Si(C*H*$_3$)$_3$); ^{13}C{^{1}H} NMR (126 MHz, $CDCl_3$) δ 141.7 (d, $^3J_{C\text{-}P}$ = 13.9 Hz, 2C, *C*6 and *C*6'), 137.6 (d, $^2J_{C\text{-}P}$ = 9.6 Hz, 2C, *C*7 and *C*7'), 134.6 (d, $^4J_{C\text{-}P}$ = 3.4 Hz, 2C, *C*5 and *C*5'), 133.6 (d, $^2J_{C\text{-}P}$ = 19.2 Hz, 2C, *C*3a and *C*3a'), 130.2 (d, $^1J_{C\text{-}P}$ = 158.2 Hz, 2C, *C*7a and *C*7a'), 124.6 (d, $^3J_{C\text{-}P}$ = 16.1 Hz, 2C, *C*4 and *C*4'), 122.8 (q, $^1J_{C\text{-}F}$ = 286.3 Hz, 2C, C3*C*F$_3$ and C3'*C*F$_3$), 122.6 (q, $^1J_{C\text{-}F}$ = 289.3 Hz, 2C, C3*C*F$_3$ and C3'*C*F$_3$) 106.7 (*C*5''), 84.8 (*C*6''), 81.2 (hept, $^2J_{C\text{-}F}$ = 30.9 Hz, 2C, *C*3 and *C*3'), 39.3 (d, $^1J_{C\text{-}P}$ = 118.2 Hz, *C*1''), 29.8 (d, $^3J_{C\text{-}P}$ = 24.2 Hz, *C*3''), 22.8 (d, $^2J_{C\text{-}P}$ = 5.7 Hz, *C*2''), 21.5 (d, $^4J_{C\text{-}P}$ = 1.5 Hz, 2C, C6*C*H$_3$ and C6'*C*H$_3$), 19.4 (d, $^4J_{C\text{-}P}$ = 1.8 Hz, *C*4''), 0.0 (3C, Si(*C*H$_3$)$_3$); ^{19}F{^{1}H} NMR (471 MHz, $CDCl_3$) δ −74.8 (q, $^4J_{F\text{-}F}$ = 9.3 Hz, 6F, C3C*F*$_3$ and C3'C*F*$_3$), −75.1 (q, $^4J_{F\text{-}F}$ = 9.4 Hz, 6F, C3C*F*$_3$ and C3'C*F*$_3$); ^{31}P{^{1}H} NMR (202 MHz, $CDCl_3$) δ −20.4; HRMS (ESI$^+$): *m/z* calcd. 697.1556 for ([M+H]$^+$), found 697.1547; The e.r. was determined by HPLC using a *Chiralcel IB-N3* analytical column (0.50 mL min^{-1}, 100% heptane, 5 °C): t_{minor} = 8.2 min and t_{major} = 8.8 min. Assignments of HPLC peaks are in accord with their UV spectra.

[*TBPY*-5-11'-*A*]-1-(4-Methoxybutyl)-6,6'-dimethyl-3,3,3',3'-tetrakis(trifluoromethyl)-3*H*,3'*H*-1λ^5,1'-spirobi[benzo[*c*][2,1]oxaphosphole] ((*S-trans*)-2o)

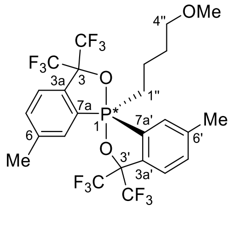

Prepared according to the general procedure **G** using P-H (apical) phosphorane **1o** (63.2 mg, 100 μmol, 1.00 eq.). The crude material was purified by column chromatography on silica gel (pentane/CH_2Cl_2 4:1) to provide the title compound as colourless oil (61.0 mg, 96.8 μmol, 97%, 91:9 e.r., 94:6 d.r.); R_f 0.45 (pentane/CH_2Cl_2 4:1); $[\alpha]_D^{24}$ – 66.7 (*c* 0.1, $CHCl_3$); IR $\tilde{\nu}_{max}$ (neat): 2924w, 2855w, 2362m, 1460w, 1276m, 1200s, 1142s, 1079m, 972s, 899w, 825w, 727w, 654w; ^{1}H NMR (500 MHz, $CDCl_3$) δ 8.18 (d, $^3J_{H\text{-}P}$= 11.4 Hz, 2H, C7*H* and C7'*H*), 7.59 (m, 2H, C4*H*, and C4'*H*), 7.48 (dtd, *J* = 7.9, 1.8, 0.8 Hz, 2H, C5*H* and C5'*H*), 3.28 (t, $^3J_{H\text{-}H}$ = 6.6 Hz, 2H, C4''*H*),

3.25 (s, 3H, OCH_3), 2.48 (s, 6H, C6CH_3 and C6'CH_3), 2.36 (dtd, J = 16.4, 12.7, 5.0 Hz, 1H, C1''H), 2.29 – 2.14 (m, 1H, C1''H), 1.95 – 1.80 (m, 1H, C2''H), 1.53 (p, J = 6.9 Hz, 2H, C3''H), 1.41 – 1.27 (m, 1H, C2''H); ^{13}C{^{1}H} NMR (126 MHz, $CDCl_3$) δ 141.8 (d, $^3J_{C\text{-}P}$ = 13.9 Hz, 2C, C6 and C6'), 137.7 (d, $^2J_{C\text{-}P}$ = 9.7 Hz, 2C, C7 and C7'), 134.7 (d, $^4J_{C\text{-}P}$ = 3.4 Hz, 2C, C5 and C5'), 133.7 (d, $^2J_{C\text{-}P}$ = 19.3 Hz, 2C, C3a and C3a'), 130.4 (d, $^1J_{C\text{-}P}$ = 158.3 Hz, 2C, C7a and C7a'), 124.7 (d, $^3J_{C\text{-}P}$ = 16.0 Hz, 2C, C4 and C4'), 122.9 (q, $^1J_{C\text{-}F}$ = 286.4 Hz, 2C, C3CF$_3$ and C3'CF$_3$), 122.7 (q, $^1J_{C\text{-}F}$ = 292.5 Hz, 2C, C3CF$_3$ and C3'CF$_3$), 81.3 (hept, $^2J_{C\text{-}F}$ = 30.7 Hz, 2C, C3 and C3'), 72.0 (d, $^4J_{C\text{-}P}$ = 1.5 Hz, C4''), 58.5 (OCH$_3$), 39.5 (d, $^1J_{C\text{-}P}$ = 117.7 Hz, C1''), 30.6 (d, $^3J_{C\text{-}P}$ = 22.9 Hz, C3''), 21.6 (d, $^4J_{C\text{-}P}$ = 1.5 Hz, 2C, C6CH$_3$ and C6'CH$_3$), 20.4 (d, $^2J_{C\text{-}P}$ = 5.8 Hz, C2''); ^{19}F{^{1}H} NMR (471 MHz, $CDCl_3$) δ −74.8 (q, $^4J_{F\text{-}F}$ = 9.4 Hz, 6F, C3CF_3 and C3'CF_3), −75.1 (q, $^4J_{F\text{-}F}$ = 9.4 Hz, 6F, C3CF_3 and C3'CF_3); this compound was isolated as a mixture of diastereomers; ^{31}P{^{1}H} NMR (202 MHz, $CDCl_3$) δ −4.6 (minor diastereomer, (*cis*)-**2o**), −20.3 (major diastereomer, (*S-trans*)-**2o**); HRMS (ESI$^+$): *m/z* calcd. 631.1266 for ([M+H]$^+$), found 631.1273; The e.r. was determined by HPLC using a *Chiralcel IB-N3* analytical column (0.50 mL min^{-1}, 100% heptane, 5 °C): t_{minor} = 12.1 min and t_{major} = 12.6 min. Assignments of HPLC peaks are in accord with their UV spectra.

[*TBPY*-5-11'-*A*]-1-(4-(1,3-Dioxolan-2-yl)butyl)-6,6'-dimethyl-3,3,3',3'-tetrakis(trifluoromethyl)-3*H*,3'*H*-1λ^5,1'-spirobi[benzo[*c*][2,1]oxaphosphole] ((*S-trans*)-2p)

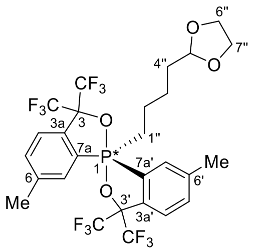

Prepared according to the general procedure **G** using P-H (apical) phosphorane **1p** (67.4 mg, 100 μmol, 1.00 eq.). The crude material was purified by column chromatography on silica gel (pentane/CH_2Cl_2 2:1) to provide the title compound as a white solid (55.0 mg, 81.8 μmol, 82%, 90:10 e.r., 93:7 d.r., m.p. = 96 – 97 °C); R$_f$ 0.40 (pentane/CH_2Cl_2 2:1); $[\alpha]_D^{25}$ – -57.5 (c 0.1, $CHCl_3$); IR $\tilde{\nu}_{max}$ (neat): 2919w, 2362w, 1487w, 1274m, 1195s, 1139s, 1077m, 962s, 881w, 827m, 726m, 652m; ^{1}H NMR (500 MHz, $CDCl_3$) δ 8.18 (d, $^3J_{H\text{-}P}$ = 11.5 Hz, 2H, C7H, and C7'H), 7.62 – 7.55 (m, 2H, C4H, and C4'H), 7.52 – 7.43 (m, 2H, C5H and C5'H), 4.77 (t, $^3J_{H\text{-}H}$ = 4.7 Hz, 1H, C5''H), 3.93 – 3.88 (m, 2H, C6''H and C7''H), 3.82 – 3.78 (m, 2H, C6''H and C7''H), 2.48 (s, 6H, C6CH_3 and C6'CH_3), 2.41 – 2.28 (m, 1H, Alk-CH), 2.26 – 2.14 (m, 1H, Alk-CH), 1.92 – 1.79 (m, 1H, Alk-CH), 1.64 – 1.49 (m, 2H, Alk-CH_2), 1.43 – 1.34 (m, 2H, Alk-CH_2), 1.34 – 1.27 (m, 1H, Alk-CH); ^{13}C{^{1}H} NMR (126 MHz, $CDCl_3$) δ 141.8 (d, $^3J_{C\text{-}P}$ = 13.9 Hz, 2C, C6 and C6'), 137.7 (d, $^2J_{C\text{-}P}$ = 9.7 Hz, 2C, C7 and C7'), 134.7 (d, $^4J_{C\text{-}P}$ = 3.4 Hz, 2C, C5 and C5'), 133.7 (d, $^2J_{C\text{-}P}$ = 19.2 Hz, 2C, C3a and C3a'), 130.4 (d, $^1J_{C\text{-}P}$ = 158.0 Hz, 2C, C7a and C7a'), 124.6 (d, $^3J_{C\text{-}P}$ = 16.1 Hz, 2C, C4 and C4'), 122.9 (q, $^1J_{C\text{-}F}$ = 286.4 Hz, 2C, C3CF$_3$ and C3'CF$_3$), 122.7 (q, $^1J_{C\text{-}F}$ = 289.9 Hz, 2C, C3CF$_3$ and C3'CF$_3$) 104.4 (C5''), 81.3 (hept, $^2J_{C\text{-}F}$ = 31.3 Hz, 2C, C3 and C3'), 65.0 (2C, C6''and C7''), 39.6 (d, $^1J_{P\text{-}H}$ = 117.3 Hz, C1''), 33.2 (d, J = 1.4 Hz, Alk-CH$_2$), 25.0 (d, J = 24.1 Hz, Alk-CH$_2$), 23.5 (d, J = 5.8 Hz, Alk-CH$_2$), 21.6 (d, $^4J_{C\text{-}P}$ = 1.5 Hz, 2C, C6CH$_3$ and C6'CH$_3$); ^{19}F{^{1}H} NMR (471 MHz, $CDCl_3$) δ −74.8 (q, $^4J_{F\text{-}F}$ = 9.4 Hz, 6F, C3CF_3 and C3'CF_3), −75.1 (q, $^4J_{F\text{-}F}$ = 9.4 Hz, 6F, C3CF_3 and C3'CF_3); this compound was isolated as a mixture of diastereomers; ^{31}P{^{1}H} NMR (202 MHz, $CDCl_3$) δ −4.8 (minor diastereomer, (*cis*)-**2p**), −20.2 (major diastereomer, (*S-trans*)-**2p**); HRMS (ESI$^+$): *m/z* calcd. 673.1372 for ([M+H]$^+$), found 673.1362; The

e.r. was determined by HPLC using two coupled *Chiralcel IB-N3* analytical columns (1.0 mL min^{-1}, 100% heptane, 40 °C): t_{minor} = 12.0 min and t_{major} = 12.4 min. Assignments of HPLC peaks are in accord with their UV spectra.

Stereodivergent control over pentavalent stereocentres

Synthesis of ((*R-trans*)-2a)

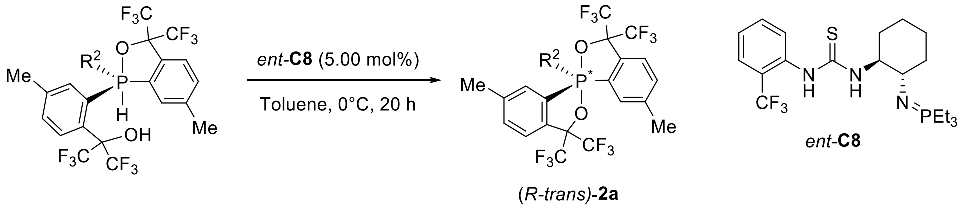

1-((1*S*,2*S*)-2-Azidocyclohexyl)-3-(2-(trifluoromethyl)phenyl)thiourea (*ent*-S7)

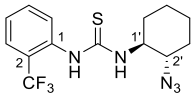

The enantiomer of the catalyst precursor *ent*-**S7** was prepared according to the procedure for the synthesis of **S7** using *tert*-butyl ((1*S*,2*S*)-2-azidocyclohexyl)carbamate (118 mg, 0.49 mmol, 1.00 eq.), TFA (1.00 mL), isothiocyanate **S6** (100 mg, 0.49 mmol, 1.00 eq.), and CH_2Cl_2 (1.0 mL). Light yellow solid (154 mg, 44.8 mmol, 91%); $[\alpha]_D^{25}$ + 63.3 (*c* 0.1, $CHCl_3$).

1-((1*S*,2*S*)-2-((Triethyl-λ^5-phosphaneylidene)amino)cyclohexyl)-3-(2-(trifluoromethyl)phenyl)thiourea (*ent*-C8)

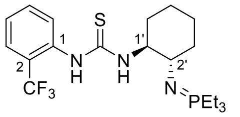

The enantiomer of the catalyst *ent*-**C8** was prepared according to the procedure for the synthesis of **C8** using triethylphosphine (19.0 µL, 128 µmol, 1.00 eq.) and azide *ent*-**S7** (44 mg, 128 µmol, 1.00 eq.) in THF (0.50 mL). White solid (51.0 mg, 118 µmol, 92%); $[\alpha]_D^{25}$ + 42.4 (*c* 0.1, $CHCl_3$).

[*TBPY*-5-11'-*C*]-1-Butyl-6,6'-dimethyl-3,3,3',3'-tetrakis(trifluoromethyl)-3*H*,3'*H*-1λ^5,1'-spirobi[benzo[*c*][2,1]oxaphosphole] ((*R-trans*)-2a)

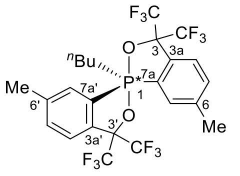

Folllowing the general procedure **E**, the reaction of hydridophosphorane substrate **1a** (60.2 mg, 100 µmol, 1.00 eq.) and catalyst *ent*-**C8** (2.17 mg, 5.00 µmol, 5.00 mol%) in toluene (5.0 mL) provided the title compound after column chromatorgraphy on silica gel (pentane) as white solid (59.0 mg, 98.3 µmol, 98%, 7:93 e.r., 99:1 d.r.). $[\alpha]_D^{25}$ + 60.9 (*c* 0.1, $CHCl_3$). The e.r. was determined by HPLC using a *Chiralcel IB-N3* analytical column (0.50 mL min^{-1}, 100% heptane, 5 °C): t_{minor} = 8.1 min and t_{major} = 7.7 min. Assignments of HPLC peaks are in accord with their UV spectra.

Stereodivergent control over pentavalent stereocentres

Optimisation of the reaction conditions for diastereodivergent control over pentavalent stereocentres: *cis*-diastereoselective bifunctional iminophosphorane-thiourea catalysed reaction

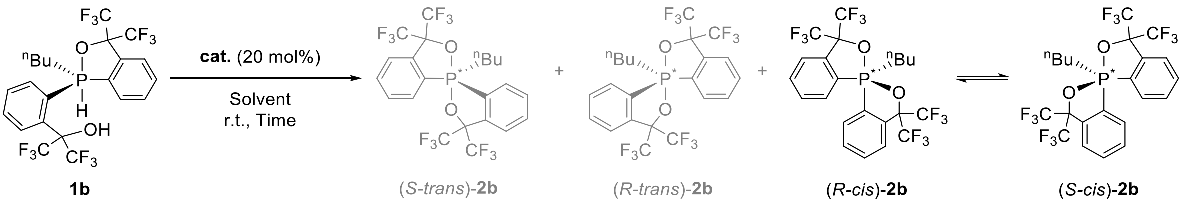

General procedure for the evaluation of the catalyst and reaction conditions

In a 1.5 mL crimp cap vial, catalyst (0.40 µmol, 20.0 mol%) and compound **1b** (1.15 mg, 2.00 µmol, 1.00 eq.) were dissolved in a solvent (0.30 mL) under an Ar atmosphere. The reaction mixture was stirred at r.t. for the time indicated. The solvent was removed under a stream of nitrogen. The residue was analysed by ^{19}F NMR to determine the conversion and d.r.

Supplementary Table 3. Catalyst and solvent evaluation for *cis*-selective dehydrogenation of **1b** on 2.00 µmol scale

Entry	Catalyst	Solvent	Time, h	Conv., %[a]	d.r.[a]
1	**SC19**	Toluene	12	21	65:35
2	**C9**	Toluene	12	59	21:79
3	**SC115**	Toluene	24	16	58:42
4	**SC116**	Toluene	24	84	78:22
5	**SC117**	Toluene	24	30	51:49
6	**C9**	Hexane	24	10	n.d.[b]
7	**C9**	PhCl	24	<5	n.d.[b]
8	**C9**	CH_2Cl_2	24	<5	n.d.[b]
9	**C9**	THF	24	<5	n.d.[b]
10	**C9**	MeCN	24	5	n.d.[b]
11	**SC117**	Hexane	24	5	n.d.[b]
12	**SC117**	PhCl	24	8	n.d.[b]
13	**SC117**	CH_2Cl_2	24	10	n.d.[b]
14	**SC117**	THF	24	15	67:33
15	**SC117**	MeCN	24	15	34:66

[a] Determined by ^{19}F NMR analysis of the crude reaction mixture. [b] The conversion was too low to determine the d.r.

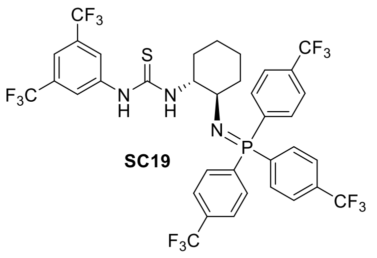

SC19

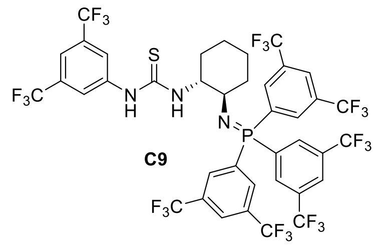

C9

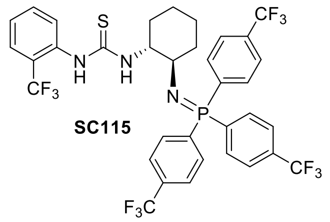

SC115

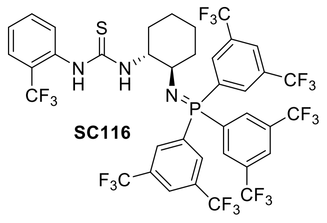

SC116

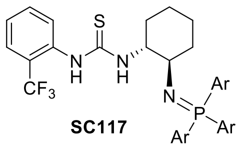

SC117

Ar = C_6F_5

Optimisation of the reaction conditions for diastereodivergent control over pentavalent stereocentres

Evaluation of the conditions for *cis*-selective Pd-catalysed reaction

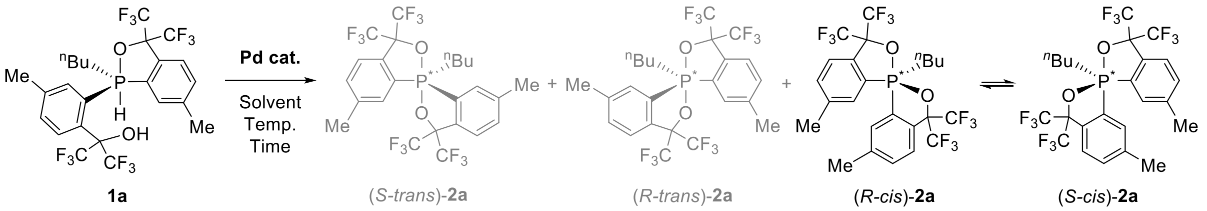

General procedure for the method optimisation

In a 1.5 mL crimp cap vial, catalyst and compound **1a** (1.20 mg, 2.00 µmol, 1.00 eq.) were dissolved in a solvent (0.30 mL). The reaction mixture was stirred at the indicated temperature for the time indicated. The solvent was removed under a stream of nitrogen. The residue was analysed by ^{19}F NMR to determine the conversion and d.r.

Supplementary Table 4. Evaluation of the conditions for Pd-catalysed *cis*-selective dehydrogenation of **1a**

Entry	Atm.	Catalyst	Loading/ mol%	Solvent	Temp./°C	Time/ h	Conv./%[a]	d.r.[a]
1	Ar	**$Pd(PPh_3)_4$**	10.0	Toluene	r.t.	7	44	13:87
2	Ar	**$Pd(PPh_3)_4$**	10.0	Toluene	r.t.	28	43	21:79
3	Ar	**$Pd(OAc)_2$**	10.0	Toluene	r.t.	7	63[b]	11:89
4	Air	**$Pd(PPh_3)_4$**	10.0	Toluene	r.t.	7	69	11:89
5[c]	Air	**$Pd(PPh_3)_4$**	10.0	Toluene	r.t.	7	66	23:77
6[d]	Air	**$Pd(PPh_3)_4$**	10.0	Toluene	r.t.	7	100	94:6
7	Air	**$Pd(PPh_3)_4$**	15.0	Toluene	r.t.	7	86	12:88
8	Air	**$Pd(PPh_3)_4$**	20.0	Toluene	r.t.	7	97	10:90
9[e]	Air	**$Pd(PPh_3)_4$**	5.00	Toluene	r.t.	8	67	15:85
10[f]	Air	**$Pd(PPh_3)_4$**	20.0	Toluene	0	20	80	5:95
11[f]	Air	**XPhos Pd G3**	20.0	Toluene	0	20	43	12:88
12[f]	Air	**$Pd(TFA)_2$**	20.0	Toluene	0	20	76	n.d[i].
13[f]	Air	**$Pd_2(dba)_3$**	20.0	Toluene	0	20	31	1:99
14[f]	Air	**$Pd(dba)_2$**	20.0	Toluene	0	20	40	1:99
15[f]	Air	**$(MeCN)_2PdCl_2$**	20.0	Toluene	0	20	98	9:91
16[f]	Air	**$[(MeCN)_4Pd](BF_4)_2$**	20.0	Toluene	0	20	27	2:98
17[f]	Air	**$Pd(PPh_3)_4$**	20.0	DMF	0	20	59	19:81

Entry	Atm.	Catalyst	Loading/ mol%	Solvent	Temp./°C	Time/ h	Conv./%[a]	d.r.[a]
18[f]	Air	**$Pd(PPh_3)_4$**	20.0	MeCN	0	20	88	4:96
19[f]	Air	**$Pd(PPh_3)_4$**	20.0	DMSO	r.t.	20	>99	28:72
20[f]	Air	**$Pd(PPh_3)_4$**	20.0	THF	0	20	91	2:98
21[f]	Air	**$Pd(PPh_3)_4$**	20.0	**MeOH**	0	20	**100**	**7:93**
22[f, g]	Air	**$Pd(PPh_3)_4$**	20.0	MeOH	0	72	88	37:63
23[f]	Air	**Pd/C**	20.0	MeOH	0	48	100	5:95
24[f, g]	Air	**Pd/C**	20.0	MeOH	0	48	>99	5:95
**25[f, h]**	**Air**	**Pd/C**	**20.0**	**MeOH**	**0**	**96**	**100**	**6:94**

[a] Determined by ^{19}F NMR analysis of the crude reaction mixture. [b] Decomposition of the starting material observed. [c] Et_3N (1.00 eq.) was used as an additive [d] Pyridine (1.00 eq.) was used as an additive [e] PPh_3 (20.0 mol%) was used as an additive. [f] 1.0 mL of the solvent was used. [g] 20.0 µmol scale. [h] 100 µmol scale. [i] Complex mixture of products was obtained.

[*TBPY*-5-12]-1-Butyl-6,6'-dimethyl-3,3,3',3'-tetrakis(trifluoromethyl)-3*H*,3'*H*-1λ^5,1'-spirobi[benzo[*c*][2,1]oxaphosphole] ((*cis*)-2a)

Synthesis of a reference material:

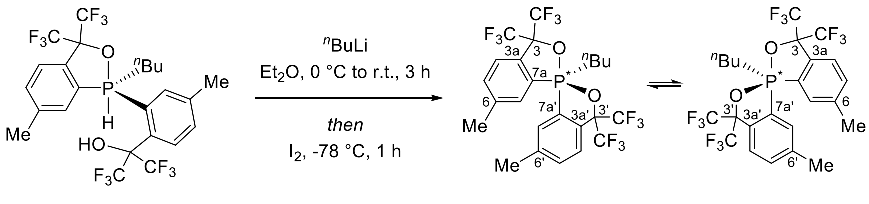

The reference material for *cis*-**2a** was obtained following literature reported procedure.[15] To a solution of **S4a** (109 mg, 0.20 mmol, 1.00 eq.) in Et_2O (2.0 mL) was added ^{n}BuLi (1.60 molL^{-1} in hexane, 0.38 mL, 0.60 mmol, 3.00 eq.) under an Ar atmosphere at 0 °C, and then the solution was stirred for 3 h at r.t. The solution was cooled to -78 °C, and then I_2 (162 mg, 0.64 mmol, 3.20 eq.) was added. The mixture was stirred for 1 h at -78 °C. The resulting solution was washed with aqueous $Na_2S_2O_3$ (2 x 5.0 mL) and brine (2 x 5.0 mL), and the organic layer was dried over anhydrous Na_2SO_4 and concentrated under the reduced pressure. The resulting residue was purified by flash column chromatography to afford *cis*-**2a** as a colourless oil (109 mg, 0.18 mmol, 91%; 14:86 d.r. for the crude, 2:98 d.r. after chromatography).

Stereodivergent *cis*-selective catalyst control:

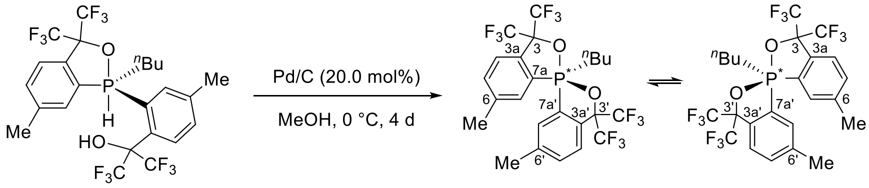

To a mixture of the hydridophosphorane substrate **1a** (60.2 mg, 100 µmol, 1.00 eq.) and Pd/C (5.00 % wt, 42.6 mg, 20.0 µmol, 20.0 mol%) in a 20 mL crimp cap vial at 0 °C under ambient atmosphere was added MeOH (HPLC-grade, 10 mL). Three needles were inserted through the cap of the vial for gas exchange with the open atmosphere. The mixture was stirred at 0 °C for 4 d, filtered through a short pad of silica gel (230 – 400 mesh) and the silica gel was washed with MeOH (2 x 5.0 mL). The solvent was removed under the reduced pressure at 10 – 20 °C to afford the title compound as a white solid (59.0 mg, 98.3 µmol, 98%, 6:94 d.r.). At room temperature, the product undergoes gradual interconversion to (±-*trans*)-**2a** and was therefore stored at –20 °C; R_f 0.20 (pentane); IR $\tilde{\nu}_{max}$ (neat): 2928m, 2364w, 1703m, 1606w, 1448m, 1275s, 1199s, 1147s, 1107s, 1038m, 974s, 911w, 826m, 716m, 651m; ^{1}H NMR (600 MHz, $CDCl_3$) δ 7.61 (d, $^3J_{H\text{-}H}$ = 8.0 Hz, 2H, C4*H* and C4'*H*), 7.44 (d, $^3J_{H\text{-}H}$ = 7.9 Hz, 2H, C5*H* and C5'*H*), 7.37 (d, $^3J_{H\text{-}P}$ = 7.5 Hz, 2H, C7*H* and C7'*H*), 2.50 – 2.40 (m, 8H, C6CH_3 and C6'CH_3 overlapping with PCH_2(CH_2)$_2$$CH_3$), 1.69 – 1.57 (m, 2H. P$CH_2CH_2$$CH_2$$CH_3$), 1.34 (tq, $^3J_{H\text{-}H}$ = 7.3, 7.3 Hz, 2H, P(CH_2)$_2$C$H_2$$CH_3$), 0.84 (t, $^3J_{H\text{-}H}$ = 7.3 Hz, 3H, P(CH_2)$_3$CH_3); ^{13}C{^{1}H} NMR (151 MHz, $CDCl_3$) δ 140.5 (d, $^3J_{C\text{-}P}$ = 10.5 Hz, 2C, *C*6 and *C*6'), 133.3 (2C, *C*5 and *C*5'), 132.4 (2C, *C*7 and *C*7'), 131.6 (2C, *C*3a and *C*3a'), 125.3 (d, $^3J_{C\text{-}P}$ = 10.7 Hz, 2C, *C*4 and *C*4'), 122.7 (q, $^1J_{C\text{-}F}$ = 286.7 Hz, 2C, 2*C*F_3), 122.2 (q, $^1J_{C\text{-}F}$ = 287.5 Hz, 2C, 2*C*F_3), 40.4 (d, $^1J_{C\text{-}P}$ = 114.2 Hz, P*C*H_2(CH_2)$_2$$CH_3$), 24.8 (d, $^2J_{C\text{-}P}$ = 7.3 Hz, PCH_2*C*$H_2$$CH_2$$CH_3$), 24.2 (d, $^3J_{C\text{-}P}$ = 20.6 Hz, P(CH_2)$_2$*C*$H_2$$CH_3$), 21.8 (d, $^4J_{C\text{-}P}$ = 1.1 Hz, 2C, C6*C*H_3 and C6'*C*H_3), 13.5 (d, $^4J_{C\text{-}P}$ = 1.1 Hz, P(CH_2)$_3$*C*H_3); Carbons 3, 3', 7a, and 7a' were not detected due to the dynamic nature of the compound; ^{19}F{^{1}H} NMR (376 MHz, $CDCl_3$)

δ −74.6 (q, $^4J_{F-F}$ = 9.1 Hz, 3F, CF_3, *cis*-**2a**), −74.7 (q, $^4J_{F-F}$ = 9.2 Hz, 0.18F, CF_3, *trans*-**2a**), −75.1 (q, $^4J_{F-F}$ = 9.6 Hz, 0.18F, CF_3, *trans*-**2a**), −75.9 (q, $^4J_{F-F}$ = 7.8 Hz, 3F, CF_3, *cis*-**2a**); $^{31}P\{^1H\}$ NMR (162 MHz, $CDCl_3$) δ −4.3 ((*cis*)-**2a**); HRMS (ESI$^+$): *m/z* calcd. 601.1160 for ($[M+H]^+$), found 601.1160.

Evaluation of the configurational stabilities

Determination of the isomerisation barriers

The stereoisomerisation of **2a** can be described as a set of three reactions: enantiomerisation between (*S-trans*)-**2a** and (*R-trans*)-**2a**, enantiomerisation between (*R-cis*)-**2a** and (*S-cis*)-**2a**, and irreversible diastereomerisation of (*cis*)-**2a** to (*trans*)-**2a** (Supplementary Figure 1). The reactions were observed separately. As an intramolecular mechanism (*via* Berry pseudorotations) is assumed for all interconversions, all reactions are expected to follow first-order kinetics.

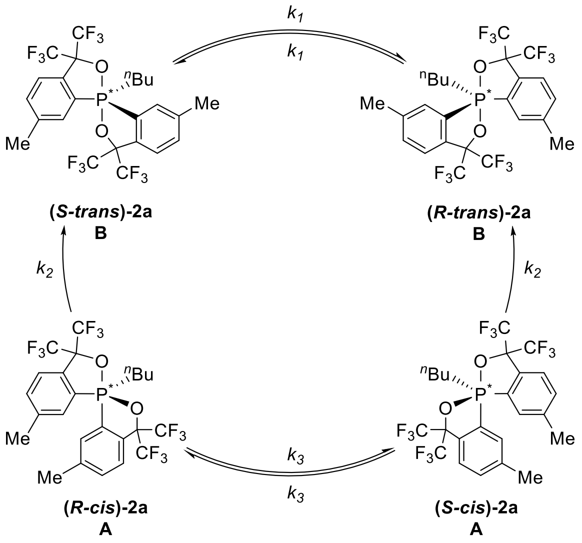

Supplementary Figure 1. Macroscopically observable stereoisomerisation reactions of **2a** with rate constants

Enantiomerisation of (*trans*)-2a

A stirred solution of enantioenriched sample (94:6 e.r., 99:1 d.r., 2.40 mg) of (*S-trans*)-**2a** in *o*-dichlorobenzene (1.5 mL) was heated to 180 °C. Aliquots (10 µL) were withdrawn in regular time intervals, the solvent was removed under a stream of nitrogen, and the residue was redissolved in heptane (90 µL) and analysed by HPLC on a chiral stationary phase (Chiralpak IB-N3 column, 3 µm, 250x4.6 mm, 100% heptane, 1.0 mL/min, 5 °C).

Supplementary Table 5. HPLC data of the isomerisation of (*S-trans*)-**2a** at 180 °C.

Entry	Time/h	ee/%	Entry	Time/h	ee/%	Entry	Time/h	ee/%
1	0	88.0	8	18	62.0	15	28	54.0
2	1	86.0	9	19	60.0	16	30	52.0
3	2	85.2	10	20	58.0	17	48	32.0
4	3	84.0	11	21	62.0	18	50	30.0
5	4	82.0	12	22	60.0	19	63	25.8
6	5	80.0	13	24	58.6	20	65	24.6
7	6	78.0	14	26	56.0			

The change of the enantiomeric excess *ee* of (*S-trans*)-**2a** in time *dt* is given by[16]

$$\frac{d(ee)}{dt} = -2k_1(ee) \quad (1)$$

and integration yields

$$\ln(ee) = -2k_1t + \ln(ee)_0 \quad (2)$$

with (ee_0) being the *ee* at $t = 0$. Fitting the ln(*ee*) values obtained by HPLC analysis to this equation allows for the determination of the rate constant (Supplementary Figure 2).

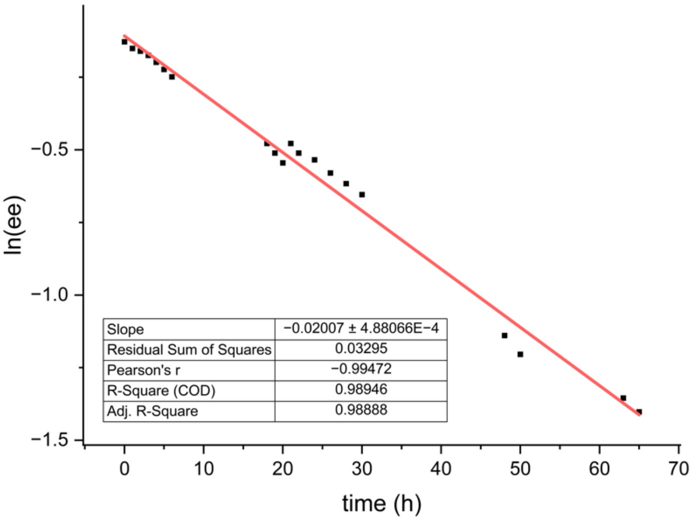

Supplementary Figure 2. Linear regression of the HPLC data of the enantiomerisation of *trans*-**2a**

According to the Eyring equation, the activation free energy is given by

$$\Delta G^{\ddagger} = -RT\ln\left(\frac{k_1h}{k_BT}\right) \quad (3)$$

with the gas constant $R = 8.314\ \mathrm{J/(mol \cdot K)}$, the temperature $T = 453.15\ \mathrm{K}$ (180 °C), Planck constant $h = 6.626 \cdot 10^{-34}\ \mathrm{J \cdot s}$, and the Boltzmann constant $k_B = 1.381 \cdot 10^{-23}\ \mathrm{J/K}$. Inserting the value of $k_1 = 0.010035\ \mathrm{h^{-1}}$ obtained by linear regression yields an activation barrier of $\Delta G^{\ddagger}_{180\,°\mathrm{C}} = 161\ \mathrm{kJ/mol}$ for the enantiomerisation between (*S-trans*)-**2a** and (*R-trans*)-**2a**.

Diastereomerisation of (±-*cis*)-2a to (±-*trans*)-2a

A stirred solution of a diastereomerically enriched sample (4:96 d.r., 2.40 mg) of (±-*cis*)-**2a** in heptane (1.5 mL) was heated to 30 °C. Aliquots (10 µL) were withdrawn in regular time intervals, diluted with heptane (90 µL) and analysed by HPLC on a chiral stationary phase (Chiralpak IB-N3 column, 3 µm, 250x4.6 mm, 100% heptane, 1.0 mL/min, 5 °C, for (±*trans*)-**2a** the sum of *R* and *S* was used).

The change of the concentration of (±-*cis*)-**2a** d[$\boldsymbol{A}$] in time *dt* is given by

$$\frac{d[\boldsymbol{A}]}{dt} = -k_2[\boldsymbol{A}] \quad (4)$$

integration gives

$$\ln[\boldsymbol{A}] = -k_2t + \ln[\boldsymbol{A}]_0 \quad (5)$$

with $[\mathbf{A}]_0$ being the concentration of **A** at $t = 0$. Fitting the HPLC peak areas to this equation allows for the determination of the rate constant (Supplementary Figure 3). Inserting the value of $k_2 = 0.04694\ \mathrm{h^{-1}}$ obtained by

linear regression yields an activation barrier of $\Delta G^{\ddagger}_{30\ °C} = 103\ \mathrm{kJ/mol}$ for the diastereomerisation of (±-*cis*)-**2a** to (±-*trans*)-**2a**.

Supplementary Table 6. HPLC data of the isomerisation of (±-*cis*)-**2a** at 30 °C.

Entry	Time/h	Rel. area (±-*cis*)-2a/%	Entry	Time/h	Rel. area (±-*cis*)-2a/%	Entry	Time/h	Rel. area (±-*cis*)-2a/%
1	0	96.0	15	7	67.0	29	14	48.0
2	0.5	93.0	16	7.5	63.0	30	15	45.0
3	1	90.0	17	8	62.6	31	16	43.0
4	1.5	88.5	18	8.5	60.0	32	17	41.0
5	2	86.0	19	9	59.7	33	18	39.5
6	2.5	82.0	20	9.5	59.9	34	19	37.0
7	3	79.5	21	10	58.0	35	20	36.0
8	3.5	77.0	22	10.5	56.5	36	21	35.7
9	4	78.0	23	11	55.6	37	22	32.0
10	4.5	74.0	24	11.5	53.0	38	23	31.0
11	5	73.0	25	12	52.5	39	24	31.0
12	5.5	72.0	26	12.5	51.0	40	25	30.7
13	6	69.0	27	13	50.8			
14	6.5	66.0	28	13.5	48.5			

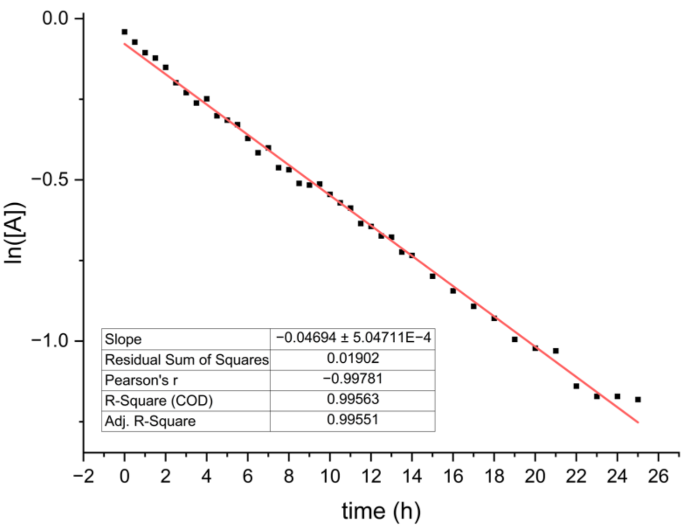

Supplementary Figure 3. Linear regression of the HPLC data of the diastereomerisation of (±-*cis*)-**2a** to (±-*trans*)-**2a**

Enantiomerisation of (*cis*)-2a (by Prof. Dr. Daniel Häussinger)

The barrier to the enantiomerisation of *cis*-**2a** was determined with a coalescence temperature method.[17] NMR spectra were recorded at 600 MHz on a *Bruker Avance III* 600 MHz equipped with a broadband direct observe BBFO probe. Experiments were calibrated using a methanol standard, showing accuracy within ± 0.2 K. The ^{19}F NMR spectra of compounds *cis*-**2a** were measured in toluene-D_8 at different temperatures ranging from 218 K to 298 K.

Four slightly broadened fluorine resonances for the four CF_3-groups were observed at 218 K between –74 and –76 ppm relative to CCl_3F (δ=0.00 ppm). The more downfield shifted pair of resonances underwent coalescence at ca. 235 K, while the upfield shifted pair of resonances collapsed at ca. 253 K. With $\Delta\nu_0$ values of 55.1 and 268 Hz, rsp., energy barriers of 47.6 and 48.1 kJ/mol resulted using modified Eyring equation:

$$\Delta G^{\ddagger} = RT_c \ln \frac{RT_c\sqrt{2}}{\pi N_A h |\nu_A - \nu_B|} e^{-\frac{\Delta G^{\ddagger}}{RT_c}} \qquad (6)$$

Within the uncertainty of ± 1 kJ/mol an average barrier of 47.9 ± 1 kJ/mol can be stated.

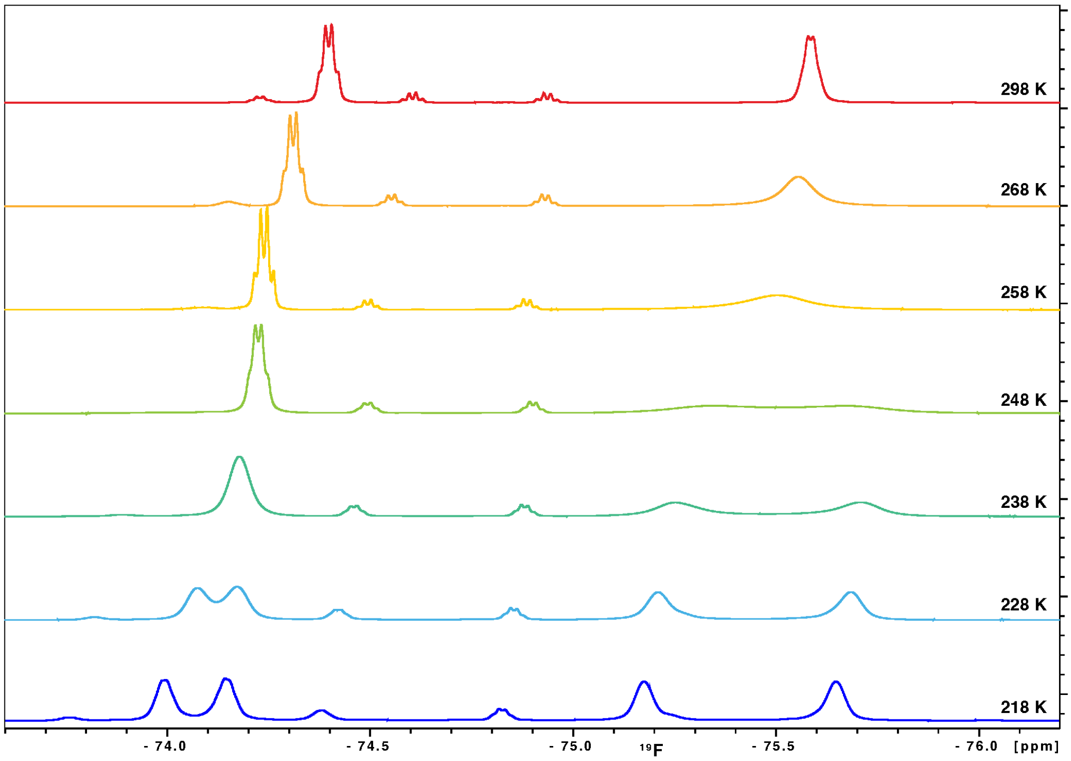

Supplementary Figure 4. ^{19}F VT-NMR (565 MHz, Toluene-d_8) spectra of (*cis*)-**2a**

Determination of the enantiomerisation barrier for 1a (by Prof. Dr. Daniel Häussinger)

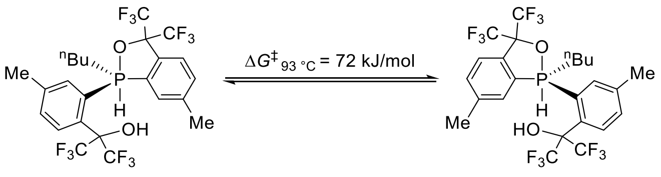

(*R*)-**1a** (*S*)-**1a**

The barrier to the enantiomerisation of **1a** was determined with a coalescence temperature method.[17] Experiments were calibrated using a glycerol standard, showing accuracy within ± 0.2 K. The ^{19}F NMR spectra of compound **1a** were measured in toluene-d_8 at different temperatures ranging from 298 K to 385 K.

Four sharp fluorine resonances for the four CF_3-groups were observed at room temperature between −72 and −77 ppm. Upon heating above 338 K, significant broadening of all four lines was observed and at ca. 366 K the two inner lines showed coalescence and a sharp averaged signal again at 385 K. The two outer lines, in contrast, which have a much larger shift difference, broadened further up to 385 K, but did not reach coalescence in the accessible temperature window. The pairwise assignment was corroborated by ^{19}F-^{19}F-COSY and ^{19}F-^{19}F-EXSY spectra.

With the modified Eyring equation an energy barrier of 72.3 ± 1 kJ/mol was obtained from T_c = 366K and $\Delta\nu_0$ = 153 Hz.

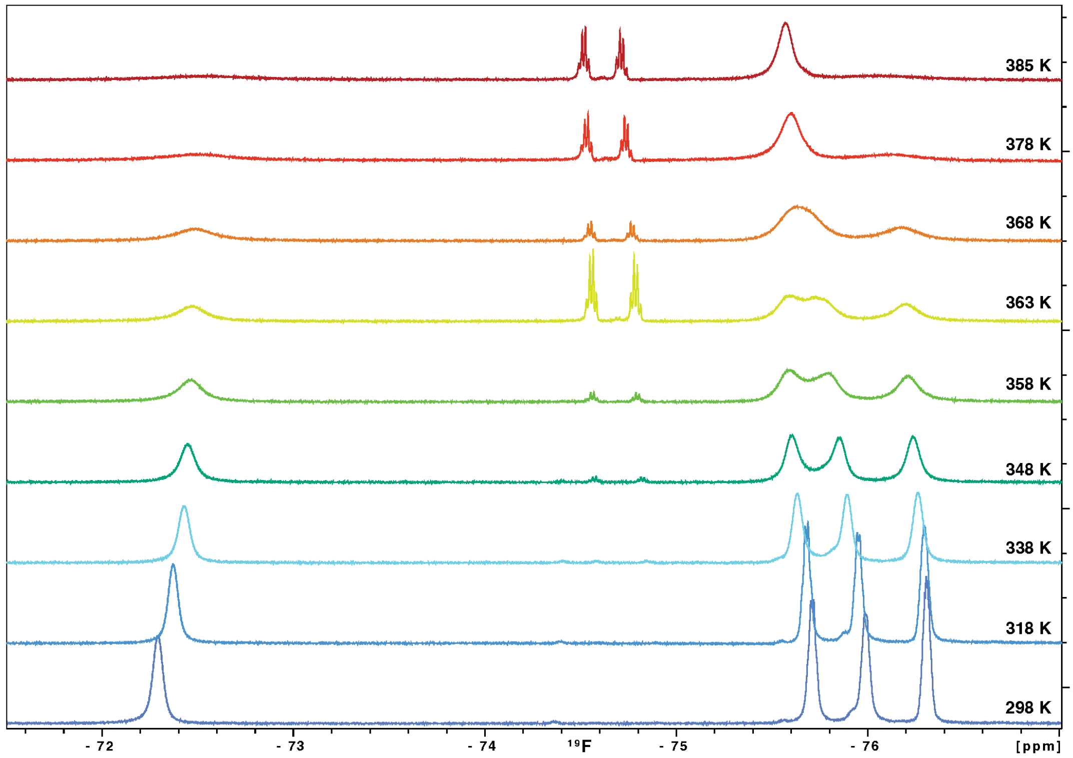

Supplementary Figure 5 ^{19}F VT-NMR (565 MHz, Toluene-d_8) spectra of **1a**

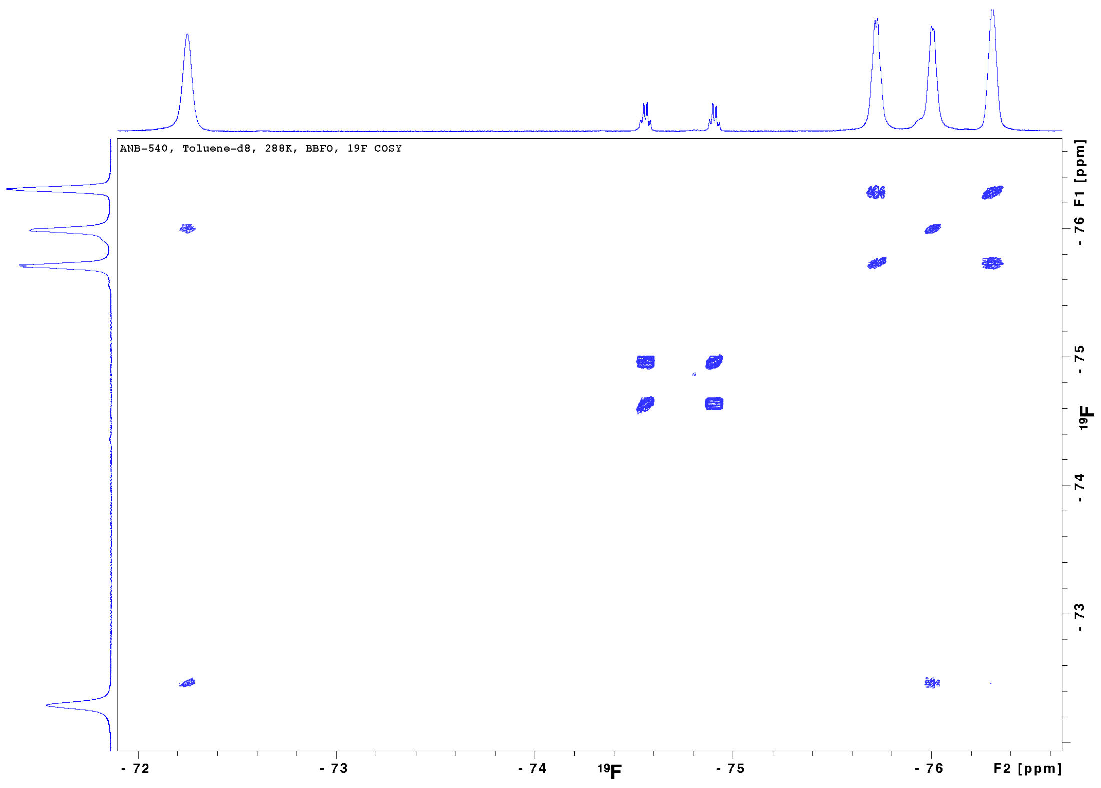

Supplementary Figure 6. ^{19}F-^{19}F-COSY (565 MHz, Toluene-d_8, 15 °C) spectrum of **1a**

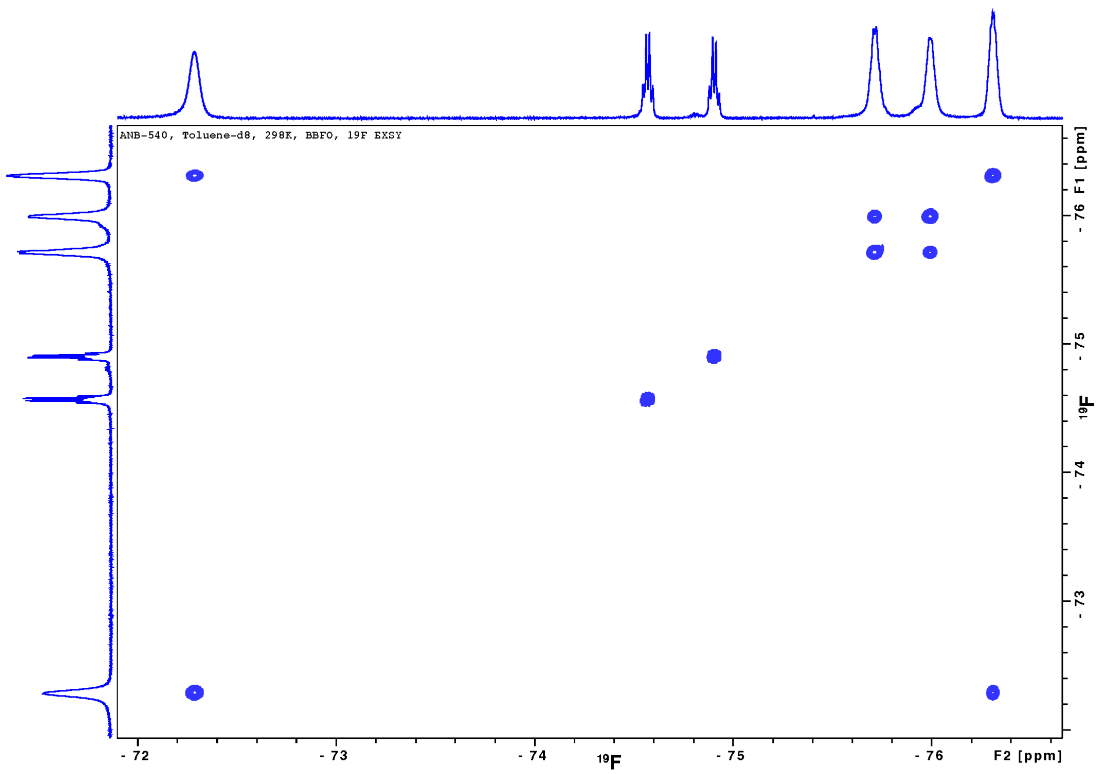

Supplementary Figure 7. ^{19}F-^{19}F-EXSY (565 MHz, Toluene-d_8, 25 °C) spectrum of **1a**

Reactivity of (*cis*)-2a

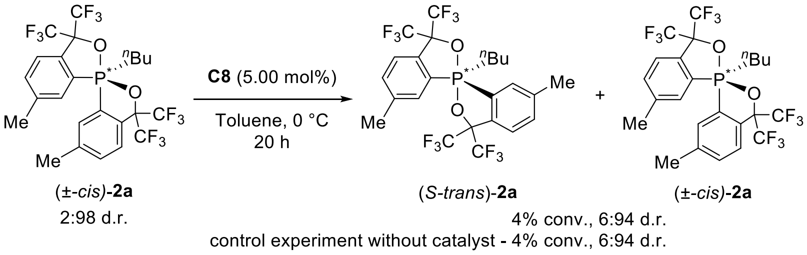

In a 20 mL crimp cap vial, **C8** (0.37 mg, 0.85 µmol, 5.00 mol%) and compound (±-*cis*)-**2a** (obtained by the literature reported procedure, 10.2 mg, 17.0 µmol, 1.00 eq., 2:98 d.r.) were dissolved in toluene (0.85 mL) under an Ar atmosphere. The reaction mixture was stirred at 0 °C for 20 h. The solvent was removed under a stream of nitrogen. The residue was analysed by ^{19}F NMR. The result was compared to a control experiment (reaction without the catalyst under the same conditions), showing the same conversion and change of diastereoselectivity, indicating that the observed reaction is a background reaction. These results suggest that (*cis*)-**2a** does not react under catalytic conditions, indicating that **1a** reacts directly to (*S*-*trans*)-**2a** in a catalytic reaction, without (*cis*)-**2a** being an intermediate.

Detection of hydrogen in the catalytic reaction

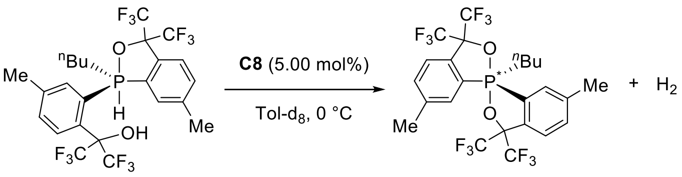

(±)-**1a** (*S*-*trans*)-**2a**

In an NMR tube with J. Young valve, **1a** (10.2 mg, 17.0 µmol, 1.00 eq.) and **C8** (0.37 mg, 0.85 µmol, 5.00 mol%) were dissolved in toluene-d_8 (2.0 mL) and sealed. The reaction was left at 0 °C for 10 h. ^{1}H NMR spectrum was recorded (Bruker Avance III 400 MHz spectrometer at 298 K) and compared to a reference spectrum of H_2 in toluene-d_8, confirming the evolution of H_2 in the catalytic reaction (Supplementary Figure 8).

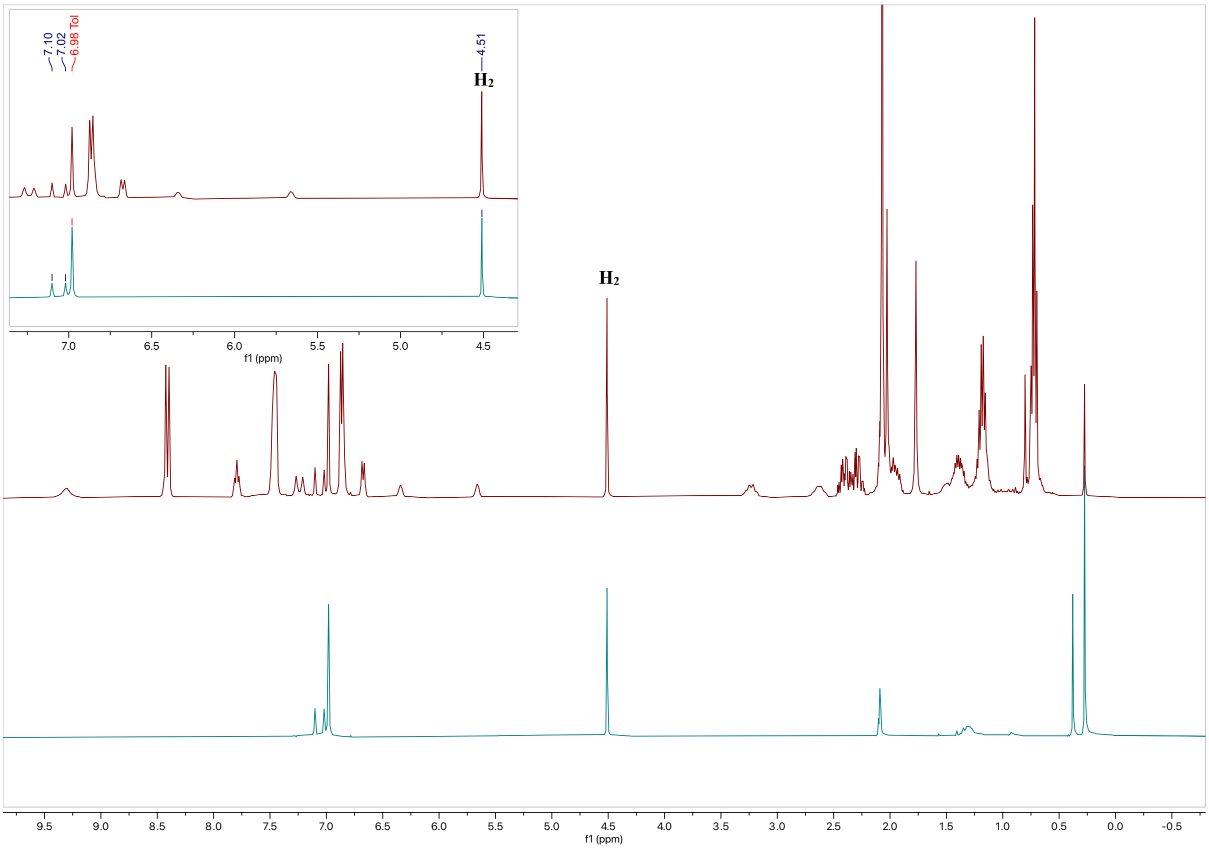

Supplementary Figure 8. Detection of hydrogen evolution in the catalytic reaction. Spectrum of the catalytic reaction (top); reference spectrum of H_2 in toluene-d_8 (bottom) (400 MHz, Tol-d_8, 25 °C)

X-ray crystallographic analysis (by Dr. Alessandro Prescimone)

[*TBPY*-5-11'-*A*]-1-Butyl-6,6'-dimethyl-3,3,3',3'-tetrakis(trifluoromethyl)-3*H*,3'*H*-1λ^5,1'-spirobi[benzo[*c*][2,1]oxaphosphole] ((*S*-*trans*)-2a)

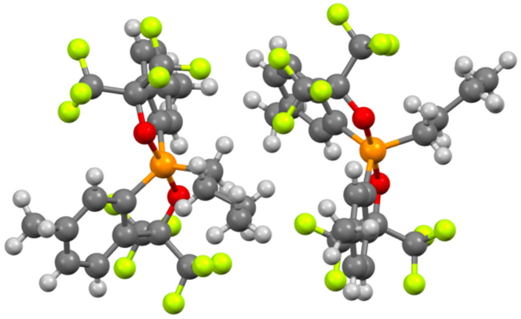

CCDC 2255853

Experimental. Single colourless plate-shaped crystals of (*S*-*trans*)-**2a** were recrystallised from pentane by solvent layering. A suitable crystal with dimensions 0.20 x 0.15 x 0.05 mm^3 was selected and mounted on a MITIGEN holder in perfluoroether oil on a STOE STADIVARI diffractometer. The crystal was kept at a steady T = 150 K during data collection. The structure was solved with the **olex2.solve** 1.5[18] solution program using iterative methods and by using Olex2 1.5[19] as the graphical interface. The model was refined with ShelXL 2018/3[20] using full matrix least squares minimisation on F^2.

Crystal Data. $C_{24}H_{21}F_{12}O_2P$, M_r = 600.38, triclinic, *P*1 (No. 1), a = 9.9642 (2) Å, b = 10.3777(2) Å, c = 16.0289(6) Å, α = 74.308(2)°, β = 69.738(2)°, g = 66.859(2)°, V = 1266.27(6) Å^3, T = 150 K, Z = 2, Z' = 2, m(Cu K$_a$) = 1.286, 38640 reflections measured, 7545 unique (R_{int} = 0.0238) which were used in all calculations. The final wR_2 was 0.0995 (all data) and R_1 was 0.0375 (I≥2 s(I)).

**Compound**	**(*S*-*trans*)-2a**
**Formula**	$C_{24}H_{21}F_{12}O_2P$
**D_{calc}/g cm^{-3}**	1.575
**m/mm^{-1}**	1.286
**Formula Weight**	600.38
**Colour**	colourless
**Shape**	plate-shaped
**Size/mm^3**	0.20×0.15×0.05
**T/K**	150
**Crystal System**	triclinic
**Flack Parameter**	0.02(2)
**Hooft Parameter**	0.006(4)
**Space Group**	*P*1

***a*/Å**	9.9642(2)
***b*/Å**	10.3777(2)
***c*/Å**	14.3710(4)
***a*/°**	74.308(2)
***b*/°**	69.738(2)
***g*/°**	66.859(2)
**V/Å^3**	1266.27(6)
Z	2
Z'	2
**Wavelength/Å**	1.34143
**Radiation type**	Cu K$_a$
**Q_{min}/°**	2.887
**Q_{max}/°**	55.490
**Measured Refl's.**	38640
**Indep't Refl's**	7545
**Refl's I≥2 *s*(I)**	7426
**R_{int}**	0.0238
**Parameters**	710
**Restraints**	3
**Largest Peak**	0.340
**Deepest Hole**	-0.380
**GooF**	1.016
**wR_2 (all data)**	0.0995
**wR_2**	0.0989
**R_1 (all data)**	0.0381
**R_1**	0.0375

Structure Quality Indicators

**Reflections:**	d min (Ga) 2Θ=111.0° 0.81	I/σ(I) 62.9	Rint 2.38%	Full 107.2° 99% to 111.0° 99.7
**Refinement:**	Shift 0.000	Max Peak 0.3	Min Peak -0.4	GooF 1.016	Hooft .006(4)

A colourless plate-shaped-shaped crystal with dimensions 0.20 × 0.15 × 0.05 mm^3 was mounted on a MITIGEN holder in perfluoroether oil. Data were collected using a STOE STADIVARI diffractometer operating at *T* = 150 K.

Data were measured using rotation method, *w* scans with Gu K$_a$ radiation. The diffraction pattern was indexed and the total number of runs and images was based on the strategy calculation from the program X-Area

Pilatus3_SV 1.31.186.0 (STOE, 2022). The maximum resolution that was achieved was Q = 55.490º (0.81 Å).

The unit cell was refined using X-Area Integrate 2.5.3.0 (STOE, 2021)X-Area LANA 2.7.5.0 (STOE, 2022) on 124910 reflections, 323% of the observed reflections.

Data reduction, scaling and absorption corrections were performed using X-Area Integrate 2.5.3.0 (STOE, 2021)X-Area LANA 2.7.5.0 (STOE, 2022). The final completeness is 99.70 % out to 55.490º in Q. A multi-scan absorption correction was performed using STOE X-Red32, absorption correction by Gaussian integration, analogous to Coppens[21]. Afterwards scaling of reflection intensities was performed within STOE LANA.[22] Finally, a spherical absorption correction was done within STOE LANA. The absorption coefficient m of this material is 1.286 mm^{-1} at this wavelength (l = 1.34143 Å) and the minimum and maximum transmissions are 0.297 and 0.449.

The structure was solved and the space group P1 (# 1) determined by the olex2.solve 1.5[18] solution program using using iterative methods and refined by full matrix least squares minimisation on $\boldsymbol{F^2}$ using version 2018/3 of **ShelXL** 2018/3[20]. All non-hydrogen atoms were refined anisotropically. Hydrogen atom positions were calculated geometrically and refined using the riding model. Hydrogen atom positions were calculated geometrically and refined using the riding model.

The value of Z' is 2. This means that there are two independent molecules in the asymmetric unit.

The Flack parameter was refined to 0.02(2). Determination of absolute structure using Bayesian statistics on Bijvoet differences using the Olex2 results in 0.006(4). Note: The Flack parameter is used to determine chirality of the crystal studied, the value should be near 0, a value of 1 means that the stereochemistry is wrong and the model should be inverted. A value of 0.5 means that the crystal consists of a racemic mixture of the two enantiomers.

HPLC Traces

[*TBPY*-5-11'-*A*]-1-Butyl-6,6'-dimethyl-3,3,3',3'-tetrakis(trifluoromethyl)-3*H*,3'*H*-1λ^5,1'-spirobi[benzo[*c*][2,1]oxaphosphole] ((*S-trans*)-2a)

HPLC conditions: Chiralpak IB-N3 (heptane, 100%), Flow: 0.50 mLmin^{-1}, Temp: 5 °C

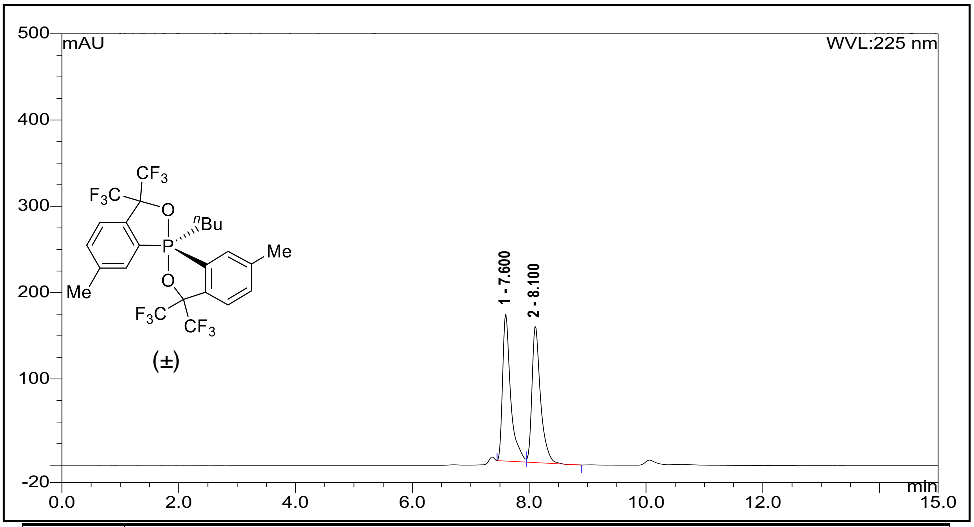

No.	Ret.Time min	Peak Name	Height mAU	Area mAU*min	Rel.Area %
1	7.60	n.a.	170.368	27.157	50.35
2	8.10	n.a.	157.679	26.777	49.65
**Total:**			328.047	53.935	100.00

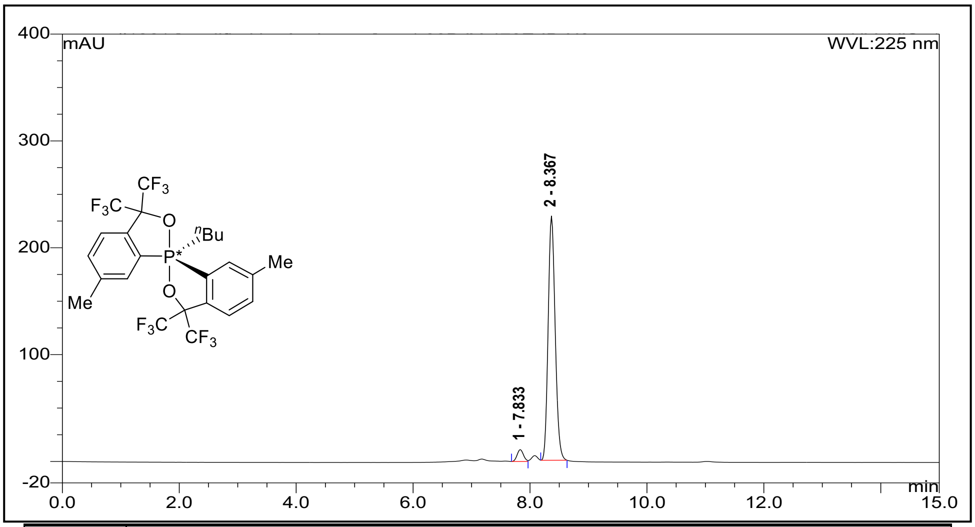

No.	Ret.Time min	Peak Name	Height mAU	Area mAU*min	Rel.Area %
1	7.83	n.a.	11.130	1.392	4.20
2	8.37	n.a.	228.615	31.774	95.80
**Total:**			239.746	33.167	100.00

Supplementary Figure 9. HPLC trace of compound (*S-trans*)-**2a**

[*TBPY*-5-11'-*A*]-1-Butyl-3,3,3',3'-tetrakis(trifluoromethyl)-3*H*,3'*H*-1λ^5,1'-spirobi[benzo[*c*][2,1]oxaphosphole] ((*S*-*trans*)-2b)

HPLC conditions: Chiralpak IB-N3 (heptane, 100%), Flow: 0.50 mLmin^{-1}, Temp: 5 °C

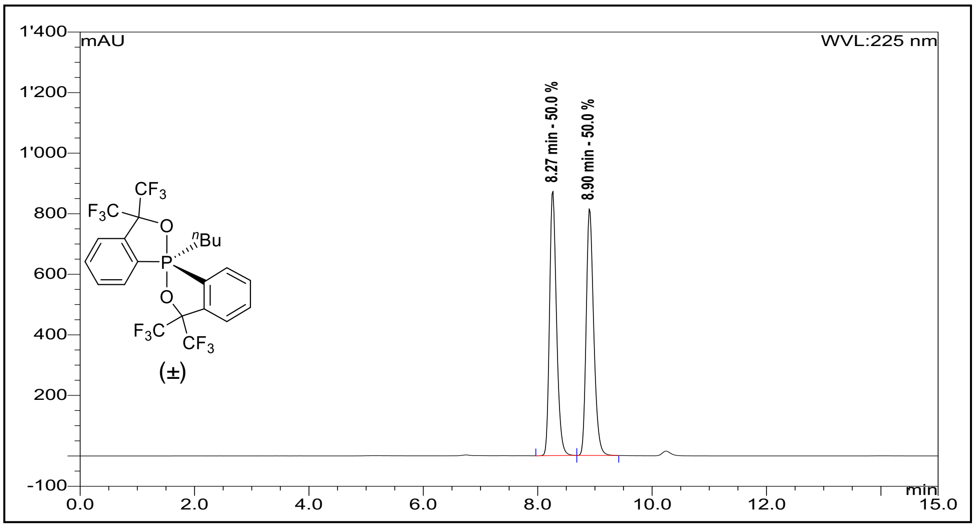

No.	Ret.Time min	Peak Name	Height mAU	Area mAU*min	Rel.Area %
1	8.27	n.a.	873.318	123.430	49.99
2	8.90	n.a.	814.774	123.467	50.01
**Total:**			1688.093	246.897	100.00

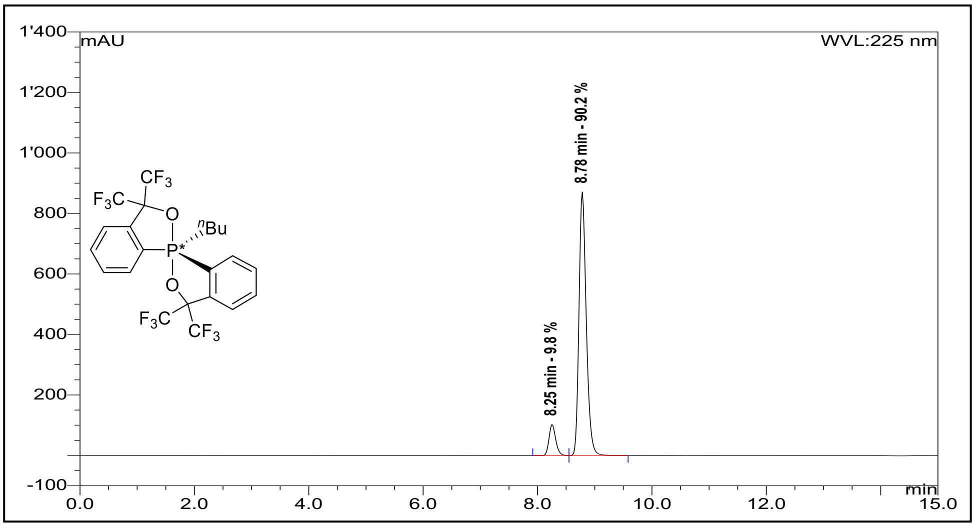

No.	Ret.Time min	Peak Name	Height mAU	Area mAU*min	Rel.Area %
1	8.25	n.a.	102.798	14.012	9.84
2	8.78	n.a.	871.273	128.344	90.16
**Total:**			974.071	142.356	100.00

Supplementary Figure 10. HPLC trace of compound (*S*-*trans*)-**2b**

[*TBPY*-5-11'-*A*]-1-Butyl-5,5'-dimethyl-3,3,3',3'-tetrakis(trifluoromethyl)-3*H*,3'*H*-1λ^5,1'-spirobi[benzo[*c*][2,1]oxaphosphole] ((*S-trans*)-2c)

HPLC conditions: Chiralpak IB-N3 (heptane, 100%), Flow: 0.50 mLmin^{-1}, Temp: 5 °C

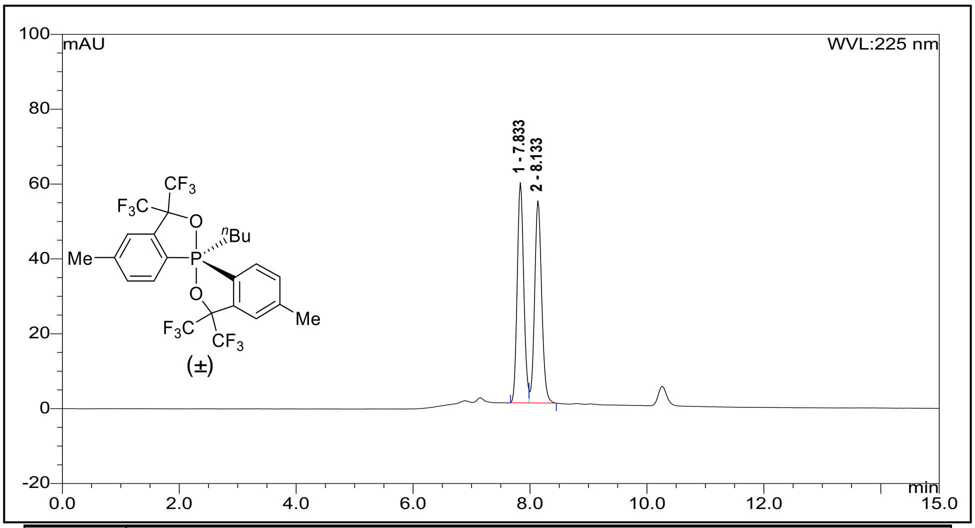

No.	Ret.Time min	Peak Name	Height mAU	Area mAU*min	Rel.Area %
1	7.83	n.a.	58.828	7.580	50.09
2	8.13	n.a.	54.016	7.552	49.91
**Total:**			112.845	15.132	100.00

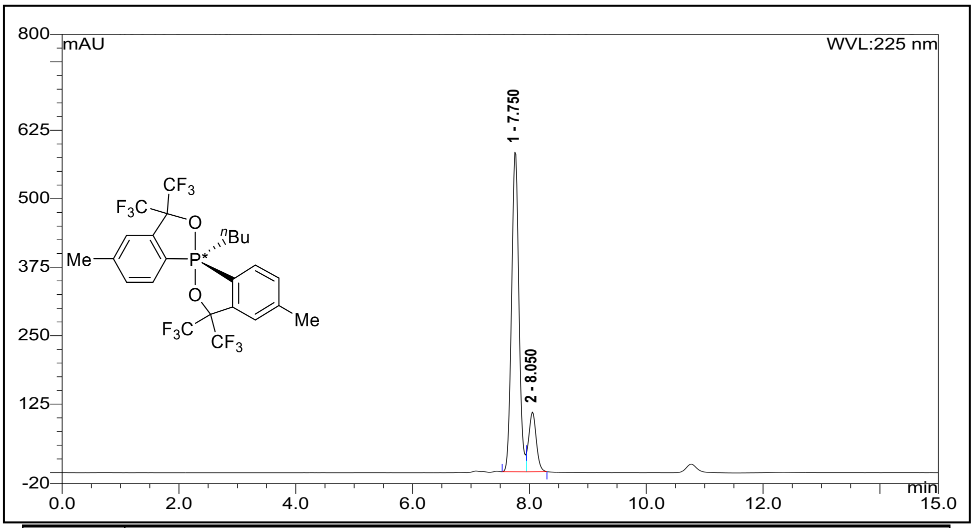

No.	Ret.Time min	Peak Name	Height mAU	Area mAU*min	Rel.Area %
1	7.75	n.a.	583.316	85.170	84.19
2	8.05	n.a.	109.169	15.996	15.81
**Total:**			692.486	101.166	100.00

Supplementary Figure 11. HPLC trace of compound (*S-trans*)-**2c**

[*TBPY*-5-11'-*A*]-1-Butyl-5,5'-dimethoxy-3,3,3',3'-tetrakis(trifluoromethyl)-3*H*,3'*H*-1λ^5,1'-spirobi[benzo[*c*][2,1]oxaphosphole] ((*S*-*trans*)-2d)

HPLC conditions: Chiralpak IB-N3 (heptane, 100%), Flow: 0.25 mLmin^{-1}, Temp: 5 °C

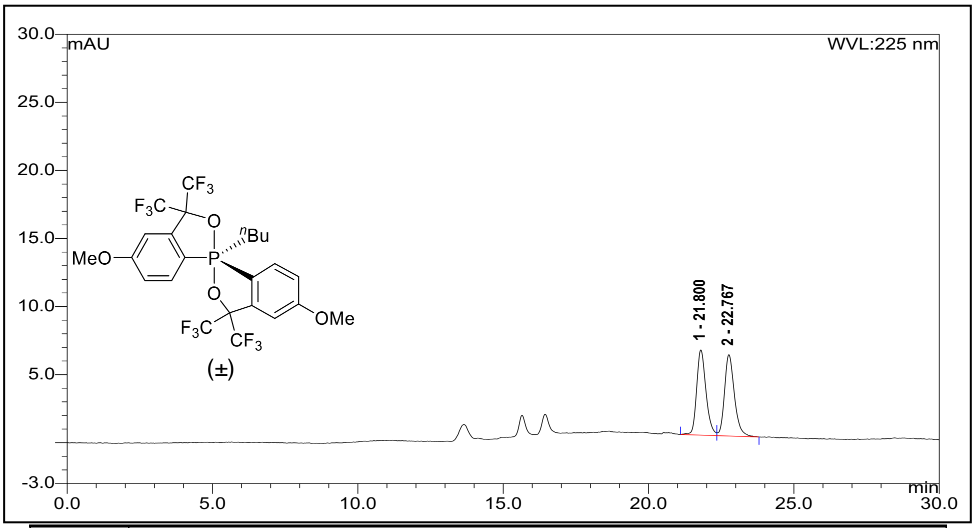

No.	Ret.Time min	Peak Name	Height mAU	Area mAU*min	Rel.Area %
1	21.80	n.a.	6.262	2.335	49.67
2	22.77	n.a.	5.976	2.366	50.33
**Total:**			12.237	4.701	100.00

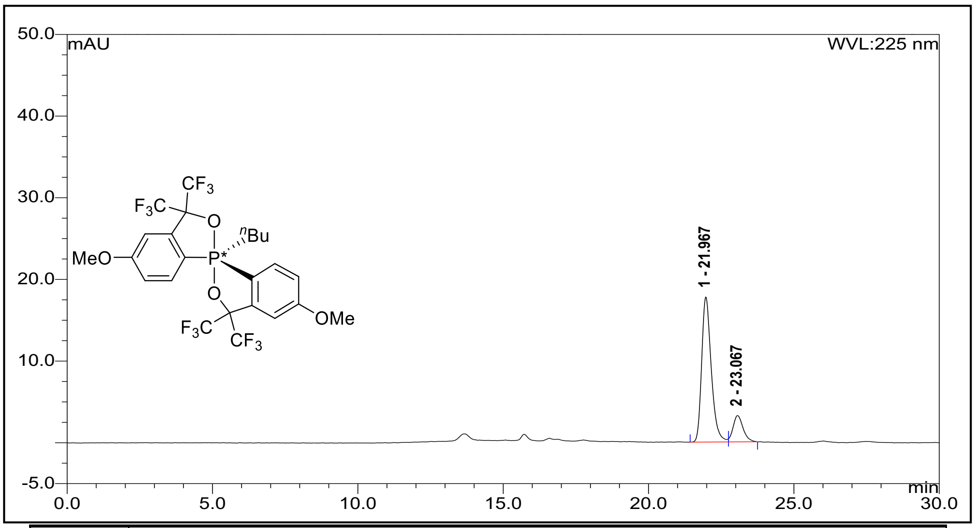

No.	Ret.Time min	Peak Name	Height mAU	Area mAU*min	Rel.Area %
1	21.97	n.a.	17.771	6.777	84.05
2	23.07	n.a.	3.228	1.286	15.95
**Total:**			20.999	8.063	100.00

Supplementary Figure 12. HPLC trace of compound (*S*-*trans*)-**2d**

[*TBPY*-5-11'-*A*]-1-Butyl-5,5'-dimethoxy-6,6'-dimethyl-3,3,3',3'-tetrakis(trifluorome-thyl)-3*H*,3'*H*-1λ^{5},1'-spirobi[benzo[*c*][2,1]oxaphosphole] ((*S*-*trans*)-2e)

HPLC conditions: Chiralpak IB-N3 (heptane, 100%), Flow: 0.20 mLmin^{-1}, Temp: 5 °C

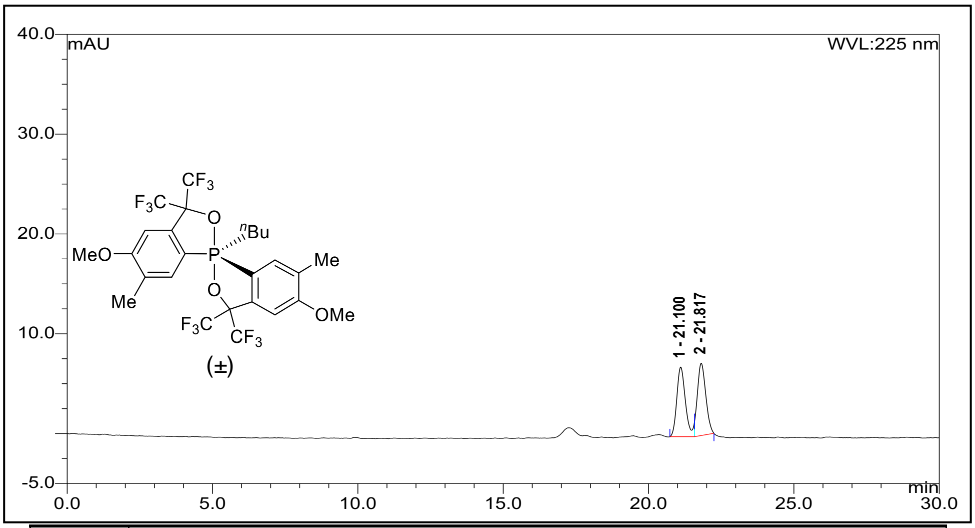

No.	Ret.Time min	Peak Name	Height mAU	Area mAU*min	Rel.Area %
1	21.10	n.a.	6.974	2.412	50.01
2	21.82	n.a.	7.244	2.411	49.99
**Total:**			14.218	4.823	100.00

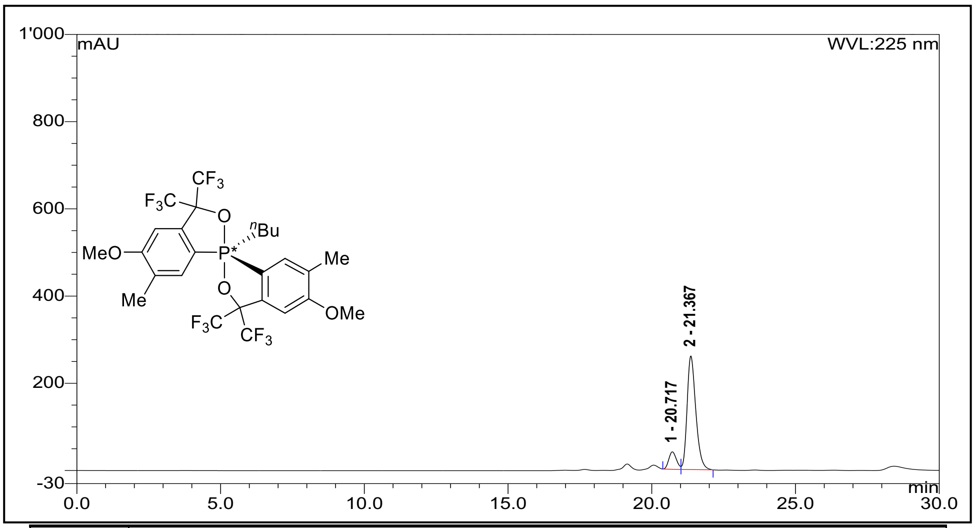

No.	Ret.Time min	Peak Name	Height mAU	Area mAU*min	Rel.Area %
1	20.72	n.a.	40.078	12.380	11.89
2	21.37	n.a.	260.462	91.742	88.11
**Total:**			300.540	104.122	100.00

Supplementary Figure 13. HPLC trace of compound (*S*-*trans*)-**2e**

[*TBPY*-5-11'-*A*]-1-Butyl-3,3,3',3'-tetrakis(trifluoromethyl)-3*H*,3'*H*-1λ[5],1'-spirobi[naphtho[2,3-*c*][2,1]oxaphosphole] ((*S*-*trans*)-2f)

HPLC conditions: Chiralpak IB-N3 (heptane, 100%), Flow: 0.50 mLmin^{-1}, Temp: 5 °C

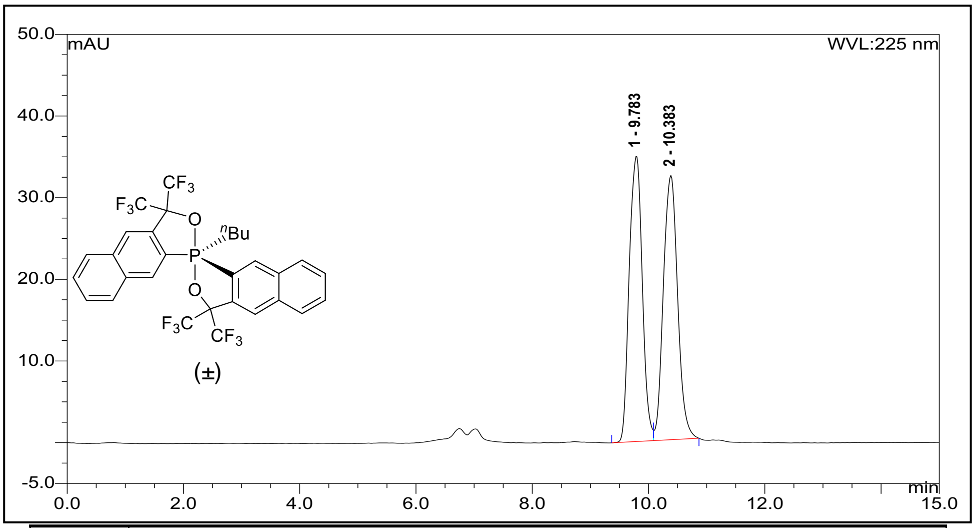

No.	Ret.Time min	Peak Name	Height mAU	Area mAU*min	Rel.Area %
1	9.78	n.a.	34.914	9.071	49.64
2	10.38	n.a.	32.357	9.204	50.36
**Total:**			67.272	18.276	100.00

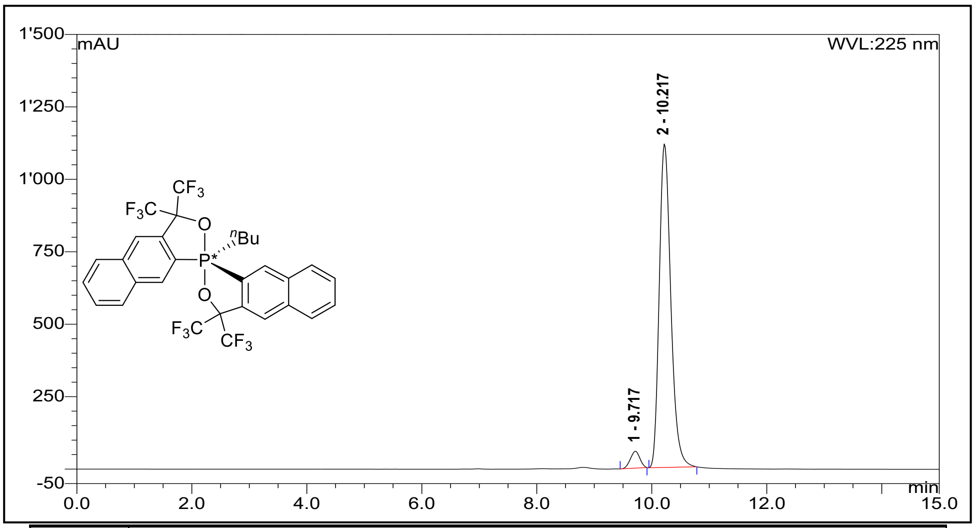

No.	Ret.Time min	Peak Name	Height mAU	Area mAU*min	Rel.Area %
1	9.72	n.a.	58.412	11.139	4.11
2	10.22	n.a.	1116.327	260.221	95.89
**Total:**			1174.739	271.360	100.00

Supplementary Figure 14. HPLC trace of compound (*S*-*trans*)-**2f**

[*TBPY*-5-11'-*A*]-1-Butyl-7,7'-dimethoxy-3,3,3',3'-tetrakis(trifluoromethyl)-3*H*,3'*H*-1λ^5,1'-spiro-bi[naphtho[2,3-*c*][2,1]oxaphosphole] ((*S-trans*)-2g)

HPLC conditions: Chiralpak IB-N3 (heptane, 100%), Flow: 0.20 mLmin^{-1}, Temp: 5 °C

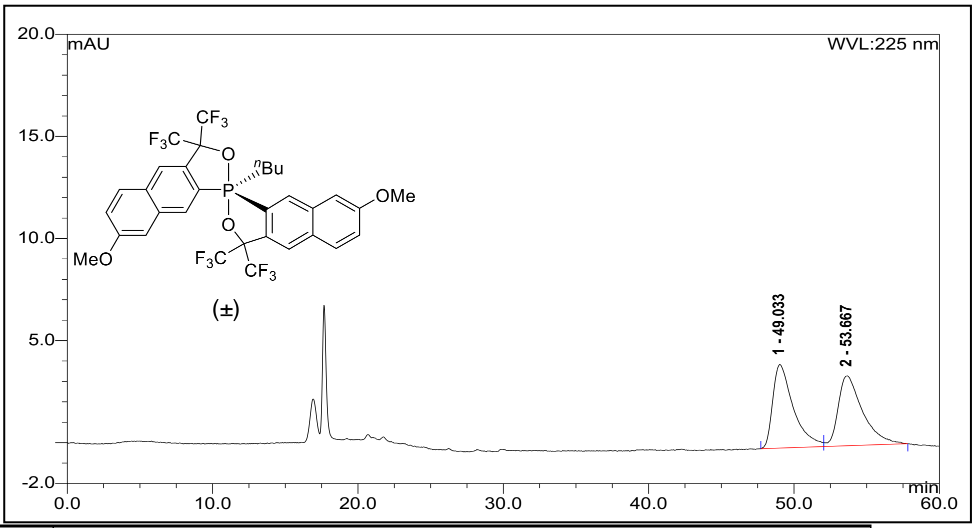

No.	Ret.Time min	Peak Name	Height mAU	Area mAU*min	Rel.Area %
1	49.03	n.a.	4.094	6.711	50.17
2	53.67	n.a.	3.421	6.667	49.83
**Total:**			7.515	13.378	100.00

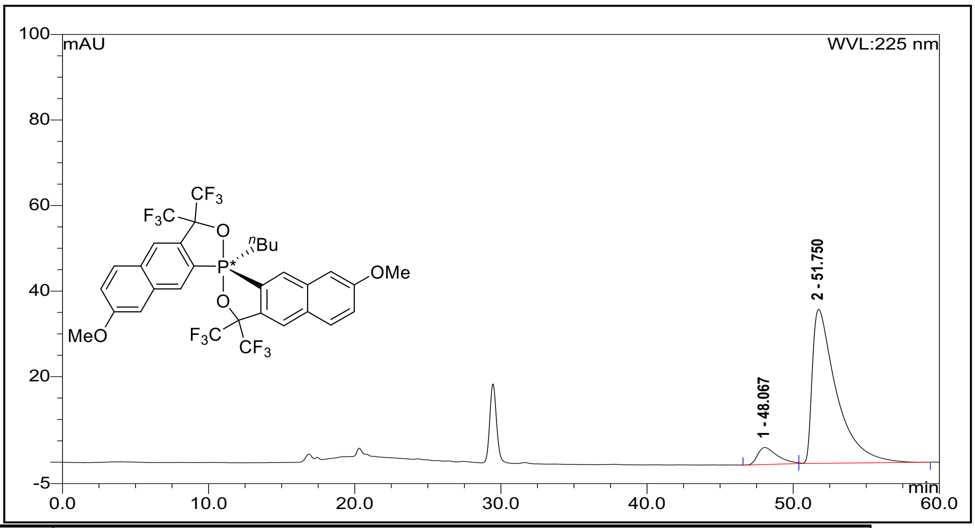

No.	Ret.Time min	Peak Name	Height mAU	Area mAU*min	Rel.Area %
1	48.07	n.a.	3.953	6.146	8.19
2	51.75	n.a.	35.983	68.908	91.81
**Total:**			39.936	75.054	100.00

Supplementary Figure 15. HPLC trace of compound (*S*-*trans*)-**2g**

[*TBPY*-5-11'-*A*]-1-Hexyl-6,6'-dimethyl-3,3,3',3'-tetrakis(trifluoromethyl)-3*H*,3'*H*-1λ^5,1'-spirobi[benzo[*c*][2,1]oxaphosphole] ((*S-trans*)-2h)

HPLC conditions: Chiralpak IB-N3 (heptane, 100%), Flow: 0.50 mLmin^{-1}, Temp: 5 °C

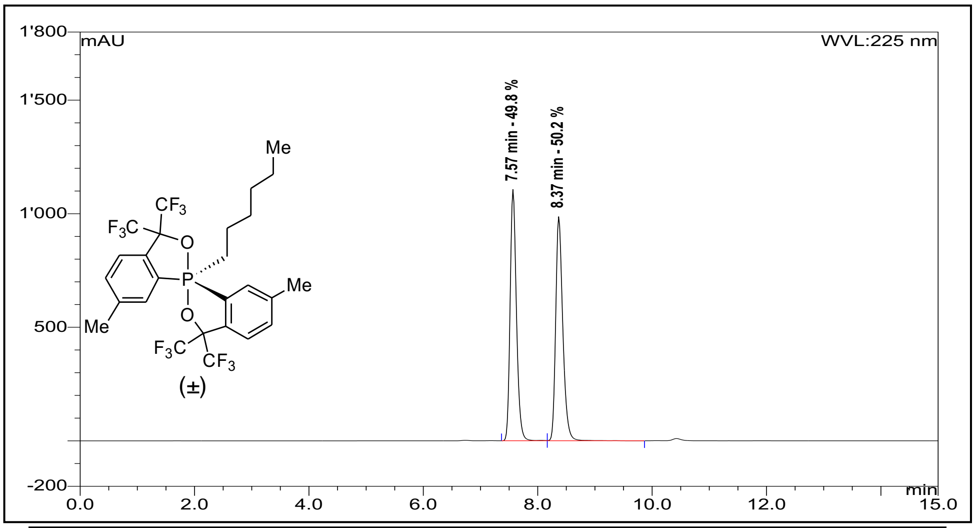

No.	Ret.Time min	Peak Name	Height mAU	Area mAU*min	Rel.Area %
1	7.57	n.a.	1106.335	141.603	49.77
2	8.37	n.a.	986.656	142.883	50.23
**Total:**			2092.991	284.486	100.00

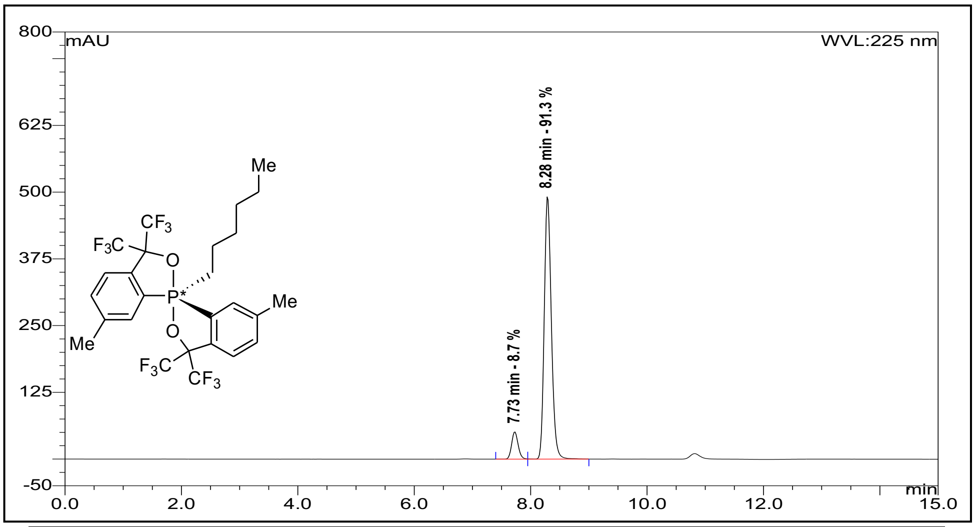

No.	Ret.Time min	Peak Name	Height mAU	Area mAU*min	Rel.Area %
1	7.73	n.a.	50.946	6.601	8.65
2	8.28	n.a.	491.241	69.675	91.35
**Total:**			542.187	76.276	100.00

Supplementary Figure 16. HPLC trace of compound (*S-trans*)-**2h**

[*TBPY*-5-11'-*A*]-6,6'-Dimethyl-1-(5-methylhexyl)-3,3,3',3'-tetrakis(trifluoromethyl)-3*H*,3'*H*-1λ[5],1'-spiro-bi[benzo[*c*][2,1]oxaphosphole] ((*S-trans*)-2i)

HPLC conditions: Chiralpak IB-N3 (heptane, 100%), Flow: 0.50 mLmin^{-1}, Temp: 5 °C

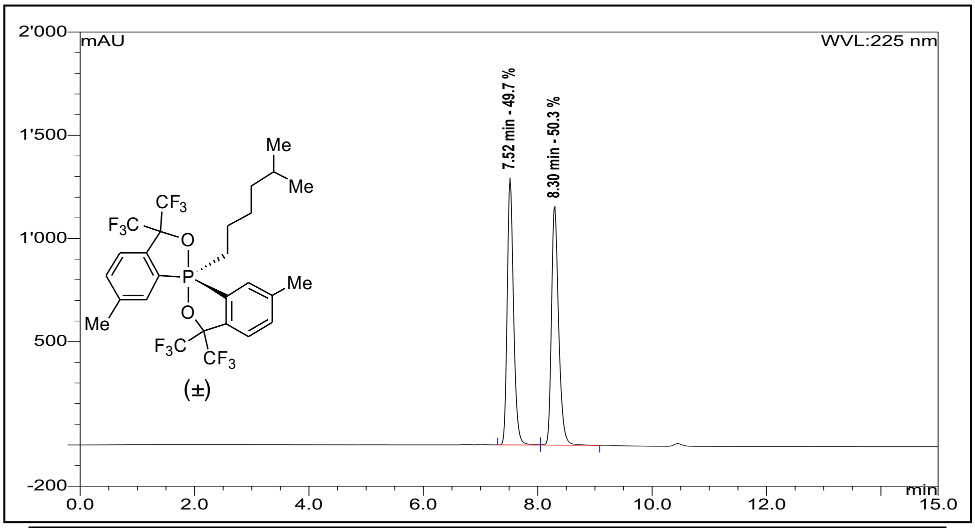

No.	Ret.Time min	Peak Name	Height mAU	Area mAU*min	Rel.Area %
1	7.52	n.a.	1293.889	166.840	49.72
2	8.30	n.a.	1155.959	168.740	50.28
**Total:**			2449.849	335.580	100.00

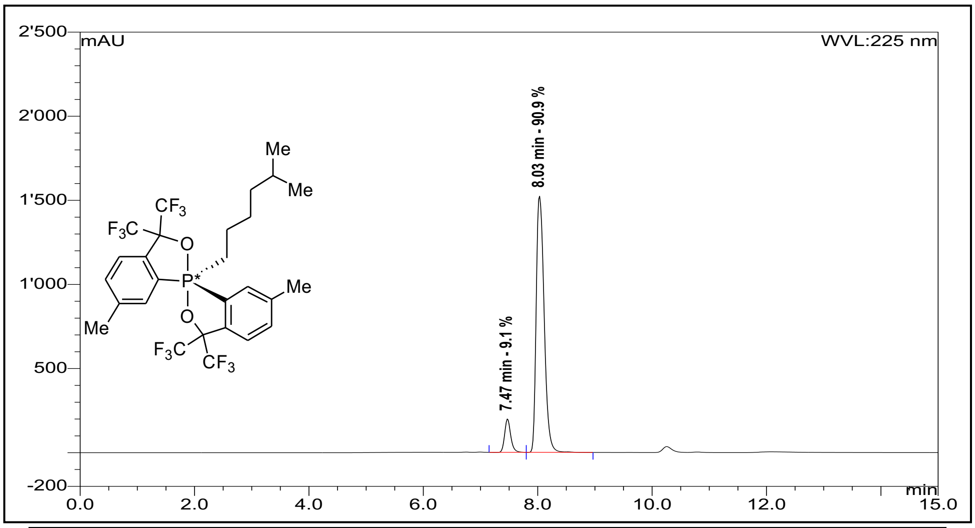

No.	Ret.Time min	Peak Name	Height mAU	Area mAU*min	Rel.Area %
1	7.47	n.a.	198.593	24.418	9.06
2	8.03	n.a.	1522.906	245.103	90.94
**Total:**			1721.499	269.521	100.00

Supplementary Figure 17. HPLC trace of compound (*S-trans*)-**2i**

[*TBPY*-5-11'-*A*]-6,6'-Dimethyl-1-(4-phenylbutyl)-3,3,3',3'-tetrakis(trifluoromethyl)-3*H*,3'*H*-1λ[5],1'-spiro-bi[benzo[*c*][2,1]oxaphosphole] ((*S*-*trans*)-2j)

HPLC conditions: Chiralpak IB-N3 (heptane, 100%), Flow: 0.50 mLmin^{-1}, Temp: 5 °C

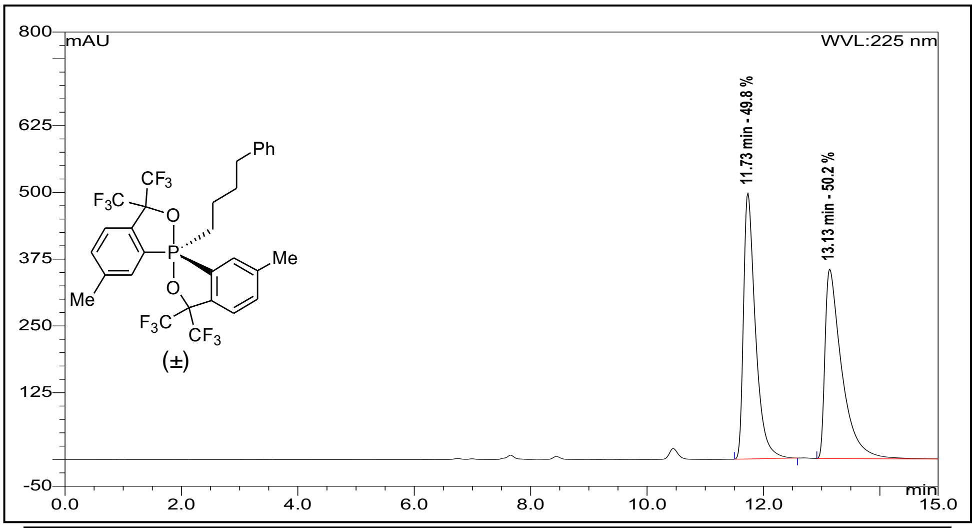

No.	Ret.Time min	Peak Name	Height mAU	Area mAU*min	Rel.Area %
1	11.73	n.a.	498.131	117.993	49.82
2	13.13	n.a.	354.918	118.860	50.18
**Total:**			853.050	236.852	100.00

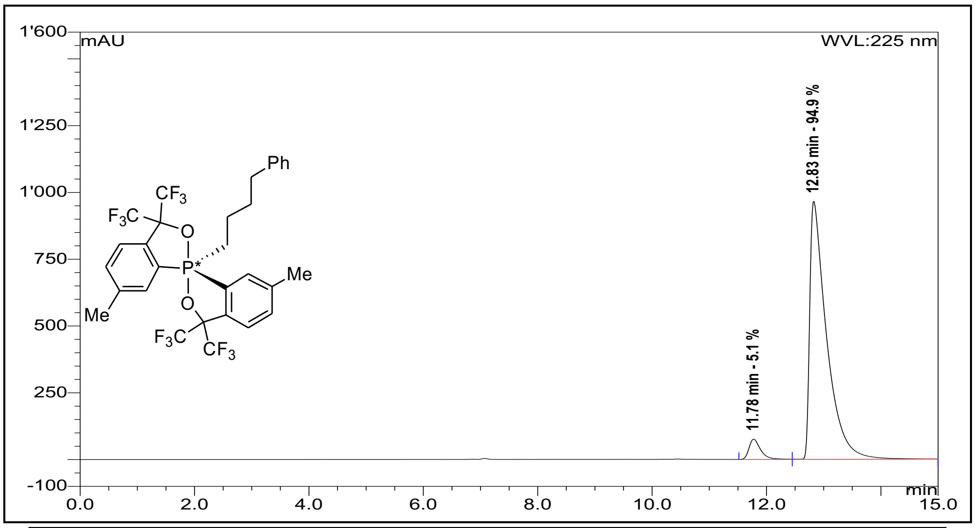

No.	Ret.Time min	Peak Name	Height mAU	Area mAU*min	Rel.Area %
1	11.78	n.a.	75.856	17.874	5.15
2	12.83	n.a.	965.266	329.352	94.85
**Total:**			1041.122	347.226	100.00

Supplementary Figure 18. HPLC trace of compound (*S*-*trans*)-**2j**

[*TBPY*-5-11'-*A*]-6,6'-Dimethyl-1-(pent-4-en-1-yl)-3,3,3',3'-tetrakis(trifluoromethyl)-3*H*,3'*H*-1λ⁵,1'-spiro-bi[benzo[*c*][2,1]oxaphosphole] ((*S-trans*)-2k)

HPLC conditions: Chiralpak IB-N3 (heptane, 100%), Flow: 0.50 mLmin^{-1}, Temp: 5 °C

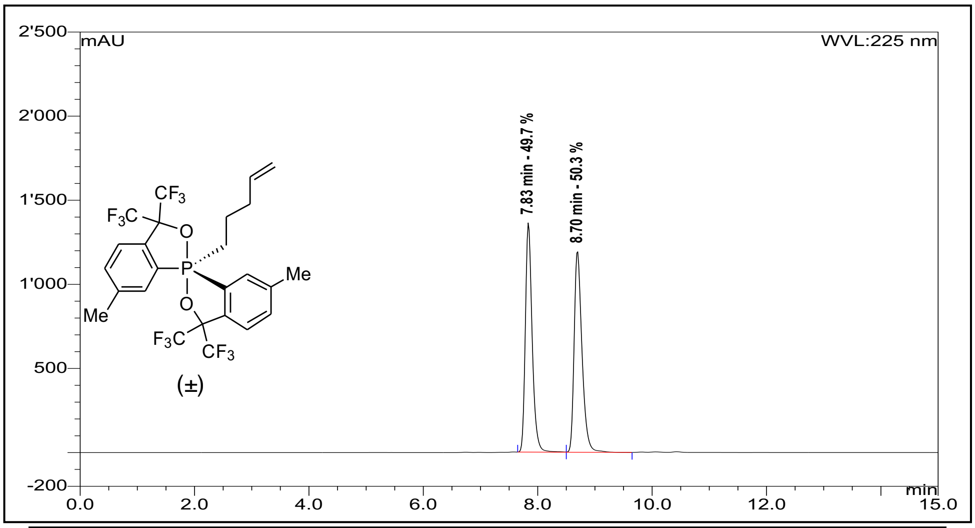

No.	Ret.Time min	Peak Name	Height mAU	Area mAU*min	Rel.Area %
1	7.83	n.a.	1362.901	187.731	49.72
2	8.70	n.a.	1192.234	189.831	50.28
**Total:**			2555.135	377.562	100.00

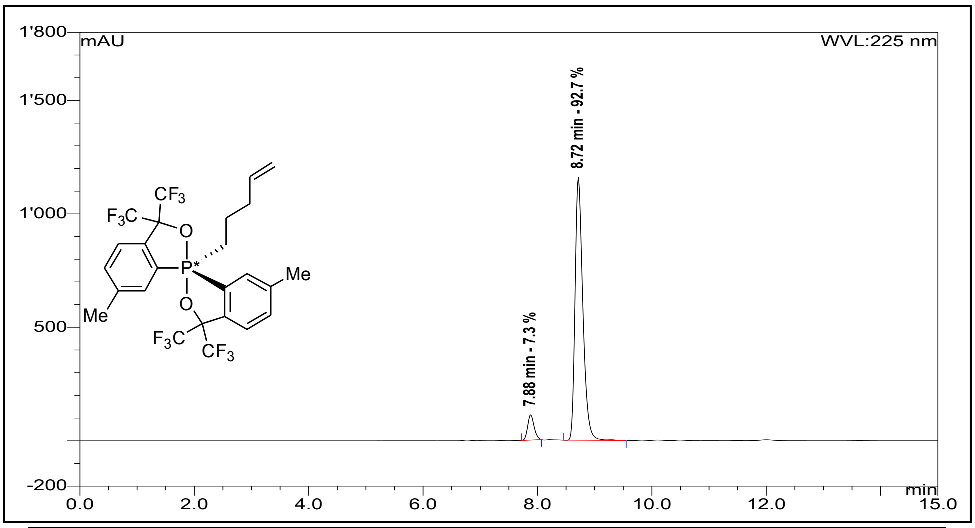

No.	Ret.Time min	Peak Name	Height mAU	Area mAU*min	Rel.Area %
1	7.88	n.a.	111.660	14.429	7.31
2	8.72	n.a.	1160.188	182.913	92.69
**Total:**			1271.848	197.342	100.00

Supplementary Figure 19. HPLC trace of compound (*S-trans*)-**2k**

[*TBPY*-5-11'-*A*]-6,6'-Dimethyl-1-phenethyl-3,3,3',3'-tetrakis(trifluoromethyl)-3*H*,3'*H*-1λ^5,1'-spiro-bi[benzo[*c*][2,1]oxaphosphole] ((*S-trans*)-2l)

HPLC conditions: Chiralpak IB-N3 (heptane, 100%), Flow: 0.50 mLmin^{-1}, Temp: 5 °C

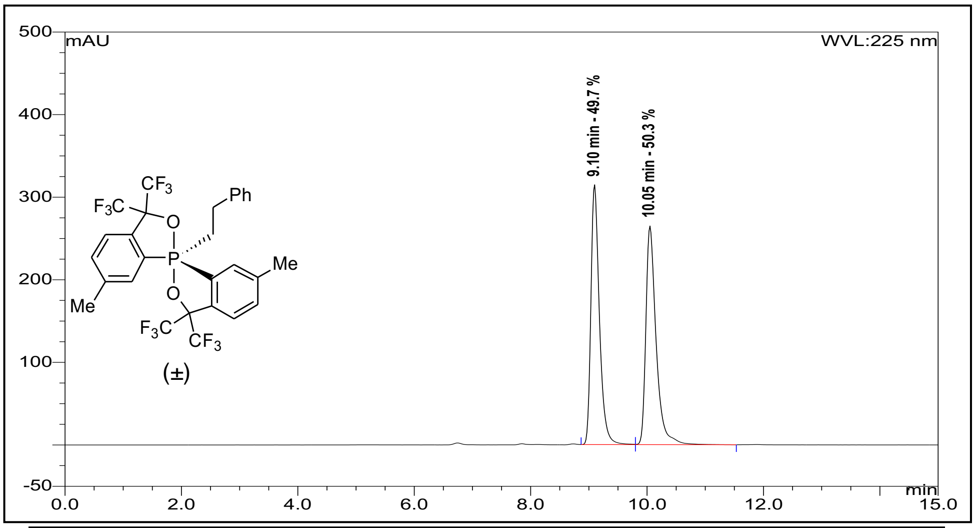

No.	Ret.Time min	Peak Name	Height mAU	Area mAU*min	Rel.Area %
1	9.10	n.a.	314.691	53.169	49.66
2	10.05	n.a.	264.753	53.903	50.34
**Total:**			579.443	107.073	100.00

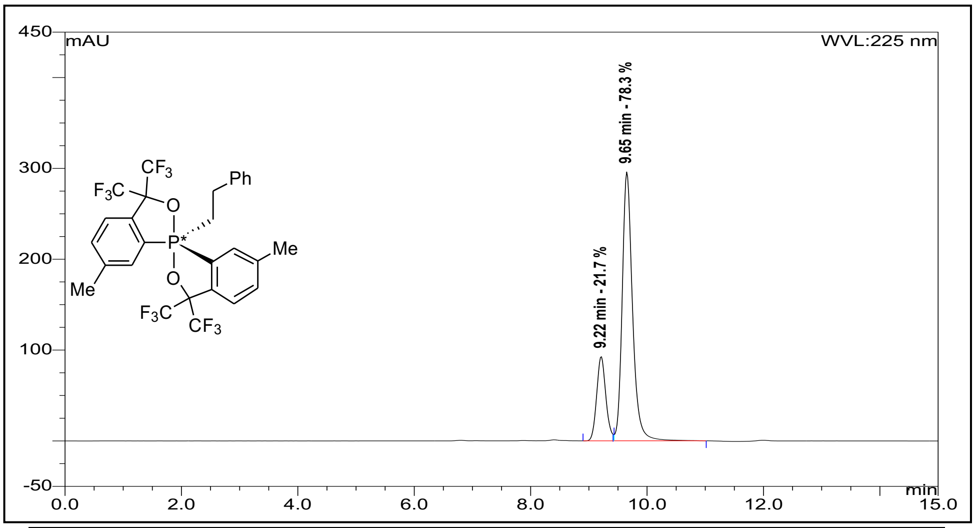

No.	Ret.Time min	Peak Name	Height mAU	Area mAU*min	Rel.Area %
1	9.22	n.a.	92.604	16.867	21.71
2	9.65	n.a.	295.982	60.818	78.29
**Total:**			388.586	77.686	100.00

Supplementary Figure 20. HPLC trace of compound (*S-trans*)-**2l**

[*TBPY*-5-11'-*A*]-1,6,6'-Trimethyl-3,3,3',3'-tetrakis(trifluoromethyl)-3*H*,3'*H*-1λ^5,1'-spirobi[benzo[*c*][2,1]oxaphosphole] ((*S*-*trans*)-2m)

HPLC conditions: Chiralpak IB-N3 (heptane, 100%), Flow: 0.50 mLmin^{-1}, Temp: 5 °C

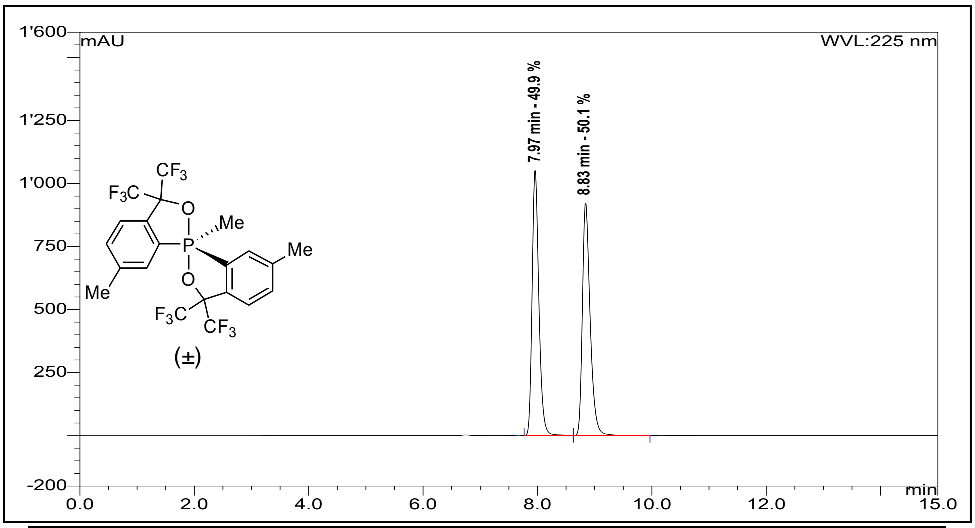

No.	Ret.Time<br>min	Peak Name	Height<br>mAU	Area<br>mAU*min	Rel.Area<br>%
1	7.97	n.a.	1050.382	143.799	49.87
2	8.83	n.a.	919.958	144.528	50.13
**Total:**			1970.340	288.328	100.00

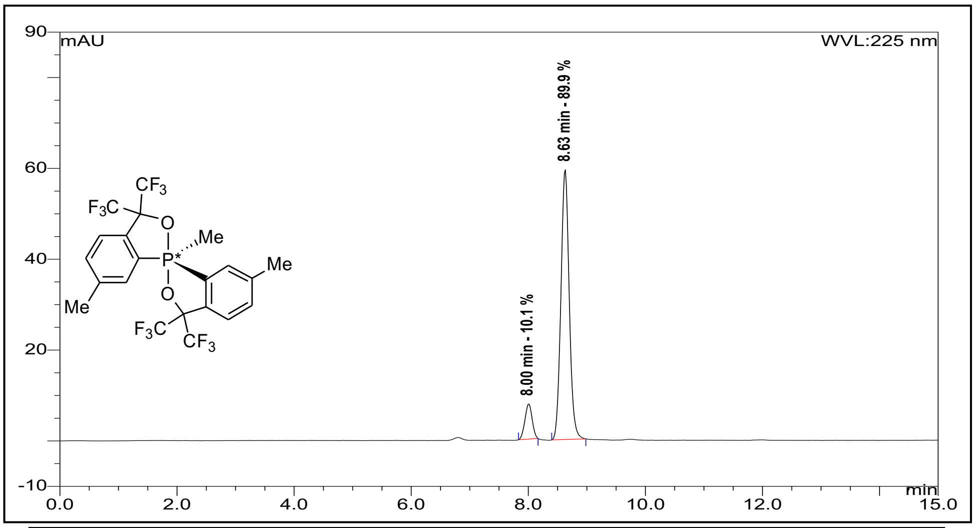

No.	Ret.Time<br>min	Peak Name	Height<br>mAU	Area<br>mAU*min	Rel.Area<br>%
1	8.00	n.a.	7.729	1.102	10.15
2	8.63	n.a.	59.395	9.751	89.85
**Total:**			67.124	10.853	100.00

Supplementary Figure 21. HPLC trace of compound (*S*-*trans*)-**2m**

[*TBPY*-5-11'-*A*]-6,6'-Dimethyl-3,3,3',3'-tetrakis(trifluoromethyl)-1-(6-(trimethylsi-lyl)hex-5-yn-1-yl)-3*H*,3'*H*-1λ^5,1'-spirobi[benzo[*c*][2,1]oxaphosphole] ((*S*-*trans*)-2n)

HPLC conditions: Chiralpak IB-N3 (heptane, 100%), Flow: 0.50 mLmin^{-1}, Temp: 5 °C

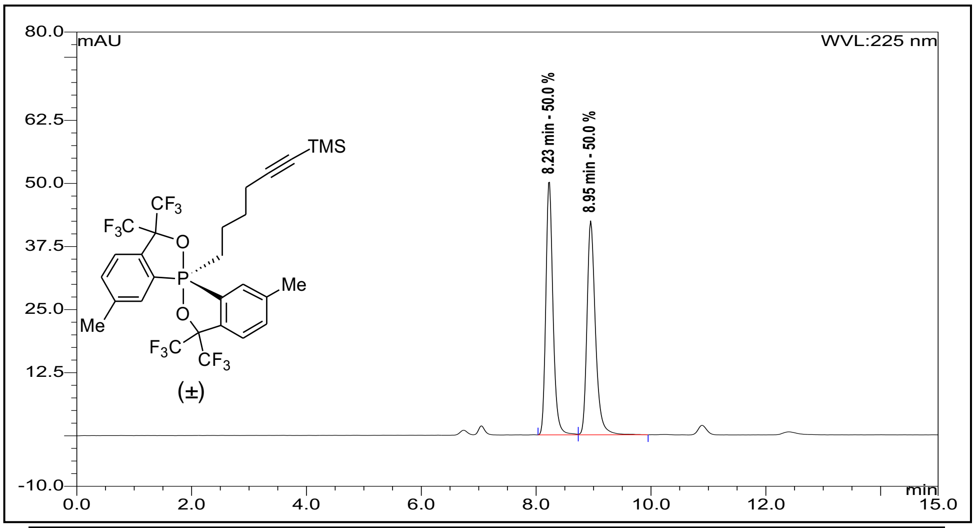

No.	Ret.Time min	Peak Name	Height mAU	Area mAU*min	Rel.Area %
1	8.23	n.a.	50.070	7.228	50.03
2	8.95	n.a.	42.410	7.219	49.97
**Total:**			92.481	14.447	100.00

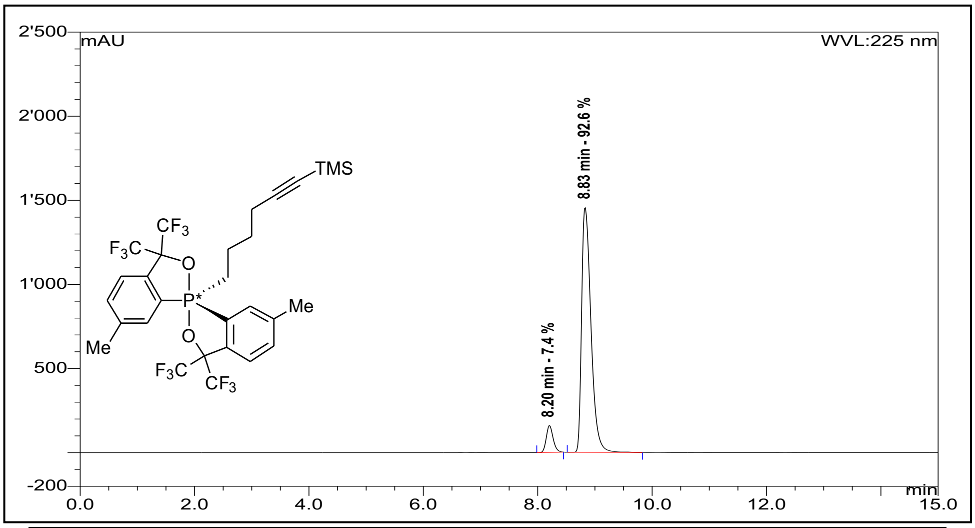

No.	Ret.Time min	Peak Name	Height mAU	Area mAU*min	Rel.Area %
1	8.20	n.a.	160.193	22.013	7.40
2	8.83	n.a.	1454.323	275.372	92.60
**Total:**			1614.516	297.385	100.00

Supplementary Figure 22. HPLC trace of compound (*S*-*trans*)-**2n**

[*TBPY*-5-11'-*A*]-1-(4-Methoxybutyl)-6,6'-dimethyl-3,3,3',3'-tetrakis(trifluoromethyl)-3*H*,3'*H*-1λ^5,1'-spirobi[benzo[*c*][2,1]oxaphosphole] ((*S*-*trans*)-2o)

HPLC conditions: Chiralpak IB-N3 (heptane, 100%), Flow: 0.50 mLmin^{-1}, Temp: 5 °C

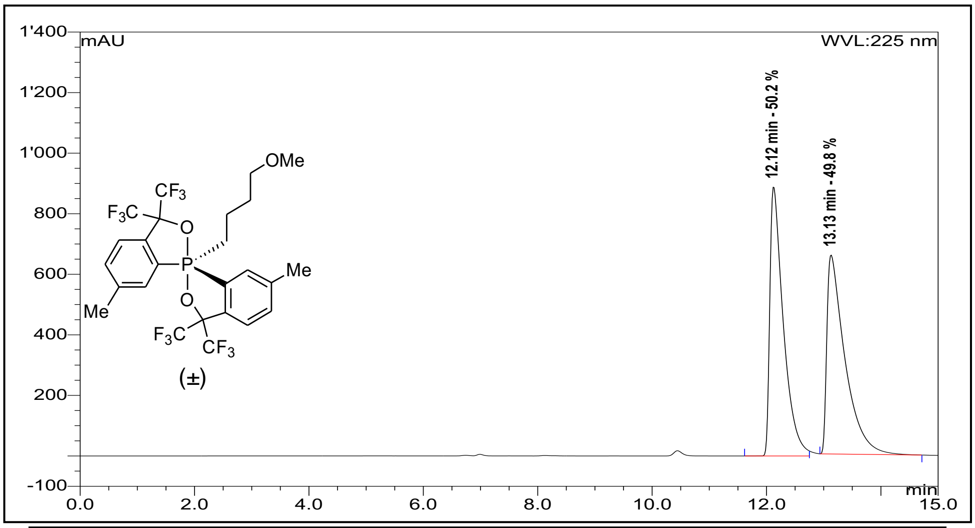

No.	Ret.Time min	Peak Name	Height mAU	Area mAU*min	Rel.Area %
1	12.12	n.a.	888.000	242.528	50.18
2	13.13	n.a.	656.952	240.749	49.82
**Total:**			1544.952	483.278	100.00

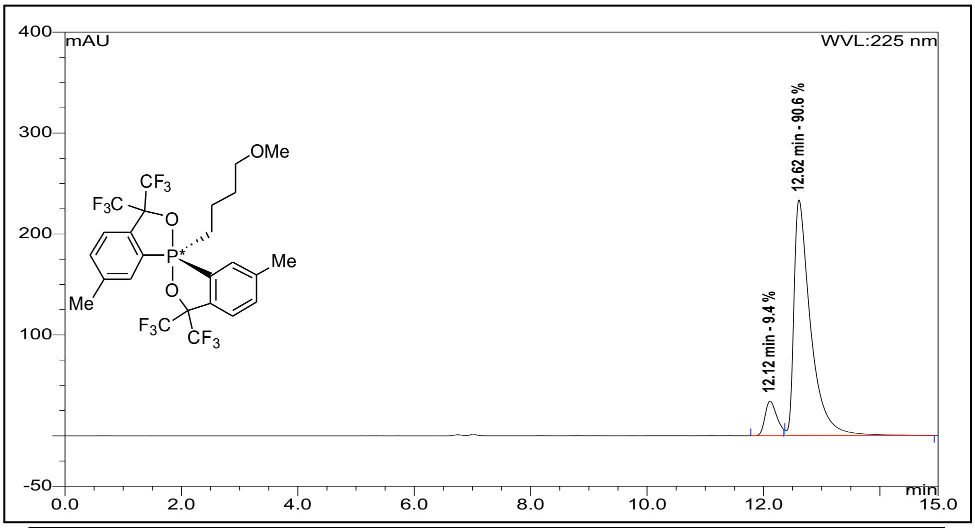

No.	Ret.Time min	Peak Name	Height mAU	Area mAU*min	Rel.Area %
1	12.12	n.a.	34.418	8.067	9.43
2	12.62	n.a.	233.592	77.458	90.57
**Total:**			268.011	85.525	100.00

Supplementary Figure 23. HPLC trace of compound (*S*-*trans*)-**2o**

[*TBPY*-5-11'-*A*]-1-(4-(1,3-Dioxolan-2-yl)butyl)-6,6'-dimethyl-3,3,3',3'-tetrakis(trifluoromethyl)-3*H*,3'*H*-1λ^5,1'-spirobi[benzo[*c*][2,1]oxaphosphole] ((*S*-*trans*)-2p)

HPLC conditions: two coupled Chiralpak IB-N3 columns (heptane, 100%), Flow: 1.0 mLmin^{-1}, Temp: 40 °C

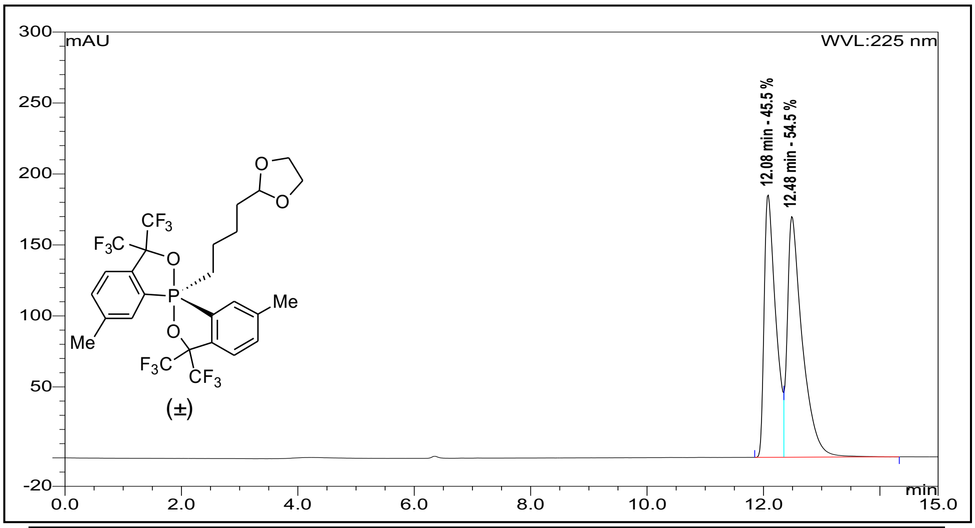

No.	Ret.Time<br>min	Peak Name	Height<br>mAU	Area<br>mAU*min	Rel.Area<br>%
1	12.08	n.a.	184.916	42.588	45.55
2	12.48	n.a.	169.643	50.910	54.45
**Total:**			354.560	93.498	100.00

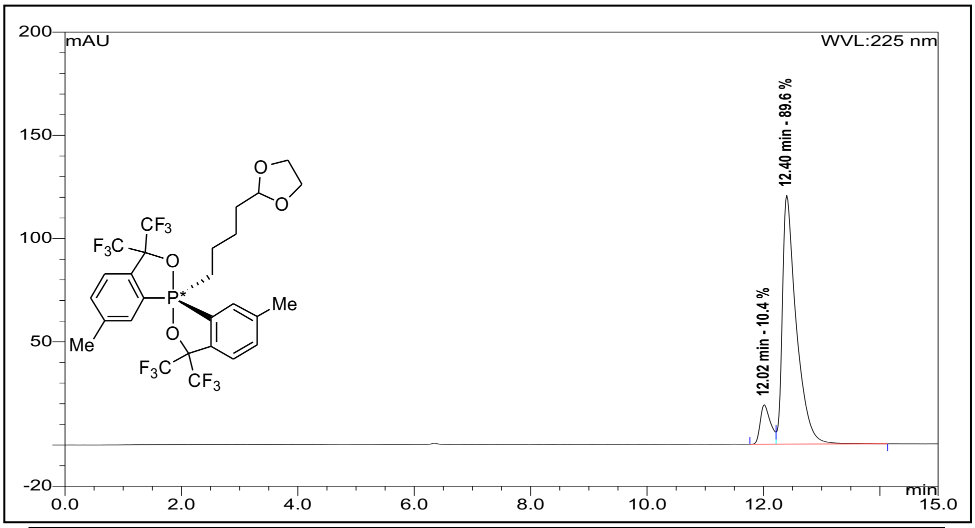

No.	Ret.Time<br>min	Peak Name	Height<br>mAU	Area<br>mAU*min	Rel.Area<br>%
1	12.02	n.a.	19.108	3.883	10.41
2	12.40	n.a.	120.495	33.424	89.59
**Total:**			139.603	37.307	100.00

Supplementary Figure 24. HPLC trace of compound (*S-trans*)-**2p**

[*TBPY*-5-11'-*C*]-1-Butyl-6,6'-dimethyl-3,3,3',3'-tetrakis(trifluoromethyl)-3*H*,3'*H*-1λ⁵,1'-spirobi[benzo[*c*][2,1]oxaphosphole] ((*R*-*trans*)-2a)

HPLC conditions: Chiralpak IB-N3 (heptane, 100%), Flow: 0.50 mLmin^{-1}, Temp: 5 °C

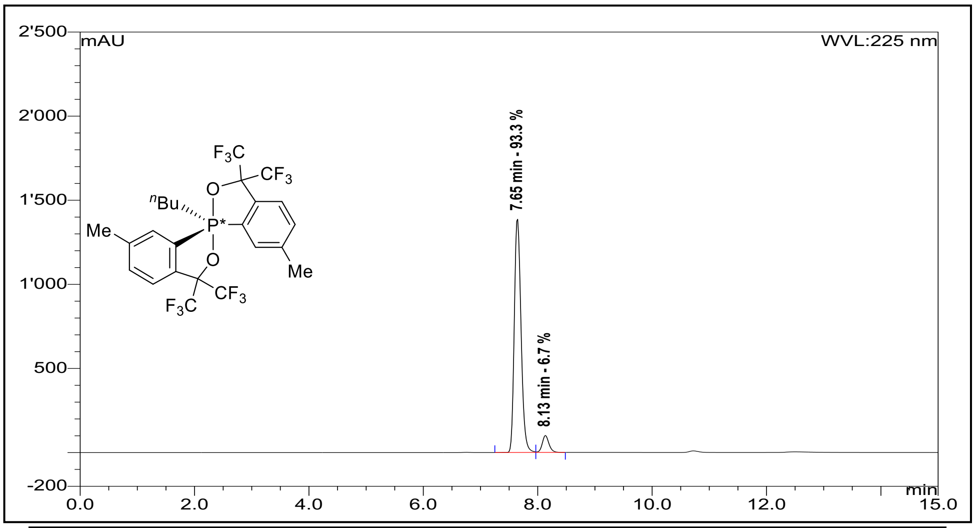

No.	Ret.Time min	Peak Name	Height mAU	Area mAU*min	Rel.Area %
1	7.65	n.a.	1386.710	189.454	93.29
2	8.13	n.a.	101.189	13.625	6.71
**Total:**			1487.899	203.078	100.00

Supplementary Figure 25. HPLC trace of compound (*R*-*trans*)-**2a**

NMR Spectra

^{1}H, $^{13}C\{^{1}H\}$ and $^{19}F\{^{1}H\}$ spectra of 2,3,4,5,6-Pentafluorophenyl 2-bromo-4-methylbenzoate (S1a)

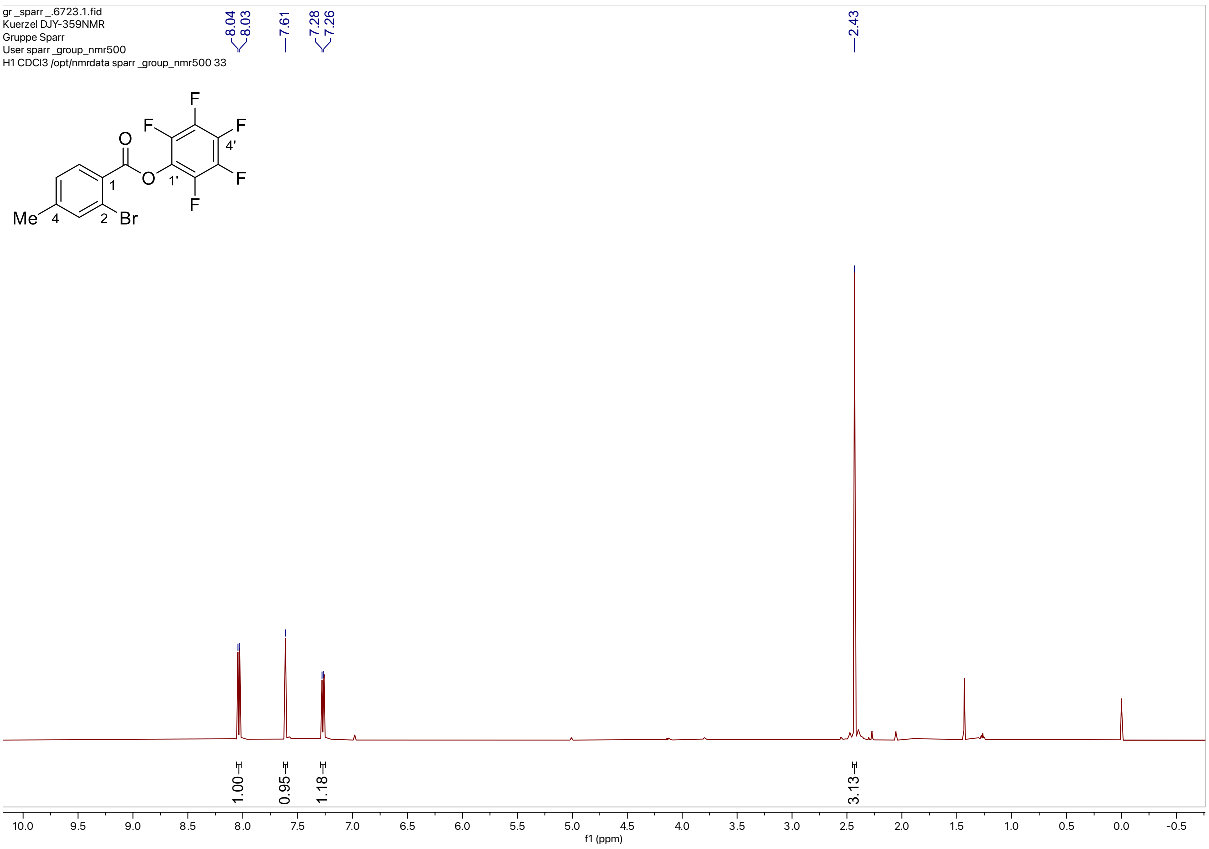

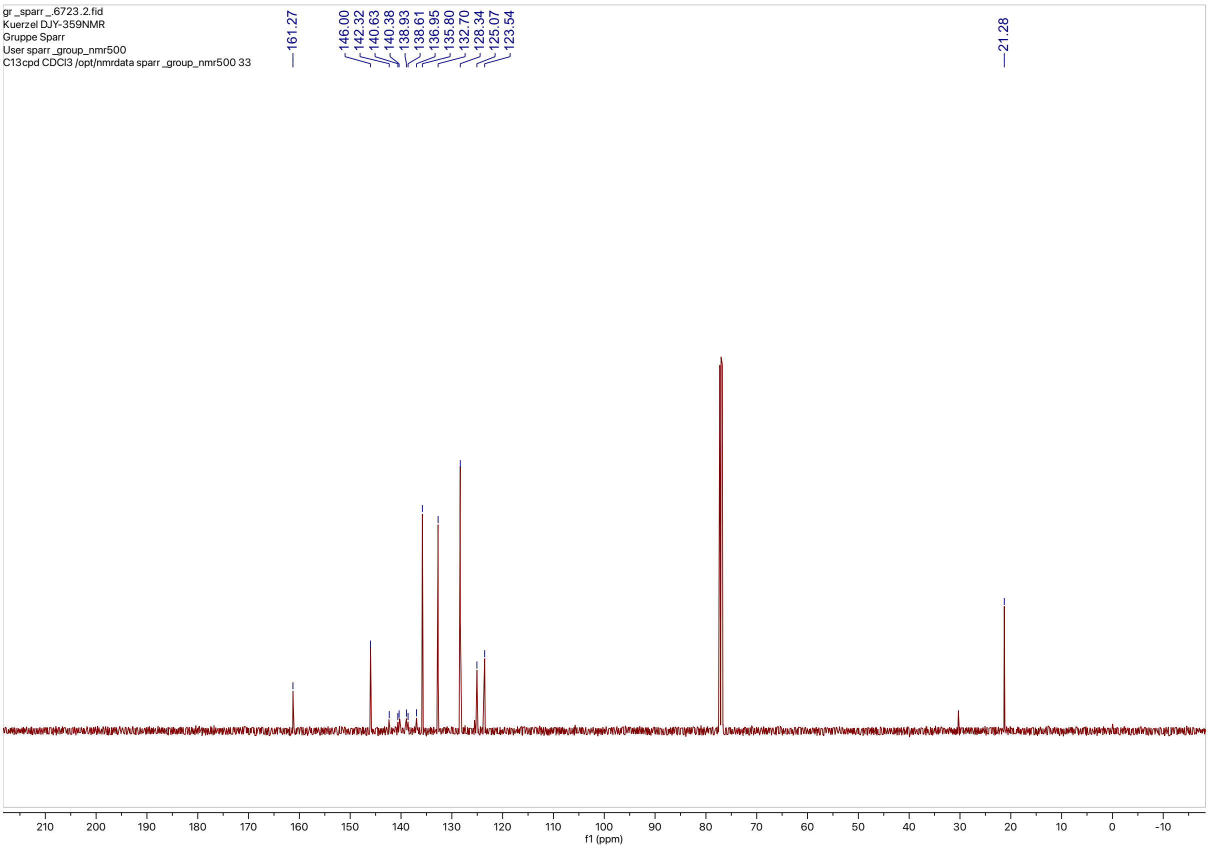

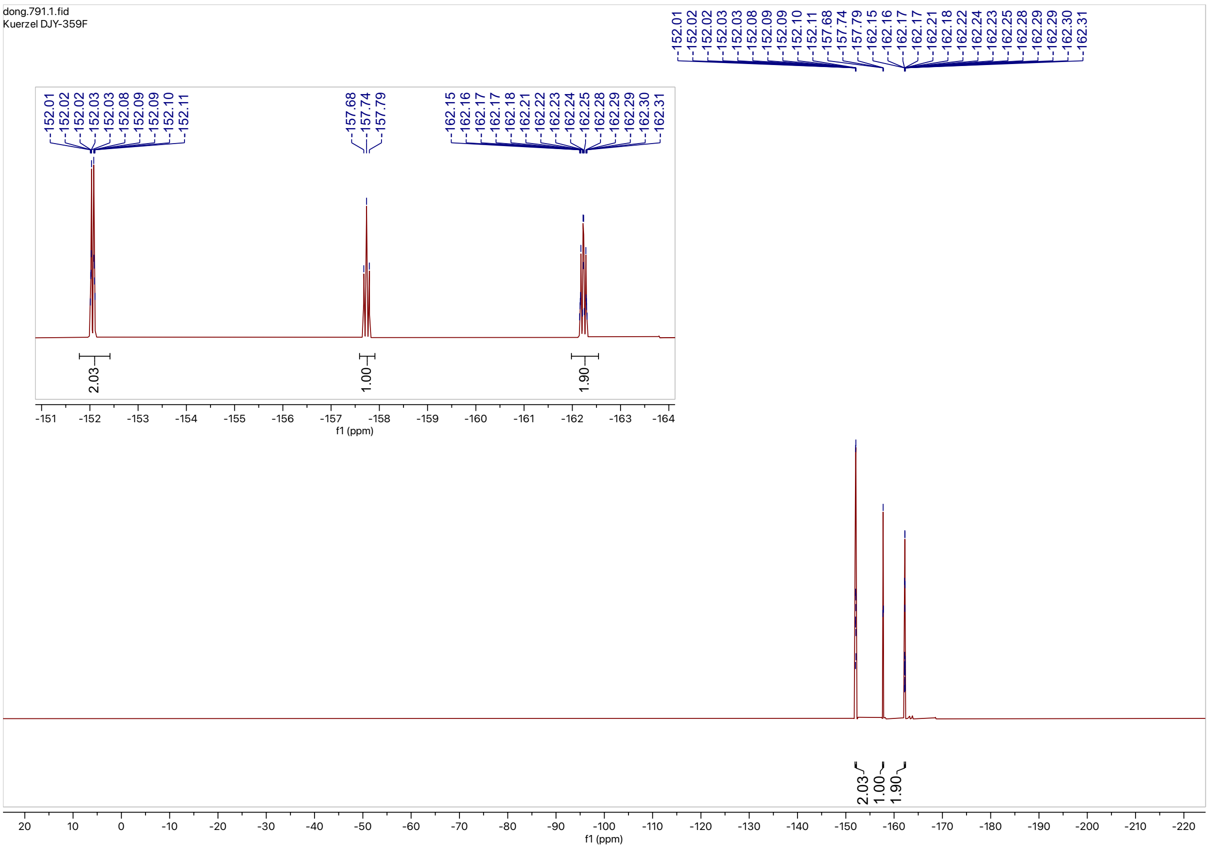

Supplementary Figure 26. ^{1}H (500 MHz, $CDCl_3$, 25 °C), $^{13}C\{^{1}H\}$ (126 MHz, $CDCl_3$, 25 °C) and $^{19}F\{^{1}H\}$ ((376 MHz, $CDCl_3$, 25 °C) spectra of **S1a**

^{1}H, $^{13}C\{^{1}H\}$ and $^{19}F\{^{1}H\}$ spectra of ((2-(2-Bromo-4-methylphenyl)-1,1,1,3,3,3-hexafluoropropan-2-yl)oxy)trimethylsilane (S2a)

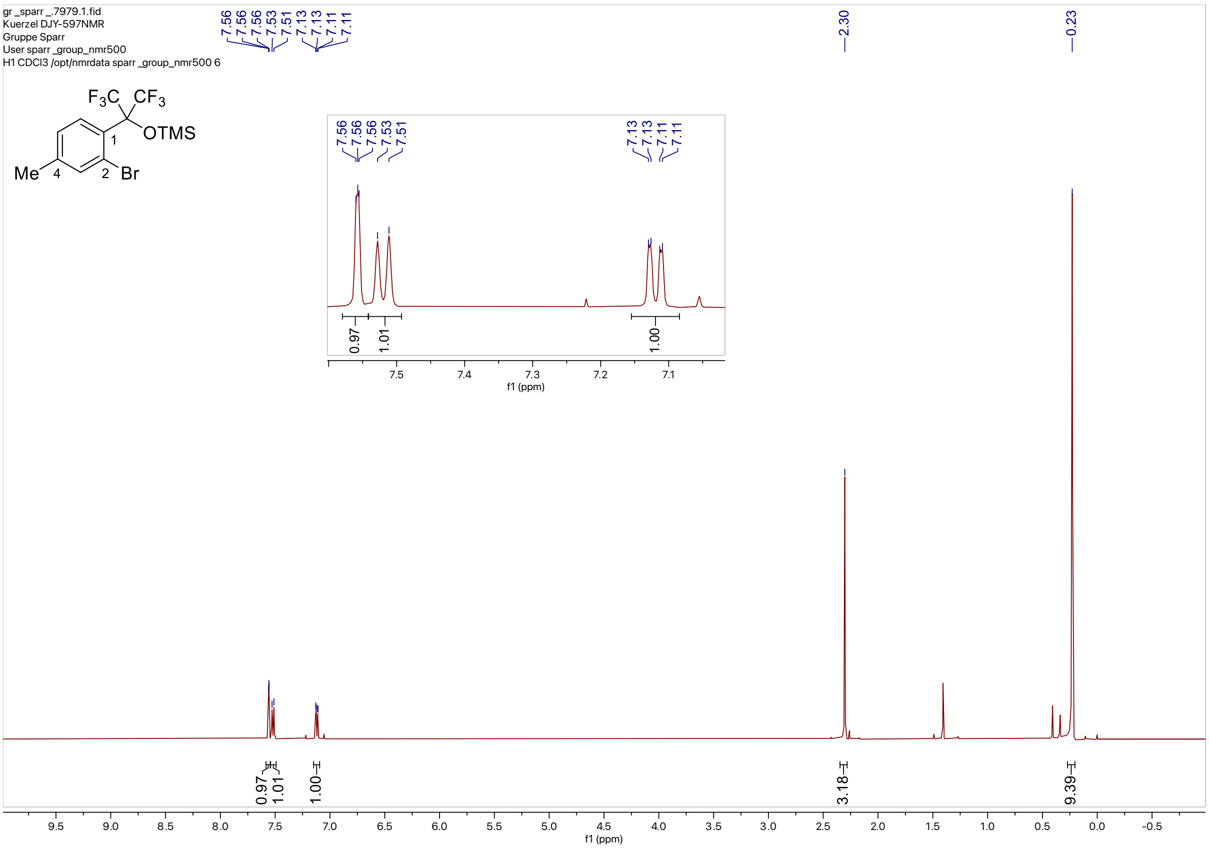

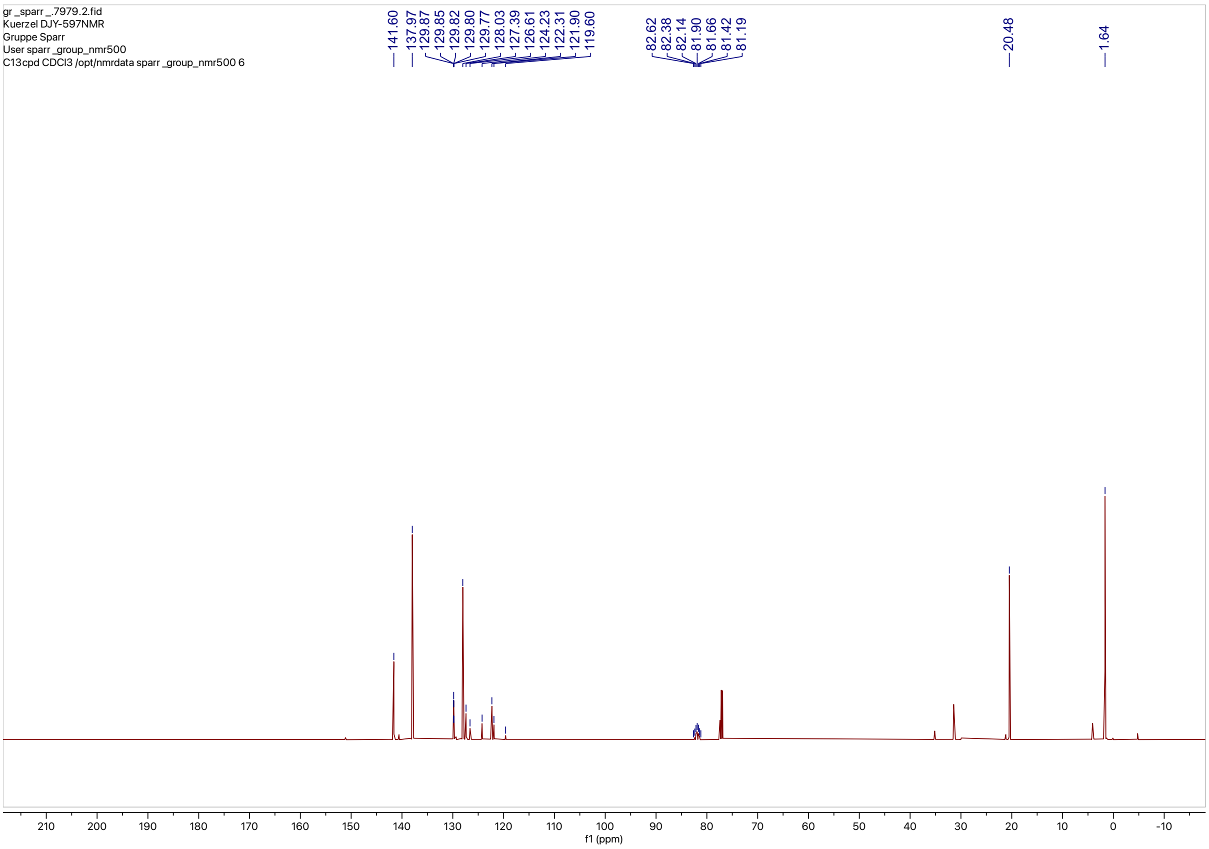

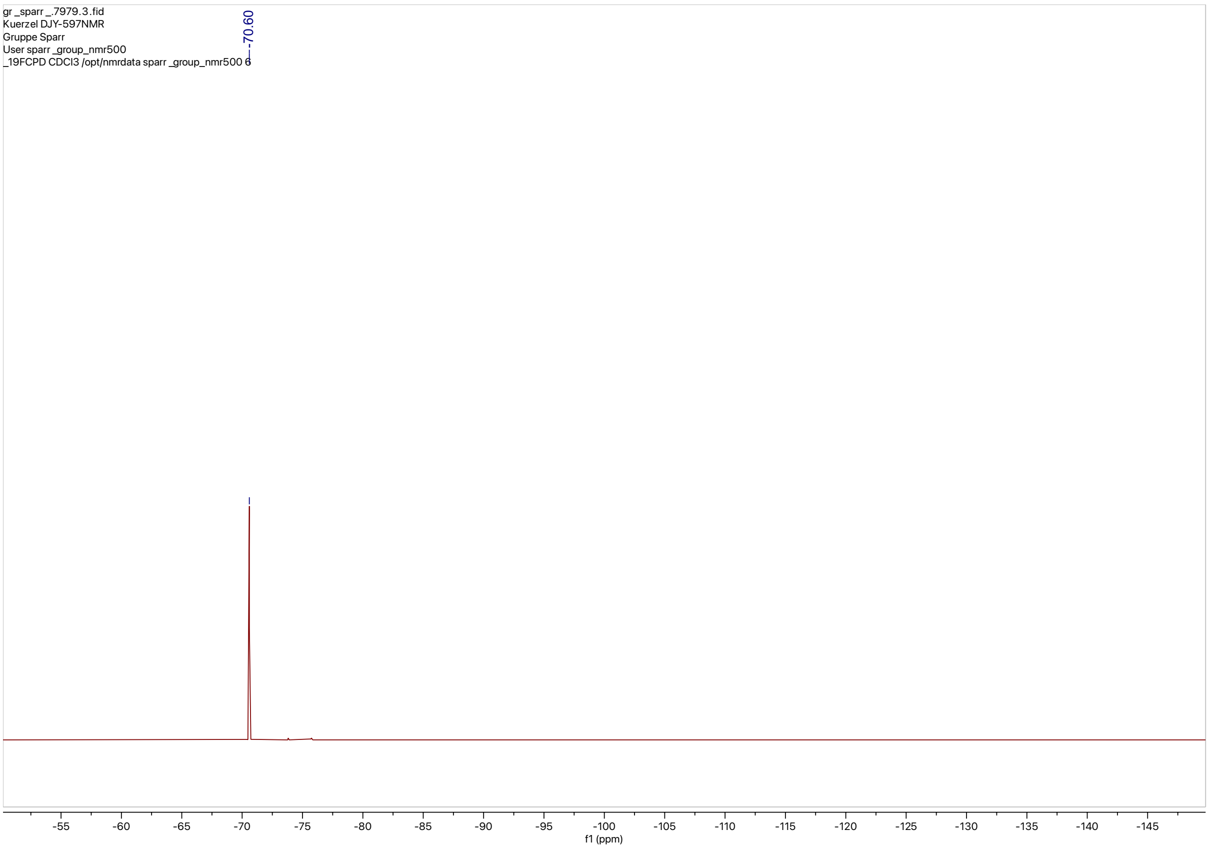

Supplementary Figure 27. ^{1}H (500 MHz, $CDCl_3$, 25 °C), $^{13}C\{^{1}H\}$ (126 MHz, $CDCl_3$, 25 °C) and $^{19}F\{^{1}H\}$ ((470 MHz, $CDCl_3$, 25 °C) spectra of **S2a**

[1]H, [13]C{[1]H} and [19]F{[1]H} spectra of 2-(2-Bromo-4-methylphenyl)-1,1,1,3,3,3-hexafluoropropan-2-ol (S3a)

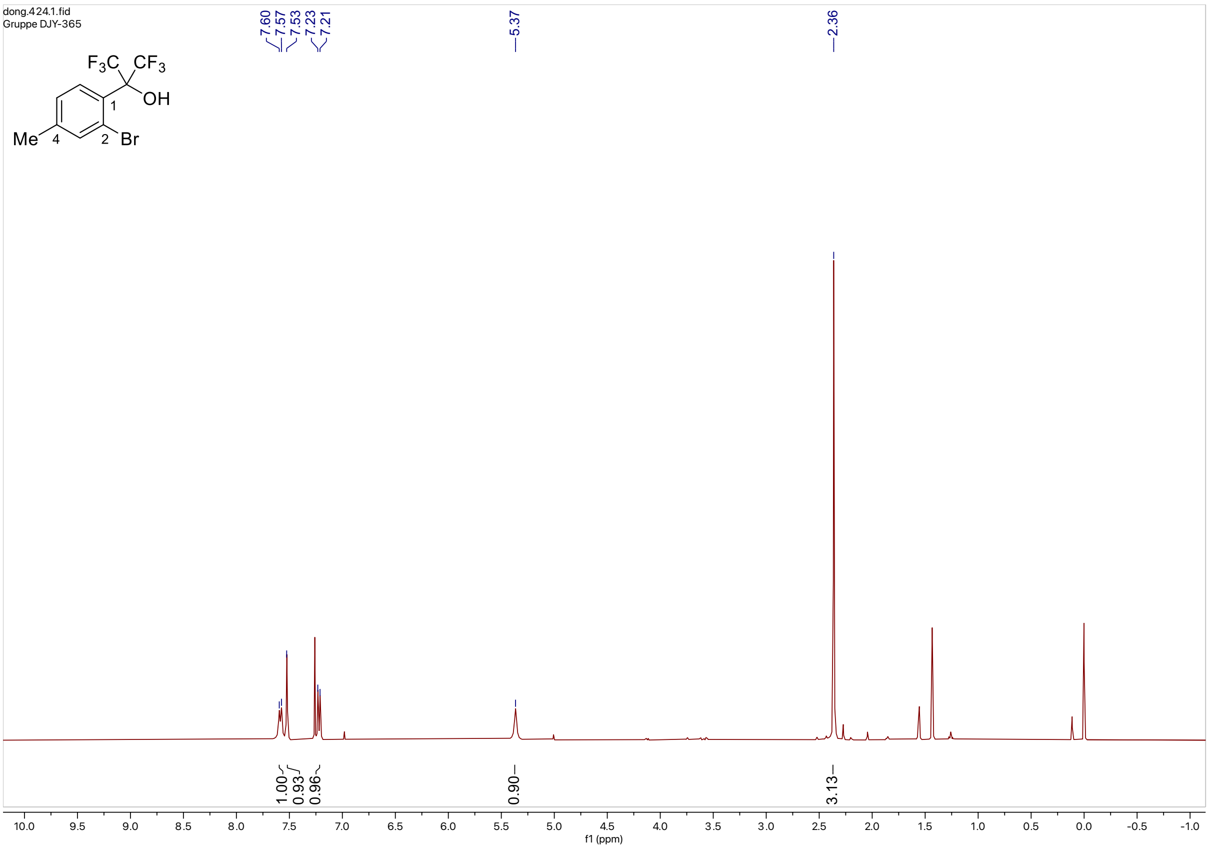

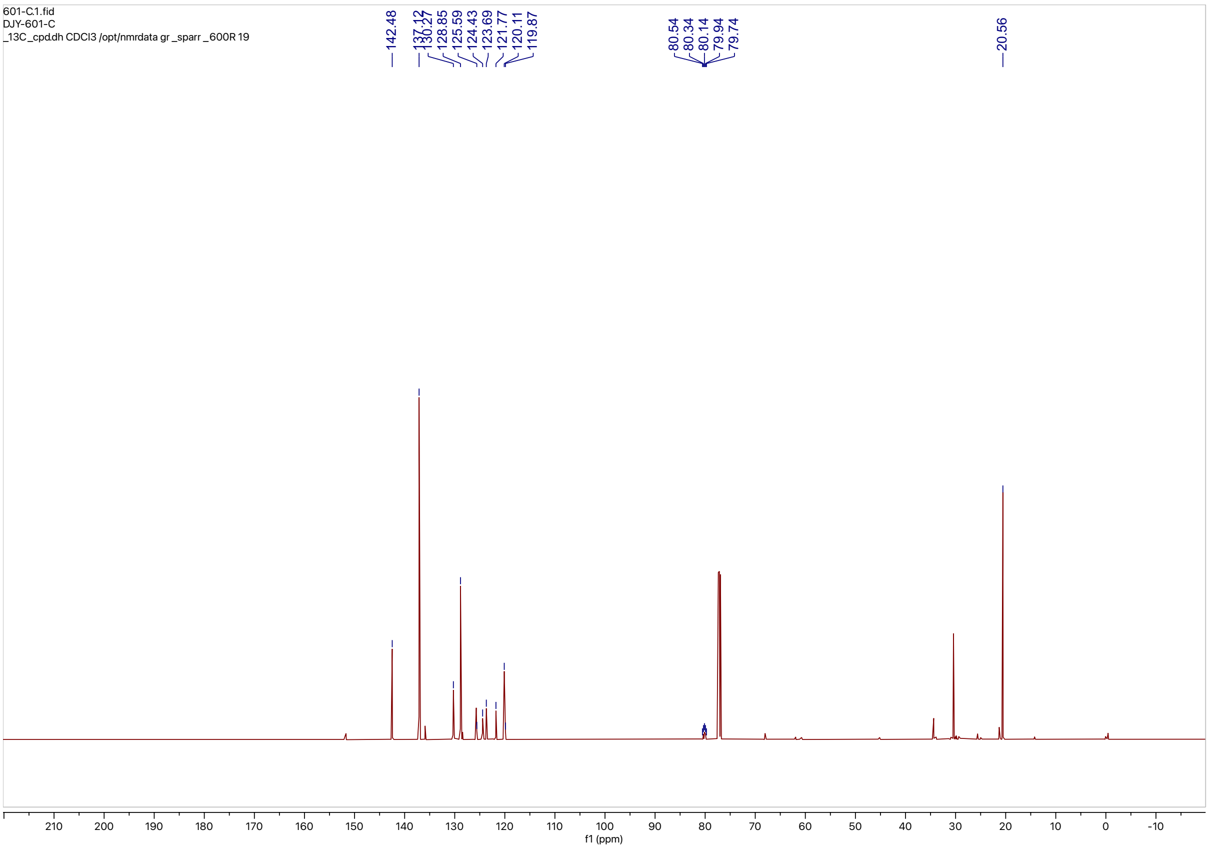

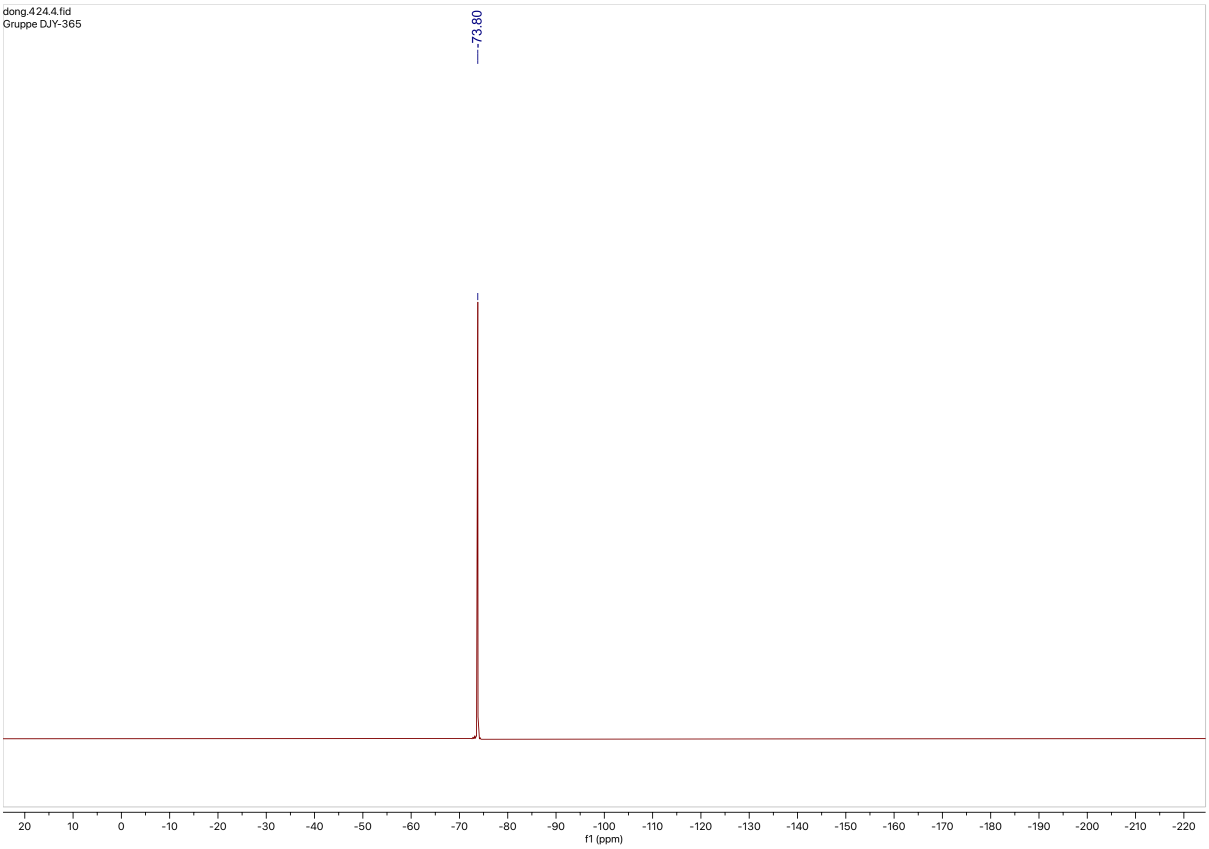

Supplementary Figure 28. ^{1}H (400 MHz, $CDCl_3$, 25 °C), $^{13}C\{^{1}H\}$ (151 MHz, $CDCl_3$, 25 °C) and $^{19}F\{^{1}H\}$ ((376 MHz, $CDCl_3$, 25 °C) spectra of **S3a**

^{1}H, $^{13}C\{^{1}H\}$, $^{19}F\{^{1}H\}$ and $^{31}P\{^{1}H\}$ spectra of [*TBPY*-5-11']-6,6'-Dimethyl-1-hydro-3,3,3',3'-tetrakis(trifluoromethyl)-3*H*,3'*H*-1λ^{5},1'-spirobi[ben-zo[*c*][2,1]oxaphosphole] (S4a)

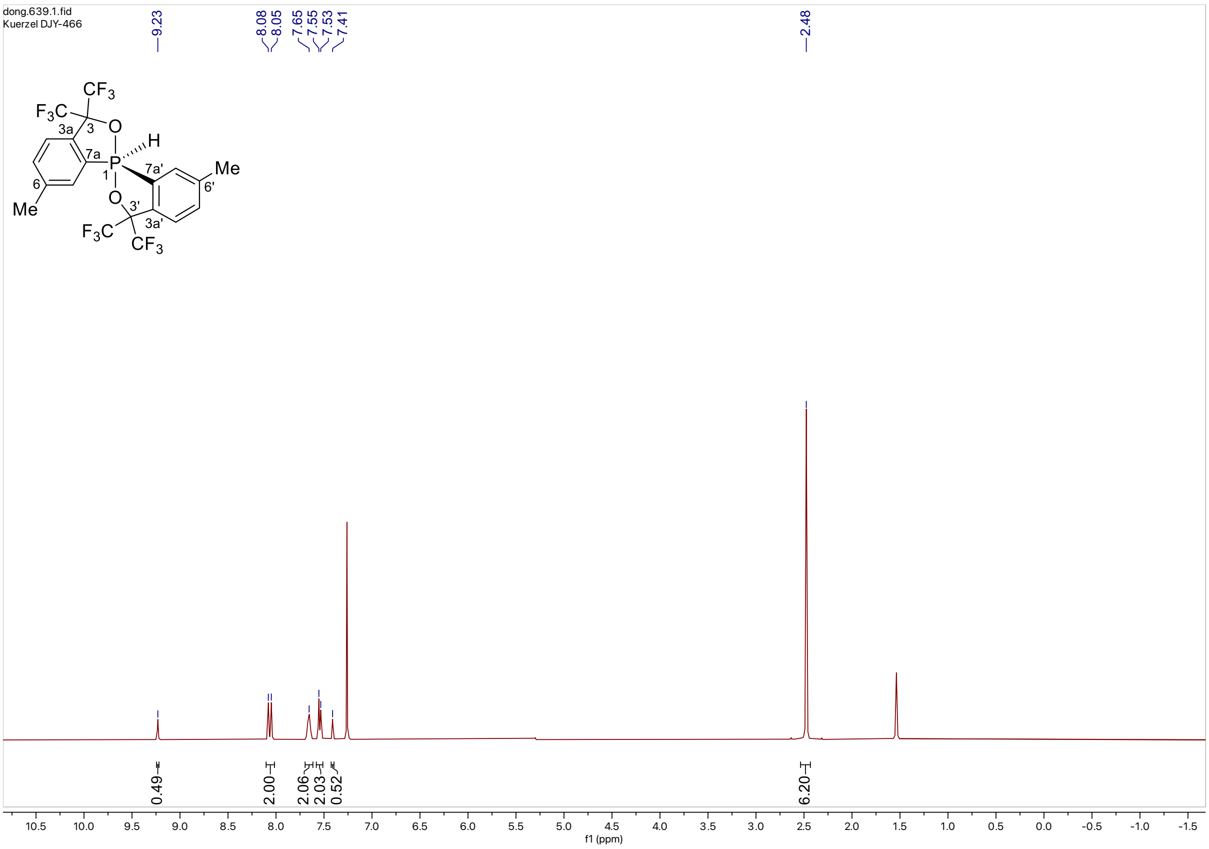

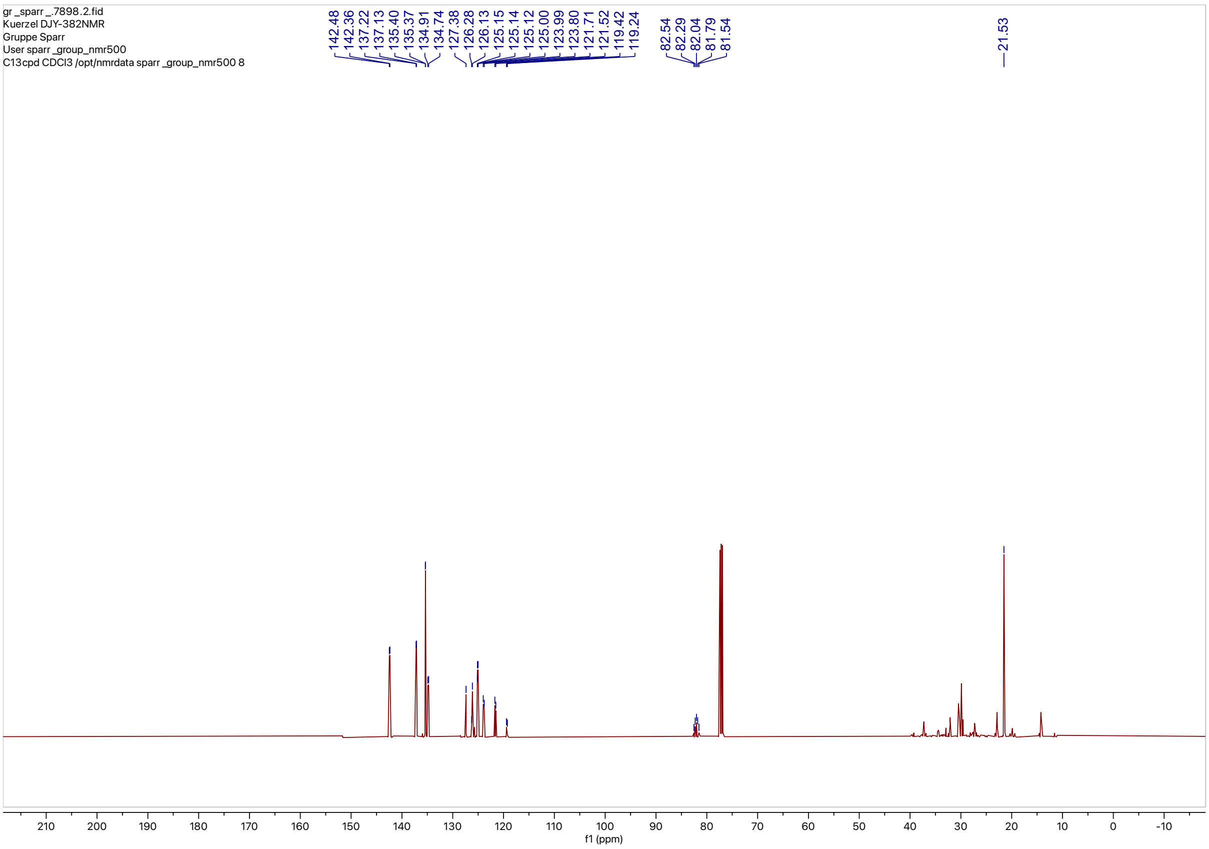

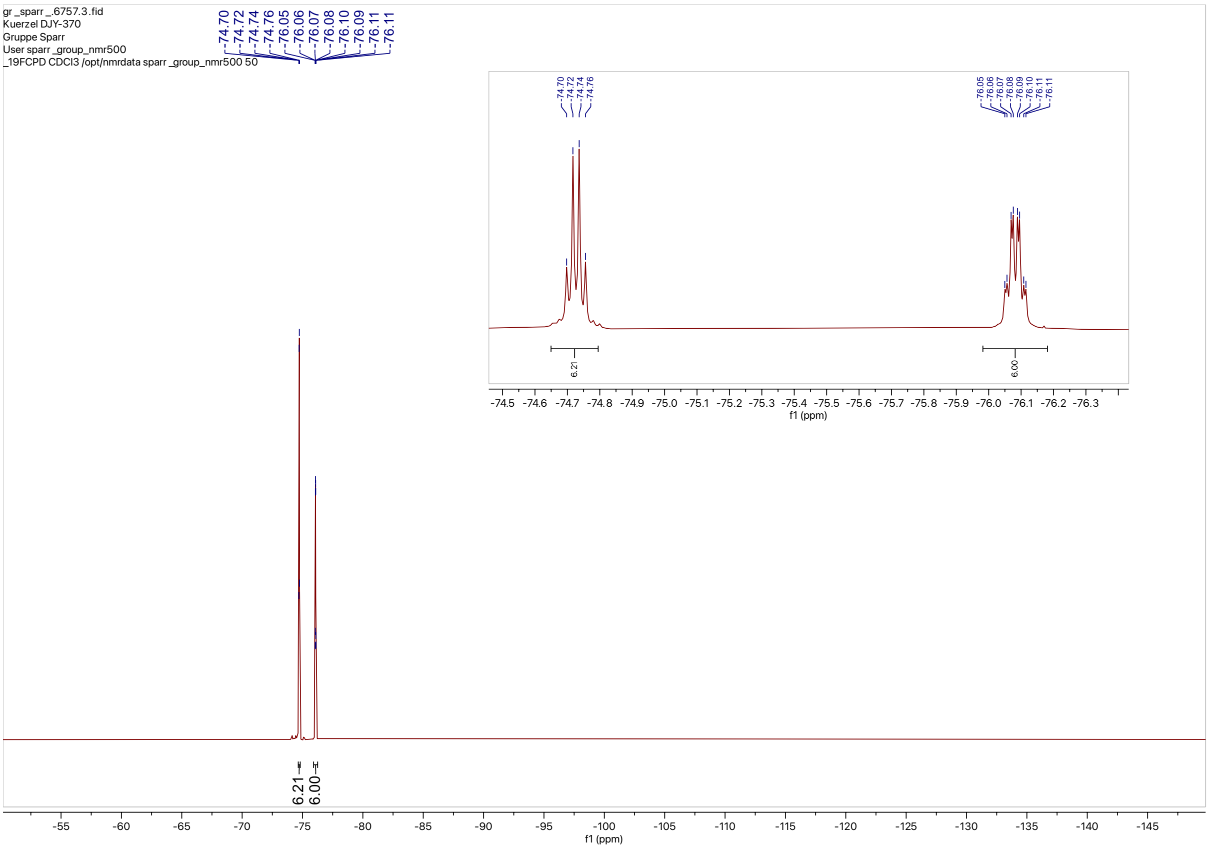

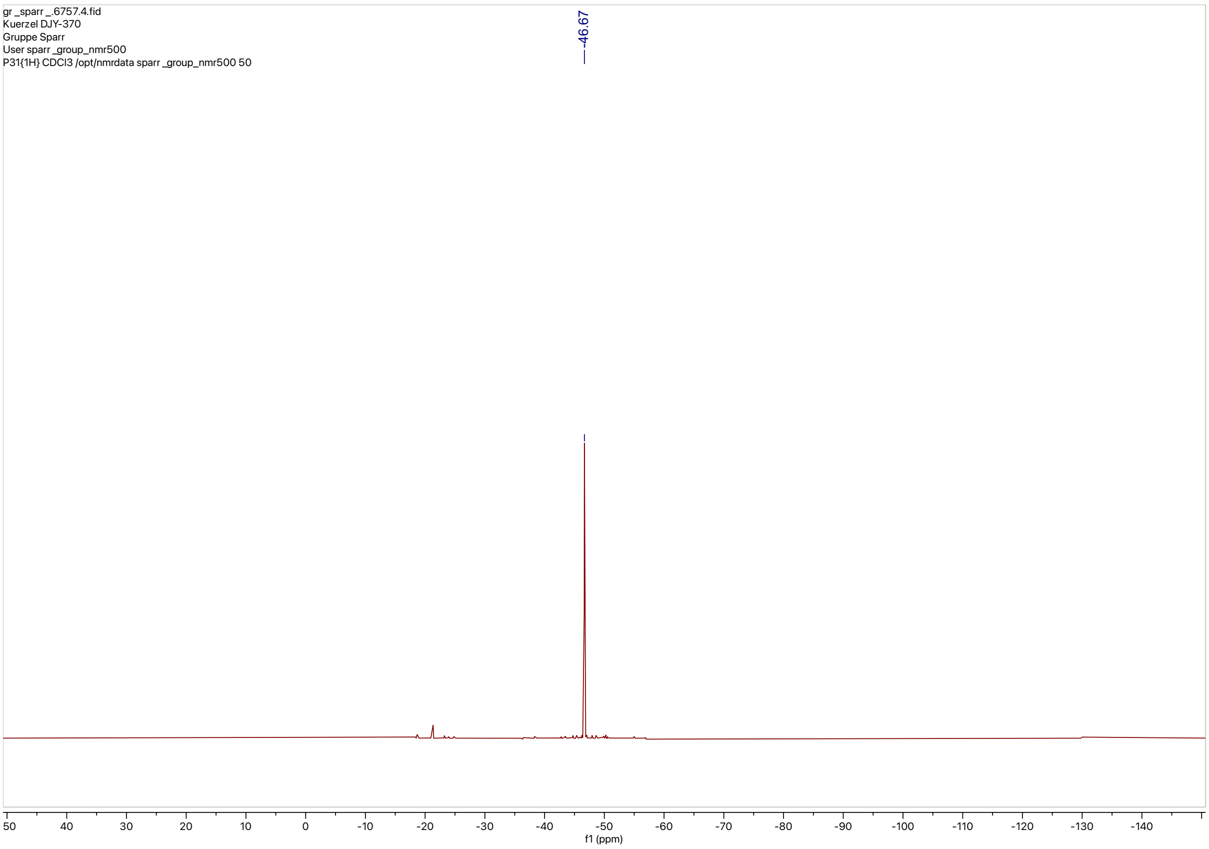

Supplementary Figure 29. ^{1}H (400 MHz, $CDCl_3$, 25 °C), $^{13}C\{^{1}H\}$ (126 MHz, $CDCl_3$, 25 °C), $^{19}F\{^{1}H\}$ (470 MHz, $CDCl_3$, 25 °C) and $^{31}P\{^{1}H\}$ (202 MHz, $CDCl_3$, 25 °C) spectra of **S4a**

^{1}H, $^{19}F\{^{1}H\}$ and $^{31}P\{^{1}H\}$ spectra of [*TBPY*-5-15]-1,1,1,3,3,3-Hexafluoro-2-(2-(1-butyl-1-hydro-6-methyl-3,3-bis(trifluoromethyl)-3*H*-1λ⁵-benzo[*c*][2,1]oxaphosphol-1-yl)-4-methylphenyl)propan-2-ol (1a)

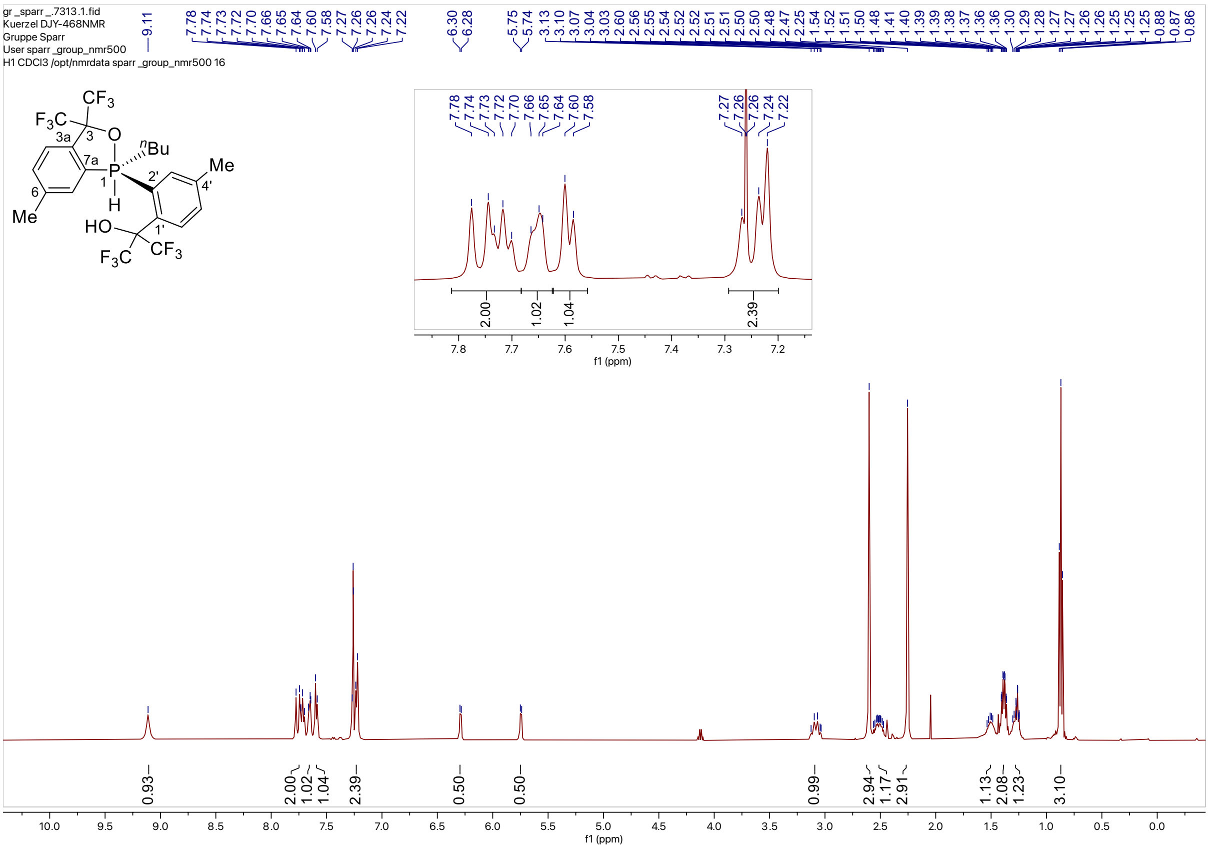

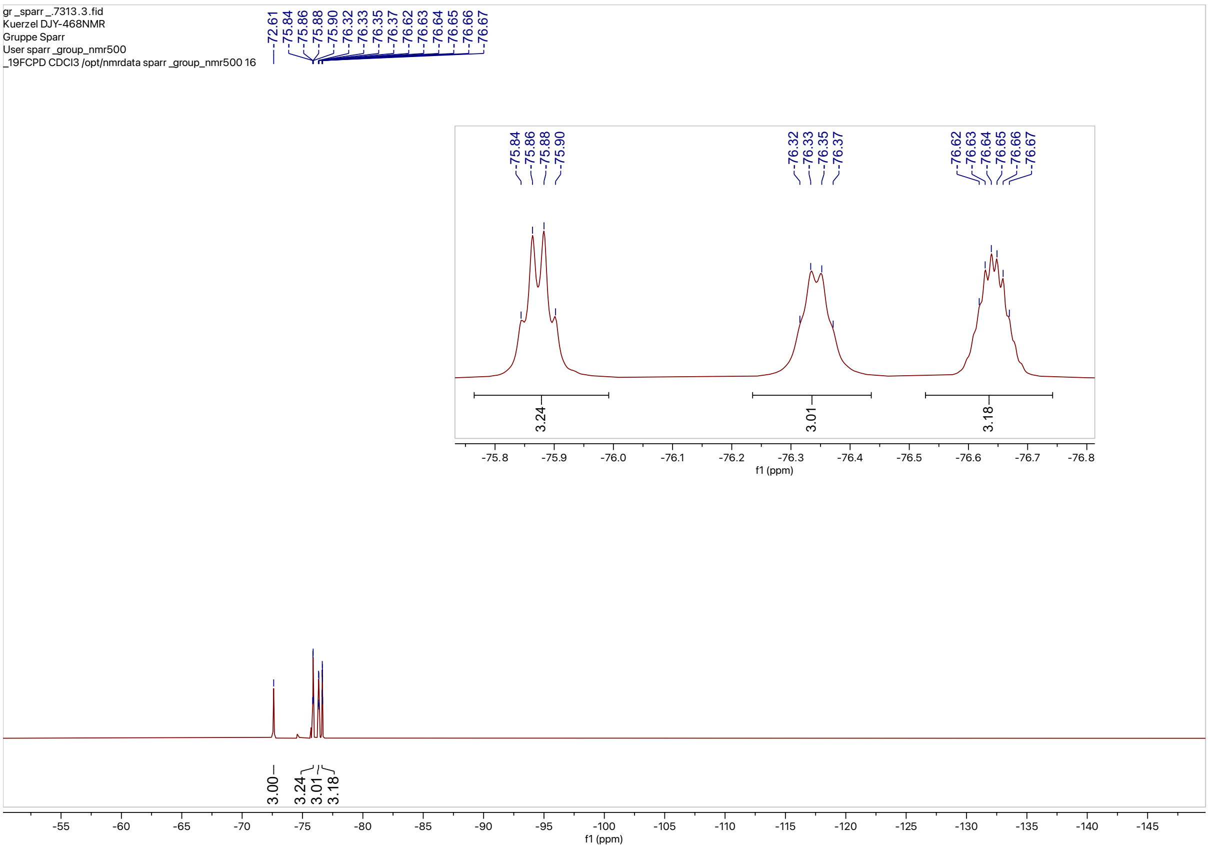

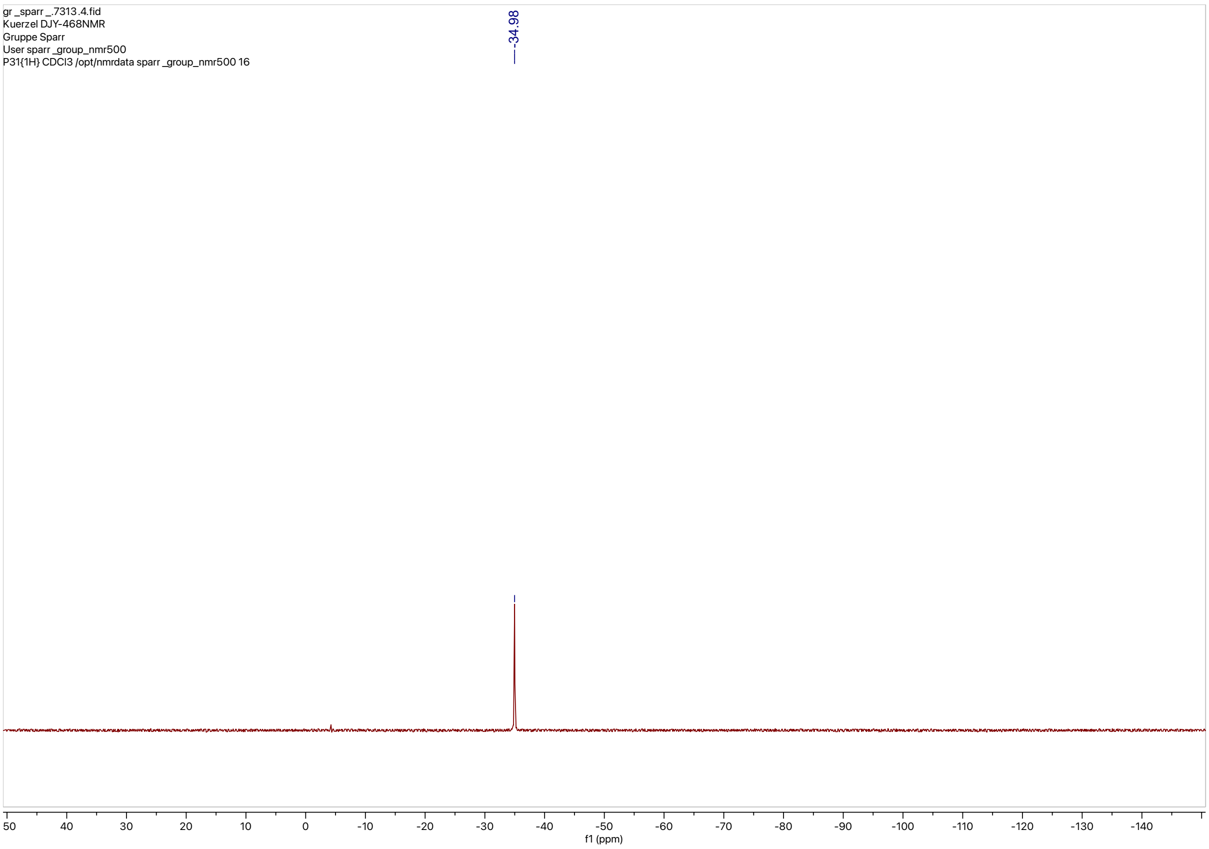

Supplementary Figure 30. ^{1}H (500 MHz, $CDCl_3$, 25 °C), $^{19}F\{^{1}H\}$ (470 MHz, $CDCl_3$, 25 °C) and $^{31}P\{^{1}H\}$ (202 MHz, $CDCl_3$, 25 °C) spectra of **1a**

^{1}H, $^{13}C\{^{1}H\}$ and $^{19}F\{^{1}H\}$ spectra of 2-(2-Bromo-5-methylphenyl)-1,1,1,3,3,3-hexafluoropropan-2-ol (S3c)

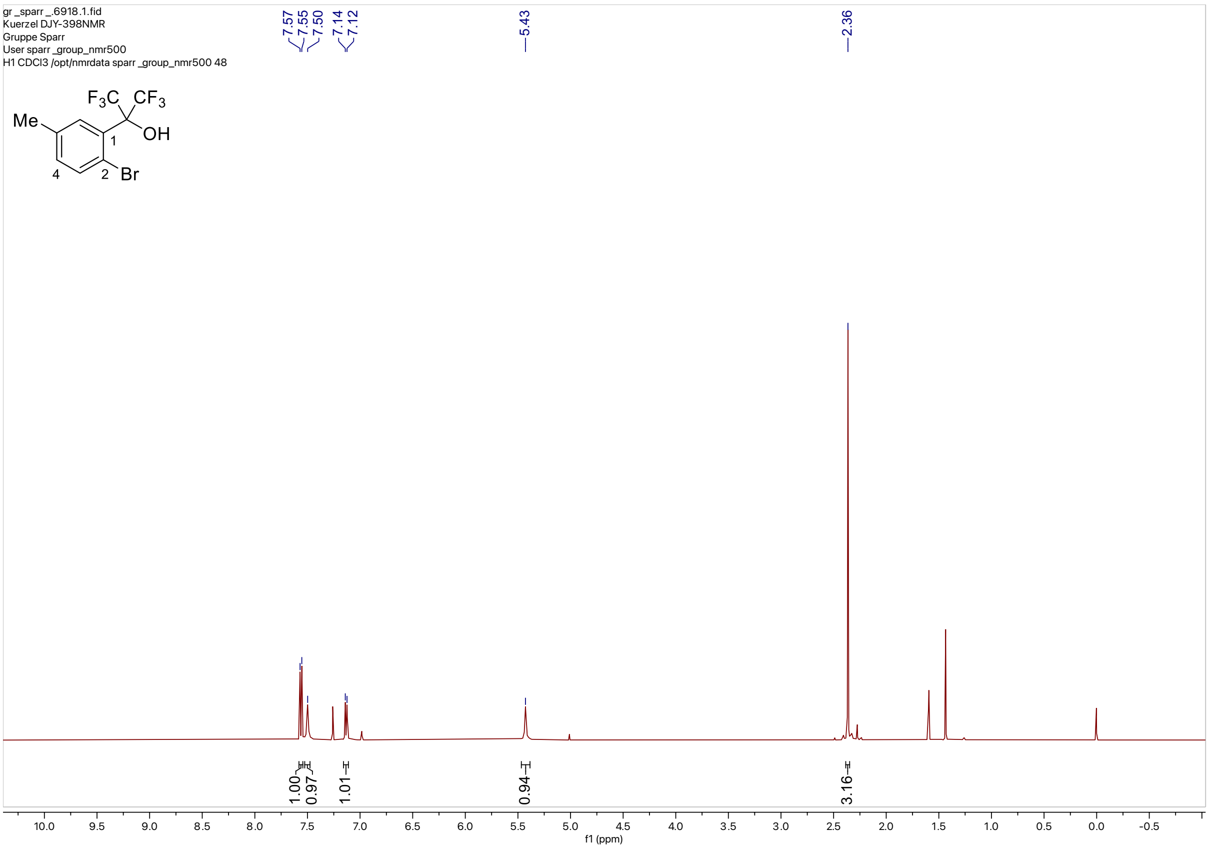

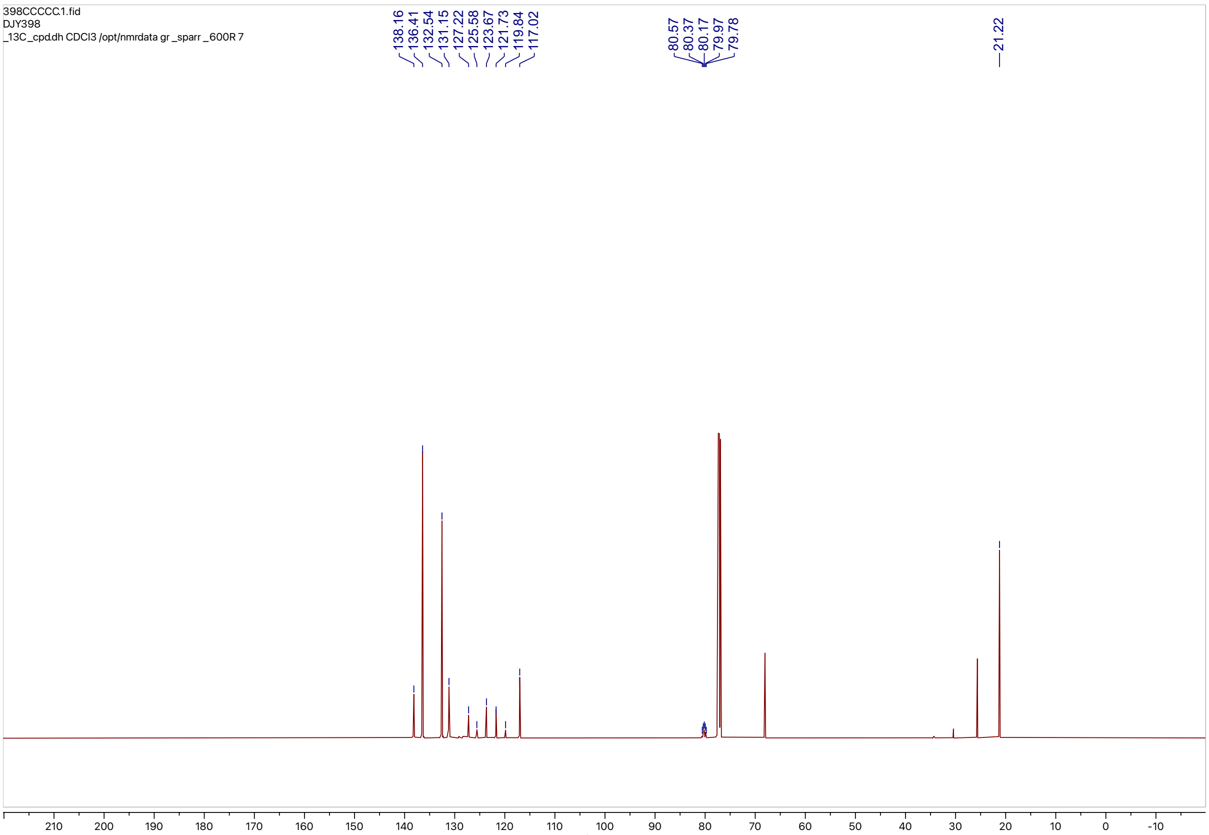

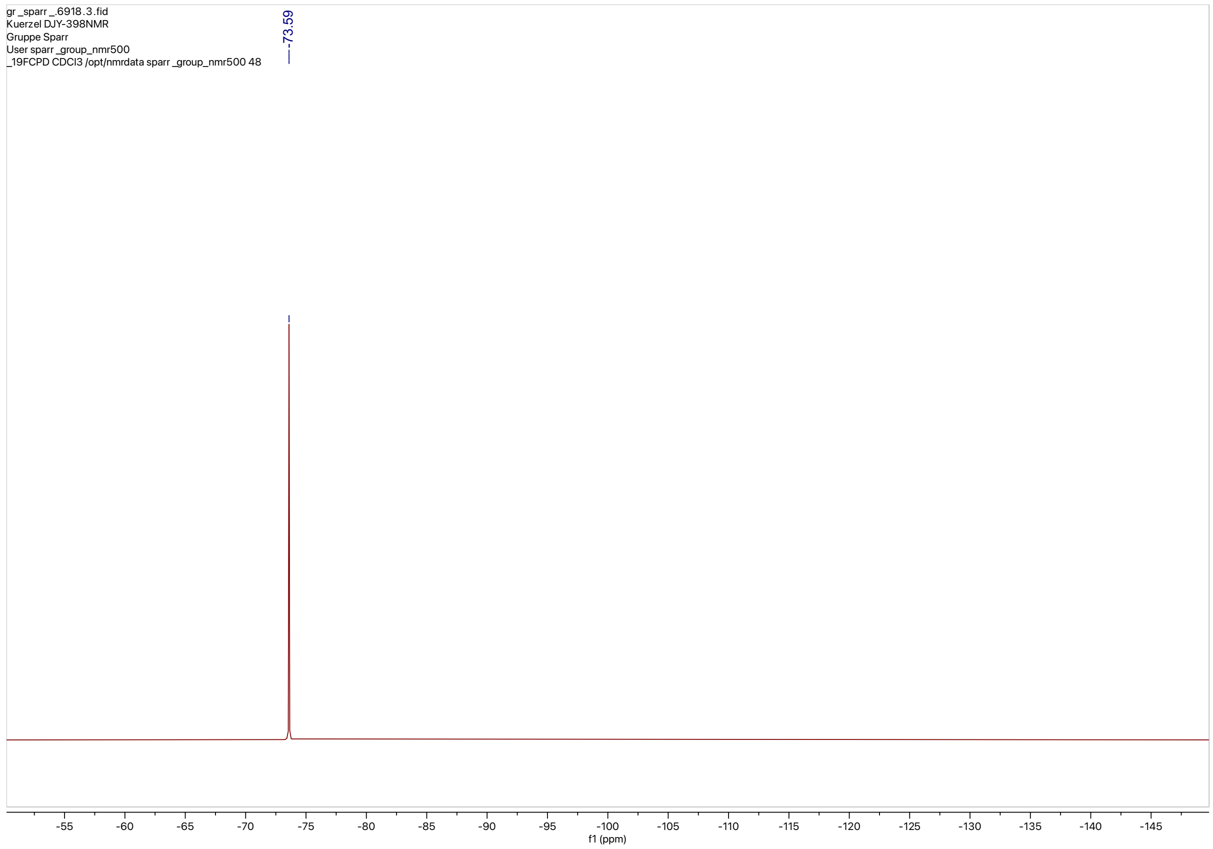

Supplementary Figure 31. ^{1}H (500 MHz, $CDCl_3$, 25 °C), $^{13}C\{^{1}H\}$ (151 MHz, $CDCl_3$, 25 °C) and $^{19}F\{^{1}H\}$ ((470 MHz, $CDCl_3$, 25 °C) spectra of **S3c**

^{1}H, ^{13}C{^{1}H}, ^{19}F{^{1}H} and ^{31}P{^{1}H} spectra of [*TBPY*-5-11']-5,5'-Dimethyl-1-hydro-3,3,3',3'-tetrakis(trifluoromethyl)-3*H*,3'*H*-1λ^{5},1'-spirobi[ben-zo[*c*][2,1]oxaphosphole] (S4c)

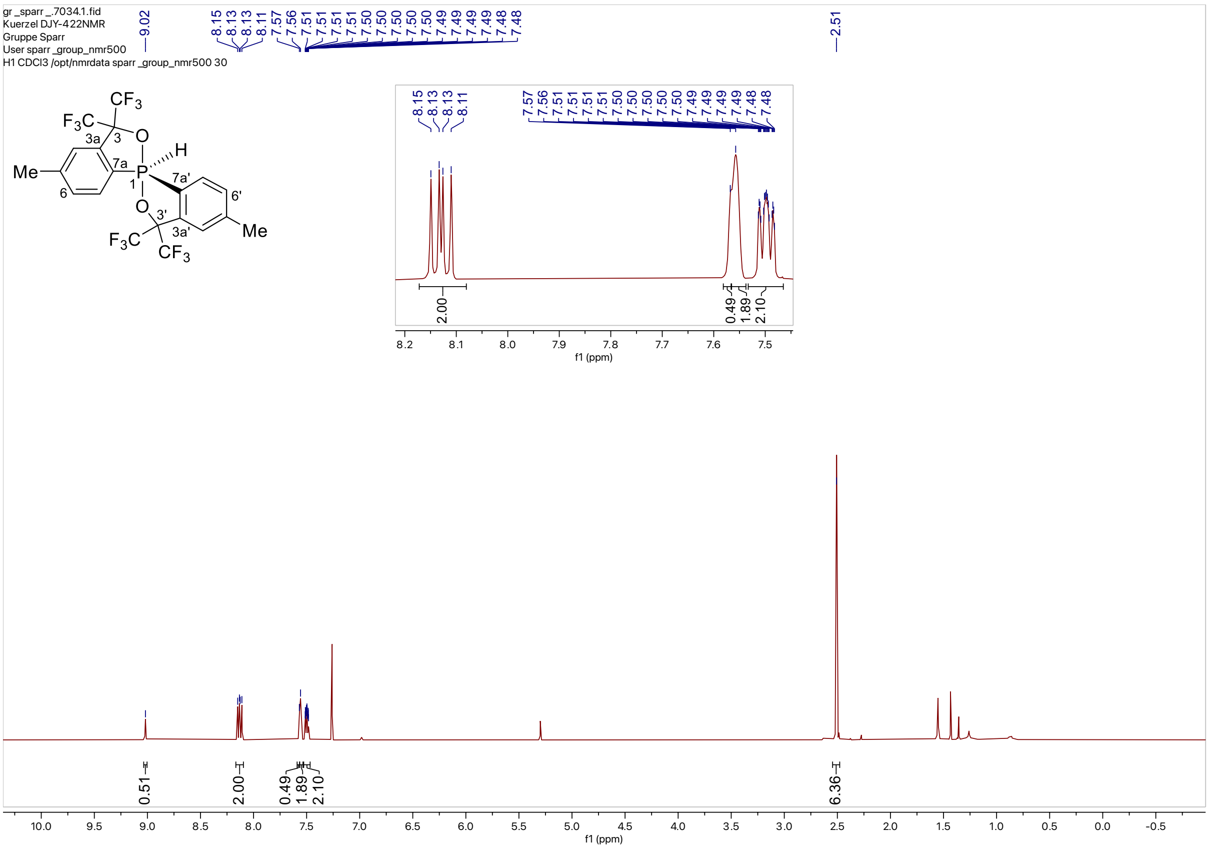

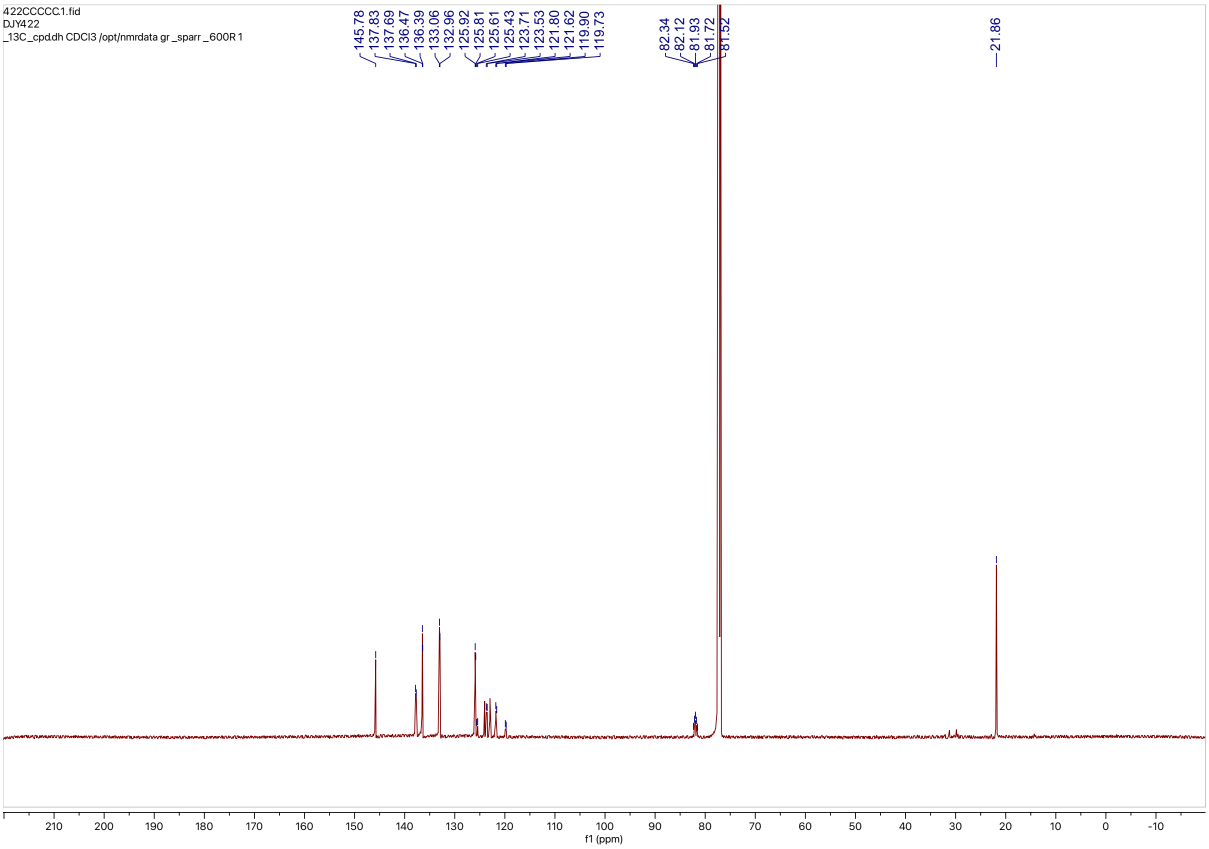

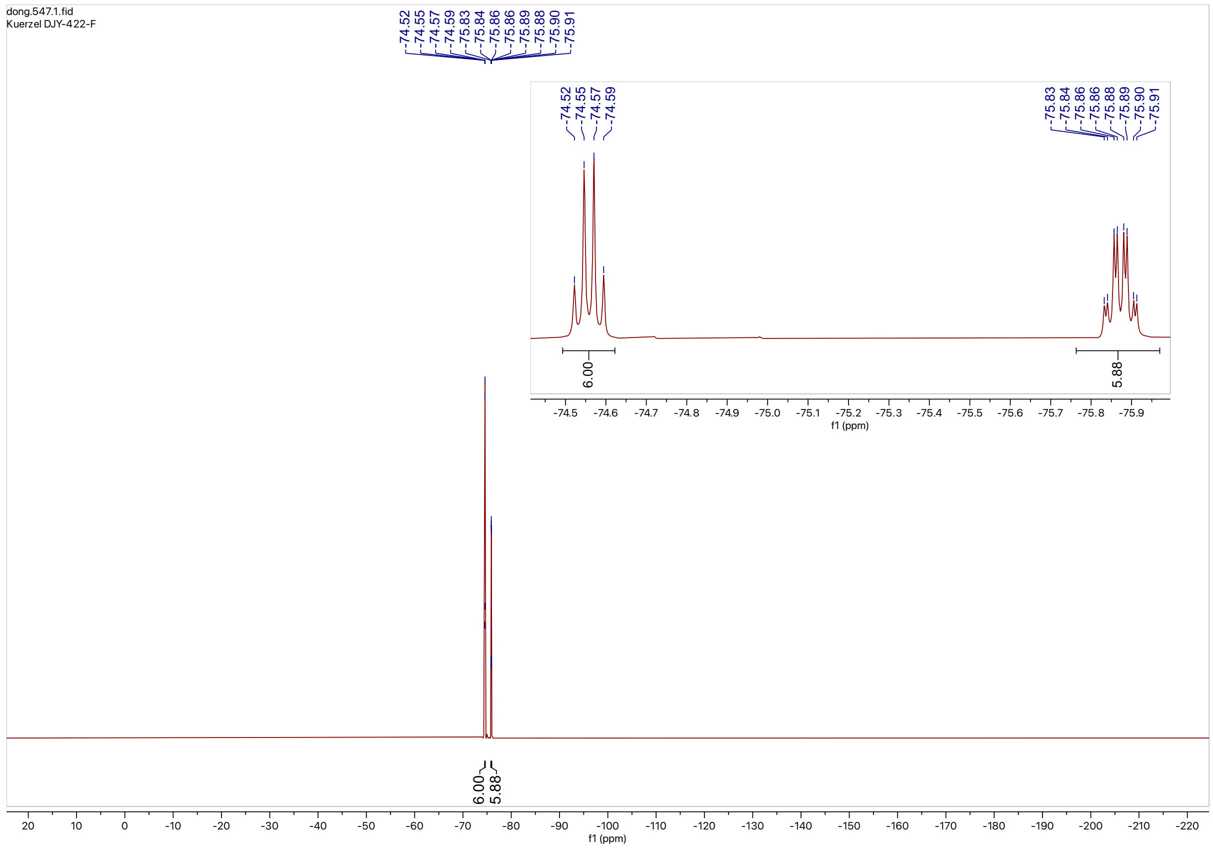

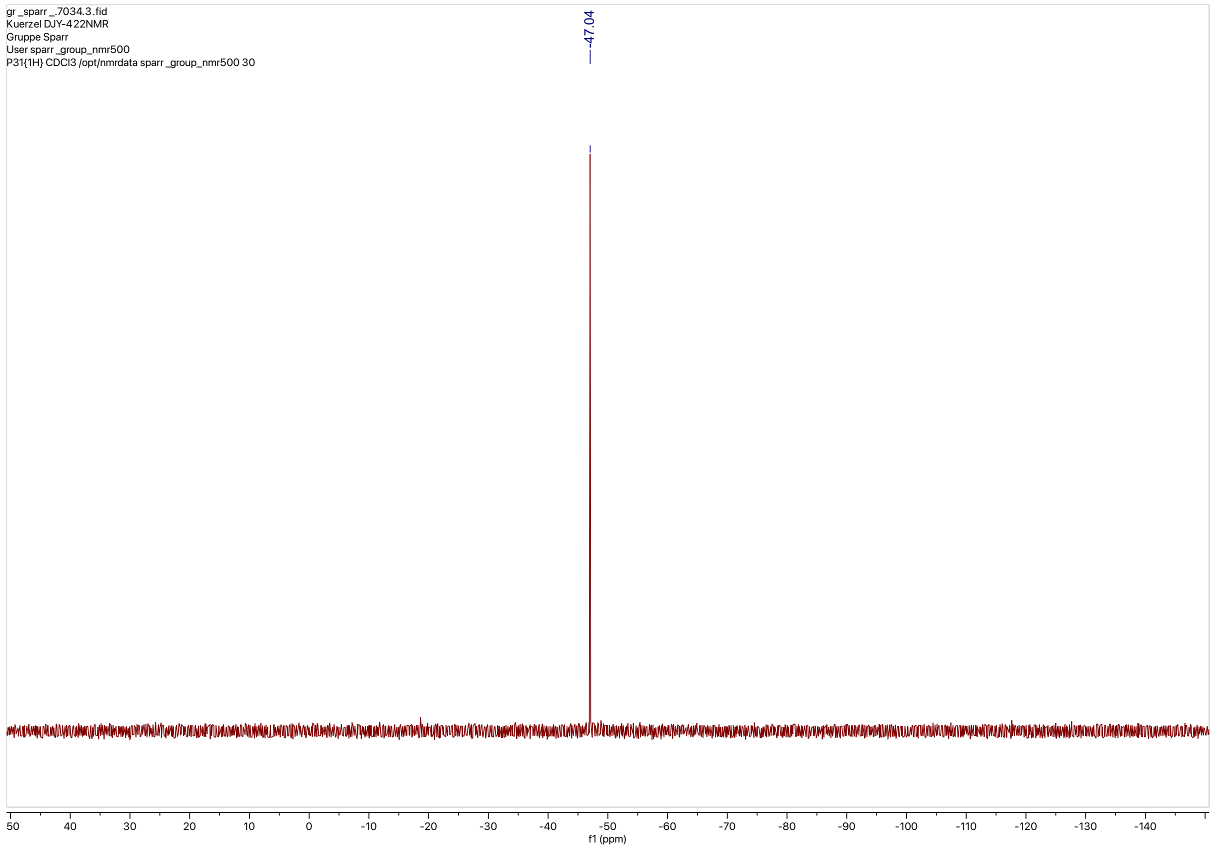

Supplementary Figure 32. ^{1}H (500 MHz, $CDCl_3$, 25 °C), $^{13}C\{^{1}H\}$ (151 MHz, $CDCl_3$, 25 °C), $^{19}F\{^{1}H\}$ (376 MHz, $CDCl_3$, 25 °C) and $^{31}P\{^{1}H\}$ (202 MHz, $CDCl_3$, 25 °C) spectra of **S4c**

^{1}H, ^{19}F{^{1}H} and ^{31}P{^{1}H} spectra of [*TBPY*-5-15]-1,1,1,3,3,3-Hexafluoro-2-(2-(1-butyl-1-hydro-5-methyl-3,3-bis(trifluoromethyl)-3*H*-1λ^{5}-benzo[*c*][2,1]oxaphosphol-1-yl)-5-methylphenyl)propan-2-ol (1c)

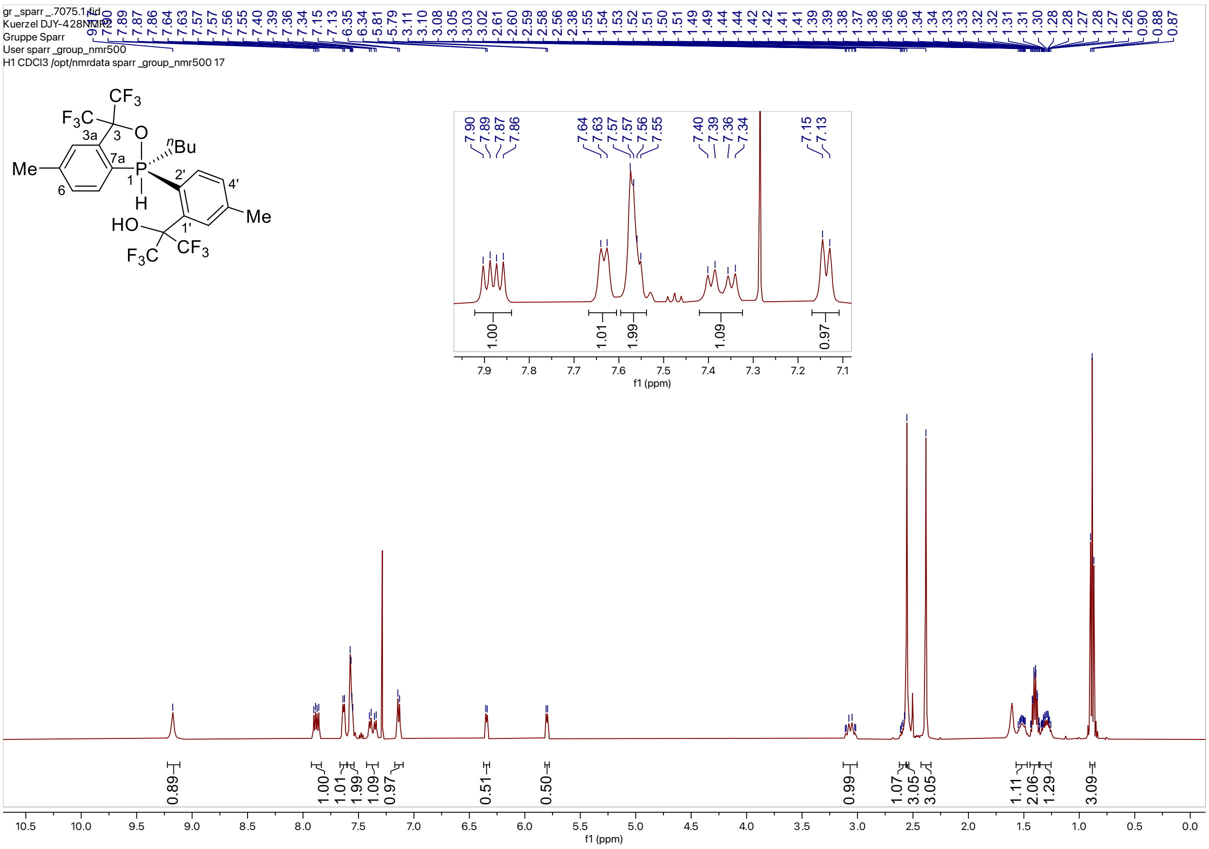

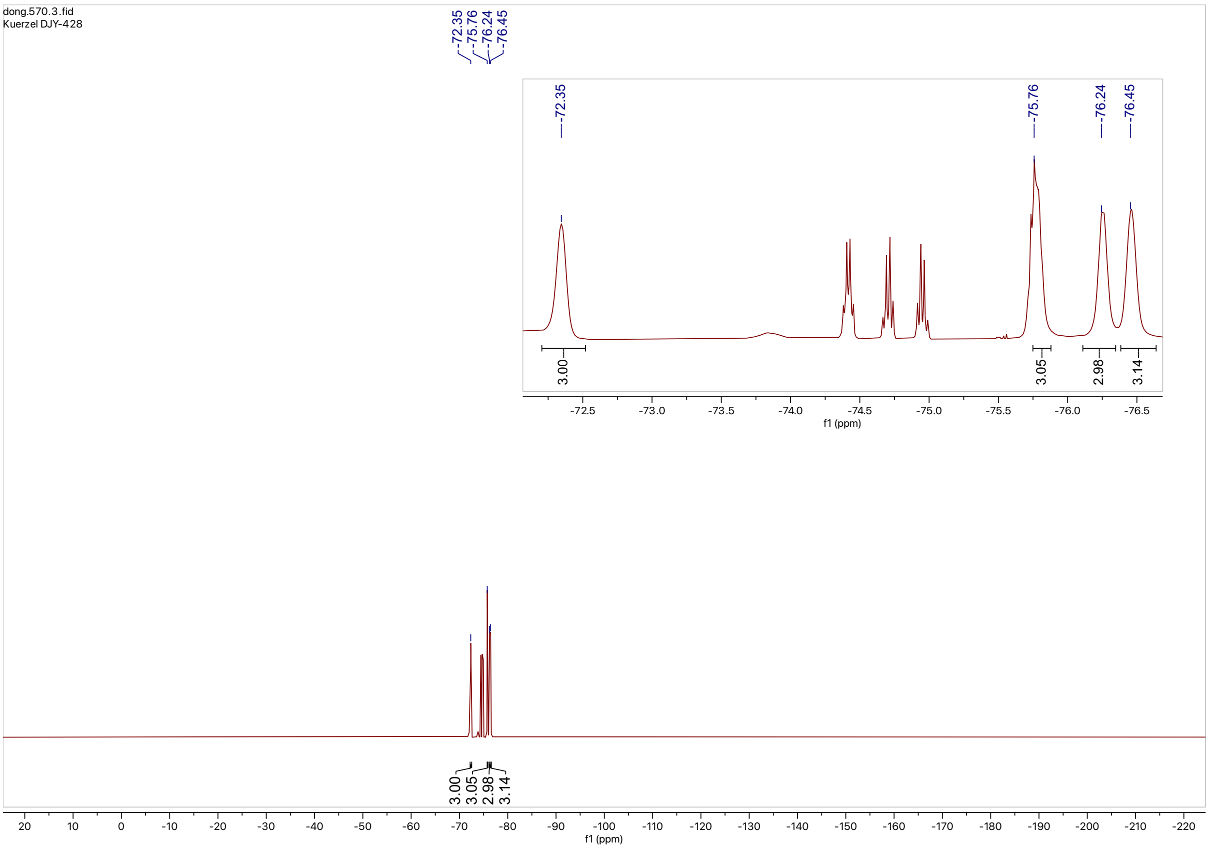

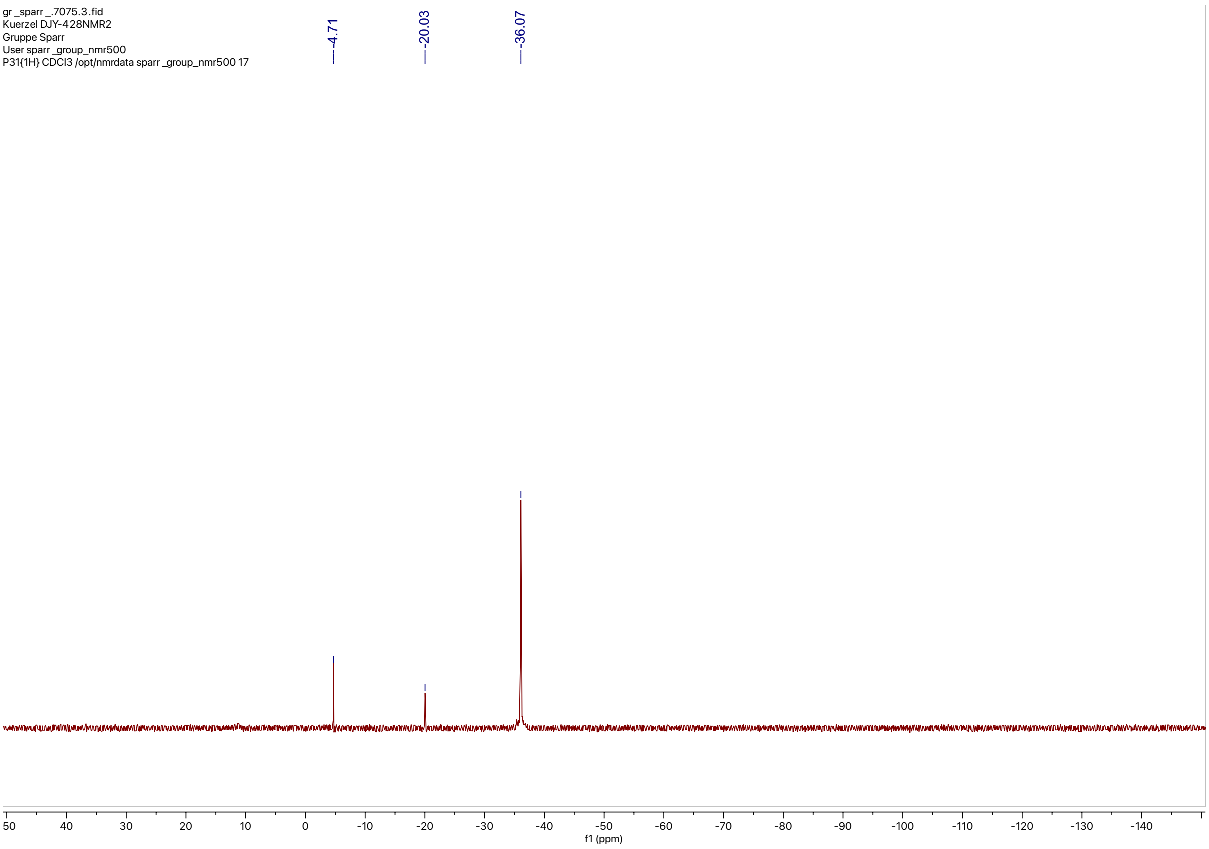

Supplementary Figure 33. ^{1}H (500 MHz, $CDCl_3$, 25 °C), $^{19}F\{^{1}H\}$ (376 MHz, $CDCl_3$, 25 °C) and $^{31}P\{^{1}H\}$ (202 MHz, $CDCl_3$, 25 °C) spectra of **1c**

^1H, $^{13}C\{^1H\}$ and $^{19}F\{^1H\}$ **spectra of 2,3,4,5,6-Pentafluorophenyl 2-bromo-5-methoxybenzoate (S1d)**

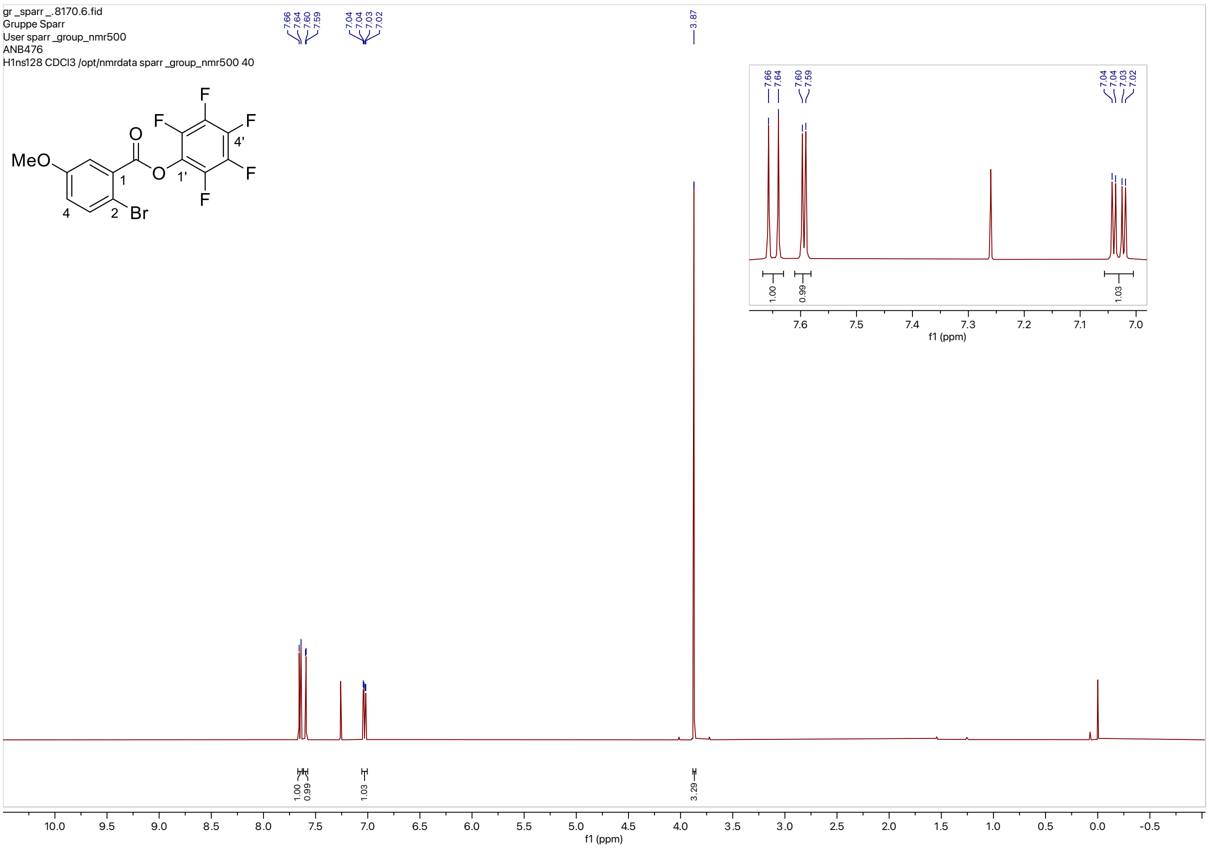

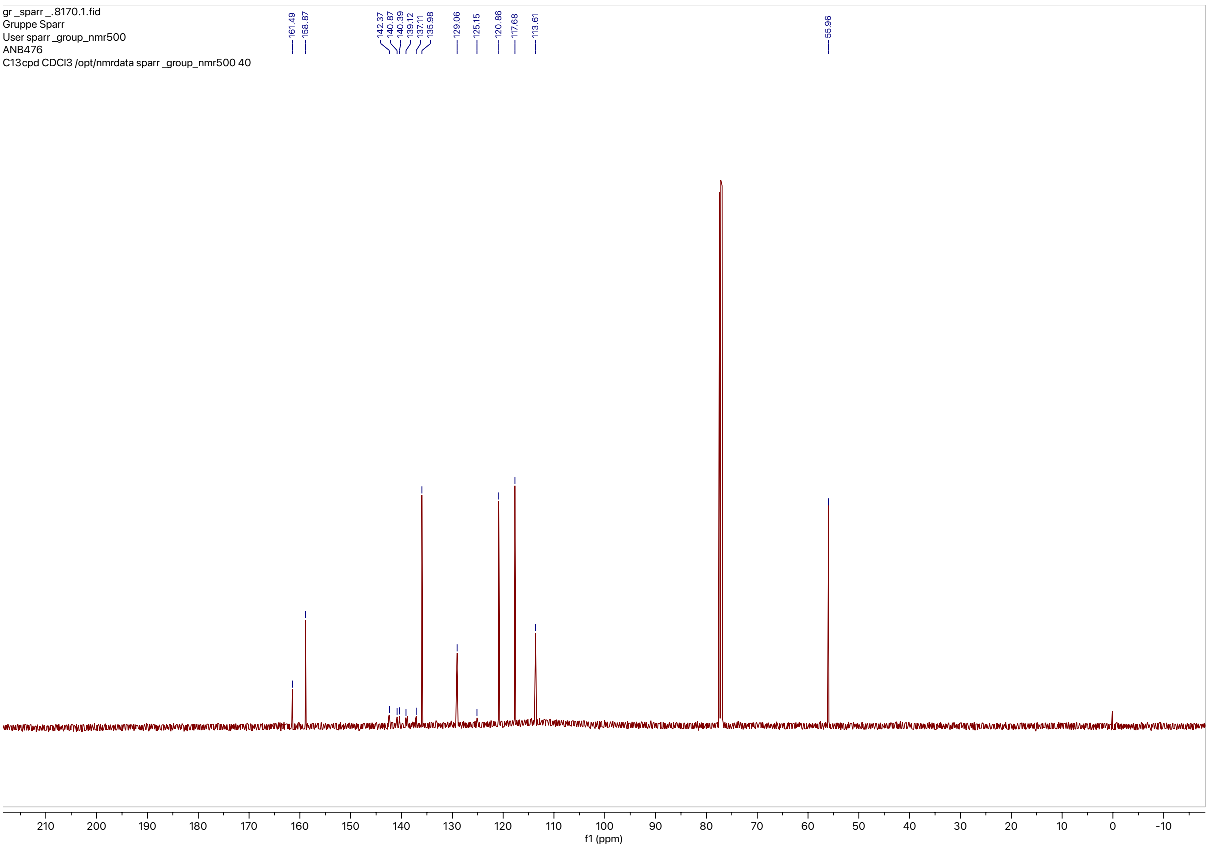

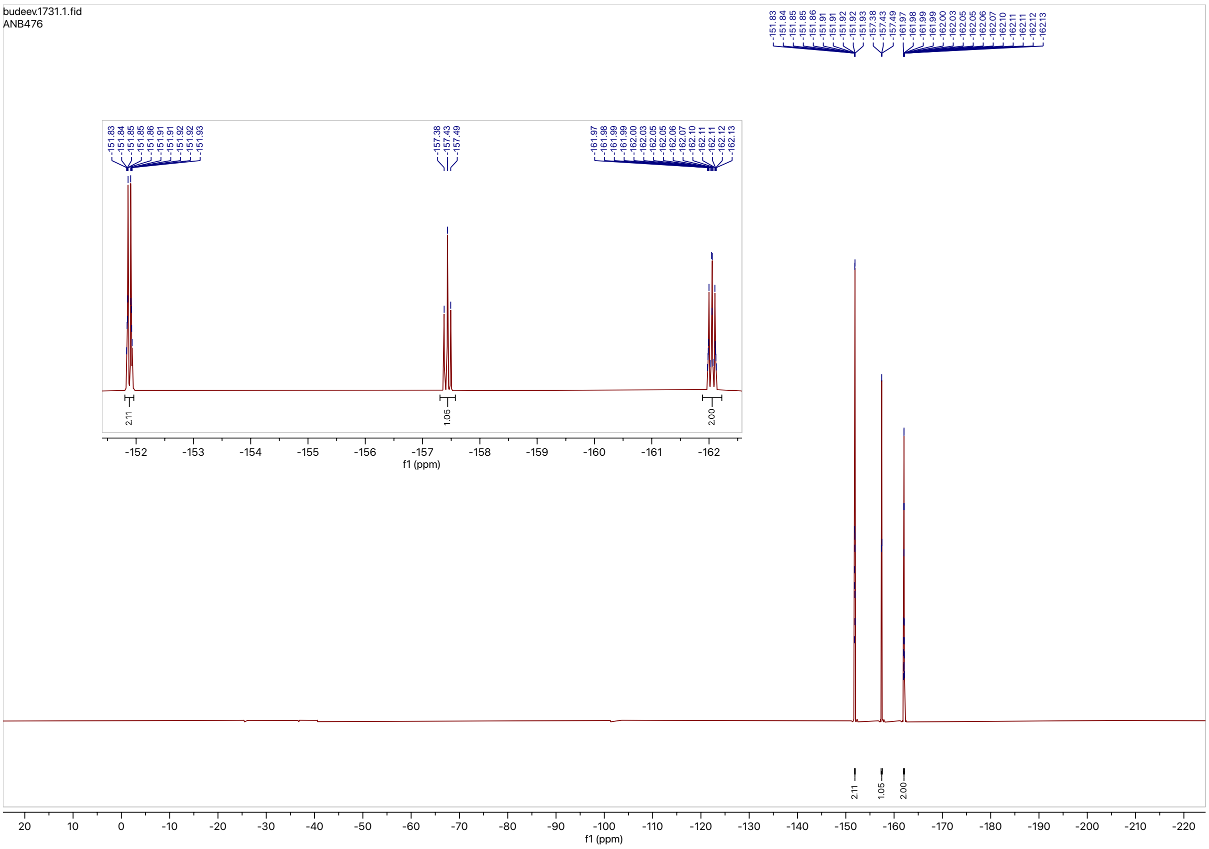

Supplementary Figure 34. ^{1}H (500 MHz, $CDCl_3$, 25 °C), $^{13}C\{^{1}H\}$ (126 MHz, $CDCl_3$, 25 °C) and $^{19}F\{^{1}H\}$ (376 MHz, $CDCl_3$, 25 °C) spectra of **S1d**

^{1}H, $^{13}C\{^{1}H\}$ and $^{19}F\{^{1}H\}$ spectra of ((2-(2-Bromo-5-methoxyphenyl)-1,1,1,3,3,3-hexafluoropropan-2-yl)oxy)trimethylsilane (S2d)

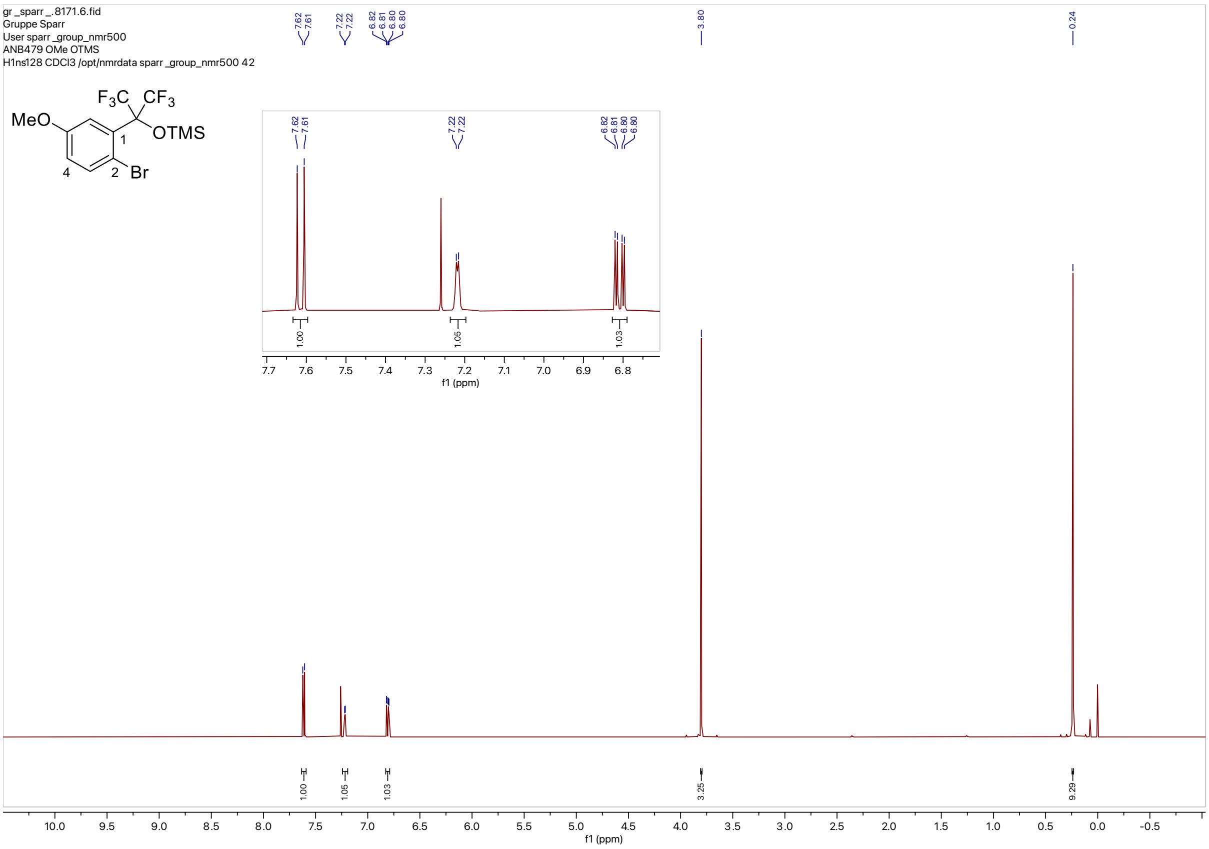

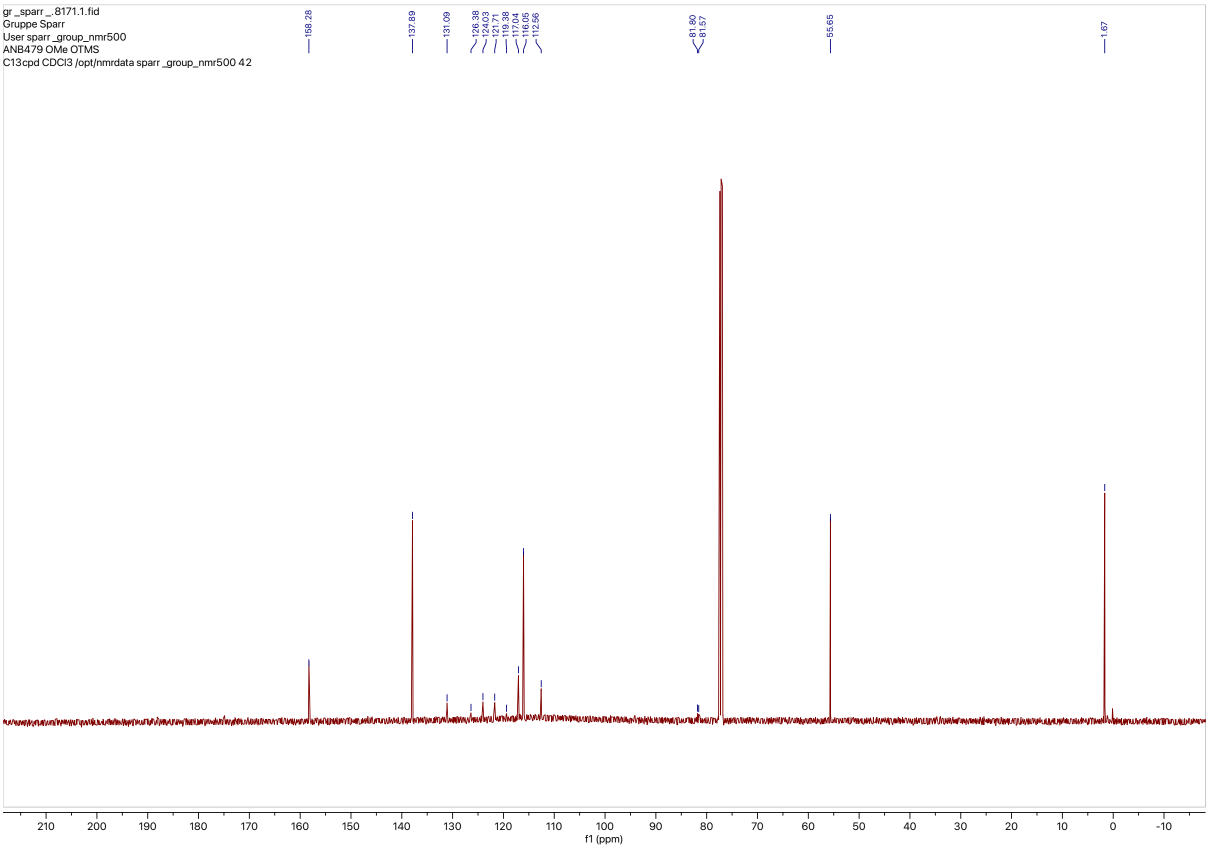

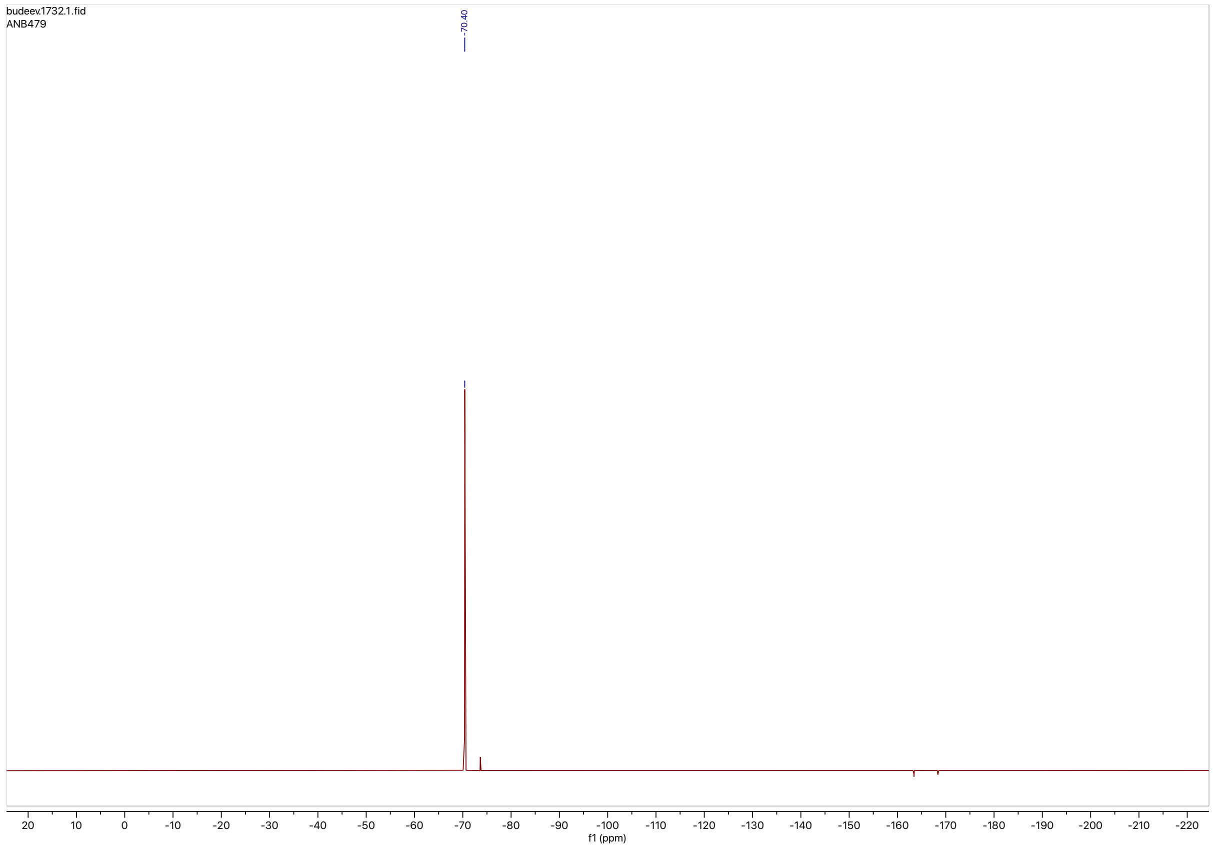

Supplementary Figure 35. ^{1}H (500 MHz, $CDCl_3$, 25 °C), $^{13}C\{^{1}H\}$ (126 MHz, $CDCl_3$, 25 °C) and $^{19}F\{^{1}H\}$ (376 MHz, $CDCl_3$, 25 °C) spectra of **S2d**

^{1}H, ^{13}C{^{1}H} and ^{19}F{^{1}H} spectra of 2-(2-Bromo-5-methoxyphenyl)-1,1,1,3,3,3-hexafluoropropan-2-ol (S3d)

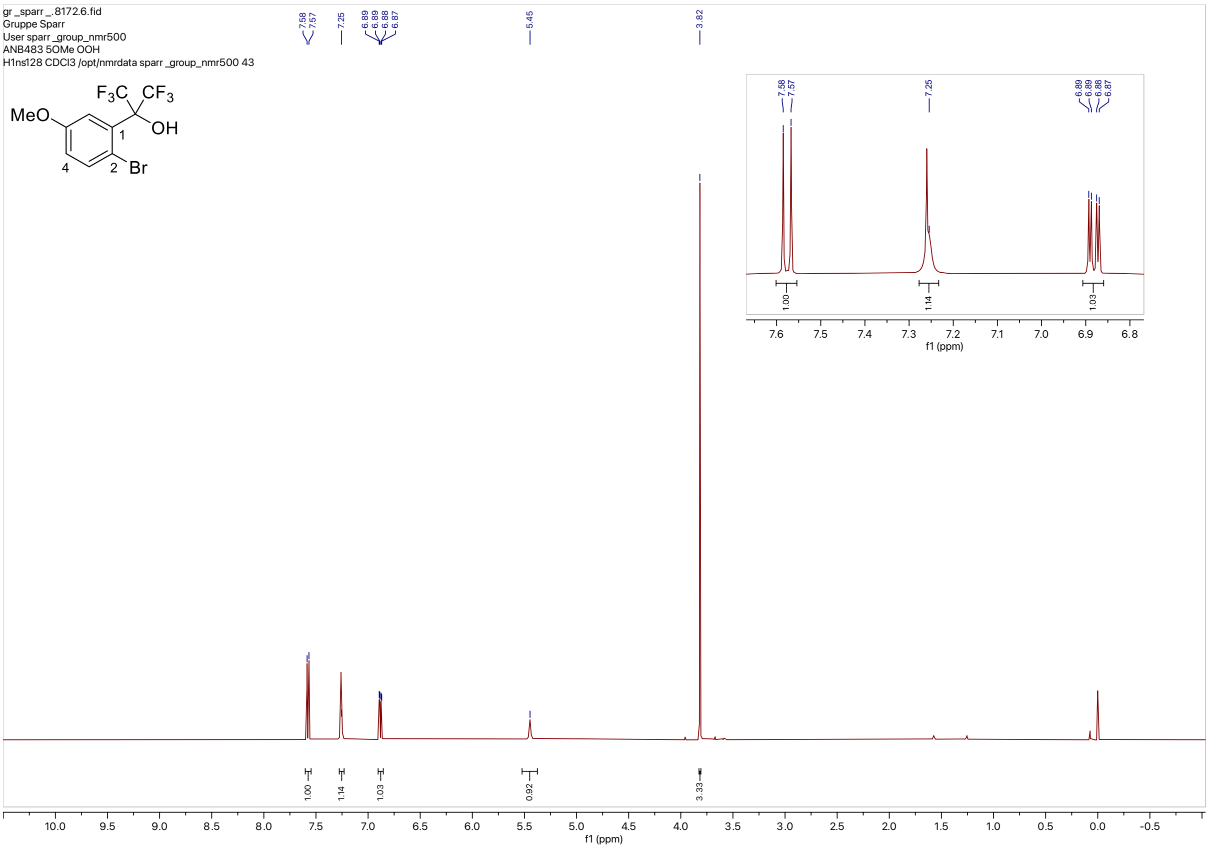

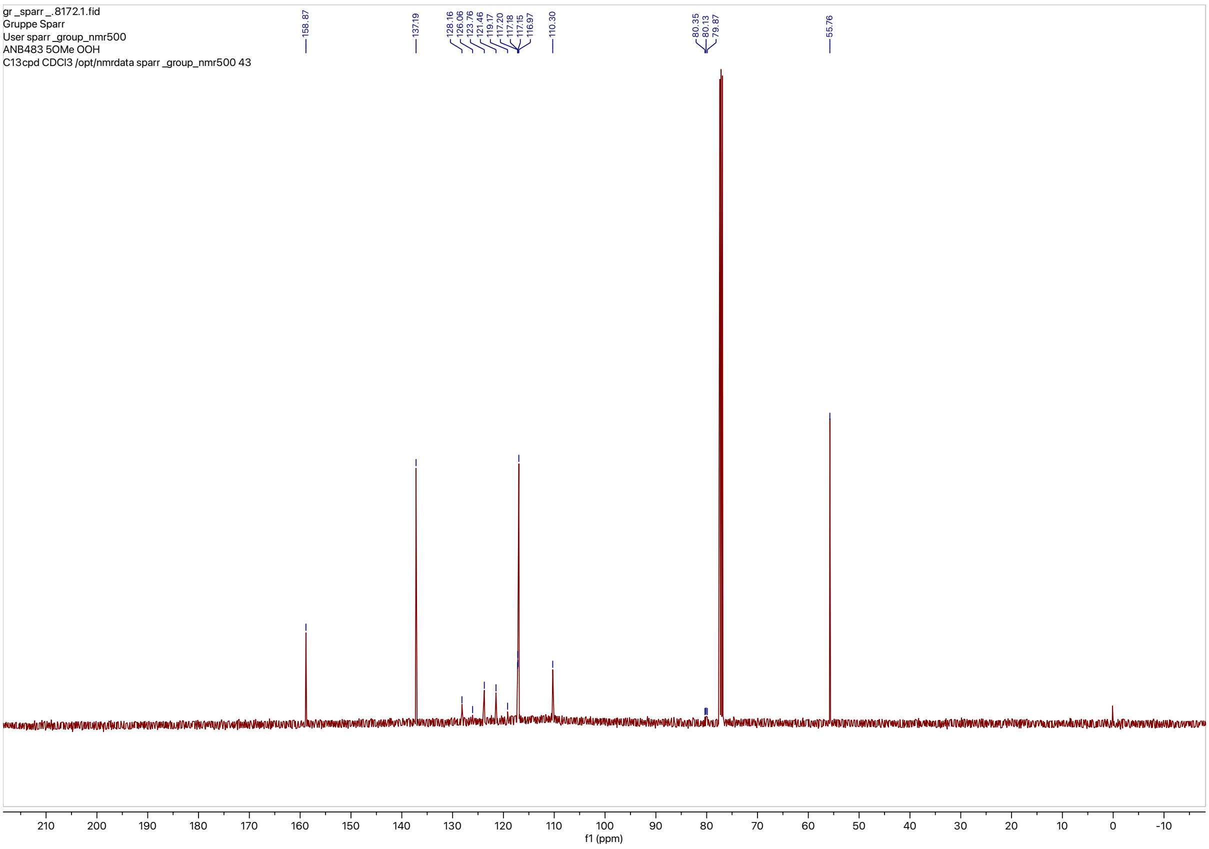

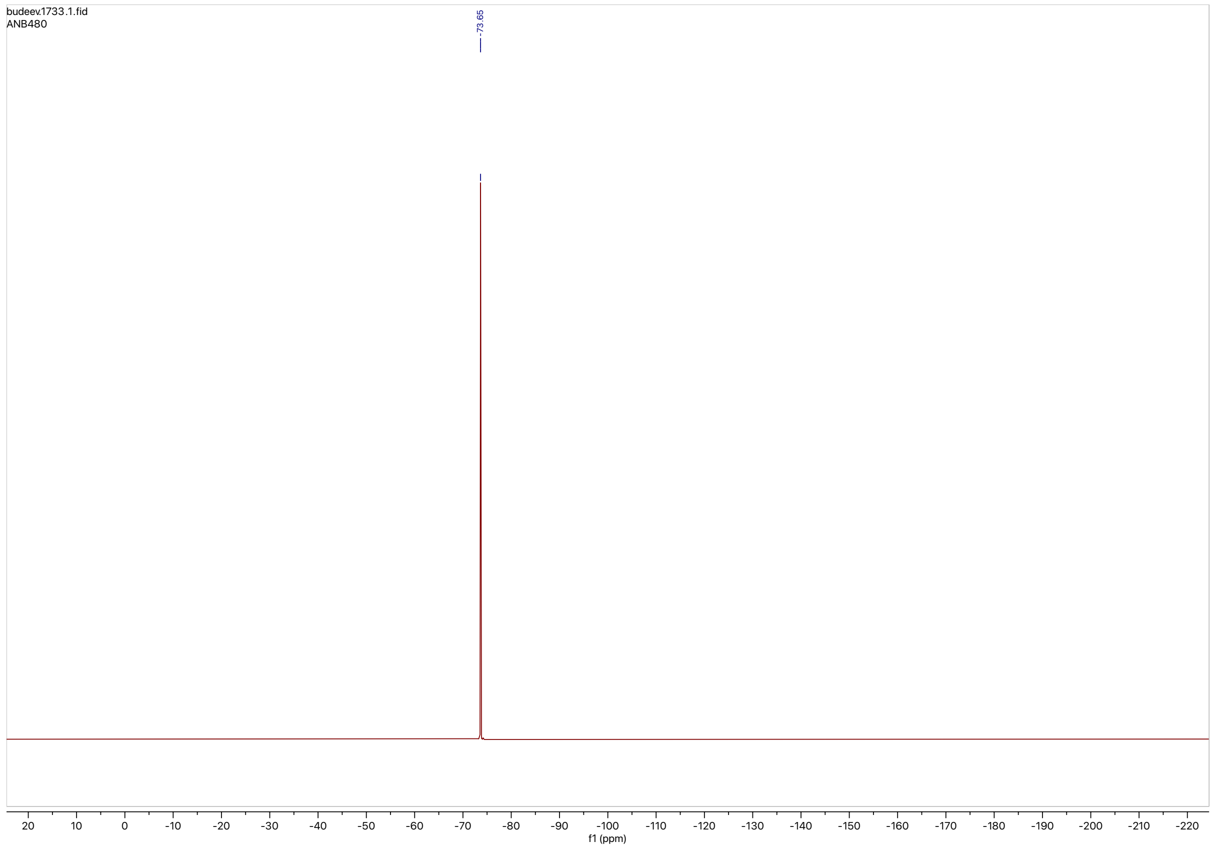

Supplementary Figure 36. ^{1}H (500 MHz, $CDCl_3$, 25 °C), $^{13}C\{^{1}H\}$ (126 MHz, $CDCl_3$, 25 °C) and $^{19}F\{^{1}H\}$ (376 MHz, $CDCl_3$, 25 °C) spectra of **S3d**

^{1}H, ^{13}C{^{1}H}, ^{19}F{^{1}H} and ^{31}P{^{1}H} spectra of [*TBPY*-5-11']-5,5'-Dimethoxy-1-hydro-3,3,3',3'-tetrakis(trifluoromethyl)-3*H*,3'*H*-1λ^{5},1'-spirobi[benzo[*c*][2,1]oxaphosphole] (S4d)

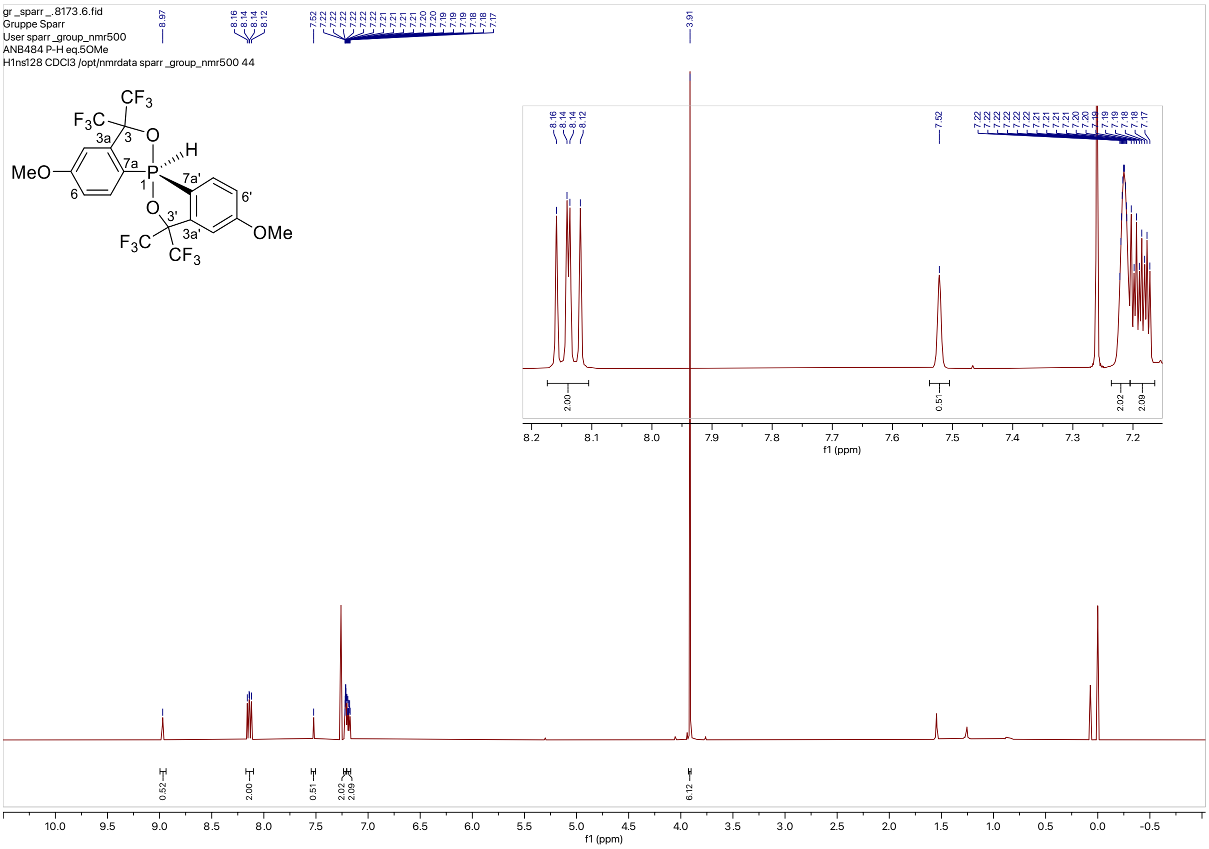

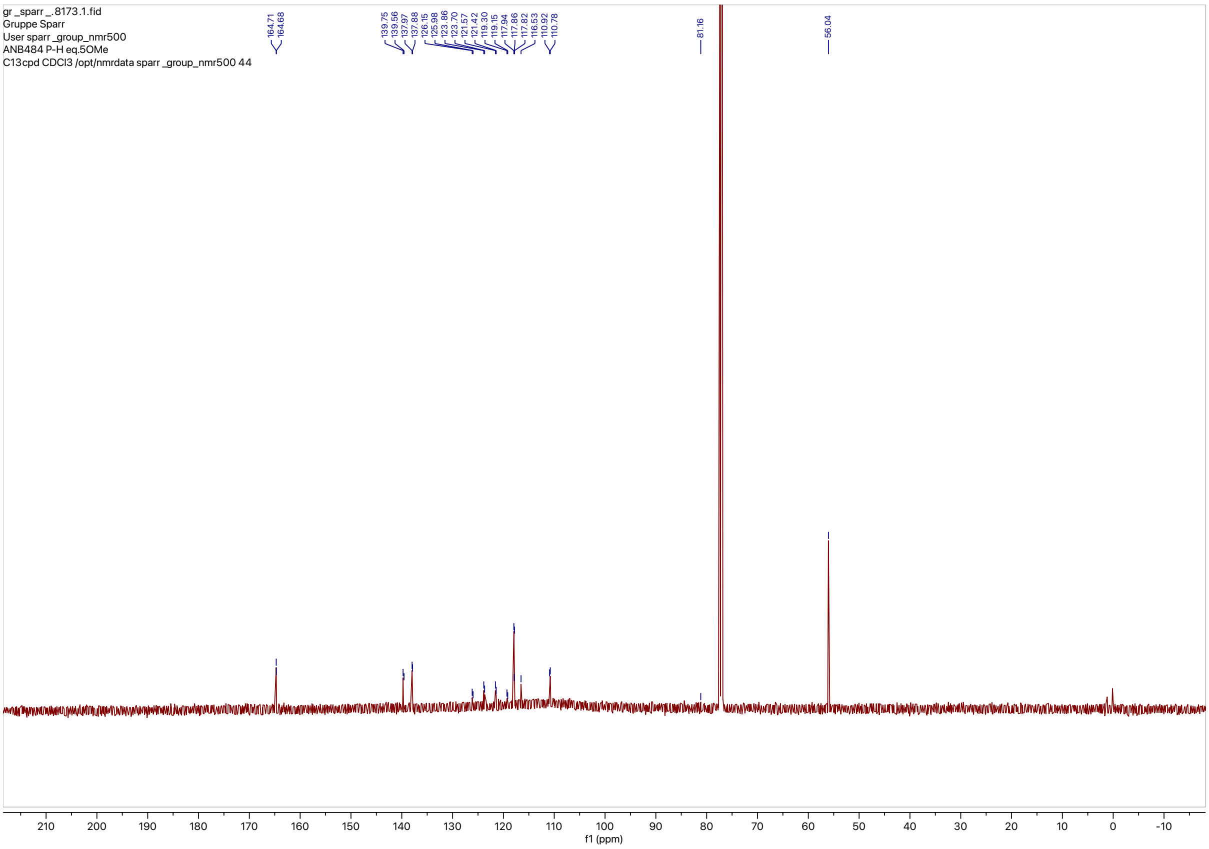

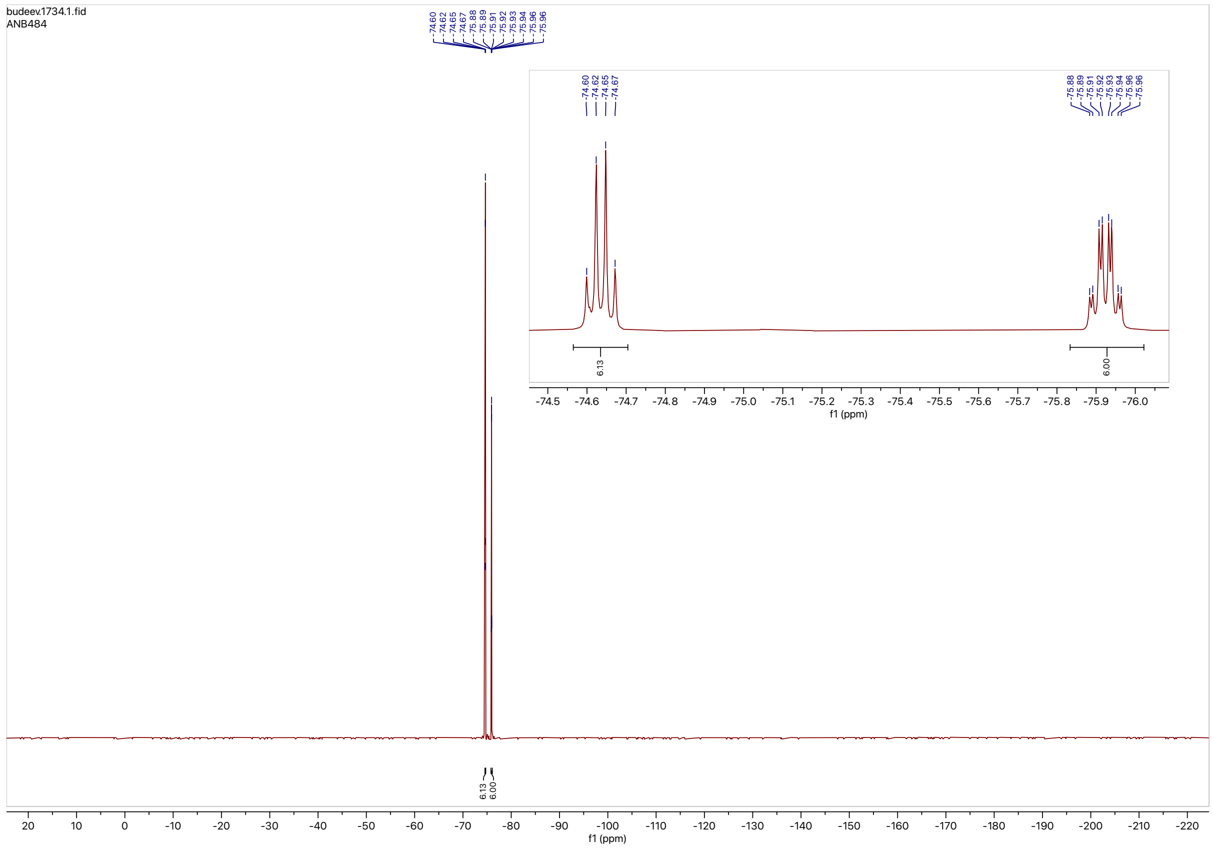

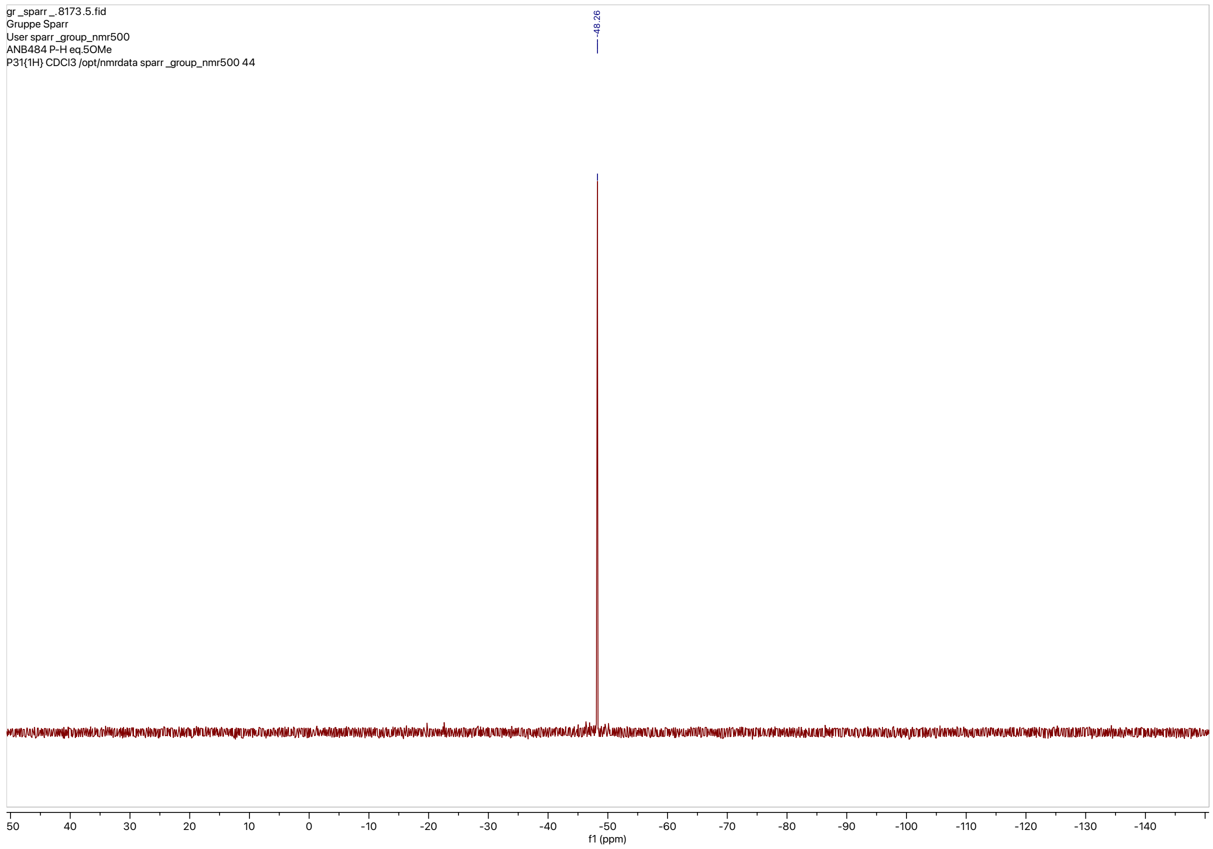

Supplementary Figure 37. ^{1}H (500 MHz, $CDCl_3$, 25 °C), $^{13}C\{^{1}H\}$ (126 MHz, $CDCl_3$, 25 °C), $^{19}F\{^{1}H\}$ (376 MHz, $CDCl_3$, 25 °C) and $^{31}P\{^{1}H\}$ (202 MHz, $CDCl_3$, 25 °C) spectra of **S4d**

^{1}H, ^{19}F{^{1}H} and ^{31}P{^{1}H} spectra of [*TBPY*-5-15]-1,1,1,3,3,3-Hexafluoro-2-(2-(1-butyl-1-hydro-5-methoxy-3,3-bis(trifluoromethyl)-3*H*-1λ^{5}-benzo[*c*][2,1]oxaphosphol-1-yl)-5-methoxyphenyl)propan-2-ol (1d)

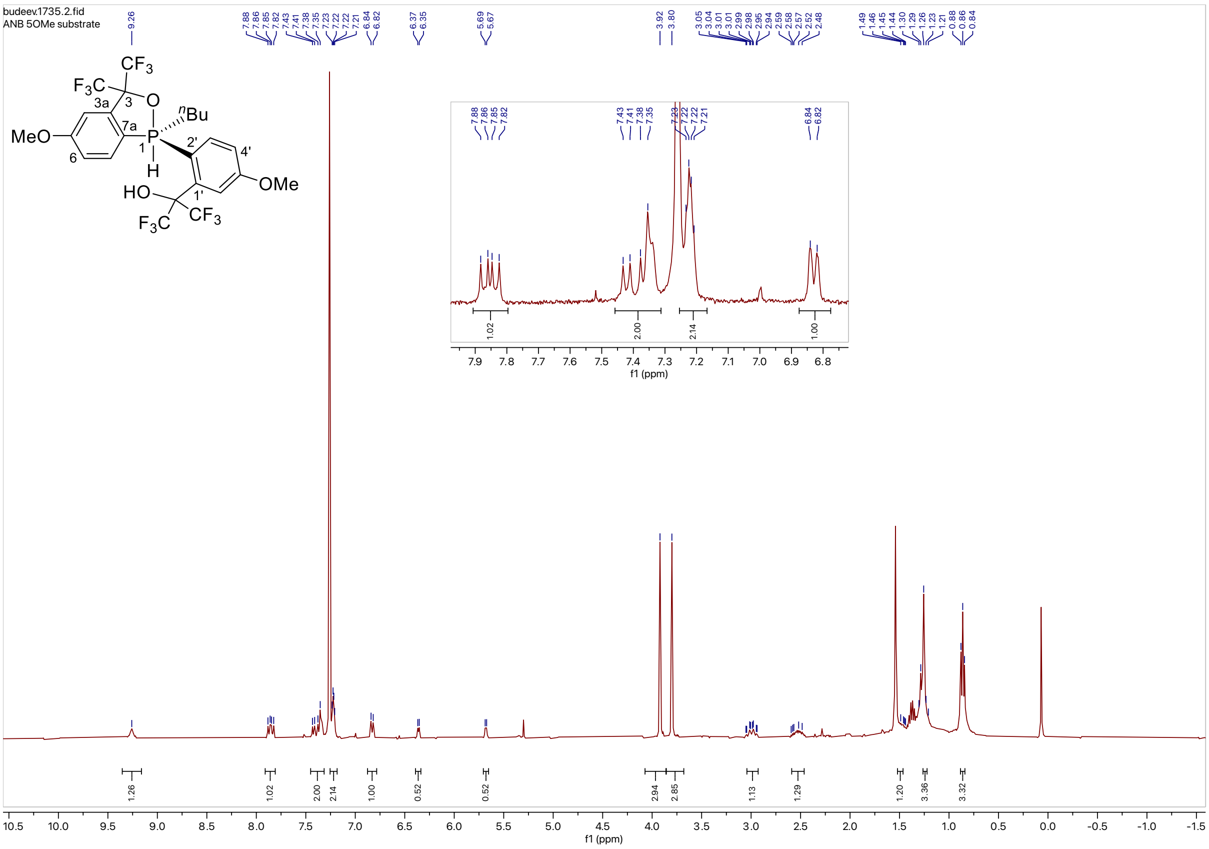

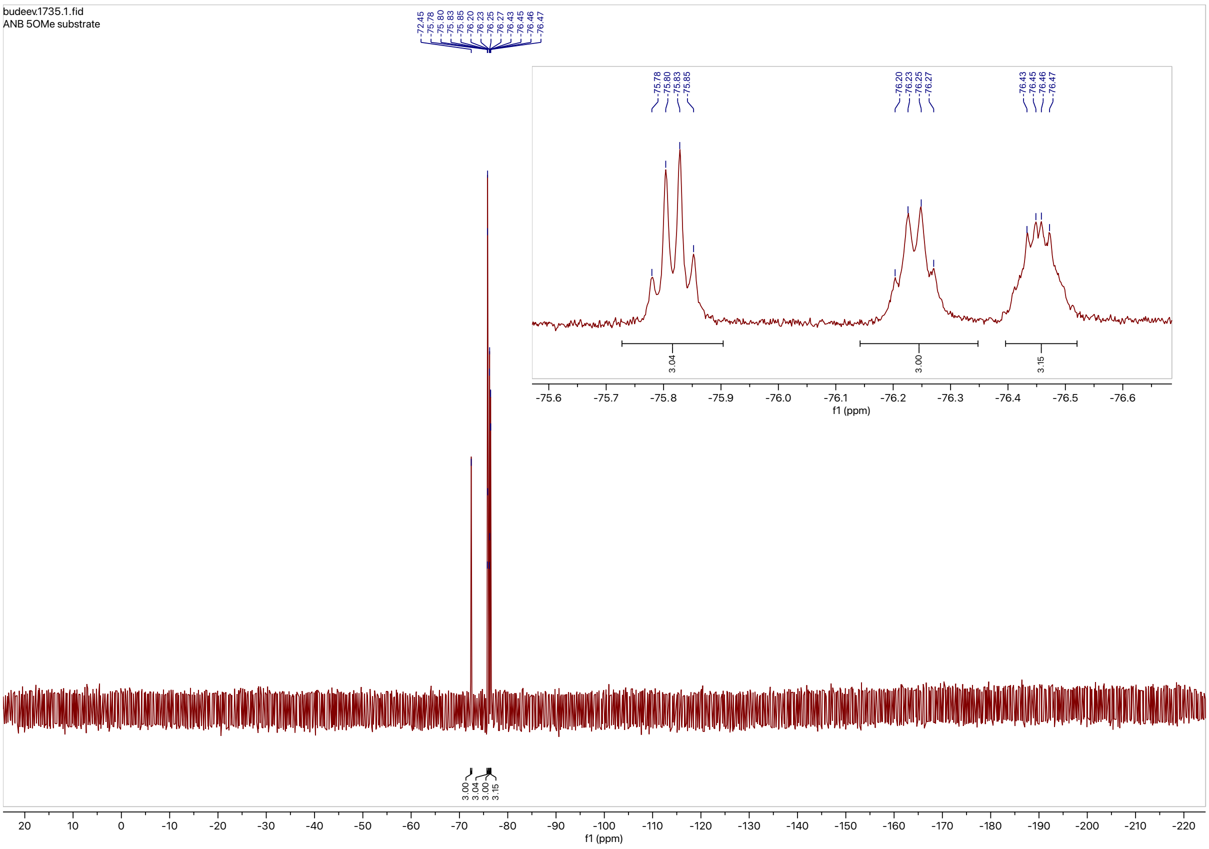

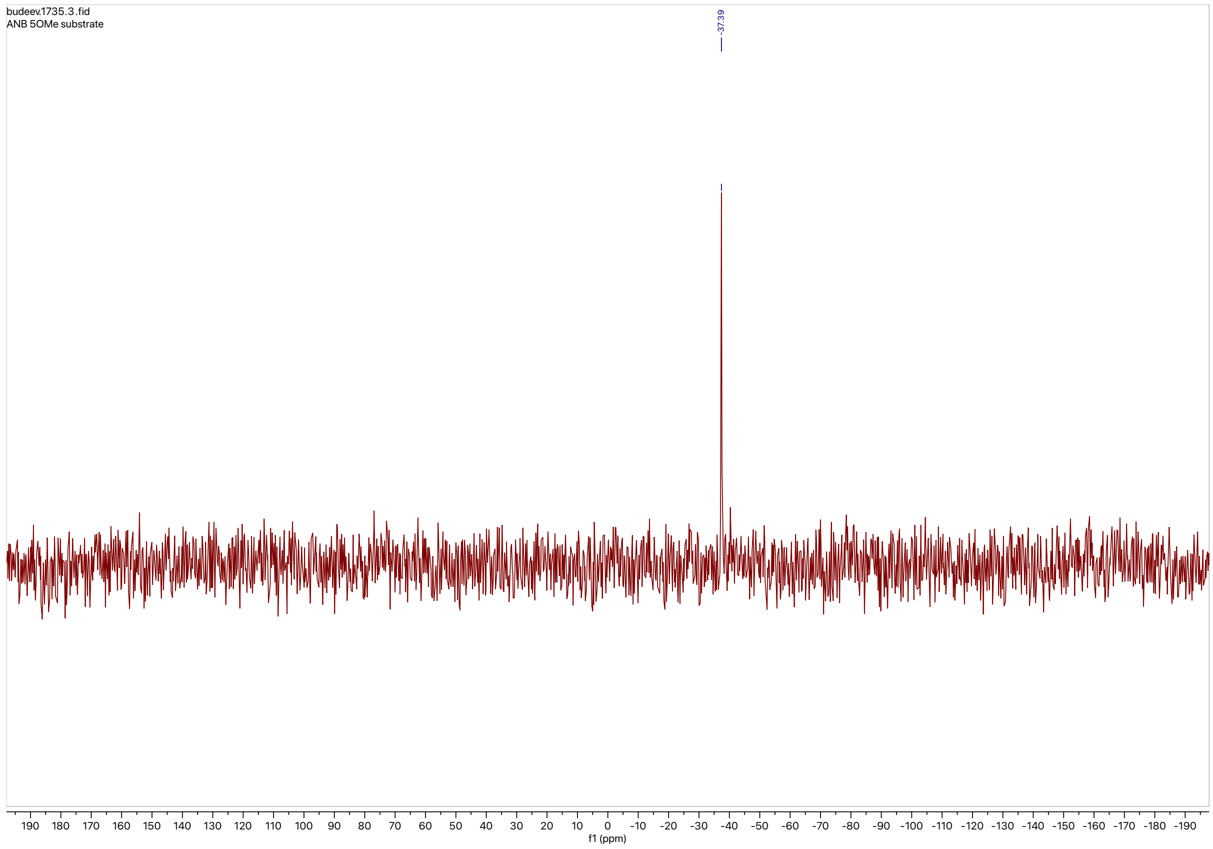

Supplementary Figure 38. ^{1}H (400 MHz, $CDCl_3$, 25 °C), ^{19}F{^{1}H} (376 MHz, $CDCl_3$, 25 °C) and ^{31}P{^{1}H} (162 MHz, $CDCl_3$, 25 °C) spectra of **1d**

^{1}H, $^{13}C\{^{1}H\}$ and $^{19}F\{^{1}H\}$ spectra of 2,3,4,5,6-Pentafluorophenyl 2-bromo-5-methoxy-4-methylbenzoate (S1e)

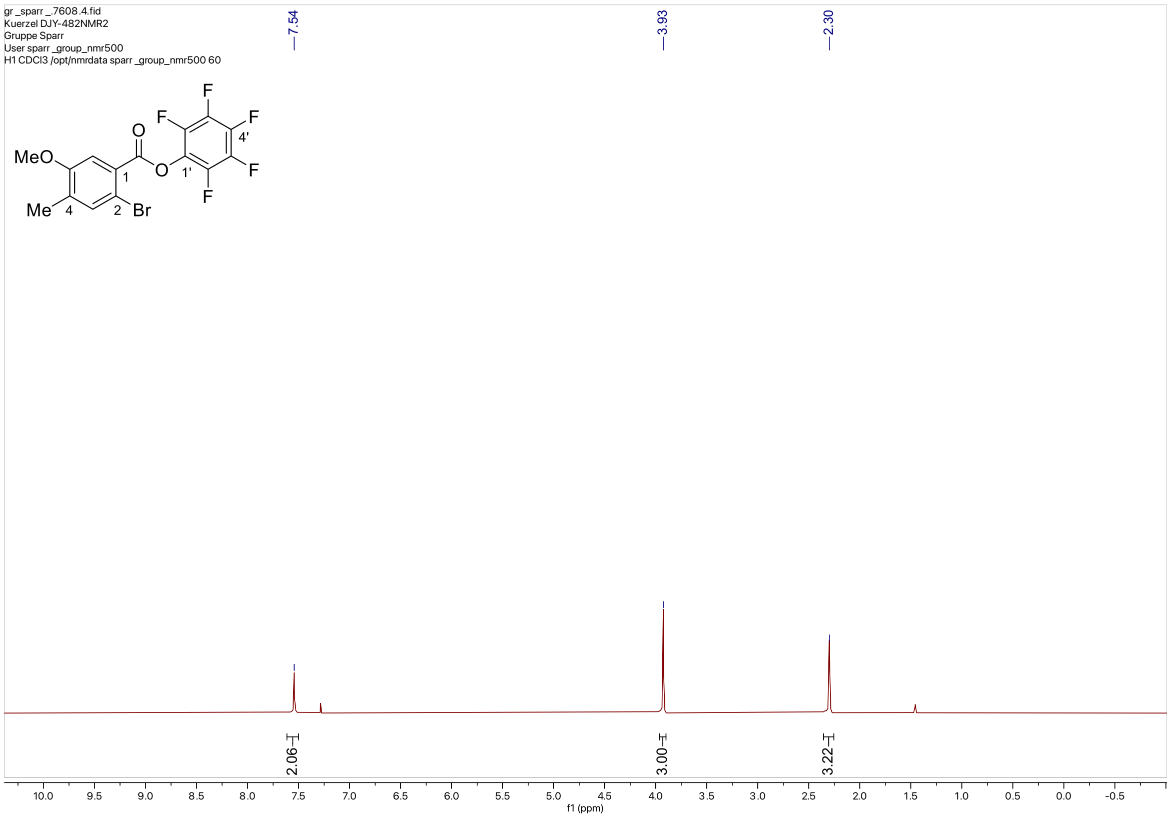

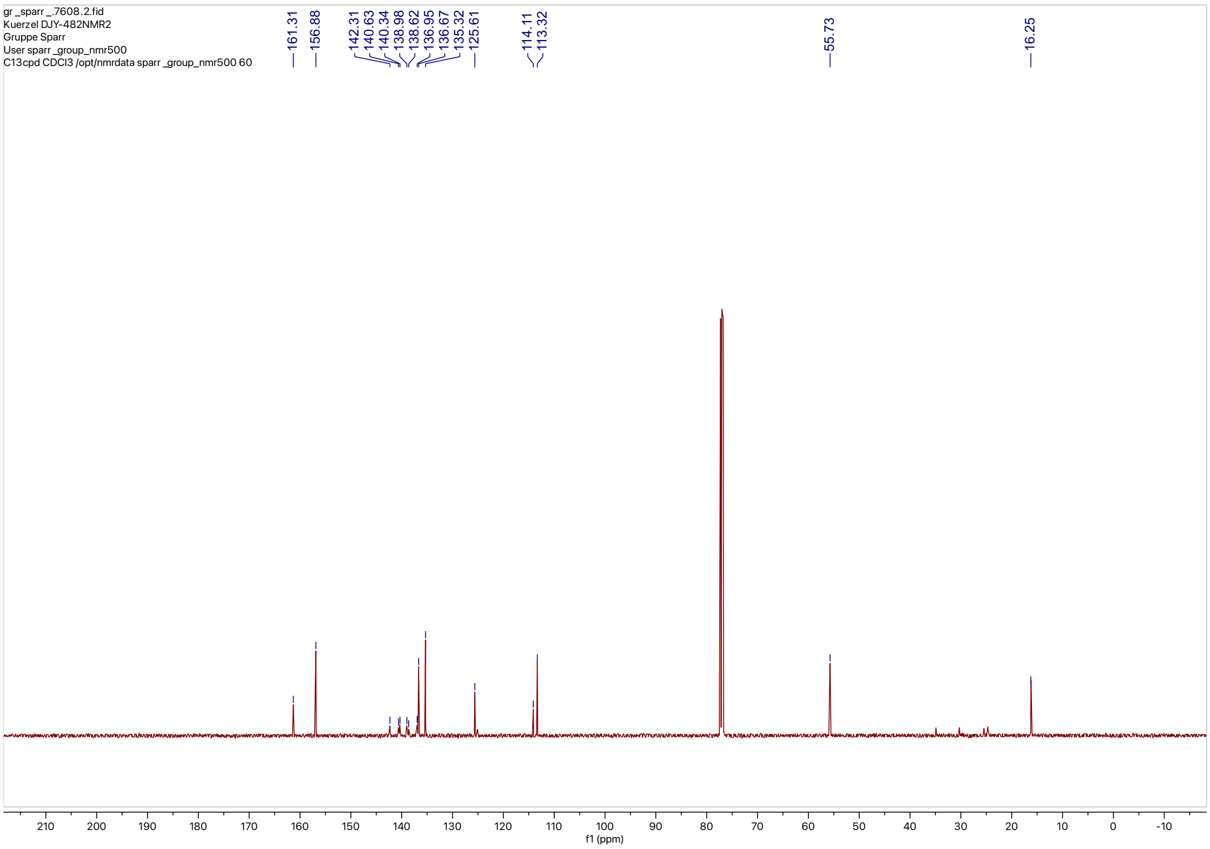

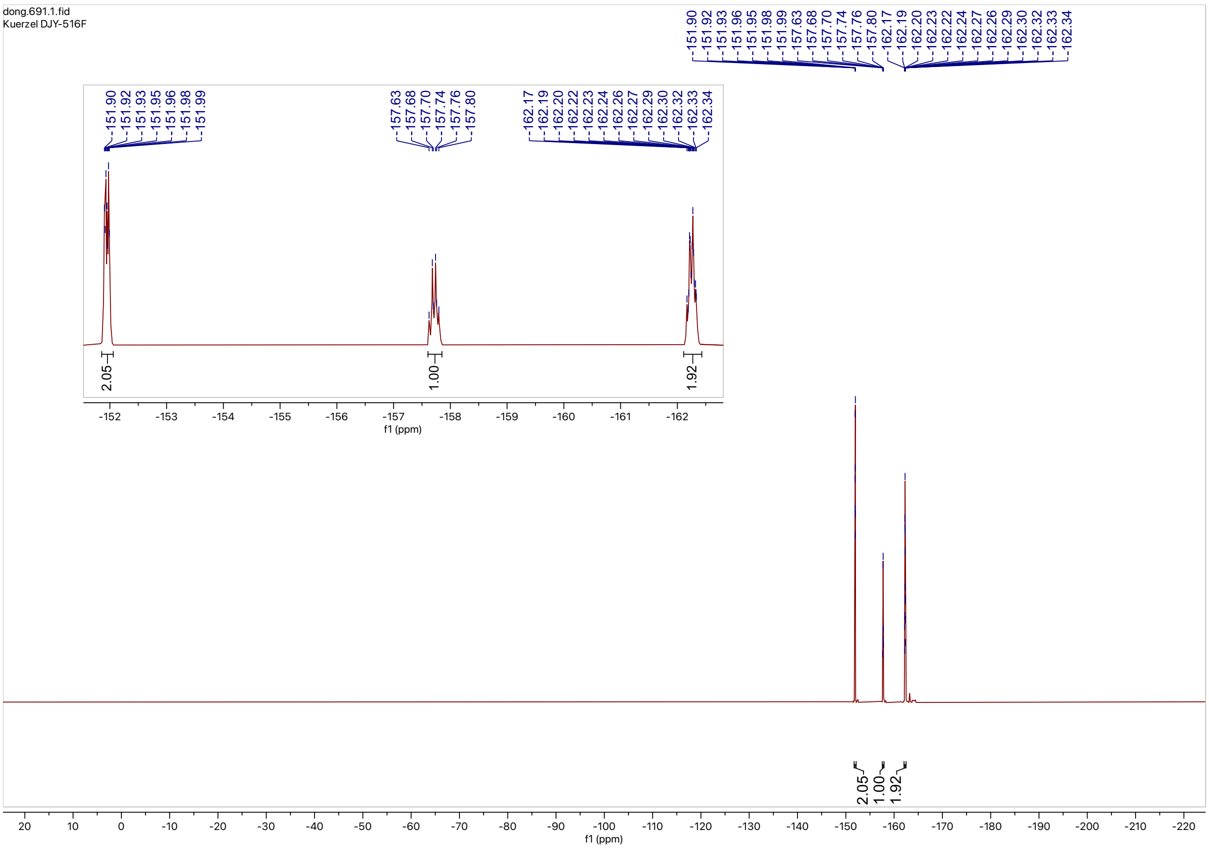

Supplementary Figure 39. ^{1}H (500 MHz, $CDCl_3$, 25 °C), $^{13}C\{^{1}H\}$ (126 MHz, $CDCl_3$, 25 °C) and $^{19}F\{^{1}H\}$ (376 MHz, $CDCl_3$, 25 °C) spectra of **S1e**

[1]H, [13]C{[1]H} and [19]F{[1]H} spectra of ((2-(2-Bromo-5-methoxy-4-methylphenyl)-1,1,1,3,3,3-hexafluoropropan-2-yl)oxy)trimethylsilane (S2e)

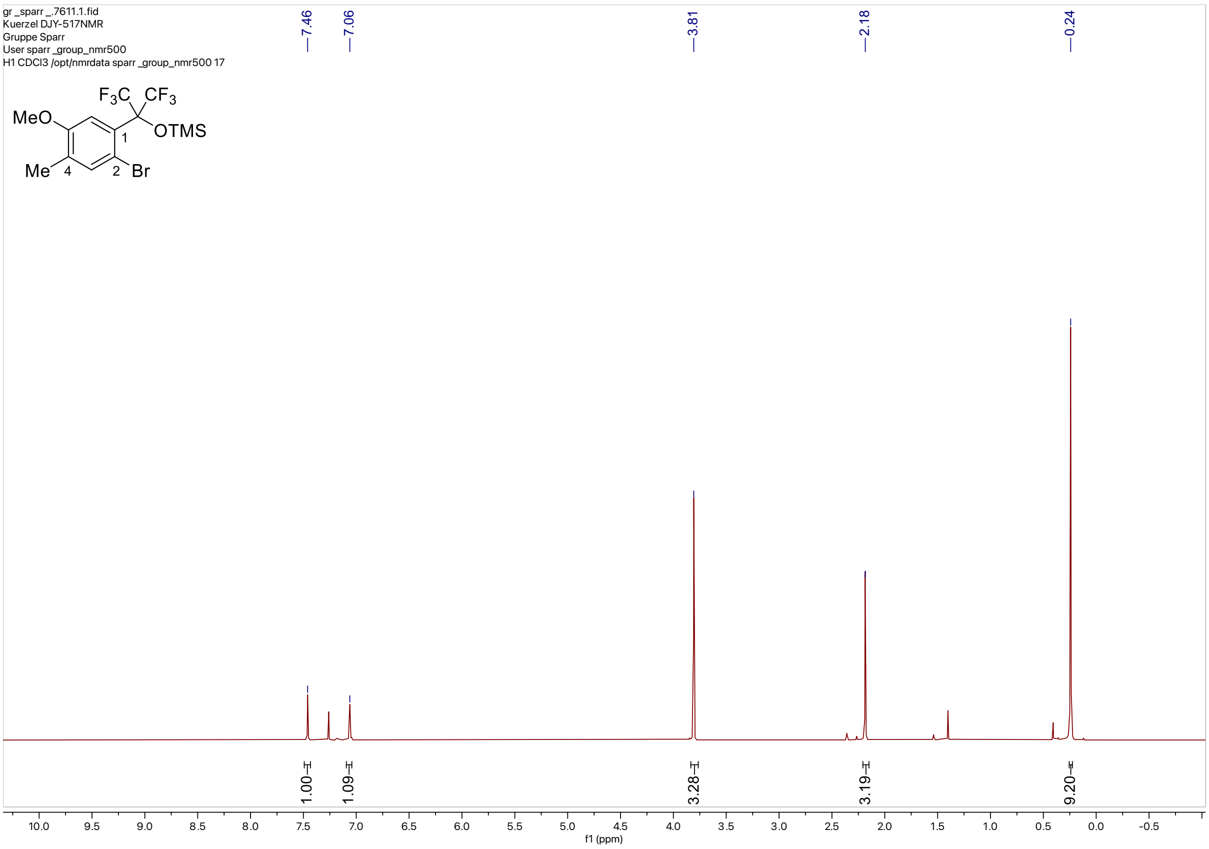

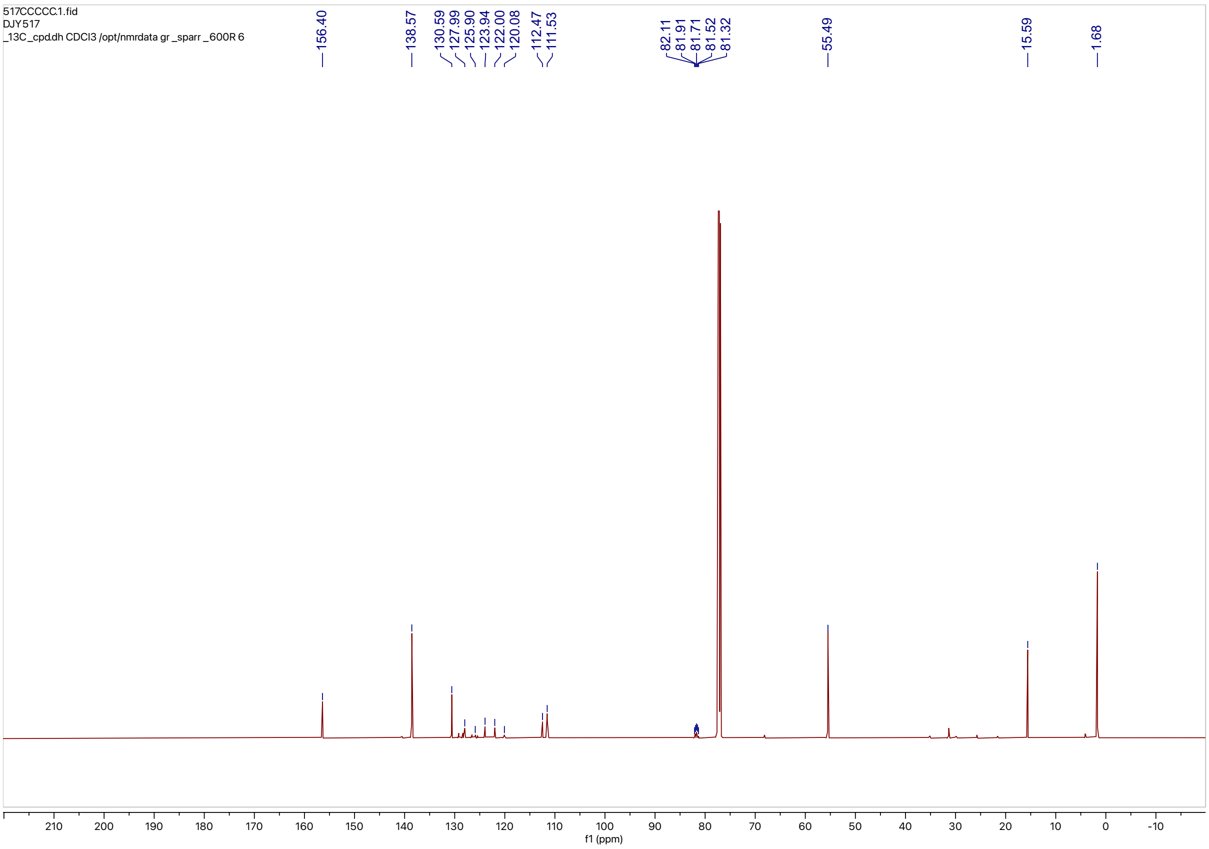

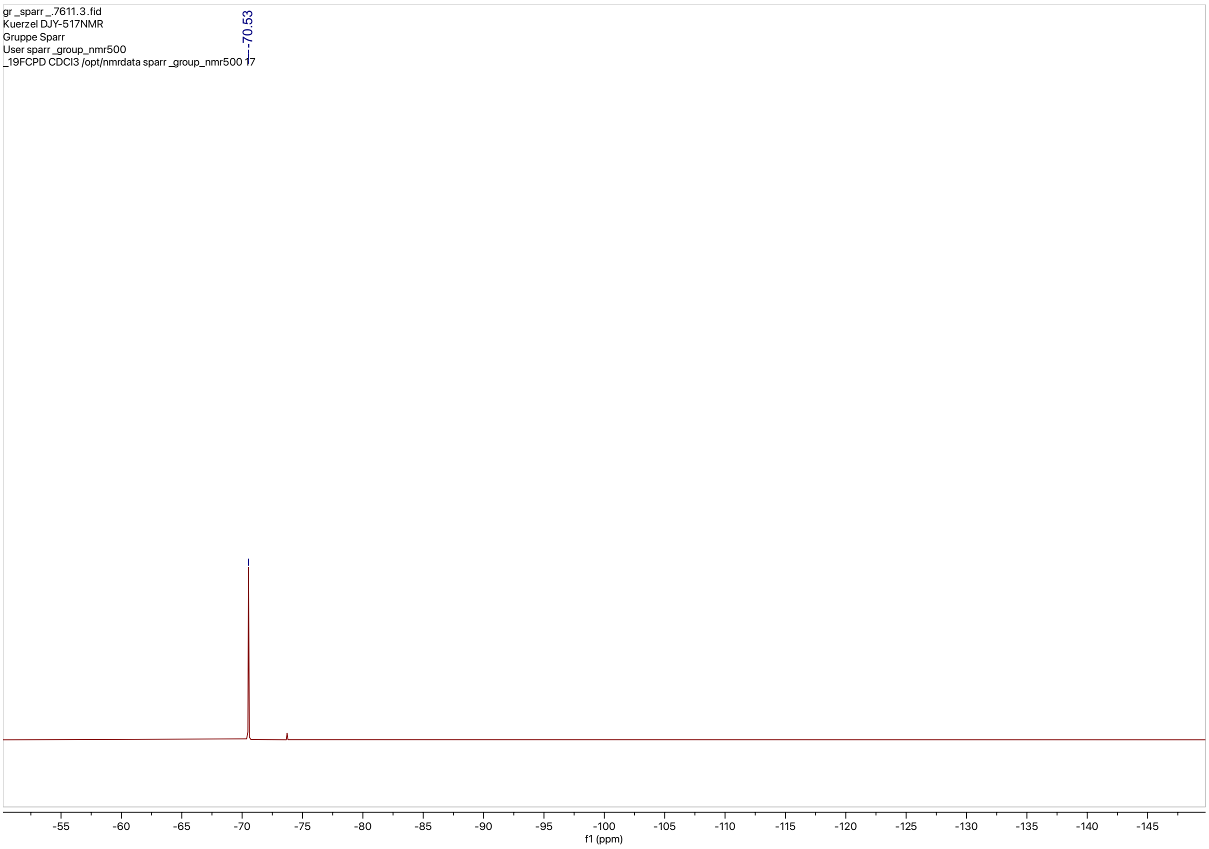

Supplementary Figure 40. ^{1}H (500 MHz, $CDCl_3$, 25 °C), $^{13}C\{^{1}H\}$ (151 MHz, $CDCl_3$, 25 °C) and $^{19}F\{^{1}H\}$ (470 MHz, $CDCl_3$, 25 °C) spectra of **S2e**

^{1}H, ^{13}C{^{1}H} and ^{19}F{^{1}H} spectra of 2-(2-Bromo-5-methoxy-4-methylphenyl)-1,1,1,3,3,3-hexafluoropropan-2-ol (S3e)

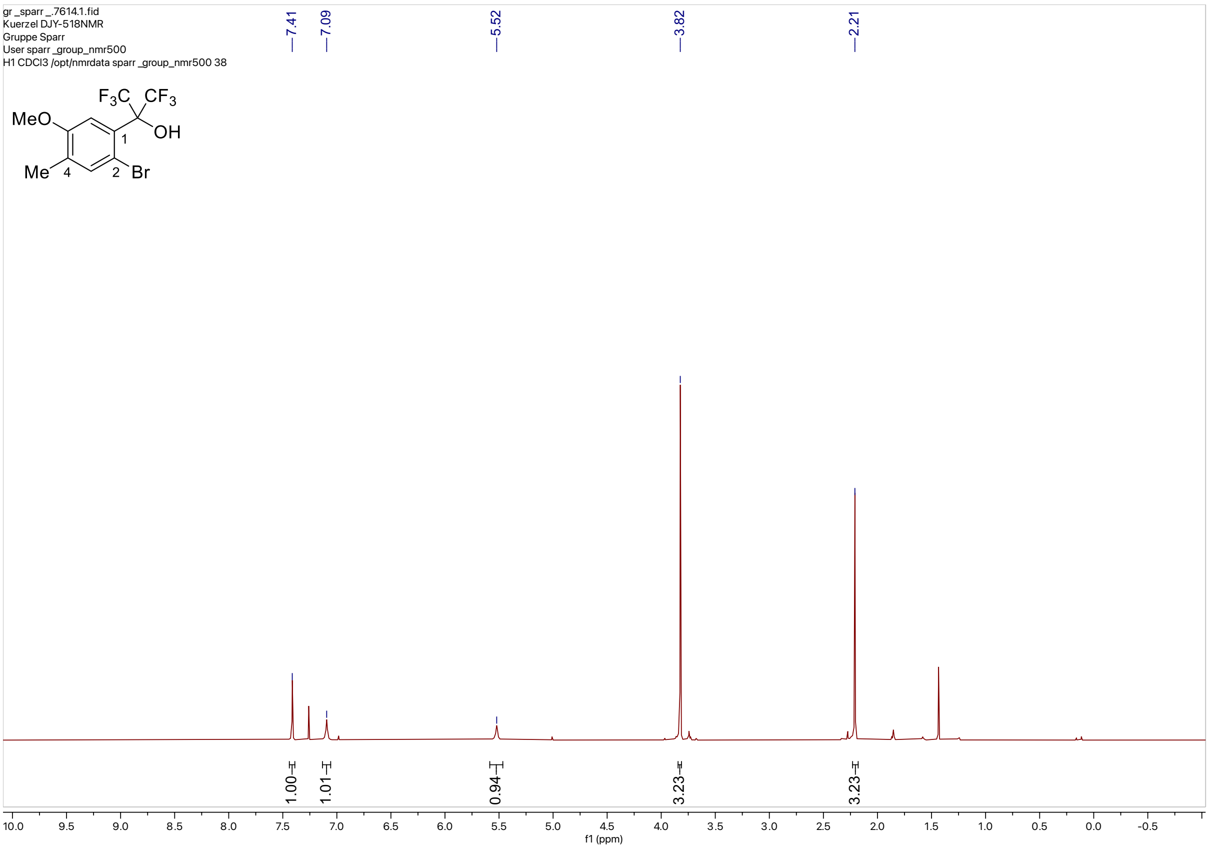

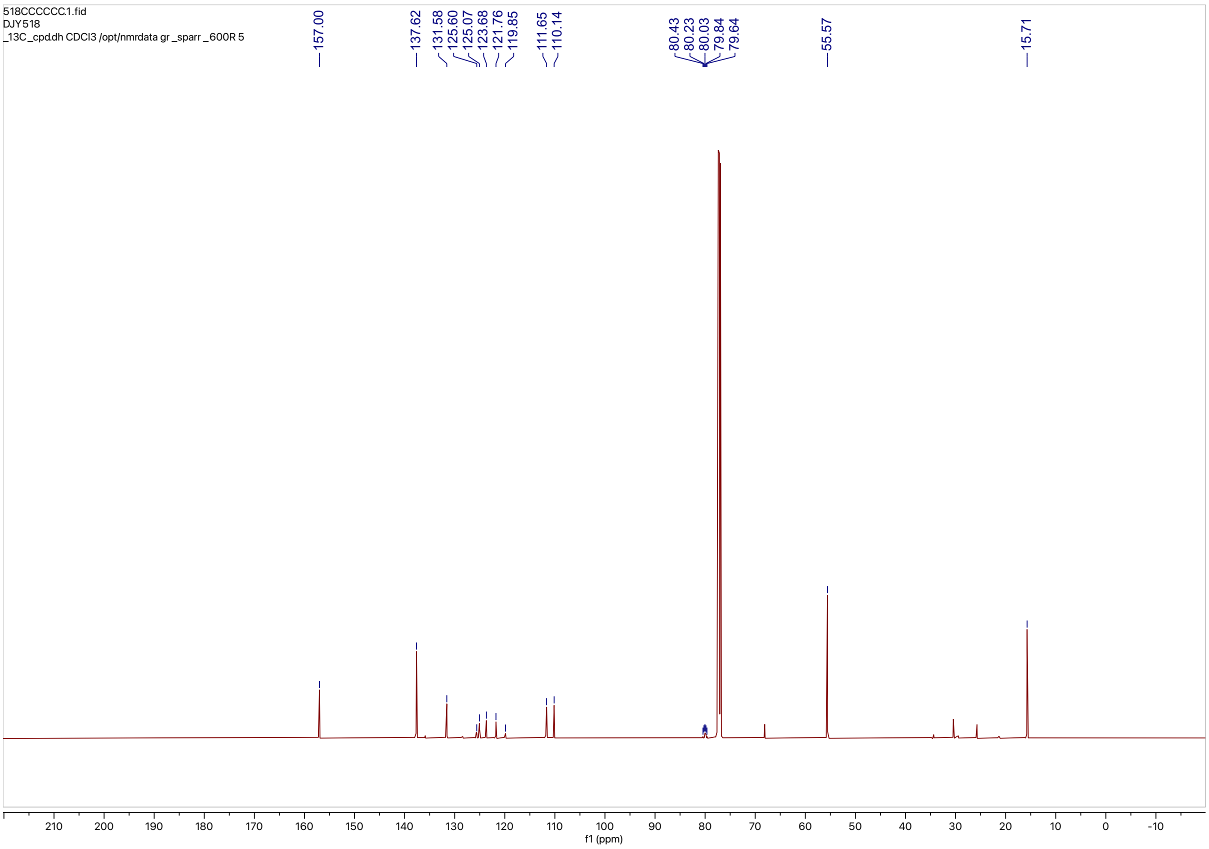

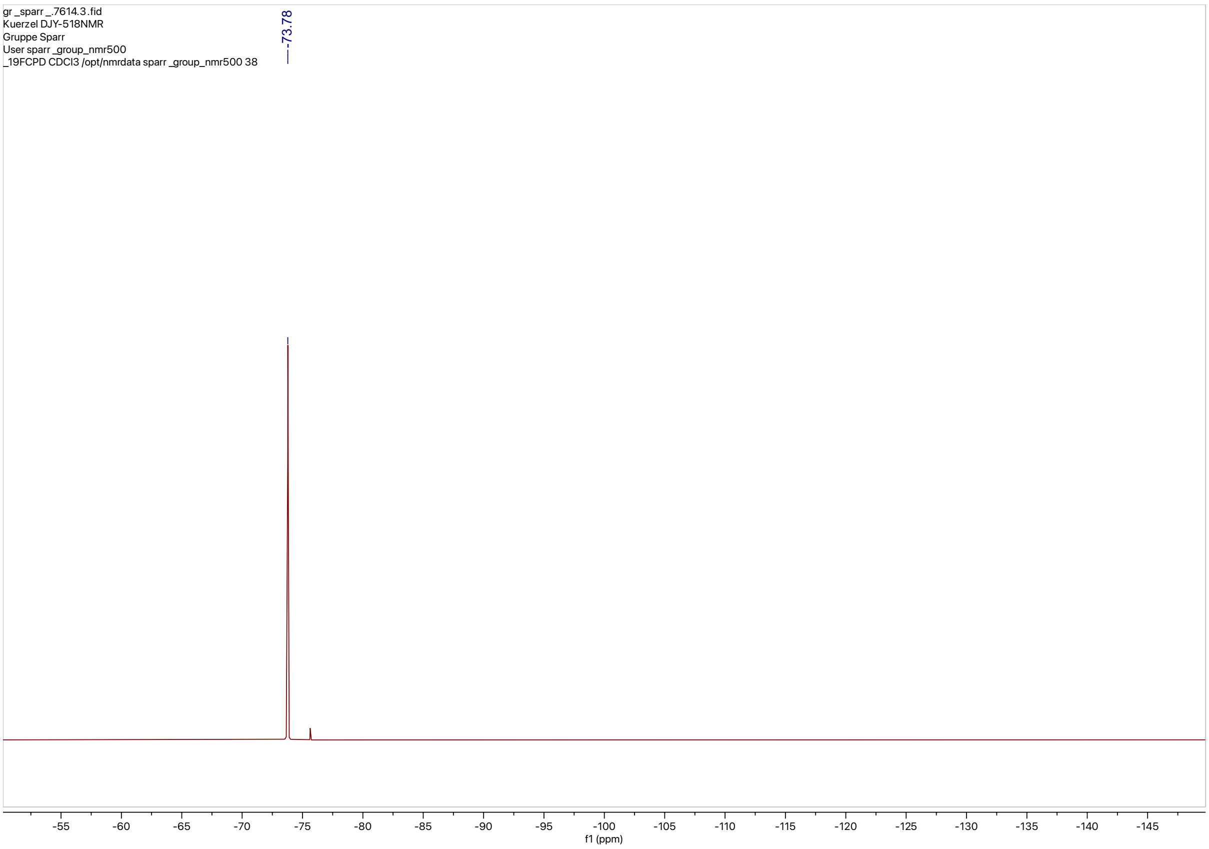

Supplementary Figure 41. ^{1}H (500 MHz, $CDCl_3$, 25 °C), $^{13}C\{^{1}H\}$ (151 MHz, $CDCl_3$, 25 °C) and $^{19}F\{^{1}H\}$ (470 MHz, $CDCl_3$, 25 °C) spectra of **S3e**

^{1}H, $^{13}C\{^{1}H\}$, $^{19}F\{^{1}H\}$ and $^{31}P\{^{1}H\}$ spectra of [*TBPY*-5-11']-5,5'-Dimethoxy-6,6'-dimethyl-1-hydro-3,3,3',3'-tetrakis(trifluoromethyl)-3*H*,3'*H*-1λ[5],1'-spirobi[ben-zo[*c*][2,1]oxaphosphole] (S4e)

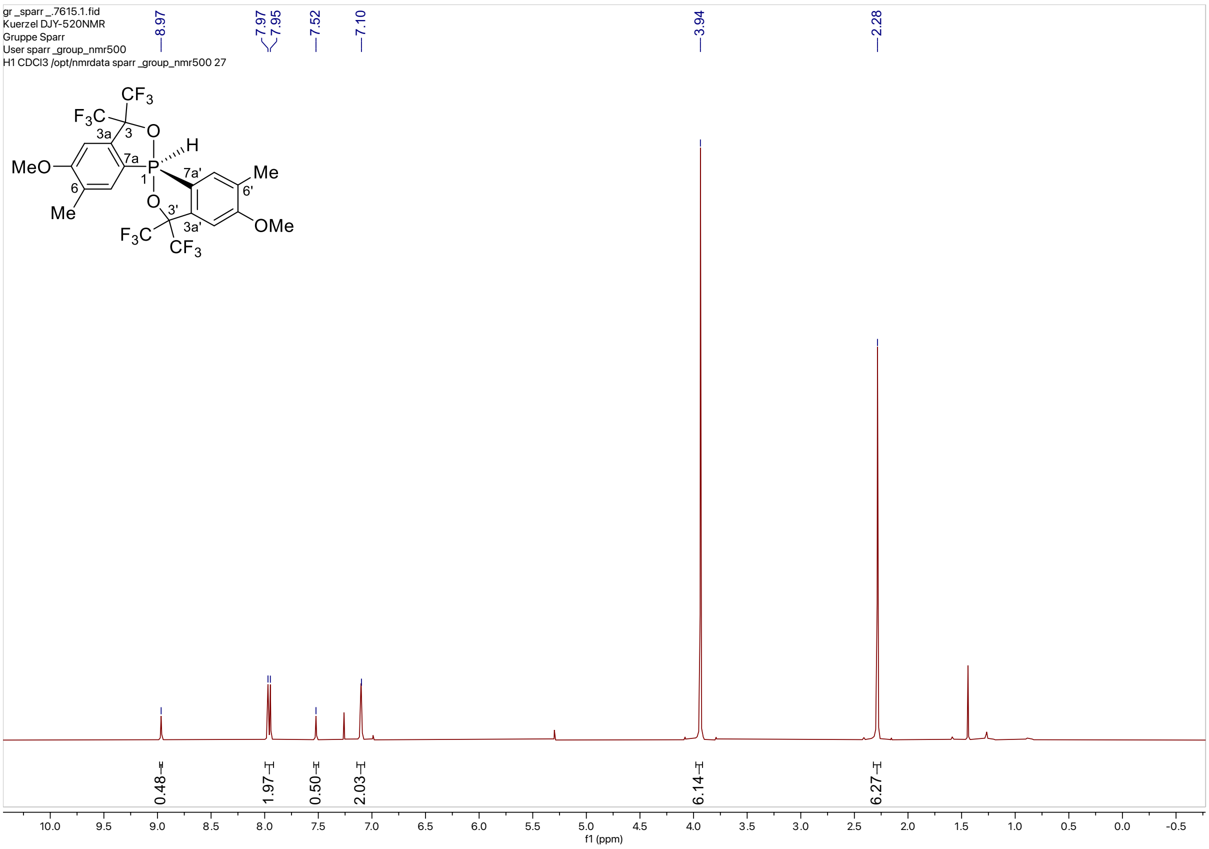

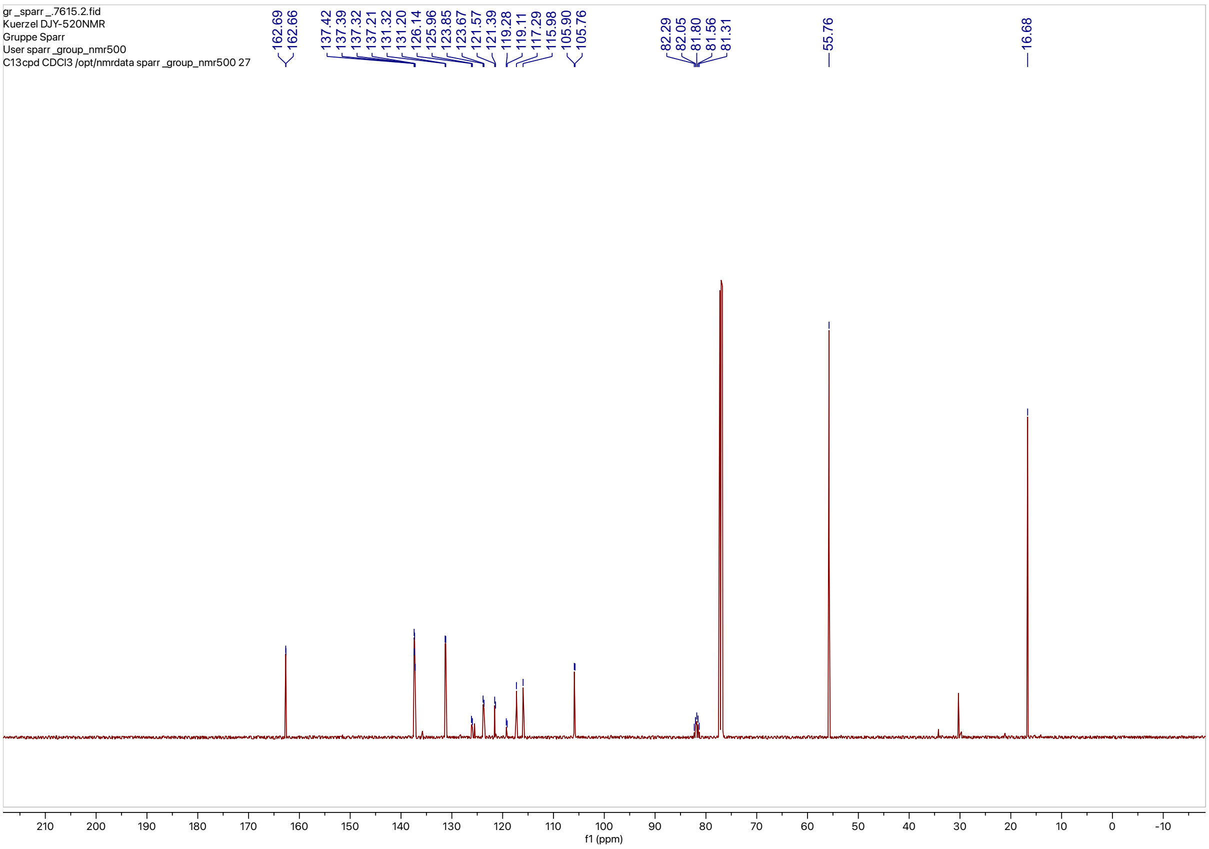

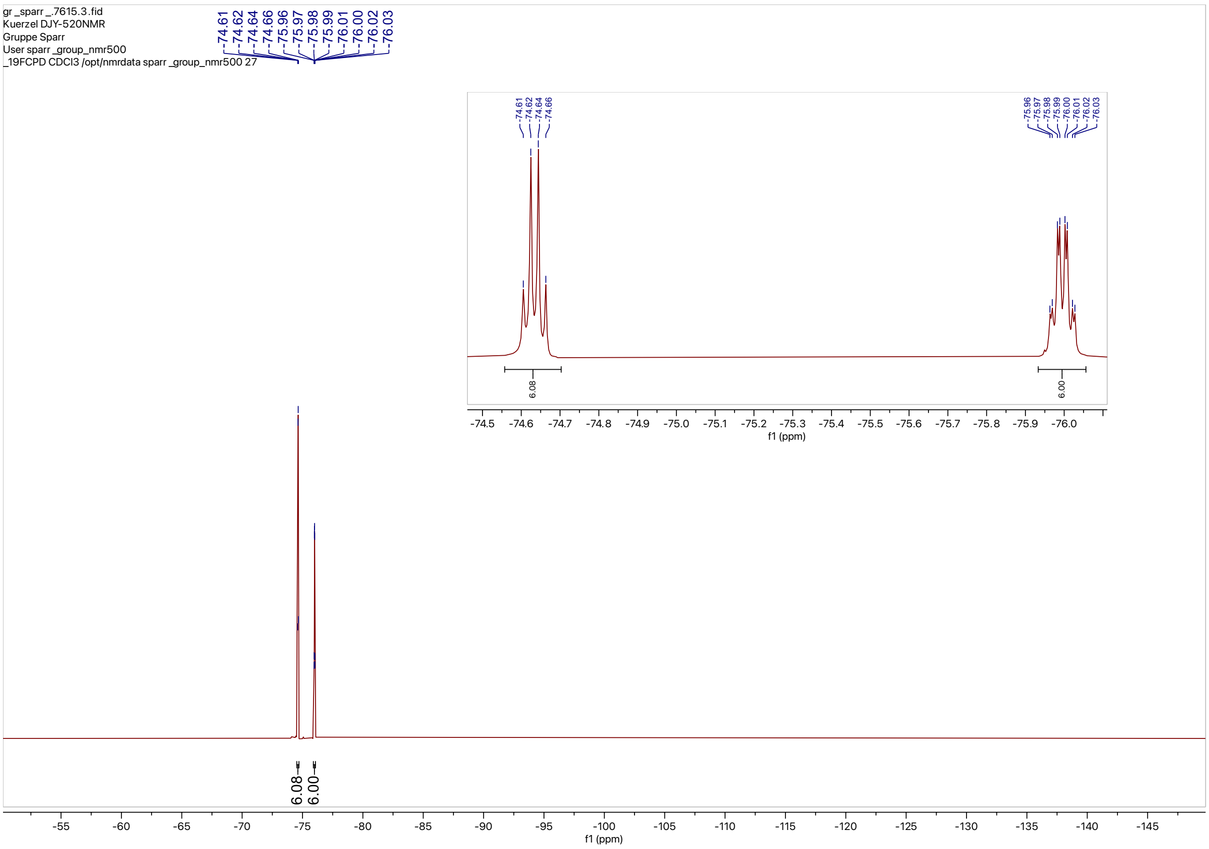

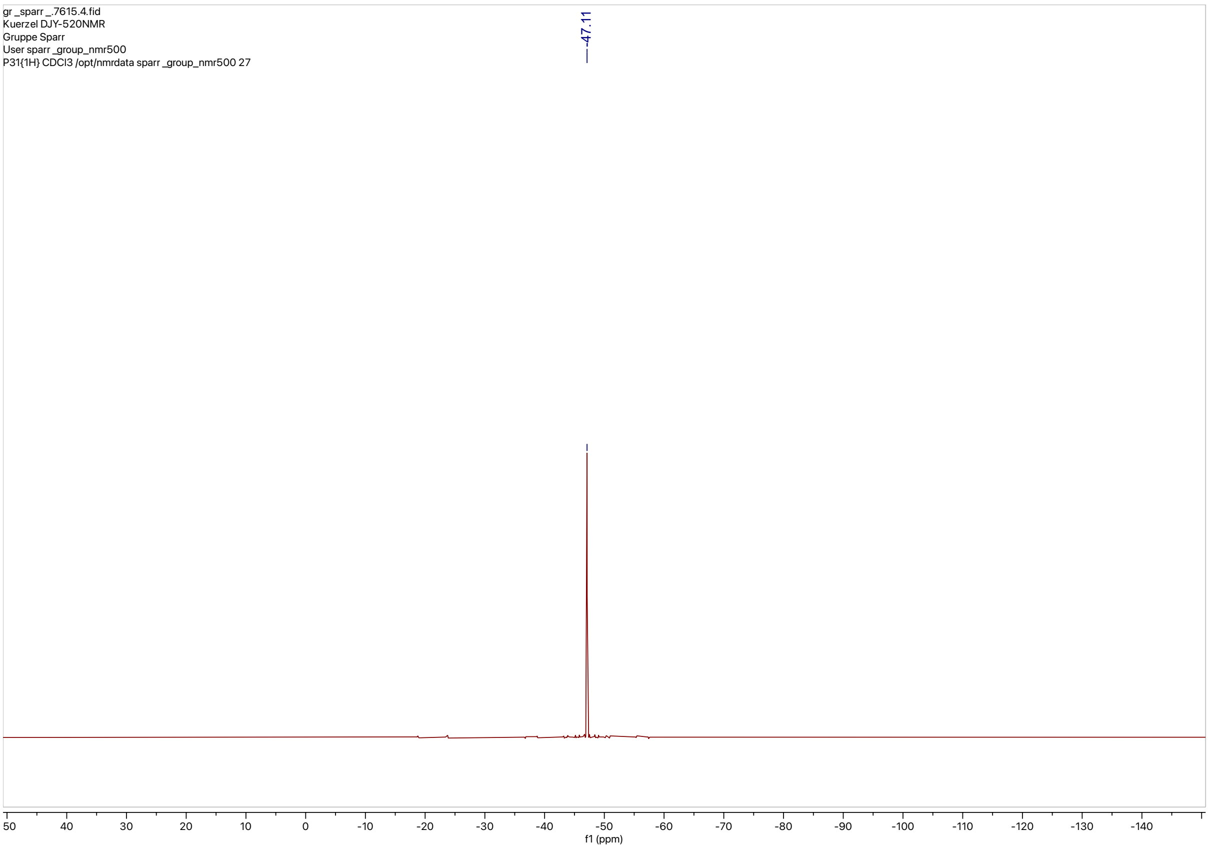

Supplementary Figure 42. ^{1}H (500 MHz, $CDCl_3$, 25 °C), ^{13}C{^{1}H} (126 MHz, $CDCl_3$, 25 °C), ^{19}F{^{1}H} (470 MHz, $CDCl_3$, 25 °C) and ^{31}P{^{1}H} (202 MHz, $CDCl_3$, 25 °C) spectra of **S4e**

^{1}H, ^{19}F{^{1}H} and ^{31}P{^{1}H} spectra of [*TBPY*-5-15]-1,1,1,3,3,3-Hexafluoro-2-(2-(1-butyl-1-hydro-5-methoxy-6-methyl-3,3-bis(trifluoromethyl)-3*H*-1λ^{5}-benzo[*c*][2,1]oxaphosphol-1-yl)-5-methoxy-4-methylphenyl)propan-2-ol (1e)

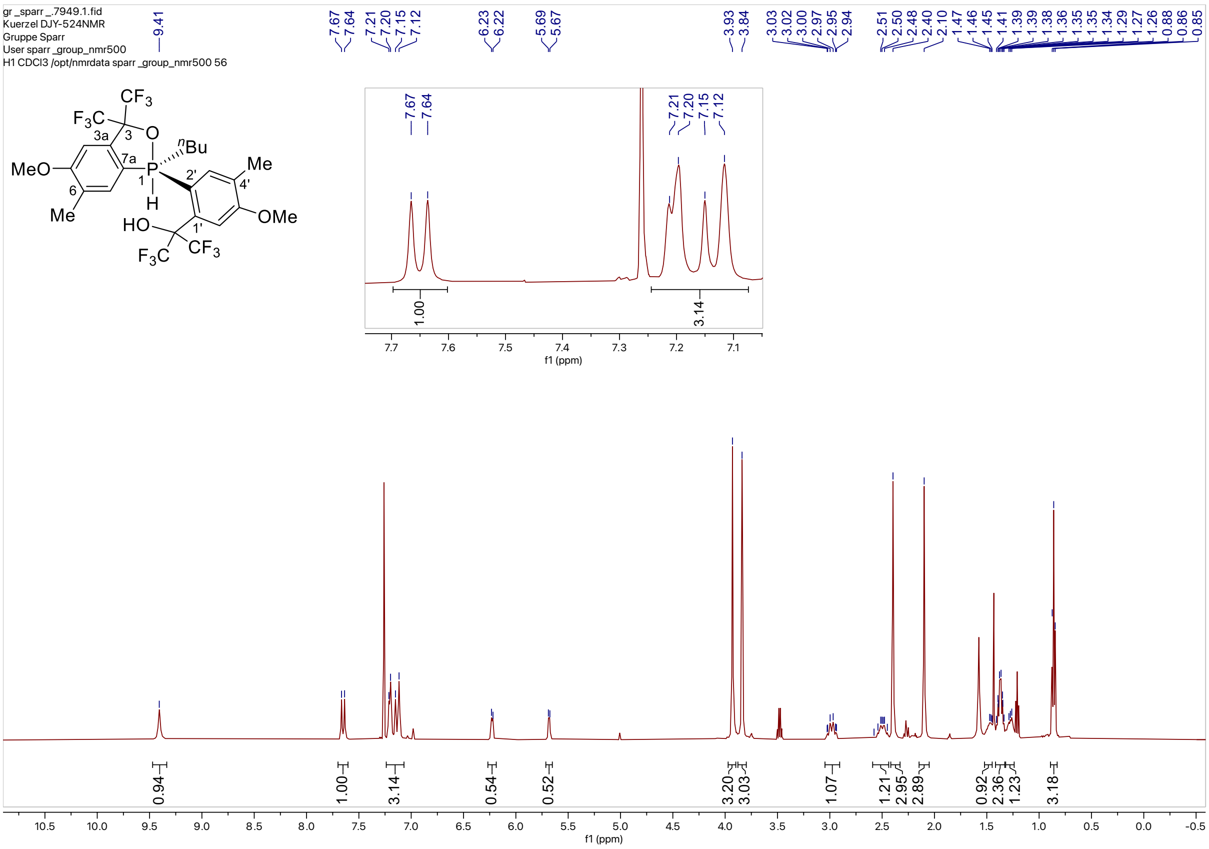

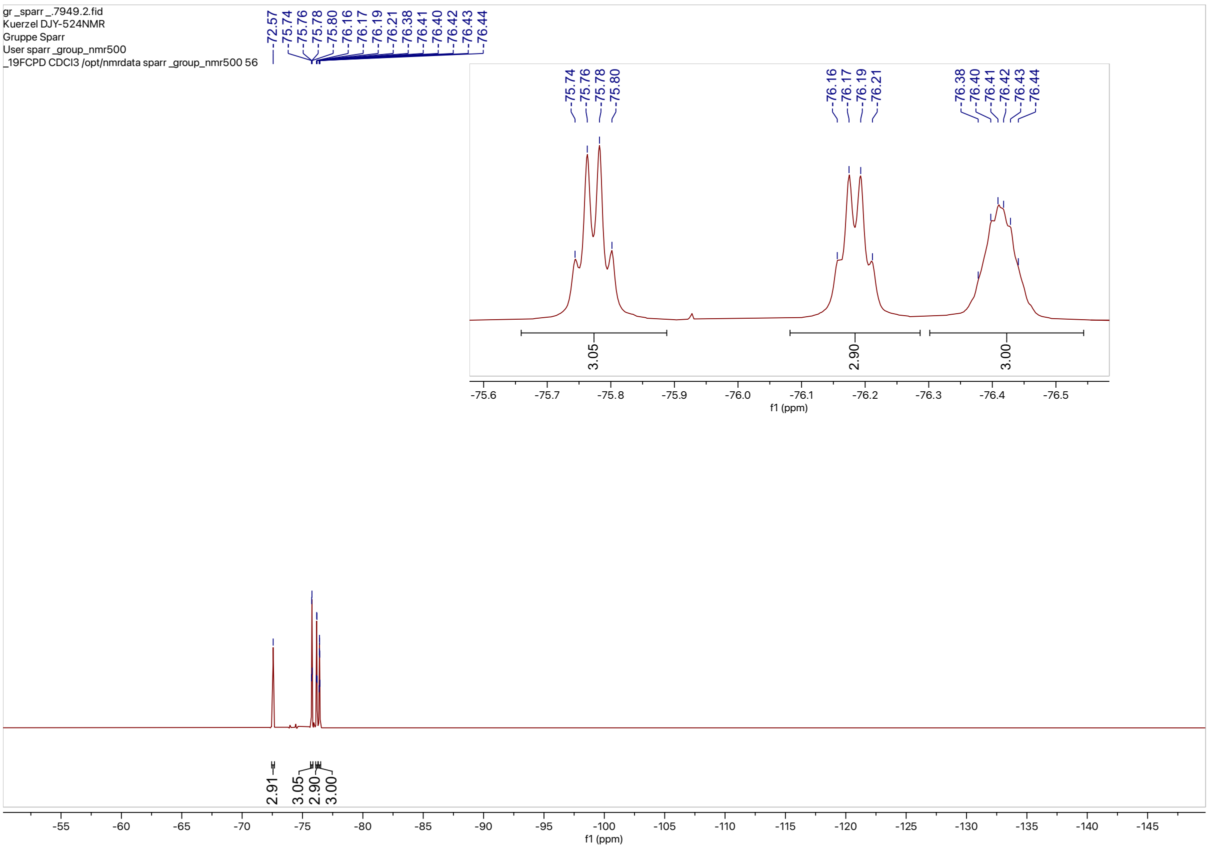

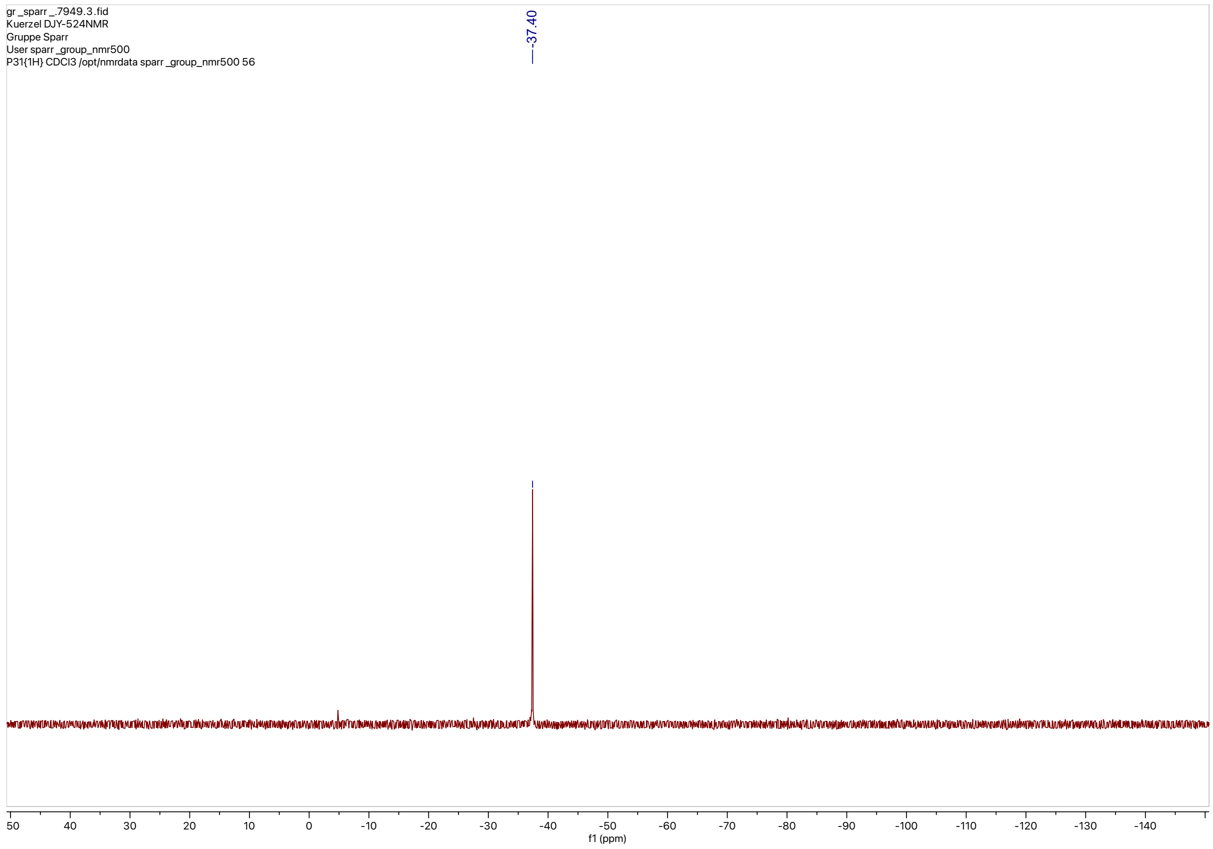

Supplementary Figure 43. ^{1}H (500 MHz, $CDCl_3$, 25 °C), ^{19}F{^{1}H} (470 MHz, $CDCl_3$, 25 °C) and ^{31}P{^{1}H} (202 MHz, $CDCl_3$, 25 °C) spectra of **1e**

^{1}H, ^{13}C{^{1}H} and ^{19}F{^{1}H} spectra of 2,3,4,5,6-Pentafluorophenyl 3-bromo-2-naphthoate (S1f)

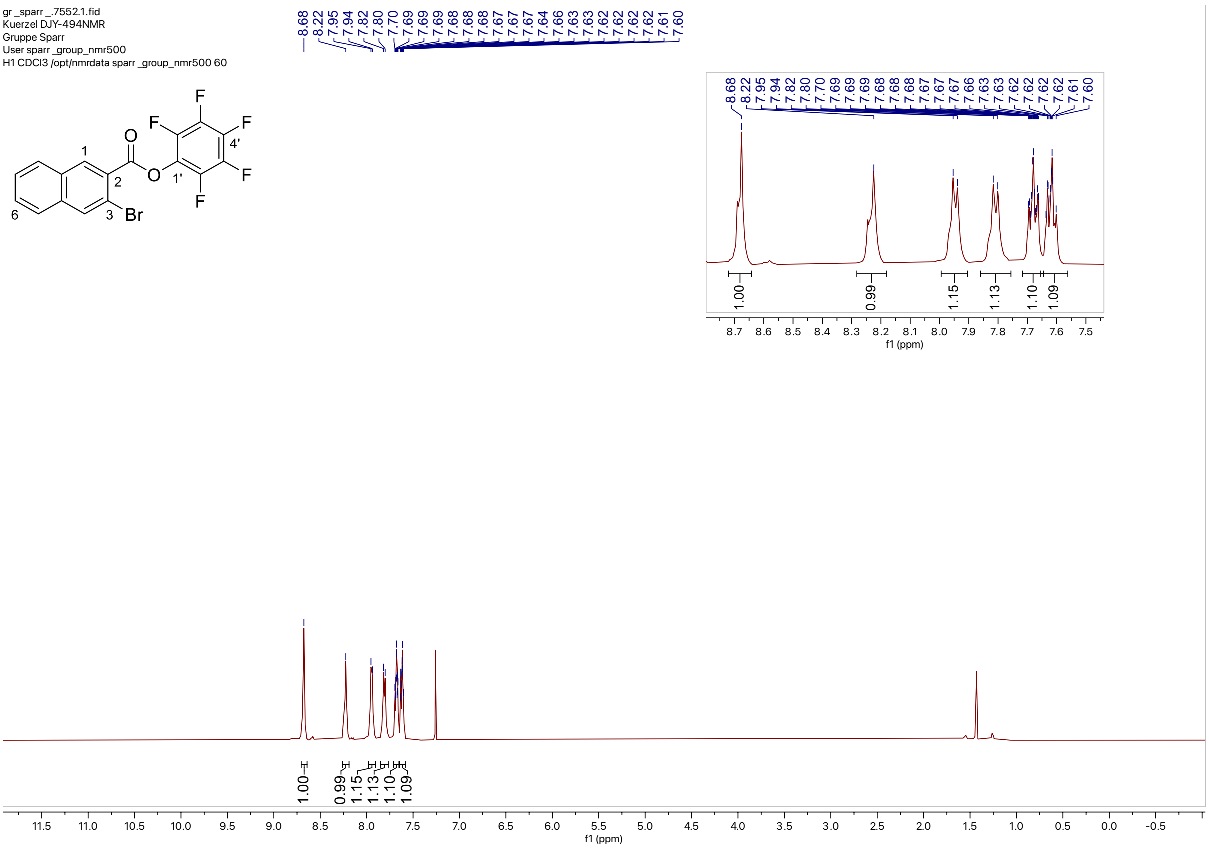

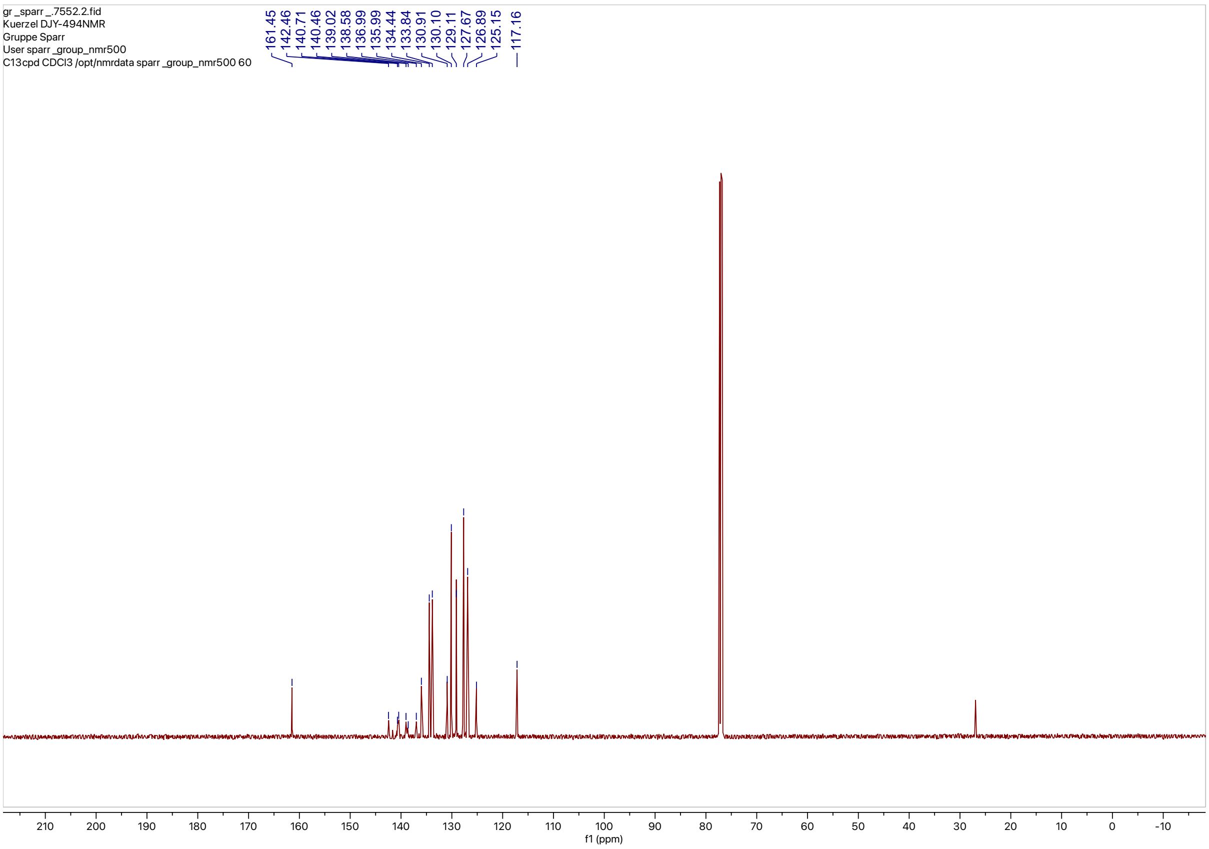

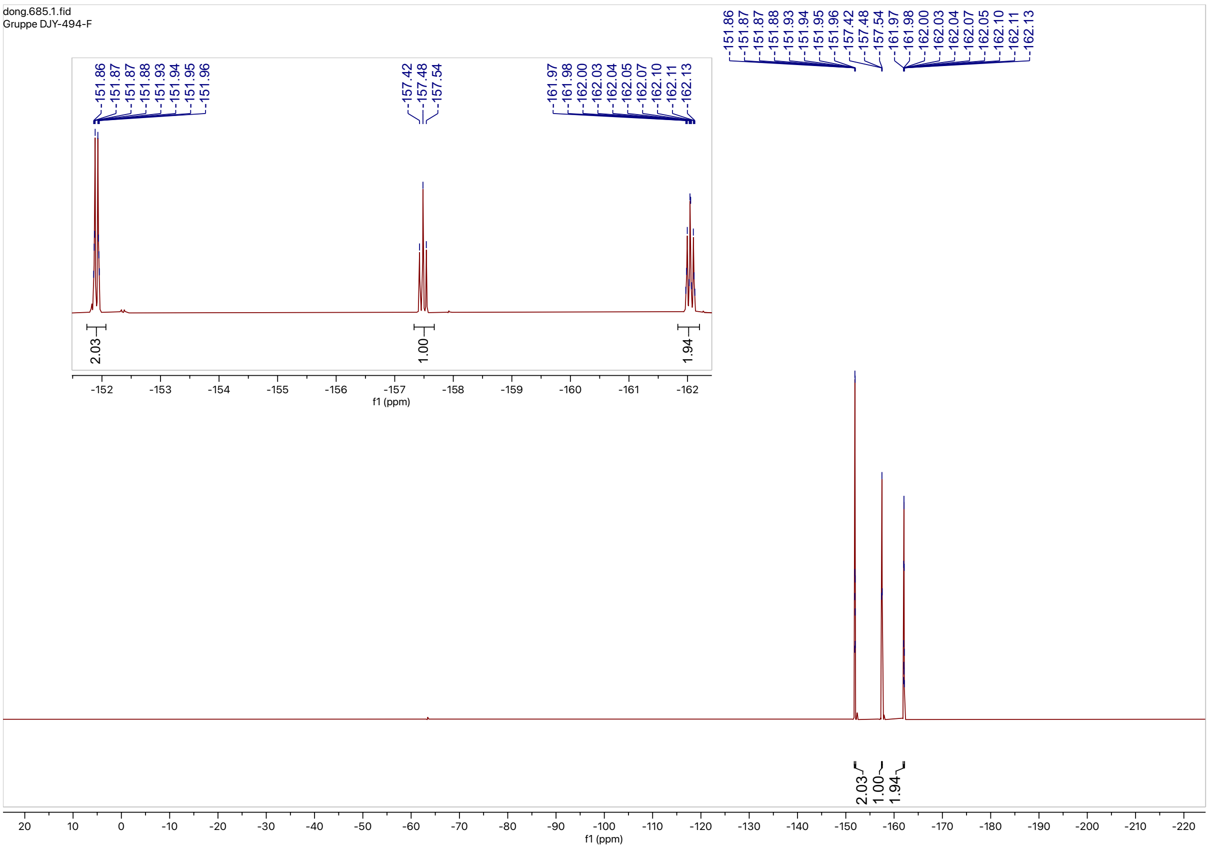

Supplementary Figure 44. ^{1}H (500 MHz, $CDCl_3$, 25 °C), $^{13}C\{^1H\}$ (126 MHz, $CDCl_3$, 25 °C) and $^{19}F\{^1H\}$ (376 MHz, $CDCl_3$, 25 °C) spectra of **S1f**

^{1}H, ^{13}C{^{1}H} and ^{19}F{^{1}H} spectra of ((2-(3-Bromonaphthalen-2-yl)-1,1,1,3,3,3-hexafluoropropan-2-yl)oxy)trimethylsilane (S2f)

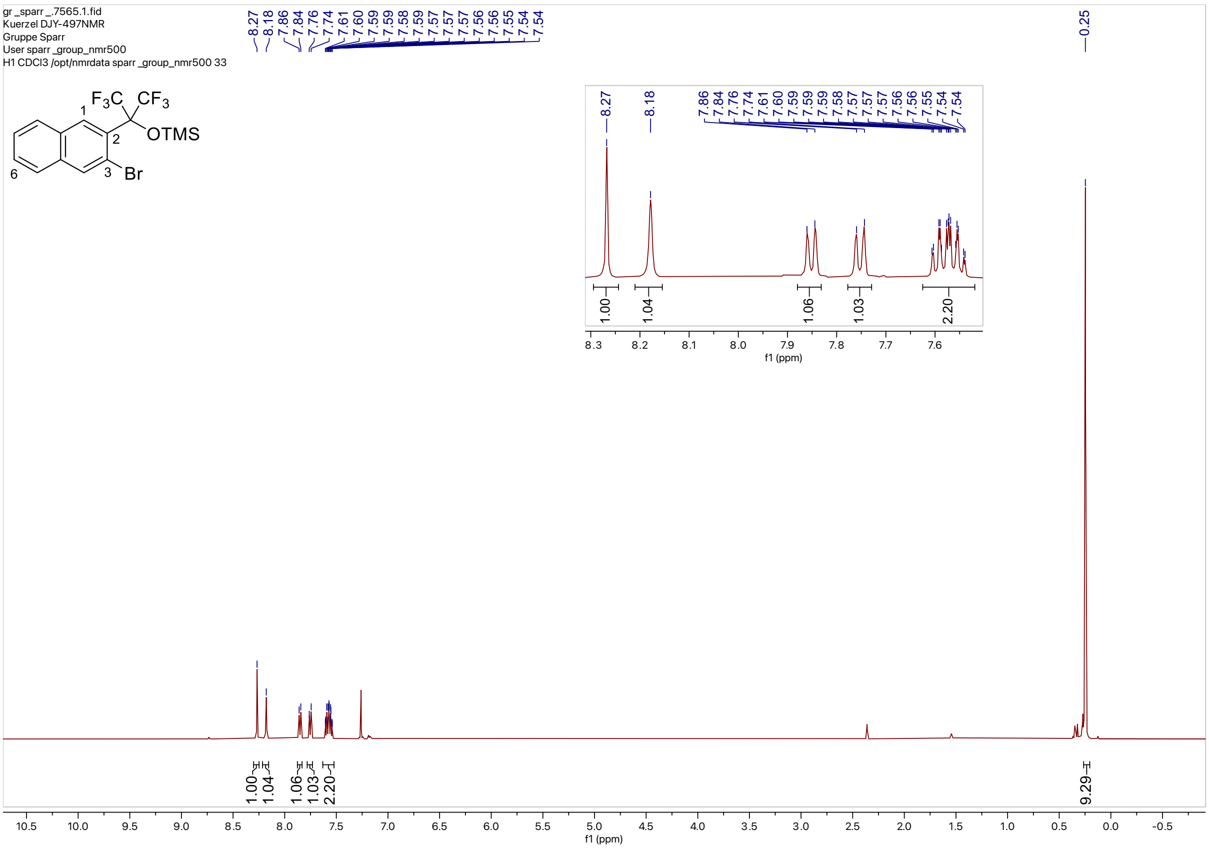

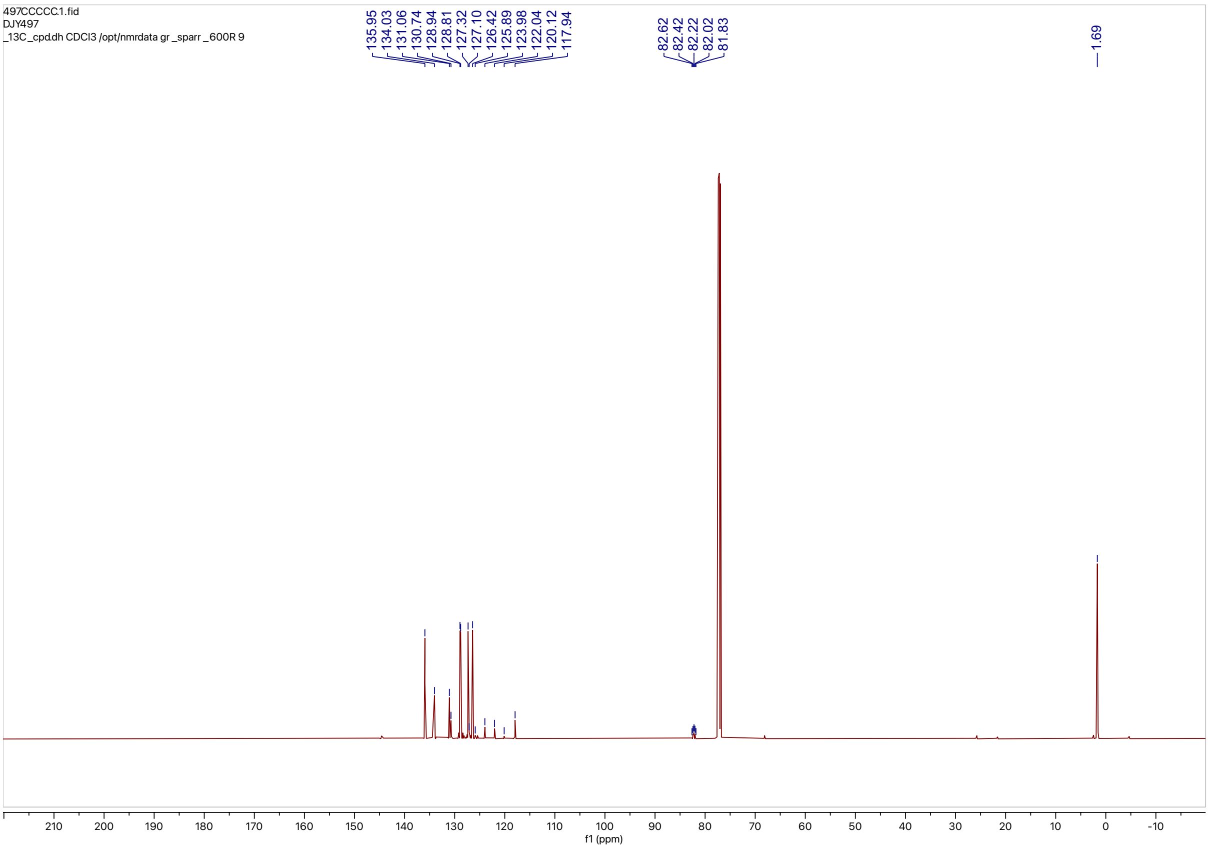

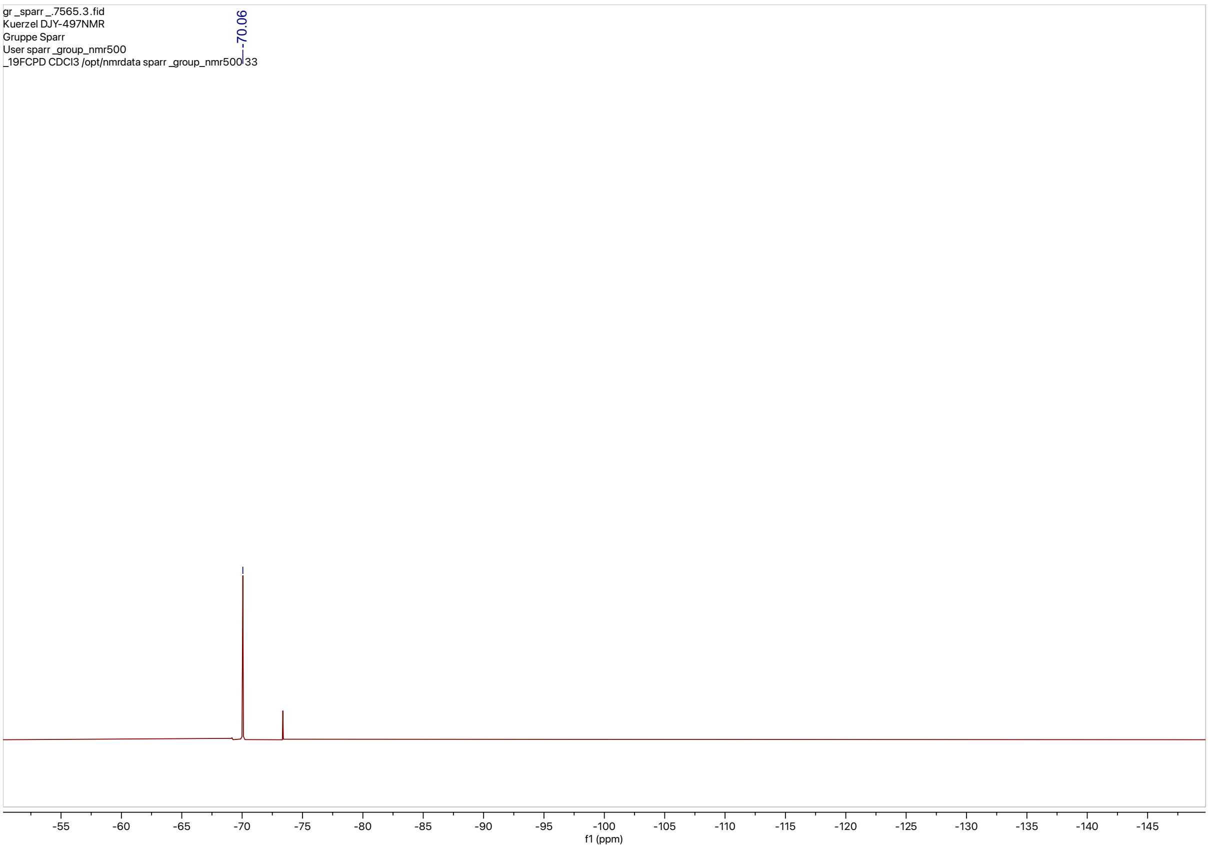

Supplementary Figure 45. ^{1}H (500 MHz, $CDCl_3$, 25 °C), $^{13}C\{^{1}H\}$ (151 MHz, $CDCl_3$, 25 °C) and $^{19}F\{^{1}H\}$ (470 MHz, $CDCl_3$, 25 °C) spectra of **S2f**

^{1}H, $^{13}C\{^{1}H\}$ and $^{19}F\{^{1}H\}$ spectra of 2-(3-Bromonaphthalen-2-yl)-1,1,1,3,3,3-hexafluoropropan-2-ol (S3f)

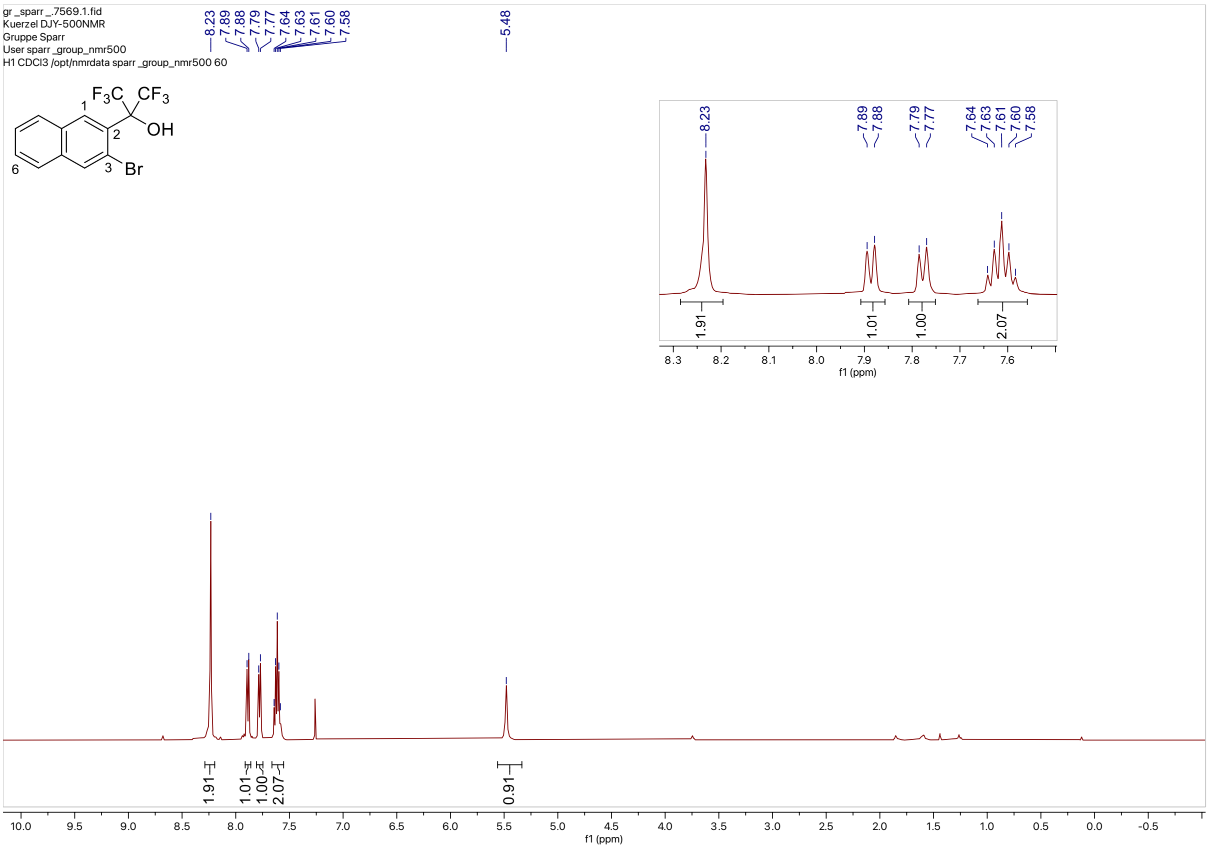

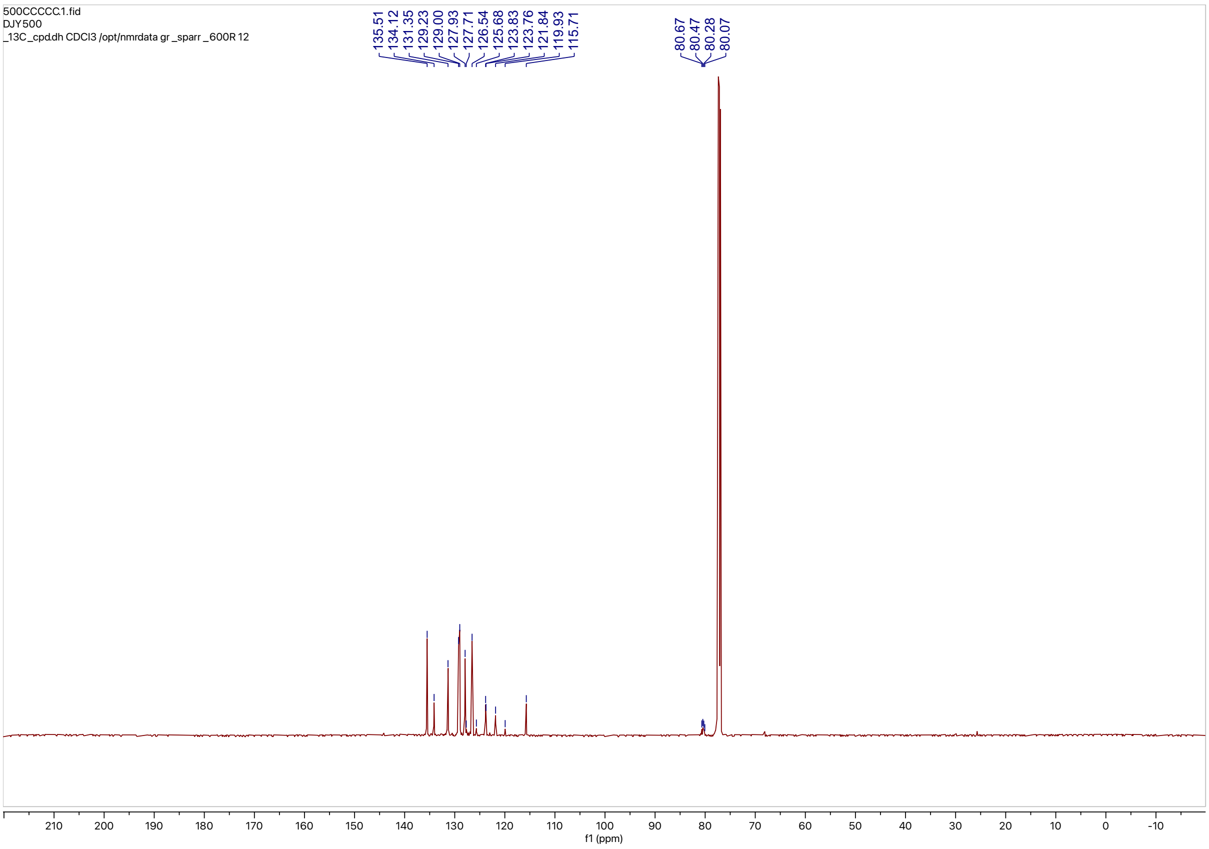

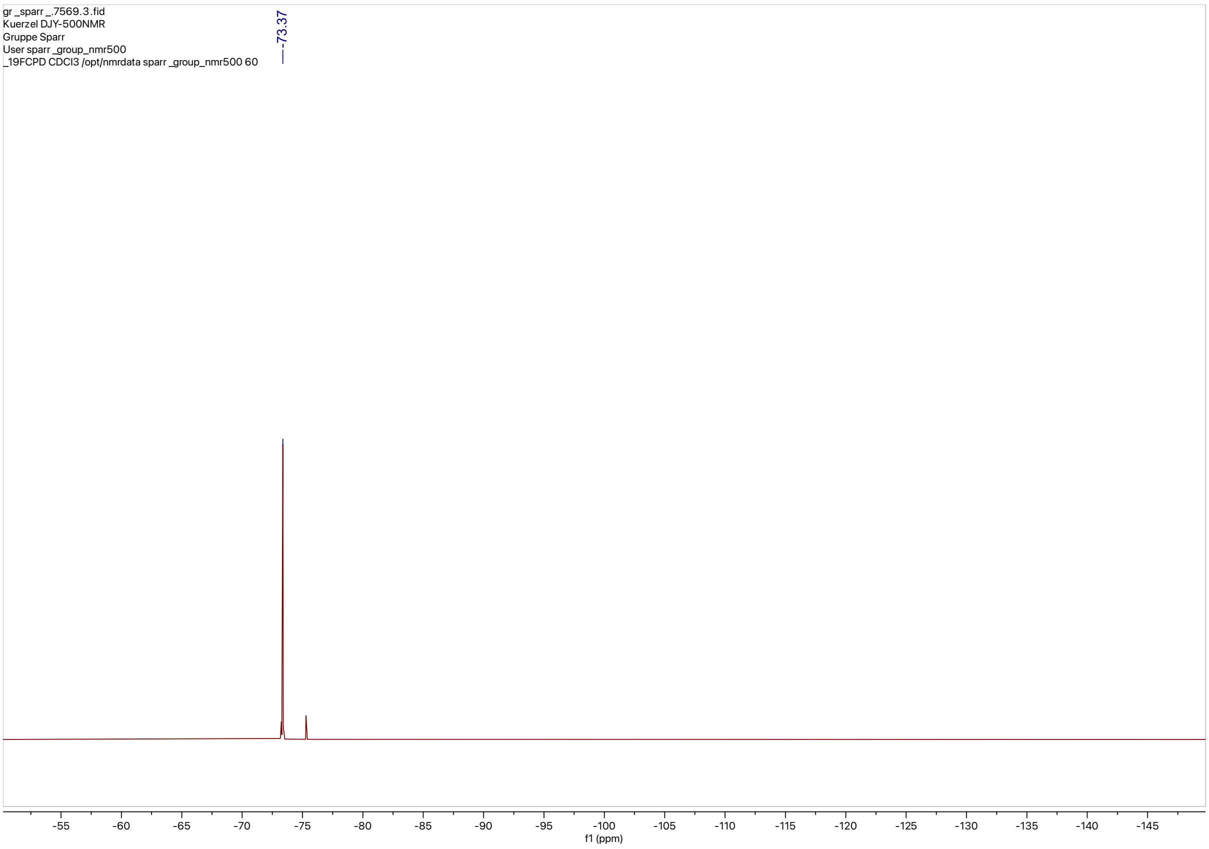

Supplementary Figure 46. ^{1}H (500 MHz, $CDCl_3$, 25 °C), $^{13}C\{^{1}H\}$ (151 MHz, $CDCl_3$, 25 °C) and $^{19}F\{^{1}H\}$ (470 MHz, $CDCl_3$, 25 °C) spectra of **S3f**

[1]H, [13]C{[1]H}, [19]F{[1]H} and [31]P{[1]H} spectra of [*TBPY*-5-11']-1-Hydro-3,3,3',3'-tetrakis(trifluoromethyl)-3*H*,3'*H*-1λ[5],1'-spirobi[naphtho[2,3-*c*][2,1]oxa-phosphole] (S4f)

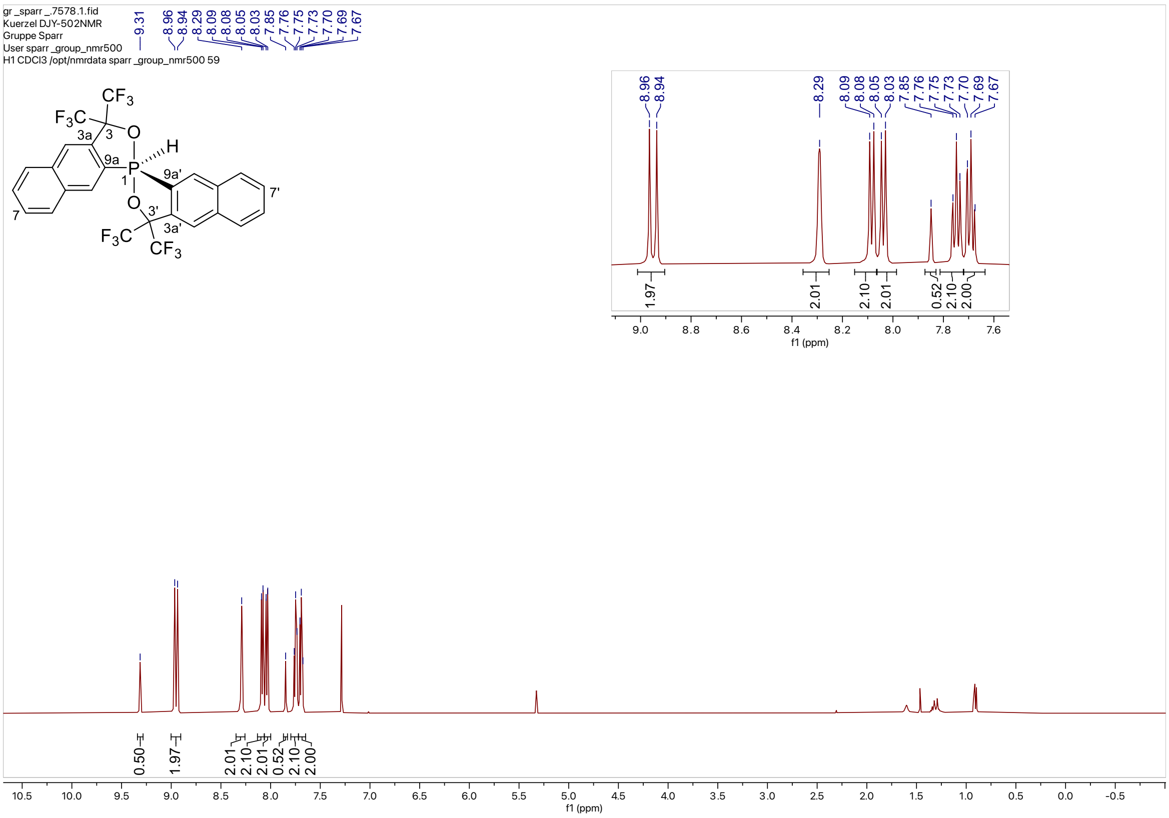

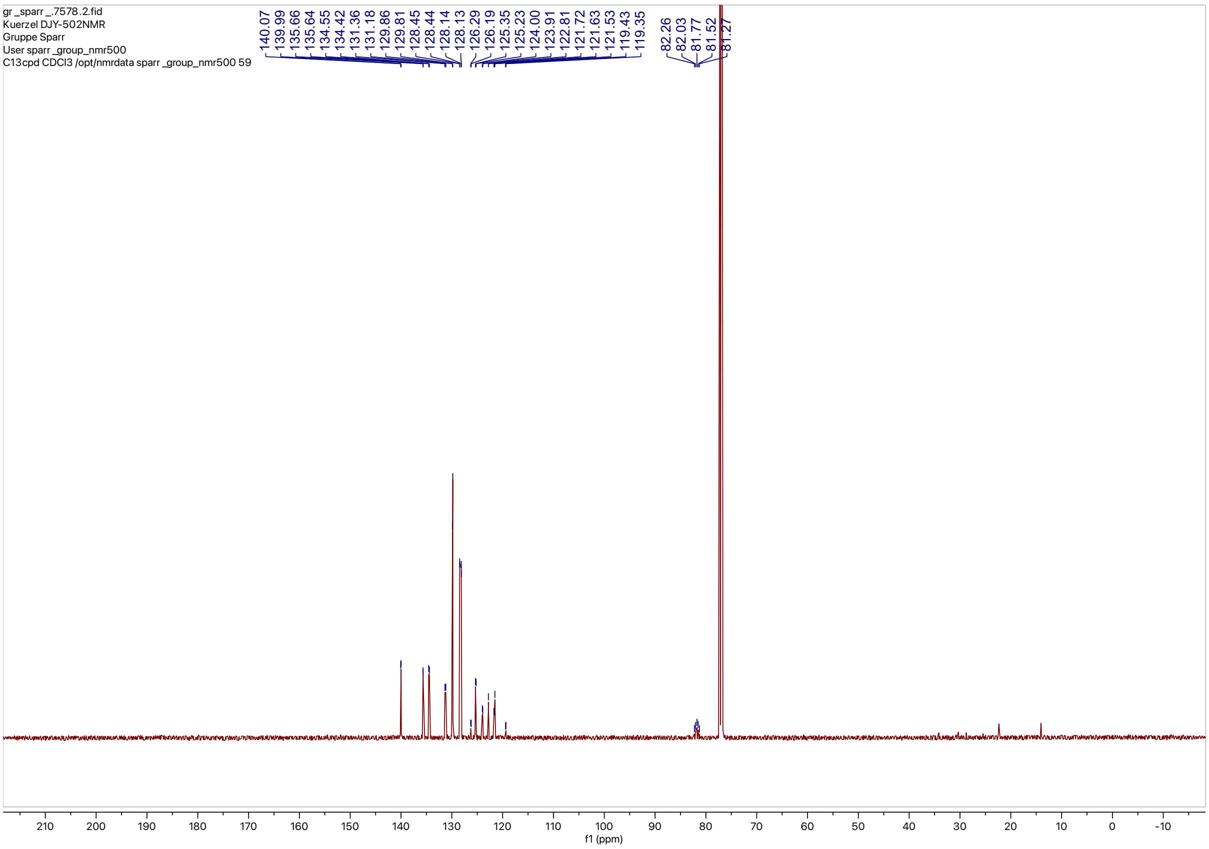

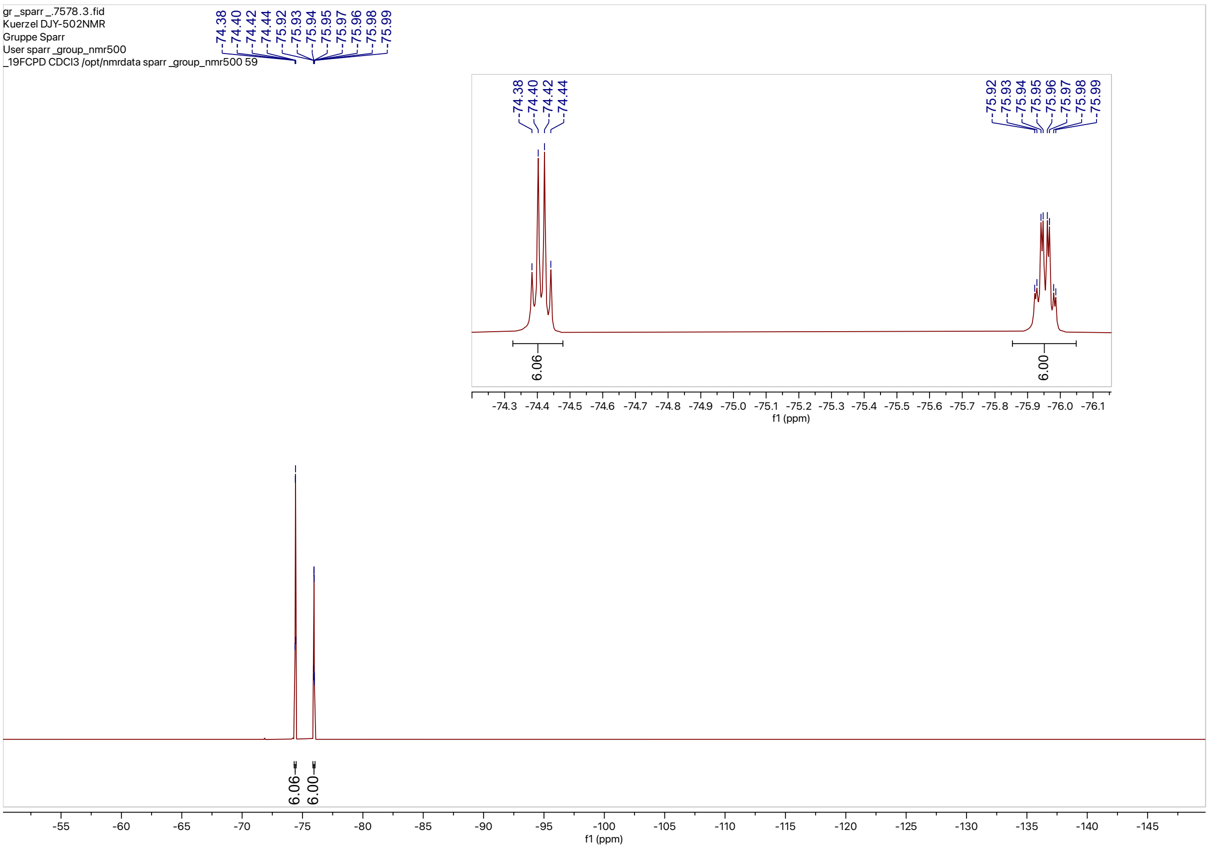

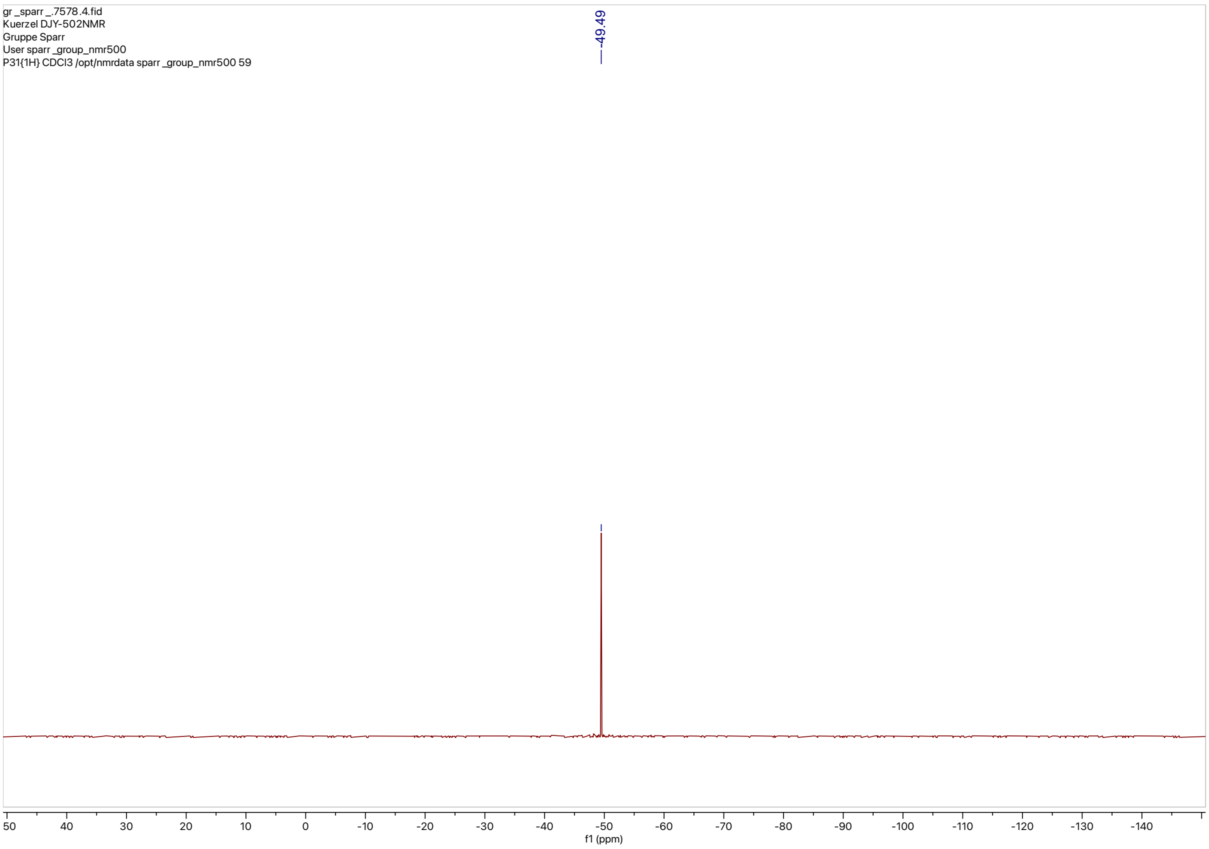

Supplementary Figure 47. ^{1}H (500 MHz, $CDCl_3$, 25 °C), $^{13}C\{^{1}H\}$ (126 MHz, $CDCl_3$, 25 °C), $^{19}F\{^{1}H\}$ (470 MHz, $CDCl_3$, 25 °C) and $^{31}P\{^{1}H\}$ (202 MHz, $CDCl_3$, 25 °C) spectra of **S4f**

^{1}H, $^{19}F\{^{1}H\}$ and $^{31}P\{^{1}H\}$ spectra of [*TBPY*-5-15]-1,1,1,3,3,3-Hexafluoro-2-(3-(1-butyl-1-hydro-3,3-bis(trifluoromethyl)-3*H*-1λ^{5}-naphtho[2,3-*c*][2,1]oxaphosphol-1-yl)-naphthalen-2-yl)propan-2-ol (1f)

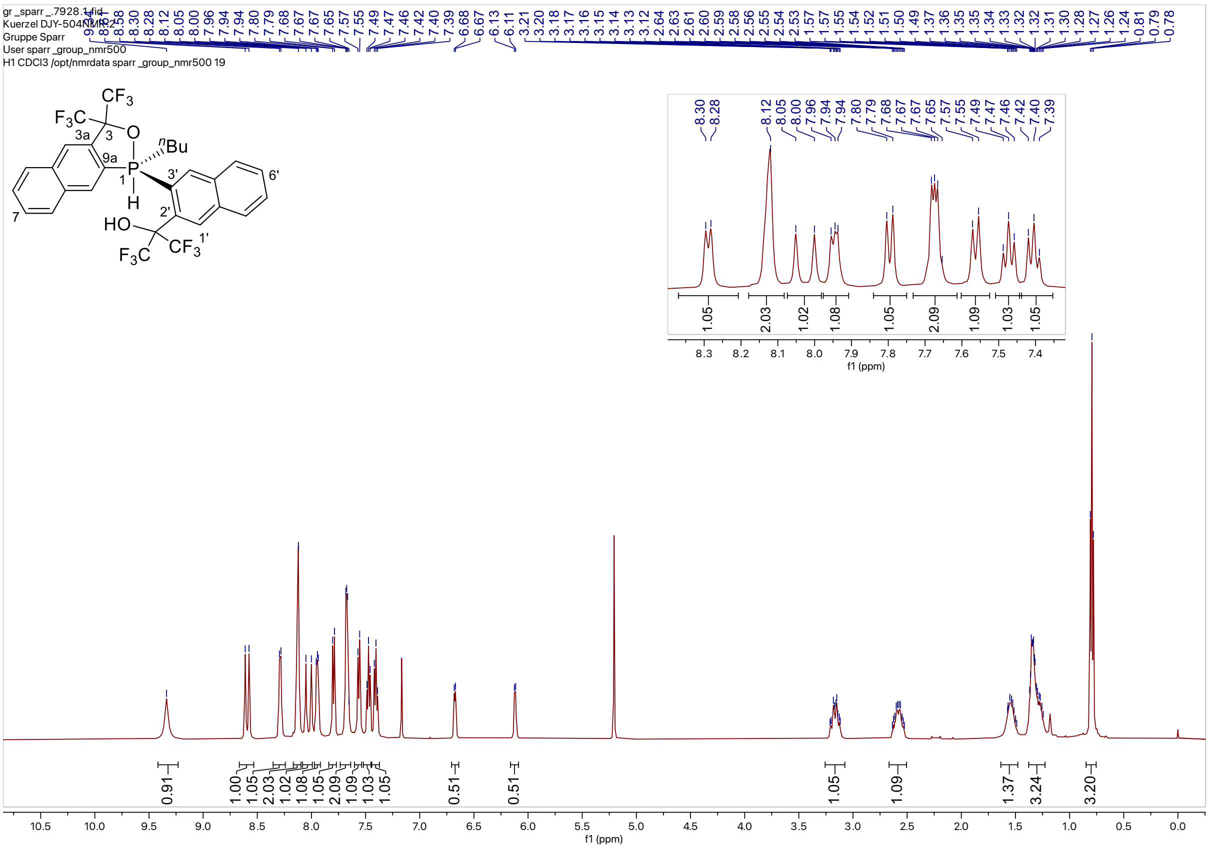

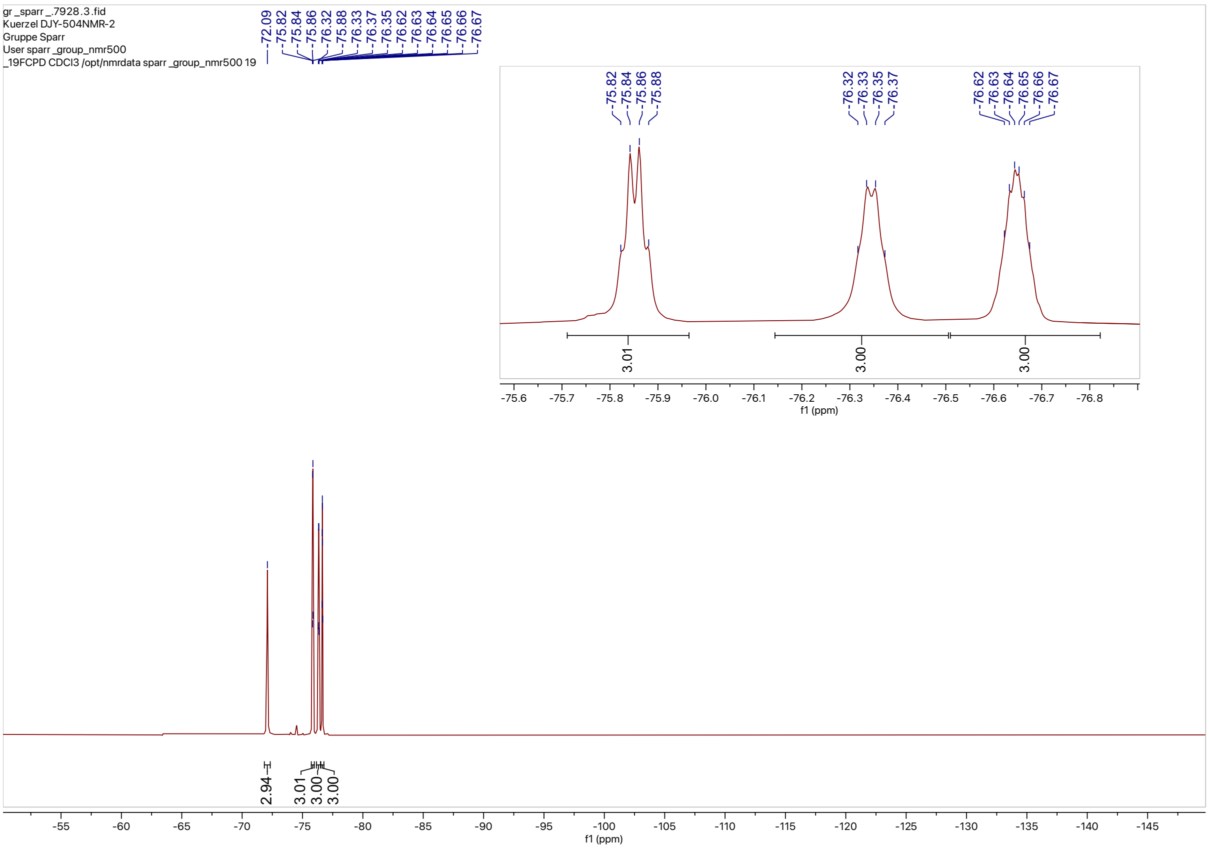

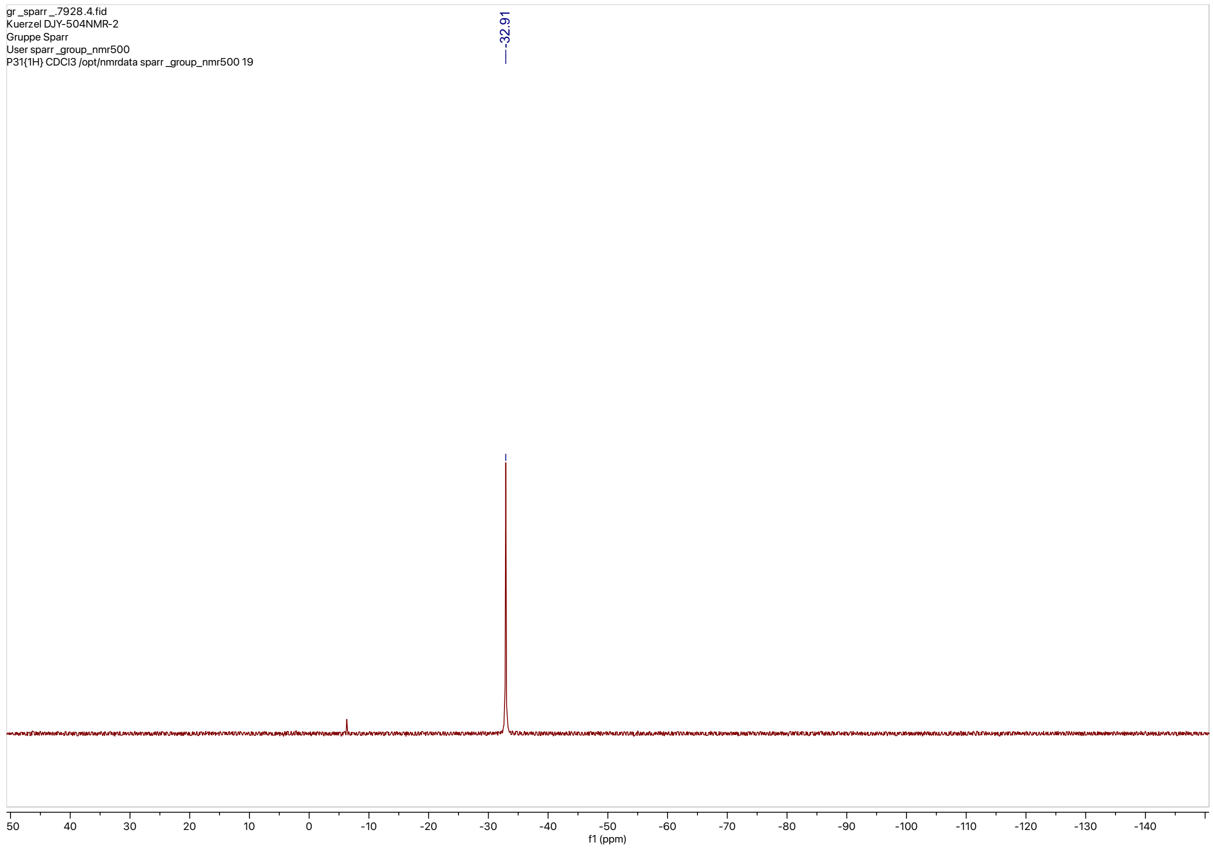

Supplementary Figure 48. ^{1}H (500 MHz, $CDCl_3$, 25 °C), $^{19}F\{^{1}H\}$ (470 MHz, $CDCl_3$, 25 °C) and $^{31}P\{^{1}H\}$ (202 MHz, $CDCl_3$, 25 °C) spectra of **1f**

[1]H, [13]C{[1]H} and [19]F{[1]H} spectra of 2,3,4,5,6-Pentafluorophenyl 3-bromo-6-methoxy-2-naphthoate (S1g)

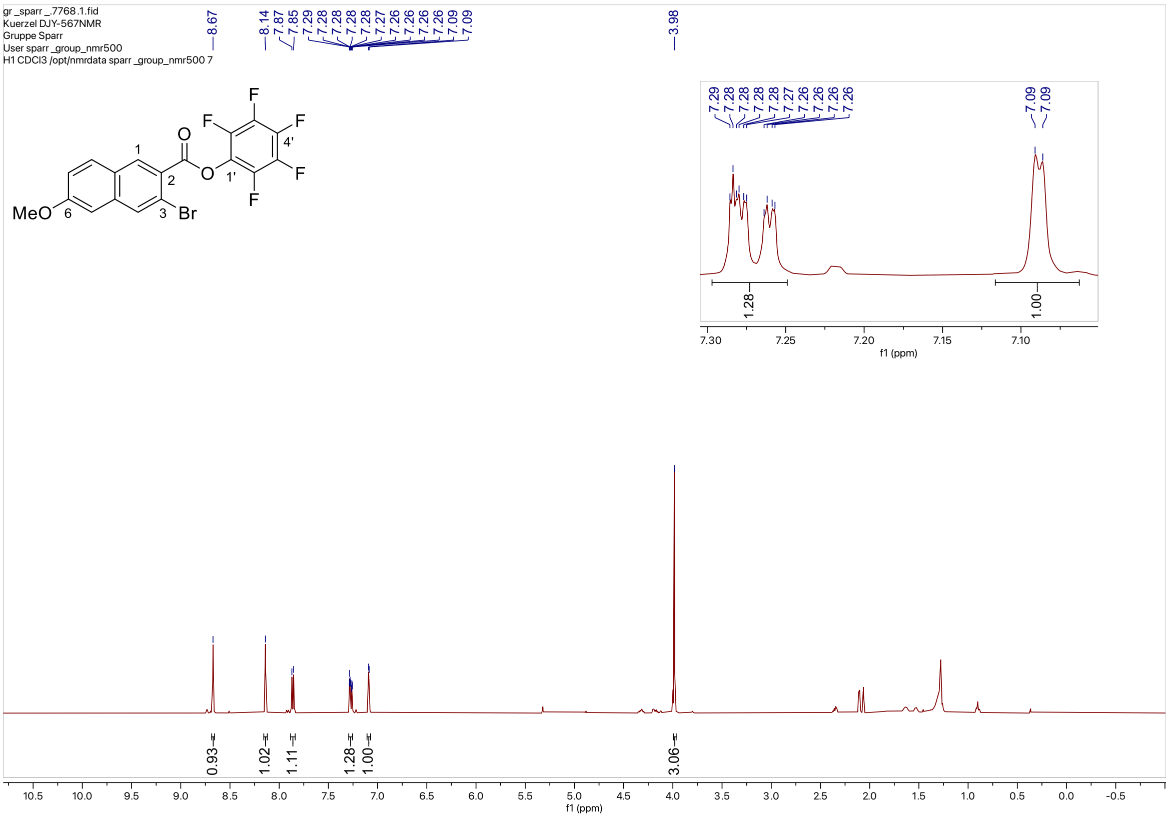

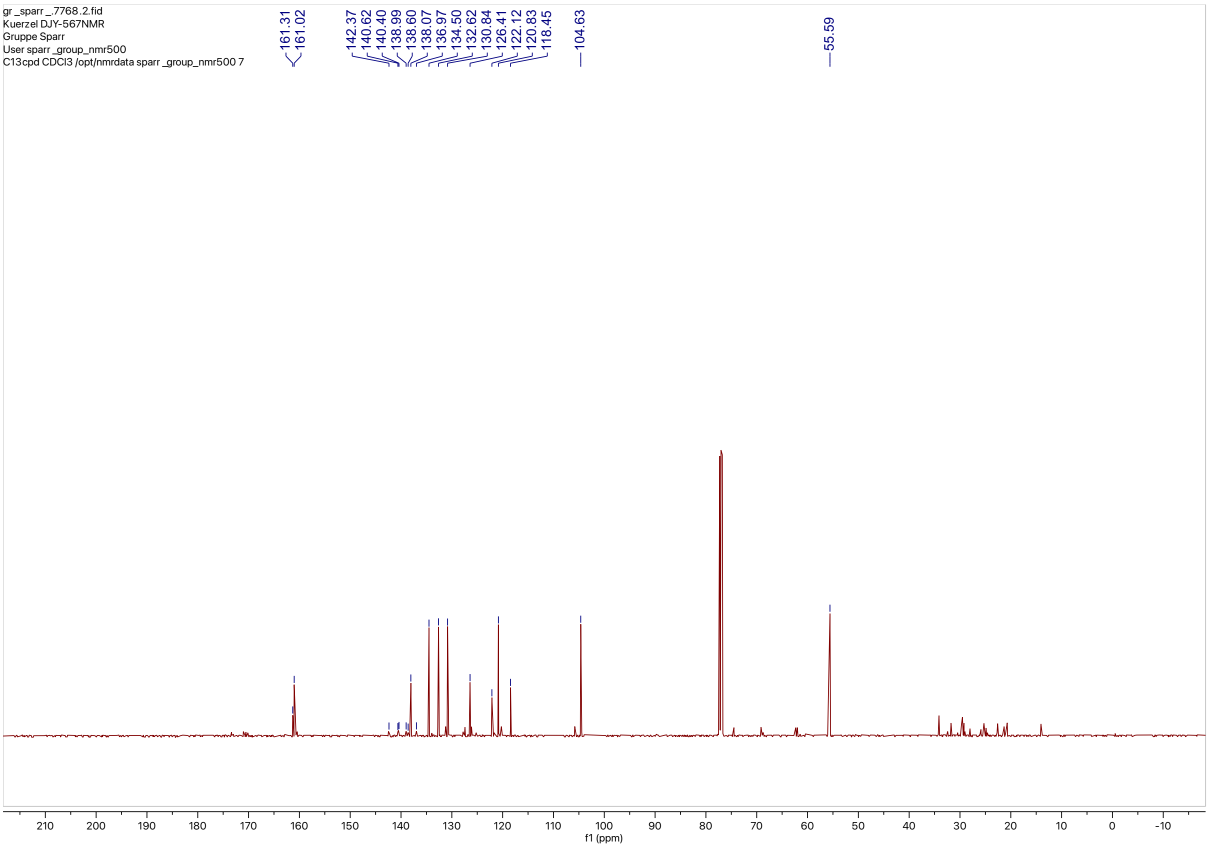

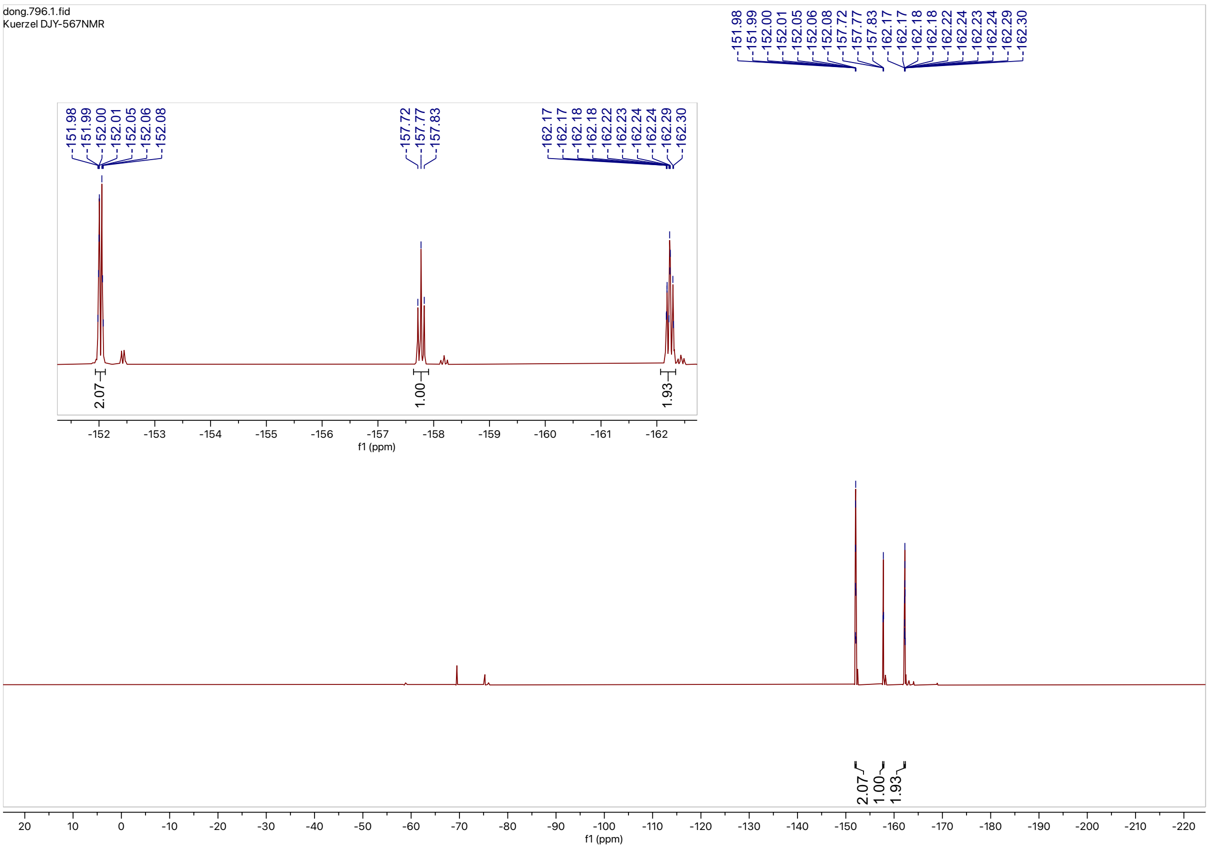

Supplementary Figure 49. ^{1}H (500 MHz, $CDCl_3$, 25 °C), $^{13}C\{^{1}H\}$ (126 MHz, $CDCl_3$, 25 °C) and $^{19}F\{^{1}H\}$ (376 MHz, $CDCl_3$, 25 °C) spectra of **S1g**

[1]H, [13]C{[1]H} and [19]F{[1]H} spectra of ((2-(3-Bromo-6-methoxynaphthalen-2-yl)-1,1,1,3,3,3-hexafluoropropan-2-yl)oxy)trimethylsilane (S2g)

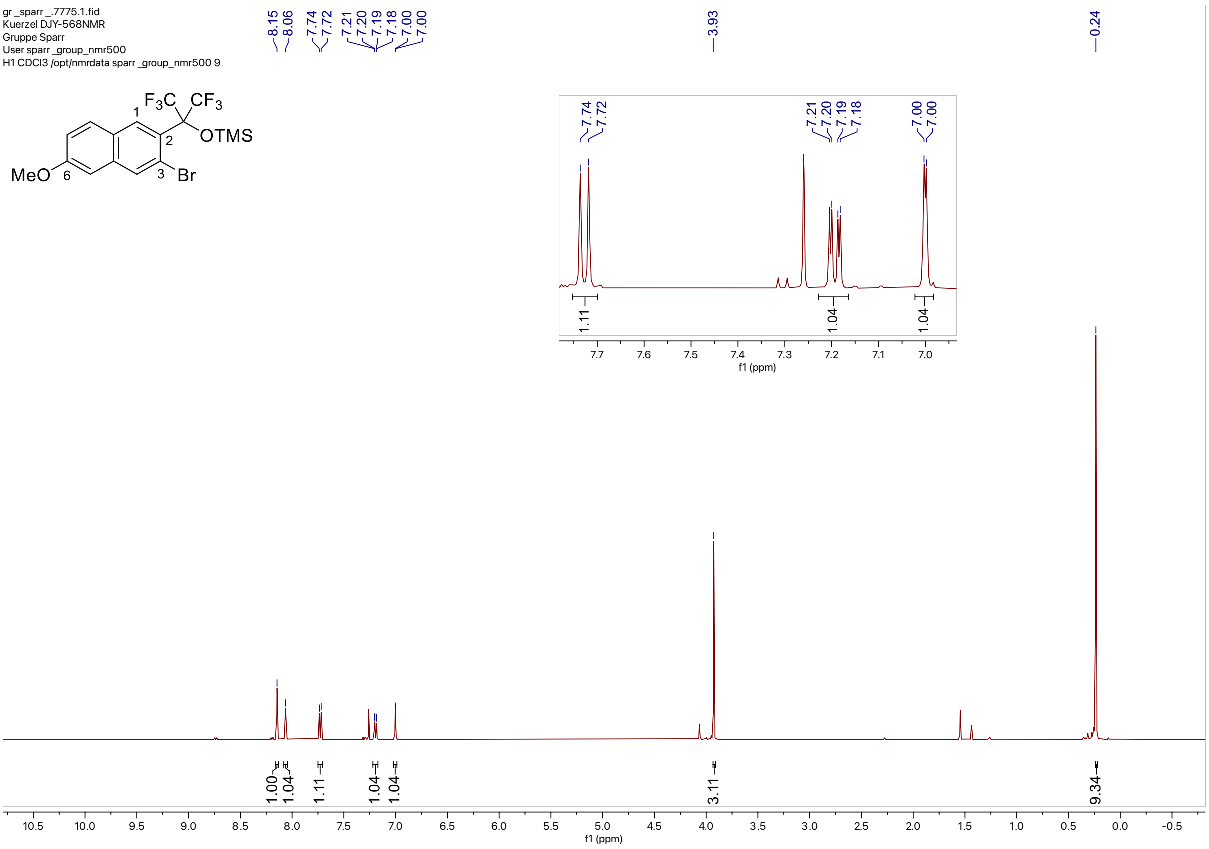

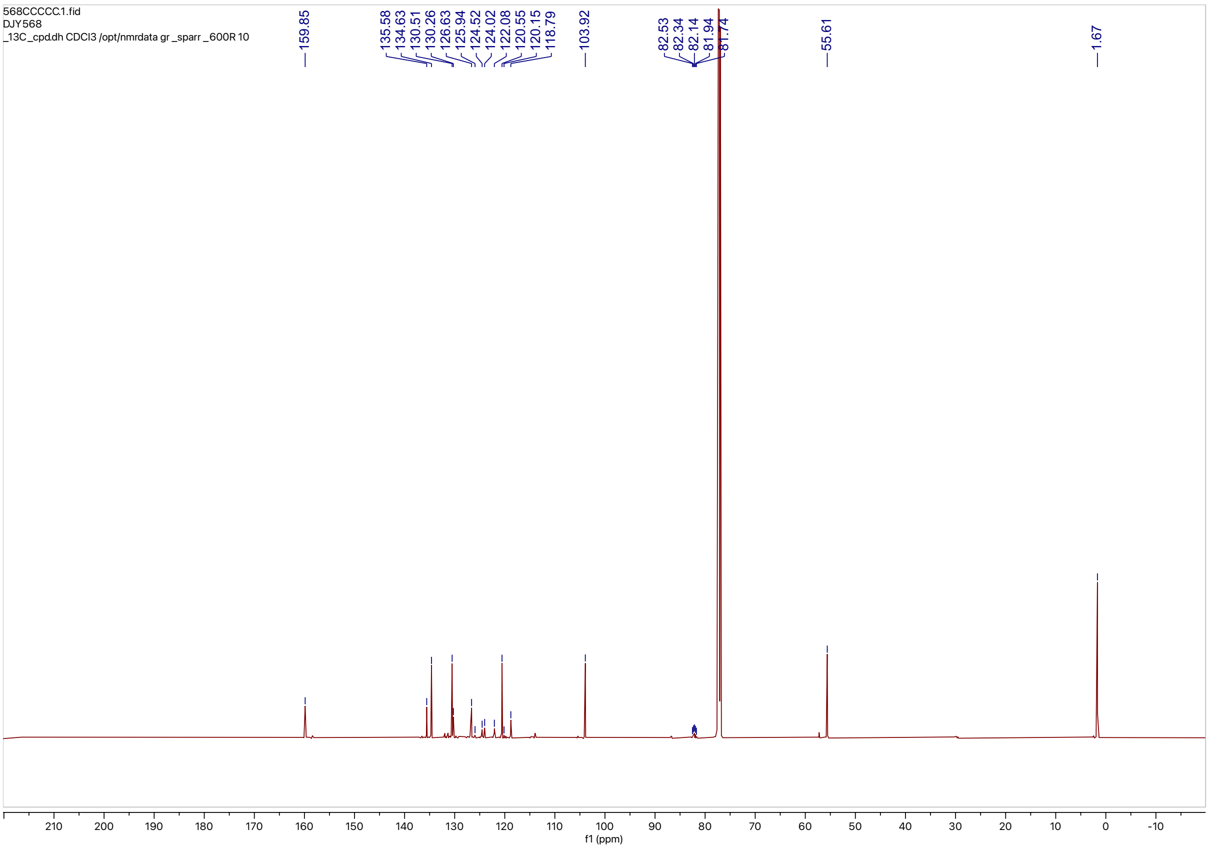

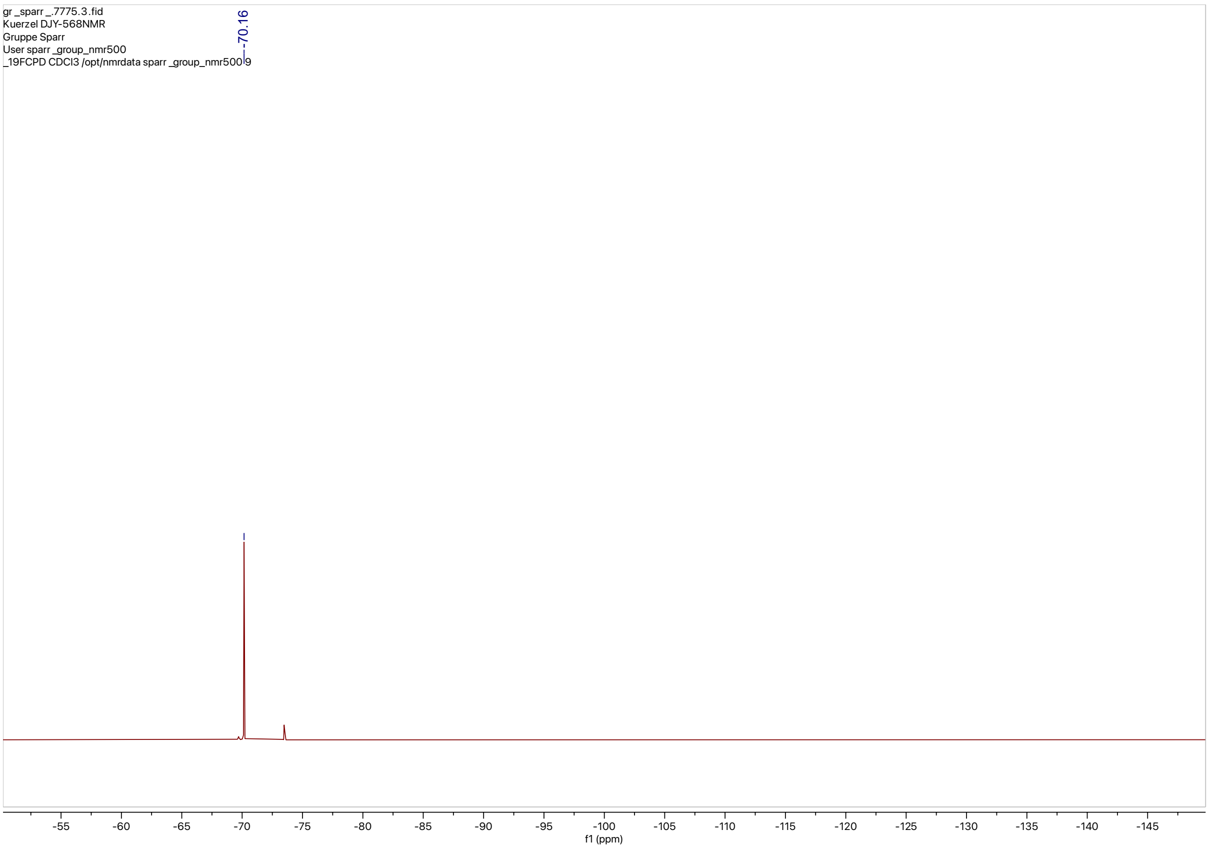

Supplementary Figure 50. ^{1}H (500 MHz, $CDCl_3$, 25 °C), $^{13}C\{^{1}H\}$ (151 MHz, $CDCl_3$, 25 °C) and $^{19}F\{^{1}H\}$ (470 MHz, $CDCl_3$, 25 °C) spectra of **S2g**

[1]H, [13]C{[1]H} and [19]F{[1]H} spectra of 2-(3-Bromo-6-methoxynaphthalen-2-yl)-1,1,1,3,3,3-hexafluoropropan-2-ol (S3g)

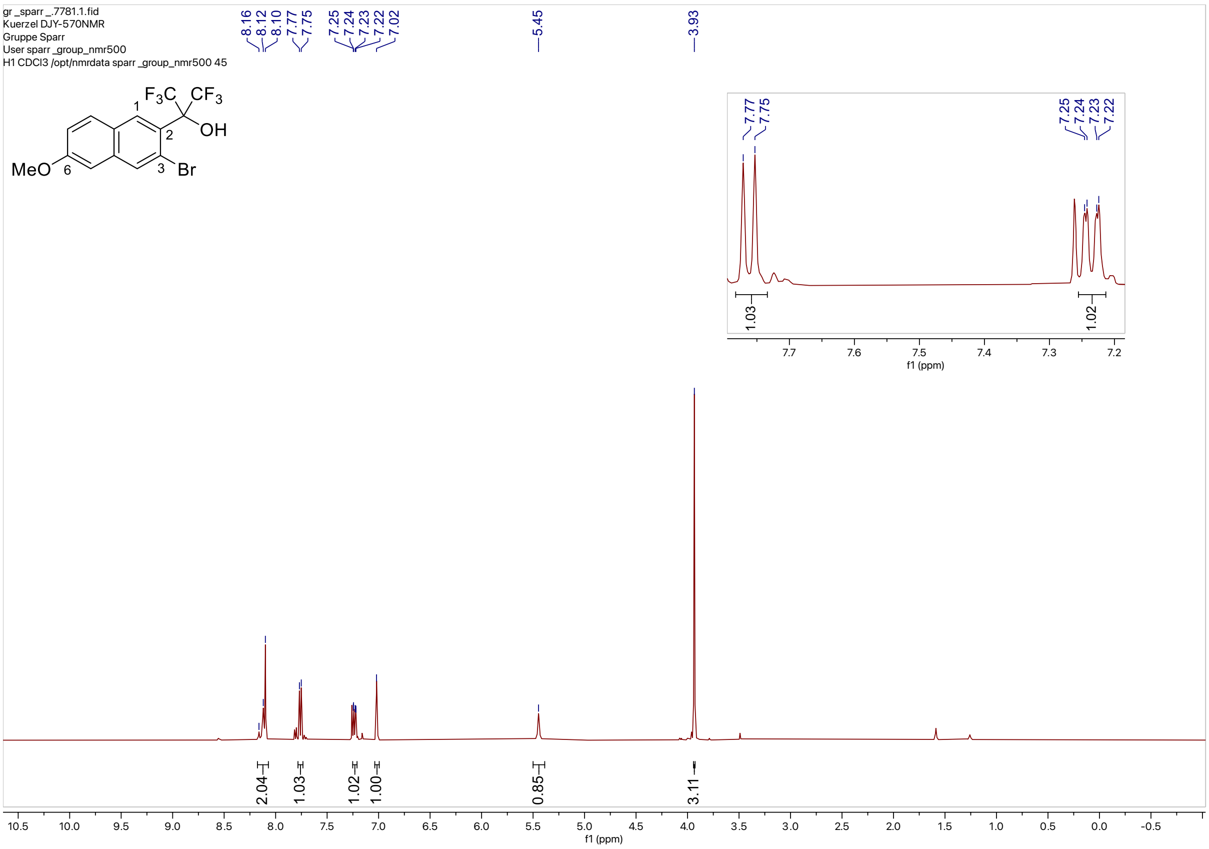

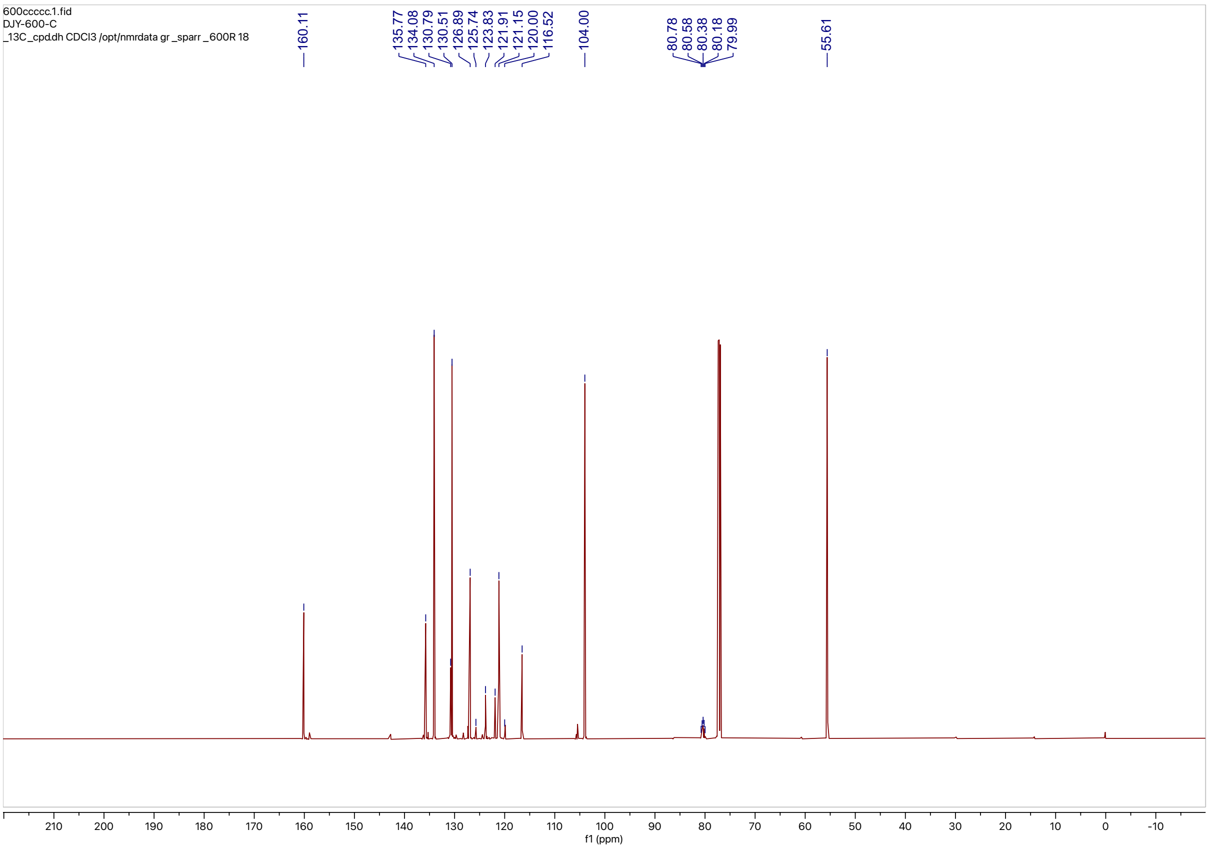

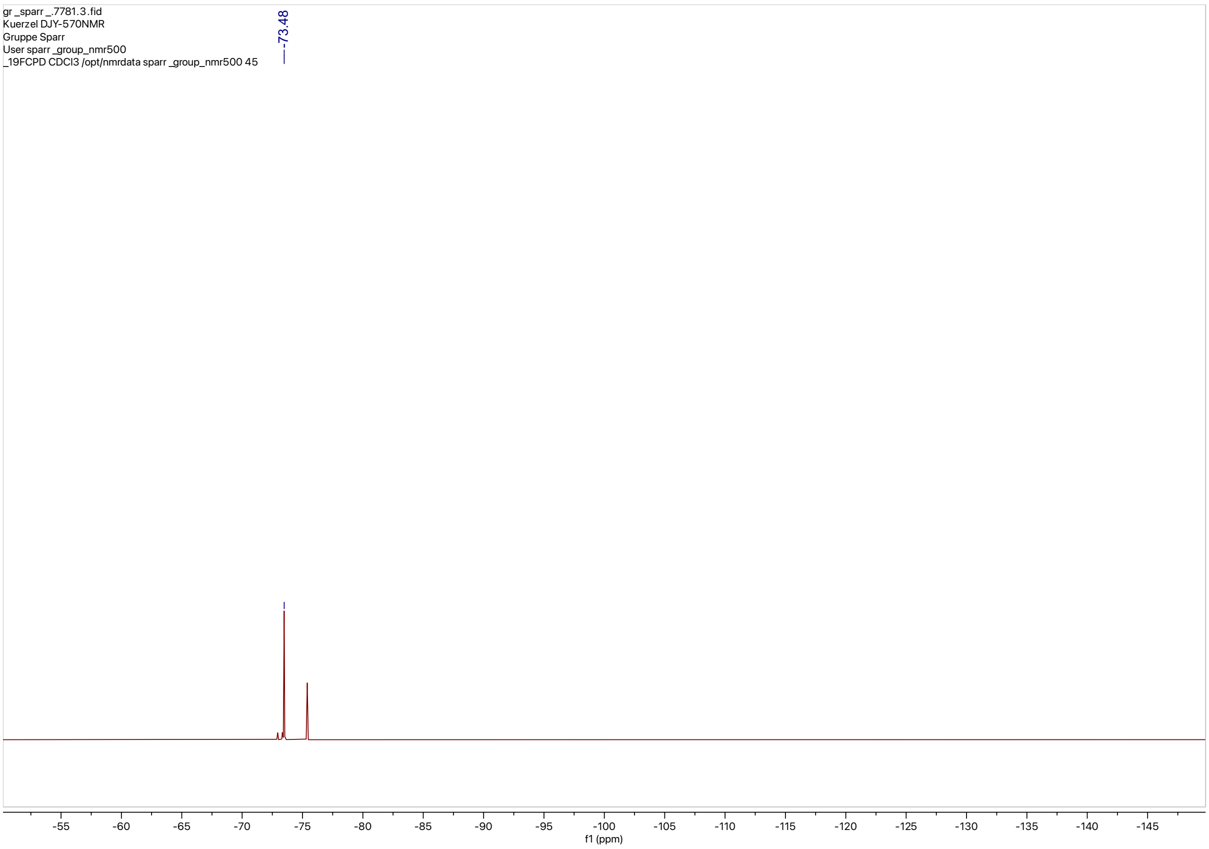

Supplementary Figure 51. ^{1}H (500 MHz, $CDCl_3$, 25 °C), $^{13}C\{^{1}H\}$ (151 MHz, $CDCl_3$, 25 °C) and $^{19}F\{^{1}H\}$ (470 MHz, $CDCl_3$, 25 °C) spectra of **S3g**

[1]H, [13]C{[1]H}, [19]F{[1]H} and [31]P{[1]H} spectra of [*TBPY*-5-11']-7,7'-Dimethoxy-1-hydro-3,3,3',3'-tetrakis(trifluoromethyl)-3*H*,3'*H*-1λ[5],1'-spirobi[naphtho[2,3-*c*][2,1]oxaphosphole] (S4g)

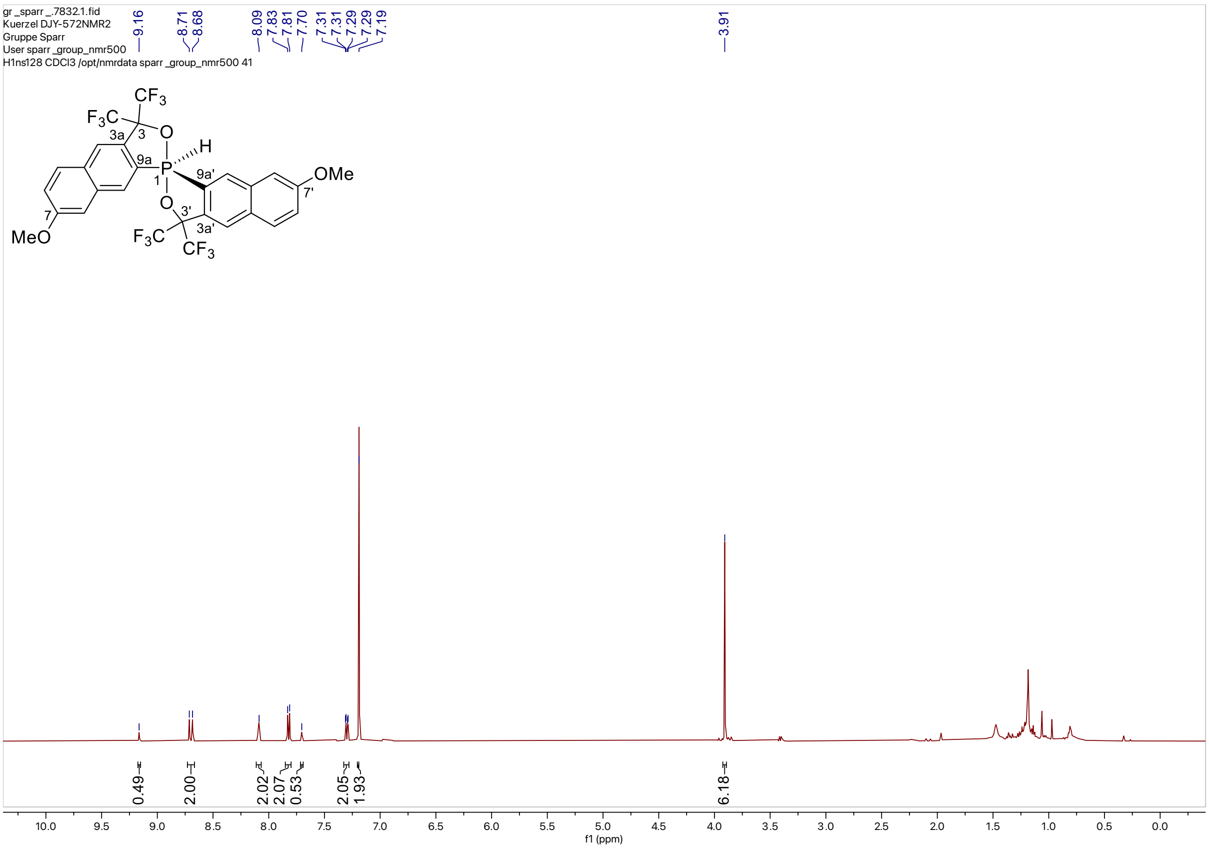

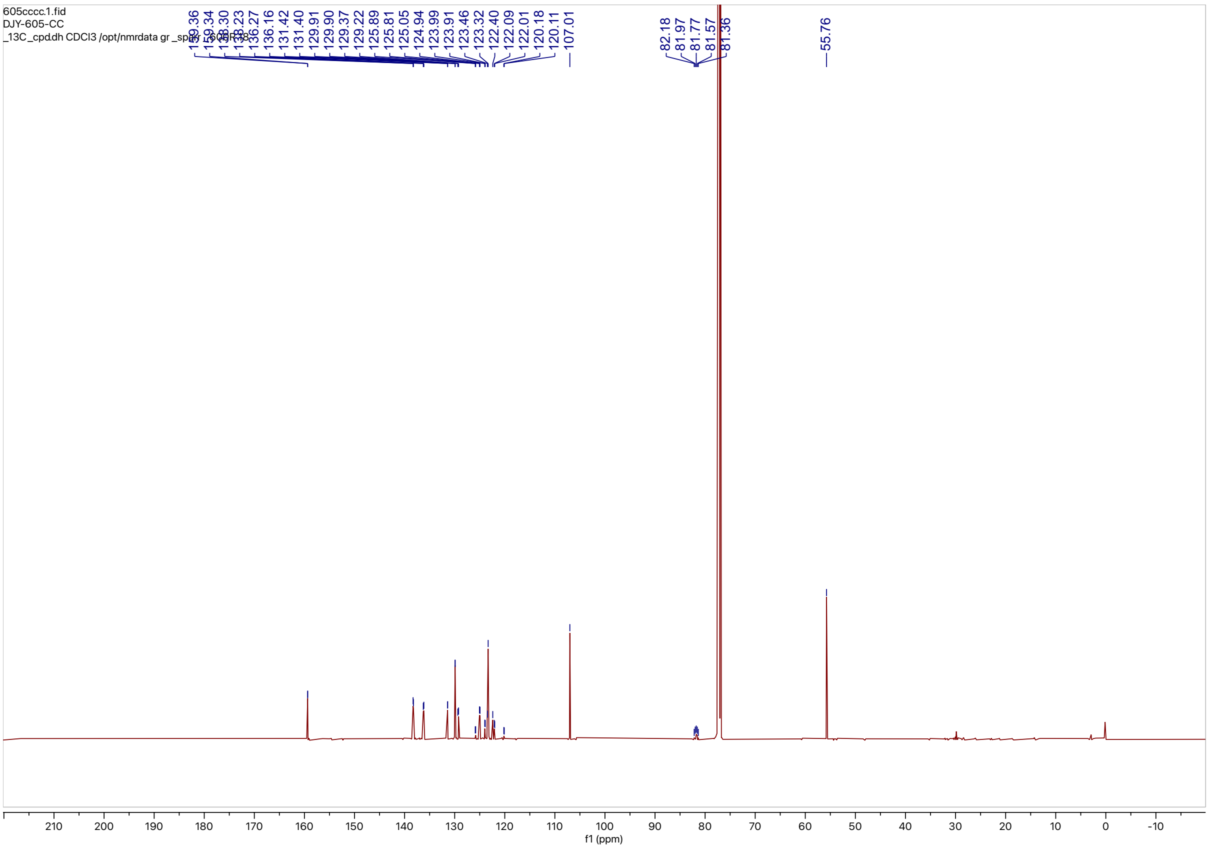

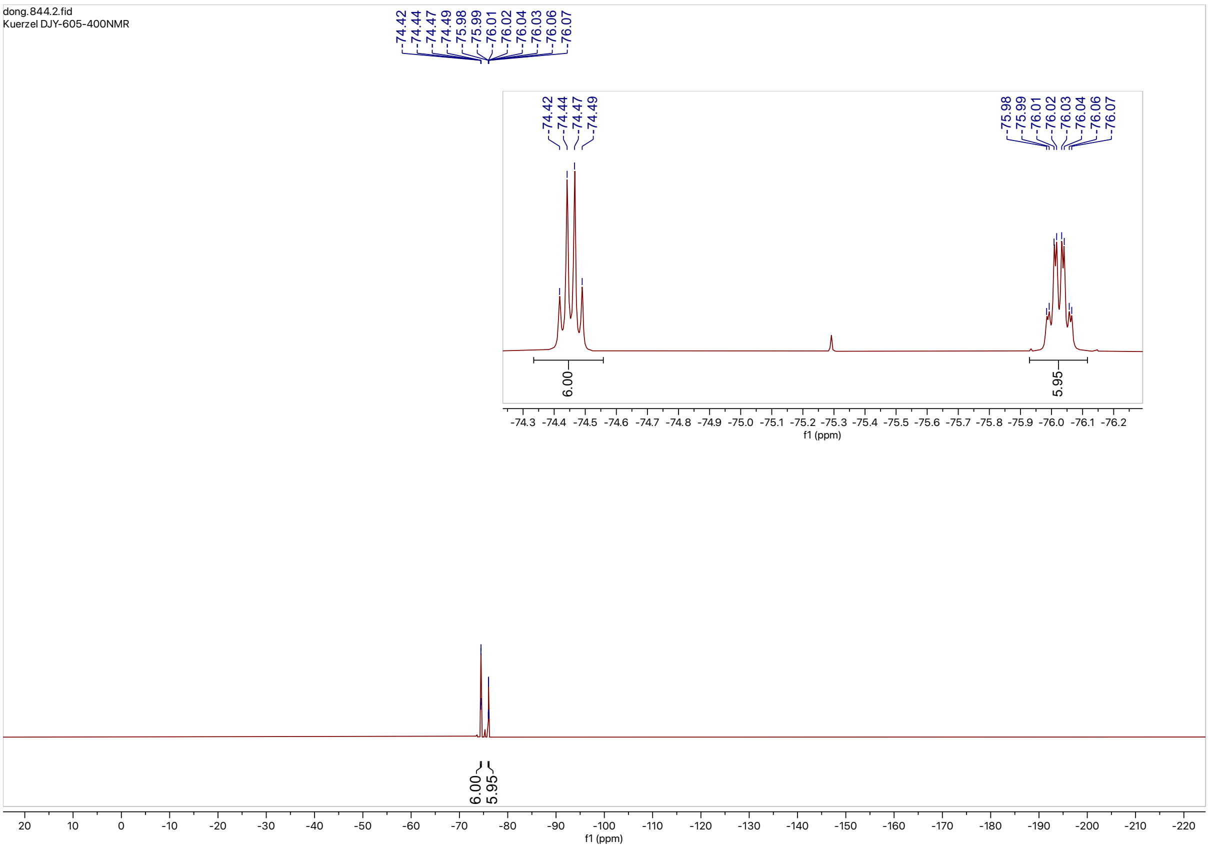

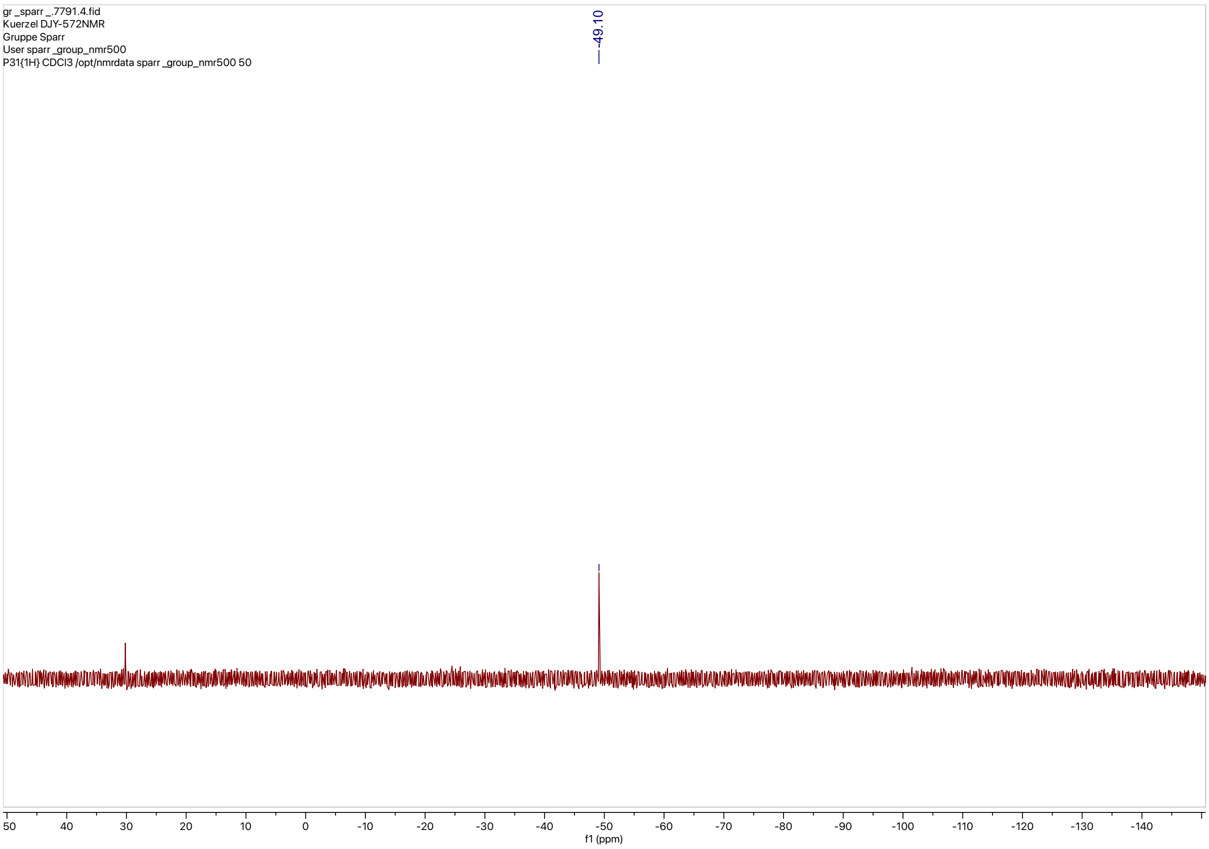

Supplementary Figure 52. ^{1}H (500 MHz, $CDCl_3$, 25 °C), $^{13}C\{^{1}H\}$ (151 MHz, $CDCl_3$, 25 °C), $^{19}F\{^{1}H\}$ (376 MHz, $CDCl_3$, 25 °C) and $^{31}P\{^{1}H\}$ (202 MHz, $CDCl_3$, 25 °C) spectra of **S4g**

^{1}H, ^{19}F{^{1}H} and ^{31}P{^{1}H} spectra of [*TBPY*-5-15]-1,1,1,3,3,3-Hexafluoro-2-(3-(1-butyl-1-hydro-7-methoxy-3,3-bis(trifluoromethyl)-3*H*-1λ^5-naphtho[2,3-*c*][2,1]oxaphosphol-1-yl)-6-methoxynaphthalen-2-yl)propan-2-ol (1g)

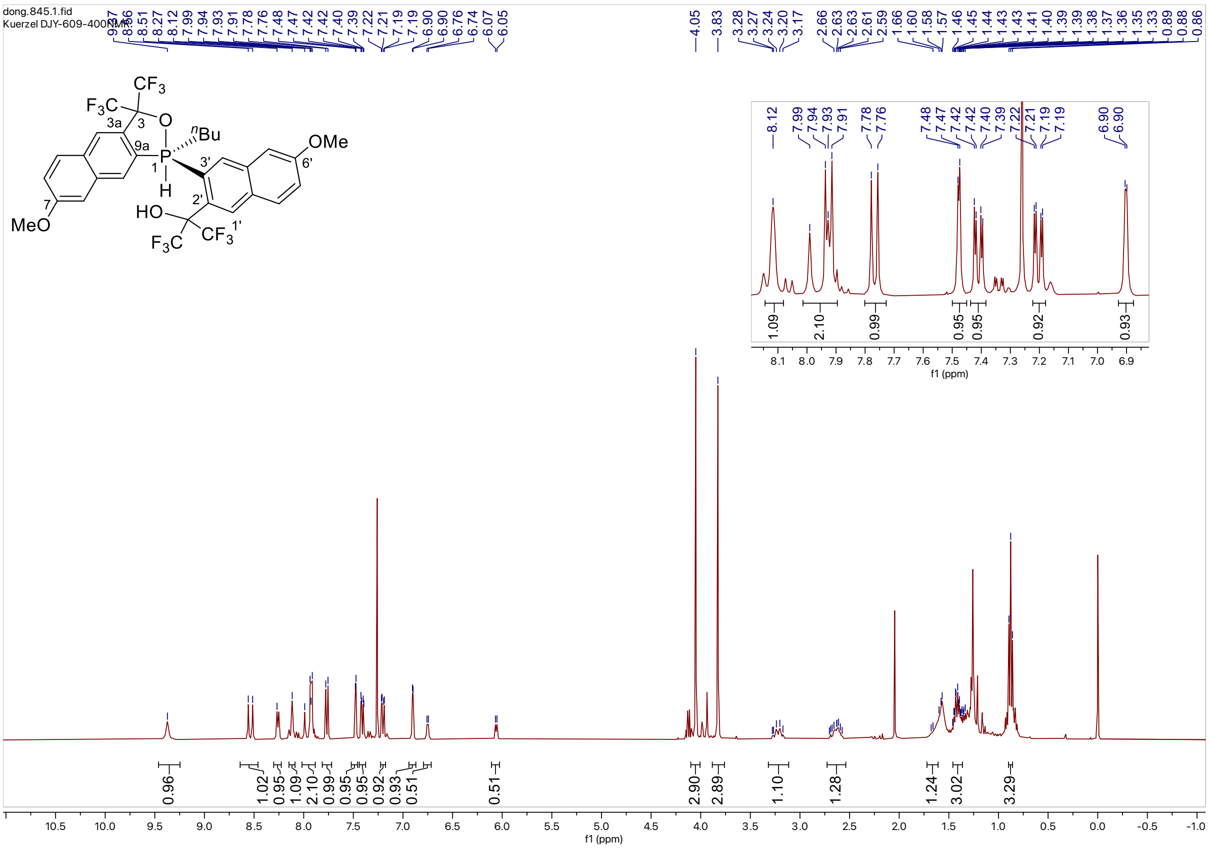

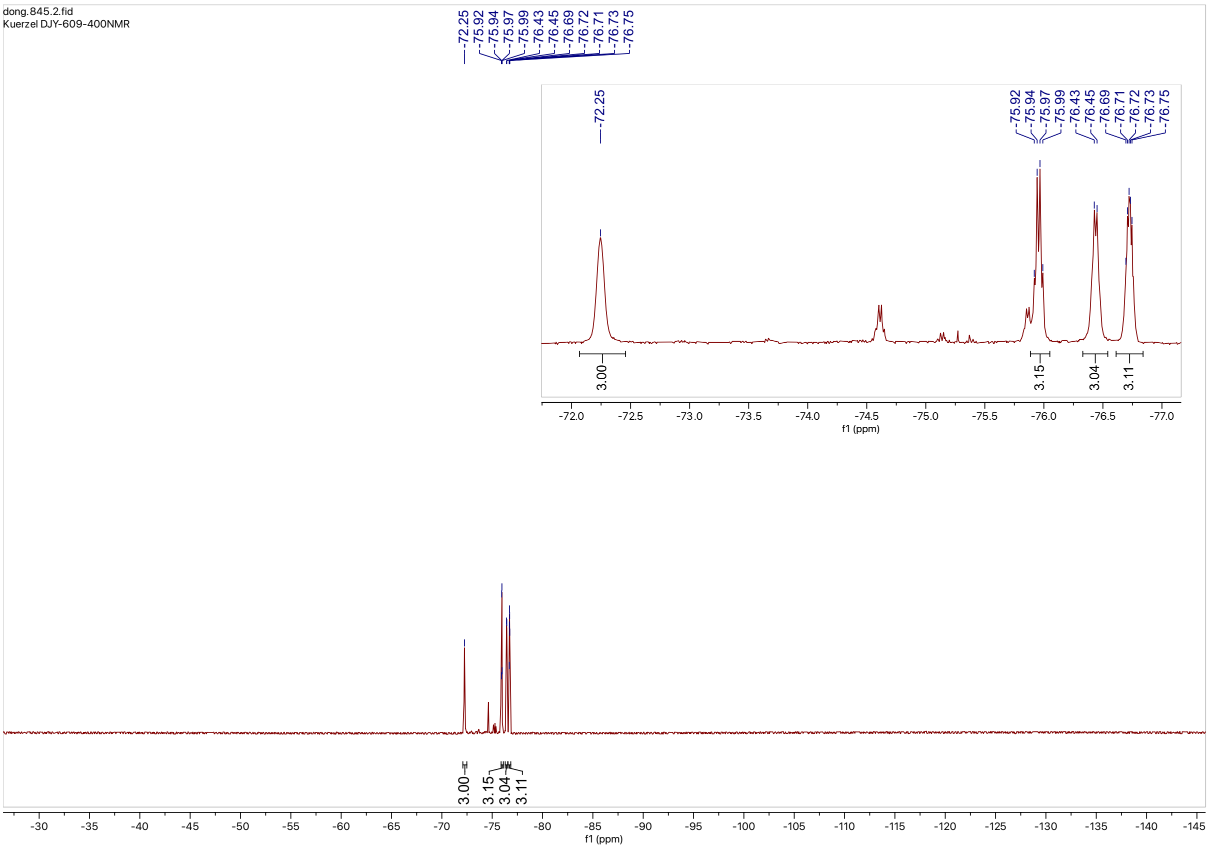

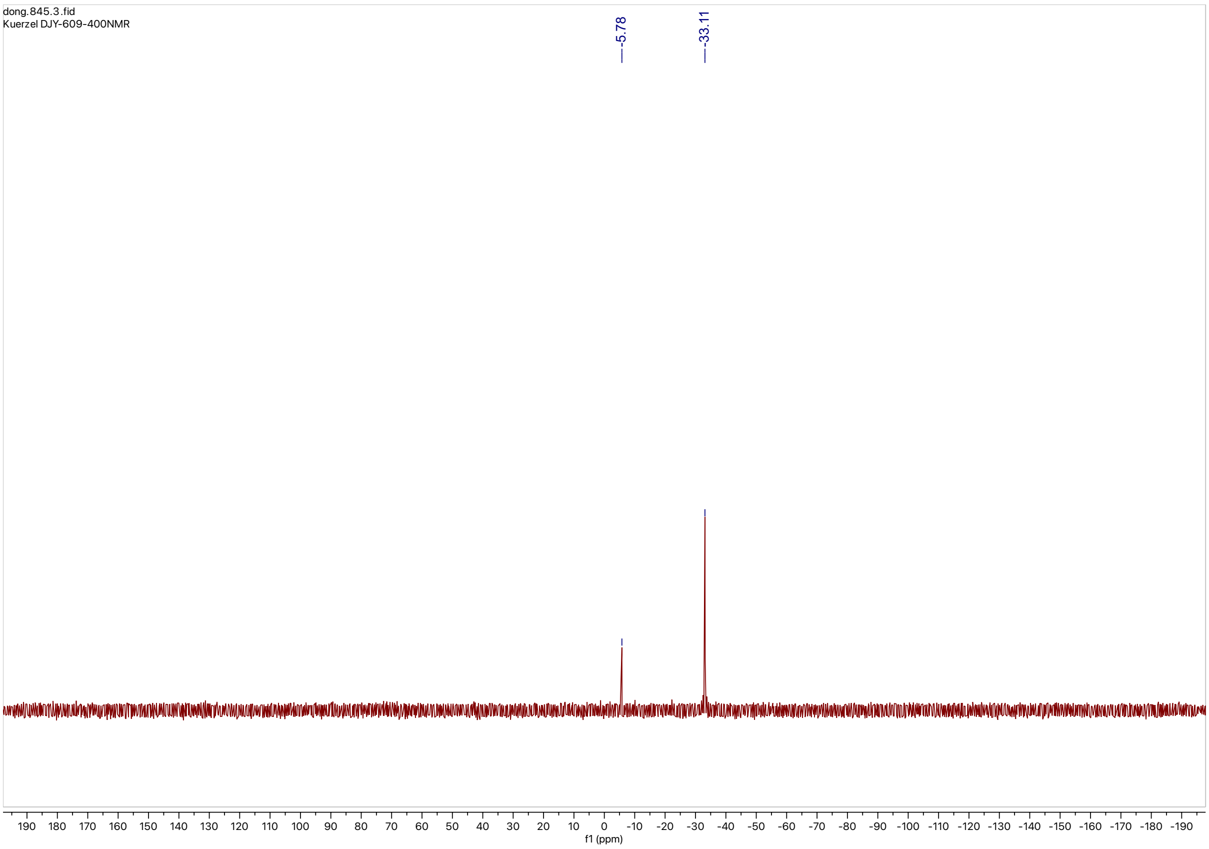

Supplementary Figure 53. ^{1}H (400 MHz, $CDCl_3$, 25 °C), $^{19}F\{^{1}H\}$ (376 MHz, $CDCl_3$, 25 °C) and $^{31}P\{^{1}H\}$ (162 MHz, $CDCl_3$, 25 °C) spectra of **1g**

[1]H, [19]F{[1]H} and [31]P{[1]H} spectra of [*TBPY*-5-15]-1,1,1,3,3,3-Hexafluoro-2-(4-methyl-2-(1-hexyl-1-hydro-6-methyl-3,3-bis(trifluoromethyl)-3*H*-1λ[5]-benzo[*c*][2,1]oxaphosphol-1-yl)phenyl)propan-2-ol (1h)

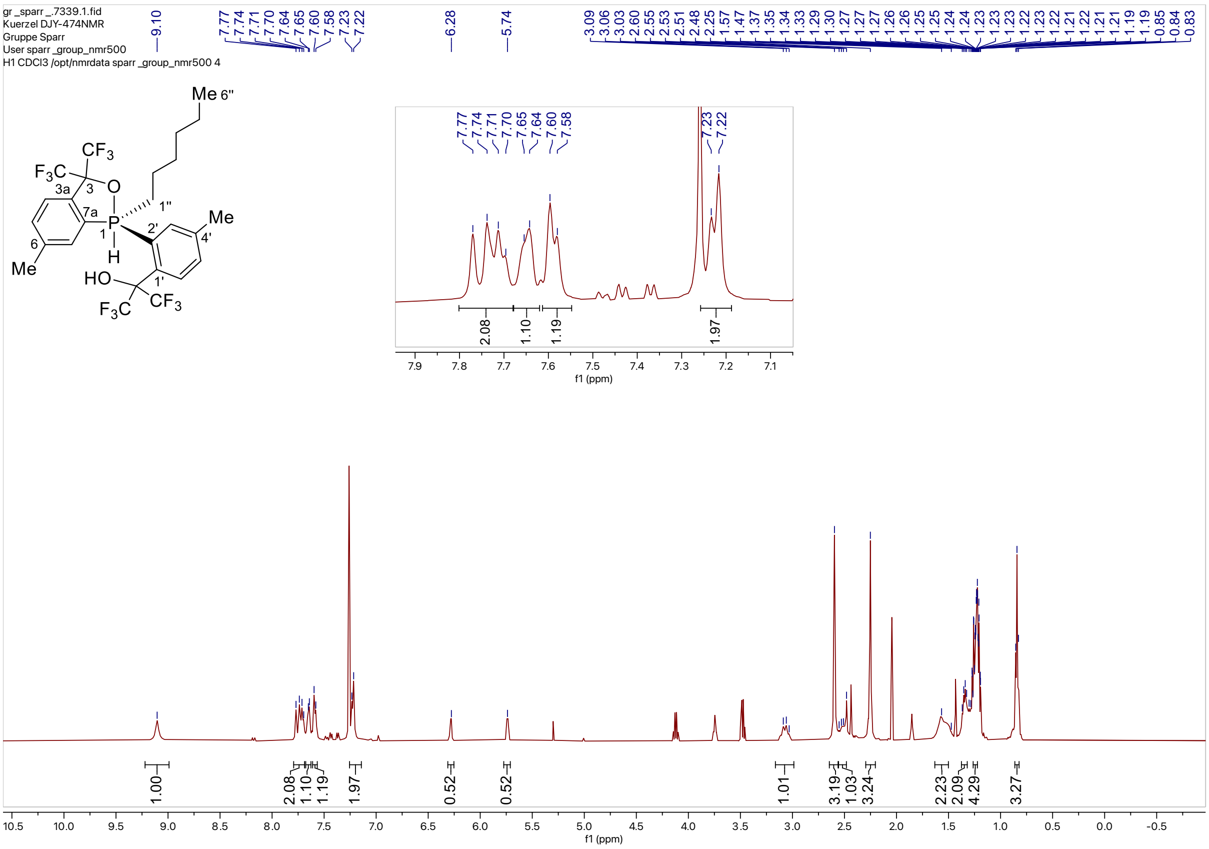

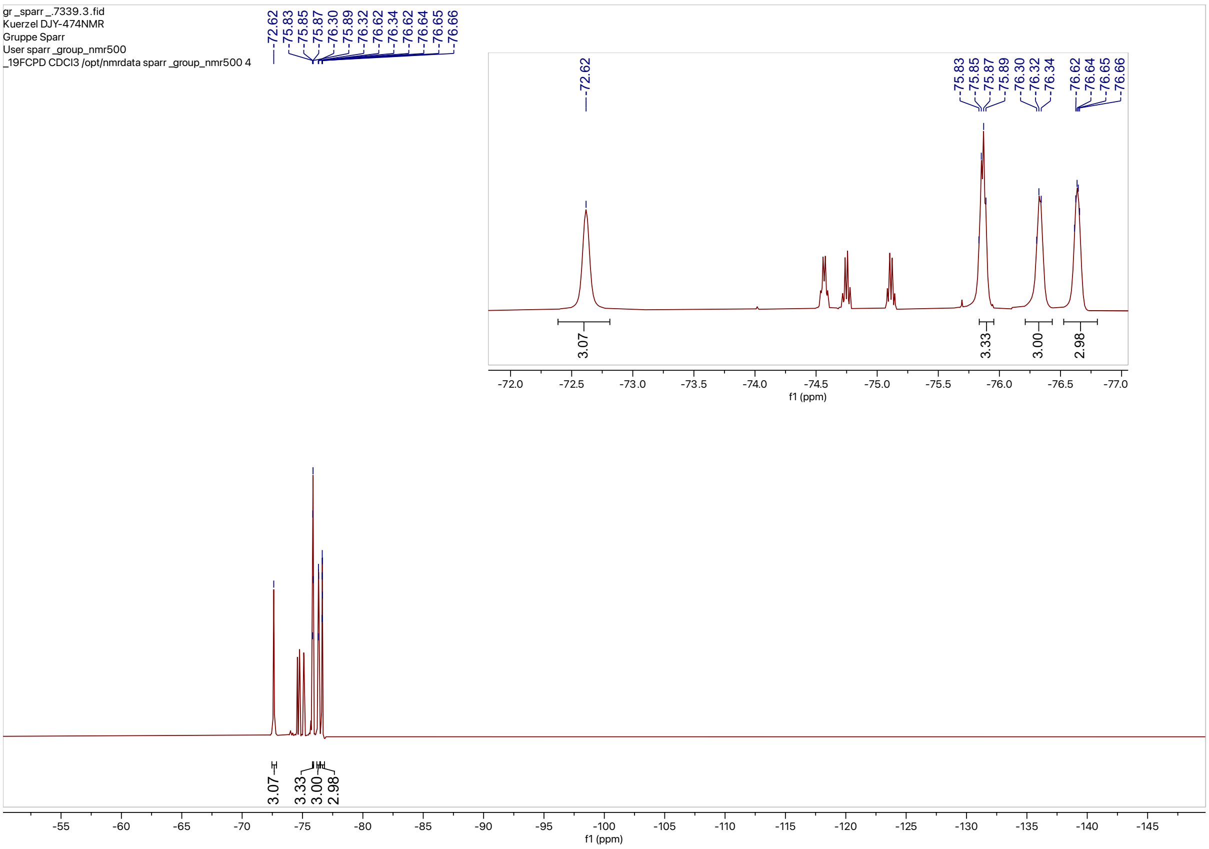

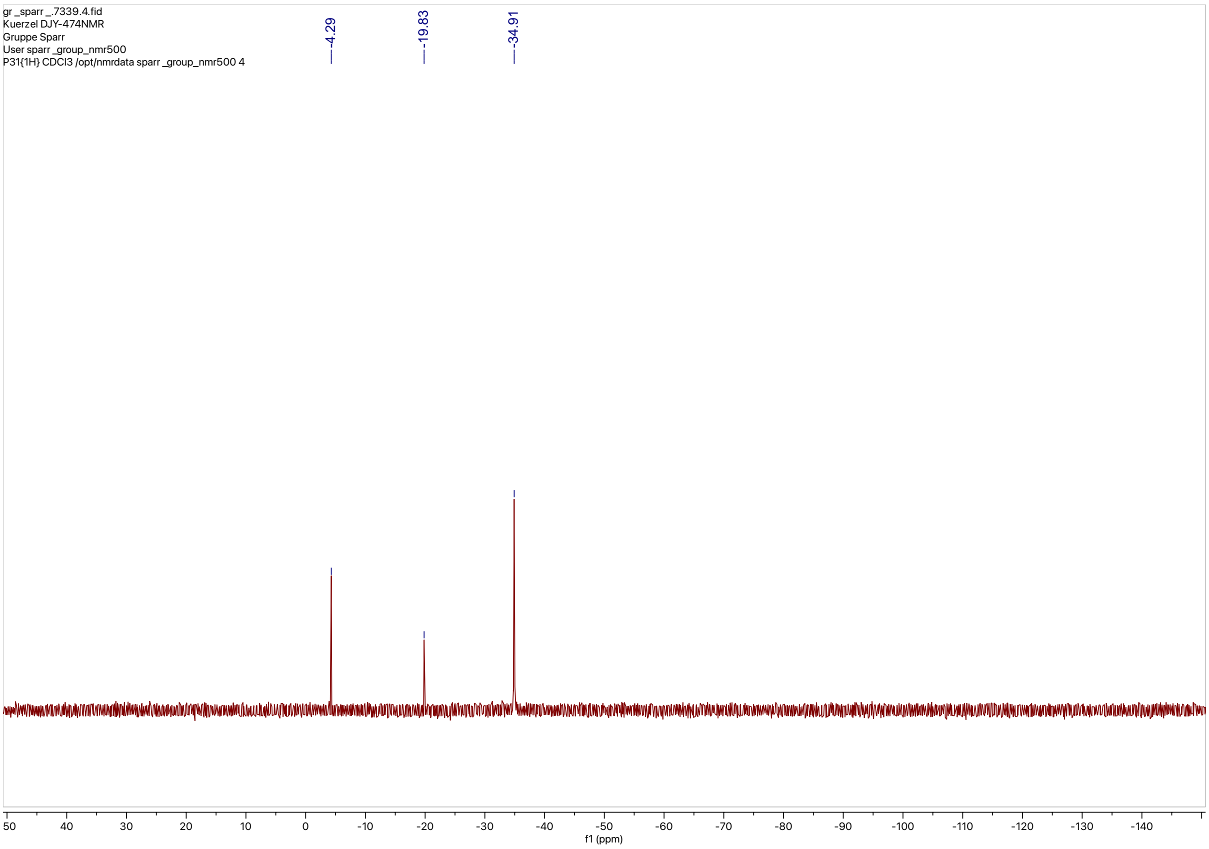

Supplementary Figure 54. ^{1}H (500 MHz, $CDCl_3$, 25 °C), $^{19}F\{^{1}H\}$ (470 MHz, $CDCl_3$, 25 °C) and $^{31}P\{^{1}H\}$ (202 MHz, $CDCl_3$, 25 °C) spectra of **1h**

^{1}H, ^{19}F{^{1}H}, ^{31}P{^{1}H} NMR spectra of [*TBPY*-5-15]-1,1,1,3,3,3-Hexafluoro-2-(4-methyl-2-(1-hydro-6-methyl-1-(5-methylhexyl)-3,3-bis(trifluoro-methyl)-3*H*-1λ^{5}-benzo[*c*][2,1]oxaphosphol-1-yl)phenyl)propan-2-ol (1i)

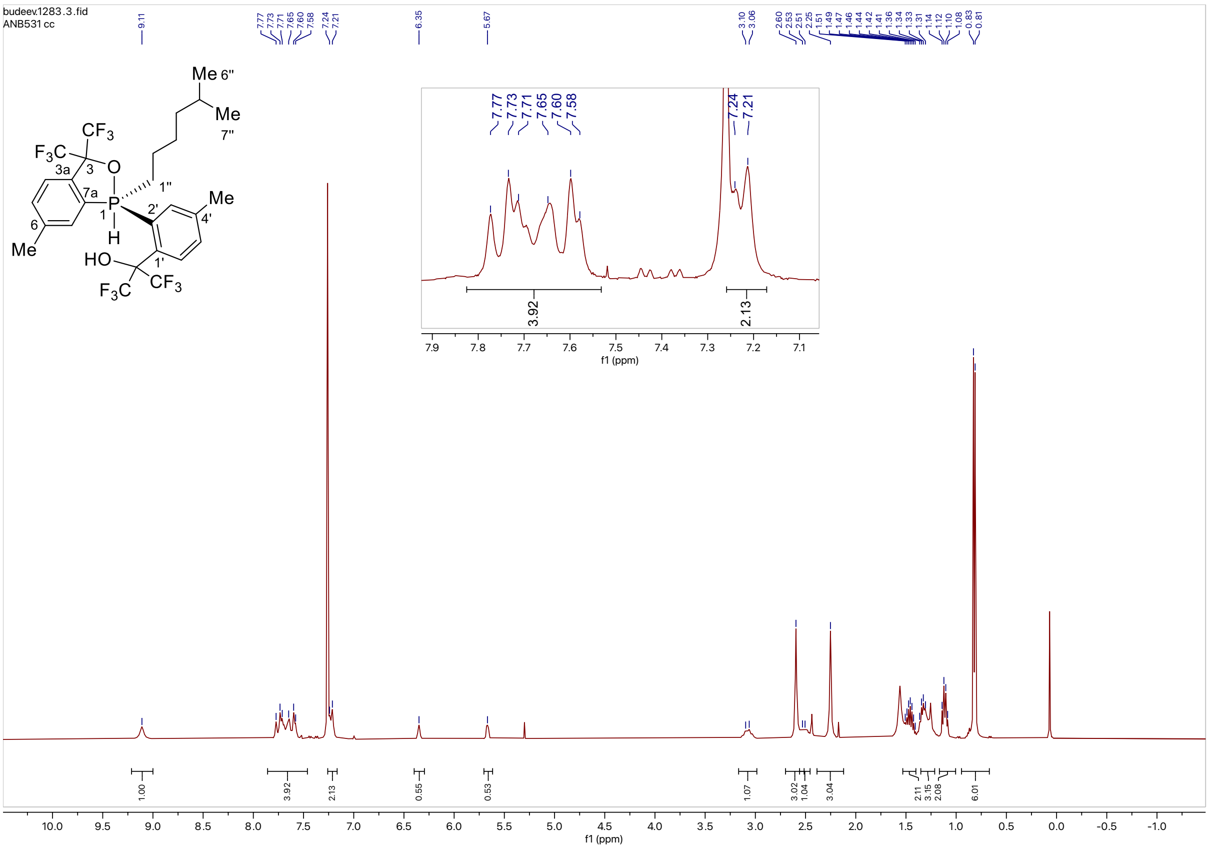

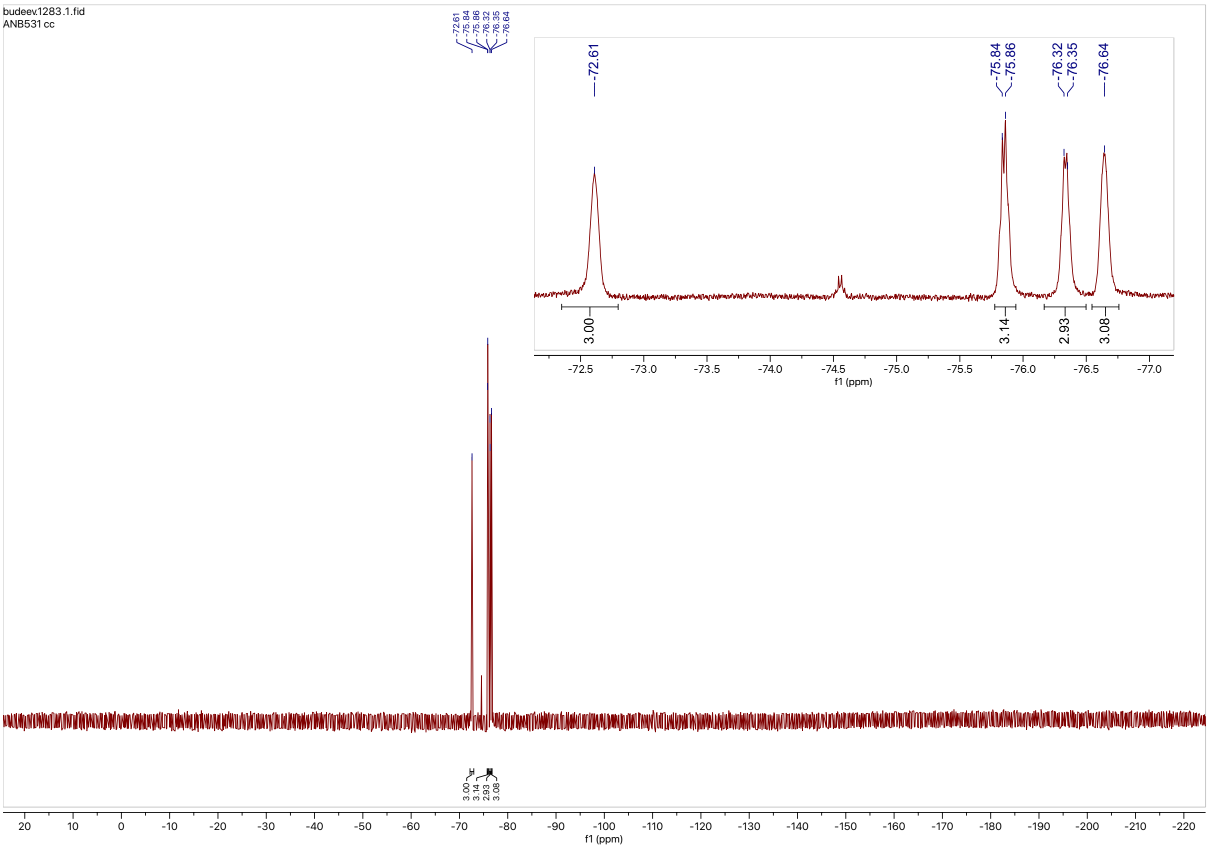

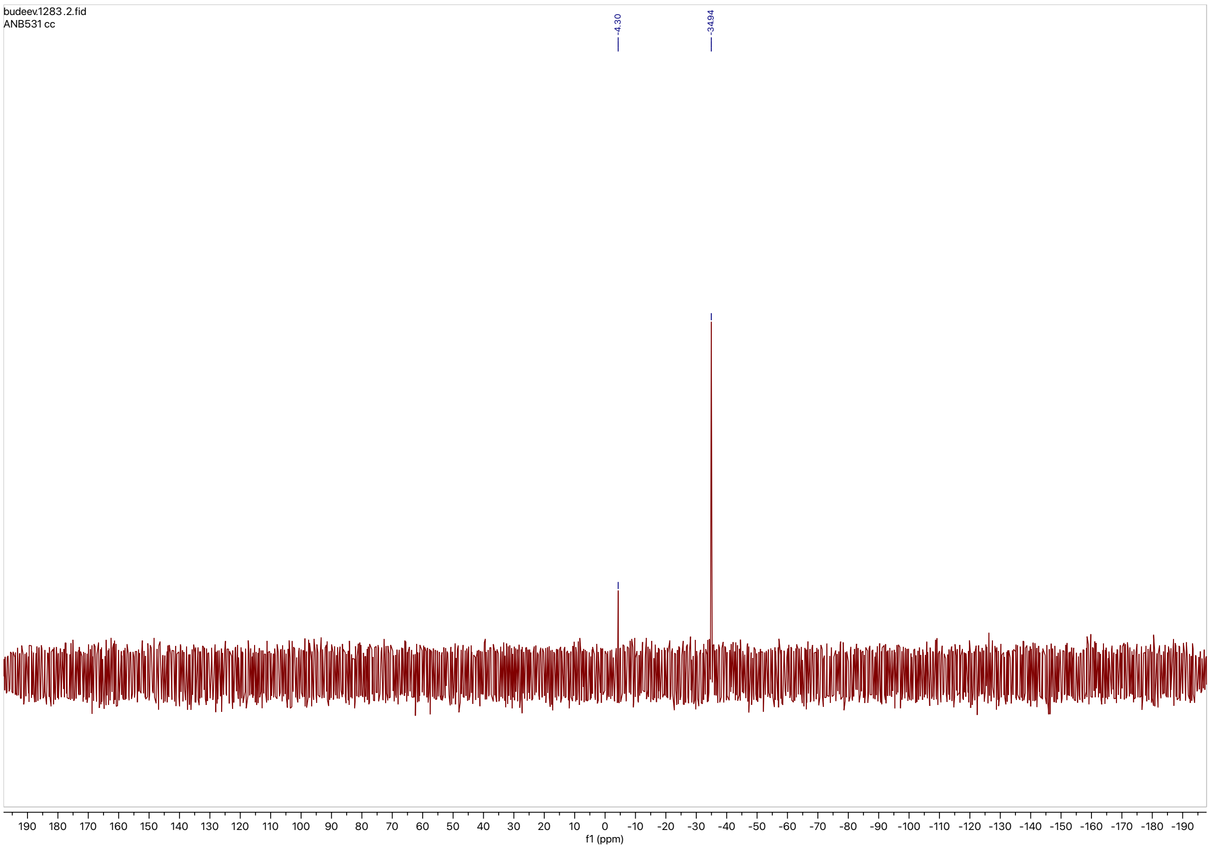

Supplementary Figure 55. ^{1}H (400 MHz, $CDCl_3$, 25 °C), ^{19}F{^{1}H} (376 MHz, $CDCl_3$, 25 °C) and ^{31}P{^{1}H} (162 MHz, $CDCl_3$, 25 °C) spectra of **1i**

^{1}H, $^{19}F\{^{1}H\}$ and $^{31}P\{^{1}H\}$ spectra of [*TBPY*-5-15]-1,1,1,3,3,3-Hexafluoro-2-(4-methyl-2-(1-hydro-6-methyl-1- (4-phenylbutyl)-3,3-bis(trifluoro-methyl)-3*H*-1λ^{5}-benzo[*c*][2,1]oxaphosphol-1-yl)phenyl)propan-2-ol (1j)

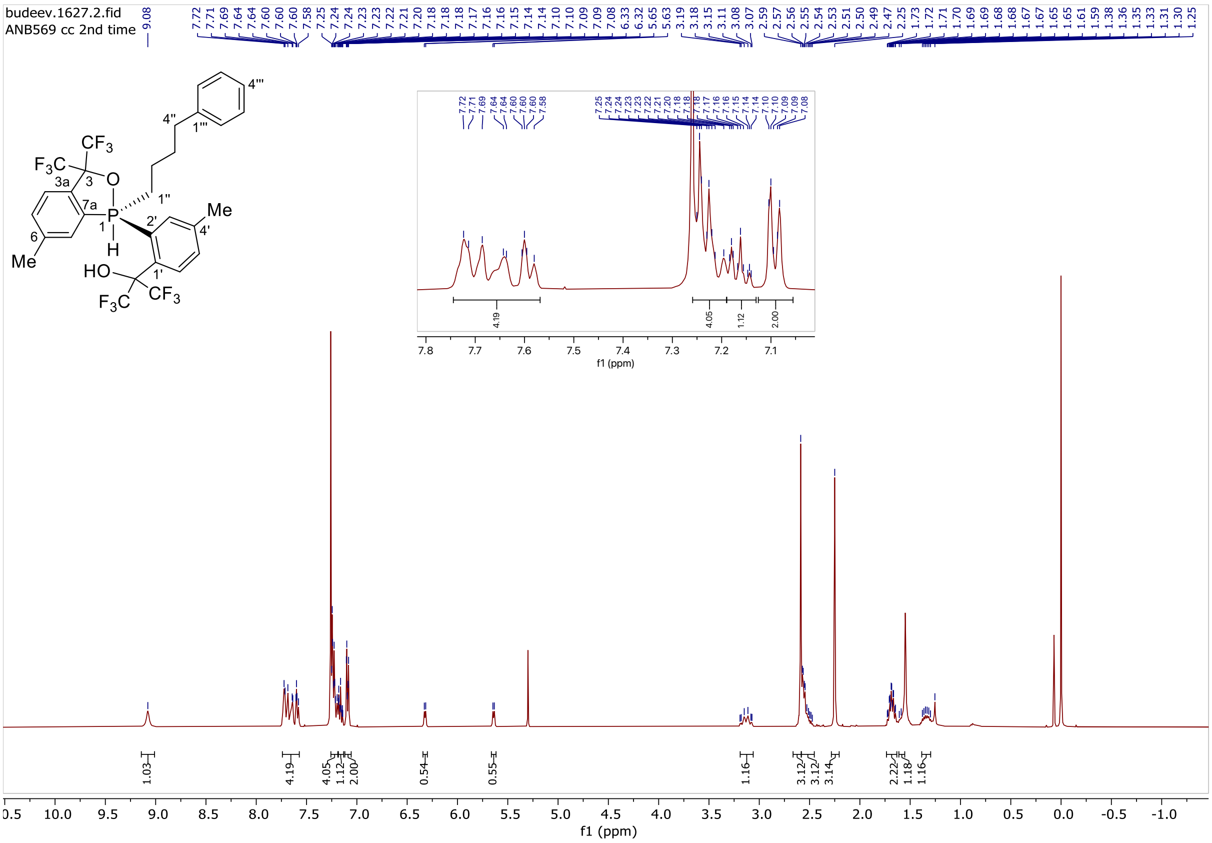

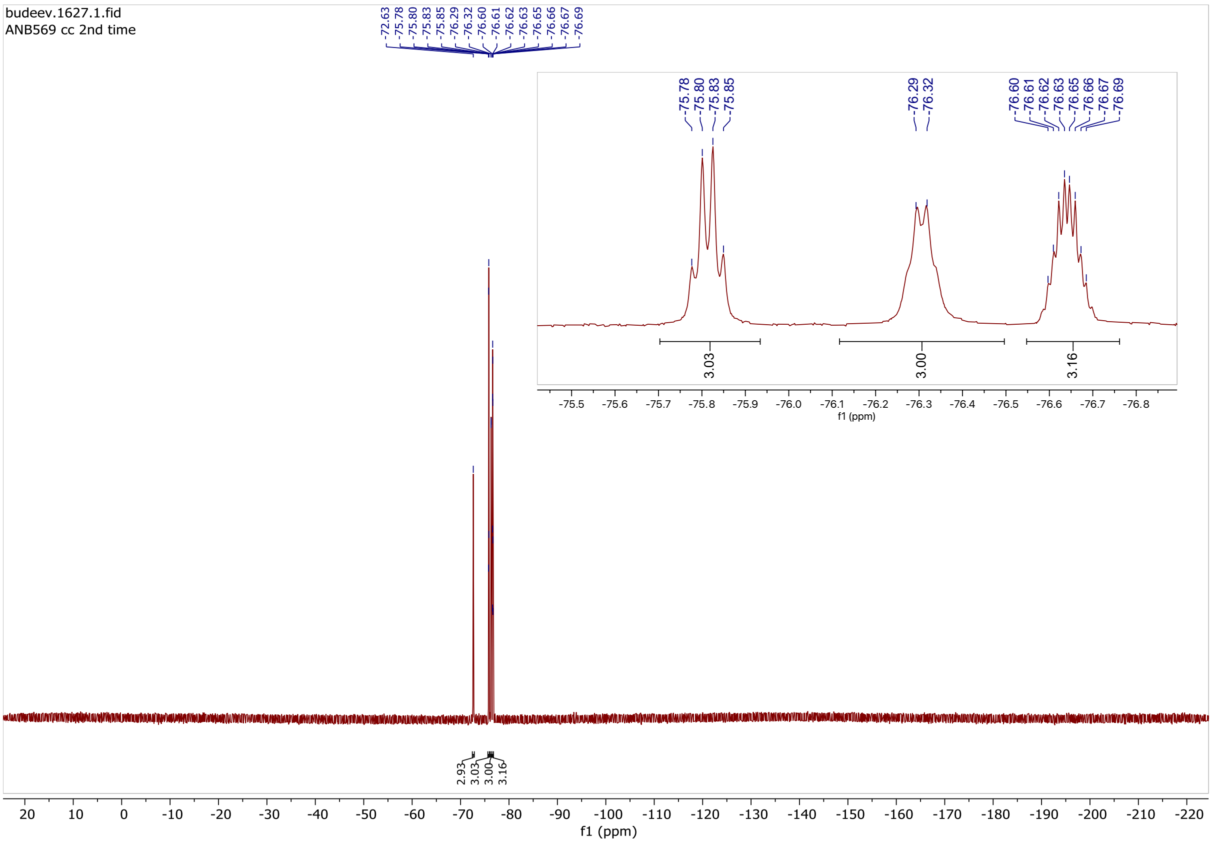

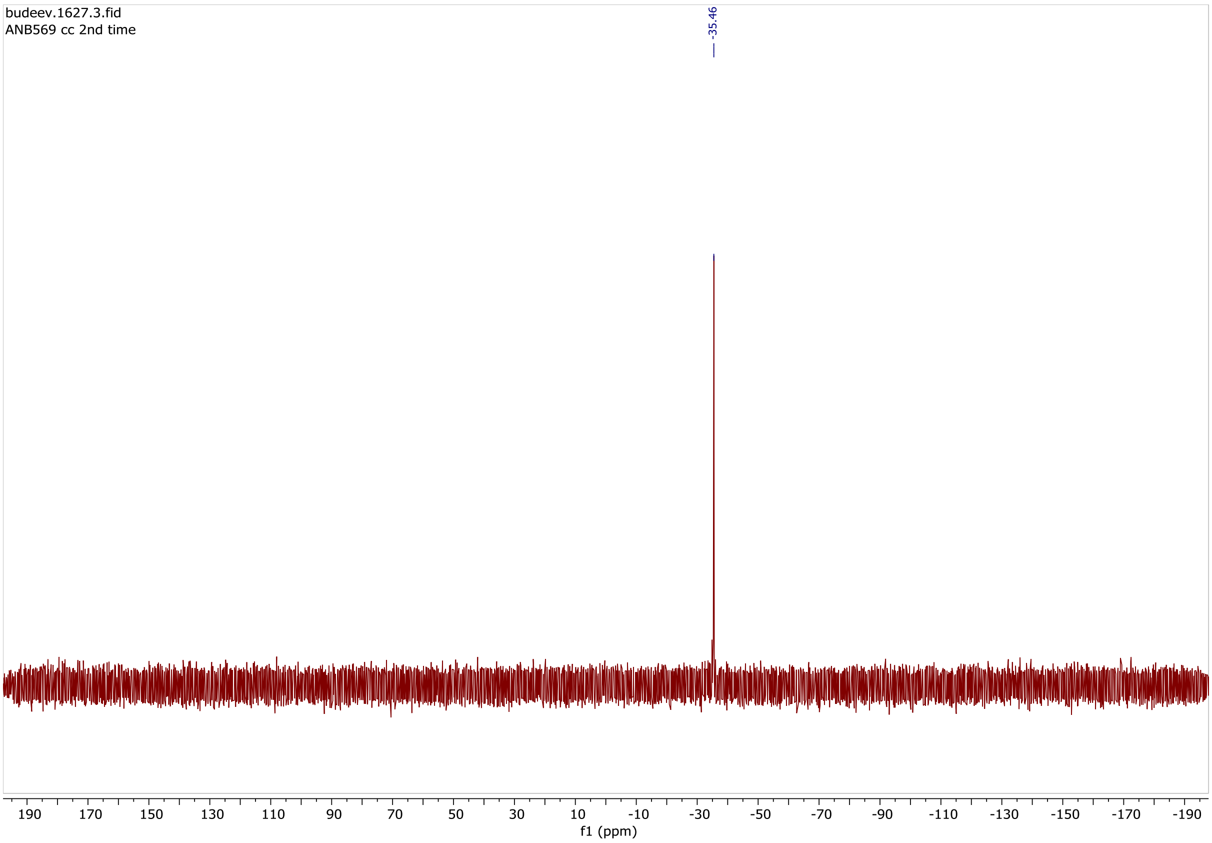

Supplementary Figure 56. ^1H (400 MHz, $CDCl_3$, 25 °C), $^{19}F\{^1H\}$ (376 MHz, $CDCl_3$, 25 °C) and $^{31}P\{^1H\}$ (162 MHz, $CDCl_3$, 25 °C) spectra of **1j**

[1]H, [19]F{[1]H} and [31]P{[1]H} spectra of [*TBPY*-5-15]-1,1,1,3,3,3-Hexafluoro-2-(4-methyl-2-(1-hydro-6-methyl-1- (pent-4-en-1-yl)-3,3-bis(trifluoromethyl)-3*H*-1λ[5]-benzo[*c*][2,1]oxaphosphol-1-yl)phenyl)propan-2-ol (1k)

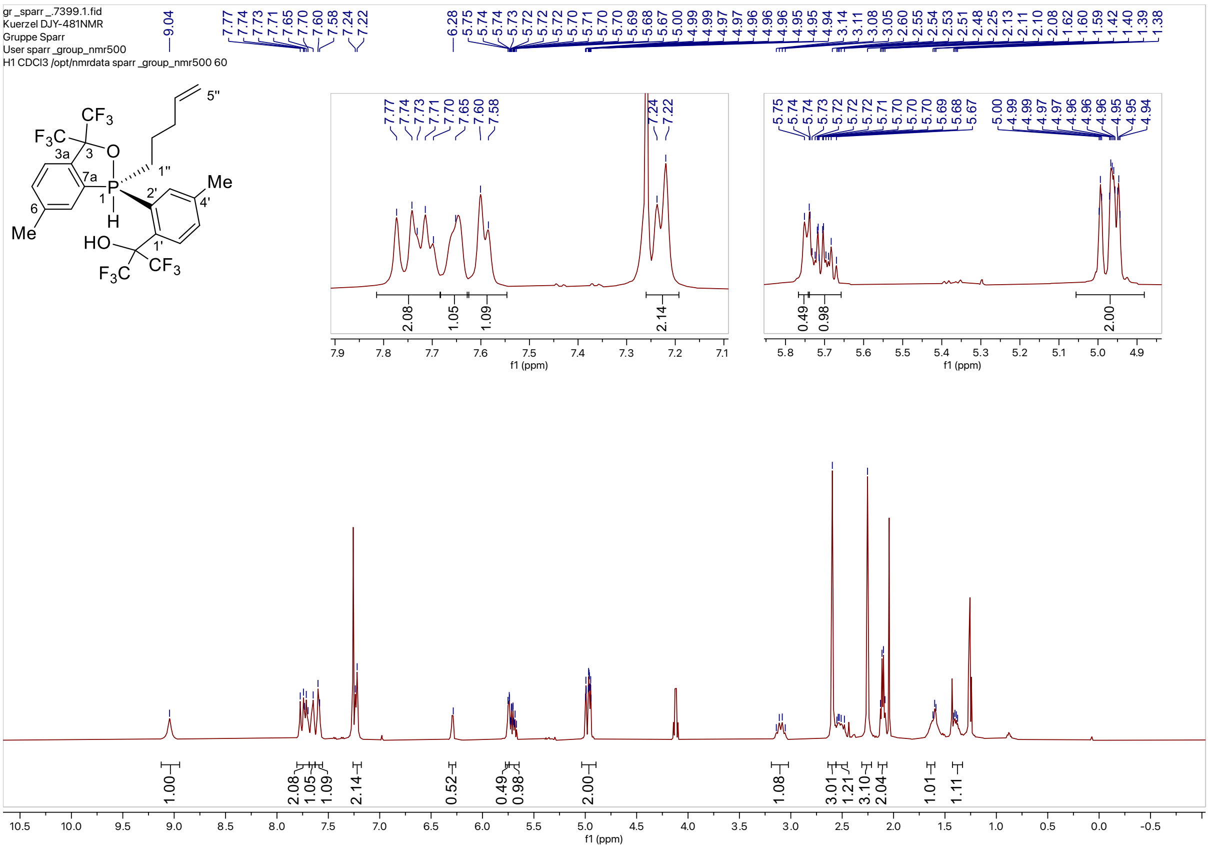

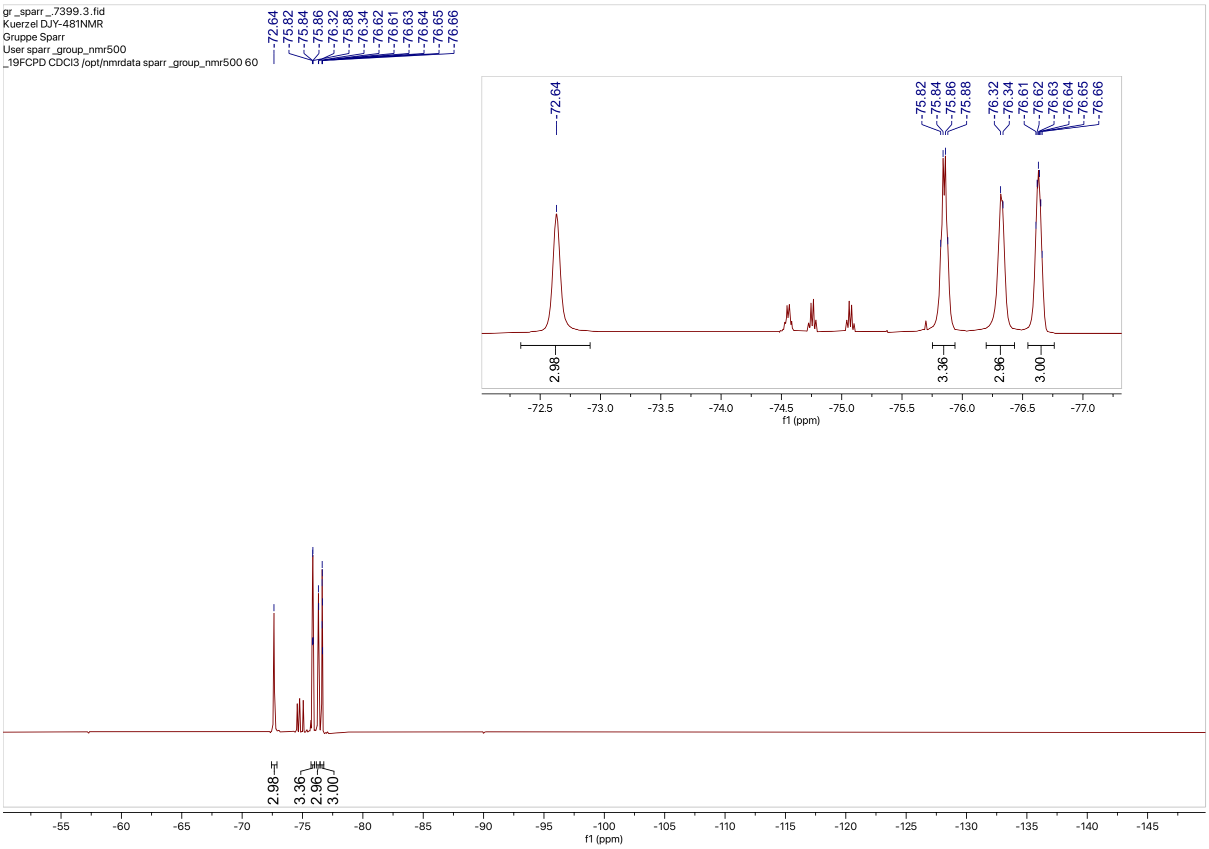

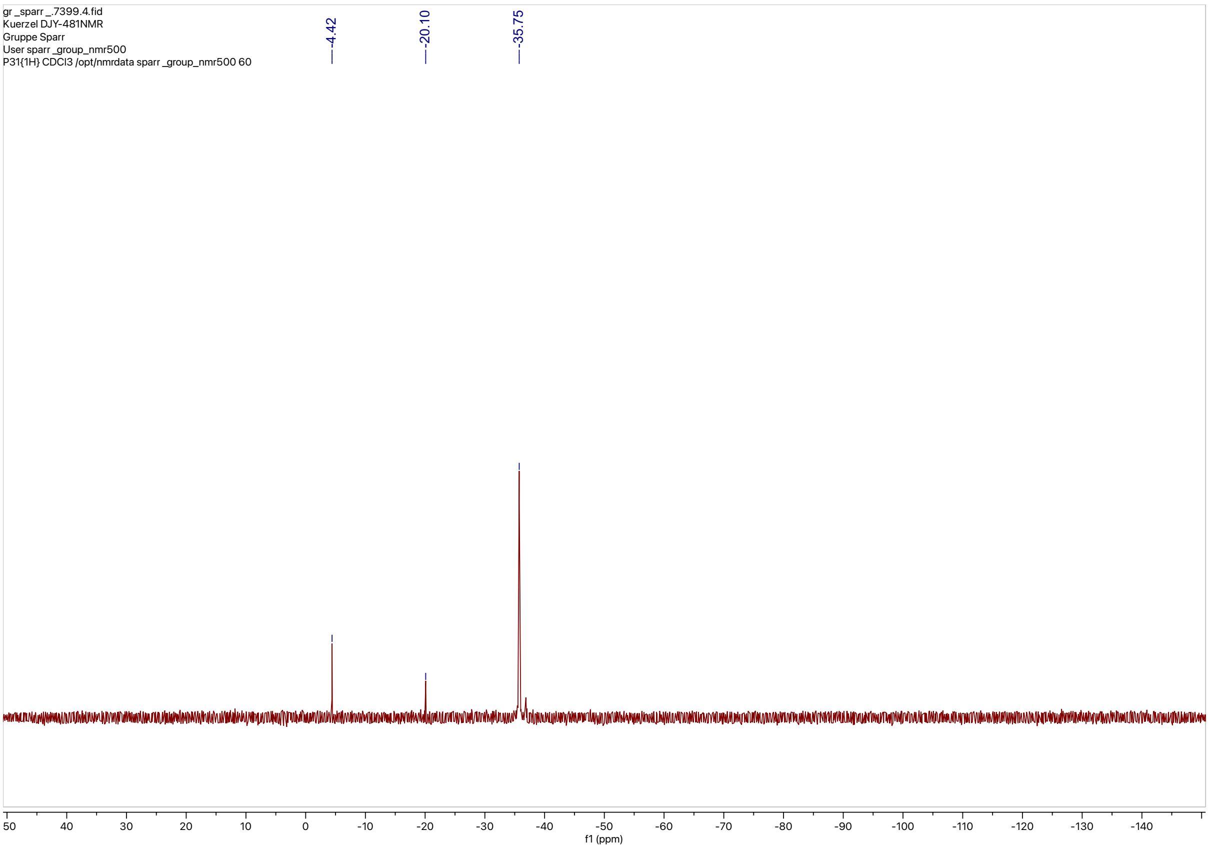

Supplementary Figure 57. ^{1}H (500 MHz, $CDCl_3$, 25 °C), $^{19}F\{^{1}H\}$ (470 MHz, $CDCl_3$, 25 °C) and $^{31}P\{^{1}H\}$ (202 MHz, $CDCl_3$, 25 °C) spectra of **1k**

^{1}H, ^{19}F{^{1}H} and ^{31}P{^{1}H} spectra of [*TBPY*-5-15]-1,1,1,3,3,3-Hexafluoro-2-(4-methyl-2-(1-hydro-6-methyl-1-phenethyl-3,3-bis(trifluorome-thyl)-3*H*-1λ^{5}-benzo[*c*][2,1]oxaphosphol-1-yl)phenyl)propan-2-ol (1l)

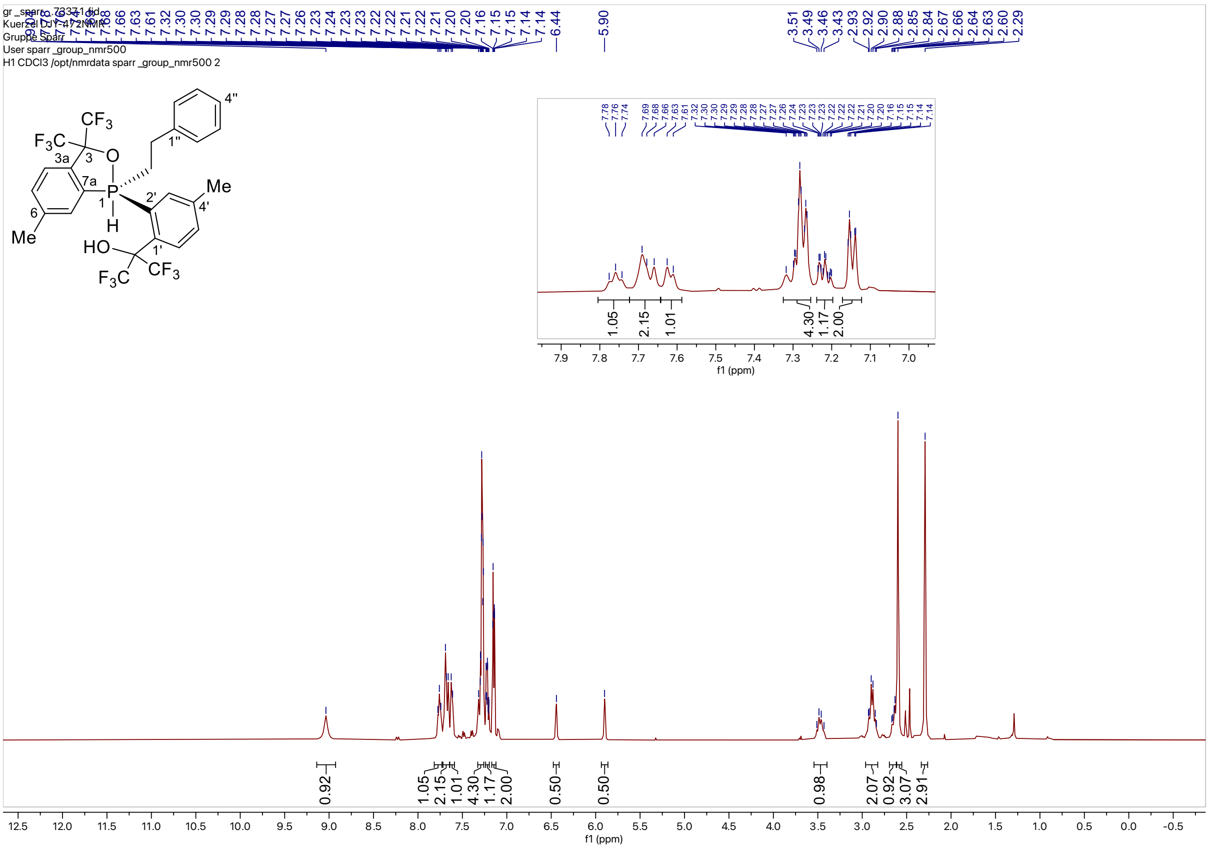

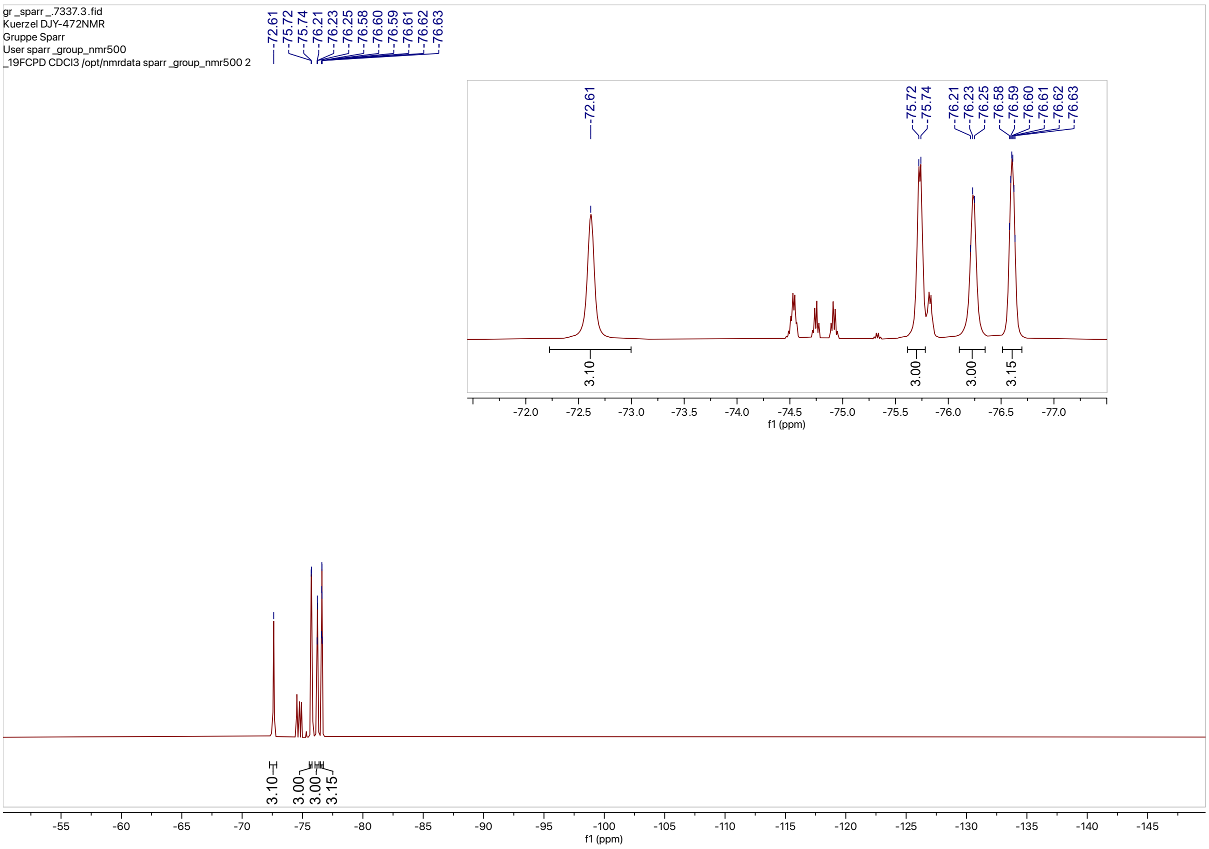

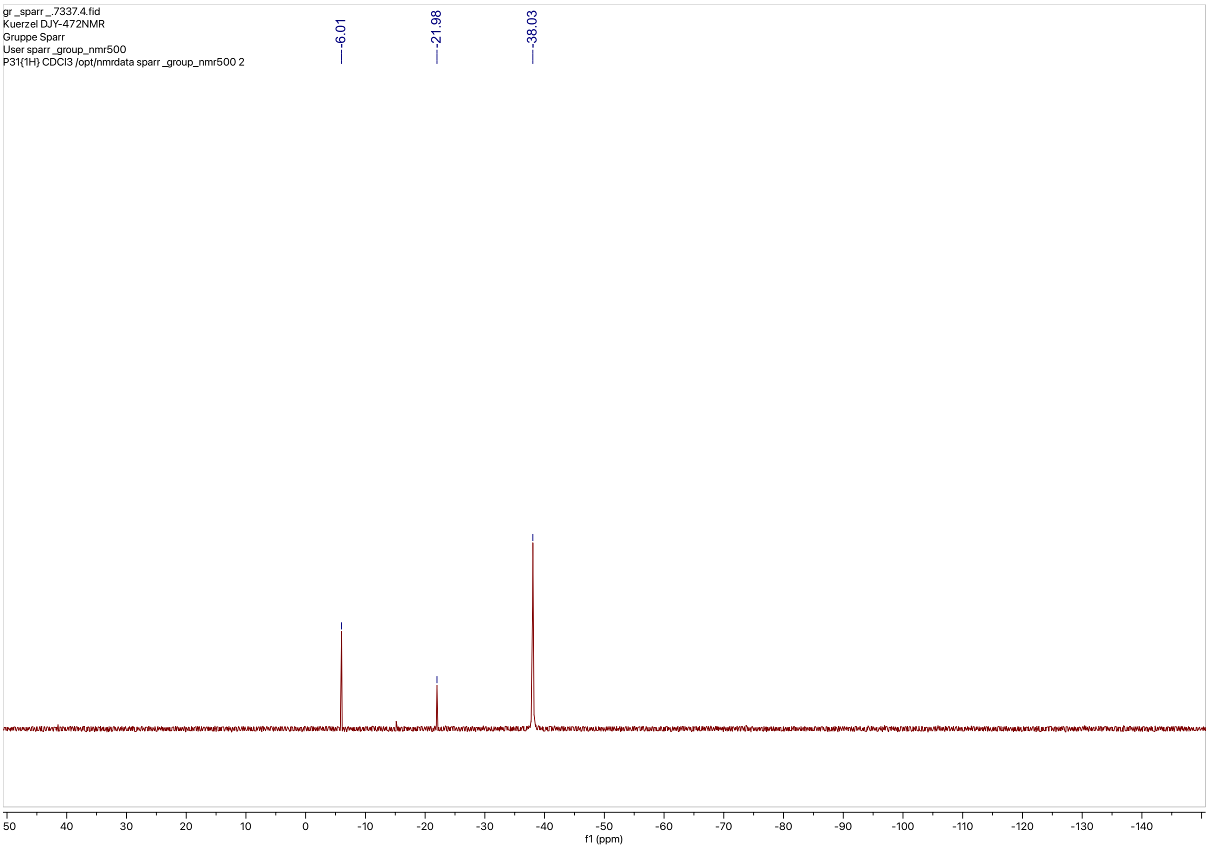

Supplementary Figure 58. ^{1}H (500 MHz, $CDCl_3$, 25 °C), $^{19}F\{^{1}H\}$ (470 MHz, $CDCl_3$, 25 °C) and $^{31}P\{^{1}H\}$ (202 MHz, $CDCl_3$, 25 °C) spectra of **1l**

^{1}H, $^{19}F\{^{1}H\}$ and $^{31}P\{^{1}H\}$ spectra of [*TBPY*-5-15]-1,1,1,3,3,3-Hexafluoro-2-(2-(1,6-dimethyl-1-hydro-3,3-bis(trifluoromethyl)-3*H*-1λ^{5}-benzo[*c*][2,1]oxaphosphol-1-yl)-4-methylphenyl)propan-2-ol (1m)

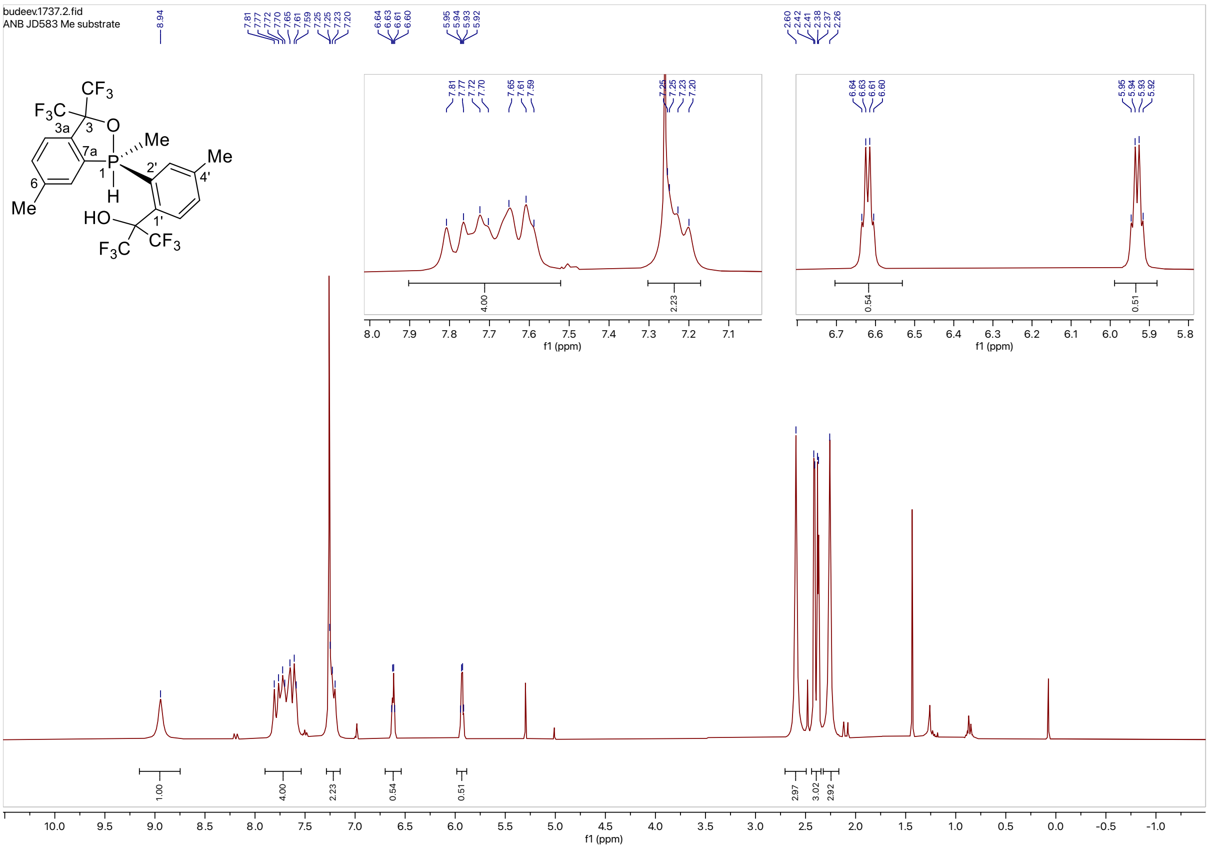

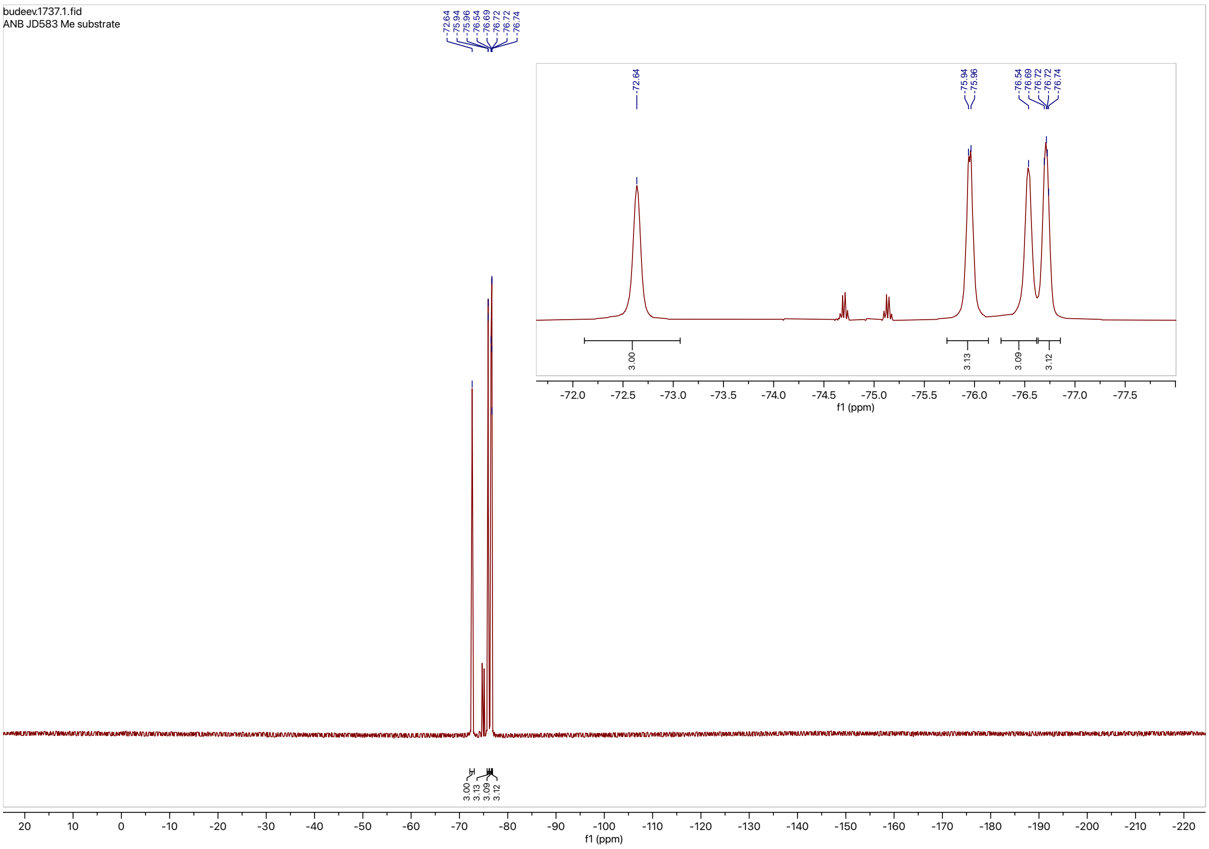

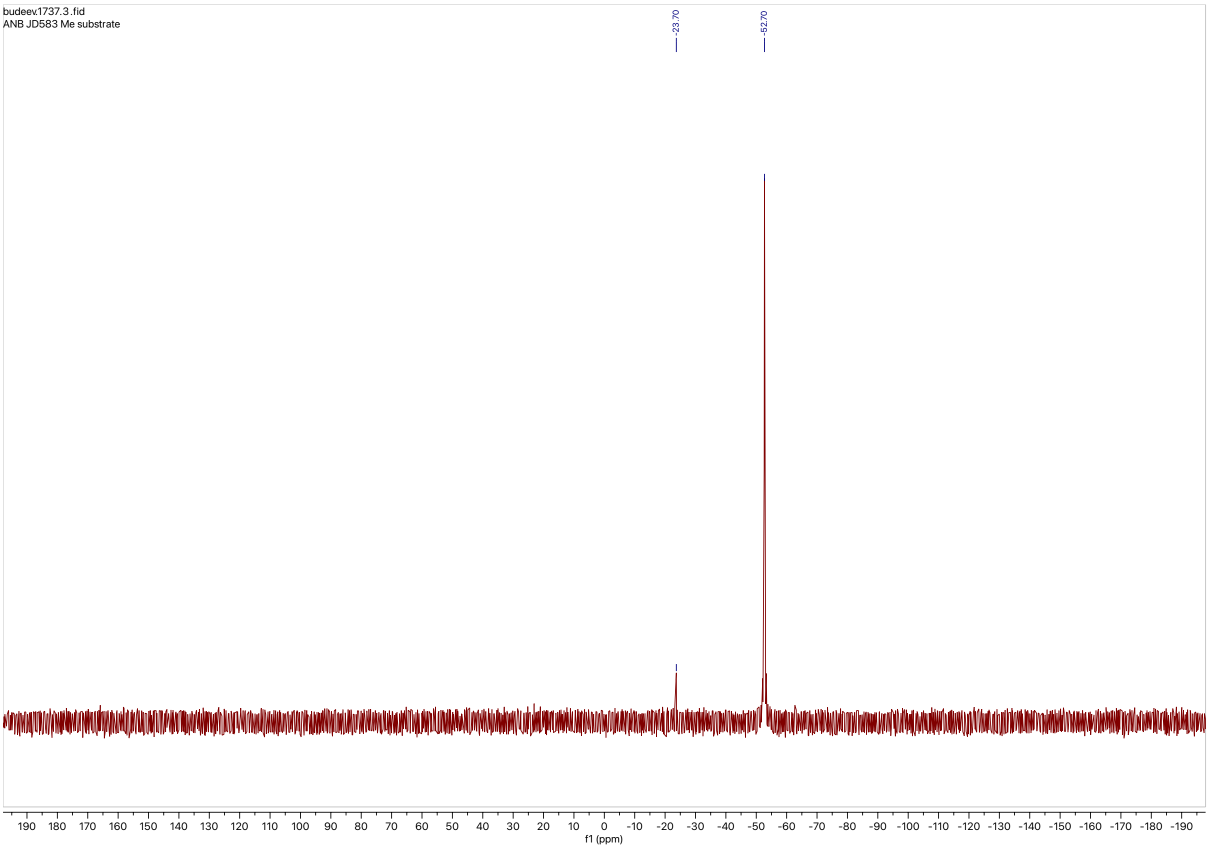

Supplementary Figure 59. ^{1}H (400 MHz, $CDCl_3$, 25 °C), $^{19}F\{^{1}H\}$ (376 MHz, $CDCl_3$, 25 °C) and $^{31}P\{^{1}H\}$ (162 MHz, $CDCl_3$, 25 °C) spectra of **1m**

^{1}H and ^{13}C{^{1}H} NMR spectra of 6-(Trimethylsilyl)hex-5-yn-1-ol

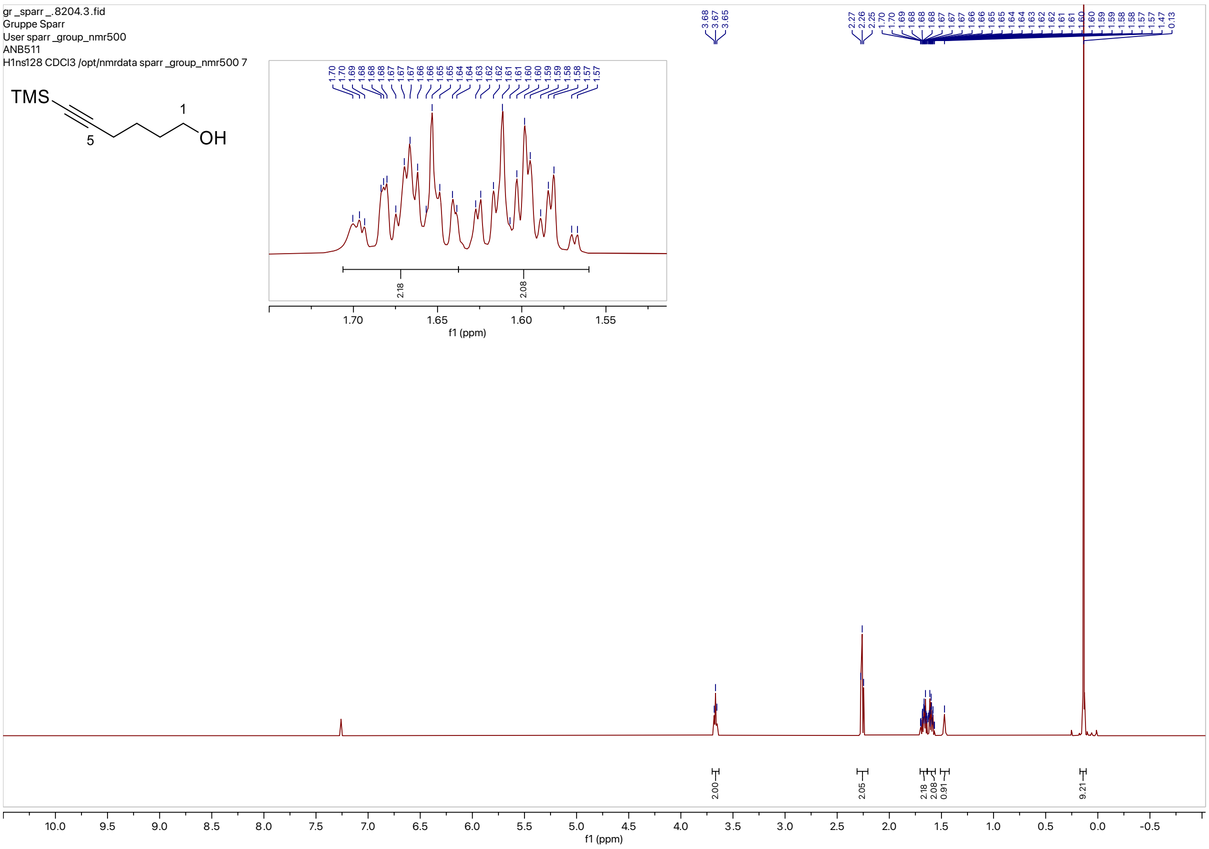

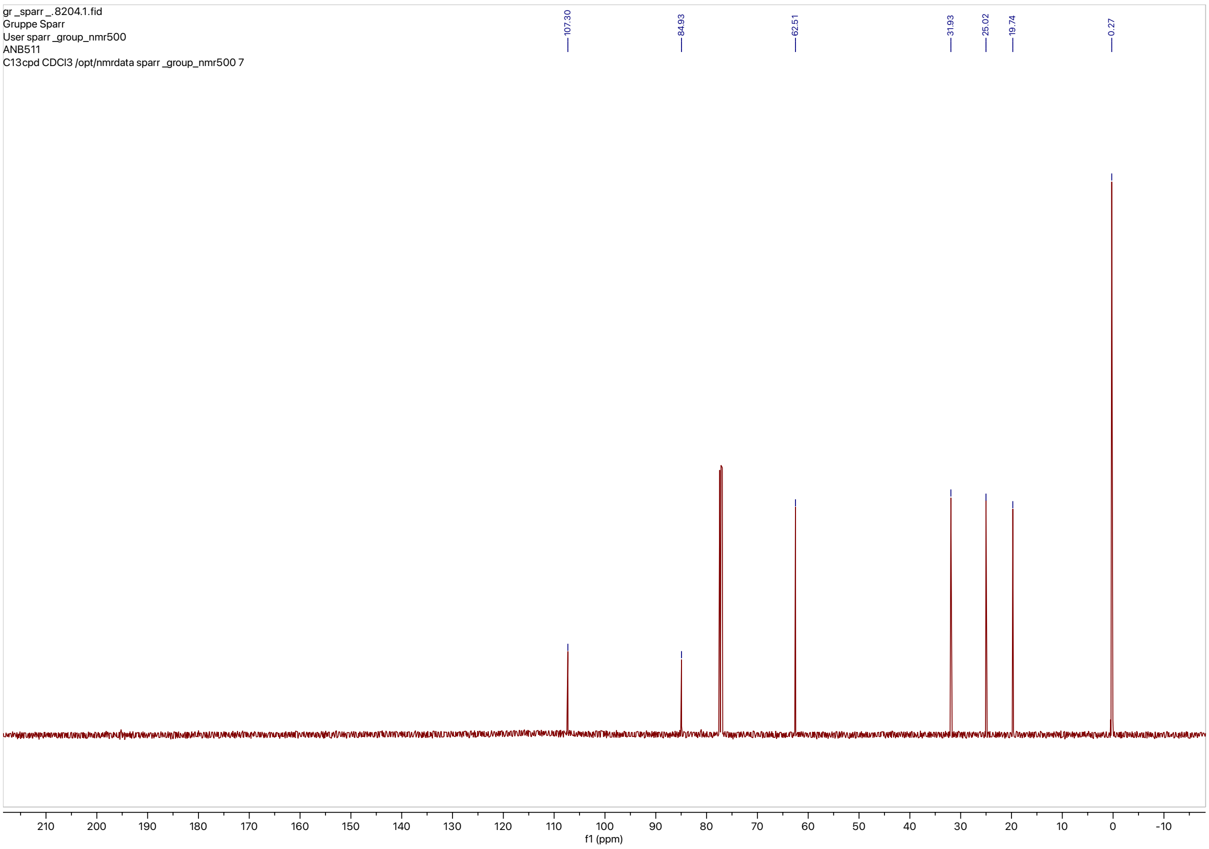

Supplementary Figure 60. ^{1}H (500 MHz, $CDCl_3$, 25 °C) and ^{13}C{^{1}H} (126 MHz, $CDCl_3$, 25 °C) spectra of 6-(trimethylsilyl)hex-5-yn-1-ol

^{1}H and $^{13}C\{^{1}H\}$ NMR spectra of (6-Bromohex-1-yn-1-yl)trimethylsilane

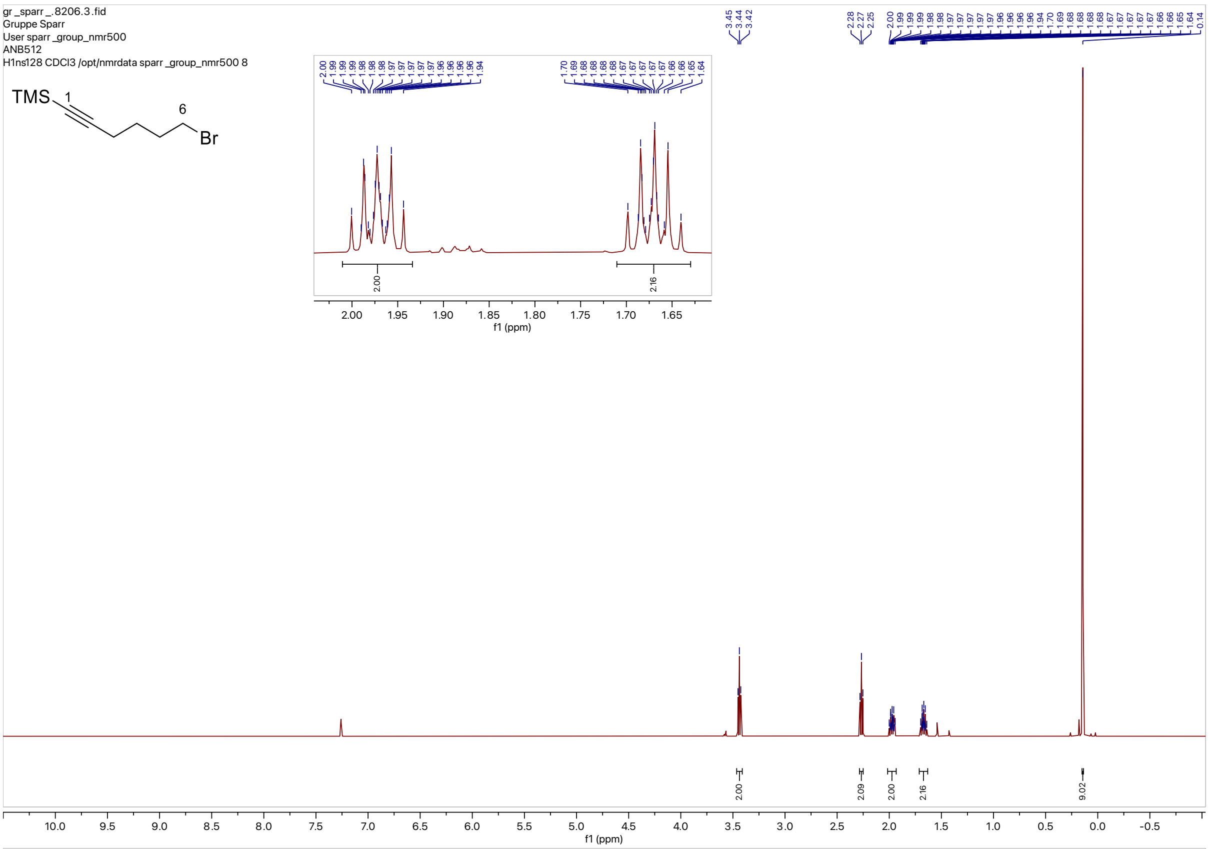

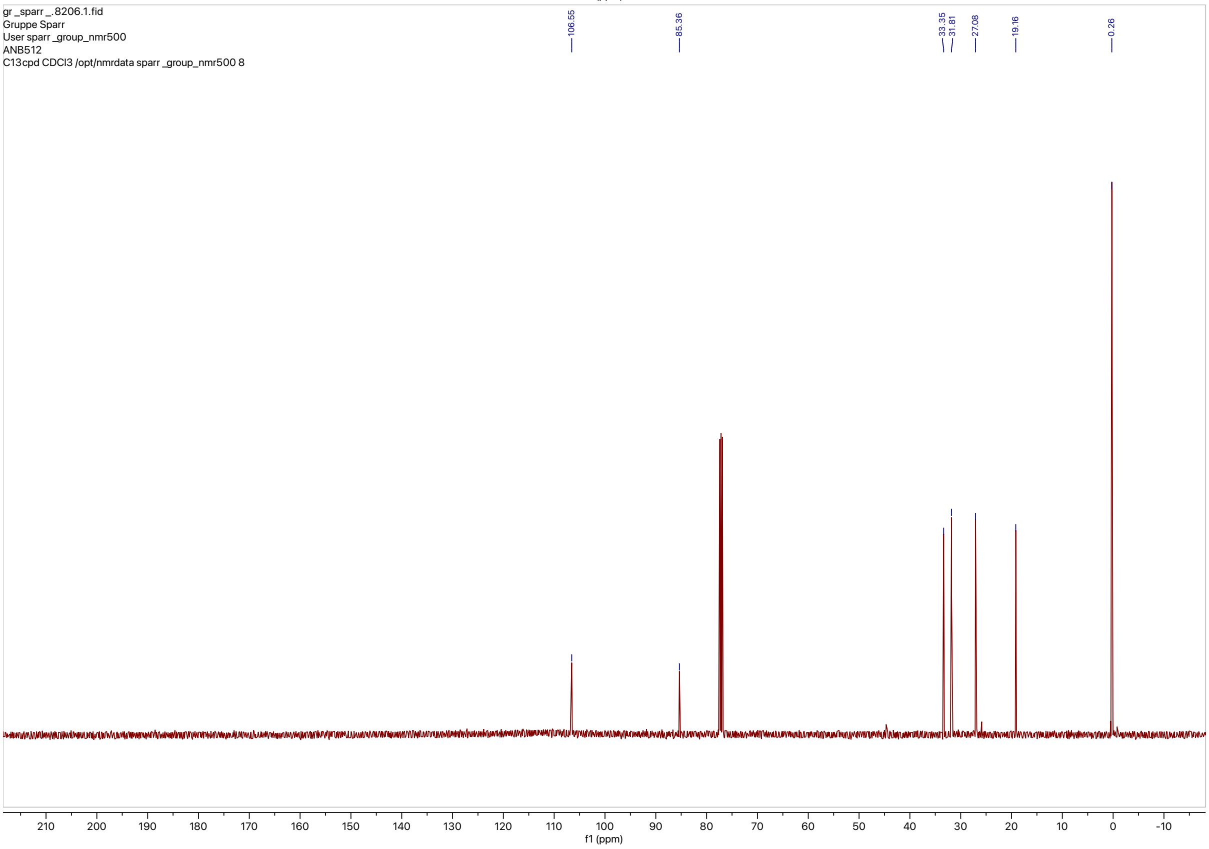

Supplementary Figure 61. ^{1}H (500 MHz, $CDCl_3$, 25 °C) and $^{13}C\{^{1}H\}$ (126 MHz, $CDCl_3$, 25 °C) spectra of (6-bromohex-1-yn-1-yl)trimethylsilane

^{1}H, $^{19}F\{^{1}H\}$, $^{31}P\{^{1}H\}$ NMR spectra of [*TBPY*-5-15]-1,1,1,3,3,3-Hexafluoro-2-(4-methyl-2-(1-hydro-6-methyl-3,3-bis(trifluoromethyl)-1-(6-(trimethylsilyl)hex-5-yn-1-yl)-3*H*-1λ^{5}-benzo[*c*][2,1]oxaphosphol-1-yl)phenyl)propan-2-ol (1n)

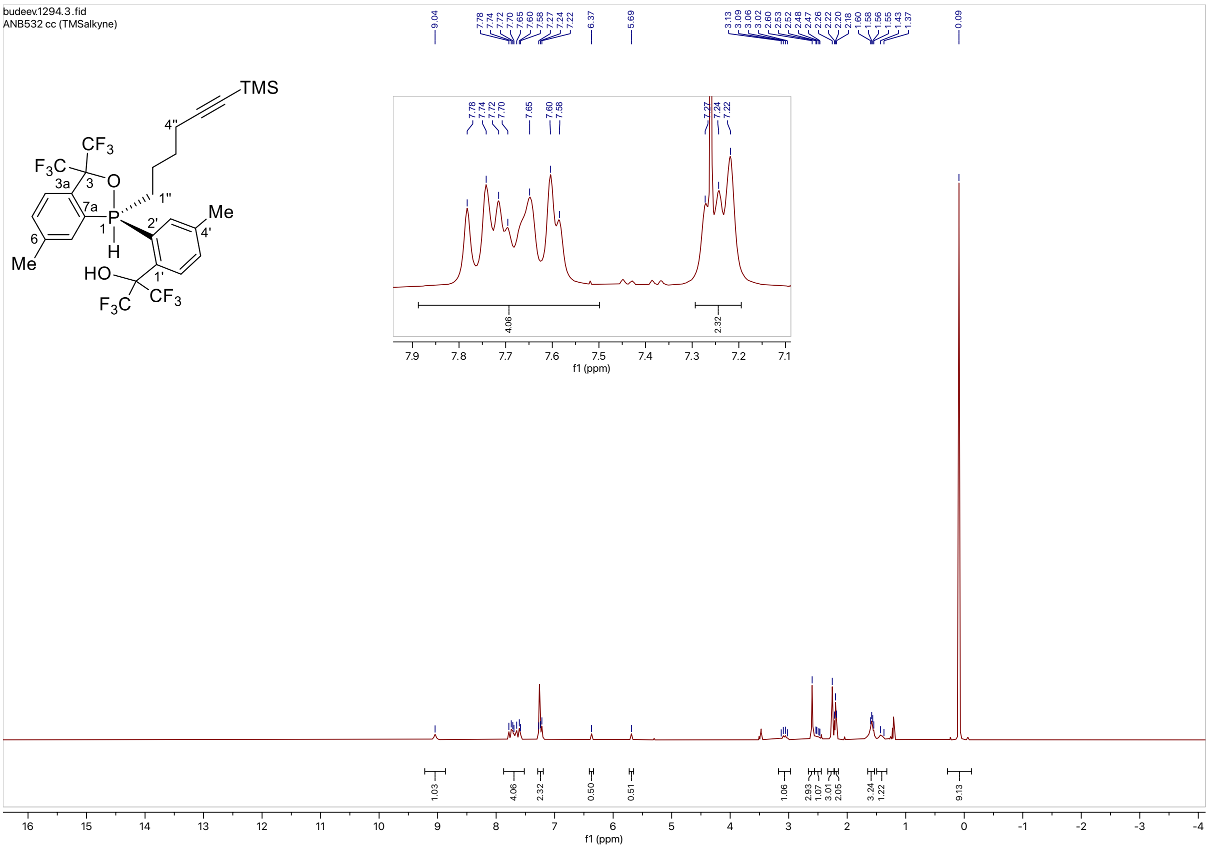

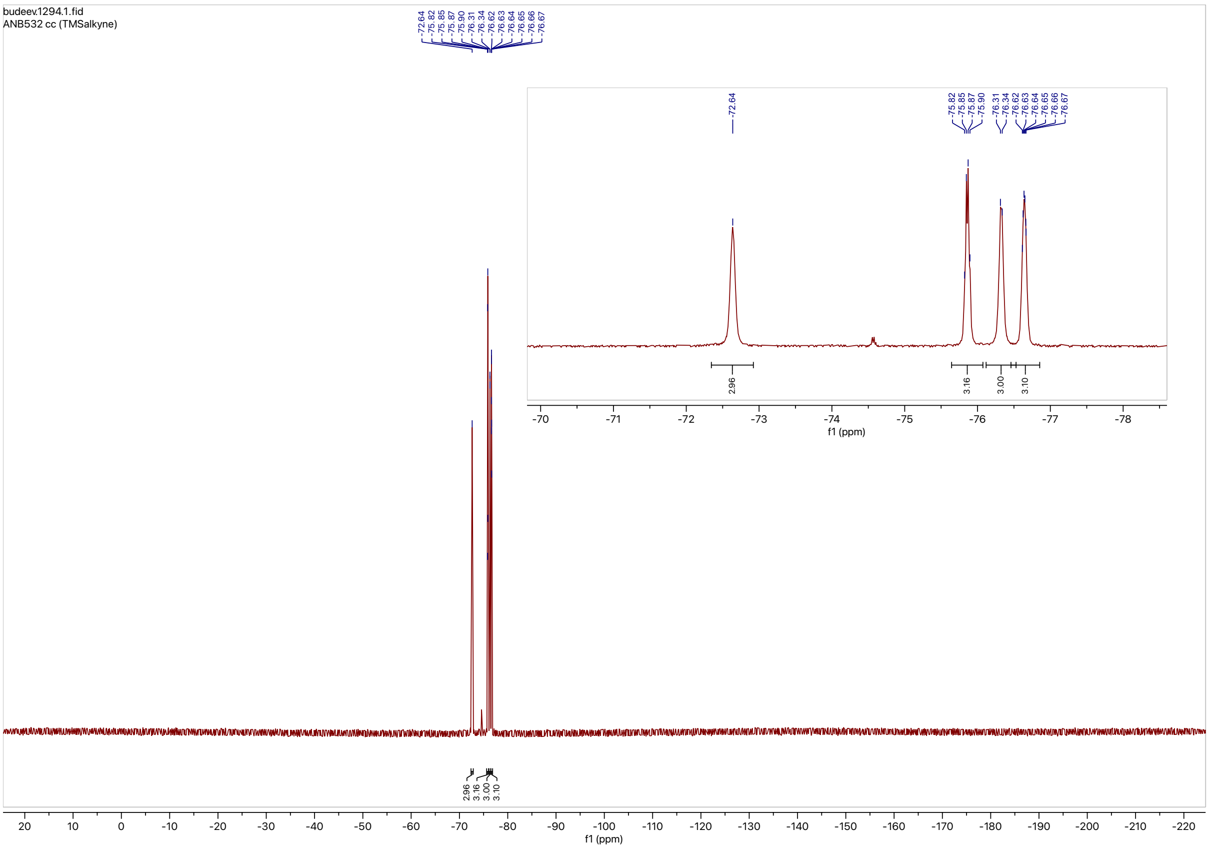

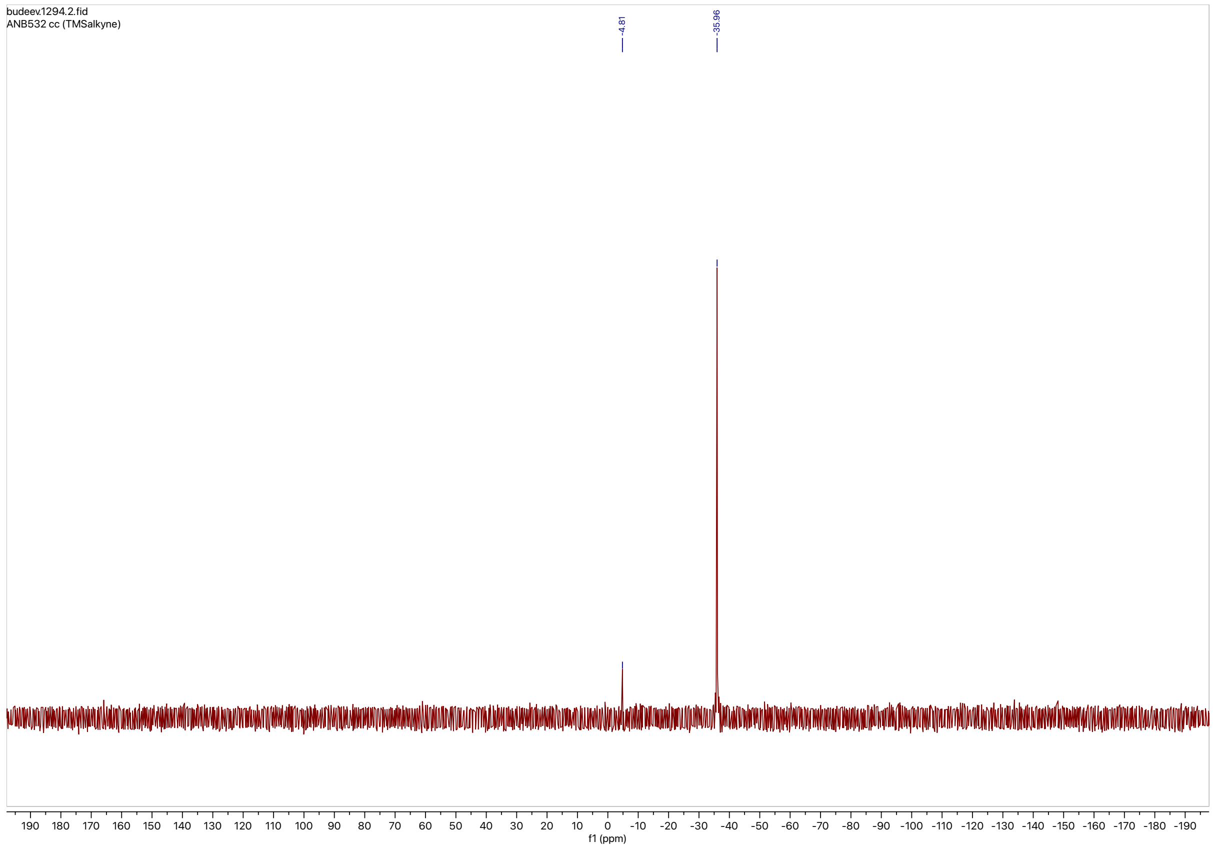

Supplementary Figure 62. ^{1}H (400 MHz, $CDCl_3$, 25 °C), $^{19}F\{^{1}H\}$ (376 MHz, $CDCl_3$, 25 °C) and $^{31}P\{^{1}H\}$ (162 MHz, $CDCl_3$, 25 °C) spectra of **1n**

^{1}H, $^{19}F\{^{1}H\}$ and $^{31}P\{^{1}H\}$ spectra of [*TBPY*-5-15]-1,1,1,3,3,3-Hexafluoro-2-(2-(1-hydro-1-(4-methoxybutyl)-6-methyl-3,3-bis(trifluoromethyl)-3*H*-1λ^{5}-benzo[*c*][2,1]oxaphosphol-1-yl)-4-methylphenyl)propan-2-ol (1o)

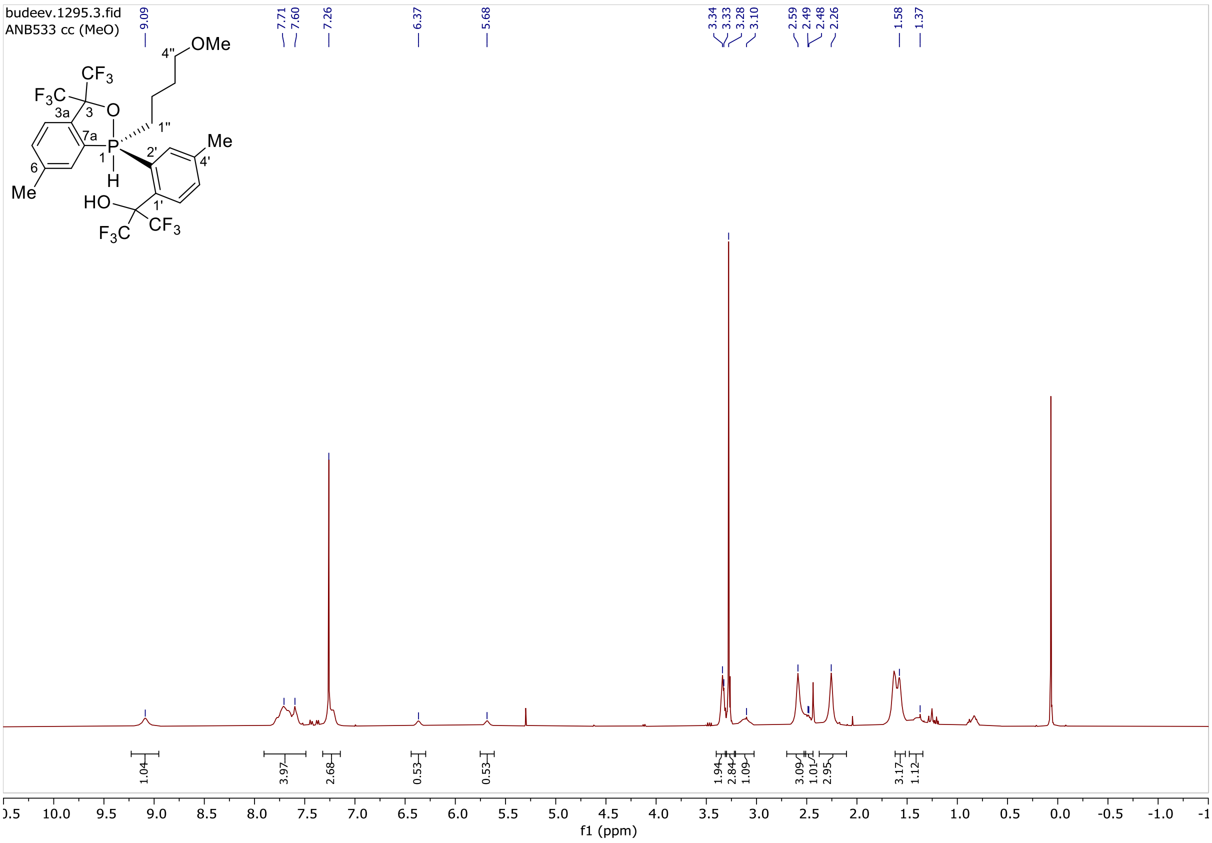

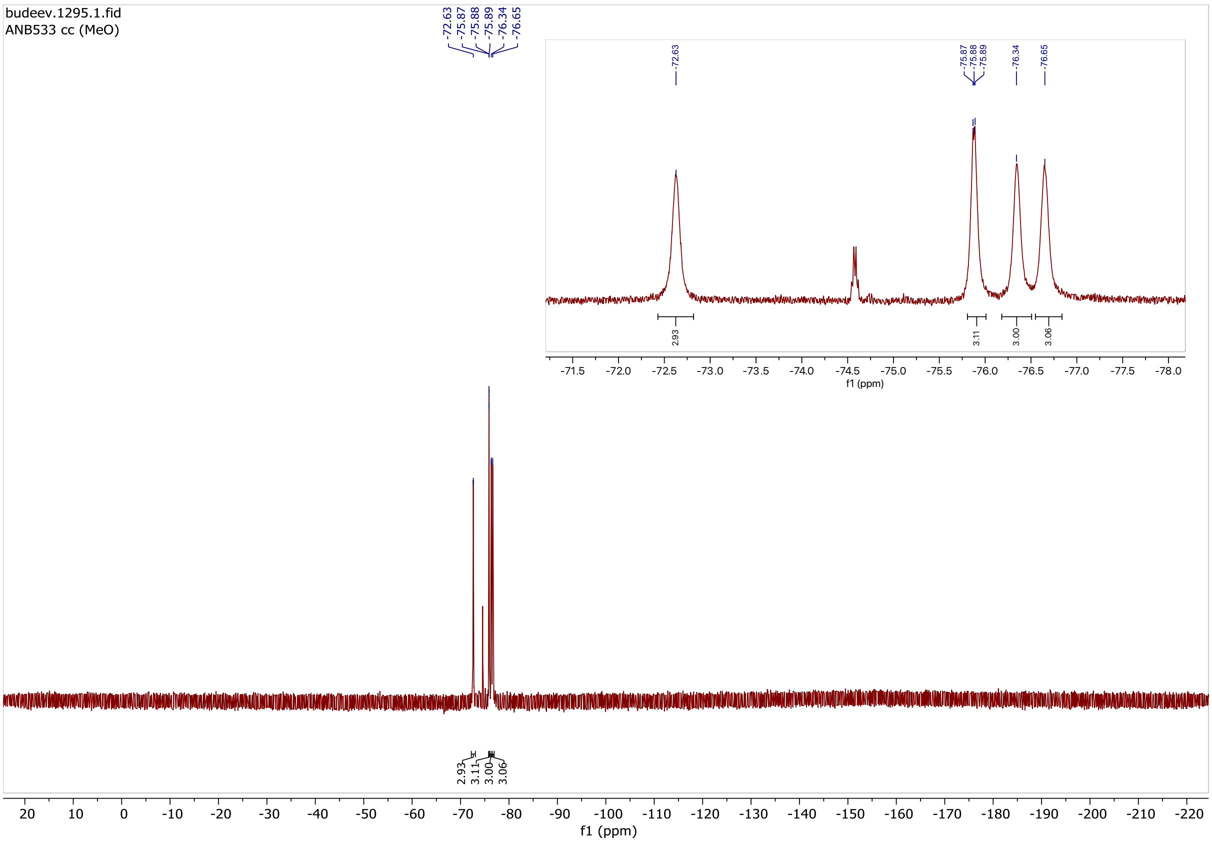

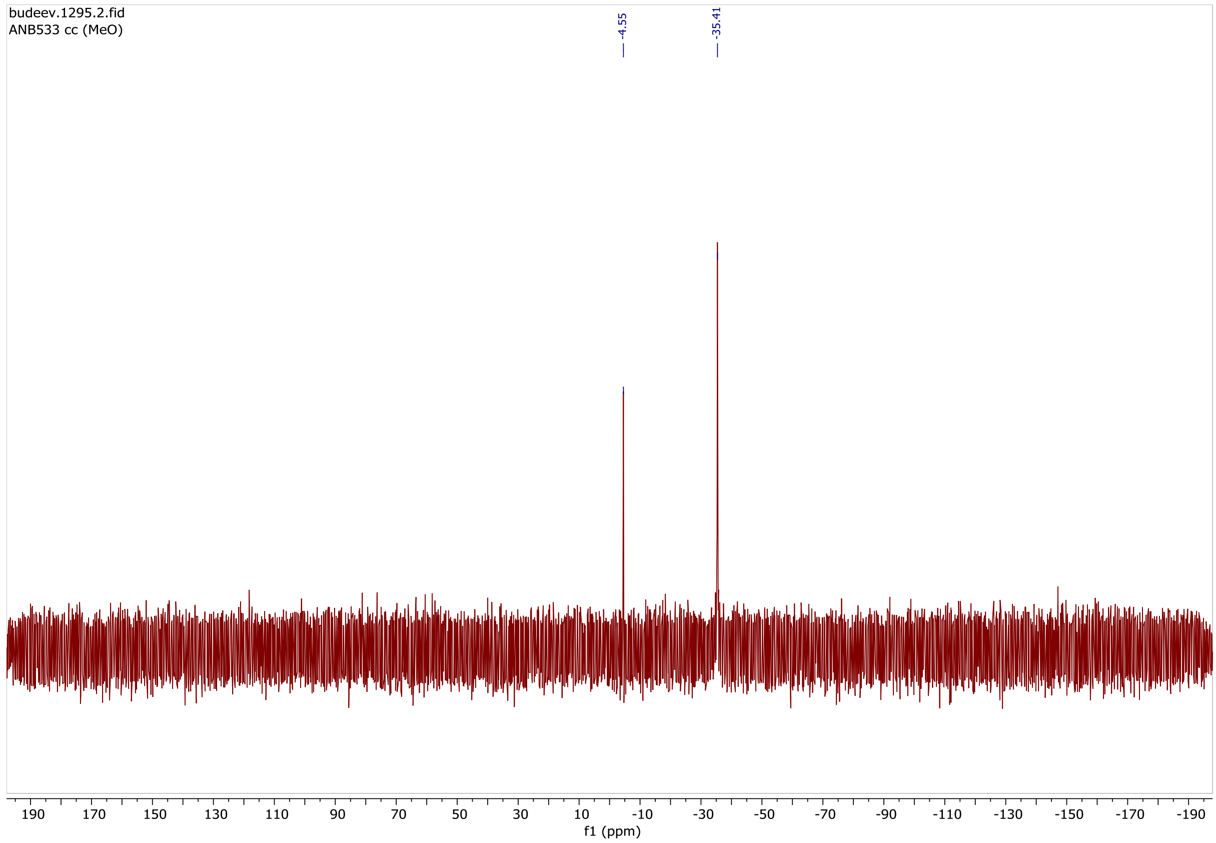

Supplementary Figure 63. ^{1}H (400 MHz, $CDCl_3$, 25 °C), $^{19}F\{^{1}H\}$ (376 MHz, $CDCl_3$, 25 °C) and $^{31}P\{^{1}H\}$ (162 MHz, $CDCl_3$, 25 °C) spectra of **1o**

^{1}H, $^{19}F\{^{1}H\}$ and $^{31}P\{^{1}H\}$ spectra of [*TBPY*-5-15]-1,1,1,3,3,3-Hexafluoro-2-(2-(1-(4-(1,3-dioxolan-2-yl)butyl)- 6-methyl-3,3-bis(trifluoromethyl)-3*H*-1λ^{5}-benzo[*c*][2,1]oxaphosphol-1-yl)-4-methylphenyl)propan-2-ol (1p)

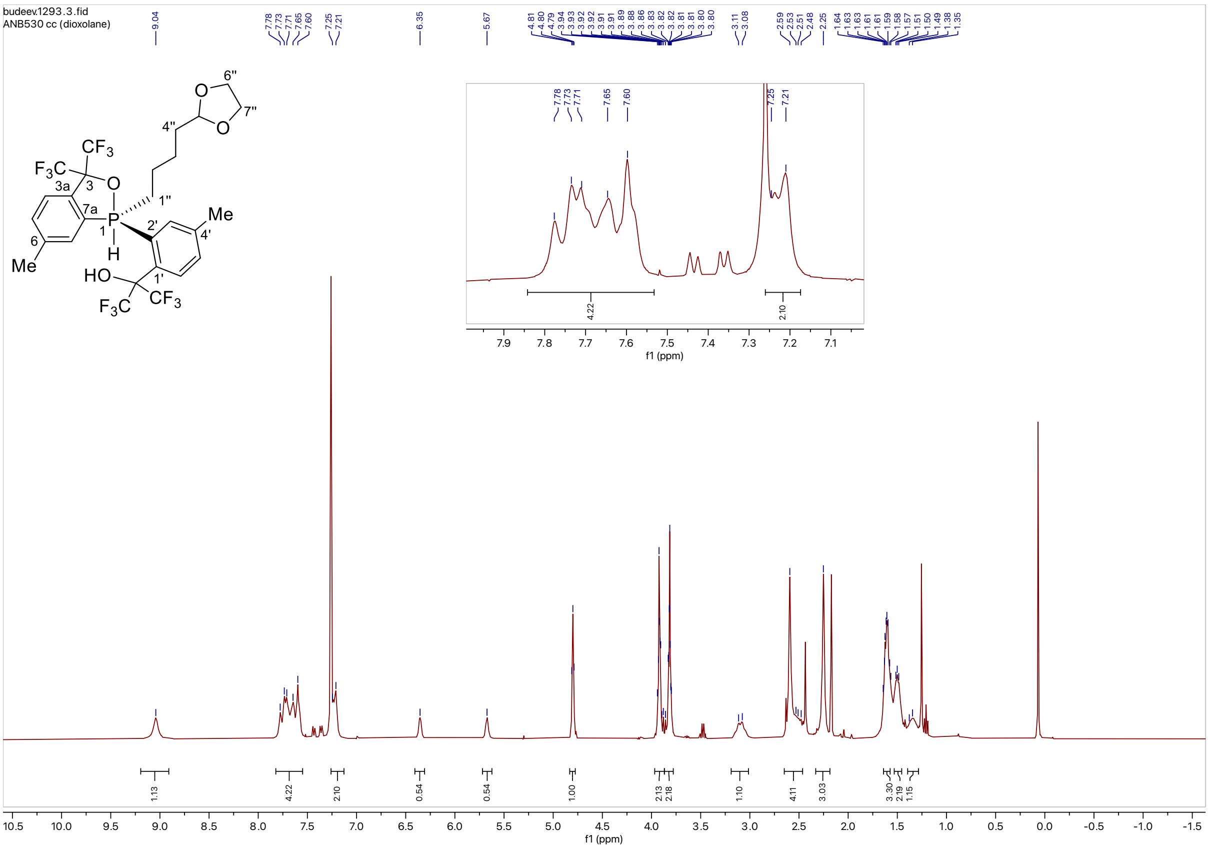

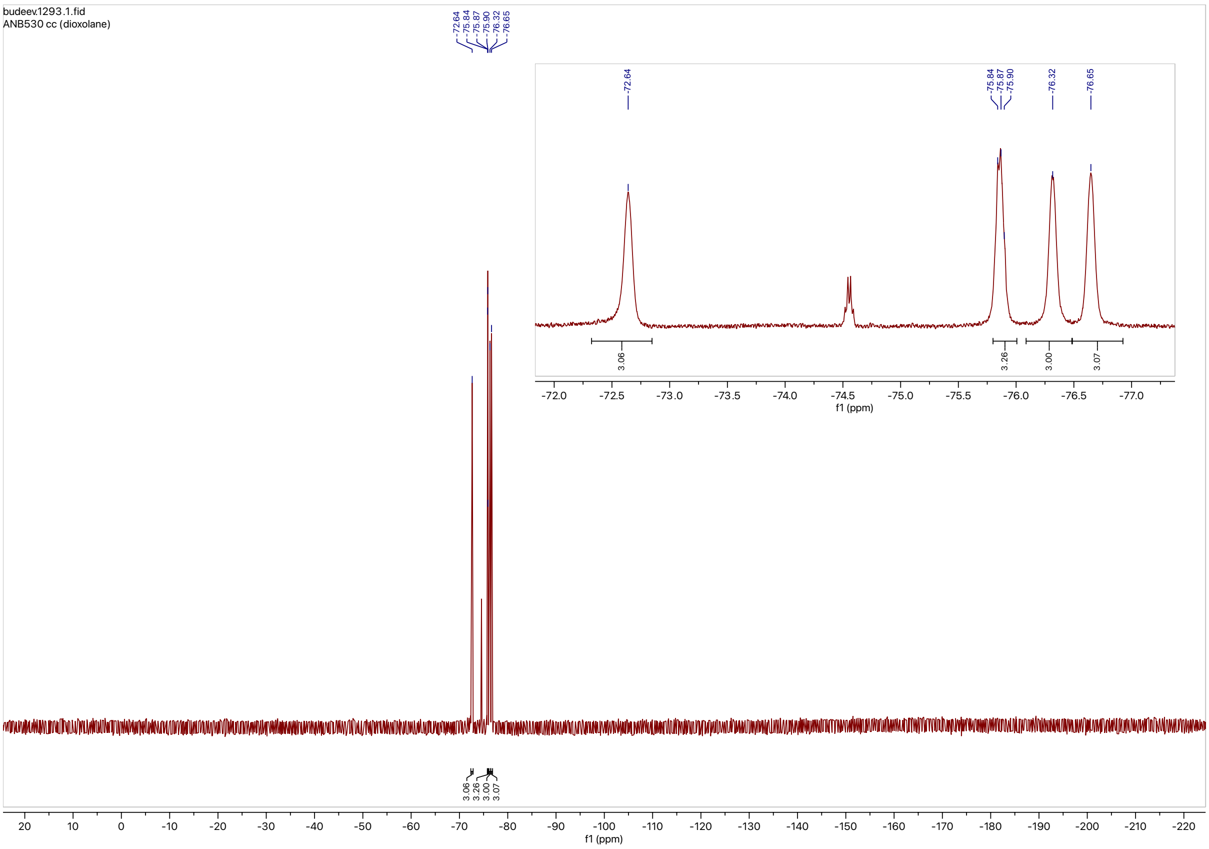

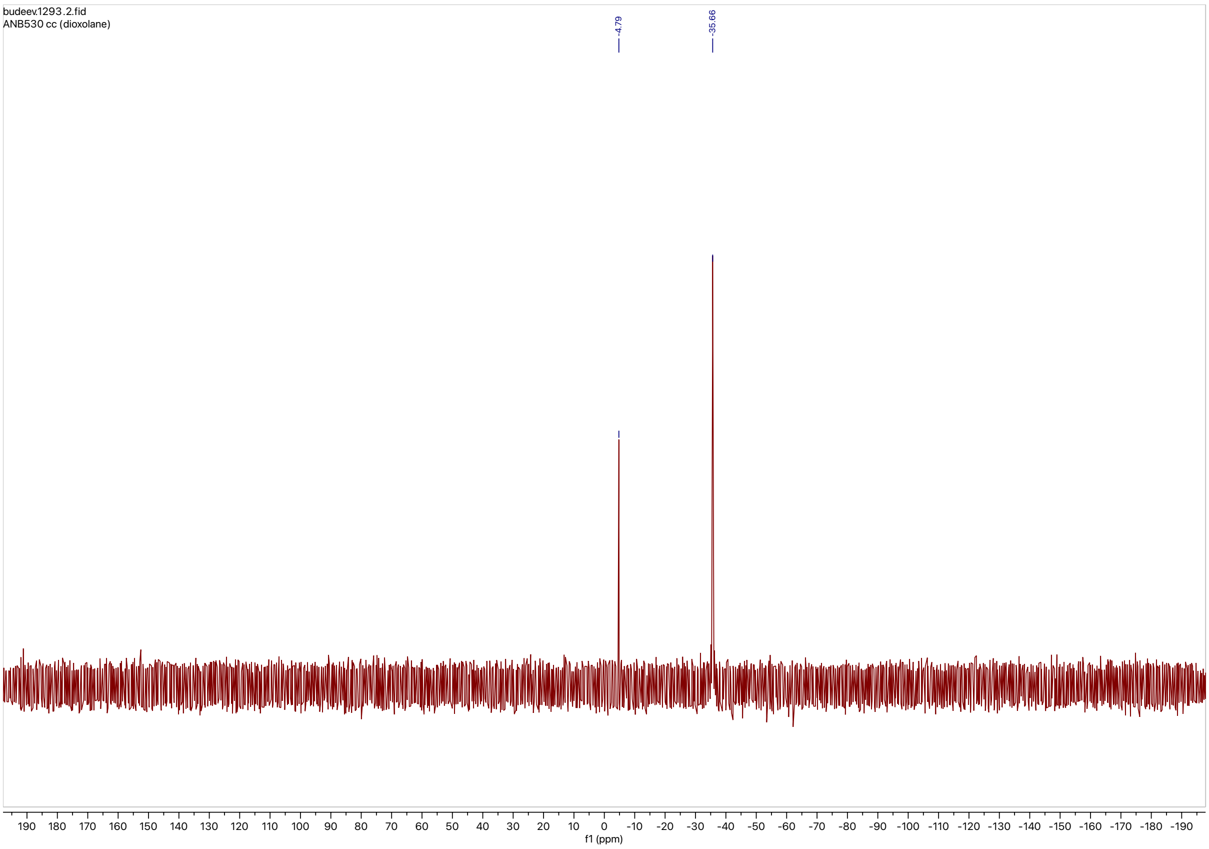

Supplementary Figure 64. ^{1}H (400 MHz, $CDCl_3$, 25 °C), $^{19}F\{^{1}H\}$ (376 MHz, $CDCl_3$, 25 °C) and $^{31}P\{^{1}H\}$ (162 MHz, $CDCl_3$, 25 °C) spectra of **1p**

^{1}H, $^{13}C\{^{1}H\}$ and $^{19}F\{^{1}H\}$ NMR spectra of 1-((1*R*,2*R*)-2-Azidocyclohexyl)-3-(2- (trifluoromethyl)phenyl)thiourea (S7)

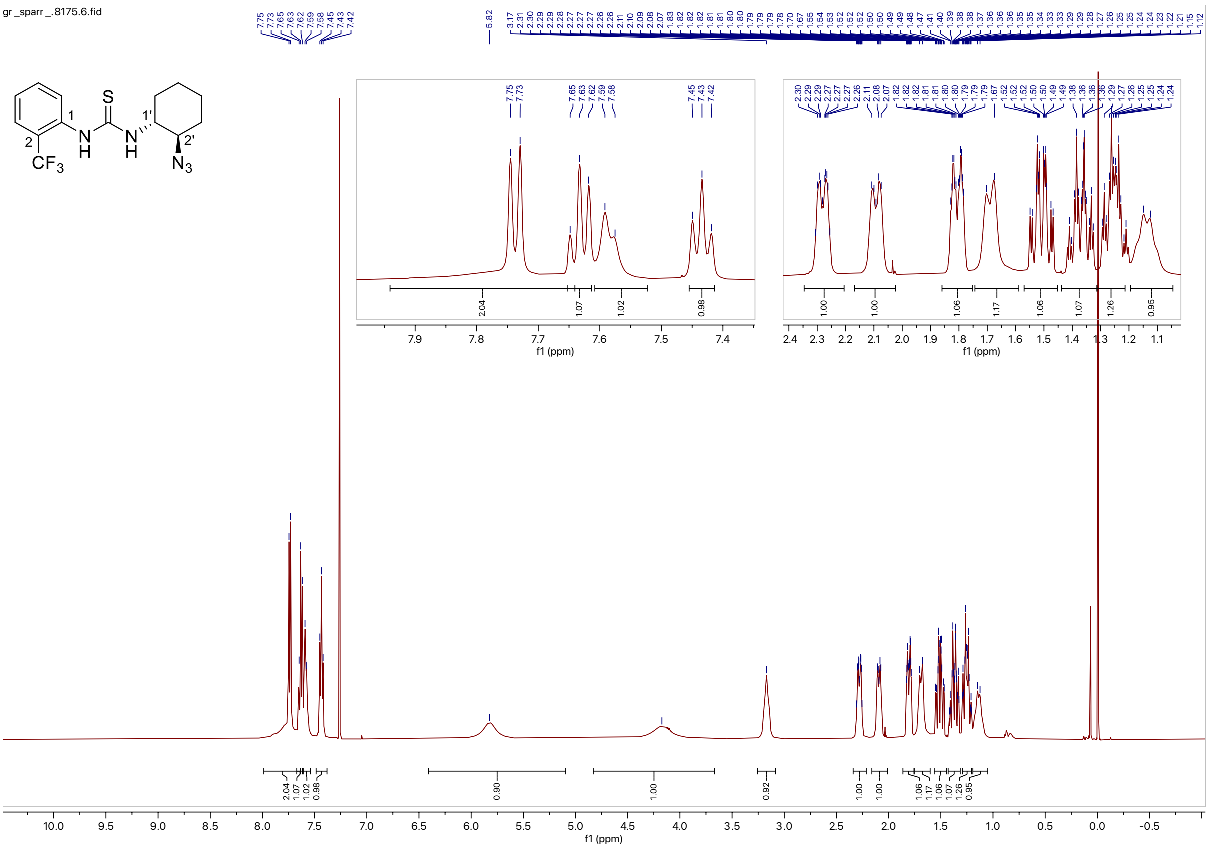

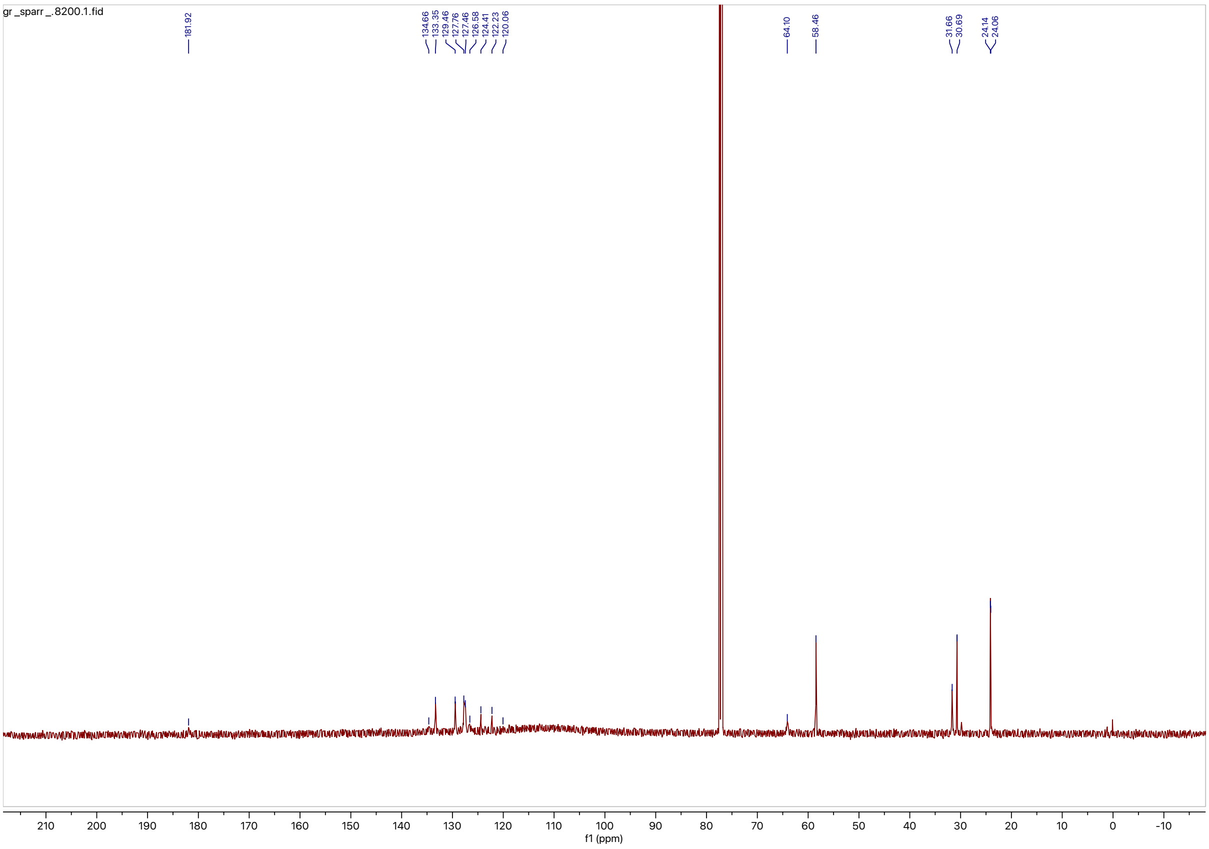

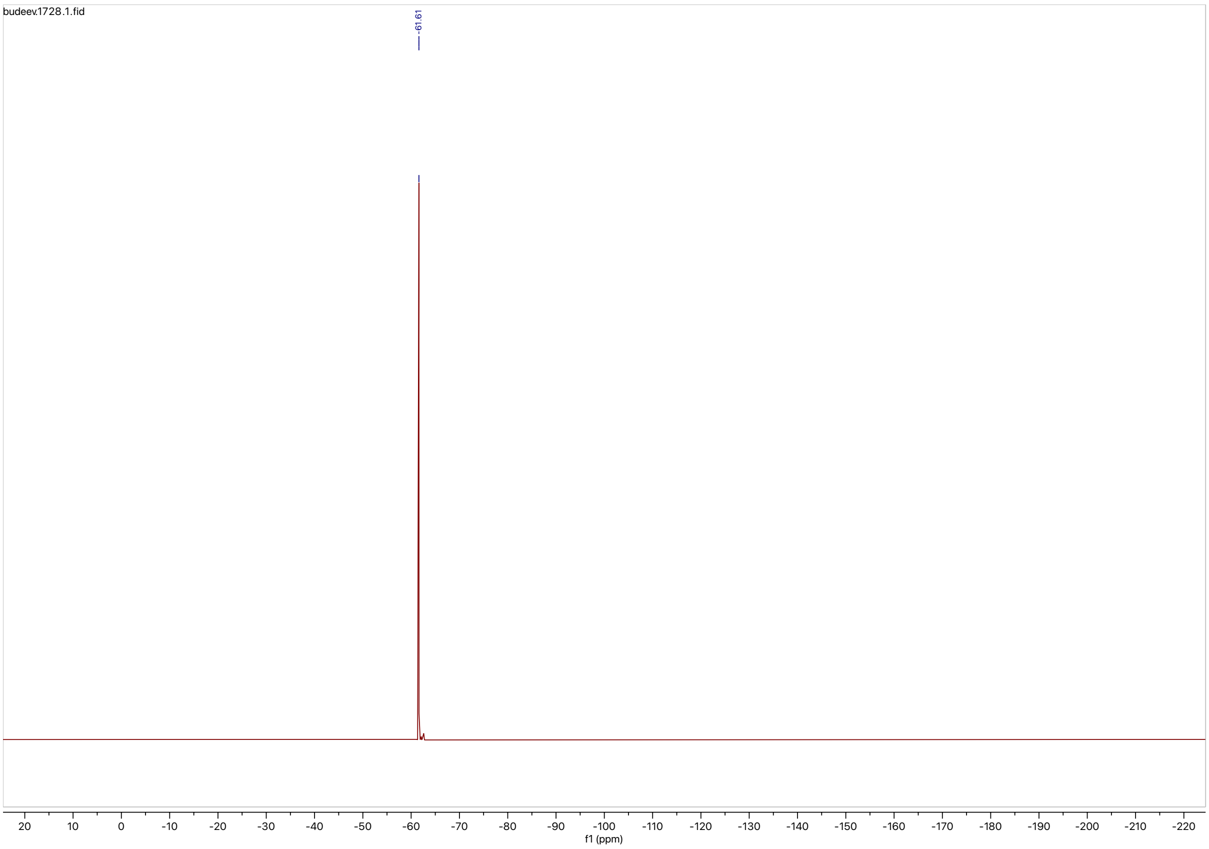

Supplementary Figure 65. ^{1}H (500 MHz, $CDCl_3$, 25 °C), $^{13}C\{^{1}H\}$ (126 MHz, $CDCl_3$, 25 °C) and $^{19}F\{^{1}H\}$ (376 MHz, $CDCl_3$, 25 °C) spectra of **S7**

^{1}H, ^{13}C{^{1}H}, ^{19}F{^{1}H} and ^{31}P{^{1}H} NMR spectra of 1-((1*R*,2*R*)-2-((Triethyl-λ^{5}-phosphaneylidene)amino)cyclohexyl)-3-(2-(trifluoromethyl)phenyl)thiourea (C8)

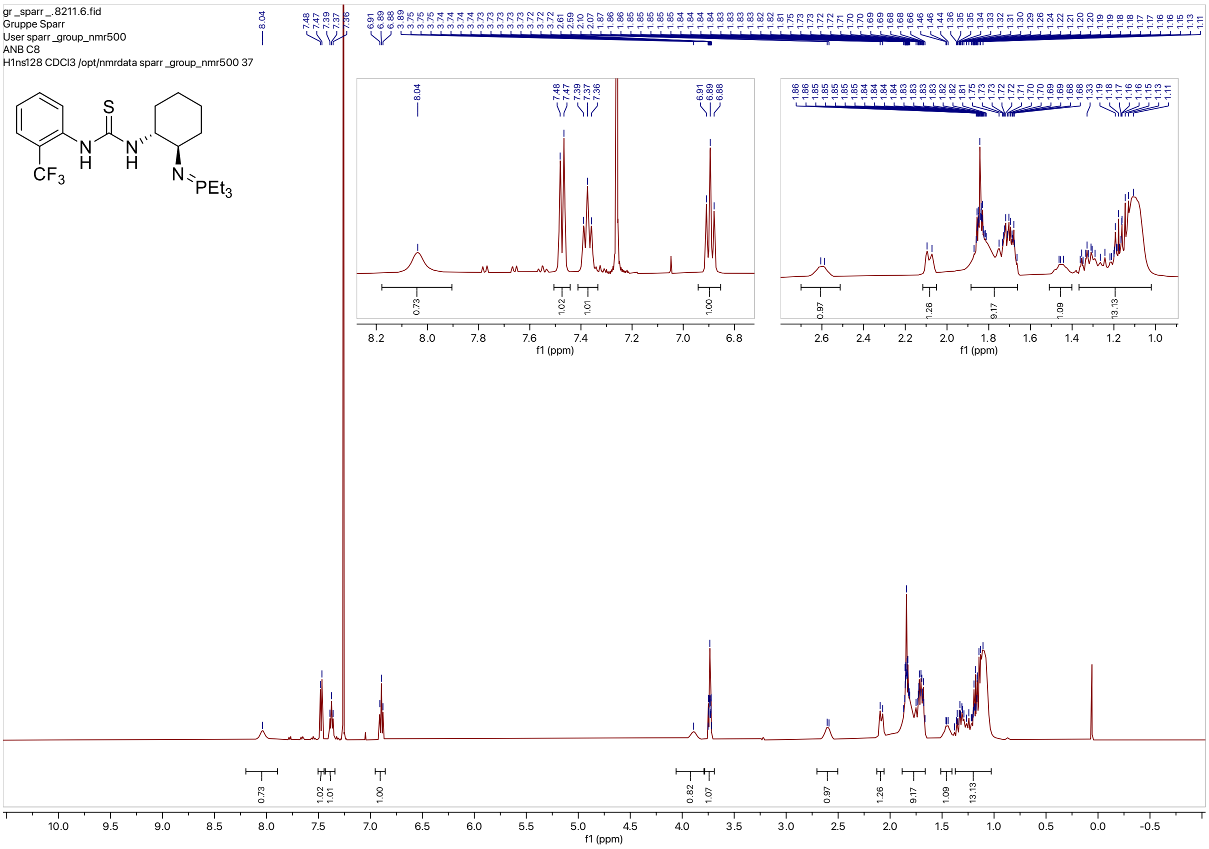

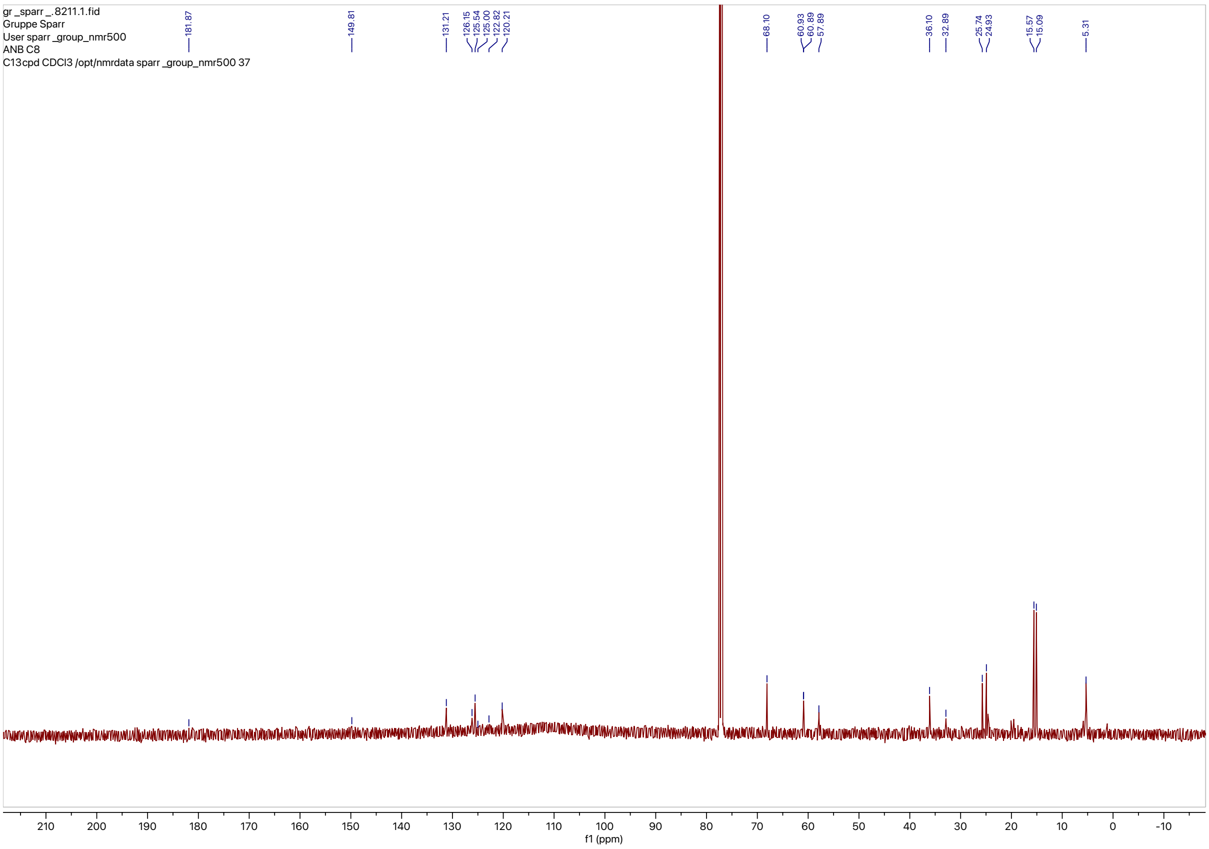

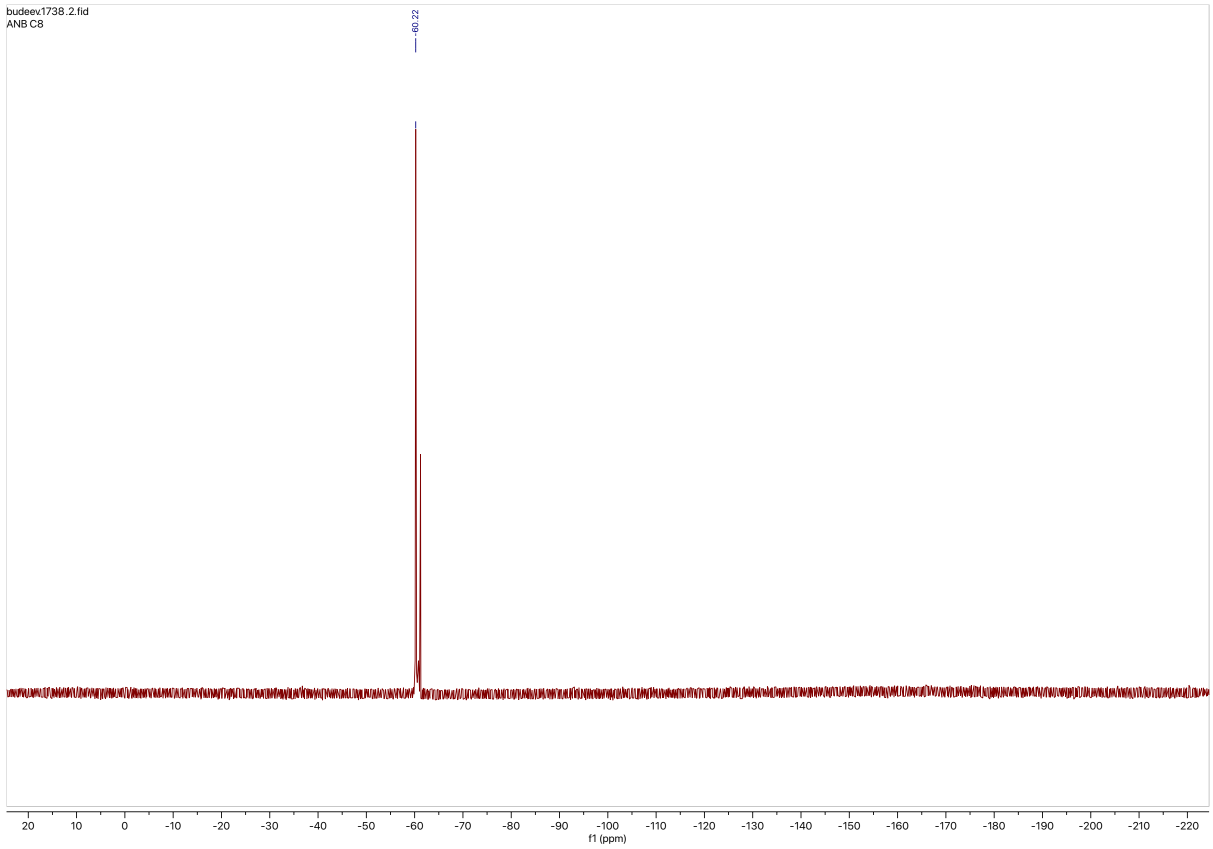

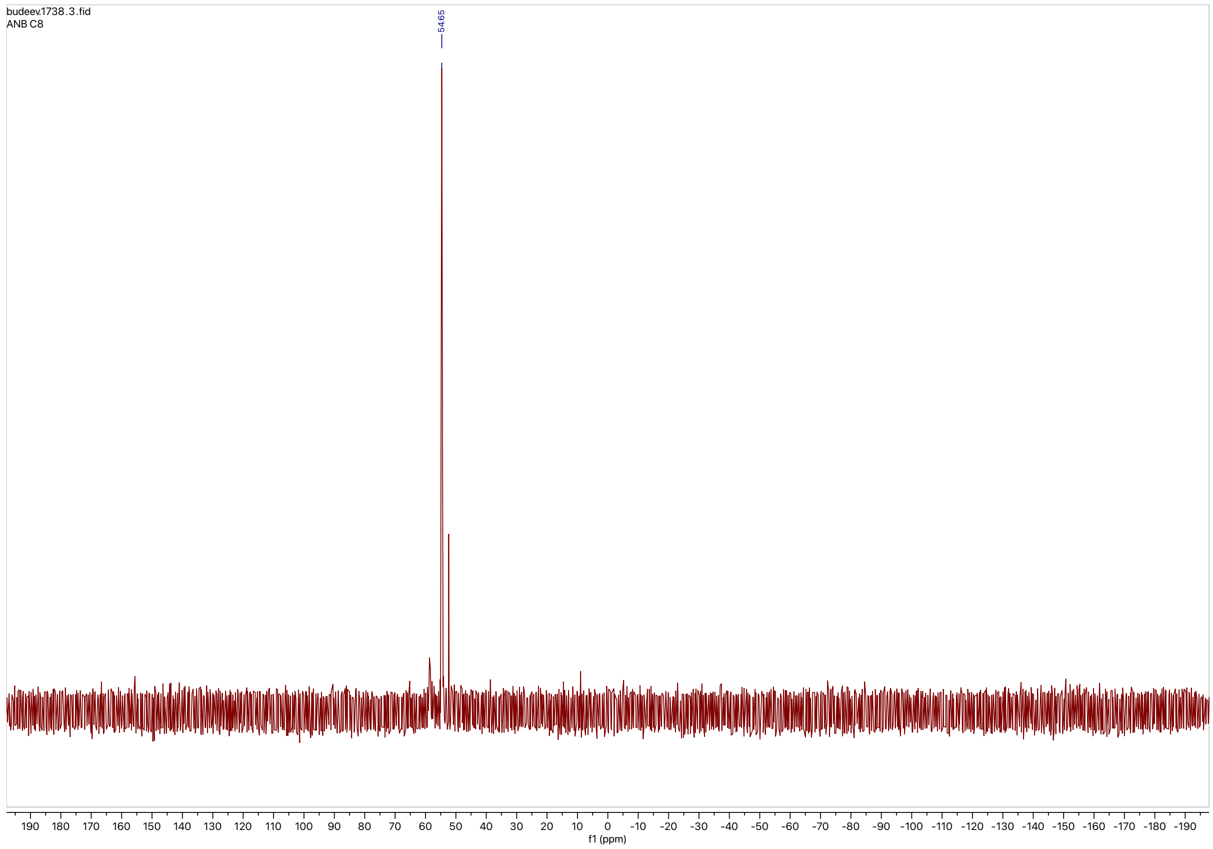

Supplementary Figure 66. ^{1}H (500 MHz, $CDCl_3$, 25 °C), $^{13}C\{^{1}H\}$ (126 MHz, $CDCl_3$, 25 °C), $^{19}F\{^{1}H\}$ (376 MHz, $CDCl_3$, 25 °C) and $^{31}P\{^{1}H\}$ (162 MHz, $CDCl_3$, 25 °C) spectra of **C8**

^{1}H, ^{13}C{^{1}H}, ^{19}F{^{1}H} and ^{31}P{^{1}H} spectra of [*TBPY*-5-11'-*A*]-1-Butyl-6,6'-dimethyl-3,3,3',3'-tetrakis(trifluoromethyl)-3*H*,3'*H*-1λ^5,1'-spirobi[benzo[*c*][2,1]oxaphosphole] ((*S*-*trans*)-2a)

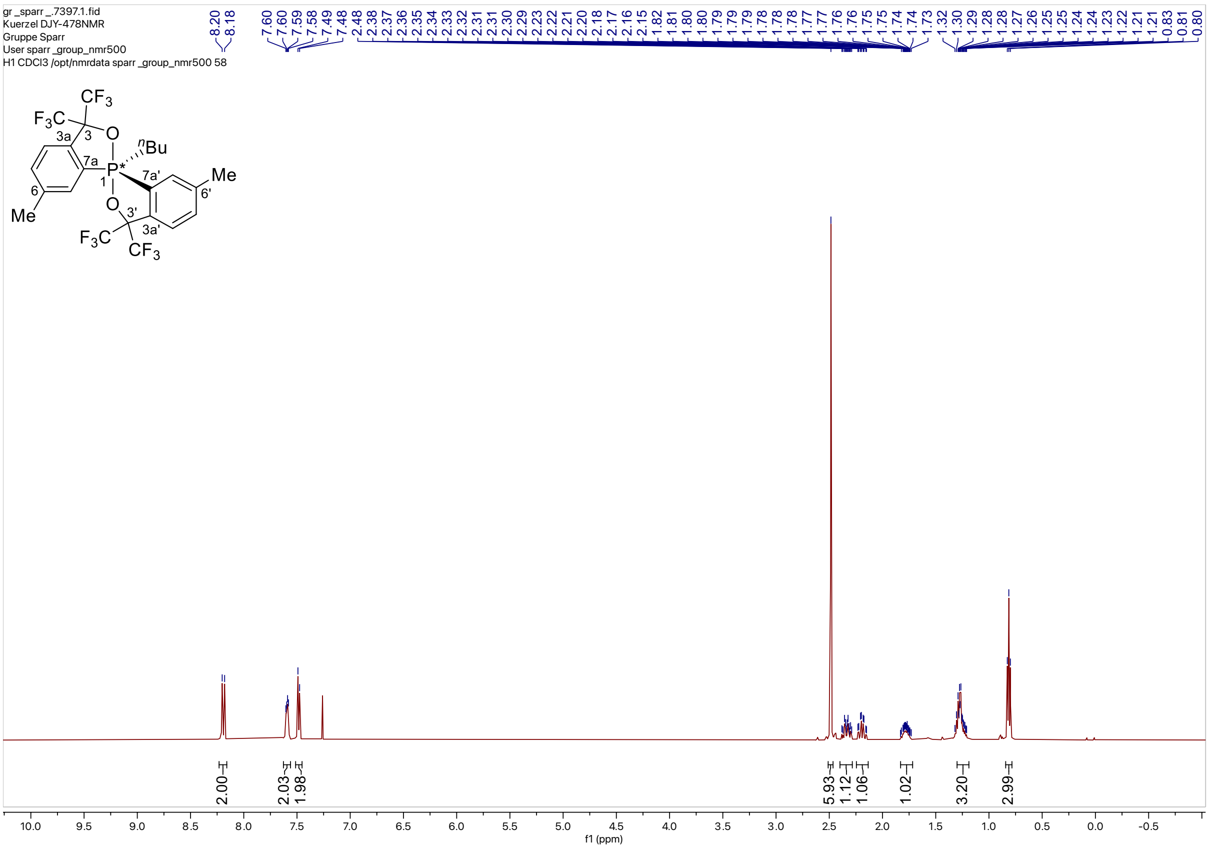

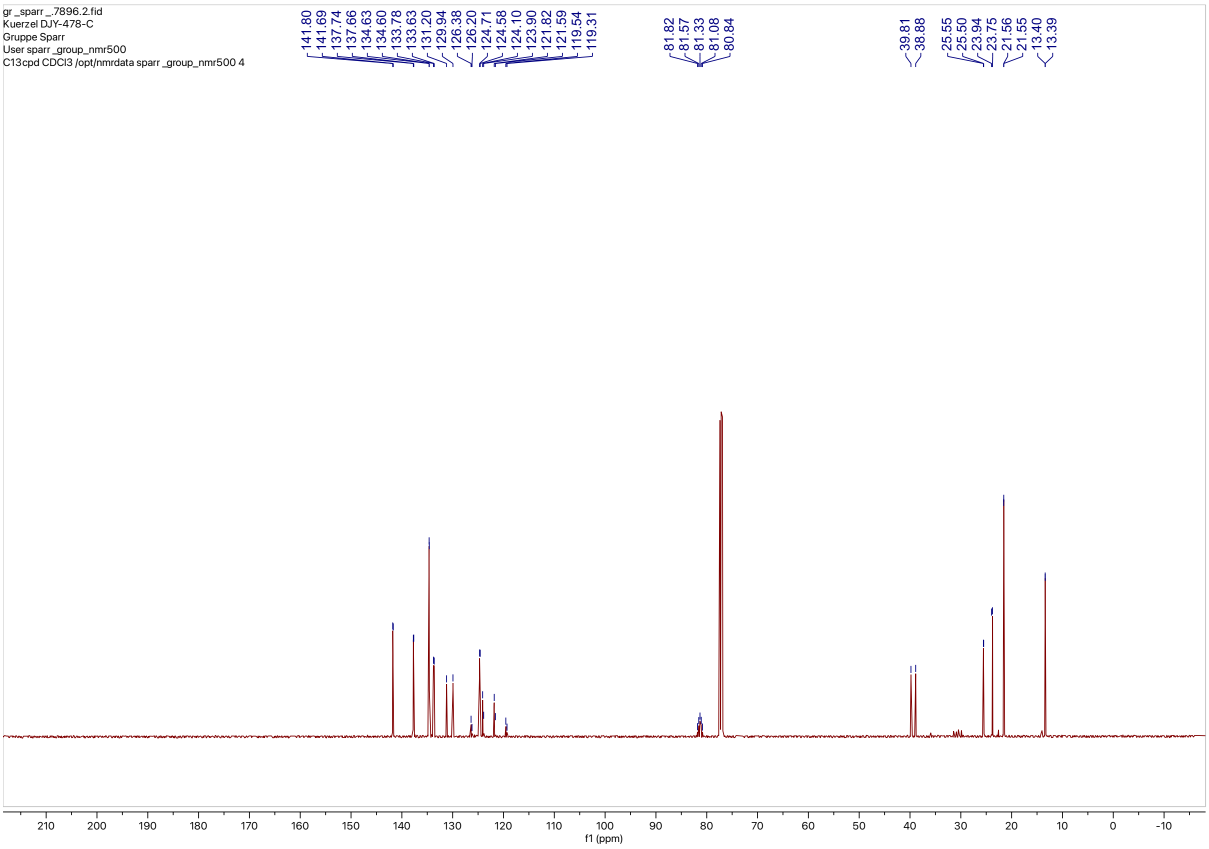

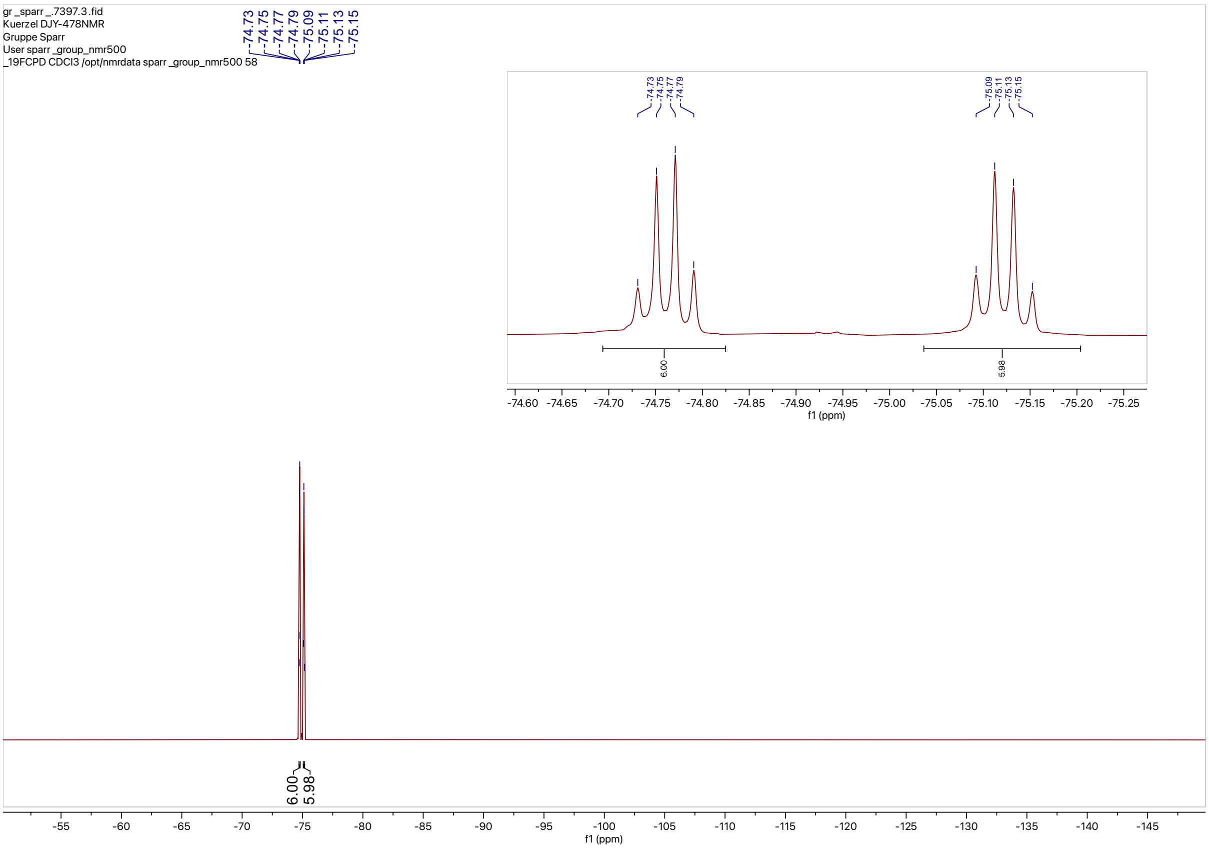

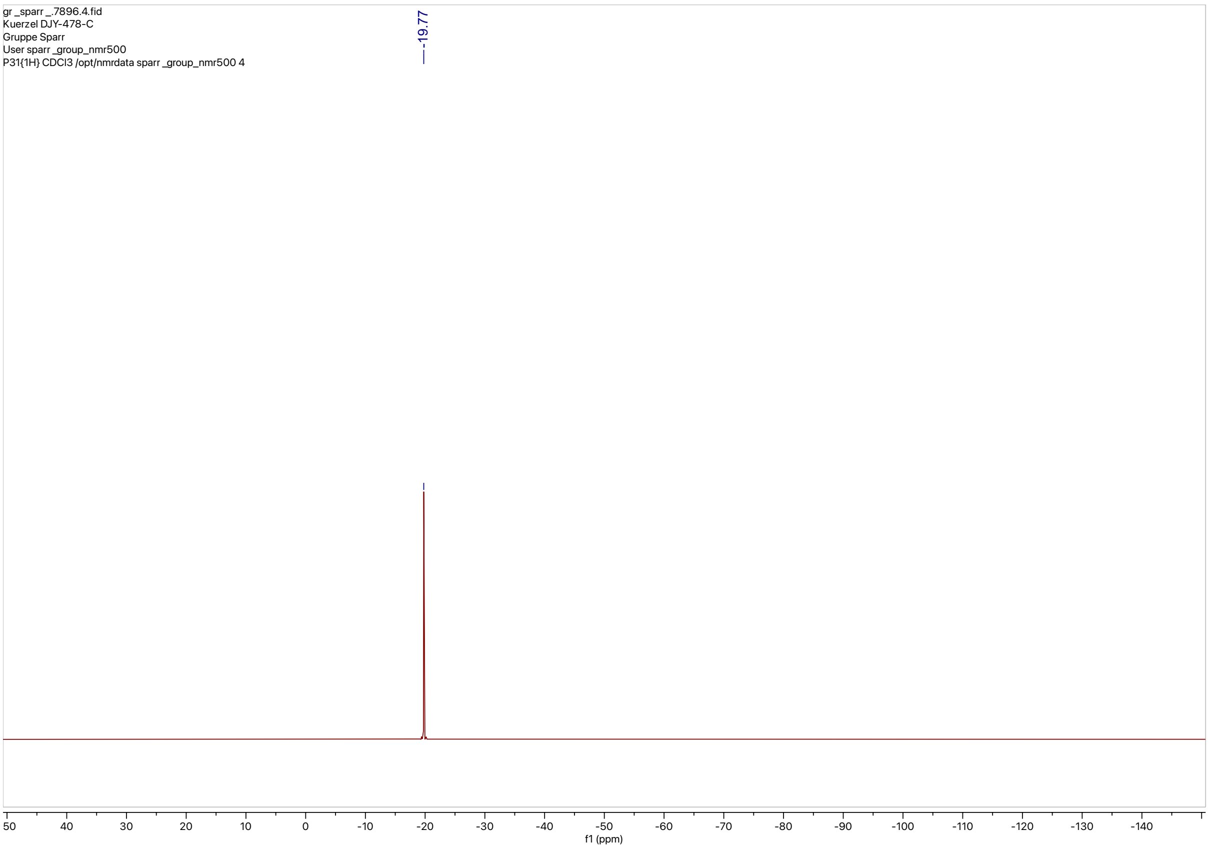

Supplementary Figure 67. ^{1}H (500 MHz, $CDCl_3$, 25 °C), $^{13}C\{^{1}H\}$ (126 MHz, $CDCl_3$, 25 °C), $^{19}F\{^{1}H\}$ (470 MHz, $CDCl_3$, 25 °C) and $^{31}P\{^{1}H\}$ (202 MHz, $CDCl_3$, 25 °C) spectra of (*S*-*trans*)-**2a**

^{1}H, ^{13}C{^{1}H}, ^{19}F{^{1}H} and ^{31}P{^{1}H} spectra of [*TBPY*-5-11'-*A*]-1-Butyl-5,5'-dimethyl-3,3,3',3'-tetrakis(trifluoromethyl)-3*H*,3'*H*-1λ^{5},1'-spirobi[benzo[*c*][2,1]oxaphosphole] ((*S*-*trans*)-2c)

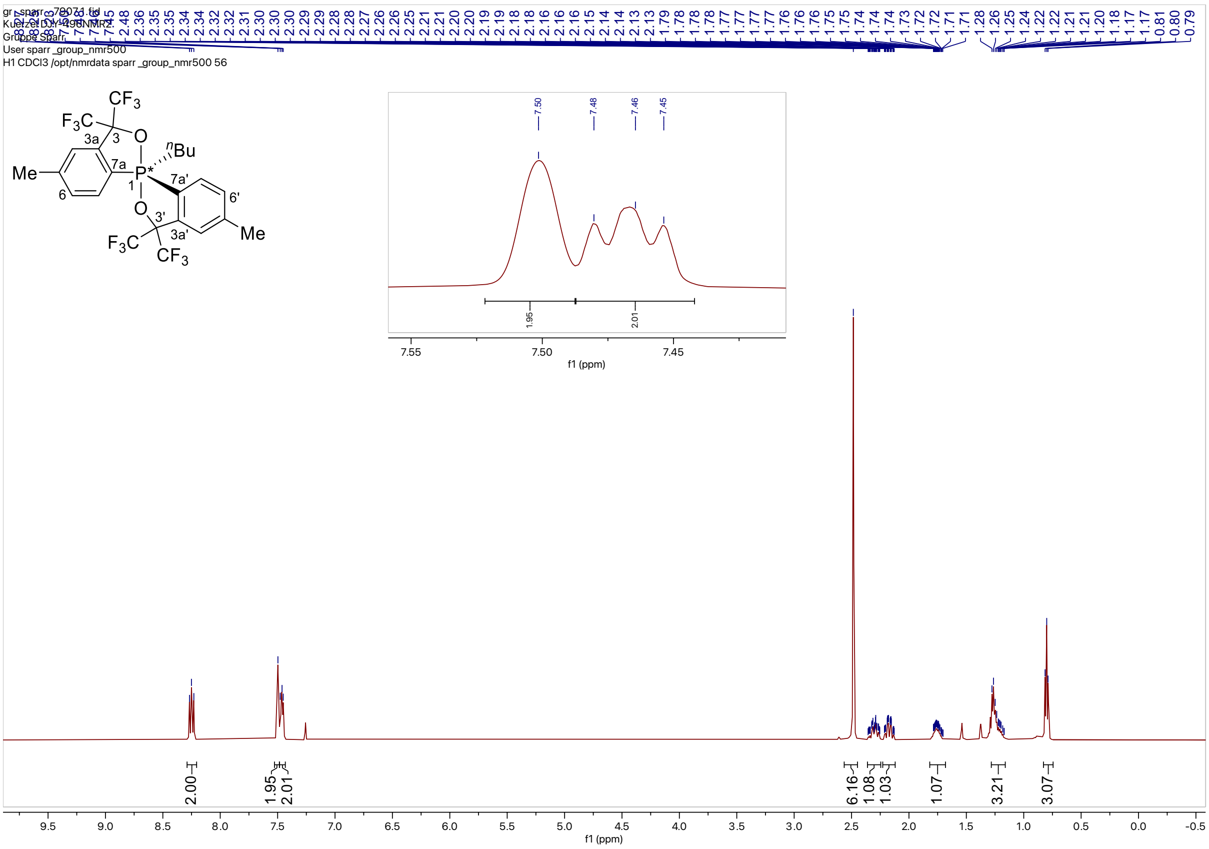

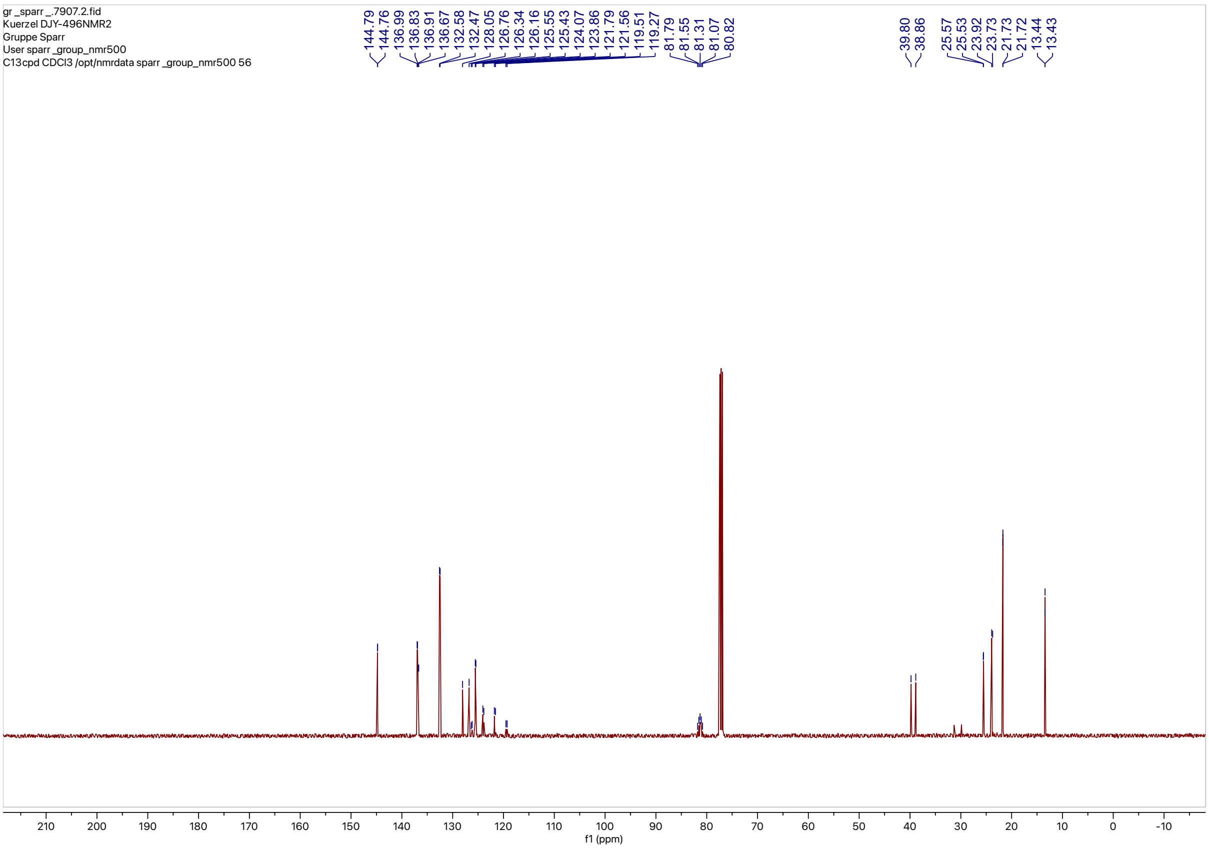

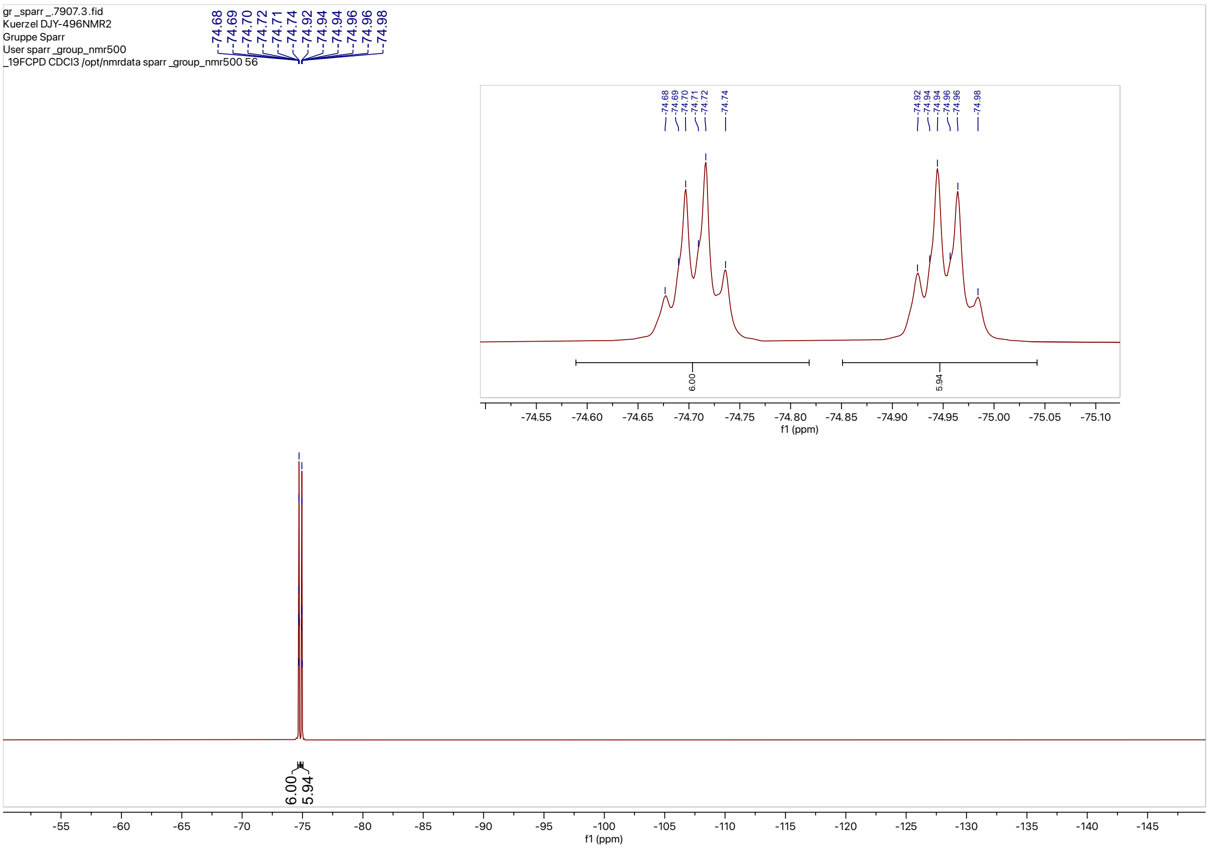

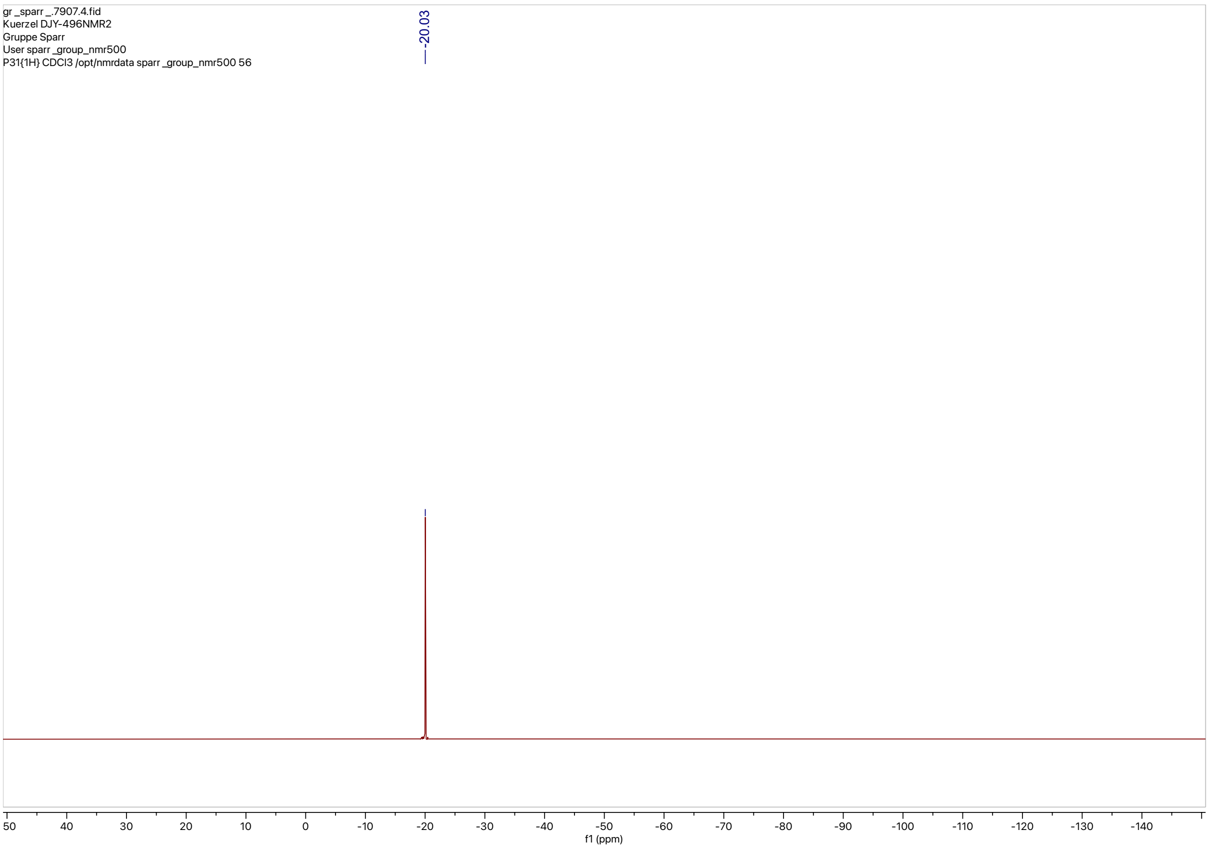

Supplementary Figure 68. ^{1}H (500 MHz, $CDCl_3$, 25 °C), $^{13}C\{^{1}H\}$ (126 MHz, $CDCl_3$, 25 °C), $^{19}F\{^{1}H\}$ (470 MHz, $CDCl_3$, 25 °C) and $^{31}P\{^{1}H\}$ (202 MHz, $CDCl_3$, 25 °C) spectra of (*S*-*trans*)-**2c**

^{1}H, $^{13}C\{^{1}H\}$, $^{19}F\{^{1}H\}$ and $^{31}P\{^{1}H\}$ spectra of [*TBPY*-5-11'-*A*]-1-Butyl-5,5'-dimethoxy-3,3,3',3'-tetrakis(trifluoromethyl)-3*H*,3'*H*-1λ^{5},1'-spirobi[benzo[*c*][2,1]oxaphosphole] ((*S*-*trans*)-2d)

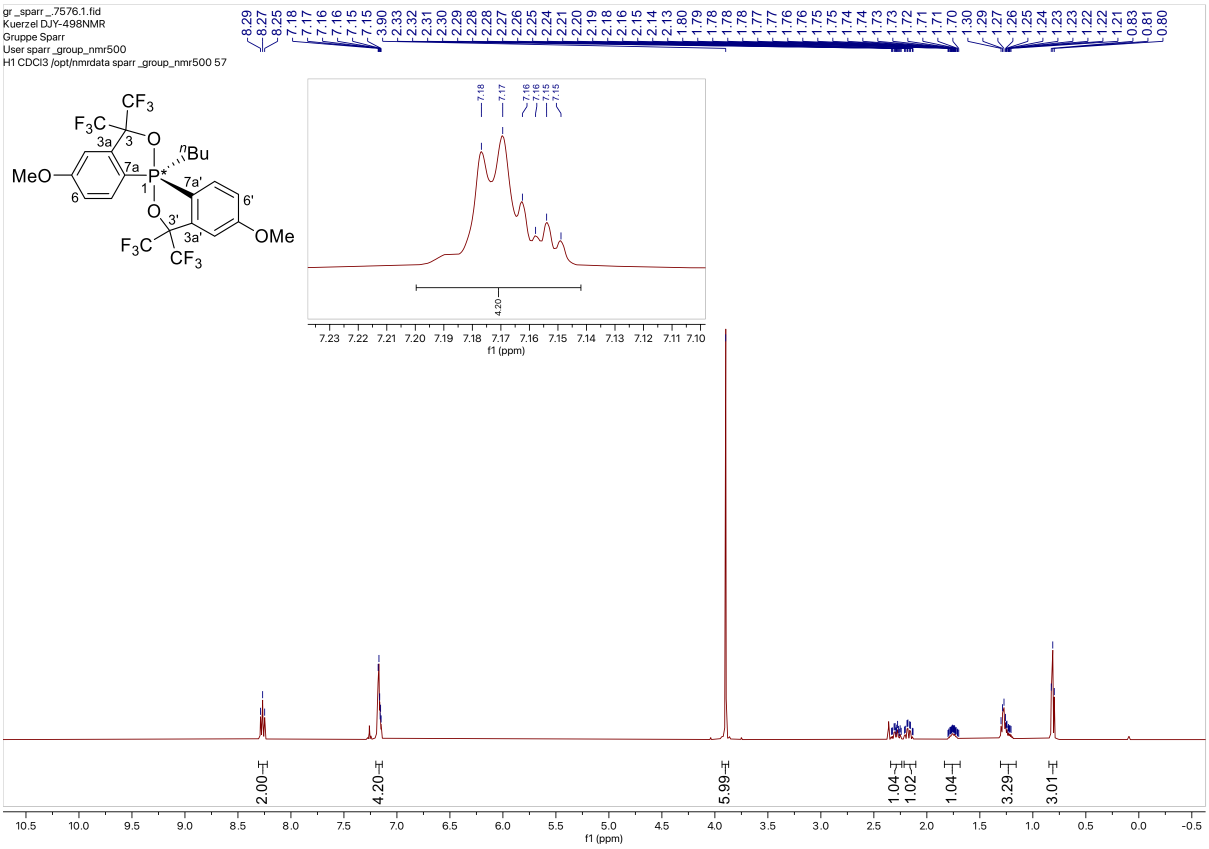

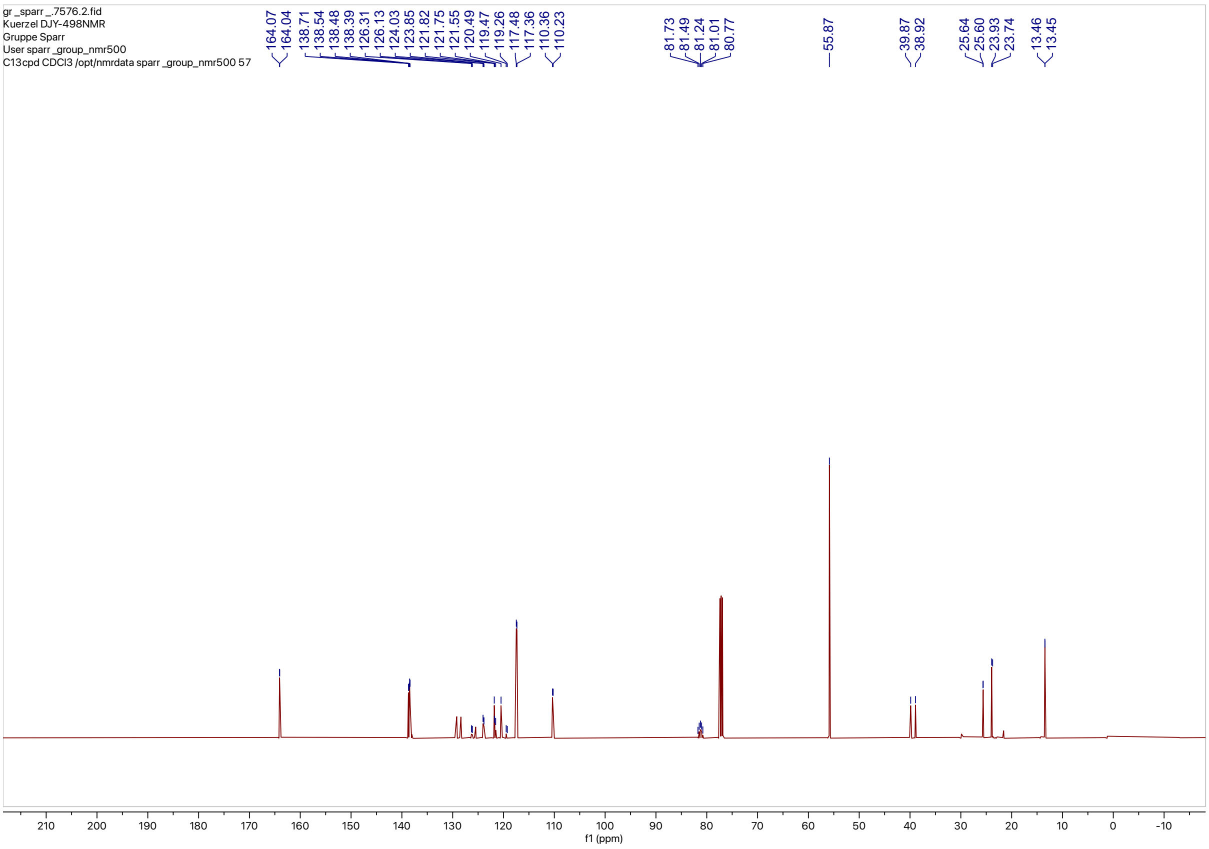

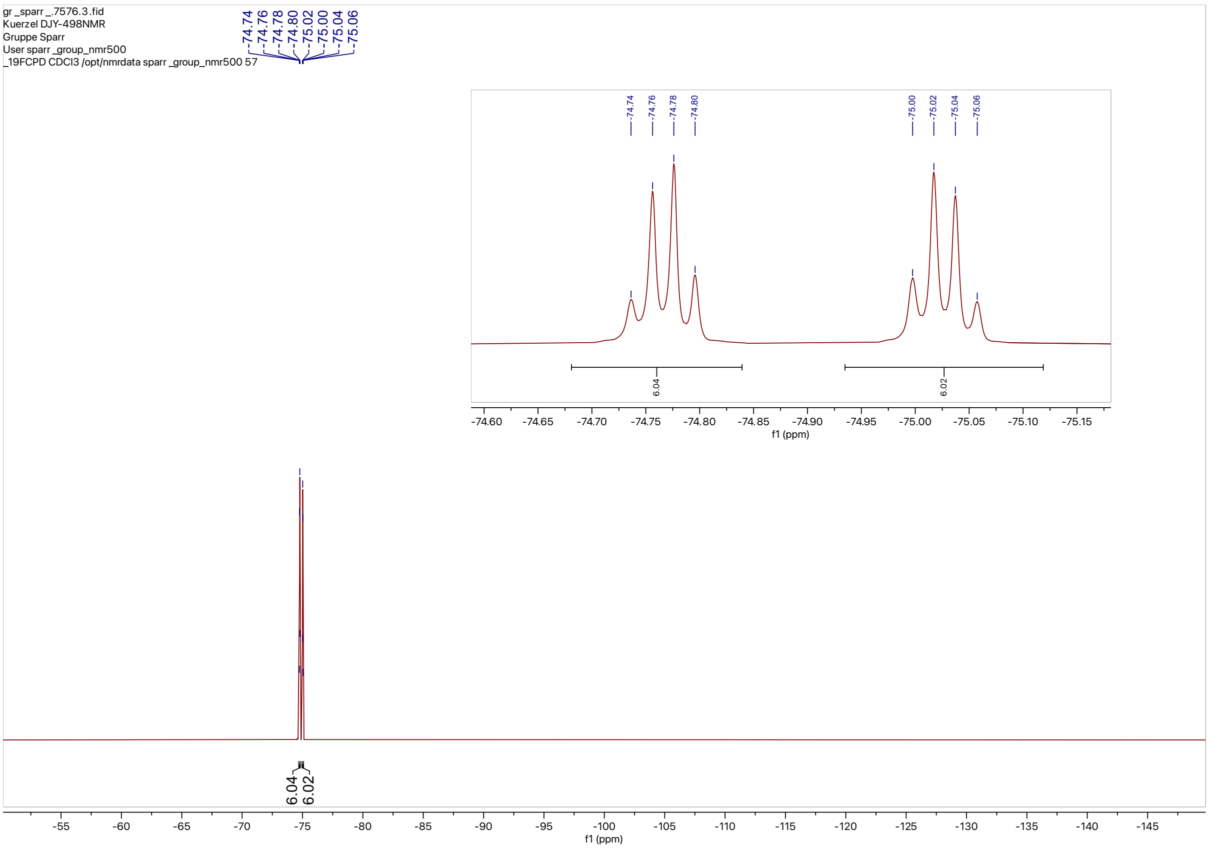

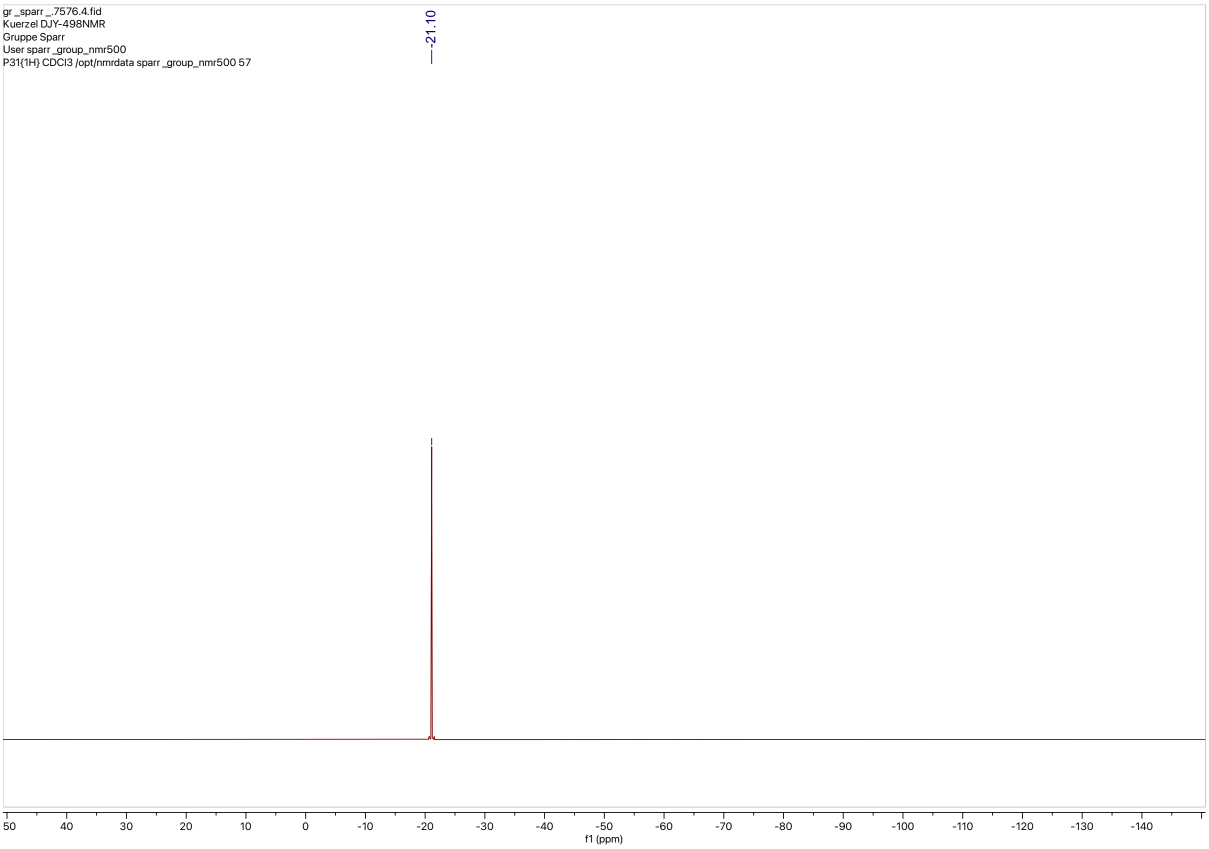

Supplementary Figure 69. ^{1}H (500 MHz, $CDCl_3$, 25 °C), ^{13}C{^{1}H} (126 MHz, $CDCl_3$, 25 °C), ^{19}F{^{1}H} (470 MHz, $CDCl_3$, 25 °C) and ^{31}P{^{1}H} (202 MHz, $CDCl_3$, 25 °C) spectra of (*S-trans*)-**2d**

^{1}H, ^{13}C{^{1}H}, ^{19}F{^{1}H} and ^{31}P{^{1}H} spectra of [*TBPY*-5-11'-*A*]-1-Butyl-5,5'-dimethoxy-6,6'-dimethyl-3,3,3',3'-tetrakis(trifluorome-thyl)-3*H*,3'*H*-1λ^5,1'-spirobi[benzo[*c*][2,1]oxaphosphole] ((*S*-*trans*)-2e)

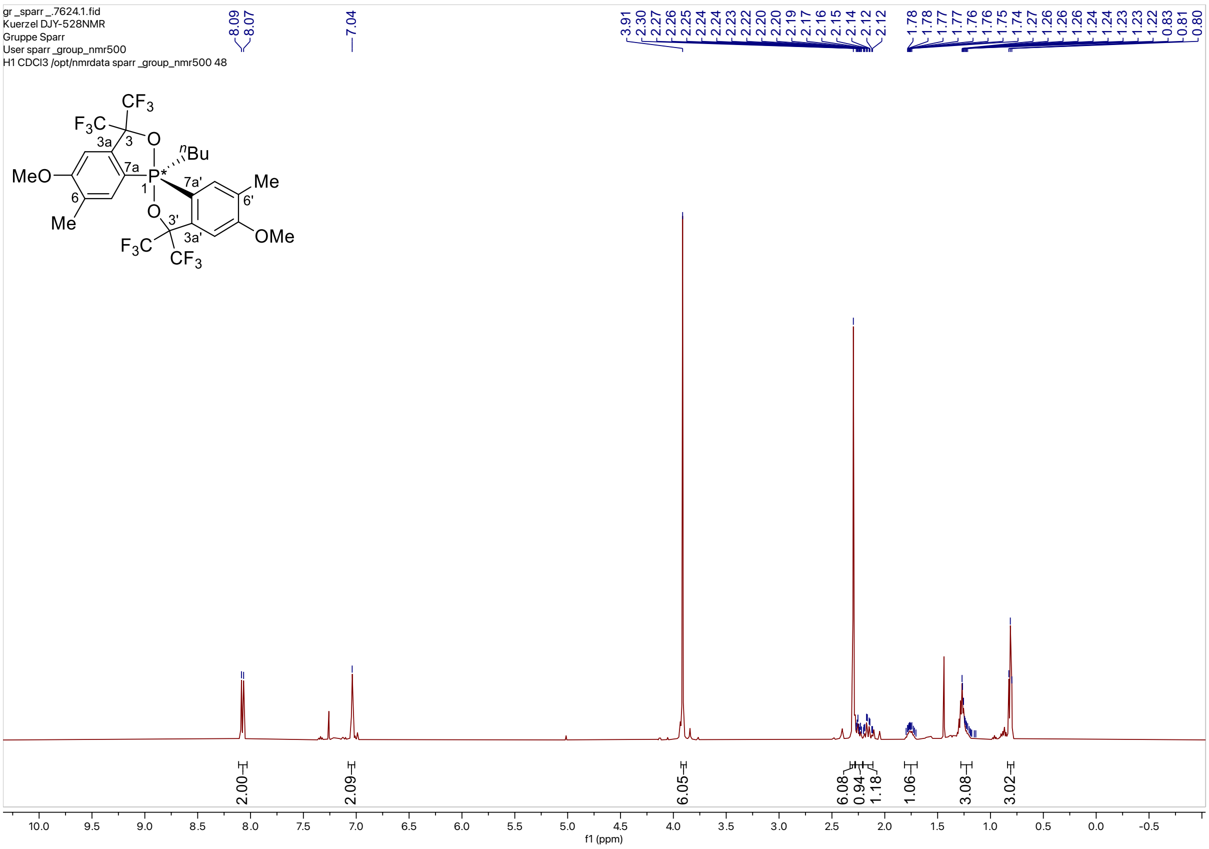

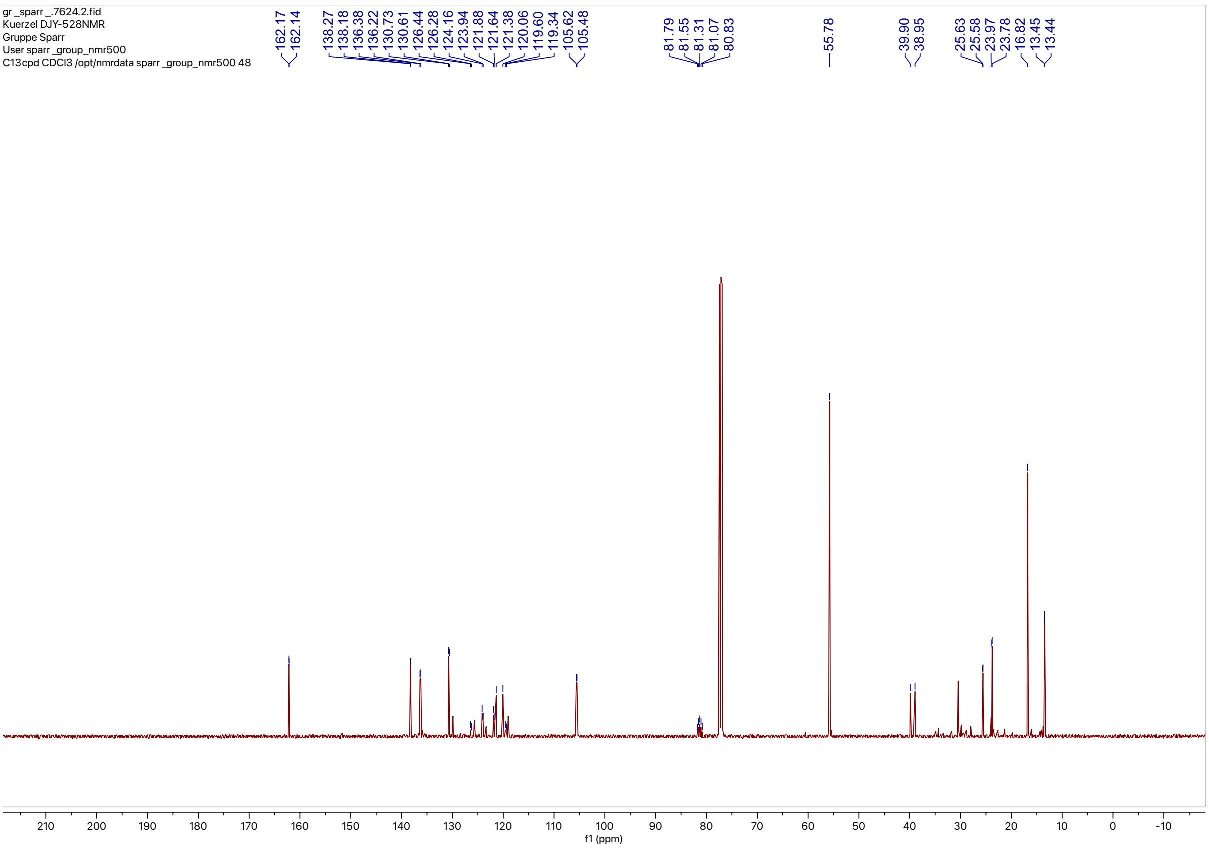

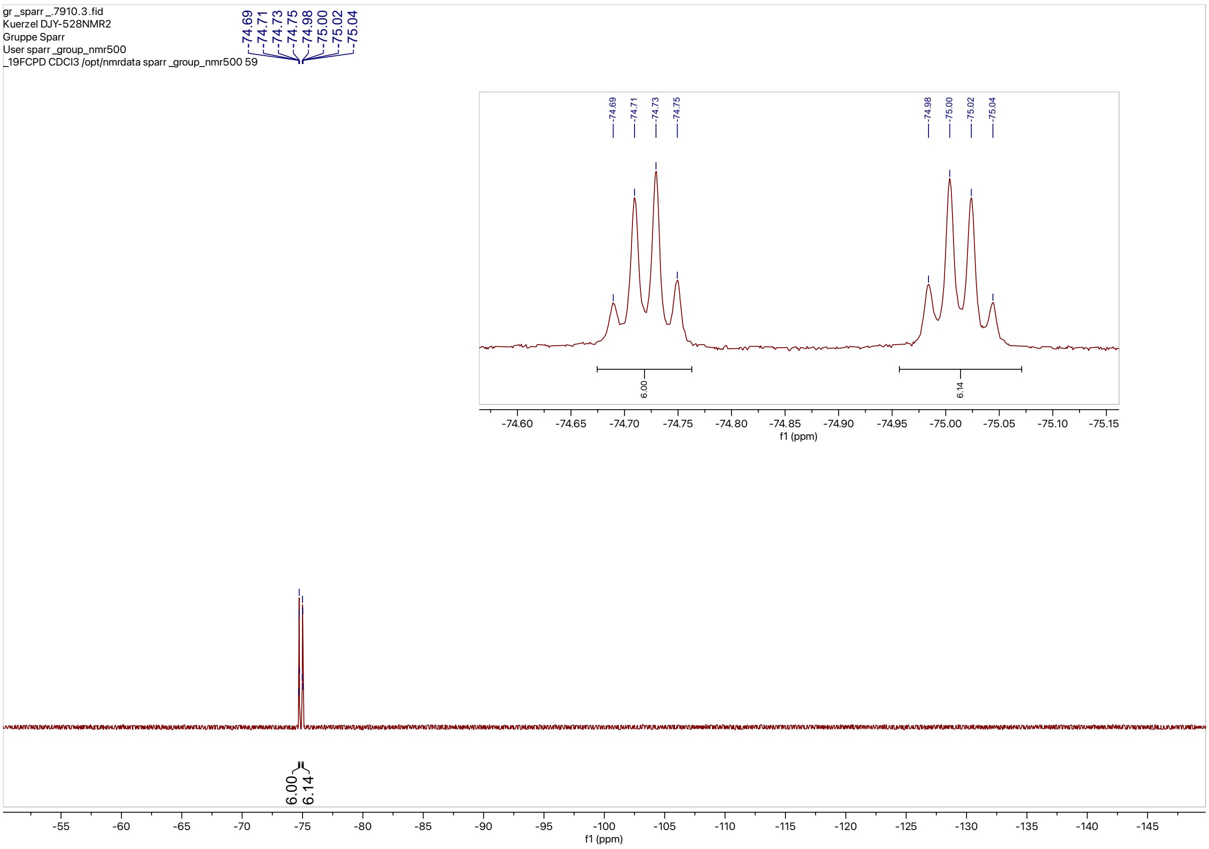

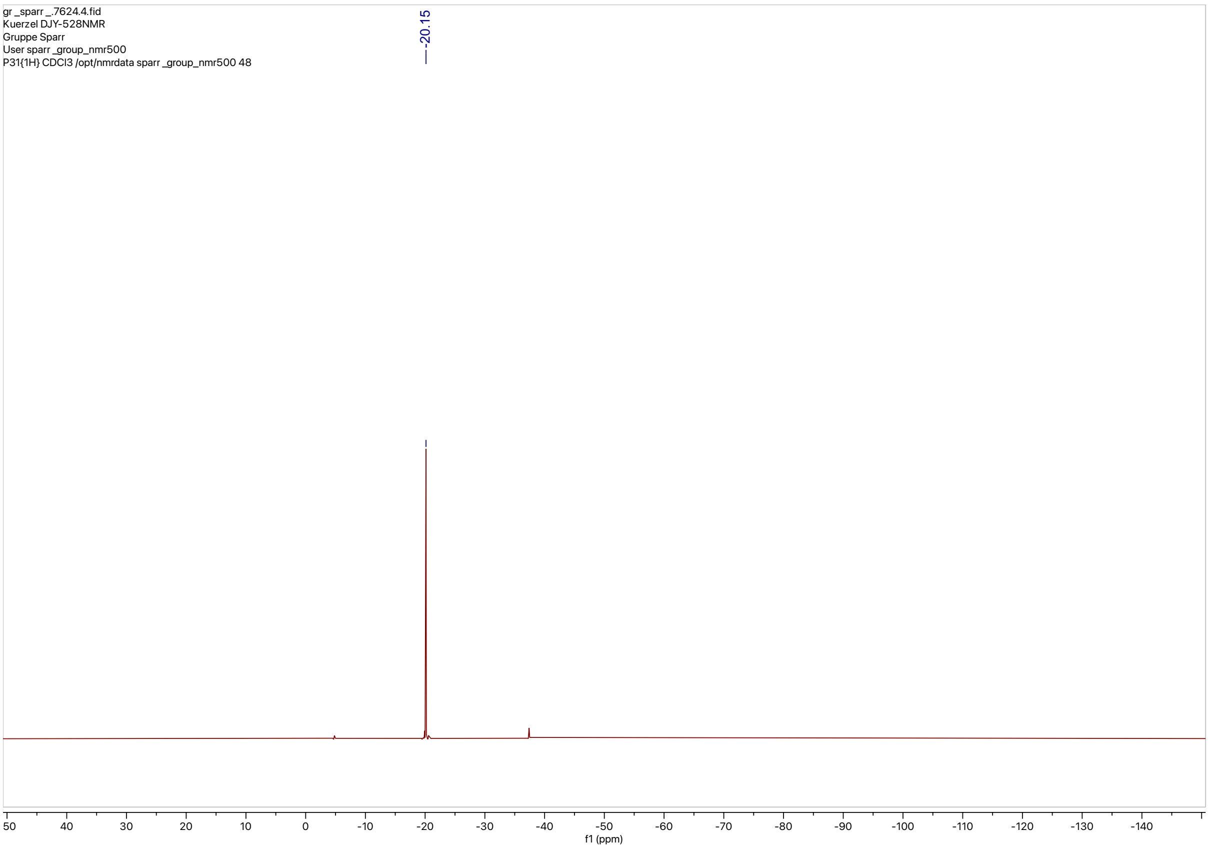

Supplementary Figure 70. ^{1}H (500 MHz, $CDCl_3$, 25 °C), $^{13}C\{^{1}H\}$ (126 MHz, $CDCl_3$, 25 °C), $^{19}F\{^{1}H\}$ (470 MHz, $CDCl_3$, 25 °C) and $^{31}P\{^{1}H\}$ (202 MHz, $CDCl_3$, 25 °C) spectra of (*S*-*trans*)-**2e**

^{1}H, ^{13}C{^{1}H}, ^{19}F{^{1}H} and ^{31}P{^{1}H} spectra of [*TBPY*-5-11'-*A*]-1-Butyl-3,3,3',3'-tetrakis(trifluoromethyl)-3*H*,3'*H*-1λ^{5},1'-spirobi[naphtho[2,3-*c*][2,1]oxaphosphole] ((*S*-*trans*)-2f)

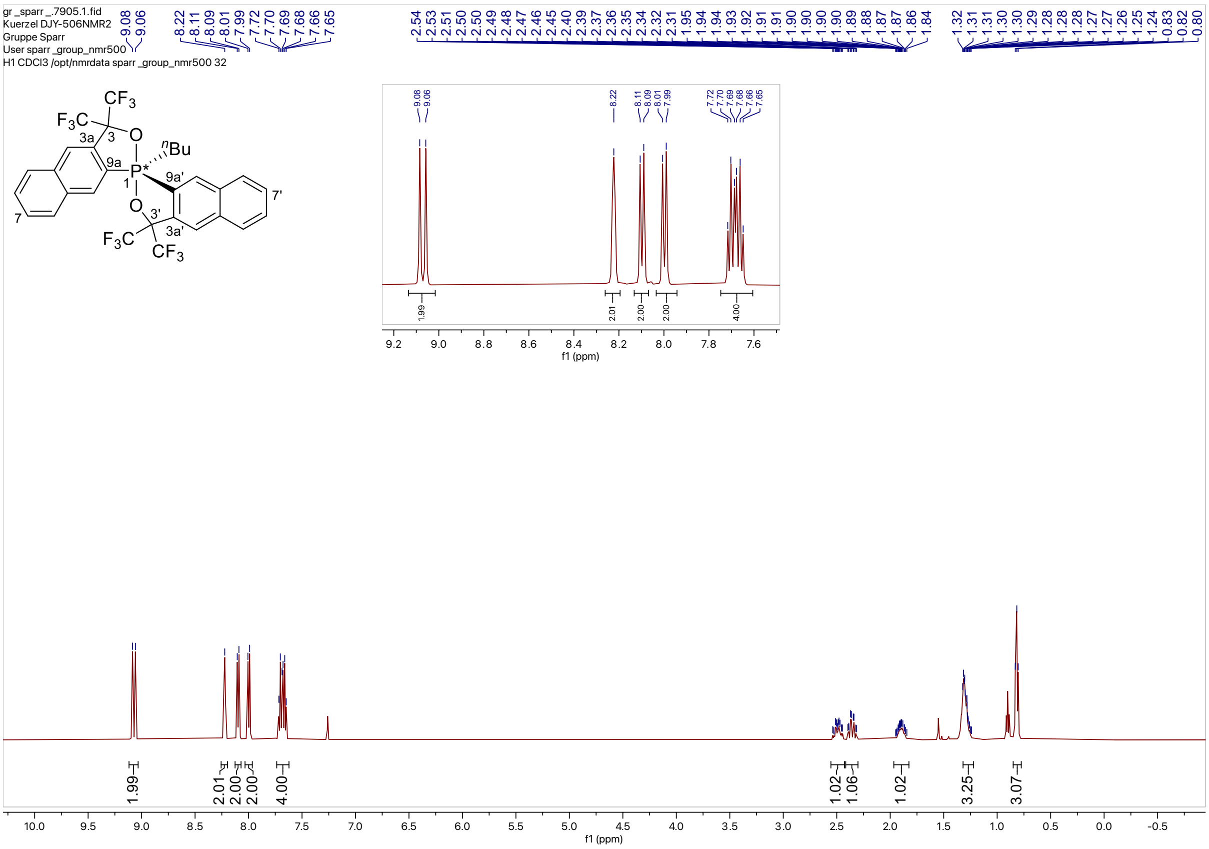

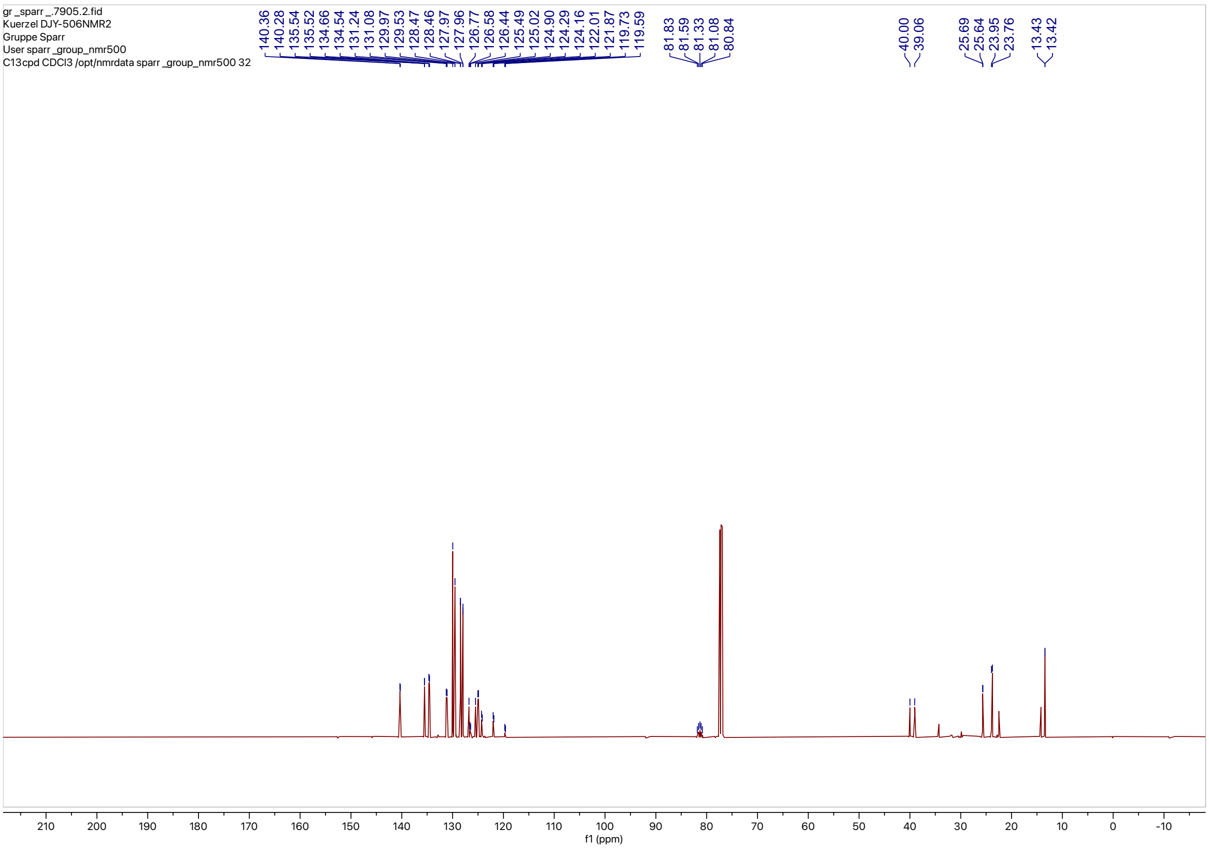

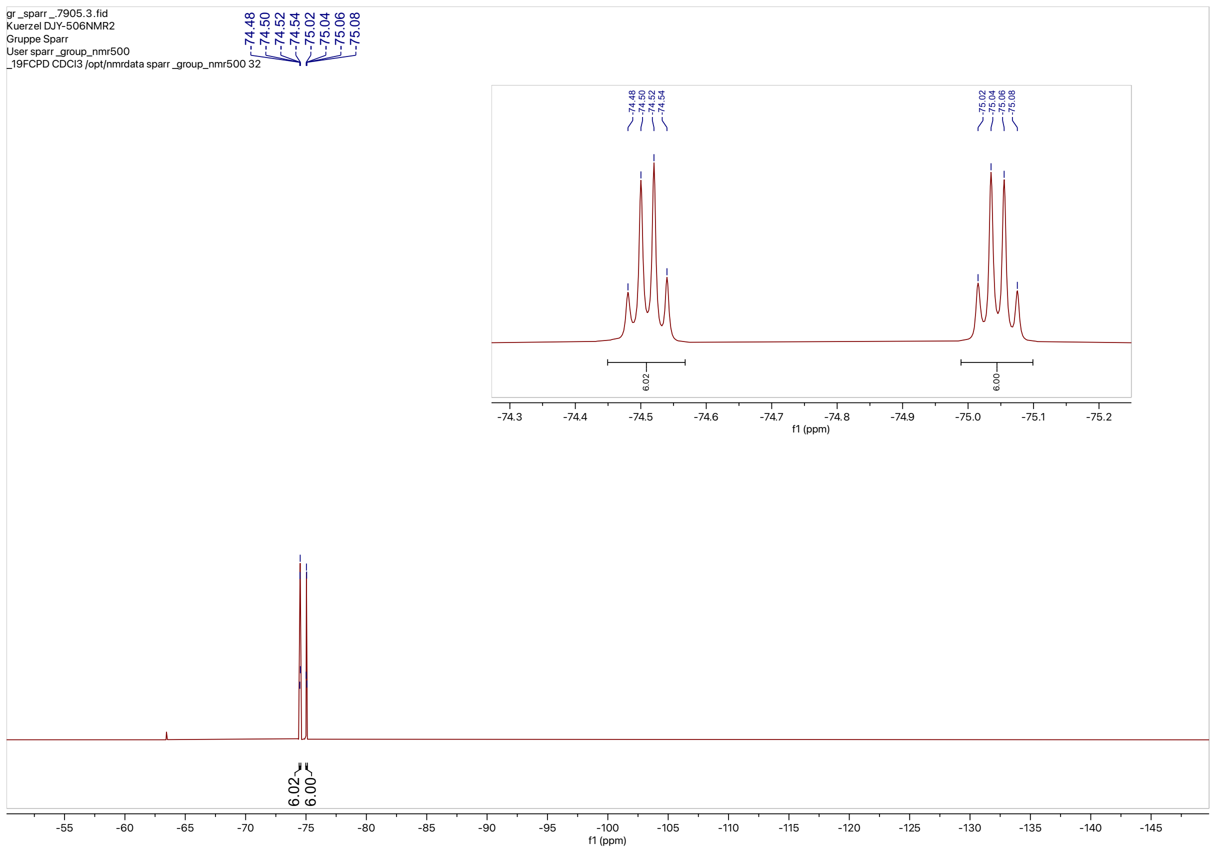

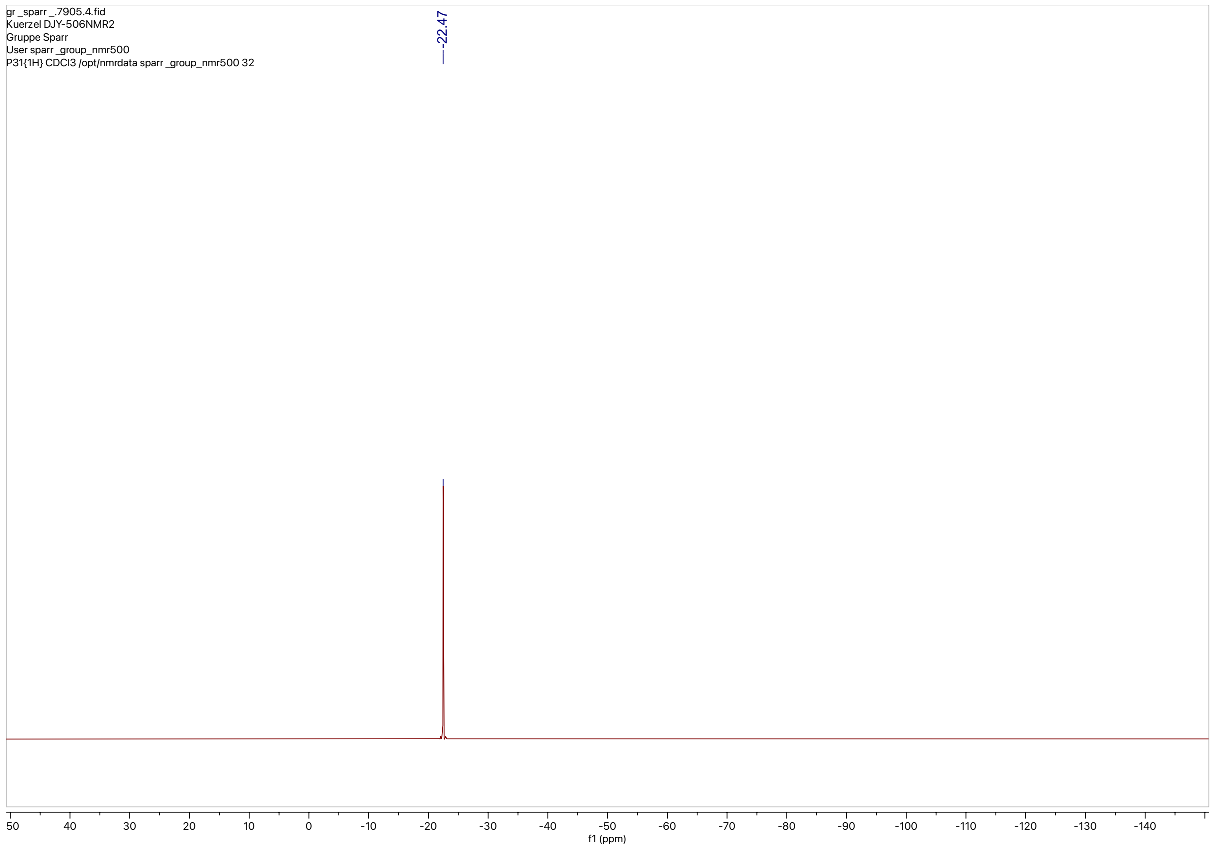

Supplementary Figure 71. ^{1}H (500 MHz, $CDCl_3$, 25 °C), $^{13}C\{^{1}H\}$ (126 MHz, $CDCl_3$, 25 °C), $^{19}F\{^{1}H\}$ (470 MHz, $CDCl_3$, 25 °C) and $^{31}P\{^{1}H\}$ (202 MHz, $CDCl_3$, 25 °C) spectra of (*S*-*trans*)-**2f**

^{1}H, ^{13}C{^{1}H}, ^{19}F{^{1}H} and ^{31}P{^{1}H} spectra of [*TBPY*-5-11'-*A*]-1-Butyl-7,7'-dimethoxy-3,3,3',3'-tetrakis(trifluoromethyl)-3*H*,3'*H*-1λ^5,1'-spirobi[naphtho[2,3-*c*][2,1]oxaphosphole] ((*S*-*trans*)-2g)

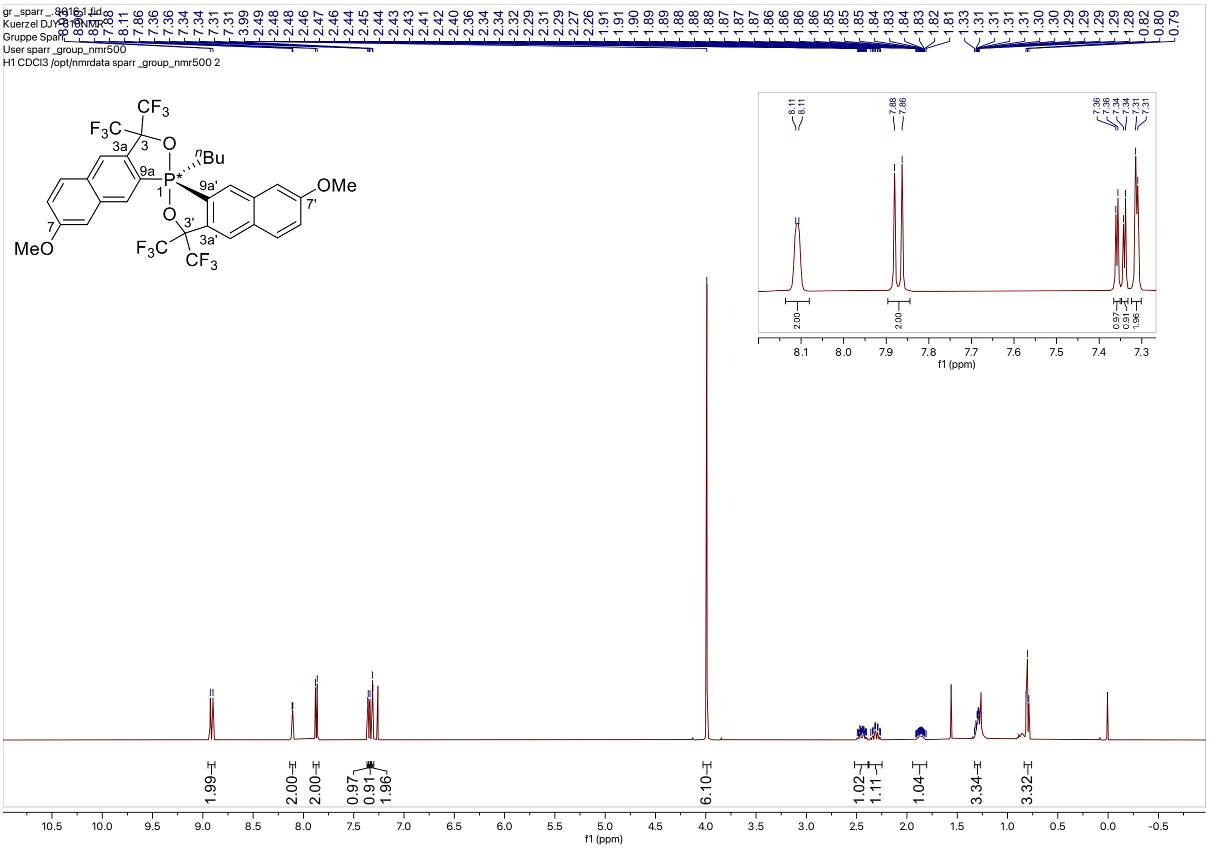

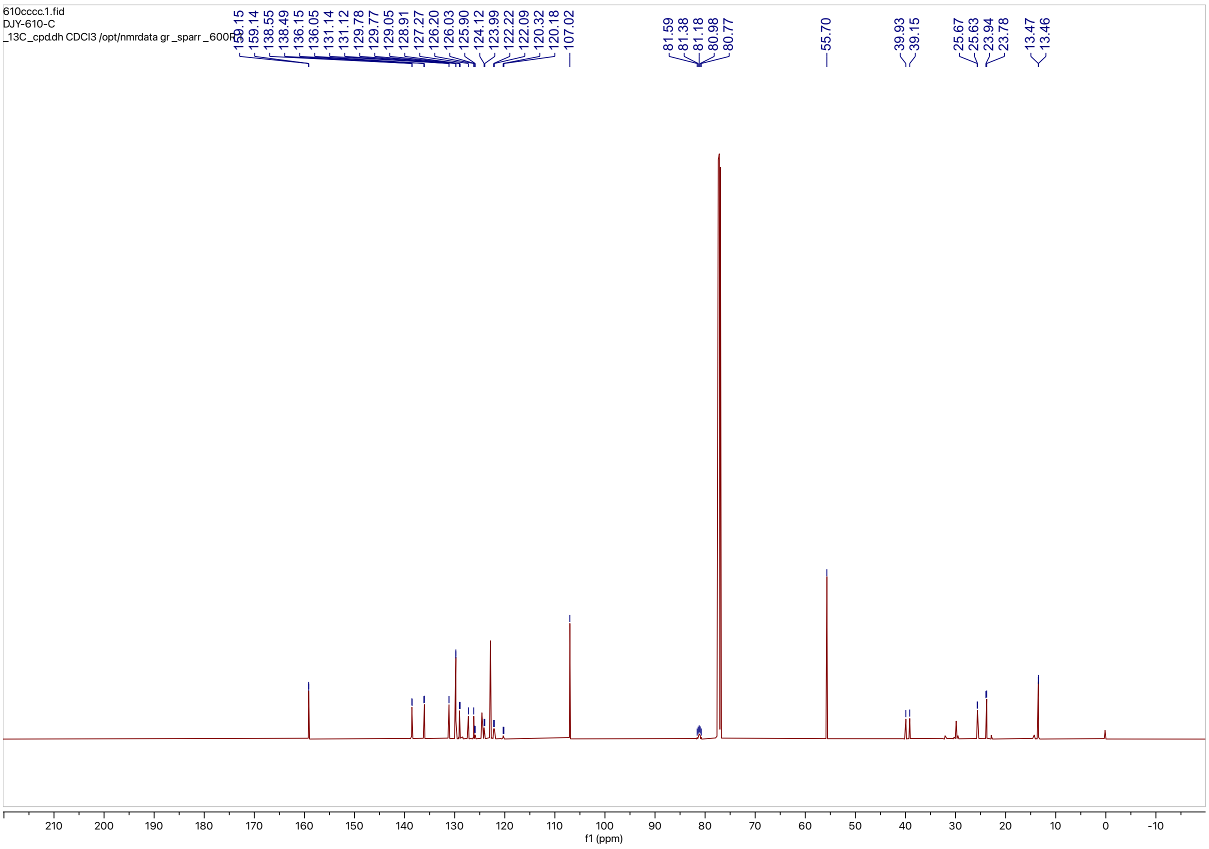

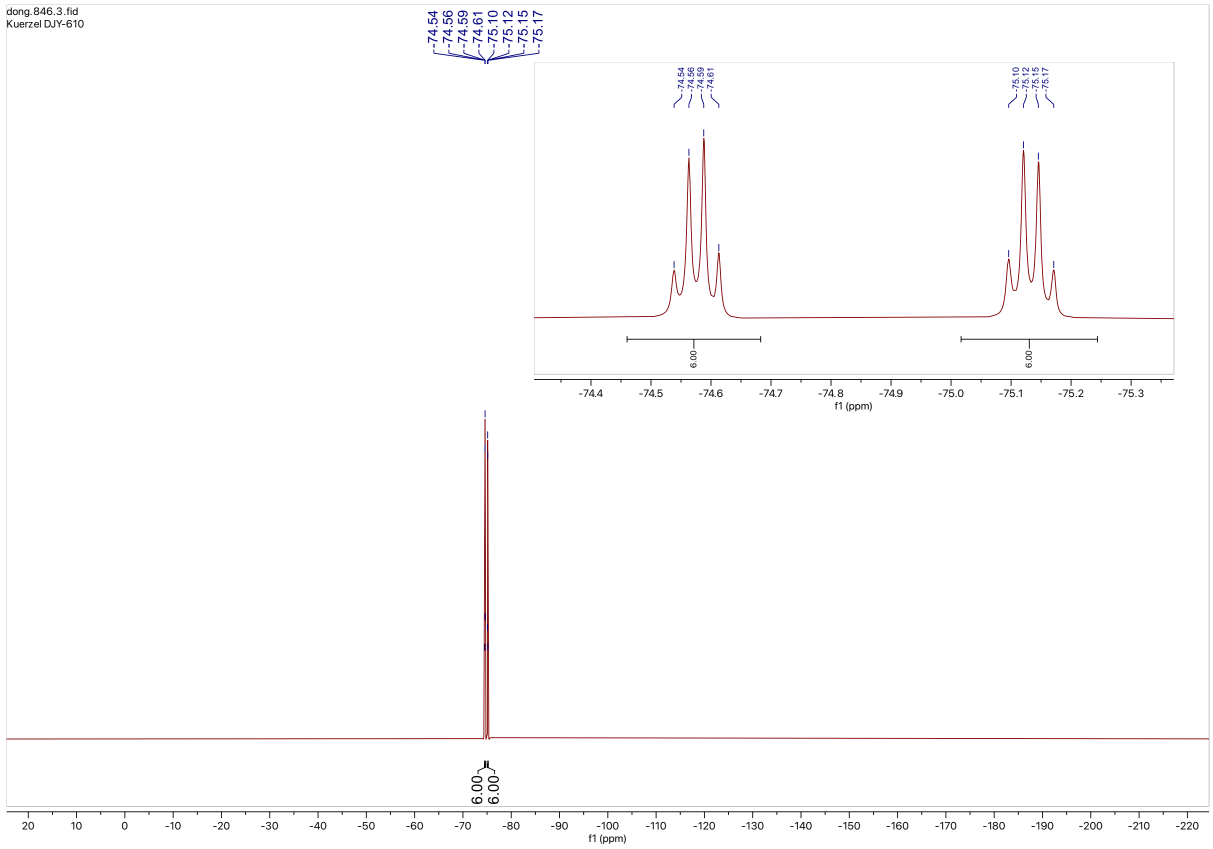

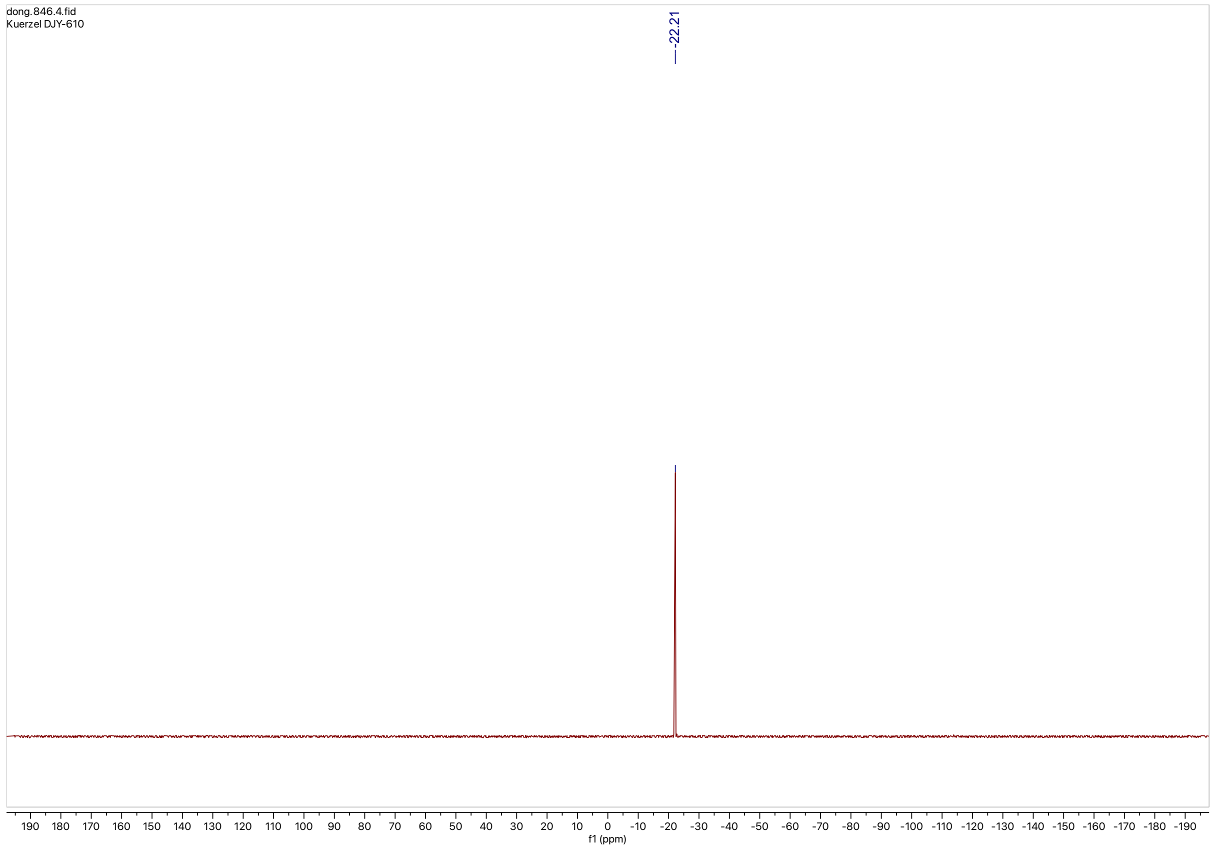

Supplementary Figure 72. ^{1}H (500 MHz, $CDCl_3$, 25 °C), ^{13}C{^{1}H} (151 MHz, $CDCl_3$, 25 °C), ^{19}F{^{1}H} (376 MHz, $CDCl_3$, 25 °C) and ^{31}P{^{1}H} (162 MHz, $CDCl_3$, 25 °C) spectra of (*S*-*trans*)-**2g**

[1]H, [13]C{[1]H}, ^{19}F{^{1}H} and ^{31}P{^{1}H} NMR spectra of [*TBPY*-5-11'-*A*]-1-Hexyl-6,6'-dimethyl-3,3,3',3'-tetrakis(trifluoromethyl)-3*H*,3'*H*-1λ[5],1'-spirobi[benzo[*c*][2,1]oxaphosphole] ((*S*-*trans*)-2h)

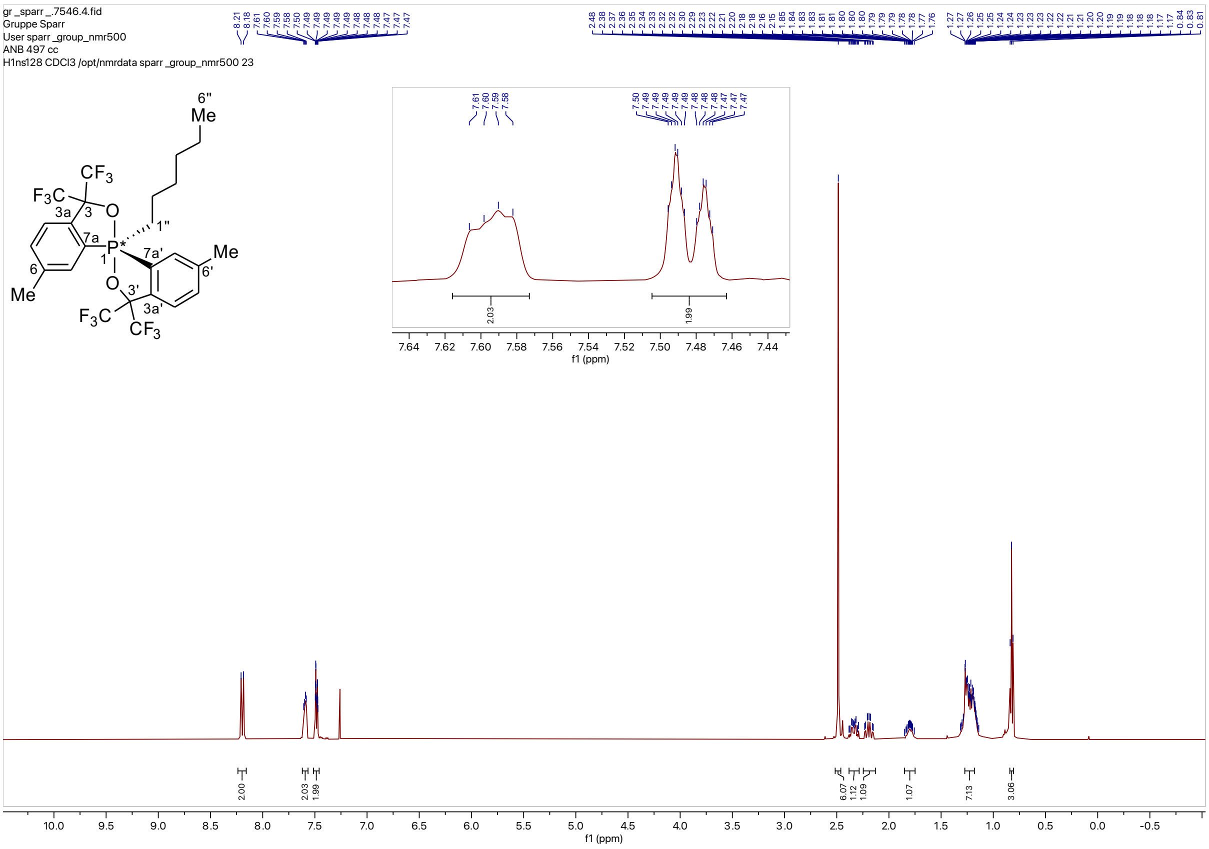

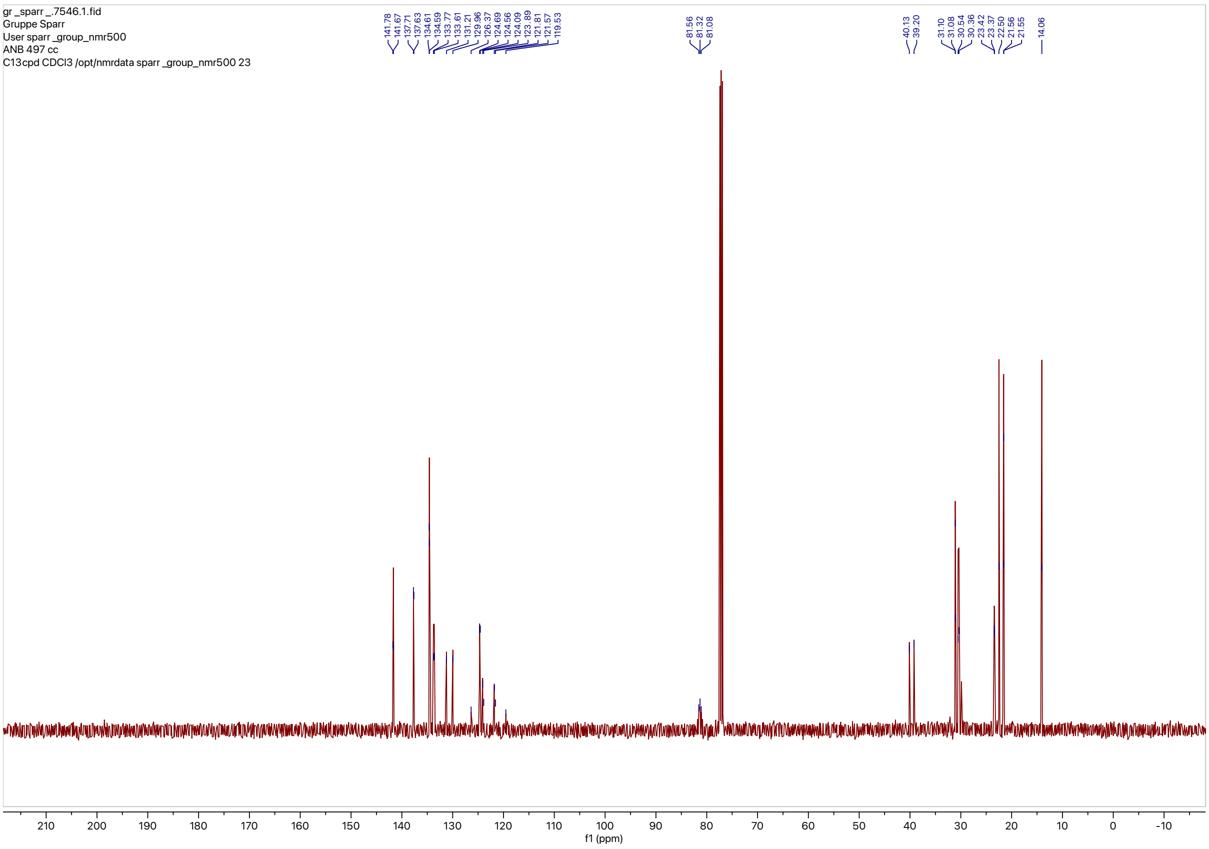

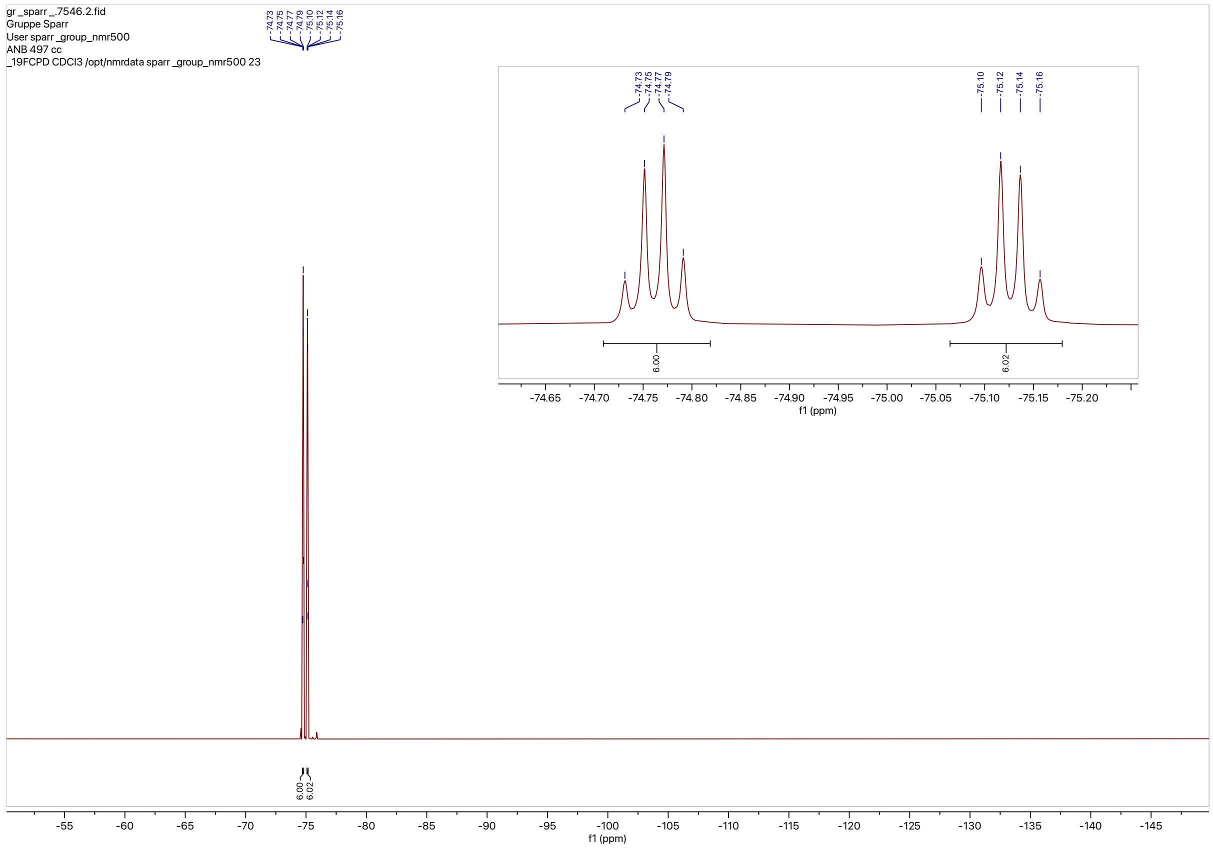

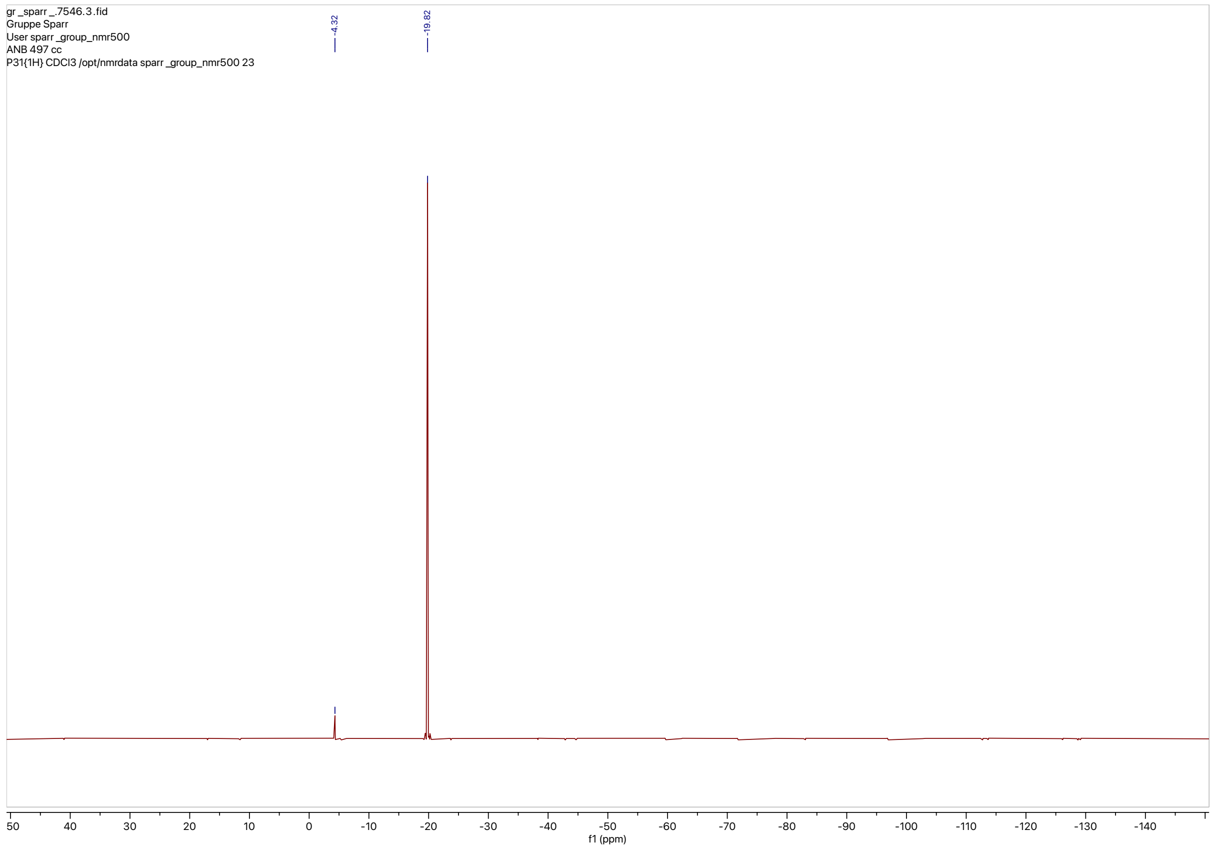

Supplementary Figure 73. ^{1}H (500 MHz, CDCl$_3$, 25 °C), ^{13}C{^{1}H} (126 MHz, CDCl$_3$, 25 °C), ^{19}F{^{1}H} (470 MHz, CDCl$_3$, 25 °C) and ^{31}P{^{1}H} (202 MHz, CDCl$_3$, 25 °C) spectra of (*S*-*trans*)-**2h**

^1H, $^{13}C\{^1H\}$, $^{19}F\{^1H\}$ and $^{31}P\{^1H\}$ NMR spectra of [*TBPY*-5-11'-*A*]-6,6'-Dimethyl-1-(5-methylhexyl)-3,3,3',3'-tetrakis(trifluoromethyl)-3*H*,3'*H*-1λ[5],1'-spirobi[benzo[*c*][2,1]oxaphosphole] ((*S*-*trans*)-2i)

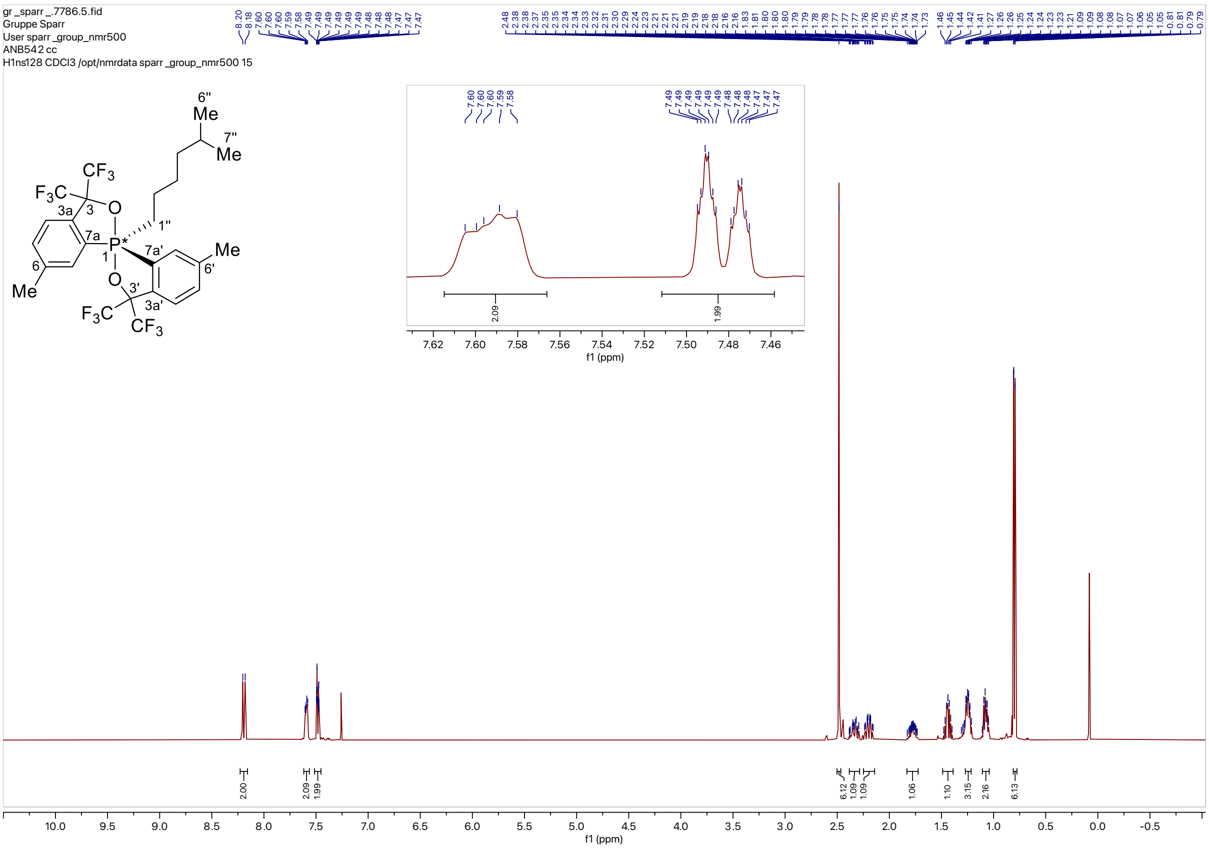

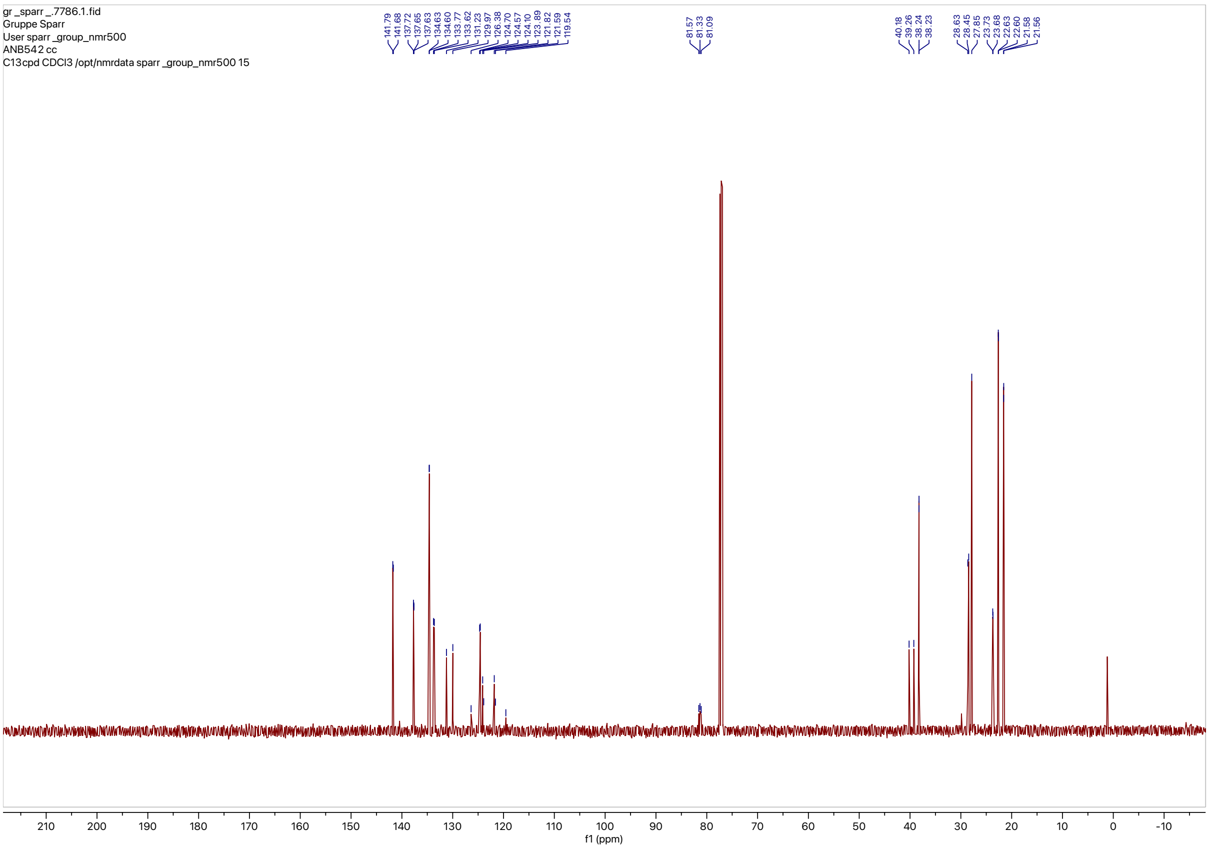

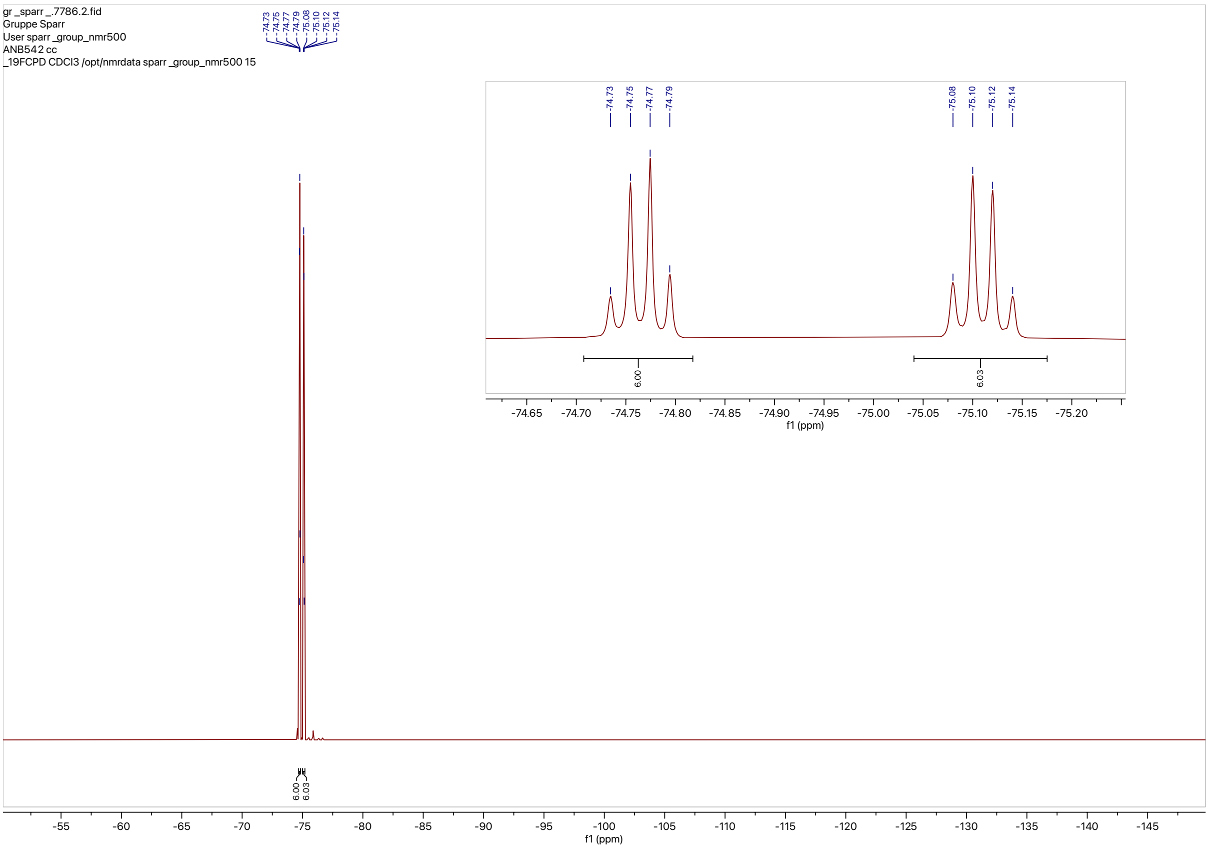

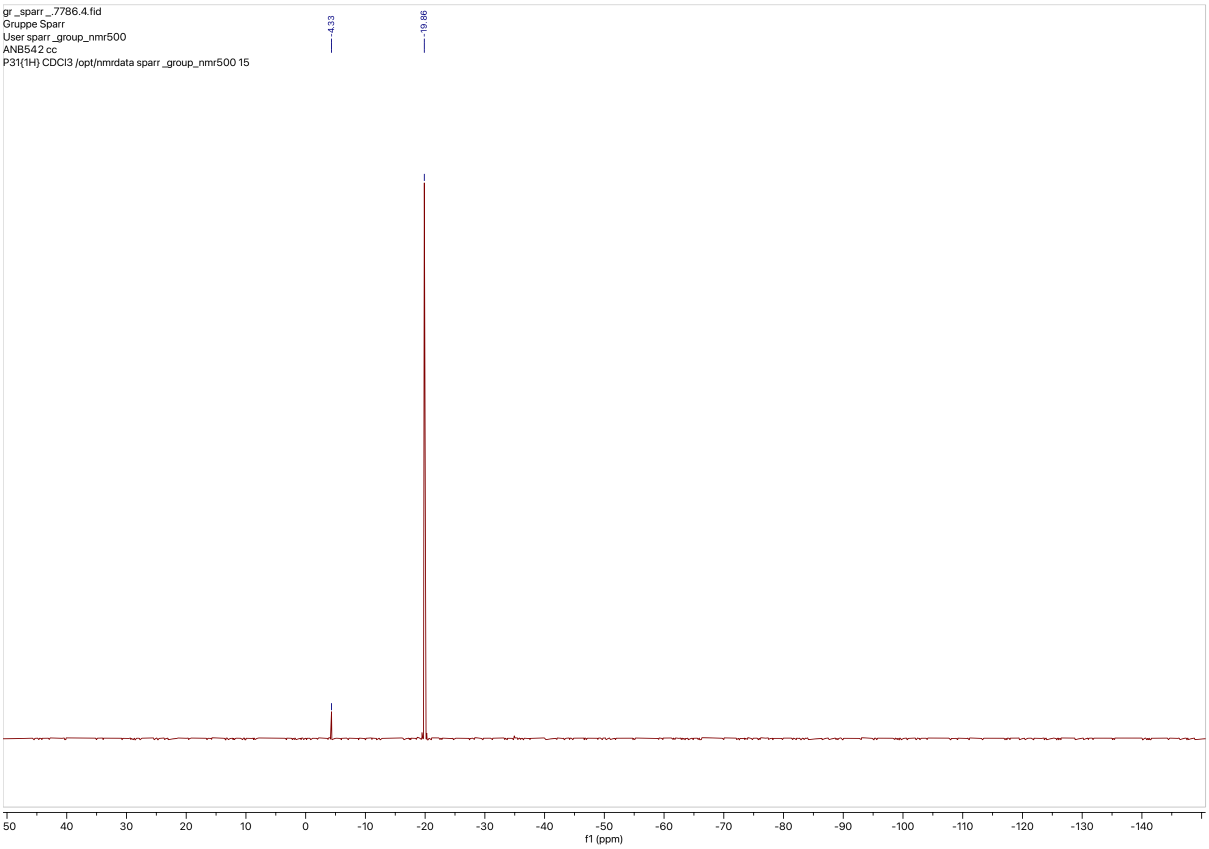

Supplementary Figure 74. ^{1}H (500 MHz, $CDCl_3$, 25 °C), ^{13}C{^{1}H} (126 MHz, $CDCl_3$, 25 °C), ^{19}F{^{1}H} (470 MHz, $CDCl_3$, 25 °C) and ^{31}P{^{1}H} (202 MHz, $CDCl_3$, 25 °C) spectra of (*S*-*trans*)-**2i**

[1]H, [13]C{[1]H}, ^{19}F{^{1}H} and ^{31}P{^{1}H} NMR spectra of [*TBPY*-5-11'-*A*]-6,6'-Dimethyl-1-(4-phenylbutyl)-3,3,3',3'-tetrakis(trifluoromethyl)-3*H*,3'*H*-1λ[5],1'-spirobi[benzo[*c*][2,1]oxaphosphole] ((*S*-*trans*)-2j)

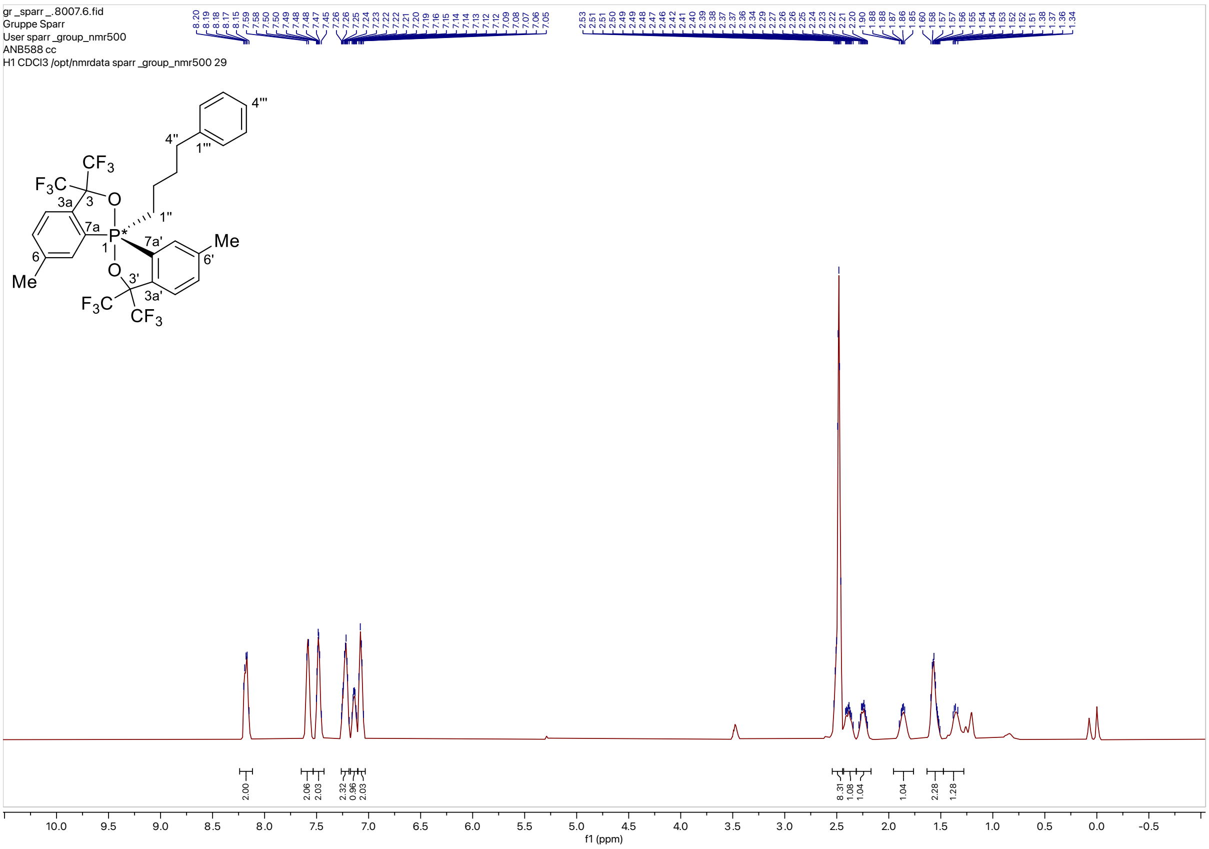

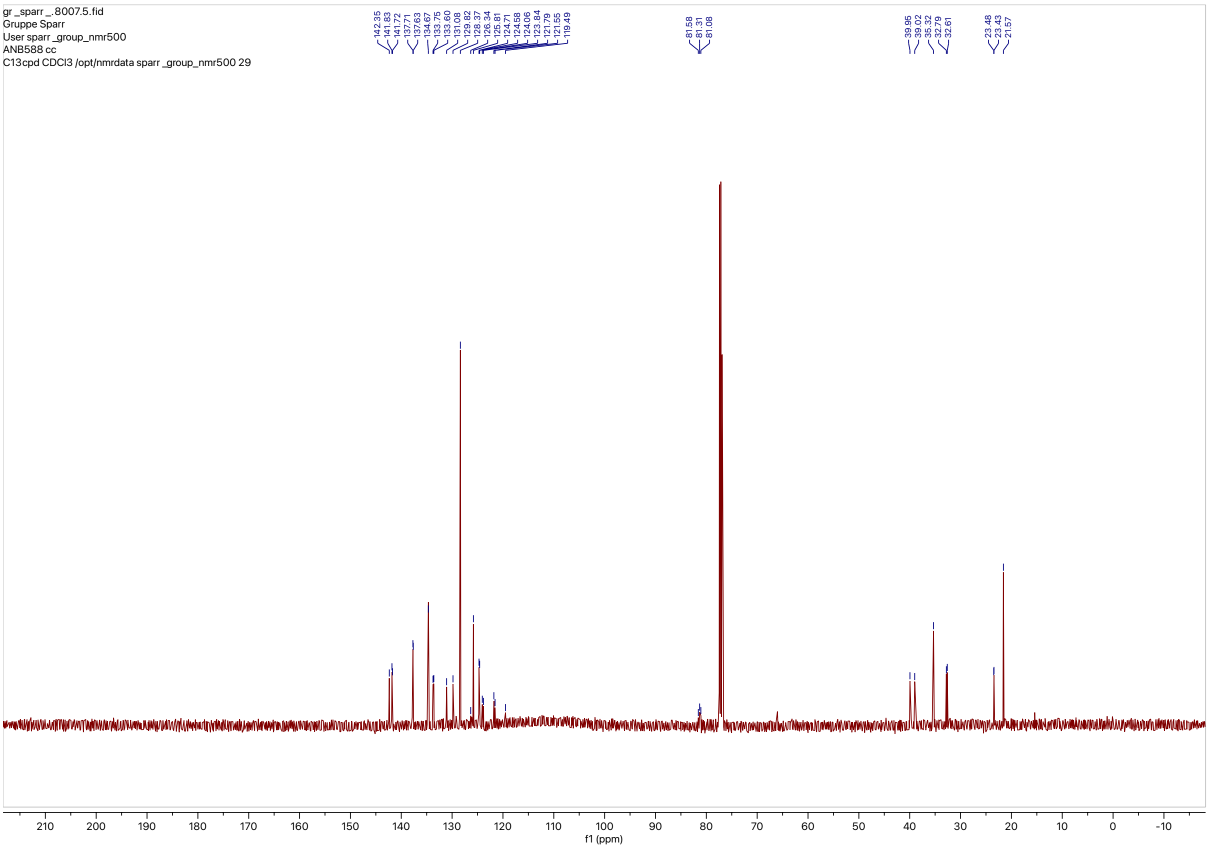

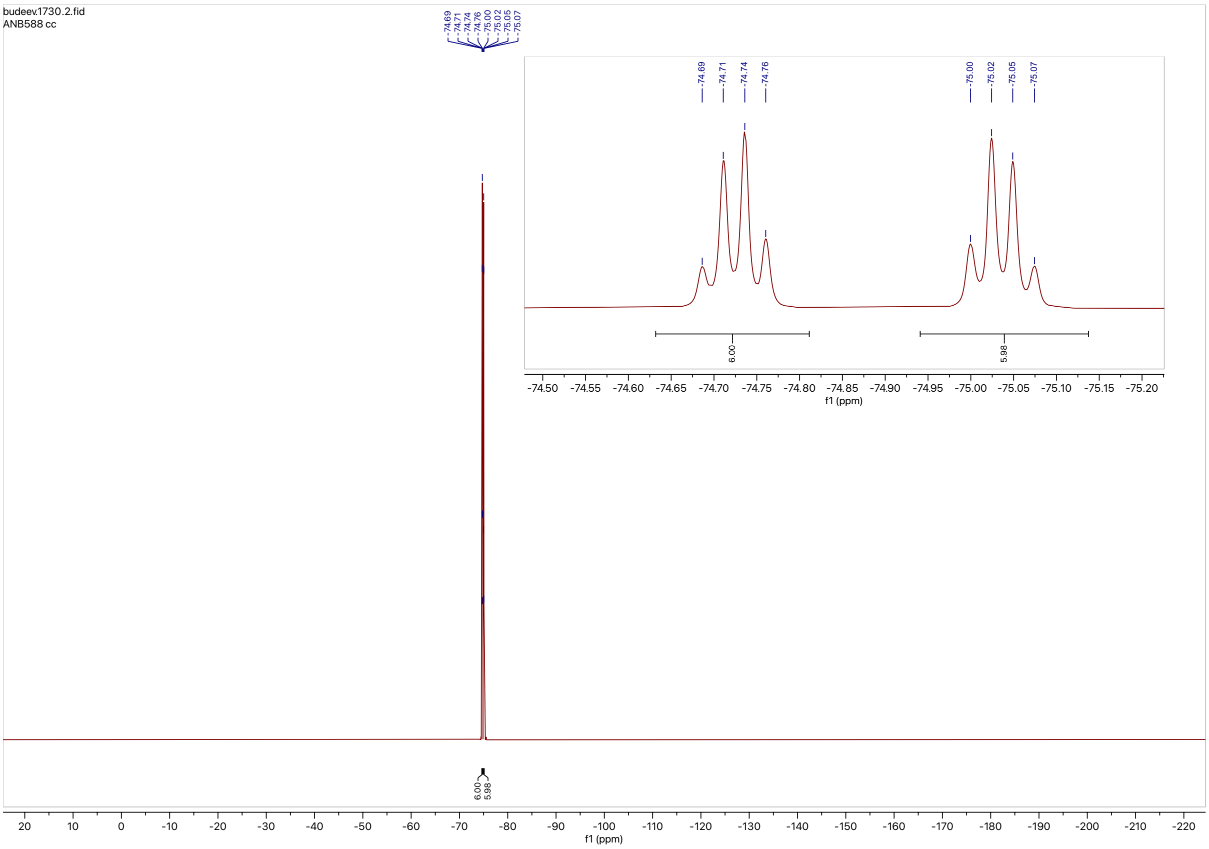

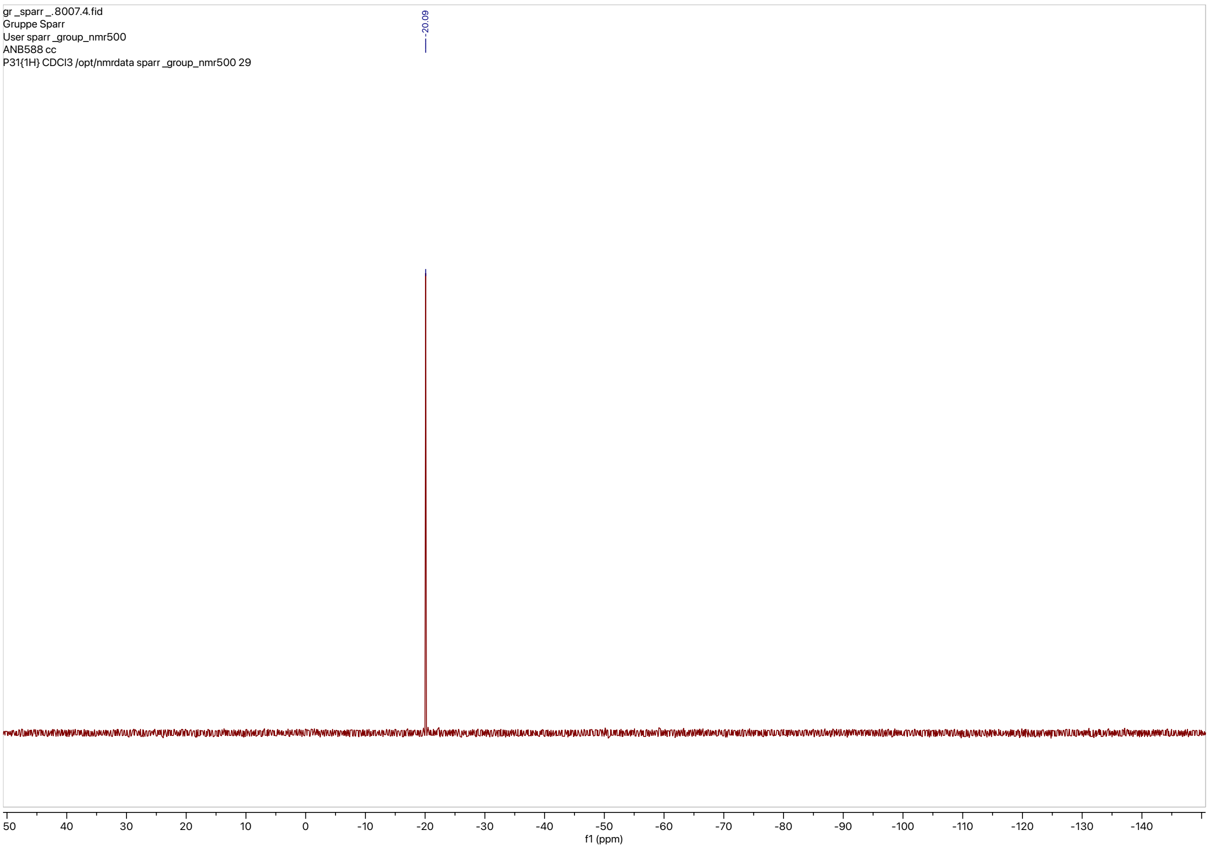

Supplementary Figure 75. ^{1}H (500 MHz, $CDCl_3$, 25 °C), ^{13}C{^{1}H} (126 MHz, $CDCl_3$, 25 °C), ^{19}F{^{1}H} (376 MHz, $CDCl_3$, 25 °C) and ^{31}P{^{1}H} (202 MHz, $CDCl_3$, 25 °C) spectra of (*S*-*trans*)-**2j**

^{1}H, $^{13}C\{^{1}H\}$, $^{19}F\{^{1}H\}$ and $^{31}P\{^{1}H\}$ NMR spectra of [*TBPY*-5-11'-*A*]-6,6'-Dimethyl-1-(pent-4-en-1-yl)-3,3,3',3'-tetrakis(trifluoromethyl)-3*H*,3'*H*-1λ^{5},1'-spirobi[benzo[*c*][2,1]oxaphosphole] ((*S*-*trans*)-2k)

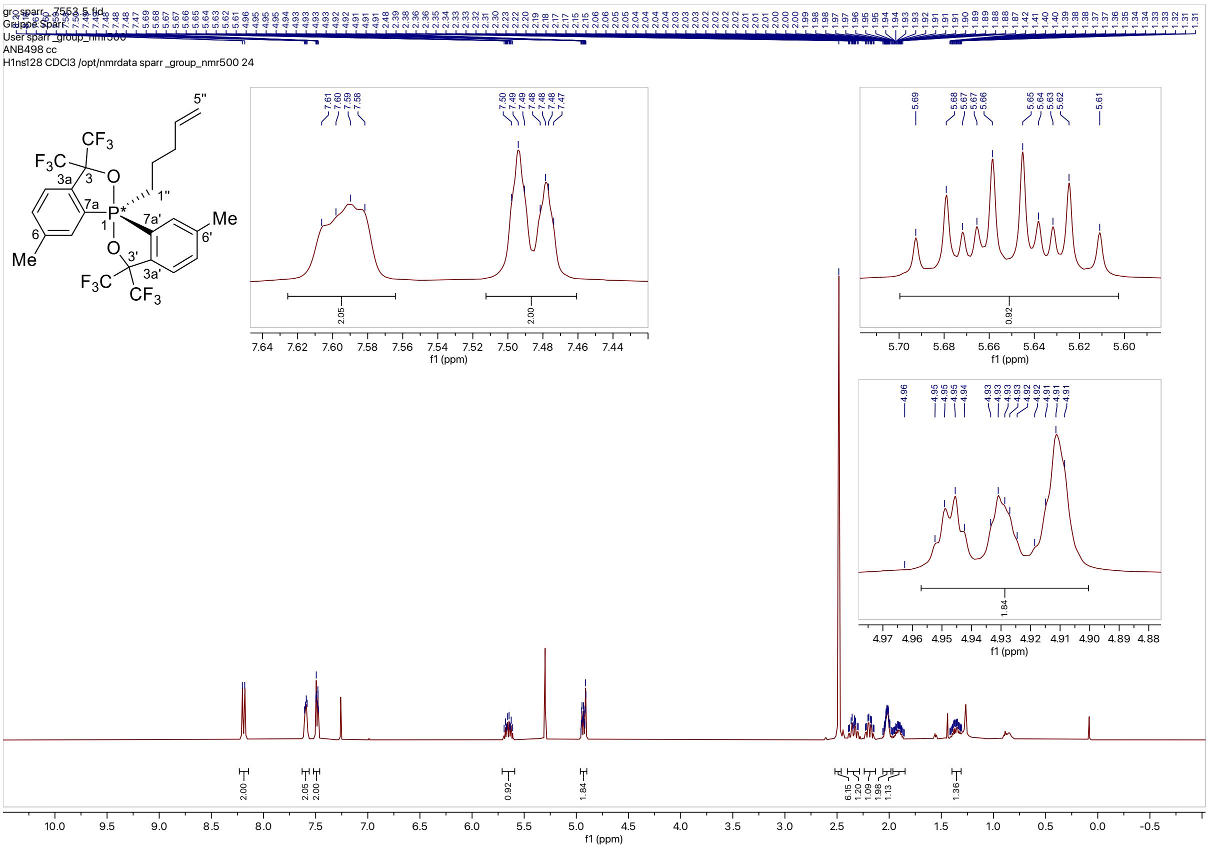

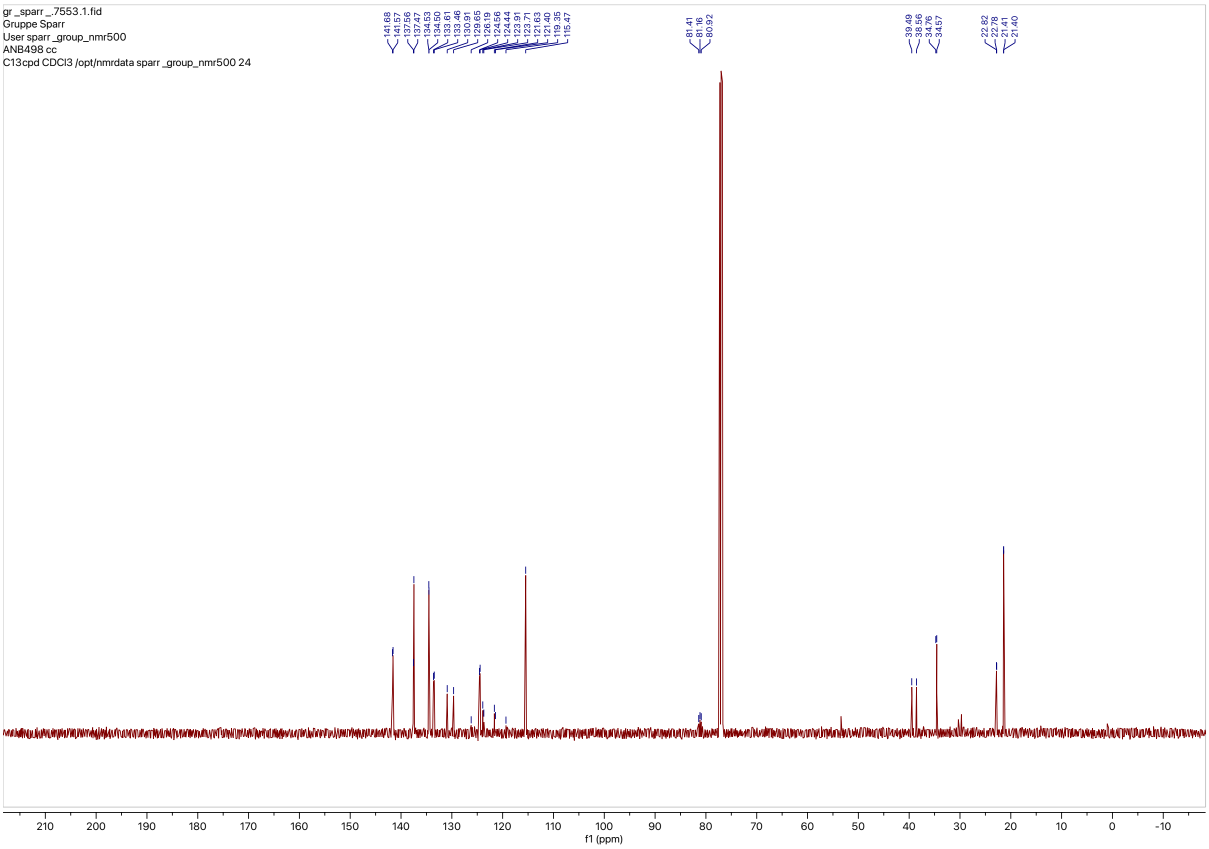

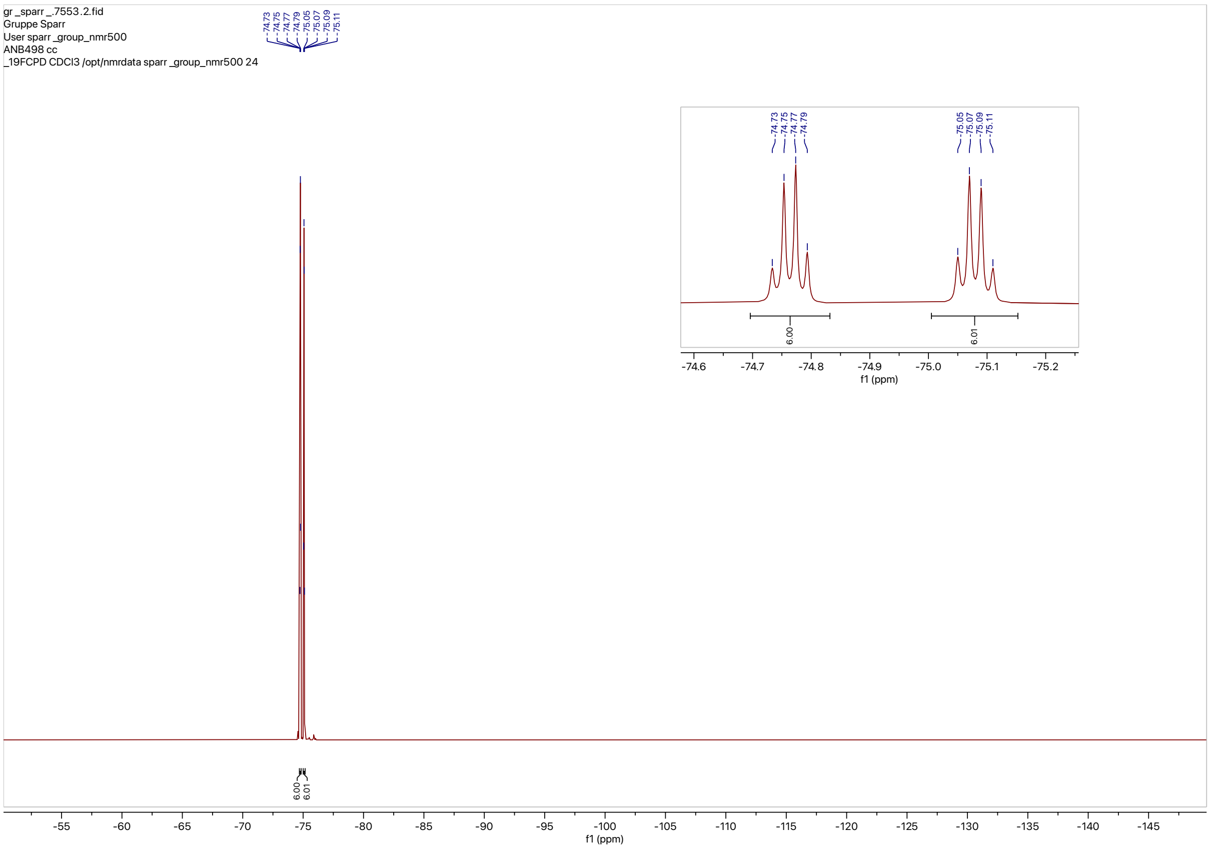

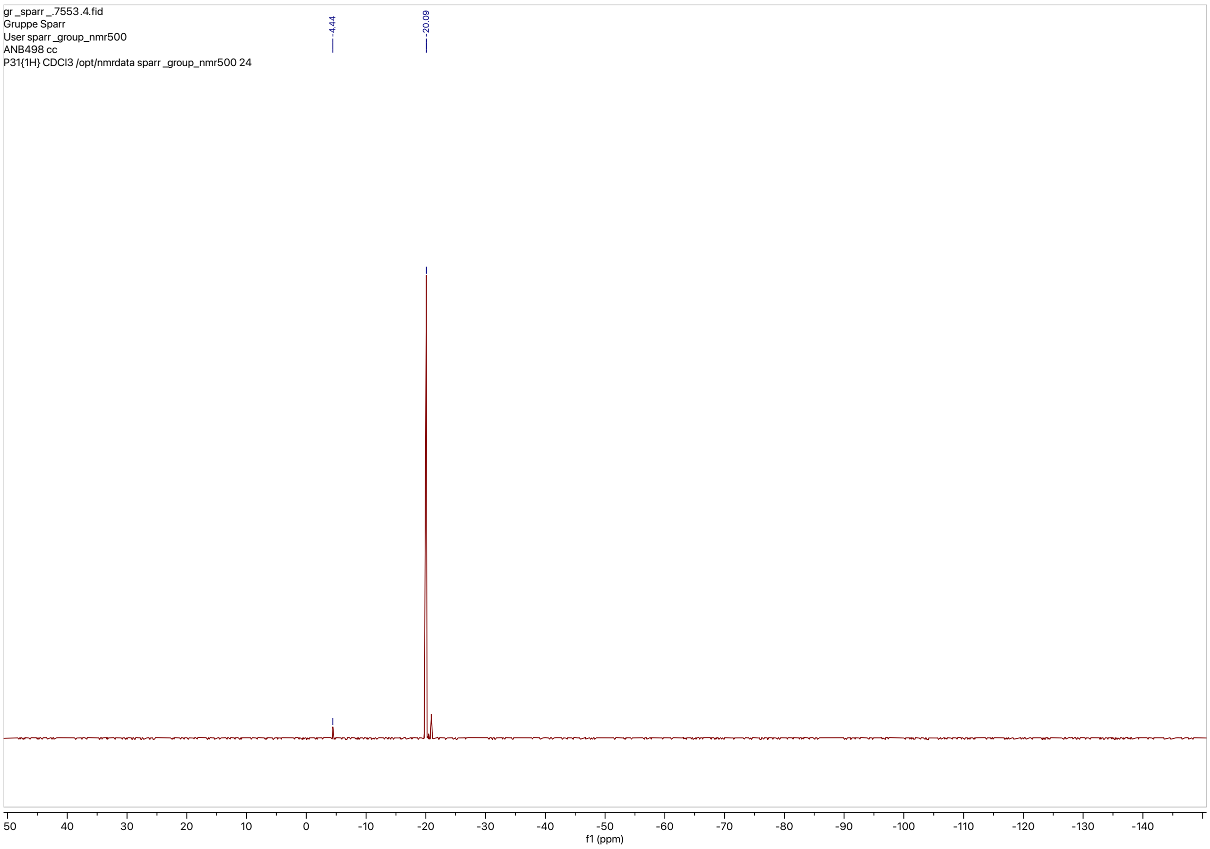

Supplementary Figure 76. ^{1}H (500 MHz, CDCl$_3$, 25 °C), ^{13}C{^{1}H} (126 MHz, CDCl$_3$, 25 °C), ^{19}F{^{1}H} (470 MHz, CDCl$_3$, 25 °C) and ^{31}P{^{1}H} (202 MHz, CDCl$_3$, 25 °C) spectra of (*S*-*trans*)-**2k**

^{1}H, ^{13}C{^{1}H}, ^{19}F{^{1}H} and ^{31}P{^{1}H} NMR spectra of [*TBPY*-5-11'-*A*]-6,6'-Dimethyl-1-phenethyl-3,3,3',3'-tetrakis(trifluoromethyl)-3*H*,3'*H*-1λ^{5},1'-spirobi[benzo[*c*][2,1]oxaphosphole] ((*S*-*trans*)-2l)

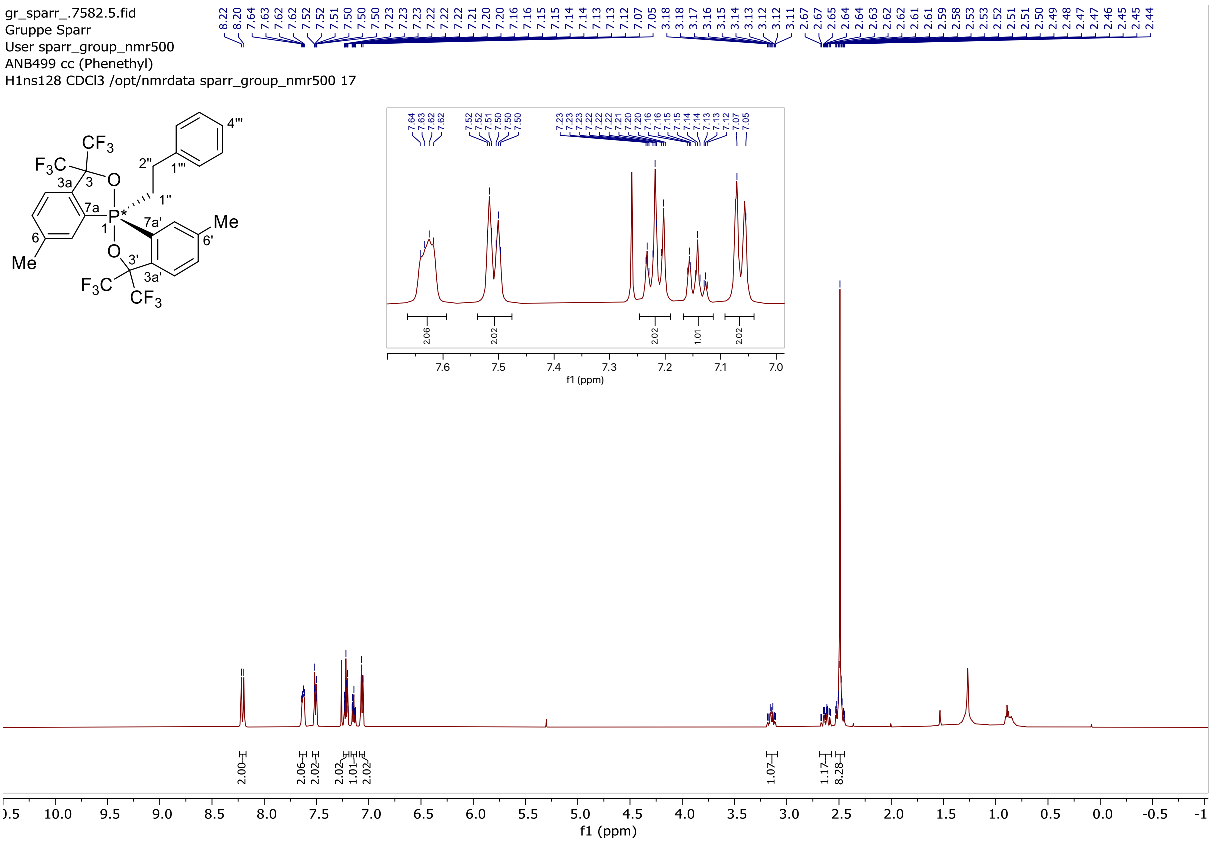

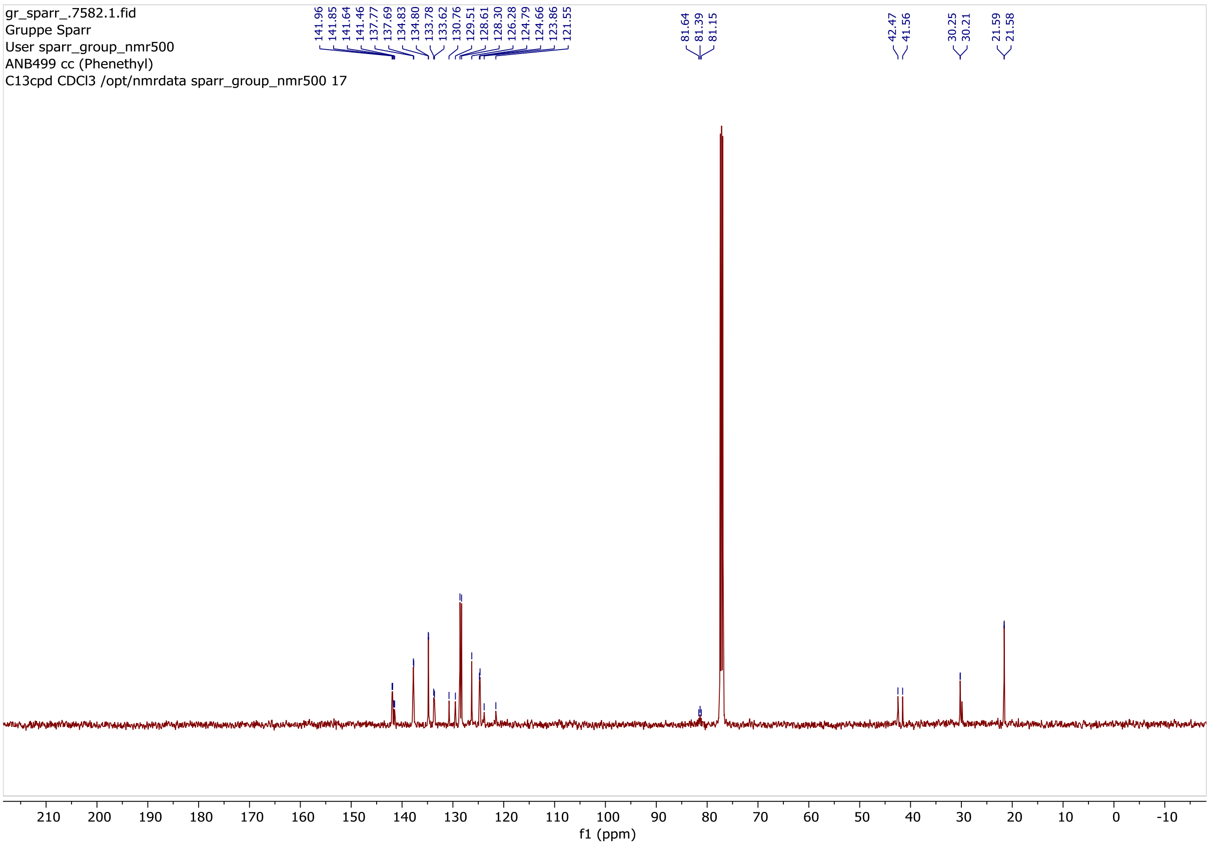

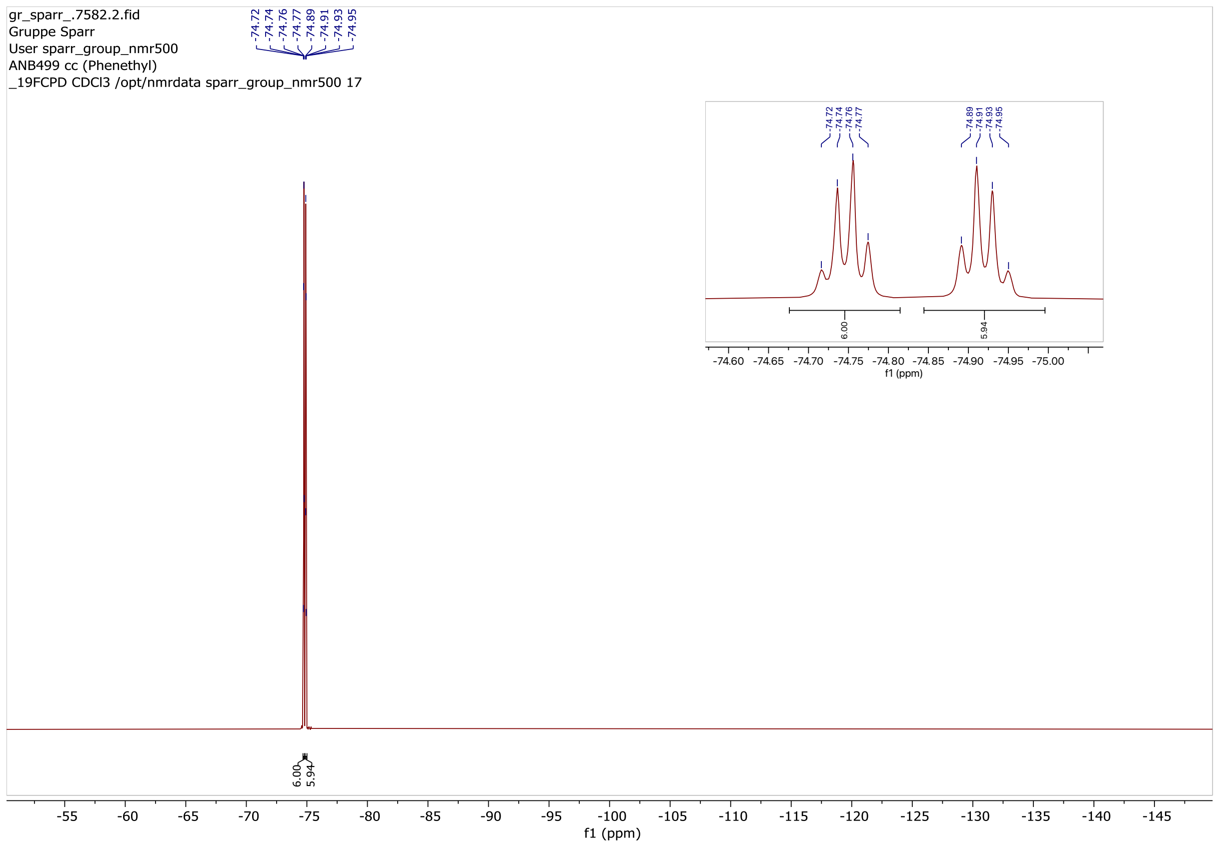

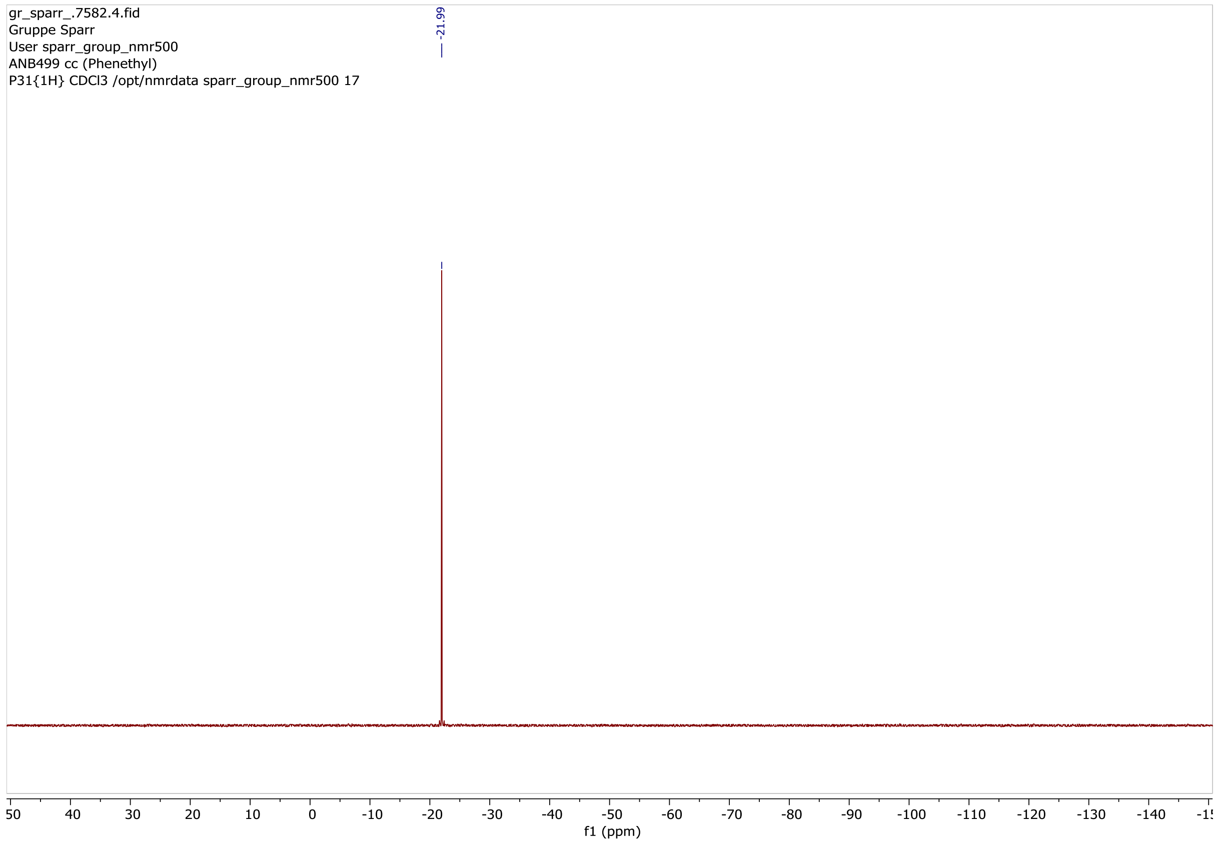

Supplementary Figure 77. ^{1}H (500 MHz, $CDCl_3$, 25 °C), $^{13}C\{^{1}H\}$ (126 MHz, $CDCl_3$, 25 °C), $^{19}F\{^{1}H\}$ (470 MHz, $CDCl_3$, 25 °C) and $^{31}P\{^{1}H\}$ (202 MHz, $CDCl_3$, 25 °C) spectra of (*S*-*trans*)-**2l**

^{1}H, $^{13}C\{^{1}H\}$, $^{19}F\{^{1}H\}$ and $^{31}P\{^{1}H\}$ NMR spectra of [*TBPY*-5-11'-*A*]-1,6,6'-Trimethyl-3,3,3',3'-tetrakis(trifluoromethyl)-3*H*,3'*H*-1λ^{5},1'-spirobi[benzo[*c*][2,1]oxaphosphole] ((*S*-*trans*)-2m)

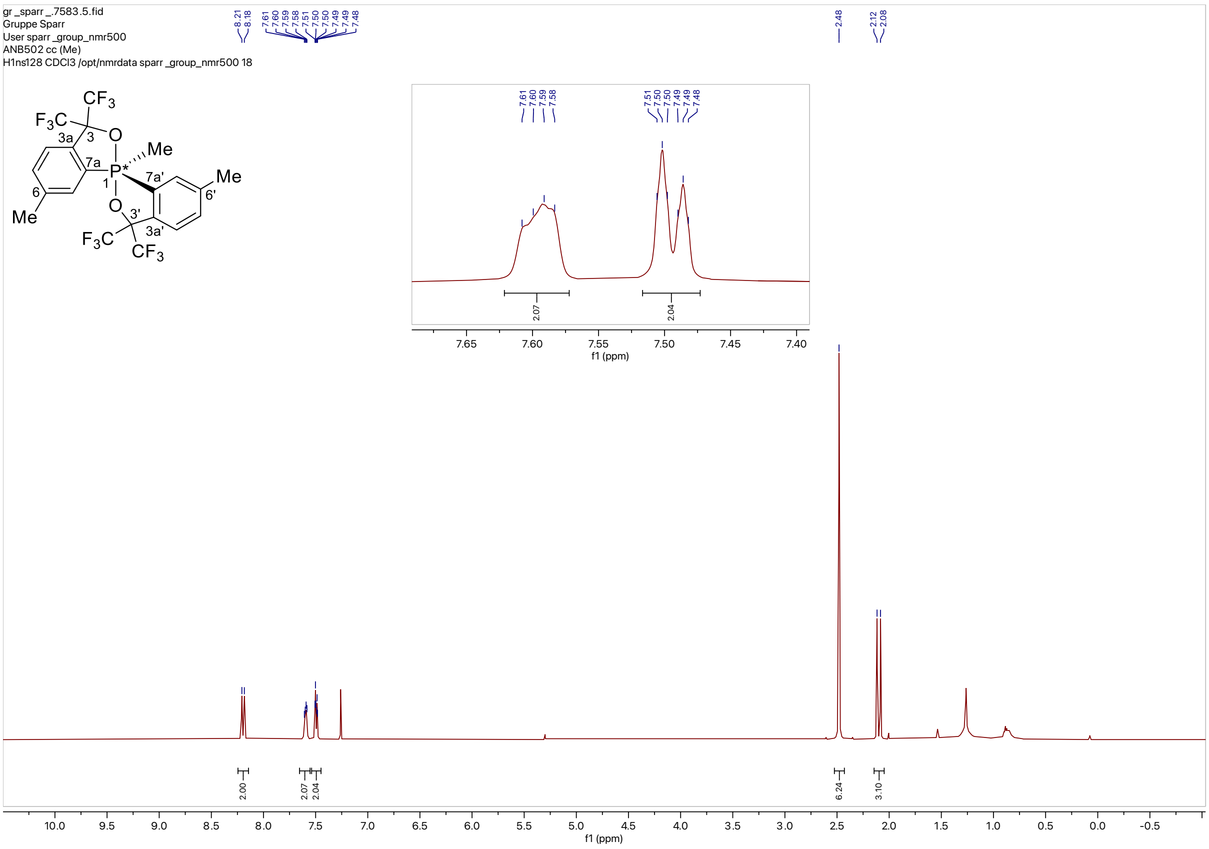

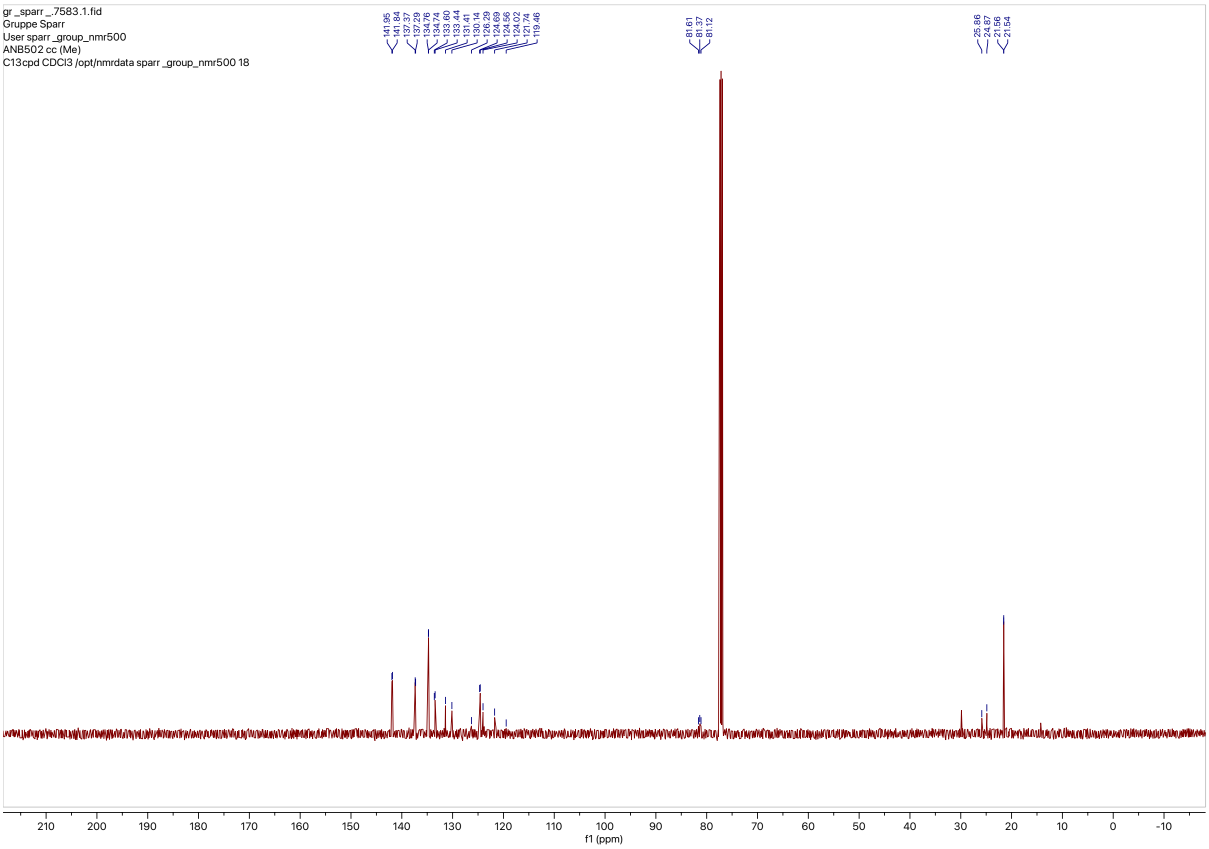

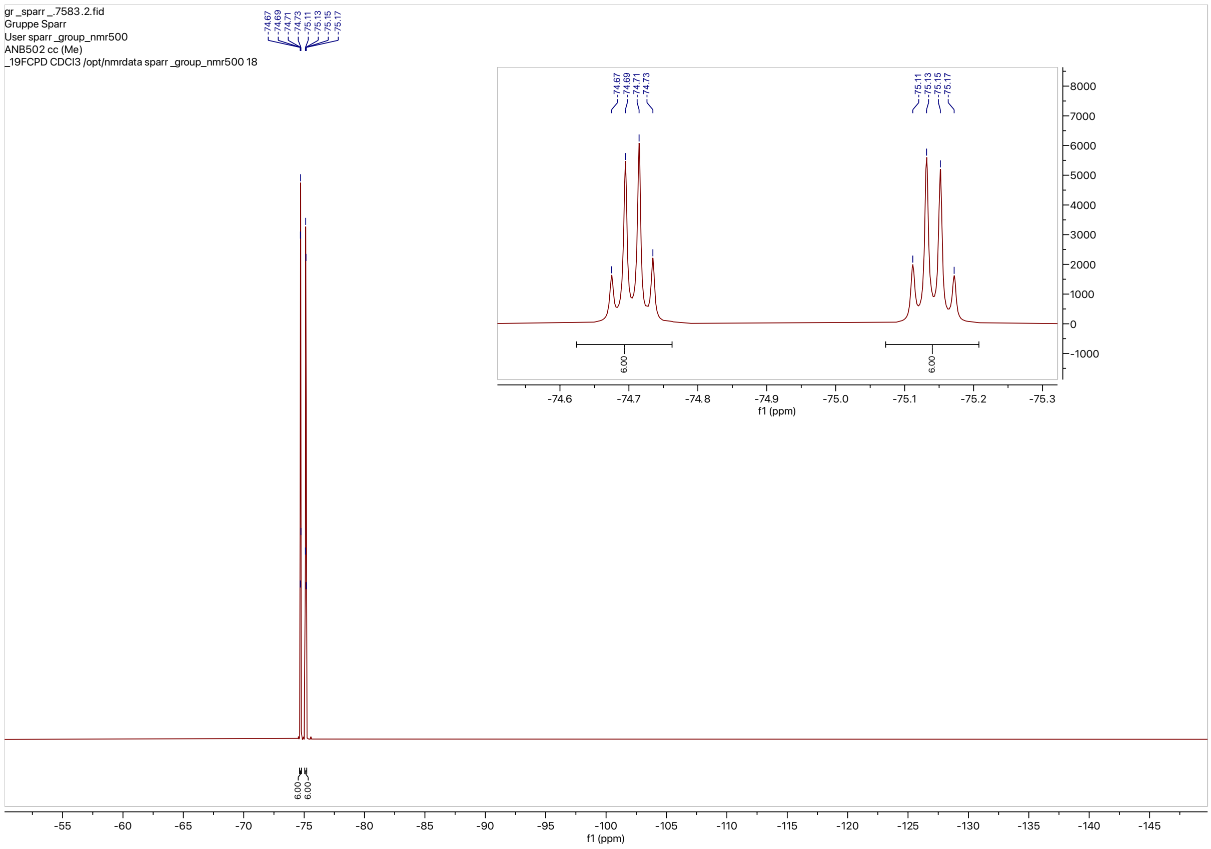

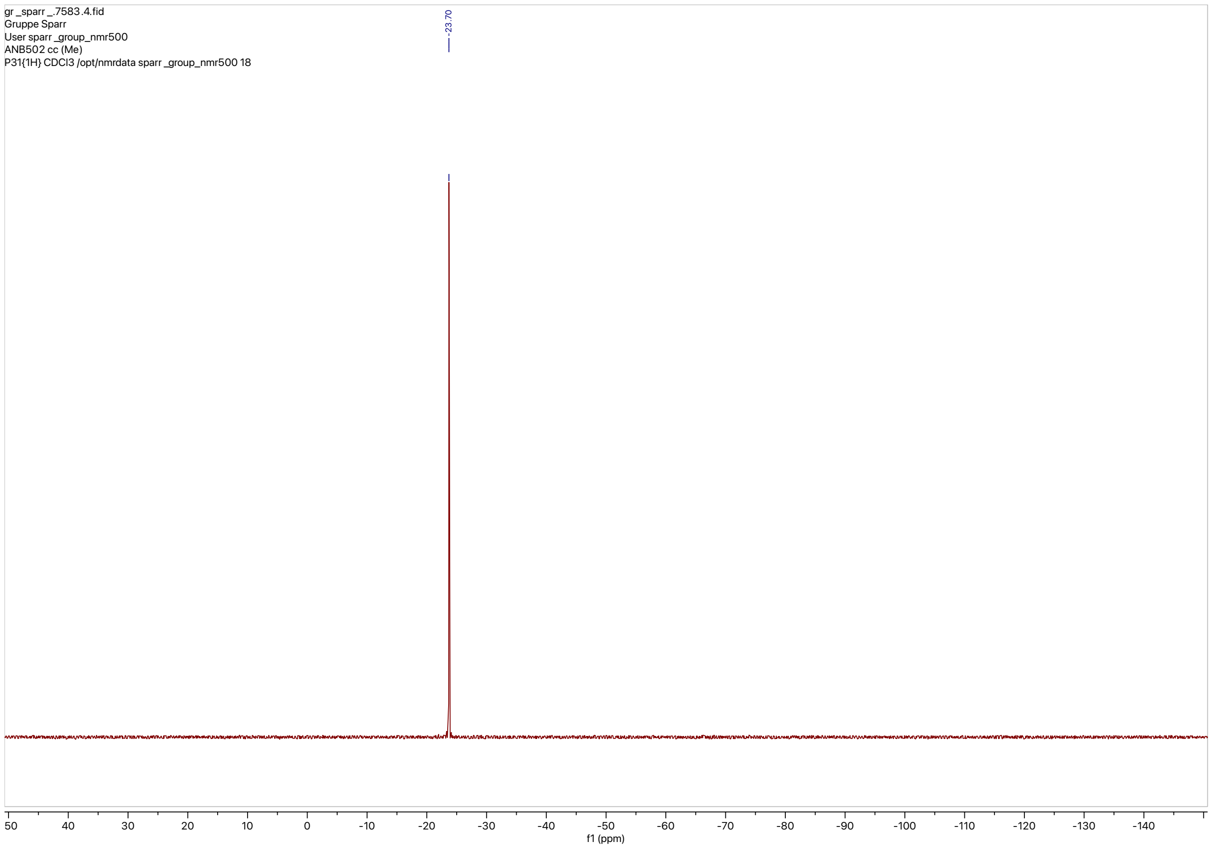

Supplementary Figure 78. ^{1}H (500 MHz, $CDCl_3$, 25 °C), ^{13}C{^{1}H} (126 MHz, $CDCl_3$, 25 °C), ^{19}F{^{1}H} (470 MHz, $CDCl_3$, 25 °C) and ^{31}P{^{1}H} (202 MHz, $CDCl_3$, 25 °C) spectra of (*S*-*trans*)-**2m**

[1]H, [13]C{[1]H}, ^{19}F{^{1}H} and ^{31}P{^{1}H} NMR spectra of [*TBPY*-5-11'-*A*]-6,6'-Dimethyl-3,3,3',3'-tetrakis(trifluoromethyl)-1-(6-(trimethylsi-lyl)hex-5-yn-1-yl)-3*H*,3'*H*-1λ[5],1'-spirobi[benzo[*c*][2,1]oxaphosphole] ((*S*-*trans*)-2n)

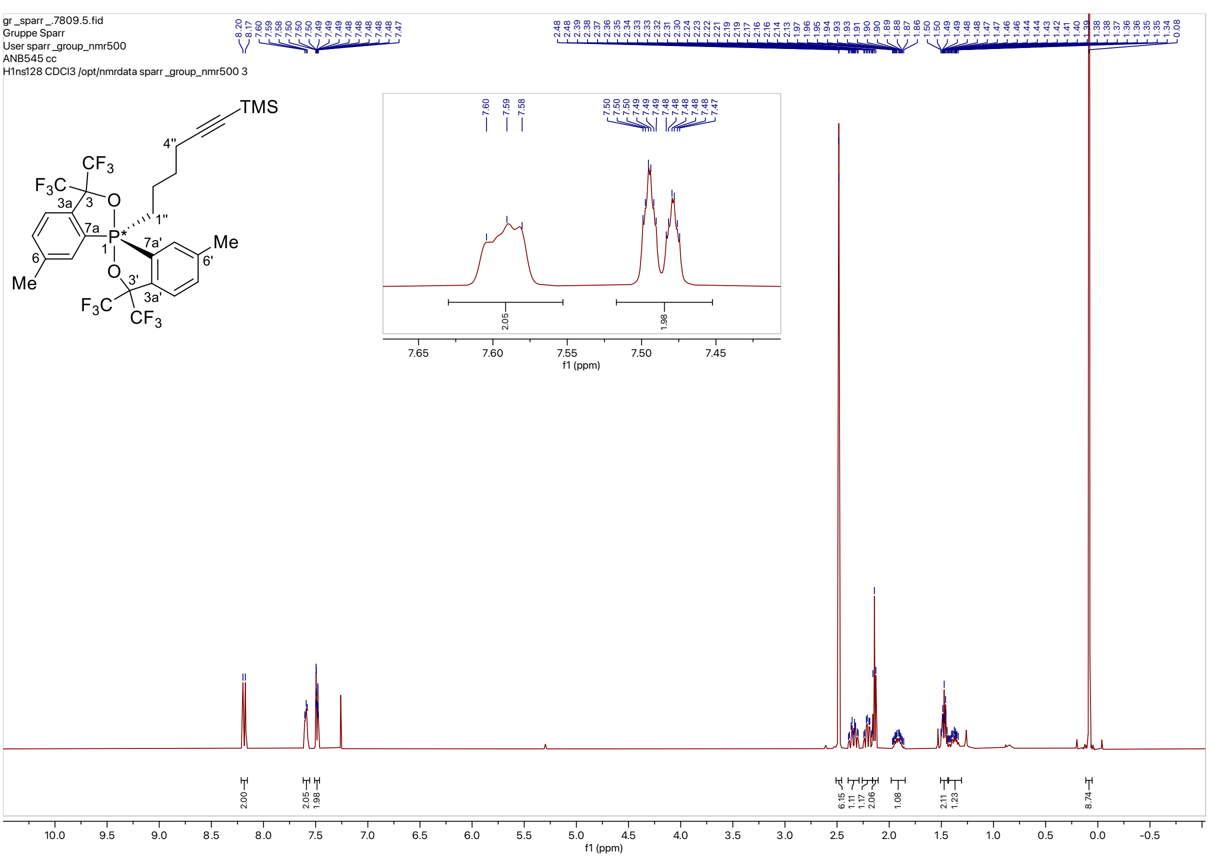

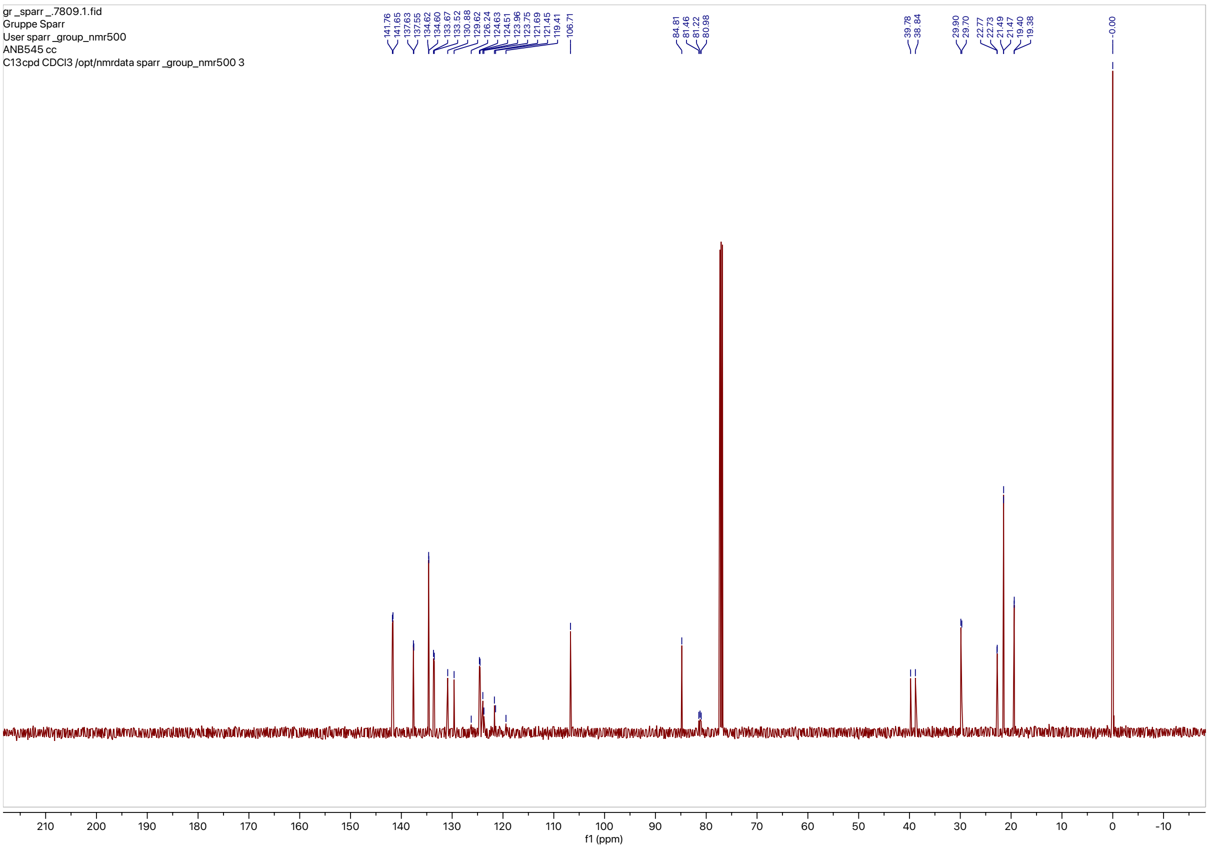

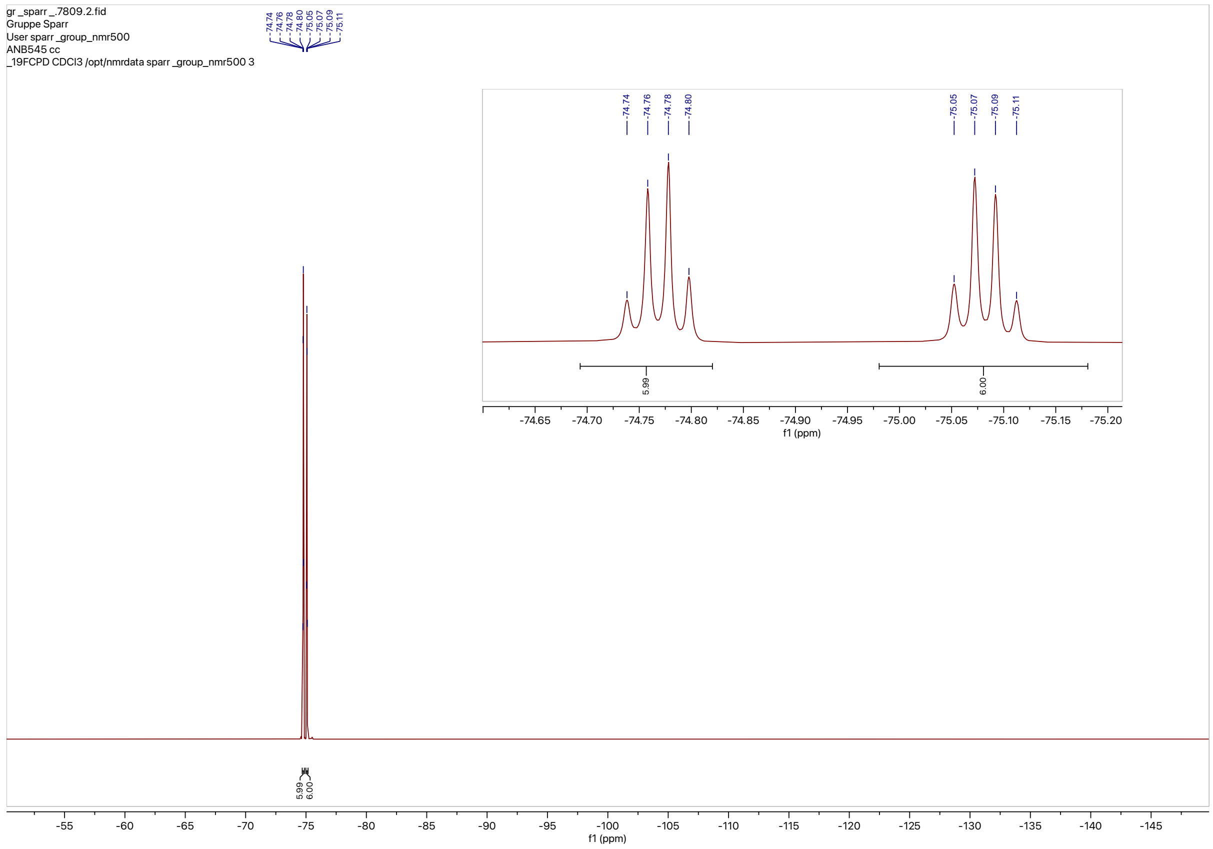

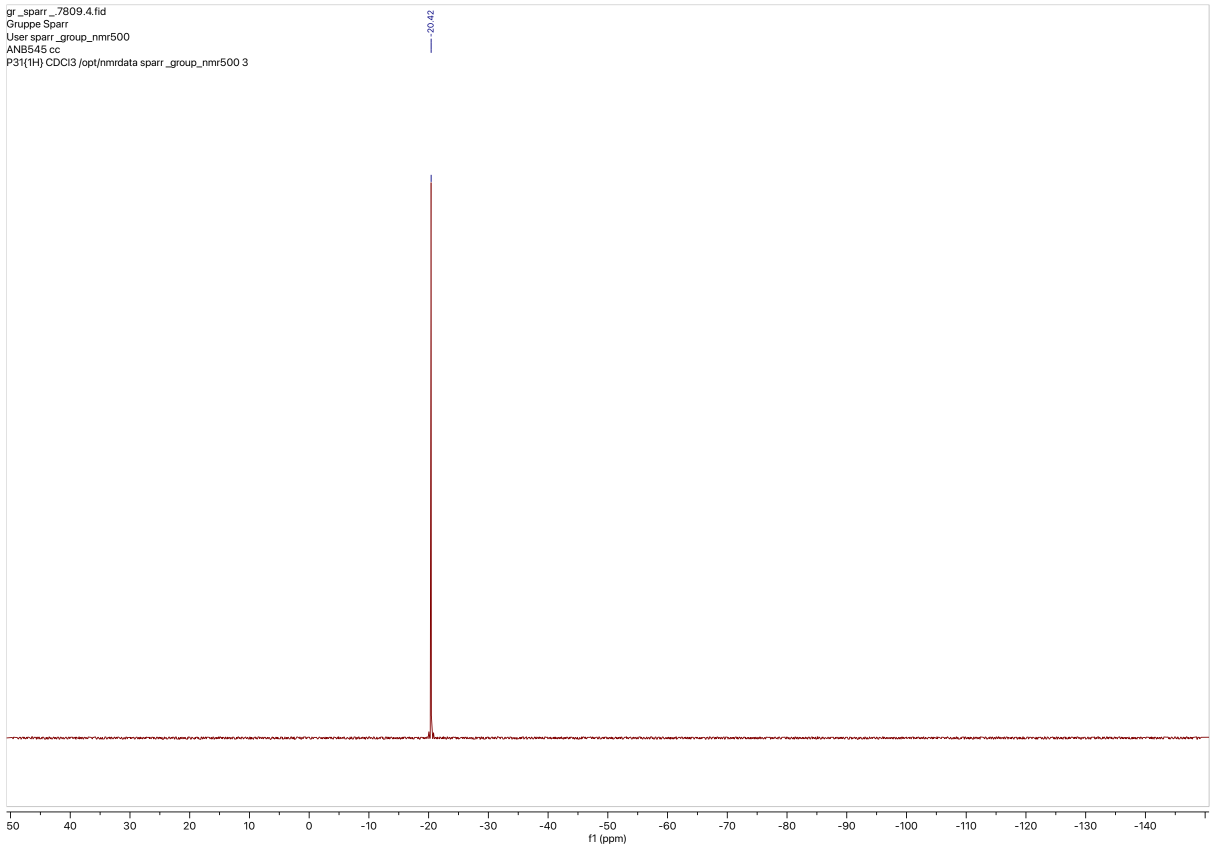

Supplementary Figure 79. ^{1}H (500 MHz, $CDCl_3$, 25 °C), ^{13}C{^{1}H} (126 MHz, $CDCl_3$, 25 °C), ^{19}F{^{1}H} (470 MHz, $CDCl_3$, 25 °C) and ^{31}P{^{1}H} (202 MHz, $CDCl_3$, 25 °C) spectra of (*S*-*trans*)-**2n**

[1]H, [13]C{[1]H}, ^{19}F{^{1}H} and ^{31}P{^{1}H} NMR spectra of [*TBPY*-5-11'-*A*]-1-(4-Methoxybutyl)-6,6'-dimethyl-3,3,3',3'-tetrakis(trifluoromethyl)-1-(6-(trimethylsi-lyl)hex-5-yn-1-yl)-3*H*,3'*H*-1λ[5],1'-spirobi[benzo[*c*][2,1]oxaphosphole] ((*S*-*trans*)-2o)

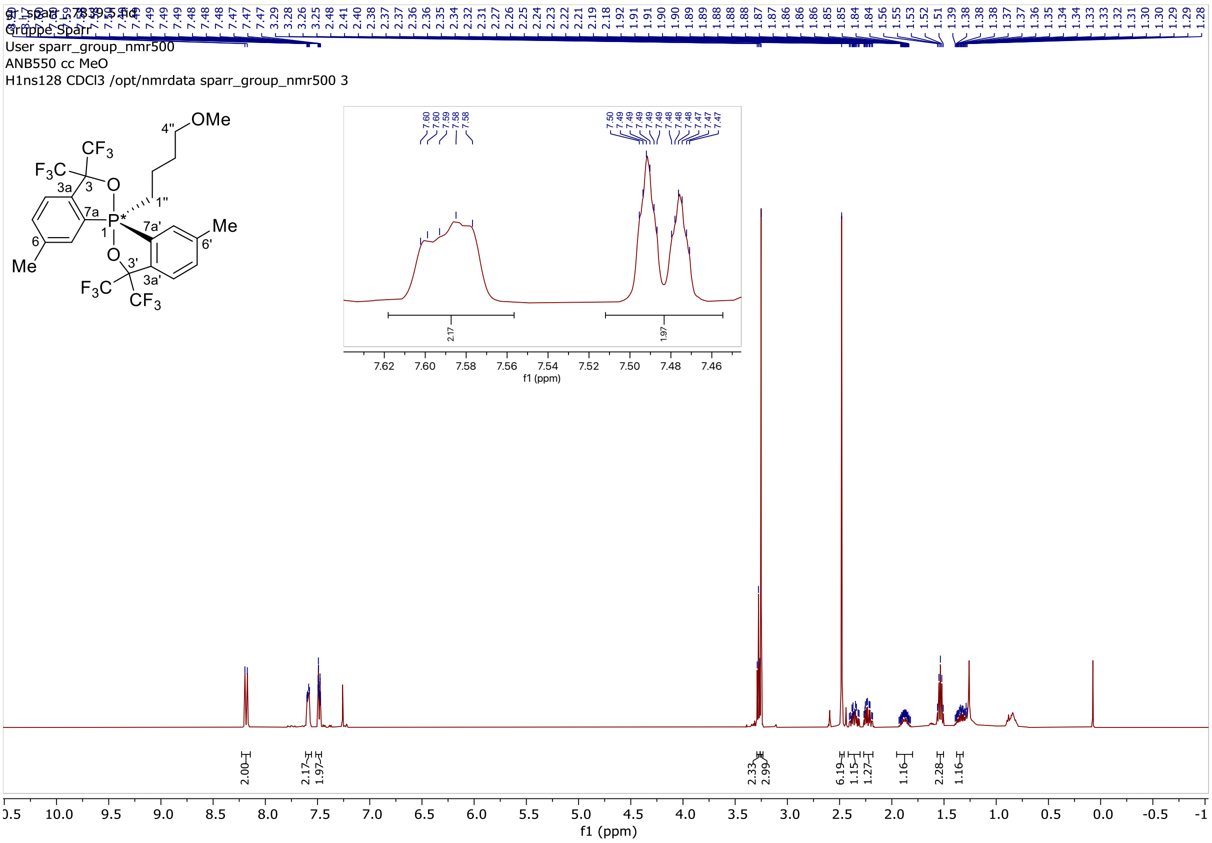

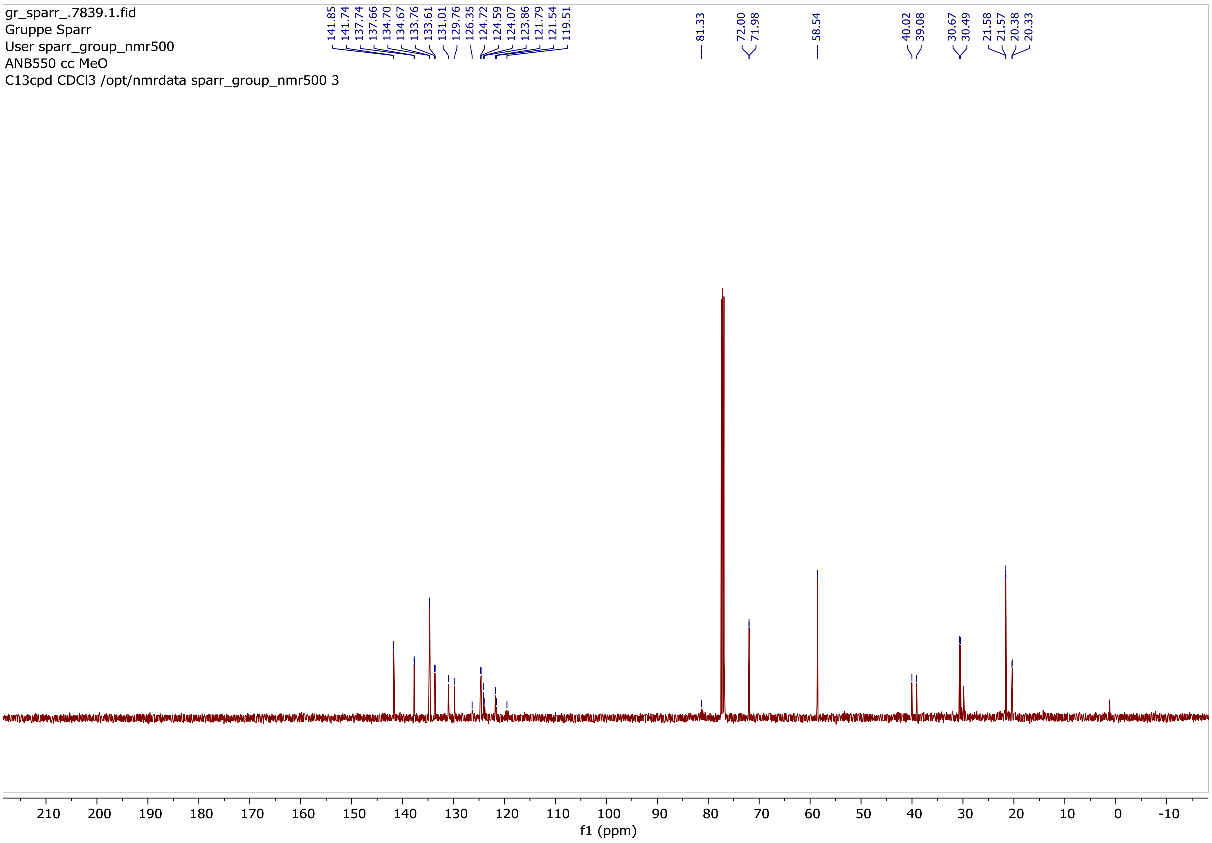

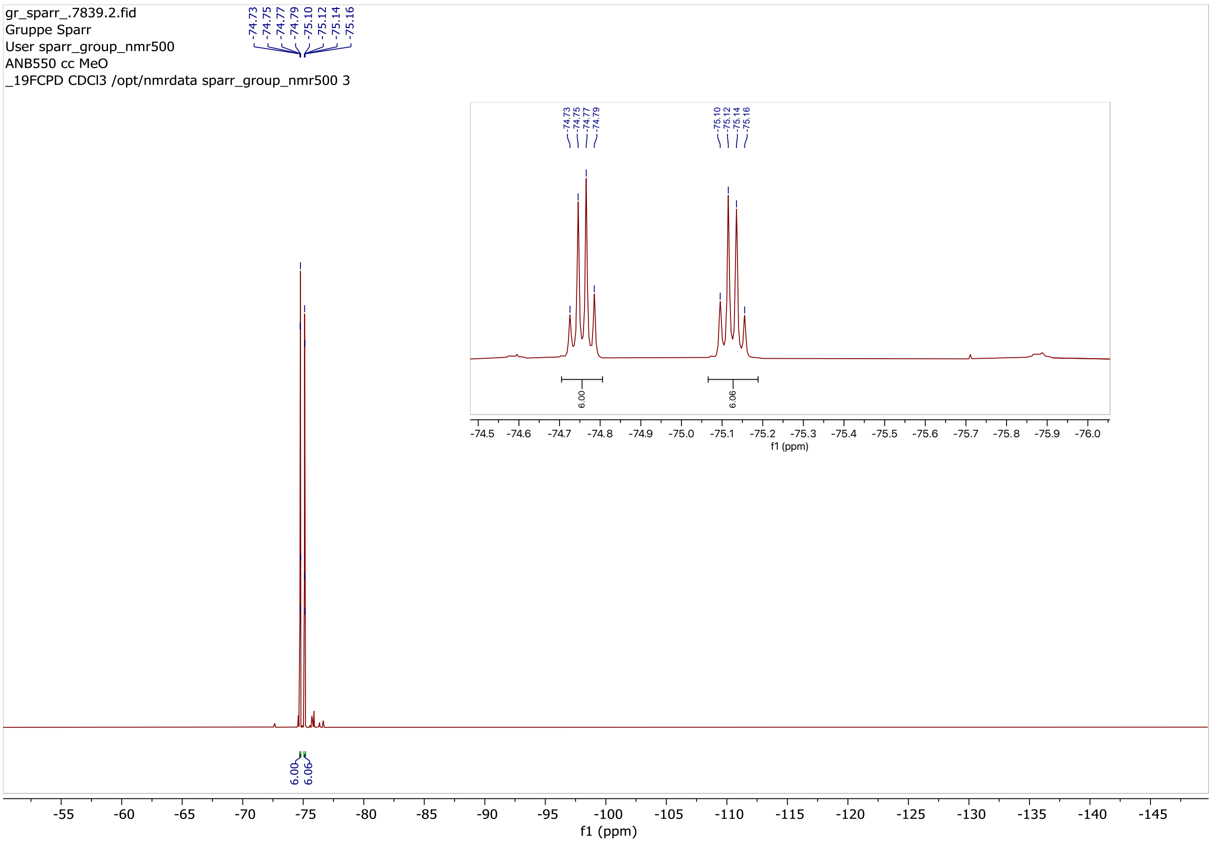

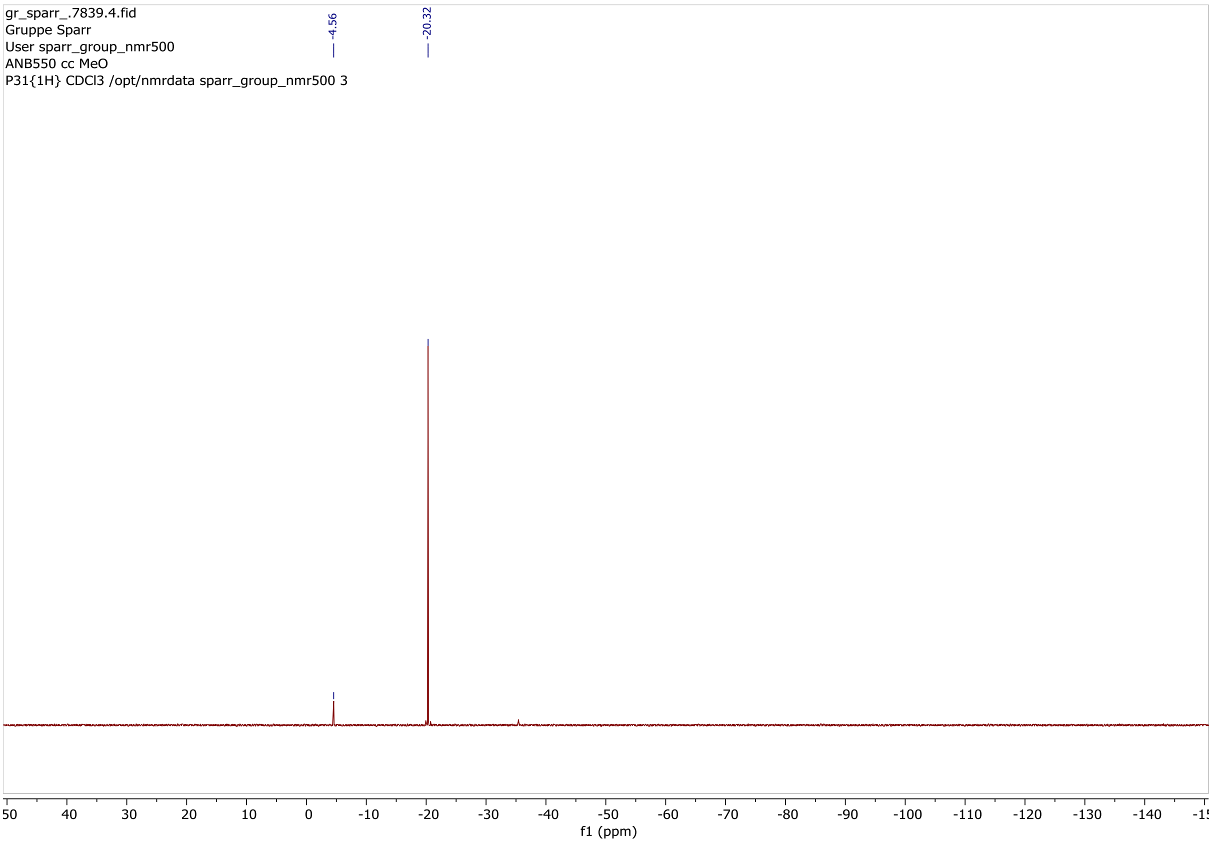

Supplementary Figure 80. ^{1}H (500 MHz, $CDCl_3$, 25 °C), ^{13}C{^{1}H} (126 MHz, $CDCl_3$, 25 °C), ^{19}F{^{1}H} (470 MHz, $CDCl_3$, 25 °C) and ^{31}P{^{1}H} (202 MHz, $CDCl_3$, 25 °C) spectra of (*S*-*trans*)-**2o**

^{1}H, ^{13}C{^{1}H}, ^{19}F{^{1}H} and ^{31}P{^{1}H} NMR spectra of [*TBPY*-5-11'-*A*]-1-(4-(1,3-Dioxolan-2-yl)butyl)-6,6'-dime-thyl-3,3,3',3'-tetrakis(trifluoromethyl)-3*H*,3'*H*-1λ^{5},1'-spirobi[benzo[*c*][2,1]oxaphosphole] ((*S*-*trans*)-2p)

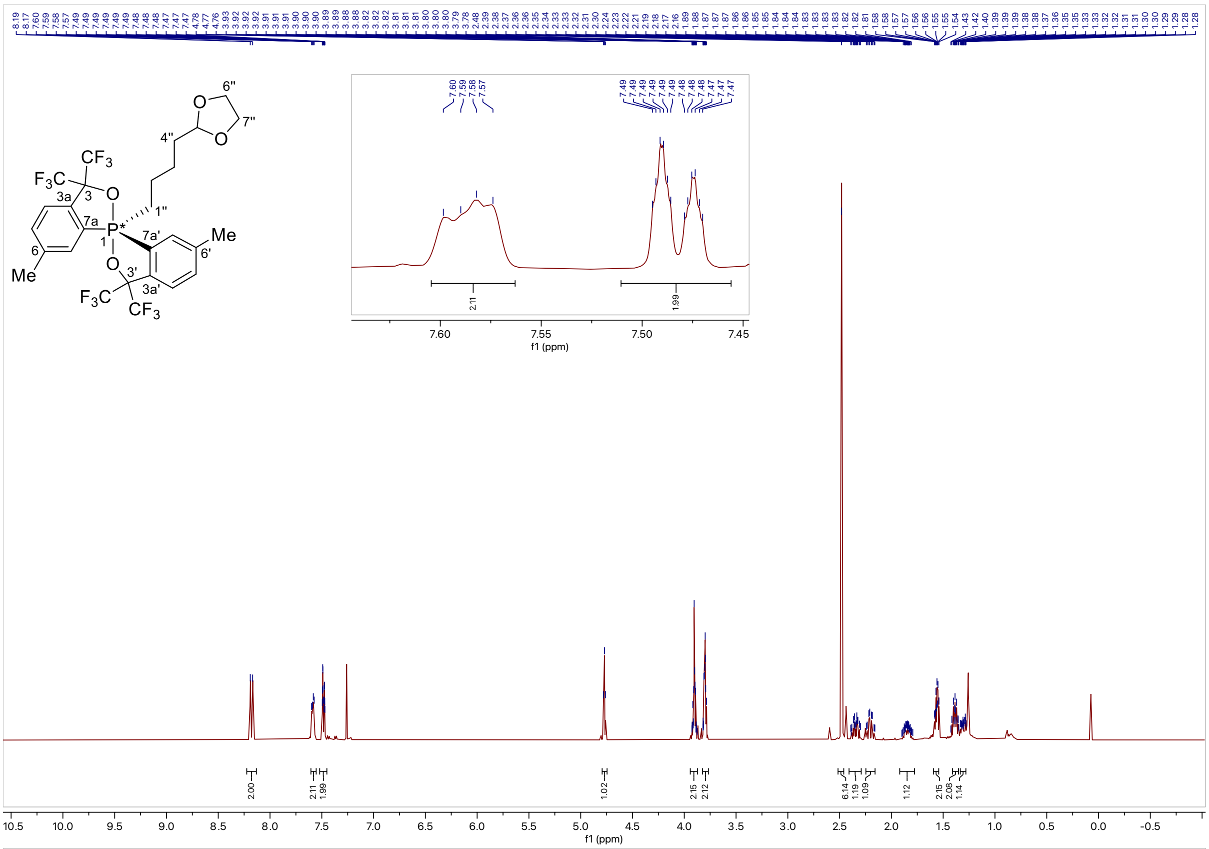

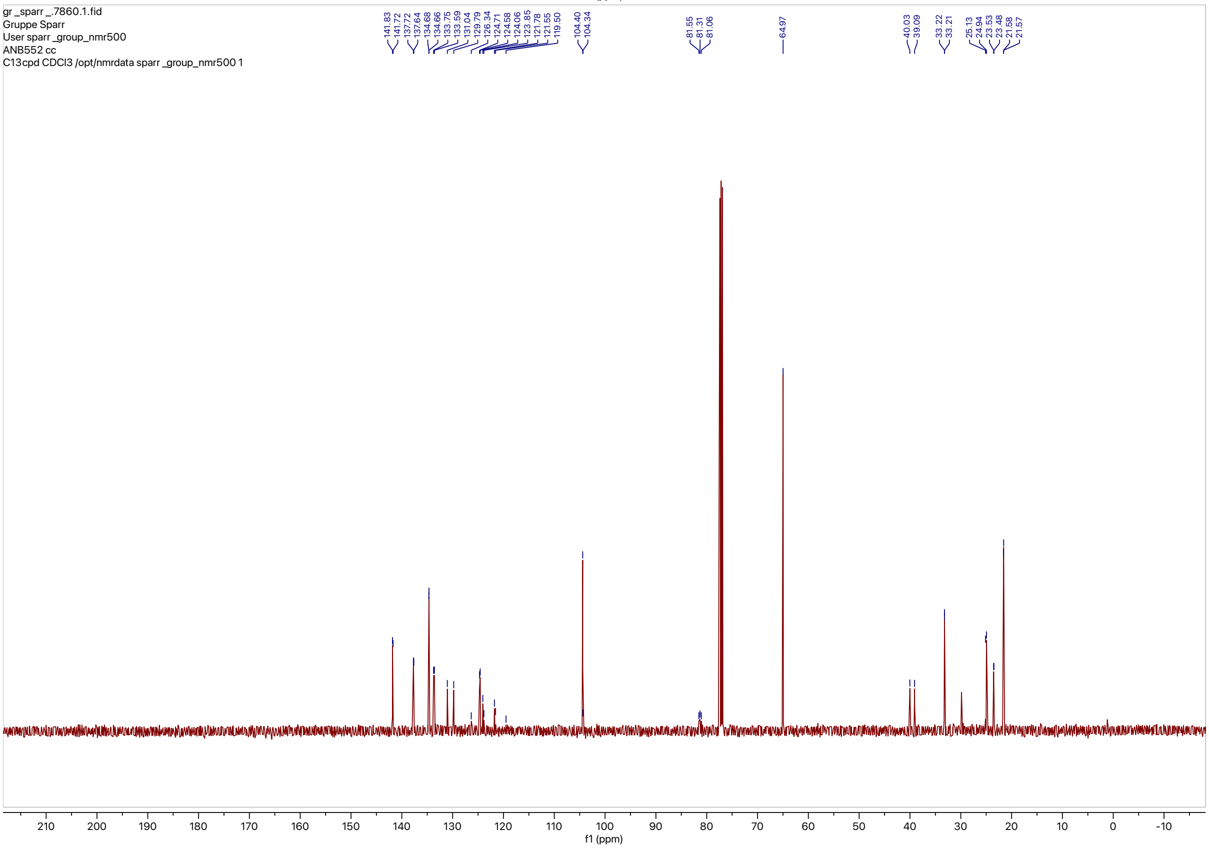

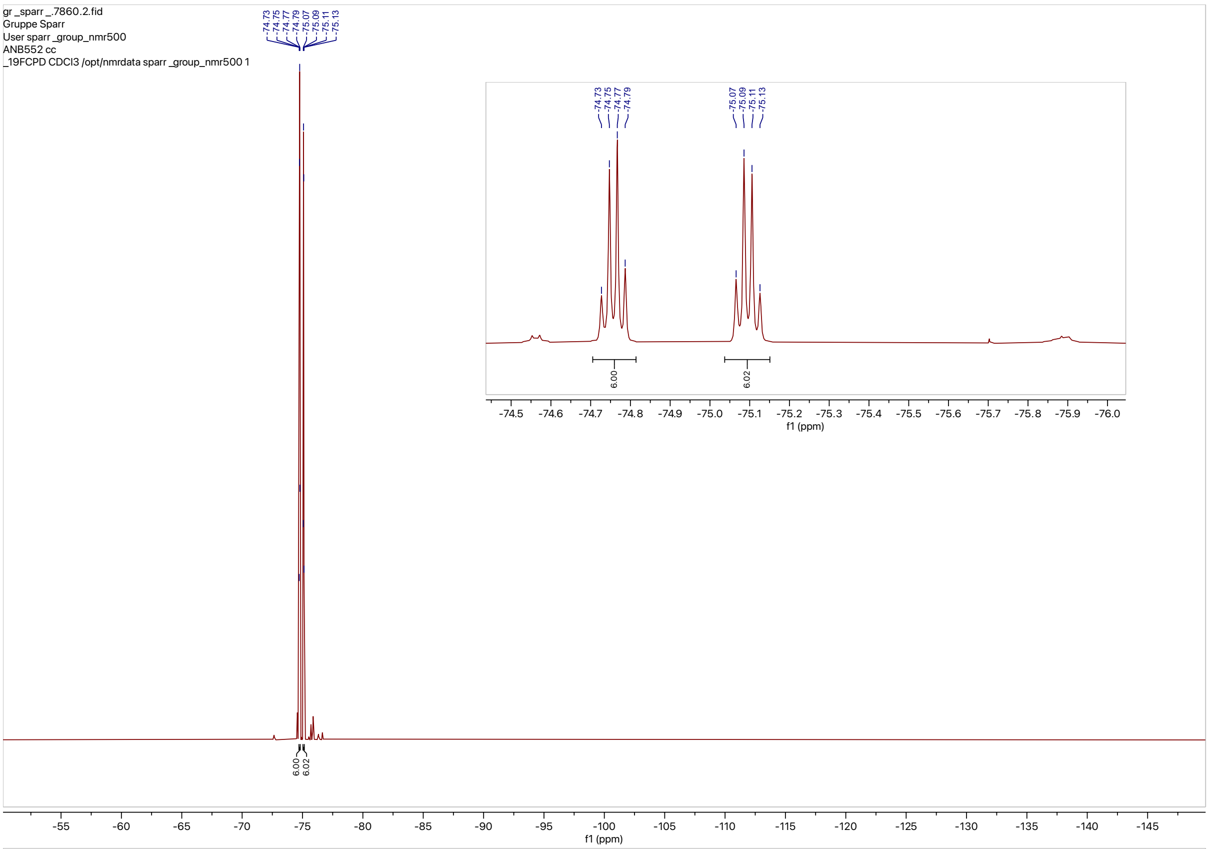

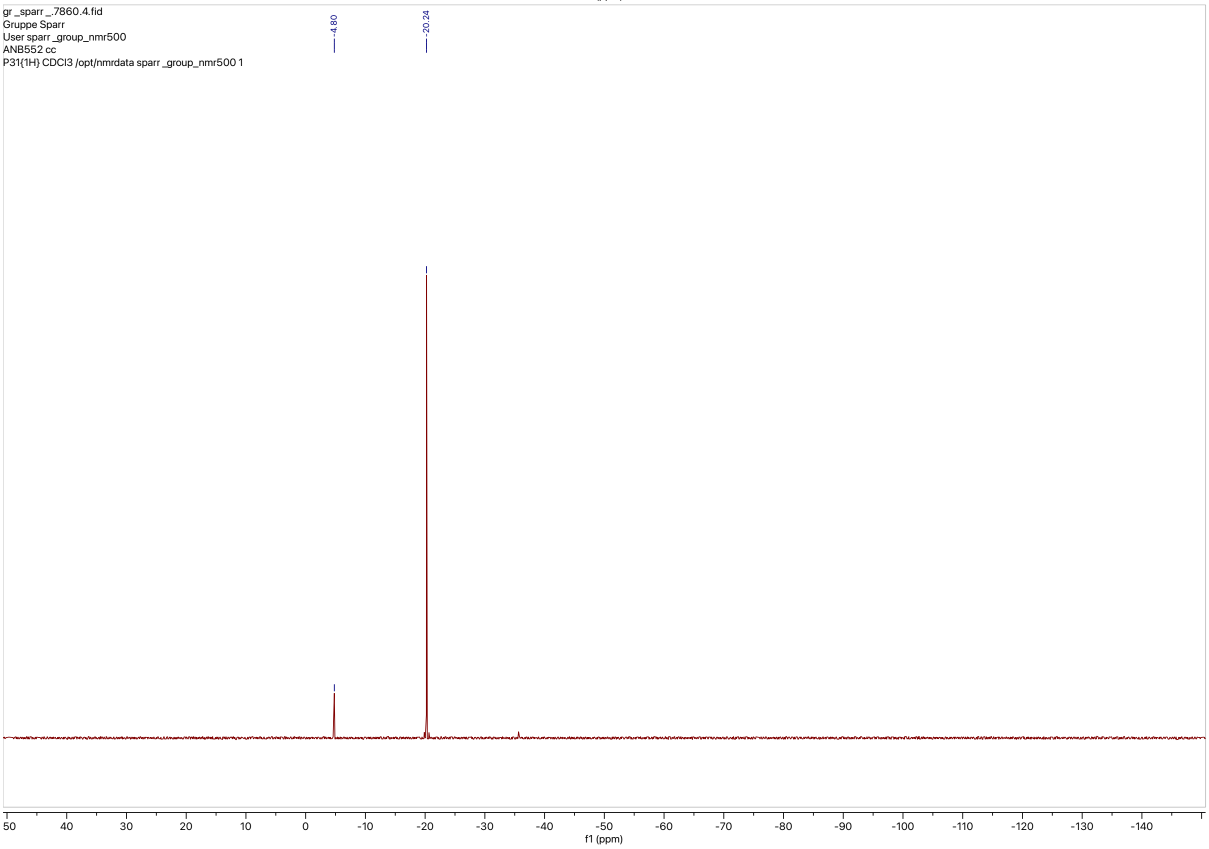

Supplementary Figure 81. ^{1}H (500 MHz, $CDCl_3$, 25 °C), ^{13}C{^{1}H} (126 MHz, $CDCl_3$, 25 °C), ^{19}F{^{1}H} (470 MHz, $CDCl_3$, 25 °C) and ^{31}P{^{1}H} (202 MHz, $CDCl_3$, 25 °C) spectra of (*S*-*trans*)-**2p**

[1]H, [13]C{[1]H}, ^{19}F{^{1}H} and ^{31}P{^{1}H} NMR spectra of [*TBPY*-5-12]-1-Butyl-6,6'-dimethyl-3,3,3',3'-tetrakis(trifluoromethyl)-3*H*,3'*H*-1λ[5],1'-spirobi[benzo[*c*][2,1]oxaphosphole] ((*cis*)-2a)

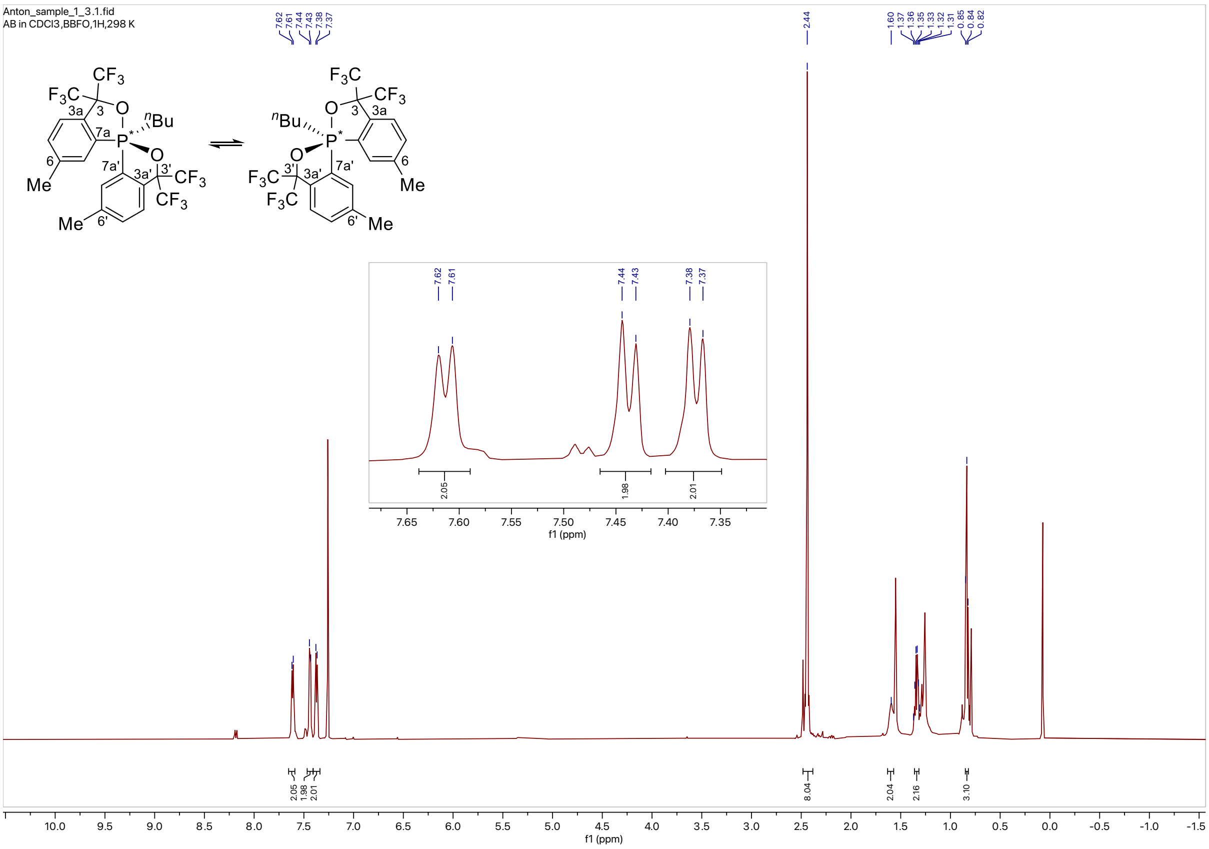

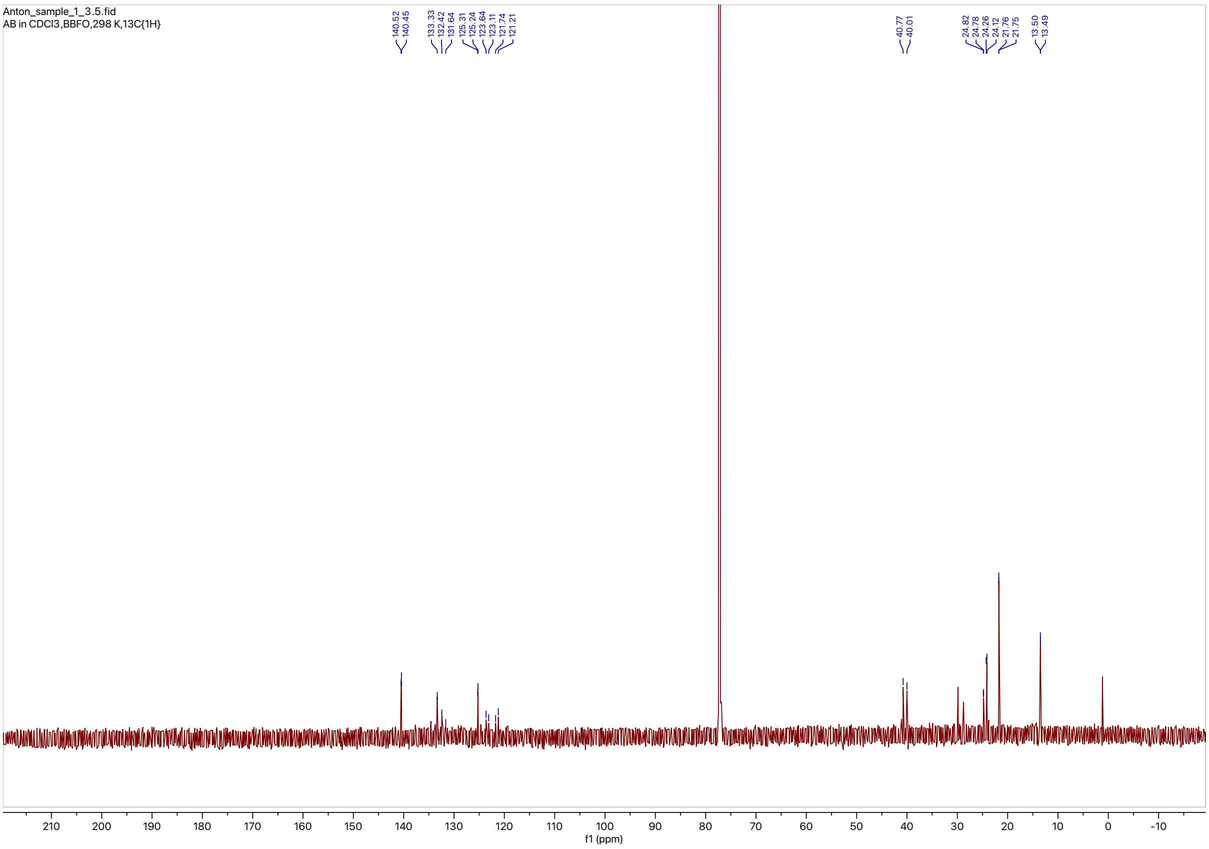

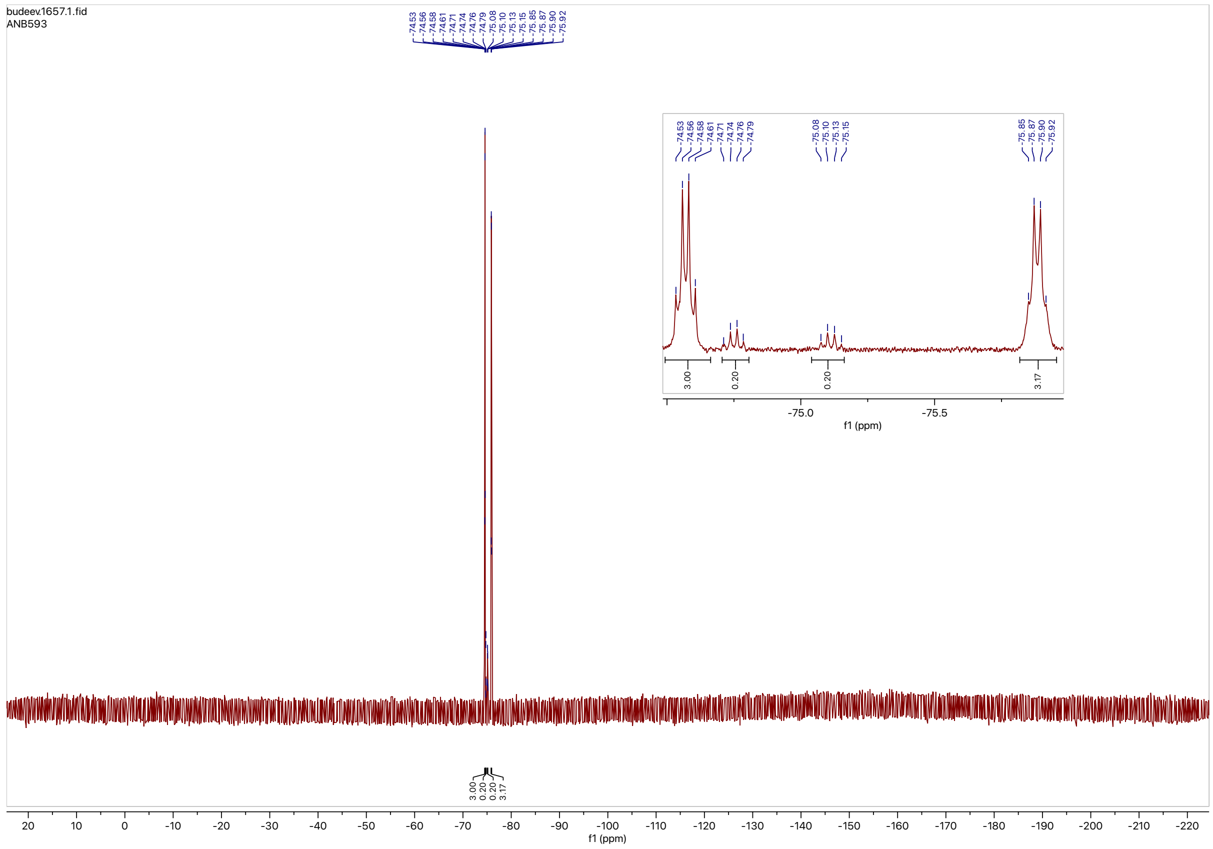

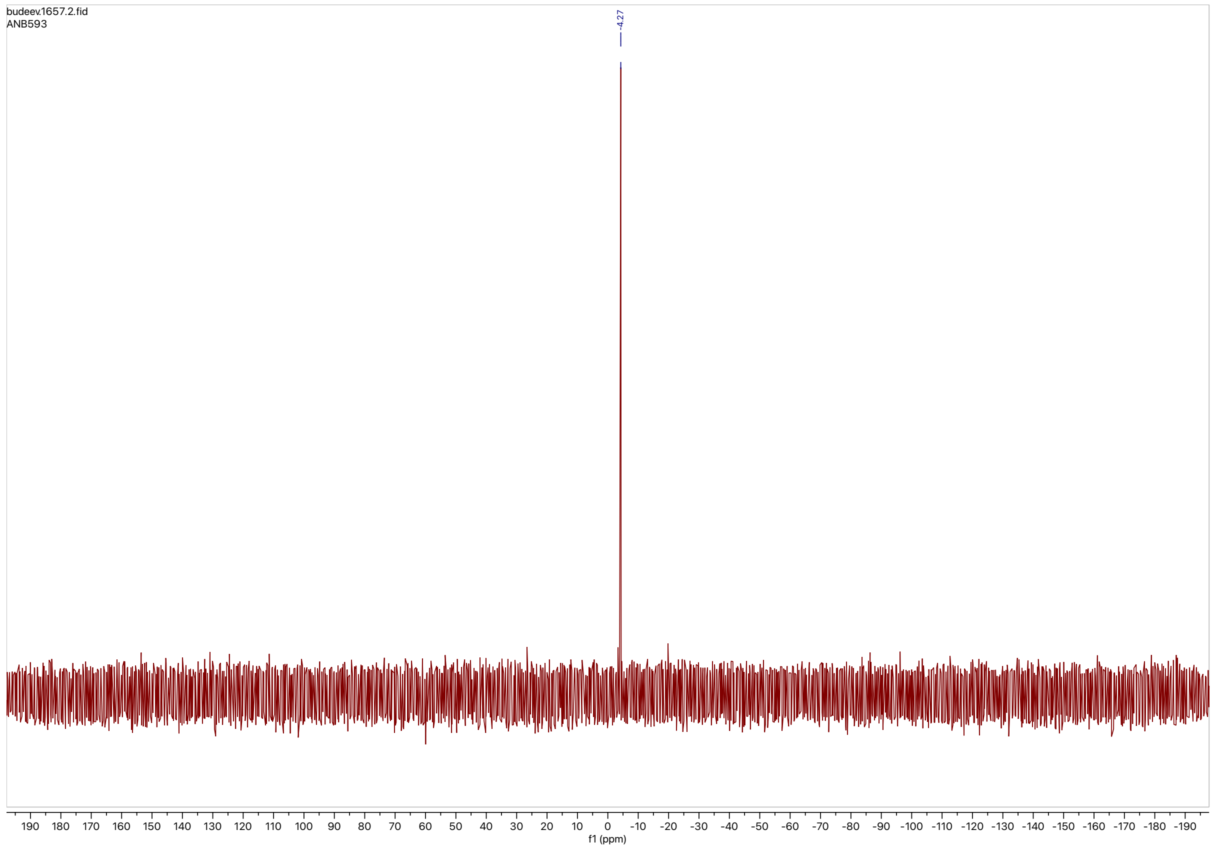

Supplementary Figure 82. ^{1}H (600 MHz, $CDCl_3$, 25 °C), $^{13}C\{^{1}H\}$ (151 MHz, $CDCl_3$, 25 °C), $^{19}F\{^{1}H\}$ (376 MHz, $CDCl_3$, 25 °C) and $^{31}P\{^{1}H\}$ (162 MHz, $CDCl_3$, 25 °C) spectra of (*cis*)-**2a**

Supplementary References

1. Kojima S., Kajiyama K., Nakamoto M., Matsukawa S. & Akiba K.-y. The Ligand-Exchange Process of P–H_{apical} Phosphoranes and the Thermal Formation and Pseudorotation of Anti-Apicophilic Spirophosphoranes. *Eur. J. Org. Chem.* **2006**, 218–234 (2006).
2. Connelly N. G., Damhus T., Hartshorn R. M. & Hutton A. T. in *Nomenclature of Inorganic Chemistry IUPAC Recommendations 2005* (Royal Society of Chemistry 2005), p. 175–193.
3. Lenormand H., Corcé V., Sorin G., Chhun C., Chamoreau L.-M., Krim L., Zins E.-L., Goddard J.-P. & Fensterbank L. Versatile Access to Martin's Spirosilanes and Their Hypervalent Derivatives. *J. Org. Chem.* **80**, 3280–3288 (2015).
4. Jiang X.-D., Kakuda K.-i., Matsukawa S., Yamamichi H., Kojima S. & Yamamoto Y. Synthesis and Application of a Bidentate Ligand Based on Decafluoro-3-phenyl-3-pentanol: Steric Effect of Pentafluoroethyl Groups on the Stereomutation of O-Equatorial C-Apical Spirophosphoranes. *Chem. Asian J.* **2**, 314–323 (2007).
5. Krylov I. M., Mailyan A. K., Zotova M. A., Bruneau C., Dixneuf P. H. & Osipov S. N. Access to Functionalized α-Trifluoromethyl-α-aminophosphonates via Intermolecular Ene–Yne Metathesis. *Synlett* **25**, 2624–2628 (2014).
6. Mei T.-S., Giri R., Maugel N. & Yu J.-Q. Pd^{II}-Catalyzed Monoselective *ortho* Halogenation of C–H Bonds Assisted by Counter Cations: A Complementary Method to Directed *ortho* Lithiation. *Angew. Chem. Int. Ed.* **47**, 5215–5219 (2008).
7. Hart D. J. & Tsai Y. M. *α*-Acylamino Radical Cyclizations: Syntheses of Isoretronecanol. *J. Am. Chem. Soc.* **106**, 8209–8217 (1984).
8. Brenninger C., Pöthig A. & Bach T. Brønsted Acid Catalysis in Visible-Light-Induced [2+2] Photocycloaddition Reactions of Enone Dithianes. *Angew. Chem. Int. Ed.* **56**, 4337–4341 (2017).
9. Fuerst D. E. & Jacobsen E. N. Thiourea-Catalyzed Enantioselective Cyanosilylation of Ketones. *J. Am. Chem. Soc.* **127**, 8964–8965 (2005).
10. Núñez M. G., Farley A. J. M. & Dixon D. J. Bifunctional Iminophosphorane Organocatalysts for Enantioselective Synthesis: Application to the Ketimine Nitro-Mannich Reaction. *J. Am. Chem. Soc.* **135**, 16348–16351 (2013).
11. Rozsar D., Formica M., Yamazaki K., Hamlin T. A. & Dixon D. J. Bifunctional Iminophosphorane-Catalyzed Enantioselective Sulfa-Michael Addition to Unactivated α,β-Unsaturated Amides. *J. Am. Chem. Soc.* **144**, 1006–1015 (2022).
12. Cortez N. A., Aguirre G., Parra-Hake M. & Somanathan R. New heterogenized C_2-symmetric bis(sulfonamide)-cyclohexane-1,2-diamine-$Rh^{III}Cp^*$ complexes and their application in the asymmetric transfer hydrogenation (ATH) of ketones in water. *Tetrahedron Lett.* **50**, 2228–2231 (2009).
13. Garimella P. D., Datta A., Romanini D. W., Raymond K. N. & Francis M. B. Multivalent, High-Relaxivity MRI Contrast Agents Using Rigid Cysteine-Reactive Gadolinium Complexes. *J. Am. Chem. Soc.* **133**, 14704–14709 (2011).
14. Anderson R. H., Lensing C. J., Forred B. J., Amolins M. W., Aegerter C. L., Vitiello P. F. & Mays J. R. Differentiating Antiproliferative and Chemopreventive Modes of Activity for Electron-Deficient Aryl Isothiocyanates against Human MCF-7 Cells. *ChemMedChem* **13**, 1695–1710 (2018).

15. Kajiyama K., Yoshimune M., Nakamoto M., Matsukawa S., Kojima S. & Akiba K.-y. Highly Selective One-Pot Synthesis of Spirophosphoranes Exhibiting Reversed Apicophilicity by Oxidation of Dianions Generated from P−H Spirophosphorane. *Org. Lett.* **3**, 1873–1875 (2001).
16. Rickhaus M., Jundt L. & Mayor M. Determining Inversion Barriers in Atropisomers – A Tutorial for Organic Chemists. *Chimia* **70**, 192–202 (2016).
17. Hesse M., Meier H. & Zeeh B. in *Spektroskopische Methoden in der Organische Chemie* (Georg Thieme Verlag 1995), p. 98–100.
18. Bourhis L. J., Dolomanov O. V., Gildea R. J., Howard J. A. K. & Puschmann H. The anatomy of a comprehensive constrained, restrained refinement program for the modern computing environment - *Olex2* dissected. *Acta Cryst. A* **71**, 59–75 (2015).
19. Dolomanov O. V., Bourhis L. J., Gildea R. J., Howard J. A. K. & Puschmann H. *OLEX2*: a complete structure solution, refinement and analysis program. *J. Appl. Cryst.* **42**, 339–341 (2009).
20. Sheldrick G. Crystal structure refinement with *SHELXL*. *Acta Cryst. C* **71**, 3–8 (2015).
21. Coppens P. in *Crystallographic Computing* (Ed. Ahmed F. R., Munksgaard 1970), p. 255–270.
22. Koziskova J., Hahn F., Richter J. & Kožíšek J. Comparison of different absorption corrections on the model structure of tetrakis(μ_2-acetato)-diaqua-di-copper(II). *Acta Chim. Slovaca* **9**, 136–140 (2016).
